# Adaptive attenuation of virulence mediated by Wzc mutation in ST11-KL47 Carbapenem-resistant *Klebsiella pneumonia*

**DOI:** 10.3389/fcimb.2025.1561631

**Published:** 2025-03-11

**Authors:** Yufeng Dai, Qiang Zhao, Huanhuan Yan, Kun Ye, Lifeng Wang, Ling Guo, Na Guo, Wenwen Li, Jiyong Yang

**Affiliations:** ^1^ Department of Laboratory Medicine, The First Medical Center of Chinese People's Liberation Army (PLA) General Hospital, Beijing, China; ^2^ Medical School of Chinese People's Liberation Army (PLA), Beijing, China

**Keywords:** CRKP, capsular polysaccharides, hypermucoviscosity, Wzc, virulence

## Abstract

**Introduction:**

The impact of the hypermucoviscosity (HMV) phenotype in ST11-KL47 carbapenem-resistant *Klebsiella pneumoniae* (CRKp) pathogenicity warrants investigation for public health risk assessment.

**Methods:**

We analyzed 230 clinical ST11-KL47 CRKp to identify the key factor in mucoviscosity acquisition via comparative genomic analysis. Sedimentation value served as the objective index to quantify HMV. The virulence *in vivo* was assessed using *Galleria mellonella* and mouse infection models. We employed genome engineering, capsular polysaccharides (CPS) quantification, and visualization to explore the role of Wzc mutation in CPS biosynthesis and HMV. The biological impact of Wzc-mediated HMV was investigated through competitive growth analysis, biofilm formation, serum resistance, anti-phagocytic ability, and adhesion assays. Transcriptomic analysis and scanning electron microscopy (SEM) were utilized to explore the relationship between polysaccharide composition, physical distribution, and changes in virulence.

**Results:**

The Wzc mutations are identified as the key to mucoviscosity acquisition. Unexpectedly, Wzc-mediated HMV CRKp exhibits reduced pathogenicity versus non-mucoviscosity (NMV) strains in different animal models, with competitive disadvantage, decreased biofilm formation, serum resistance, and adhesion, yet higher anti-phagocytic ability *in vitro*. CPS extraction and visualization of genome-engineered strains verify the Wzc mutations mediate HMV by standardizing CPS chain length and overproducing cell-free extracellular polysaccharides (cell-free EPS). Transcriptomic results, lipopolysaccharides (LPS) quantification, and SEM collectively indicate a downregulation of LPS synthesis and the masking of LPS in HMV strains.

**Discussion:**

These findings demonstrate that the Wzc-induced HMV attenuates ST11-KL47 CRKp virulence by modifying the exopolysaccharide composition and physical distribution.

## 1 Introduction

The rising global prevalence of Carbapenem-resistant *Klebsiella pneumoniae* (CRKp) poses a formidable challenge to antimicrobial therapy (Antimicrobial Resistance, 2022). In Asia, the dominant clones are sequence type (ST) 11-capsule type KL64 and KL47 CRKp (Zhang et al., 2017; Zhou et al., 2020). Genomically, ST11-KL64 evolved from ST11-KL47 by acquiring a pLVPK-like virulence plasmid harboring the *rmpADC/A2, iucABCD*, and *iutA* genes, which encode RmpADC/A2 and siderophore, or through chromosome region swapping that contains the capsular biosynthesis *locus* (Chen, 2023b; Zhou et al., 2023). These regulators enhance capsular polysaccharide (CPS) biosynthesis and contribute to hypermucoviscosity (HMV) phenotype formation, increasing the pathogenicity of *K. pneumoniae* (Yang X. et al., 2021; Zhou et al., 2023). However, many ST11-KL47 CRKps exhibit the HMV phenotype without these known factors, suggesting the existence of unknown determinants that influence HMV and virulence.

Historically, HMV was believed to overlap with CPS overproduction; decreased CPS levels resulted in the loss of HMV (Walker and Miller, 2020). However, recent studies have shown that HMV and CPS production are distinct yet coordinated traits (Mike et al., 2021). Miller et al. demonstrated that *rmpC* and *rmpD* affect CPS and HMV phenotypes, respectively. Notably, *rmpC* deletion strain retains HMV despite reduced CPS levels, while *rmpD* overexpression enhances HMV without increasing CPS (Walker et al., 2019; Ovchinnikova et al., 2023). CPS biosynthesis also relies on capsule-encoding genes on the chromosome (Dorman et al., 2018). The Wzc protein, a tyrosine auto-kinase encoded by the *wzc* gene, serves as a master regulator of CPS polymerization and translocation in various pathogenic bacteria (Shu et al., 2009). Its autophosphorylation can halt polymerization and close the outer membrane translocation of Wza, hindering CPS export (Yang Y. et al., 2021), while its dephosphorylation by tyrosine phosphatase Wzb allows CPS transport (Tiwari et al., 2020). This dynamic cycling of Wzc between phosphorylation and dephosphorylation regulates CPS production and export (Yang Y. et al., 2021). Recent research has shown that single nucleotide polymorphisms (SNPs) in *wzc* can increase CPS production (Ernst et al., 2020; He et al., 2023). Specifically, Wzc variants of K2 serotype *K. pneumoniae* influence the HMV phenotype via altering CPS chain length and increasing cell-free extracellular polysaccharides (EPS) (Khadka et al., 2023). Comprehending the regulatory interactions mediated by Wzc between CPS biosynthesis and HMV phenotype is vital for future molecular research.

HMV colony morphology is a critical strategy employed by pathogenic bacteria to invade host innate immunity and was previously recognized as a hallmark of hypervirulent *Klebsiella pneumoniae* (hvKp) (Rendueles and Velicer, 2017; Almeida et al., 2024). The loss of HMV has been proven to weaken virulence in various K types of *K. pneumoniae*, including K1, K2, K57, and K64 (Walker and Miller, 2020; Shoja et al., 2022; He et al., 2023; Liang et al., 2024). However, Mina et al. suggested that HMV *K. pneumoniae* may exhibit lower cell toxicity compared to non-mucoviscosity (NMV) strains (Mina et al., 2024). Additionally, EPS-positive strains have been found to cause milder sickness than EPS-negative strains in *Pseudomonas aeruginosa* pneumonia (Granton et al., 2024). These findings indicated that HMV has manifold biological functions and may lead to varying virulence outcomes. The impact of the HMV phenotype on the pathogenicity of ST11-KL47 CRKp is not yet fully understood, which limits the assessment of its public health threat.

In this study, we evaluated the capsule-related characteristics and conducted *in vitro* and *in vivo* experiments to elucidate the role of Wzc mutations in HMV phenotype formation and the pathogenicity of ST11-KL47 CRKp. We hypothesize that the Wzc-associated HMV phenotype may attenuate the virulence of ST11-KL47 CRKp by altering the composition and physical distribution of exopolysaccharides.

## 2 Materials and methods

### 2.1 Bacterial isolates and antimicrobial susceptibility testing

All strains of *K. pneumoniae* were stored in the First Medical Center of Chinese PLA General Hospital and identified by Vitek II (bioMérieux, Marcy-l’Étoile). The Minimal inhibitory concentrations (MICs) were determined by the Clinical and Laboratory Standards Institute (CLSI) broth microdilution method, with *Escherichia coli* ATCC 25922 as the quality control strain. Carbapenem-resistant *K. pneumoniae* (CRKp) strains showed resistance to at least one carbapenem. The hvKp reference stains NTUH-K2044 (K1), American Type Culture Collection (ATCC) 43816 (K2), and the cKp strain ATCC 700603 were used in selected experiments. Unless noted, all strains were recovered from frozen stocks and cultured in lysogeny broth (LB) medium or on LB agar plates at 37°C.

### 2.2 Sedimentation assay

Briefly, bacterial isolates were grown overnight in LB broth, and the optical density at 600 nm (OD_600_) of whole cultures and supernatants was measured before and following centrifugation at 1,000 *g* for 5 min. The ratio of the supernatant OD_600_ to the OD_600_ of the original culture was calculated as the sedimentation value, which was used to classify viscosity levels with > 0.4 as hypermucoviscosity, 0.2 to 0.4 as low viscosity, and < 0.2 as no viscosity (Walker and Miller, 2020). Each strain was tested in triplicate batches.

### 2.3 Whole-genome sequencing and analysis

Isolates were sequenced using a paired-end library with an average insert size of 350 base pairs (bp) and a read length of 150 bp on the Illumina NovaSeq platform. Fastp was used for the read quality assessment and filtering (Chen, 2023a). *de novo* assembly of genome was performed with ABySS (Jackman et al., 2017). The K serotype, multilocus sequence typing (MLST), O-locus classification, antibiotic resistance genes, and virulence genes were analyzed using Kleborate (Lam et al., 2021) and Kaptive (Lam et al., 2022). Reads were annotated using Prokka (version 1.13.7) and RAST (Seemann, 2014). A phylogenetic tree was constructed using kSNP4 for high-resolution phylogeny (Hall and Nisbet, 2023) and visualized with the online tool Tree Annotator software iTOL (http://itol.embl.de/). Single nucleotide polymorphisms (SNPs) in capsular genes were identified by mapping Illumina raw reads to the reference sequence for *K. pneumoniae* capsular polysaccharide biosynthesis gene cluster, type K47 (RefSeq: LT174558.1).

### 2.4 *Galleria mellonella* infection

Research-grade ***Galleria mellonella* larvae** *(*200–400 mg) were purchased from Tianjin Huiyude Biotech and used within 48 h. Overnight bacteria cultures were adjusted to an OD_600_ of 1, and diluted in sterile PBS. For each bacterial strain, ten **larvae** were infected with 10 µL of a bacterial suspension containing 1×10^6^ colony-forming units (CFUs) using a Hamilton syringe. The infected **larvae** were incubated at 37°C and monitored every 12 h for 48 h. Bacterial cultures were serially diluted and plated on LB agar plates at 37°C for 16-18 h to determine CFU count. Each strain was tested in triplicate batches.

### 2.5 Introduction of site mutations in *wzc*

To construct B1230-C (*wzc*_T1598C_^B1230^) and IR5061-M (*wzc*_C1598T_^IR5061^), a two-step allelic exchange was performed using the suicide vector pKOV-Kan. A 1383-bp fragment of the *wzc* gene (572-1954) from the respective strain B1230 or IR5061 was amplified with primers 01/04 (**Supplementary Table S1**) and assembled into the Rep101-dependent and *sacB*-harboring pKOV-Kan vector. The resulting constructs were electroporated into the respective B1230 or IR5061 strains. Successful transformants were selected on LB medium containing 100 μg ml^−1^ kanamycin (Kan) or 15% sucrose agar plates at different temperatures (30°C or 42°C). All desired mutants were confirmed by PCR using primers F0/R0 (**Supplementary Table S1**) followed by Sanger sequencing. The sequences were further confirmed using SnapGene software.

### 2.6 Mouse models of intraperitoneal infection

All vertebrate animal procedures were performed following the Regulations for the Administration of Affairs Concerning Experimental Animals and approved by the Animal Ethics Review Committee of Novel Science (Beijing) (project number NWAKLL-2024-10-015). Female BALB/c mice (6–8 weeks old) were purchased from SiPeiFu Laboratory Animal Technology (Beijing) and randomized into five groups (n=10 per group). Mice were infected intraperitoneally with 100 μL of bacterial suspension containing 1×10^8^ *K. pneumoniae* strains in the exponential growth phase, and their survival was monitored every 12 h for 3 days. Survival curves were generated using Prism 8. For histopathology, mice were infected intraperitoneally with 2×10^7^ *K. pneumoniae* strains and euthanized 24 h post-infection. Livers were collected for CFU counts and fixed in 4% formalin in PBS for 24 h, followed by histological slide preparation for hematoxylin and eosin (H&E) stain. The infection experiments were performed at the Biosafety Level 2 laboratory.

### 2.7 India ink staining

Briefly, bacterial colonies were suspended in PBS, and 20 μL of the bacteria suspension was mixed with a drop of India ink on a slide. The slide was then observed under a microscope with oil immersion at 100× magnification to visualize the capsules against the stained background. Images were representatives of three independent experiments.

### 2.8 Transmission electron microscopy and scanning electron microscope

Low voltage transmission electron microscopy (LVTEM) and field emission scanning electron microscopy (FESEM) were employed to observe bacterial capsules and extracellular matrix. For LVTEM, overnight bacterial clones grown on agar plates were carefully transferred to 1.5-mL polypropylene tubes and fixed in 2.5% glutaraldehyde at 4 °C overnight. For SEM, coverslips were placed on colonies growing on agar plates and incubated overnight. The coverslips were then directly fixed in 2.5% glutaraldehyde at 4°C overnight. Images were representatives of three independent experiments.

### 2.9 Capsule extraction and quantification

Capsules were extracted and quantified using a modified uronic acid assay (Wang et al., 2022; Khadka et al., 2023; Ovchinnikova et al., 2023). For total capsule polysaccharides quantification, 500 µL of the overnight bacterial LB cultures were mixed with 100 µL of 1% Zwittergent 3-14 detergent in 100 mM citric acid (pH 2.0) and heated at 50°C for 30 min with vortexing. After centrifugation at 14,000 rpm for 5 min, 250 µL of the supernatant was precipitated by 1 mL of absolute ethanol (final concentration 80%) at 4°C for 30 min. The samples were centrifuged and the supernatant was discarded. The dried precipitates were dissolved in 100 µL of distilled water and mixed with 600 µL of 12.5 mM sodium tetraborate in H_2_SO_4_ and boiled at 100°C for 5 min to hydrolyze the polysaccharides. After cooling, 10 µL of 0.15% 3-phenylphenol (Aladdin) was added, followed by a 15-min incubation. The OD_520_ was measured in a 96-Well microtiter plate. For cell-free EPS, ultra-pure water replaced the 1% Zwittergent 3-14 detergent. Uronic acid concentrations were determined using a glucuronic acid (Sigma-Aldrich) standard curve and results were expressed in micrograms per OD_600_. Each strain was tested in triplicates.

### 2.10 Capsular chain length analysis

Capsule SDS-PAGE was performed as described with modifications (Ovchinnikova et al., 2023). The extraction of total CPS and cell-free EPS followed the aforementioned protocol. Precipitated total CPS or EPS was solubilized in 100 µL 1× SDS loading buffer, incubated at 100°C for 10 min, and treated with proteinase K overnight at 55°C. Samples (10 µL) were loaded onto 6% acrylamide resolving gels (Bio-Rad) and run at 300 V for 4.5 h at 4°C. After electrophoresis, the gels were washed five times in ultrapure water, and stained with 0.1% alcian blue in solution comprising 40% ethanol and 20 mM sodium acetate (pH 4.75) for 1 h with rocking. After de-staining gels overnight to minimize the background, gels were stained with Pierce Silver Stain Kit (Thermo Scientific) and imaged using an imager (Bio-rad). Each strain was tested in triplicate.

### 2.11 Growth curves and competitive growth assay

Growth rate and competitive growth assays were conducted in an antibiotic-free environment as previously described (Hu et al., 2024). Briefly, for the growth rate assay, bacterial strains were grown overnight in LB broth at 37°C. Cultures were adjusted to an OD_600_ of 1.0, and 10 μL of each culture was inoculated into 2 mL fresh LB broth and cultures at 37 °C for 24 h, with OD_600_ measurements taken every 4 h. In the competitive growth assay, 10 μL of the wild-type strain and isogenic mutant were co-inoculated in 2 mL of fresh LB broth and cultured at 37°C for 24 h. The cultures were then serially diluted and plated on LB agar plates, and the ratio of mutant to wild-type was calculated to determine the competitive index.

### 2.12 Biofilm assay

Biofilm assays were performed as previously described (Hu et al., 2024) with slight modifications. Briefly, stationary-phase cultures (OD_600_ = 0.4) were diluted 10-fold and transferred to a 96-well plate. After incubating at 37°C for 24 h, the plate was washed with PBS, and biofilms were fixed with 100 μL of methanol. After air-drying, biofilms were stained with 100 μL of 0.1% crystal violet for 5 min. Excess dye was removed with PBS washes. Crystal violet was then solubilized by incubating with 100 μL of ethanol for 30 min, and the OD_595_ of solubilized crystal violet was measured. The LB broth without bacteria served as the negative control. The results are presented based on at least three biological replicates.

### 2.13 Macrophage phagocytosis assay

The murine macrophage cell line Raw 264.7 was cultured in Dulbecco’s modified Eagle’s medium (DMEM) with 10% fetal bovine serum (Gibco). Bacterial phagocytosis assays were performed as previously described (He et al., 2023). Raw 264.7 cells were seeded into 24-well plates at 2.5 × 10^5^ cells per well and cultured overnight before infection at a multiplicity of infection (MOI) of 100 for 1 h and incubated at 37°C with 5% CO_2_. Post-infection, cells were washed with ice-cold PBS and treated with 100 μg ml^−1^ kanamycin containing DMEM for 1 h to eliminate extracellular bacteria. Cells were then gently washed with PBS and lysed with 0.2% Triton X-100 (Sigma Chemical) for 10 min. Bacterial uptake and intracellular phagocytosis rates were assessed by counting CFU on LB agar plates. The results are presented based on at least three biological replicates, each with three technical replicates. For visualization, bacteria were labeled with pHrodo red (Thermo Scientific) and the infection procedure was identical to the initial assay (Wang et al., 2024). After kanamycin treatment, cells and bacteria were fixed with 4% paraformaldehyde and then blocked with 1% BSA. Actin-Tracker Green (Beyotime) and DAPI (Beyotime) were used to stain cells. Images were representatives of three independent experiments.

### 2.14 Human lung epithelial cell adhesion assay

The human lung epithelial cell line A549 was used for the adhesion assay. The same protocol as the macrophage phagocytosis assay was followed, except without antibiotic treatment. Results were presented based on at least three biological replicates, each consisting of three technical replicates.

### 2.15 Serum killing assay

Bacteria cultures were adjusted to 1 × 10^7^ CFU/mL. A 25 μL aliquot of the suspension was added to 75 μL of pre-warmed human serum (Sigma, H4522). Heat-inactivated serum (56°C for 30 min) was served as a negative control. Viable bacterial counts were determined at 0, 60, 120, and 180 min post-incubation at 37°C. The mixtures were diluted and plated on LB agar plates for overnight culture to assess bacterial survival. Survival rates were calculated by comparing viable counts from active and heat-inactivated sera.

### 2.16 Limulus amebocyte lysate test

Briefly, 100 µL of bacterial suspension (1 ×10^4^ CFU/mL) was mixed with 400 µL of standard dilutions and incubated at 75°C for 10 min. After a 5-minute cooling period, 100 µL of sample was mixed with 50 µL of LAL reagent and incubated for 75 min. The absorbance was read at 405 nm. Each test included positive and negative controls, and all steps were performed in endotoxin-free conditions.

### 2.17 LPS extraction and visualization

LPS was extracted following the method of Granton and Brown (Granton et al., 2024). Briefly, overnight bacterial cultures were washed and resuspended in PBS to an OD_600_ of 0.4. After centrifugation, cell pellets were lysed in 100 μL of lysis buffer at 100°C for 10 min. Samples were then digested with proteinase K (Sigma-Aldrich) at 60°C for 1 h and prepared in 1× SDS loading buffer, separated via SDS-PAGE for 45 min at 150 V, and visualized using silver staining. Molecular weight was determined using a PageRuler™ Plus ladder (Thermo Fisher Scientific, 26619).

### 2.18 RNA sequencing analysis

RNA sequencing was performed as previously described (Hu et al., 2024). B1230 and B1230-C were grown in LB to mid-exponential phase at 37°C. Bacteria were harvested and total RNAs were extracted (QIAGEN, 74104). RNA sequencing was performed at the Beijing Novogene Biotechnology (Beijing, China). The Kyoto Encyclopedia of Genes and Genomes (KEGG) pathways functional analysis was performed on differentially expressed genes by cluster profile. *P*< 0.05 was defined as a significant difference. Data represents three biological replicates of each strain.

### 2.19 Data analysis

Statistical analysis was performed using GraphPad Prism 8 software. *P*-values were calculated using unpaired *t*-tests for comparison between two groups or one-way ANOVA for multiple groups. Mouse/*Galleria mellonella* larvae survival rates were determined using Kaplan–Meier survival analysis with a log-rank test. Statistical significance was determined as follows: ns *P* ≥ 0.05, *P < 0.05, **P < 0.01, ***P < 0.001, and ^#^ *P*<0.0001.

## 3 Results

### 3.1 Wzc Mutations: Key to mucoid phenotype acquisition and virulence diversity in ST11-KL47 CRKp

**Figure 1:**
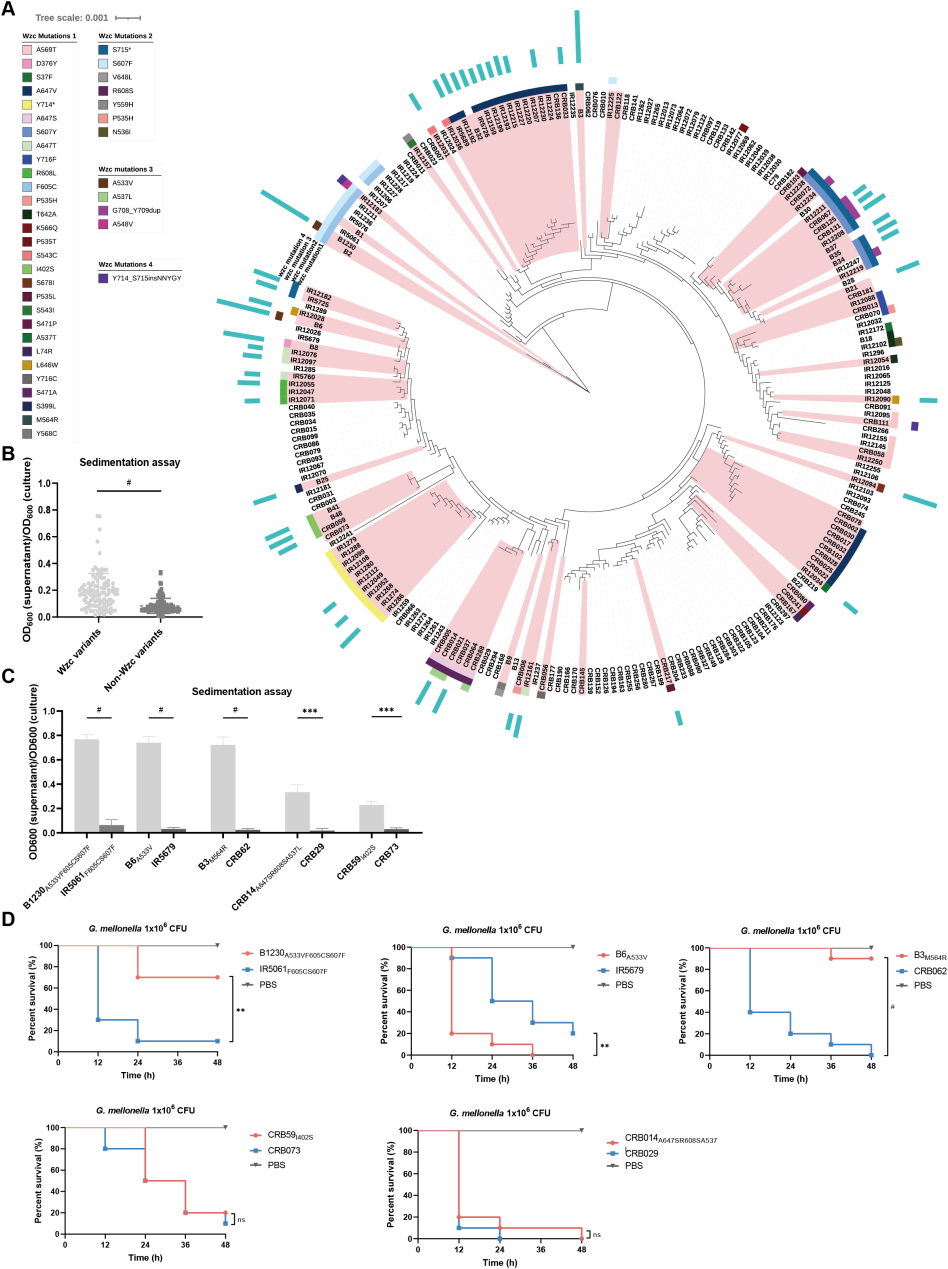
The genetic diversity of Wzc, the HMV-related and virulence-related characteristics of ST11-KL47 CRKp. **(A)** Phylogenetic tree based on genomic sequences of 230 ST11-KL47 CRKp. The Wzc mutations of stains are mapped to the phylogenetic tree. Red branches mark the mucoid strains, and the length of the blue strip represents the level of sedimentation values (sedimentation value <0.2 not shown). **(B)** Sedimentation values between Wzc variants group (n=108) and Non-Wzc variants group (n=122). Each point represents the mean value of three biological replicates of one strain. *P* < 0.0001. ^#^ *P* < 0.0001; Unpaired two-sided Welch’s *t*-test. **(C)** Sedimentation assay of five pairs ST11-KL47 CRKp for mucoviscosity quantification. n = 3 biological replicates, bars indicate mean ± SD. *P* values from left to right: < 0.0001, < 0.0001, < 0.0001, 0.0009, 0.0003. ***P < 0.001, ^#^ *P* < 0.0001; two-tailed Student’s *t*-test. **(D)** Survival rate of *Galleria mellonella* larvae. *Galleria mellonella* larvae were inoculated with 1×10^6^ CFU of strains and the Kaplan–Meier survival curves were recorded. n = 10/group. The significance is calculated using the log-rank test. *P* values from left to right: 0.0012, 0.0038, <0.0001, 0.5544, 0.3779. ns, not significant, **P < 0.01, ***P < 0.001, ^#^ *P* < 0.0001.

To analyze the diversity and mutation patterns of Wzc in ST11-KL47 CRKp, we constructed a phylogenetic tree based on the genomic sequences of 230 non-duplicate isolates from a tertiary hospital in China. Mutations in the Wzc were present in 47.39% (109/230) of these CRKp isolates, distributed across 29 distinct sites with 40 different types. Some Wzc-mutated isolates clustered together (**Figure 1A**). The most common mutation type was missense point mutation, the most frequent site being position 647 (30.48%, 32/105), followed by position 607 (23.81%, 25/105) (**Supplementary Figure S1A**). A similar trend was observed in a broader set of 2517 ST11-KL47 *K. pneumoniae* genomes from the NCBI GenBank, where Wzc mutations appeared frequently (30.99%, 780/2517). Missense mutations (90%, 702/780) were the most common mutational events, notably at position 607 (6.79%, 53/780), followed by position 647 (6.54%, 51/780) (**Supplementary Figure S1A**). Out of the 29 mutation sites, 86.21% (25/29) occurred within the cytoplasmic domain (amino acids (aa) 448-722) (**Supplementary Figure S1B**), suggesting a hypervariable region. Specifically, 51.72% (15/29) of these mutation sites were located within the ATPase activity domain (AAA domain, aa 537-649). These findings suggest that Wzc mutations are frequent and diverse in ST11-KL47 CRKp, potentially impacting biological functions and enhancing dissemination.

We examined the colony morphology of 230 strains on blood agar and assessed the mucoviscosity via sedimentation assay (**Supplementary Table S2**). Most mucoid phenotypes (92%, 92/100) possessed Wzc mutations. A significant difference in sedimentation values was observed between strains with and without Wzc mutations (**Figure 1B**). Interestingly, only 25% of mucoid strains (25/100) harbored truncated *rmpA2*, and a higher sedimentation value was found in the non-truncated *rmpA2* harboring group (**Supplementary Figure S1C**). Notably, CRB078, carrying a pNDM-Mar plasmid (a hybrid conjugative plasmid harbored *iucABCD*, *rmpADC*, *rmpA2*, and siderophore gene clusters), yet did not show strong sedimentation (**Supplementary Table S2**), indicating that virulent plasmids may not be pivotal in sedimentation in ST11-KL47 *K. pneumoniae*. These results suggest that *wzc* mutations, rather than the virulent plasmids or truncated *rmpA2*, are the primary and positive strategy contributing to the mucoid phenotype in ST11-KL47 CRKp.

We further selected ten strains devoid of *rmpA2* or virulent plasmids, including three hyper-mucoid strains (sedimentation value >0.4) and two low-mucoid strains (sedimentation value 0.2 to 0.4) with Wzc mutations, alongside their NMV strains (sedimentation value <0.2) based on phylogeny (**Figure 1C**). Using *Galleria mellonella* larvae mortality as the objective index, we assessed the virulence of ten strains *in vivo*. Intriguingly, HMV strains B1230_A533VF605CS607F_ and B3_M564R_ exhibited significantly reduced virulence compared to their NMV counterparts (**Figure 1D**). This suggests that the Wzc-associated HMV phenotype and high sedimentation values may contribute to virulence attenuation in ST11-KL47 CRKp, which contrasts with findings in other K-types (Mike et al., 2021).

### 3.2 Wzc_A533V_-mediated HMV attenuates the virulence of ST11-KL47 CRKp

To investigate how the Wzc mutation attenuates virulence in ST11-KL47 CRKp, we compared the HMV strain B1230 (short for B1230_A533VF605CS607F_) and its counterpart NMV strain IR5061 (short for IR5061_F605CS607F_). B1230 had the highest sedimentation value among the 230 strains and exhibited reduced virulence. B1230 and IR5061 possessed similar HMV- and virulence-related traits. Neither harbored large virulence plasmids, integrative conjugative elements, nor *rmpADC/A2* on their chromosomes (**Supplementary Table S3**). There were 46 single nucleotide polymorphisms (SNPs) in total between B1230 and IR5061 (32 nonsynonymous, 14 synonymous) (**Supplementary Table S4**), implying that they were closely related in phylogeny and derived from the same clone. The sole missense variant in the *cps* biosynthesis gene cluster was the Wzc_A533V_ caused by C1598T substitution in *wzc*.

**Figure 2:**
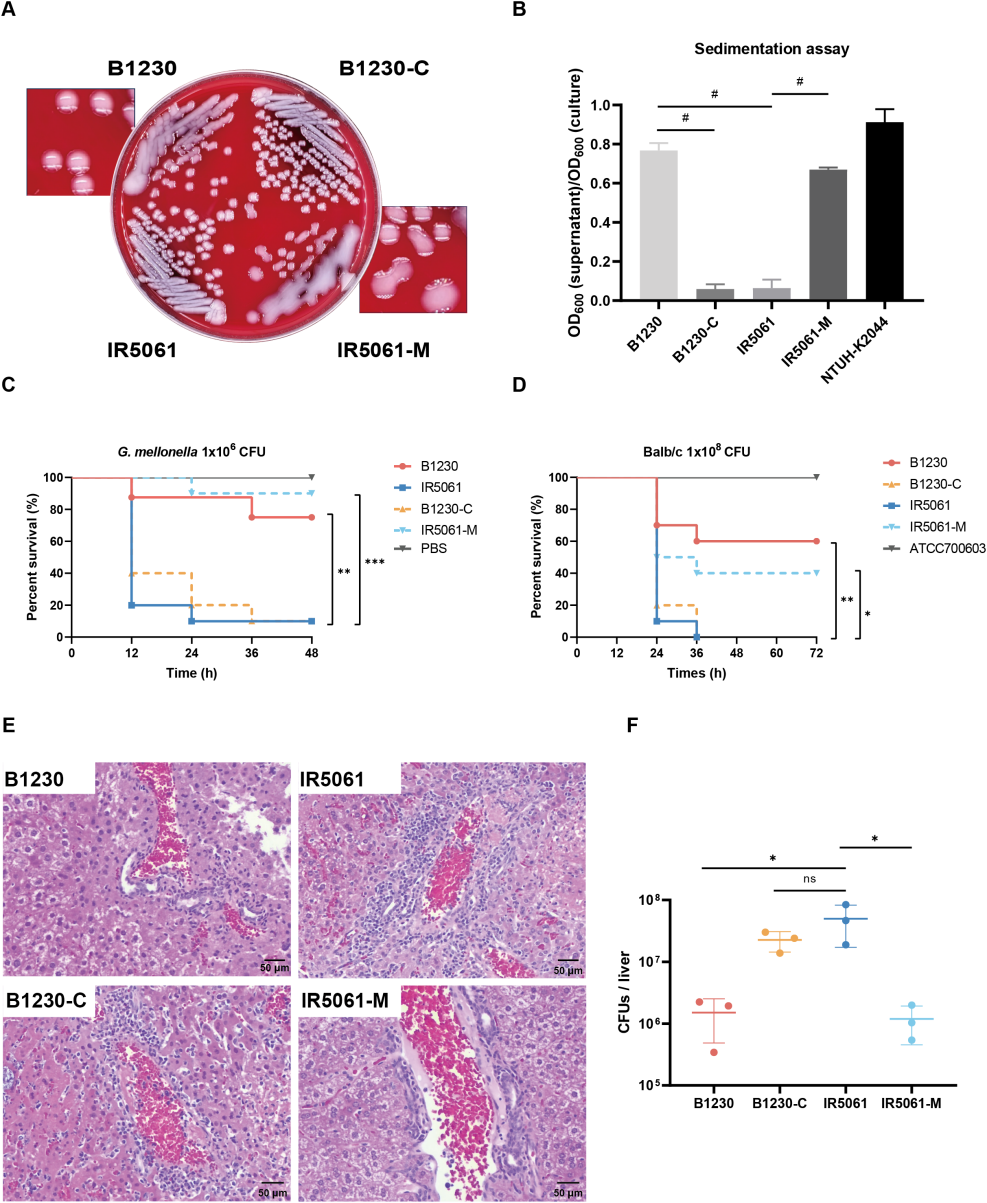
HMV-related and virulence-related characteristics of B1230, IR5061, and the corresponding mutants. **(A)** Colony morphology of B1230, IR5061, and the corresponding mutants on Columbia blood agar plate. **(B)** Sedimentation assay for mucoviscosity quantification. n = 3 biological replicates, bars indicate mean ± SD. *P* < 0.0001. ^#^ *P* < 0.0001; one-way ANOVA test with *post-hoc* Tukey’s multiple comparisons. **(C)** Survival rate of *Galleria mellonella* larvae. *Galleria mellonella* larvae were inoculated with 1×10^6^ CFU of B1230, IR5061, and corresponding mutants, and the Kaplan–Meier survival curve was recorded. n = 10/group. The significance was calculated using the log-rank test. *P* values from left to right: 0.0025, 0.0037, 0.0002. **P < 0.01, ***P < 0.001. **(D)** Survival rate of mice. BALB/c mice were intraperitoneally inoculated with 1×10^8^ CFU of ATCC700603, B1230, IR5061, and corresponding mutants, and the Kaplan–Meier survival curve was recorded. n = 10/group. The significance was calculated using the log-rank test. *P* values from left to right: 0.0018, 0.0032, 0.0206. *P < 0.05, **P < 0.01. **(E)** Representative histological images of haematoxylin and eosin (H&E) stained liver tissue sections from mice at 24 hpi (hour post infection). BALB/c mice were intraperitoneally inoculated with 2×10^7^ CFU of PBS, B1230, IR5061, and corresponding mutants. n = 3/group. **(F)** Bacterial load in liver. The burdens of B1230, IR5061, and corresponding mutants in the liver from mice at 24 hpi were counted by serial dilution on LB agar plates. n = 3/group, bars indicate mean ± SD, lines indicate median value. Adjusted *P* values from left to right: 0.0325, 0.2722, 0.0314. *P < 0.05; one-way ANOVA test with *post-hoc* Tukey’s multiple comparisons.

To confirm the role of Wzc_A533V_ in mediating the HMV phenotype, we engineered two strains: B1230-C (B1230 complemented with a *wzc*_T1598C_ mutation) and IR5061-M (IR5061 with the same *wzc*_C1598T_ mutation). On blood agars, B1230-C lost its mucoid phenotype, while IR5061-M gained a mucoid phenotype (**Figure 2A**). In the sedimentation assay, IR5061-M displayed increased mucoviscosity, while B1230-C showed a significantly reduced mucoviscosity (**Figure 2B**). The findings indicate that the Wzc_A533V_ mutation is responsible for the HMV phenotype and increased sedimentation resistance, independent of known virulent plasmids and *rmpADC/A2 loci*.

In the *G. mellonella* larvae model, when the Wzc_A533V_ mutation was reversed in HMV strain B1230, the virulence of B1230-C increased to match that of NMV strain IR5061 (**Figure 2C**). This suggests that the Wzc_A533V_ mutation-mediated HMV attenuates the virulence of CRKp in the *G. mellonella* larvae model. Considering the differences in virulence levels in different animal models, we infected Balb/c mice intraperitoneally with 1 × 10^8^ CFU B1230, IR5061, and their engineered counterparts. As a control, we used a classical *K. pneumoniae* strain, ATCC 700603, which caused no mouse mortality within 3 days of infection (**Figure 2D**). Mice infected with B1230 and IR5061-M showed lower mortality compared to those infected with IR5061, while B1230-C exhibited significantly higher virulence compared to its parental strain B1230 (**Figure 2D**).

Given the role of hvKp in liver abscesses (PLA), we also examined liver tissue damage 24 h after intraperitoneal infection with 2 × 10^7^ CFU of CRKp. Liver infected with IR5061 and B1230-C showed increased inflammation compared to those infected with B1230 and IR5061-M after 24 h of infection (**Figure 2E**). Additionally, bacterial loads in the liver were significantly higher in IR5061-infected mice than those infected with B1230 (**Figure 2F**), indicating that NMV strains confer greater virulence and infectivity than HMV strains. These results verify that the Wzc_A533V_-mediated HMV phenotype attenuates virulence in ST11-KL47 CRKp across different animal models, though the specific mechanism remains unknown.

### 3.3 Wzc_A533V_ alters CPS spatial distribution and chain length to acquire HMV phenotype in ST11-KL47 CRKp

Since the HMV phenotype and sedimentation resistance are correlated with the thickness and state of the CPS, which participates in various bacterial pathogenesis processes, we analyzed how the Wzc_A533V_ mutation affects CPS formation.

**Figure 3:**
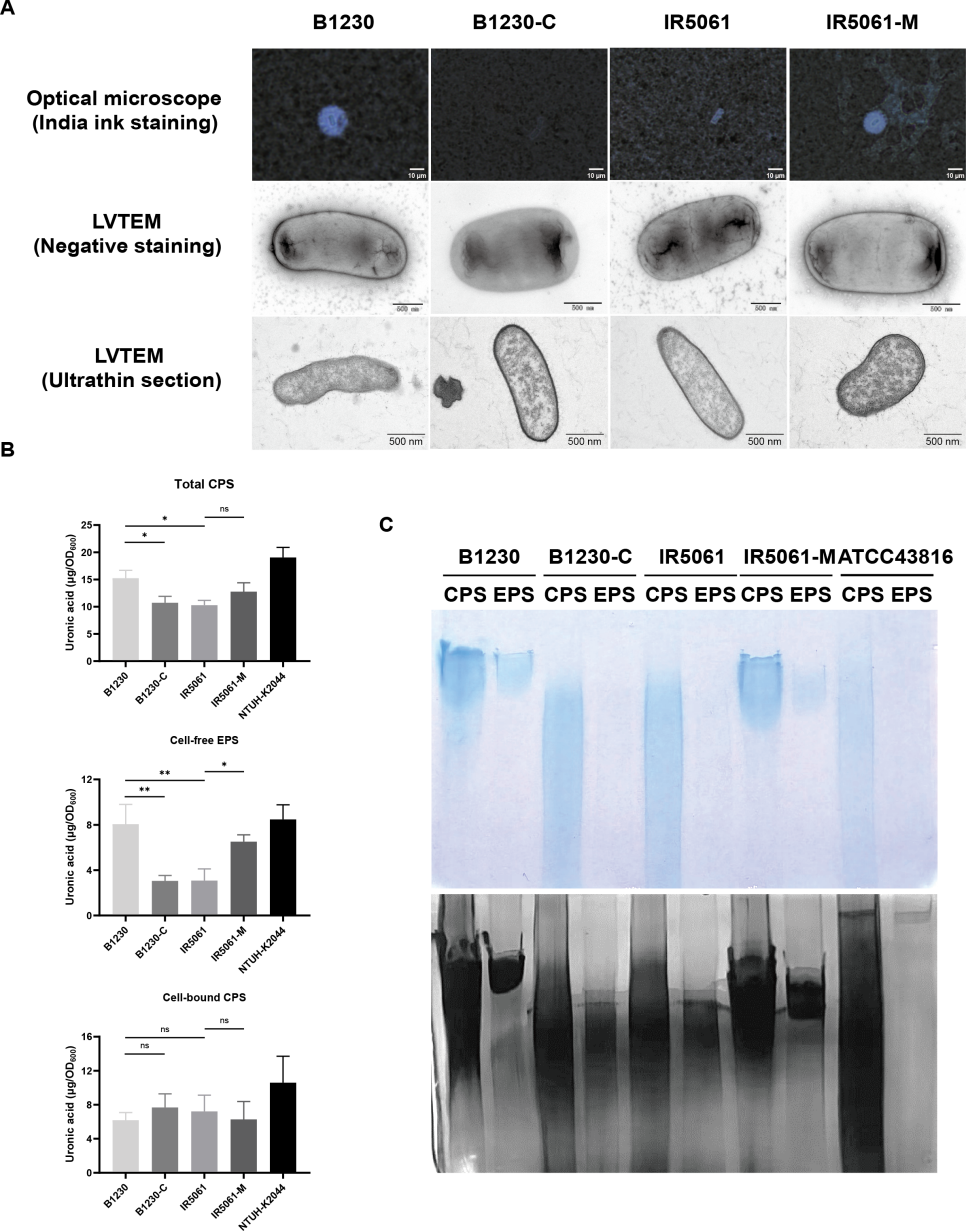
Capsule heterogeneity of B1230, IR5061, and the corresponding mutants. **(A)** Representative India ink staining (100× magnification) images and TEM images of B1230, IR5061, and the corresponding mutants. Images are representatives of three independent experiments. **(B)** Uronic acid measure for capsule polysaccharide (CPS) quantification. n = 3 biological replicates, bars indicate mean ± SD. *P* values from left to right: 0.0187, 0.0294, 0.2768, 0.0022, 0.0021, 0.0195, 0.8740, 0.7134, 0.8995. ns, not significant, *P < 0.05, **P < 0.01; one-way ANOVA test with *post-hoc* Tukey’s multiple comparisons. **(C)** Alcian blue staining (up) and silver staining (down) of total CPS (CPS) and cell-free EPS (EPS) resolved on SDS-PAGE. Images are representatives of three independent experiments.

We first visualized the capsule of the four strains via India ink staining assay and LVTEM. Significant differences were observed in capsule abundance and architecture between strains. HMV strains B1230 and IR5061-M, showed much larger non-stained capsular halos in India ink staining than NMV strains B1230-C and IR5061 (**Figure 3A**). LVTEM also revealed that B1230 and IR5061-M displayed more extracellular matrix and a denser capsular layer with thick, slime-like filaments surrounding the bacteria than IR5061 and B1230-C (**Figure 3A**). We then quantified CPS by measuring uronic acid levels and categorized it into the cell-bound CPS and the cell-free EPS. HMV B1230 produced slightly more total uronic acid than IR5061 (**Figure 3B**). However, a substantially higher level of cell-free EPS was generated in B1230 than in IR5061. The Wzc_A533V_ mutation in IR5061-M increased cell-free EPS to levels similar to B1230 (**Figure 3B**). Surprisingly, no substantial differences in cell-bound CPS were observed among B1230, IR5061, and their counterparts (**Figure 3B**). These findings suggest that the HMV phenotype is primarily driven by the accumulation of cell-free EPS induced by Wzc_A533V_ mutation rather than the overproduction of cell-bound CPS. We further analyzed CPS chain length distribution using SDS-PAGE and polysaccharide silver staining. B1230 and IR5061-M showed a distinct polysaccharide band with uniform molecular weight, while IR5061 and B1230-C displayed a heterogeneous “smear” with varying chain lengths (**Figure 3C**), showing that the Wzc_A533V_ mutation leads to a more standardized CPS chain length. The CPS profile of the reference K2 serotype *K. pneumoniae* ATCC 43816 showed a similar uniform high-molecular-weight polysaccharide band, consistent with previous findings (Ovchinnikova et al., 2023). These findings were further confirmed in four additional strain pairs (**Supplementary Figure S2**), confirming that the Wzc mutation specifically promotes HMV through defined CPS chain length distribution and the accumulation of cell-free EPS.

### 3.4 Effects of Wzc_A533V_ on bacteria fitness of ST11-KL47 CRKp and host interactions

HMV and CPS are known to significantly affect bacterial fitness and cellular processes. We, therefore, investigated the impact of Wzc_A533V_-induced HMV on bacterial fitness and host interactions by analyzing key properties involved in *K. pneumoniae* survival, including growth dynamics, biofilm formation, macrophage phagocytosis, epithelial cell adhesion, and serum-killing resistance.

**Figure 4:**
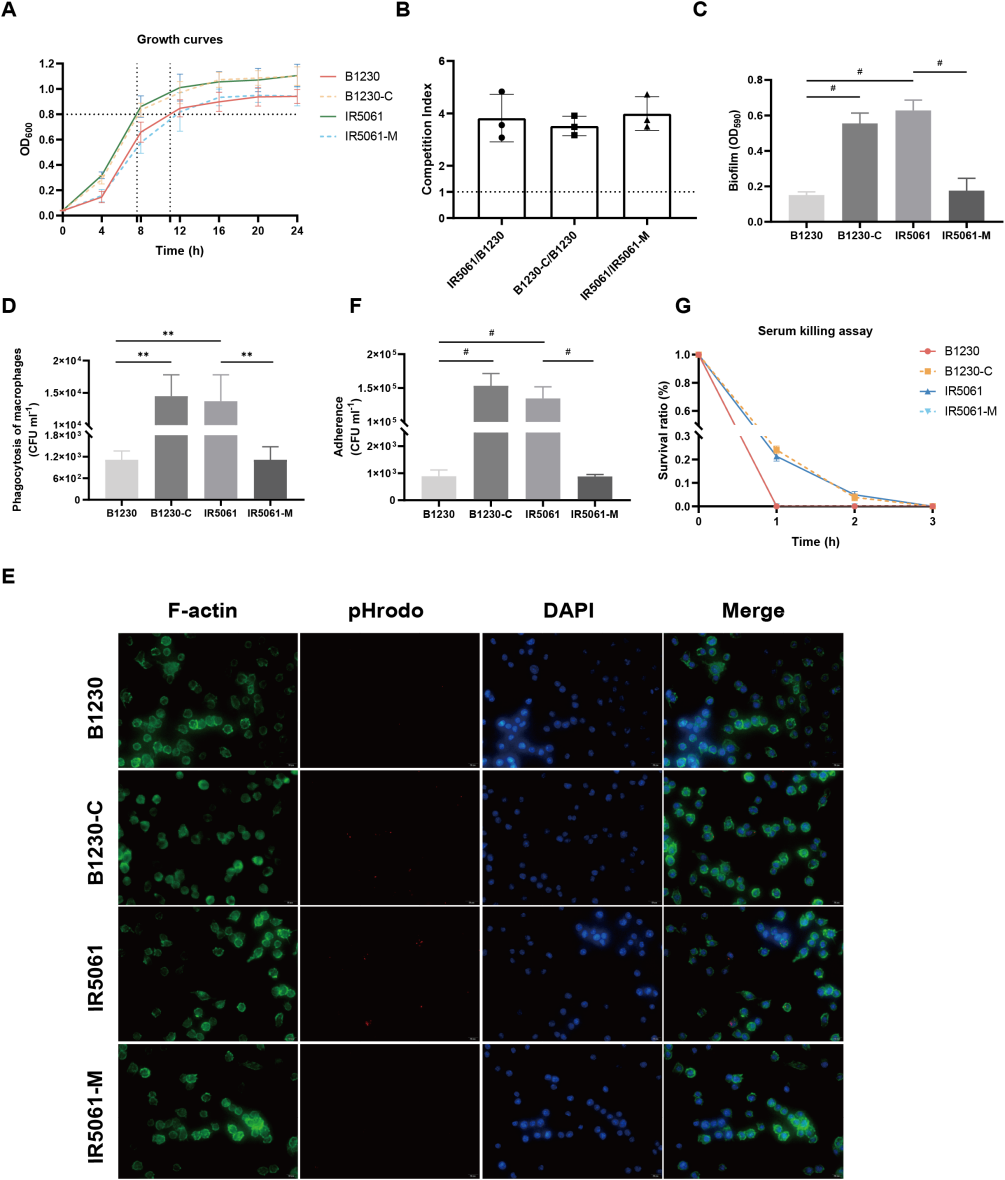
Effects of WzcA533V on bacteria fitness of ST11-KL47 CRKp and host interactions. **(A)** Growth curve of the B1230, IR5061, and the corresponding mutants. **(B)** Pair-wise competition assays. **(C)** Biofilm formation. n = 3 biological replicates, bars indicate mean ± SD. *P* values from left to right: <0.0001, <0.0001, <0.0001. ^#^ *P* < 0.0001; one-way ANOVA test with *post-hoc* Tukey’s multiple comparisons. **(D)** Phagocytosis of B1230, IR5061, and corresponding mutants by RAW 264.7 macrophages. n = 3 biological replicates, bars indicate mean ± SD. *P* values from left to right: 0.0018, 0.0012, 0.0018. **P < 0.01; one-way ANOVA test with *post-hoc* Tukey’s multiple comparisons. **(E)** Representative fluorescence microscopy images of the survival of B1230, IR5061, and corresponding mutants within macrophages. Images are representatives of three independent experiments. **(F)** Cell adhesion of B1230, IR5061, and corresponding mutants to A549 lung epithelial cells. n = 3 biological replicates, bars indicate mean ± SD. *P* values from left to right: <0.0001, <0.0001, <0.0001. ^#^ *P* < 0.0001; one-way ANOVA test with *post-hoc* Tukey’s multiple comparisons. **(G)** Survival in human serum.

The growth curves of the isolates revealed that HMV strains B1230 and IR5061-M grew more slowly in LB media compared to the NMV strains IR5061 and B1230-C (**Figure 4A**). B1230 required 11 h to reach an OD_600_ of 0.8, whereas IR5061 achieved the same growth in 7.6 h, suggesting that NMV strain IR5061 had a growth advantage and lower fitness cost. In competitive growth experiments, NMV strain IR5061 also showed a competitive advantage over HMV strain B1230. The complementary strain B1230-C, which lacks the Wzc-mediated HMV phenotype, restored competition levels similar to IR5061 (**Figure 4B**), suggesting that eliminating cell-free EPS synthesis and HMV phenotype might reduce metabolic burden. We applied the crystal violet staining to evaluate the biofilm formation capacity of those strains. B1230 and IR5061-M with HMV phenotype exhibited lower biofilm formation ability while the NMV strains IR5061 and B1230-C showed higher biofilm production (**Figure 4C**), suggesting a greater colonization ability among NMV strains.

Consistent with the previous studies in hvKp genetic backgrounds (Mike et al., 2021; He et al., 2023), the CRKp strains B1230 and IR5061-M with HMV phenotype demonstrated a strong resistance to phagocytosis by RAW264.7 macrophages. In contrast, the NMV strains IR5061 and B1230-C were efficiently engulfed (**Figure 4D**). Fluorescence microscopy further confirmed that NMV strains IR5061 and B1230-C were readily engulfed by macrophages, while HMV strains B1230 and IR5061-M resisted phagocytosis (**Figure 4E**). A similar trend was observed in the adhesion assay. NMV strains IR5061 and B1230-C strongly adhered to human lung epithelial A549 cells, whereas the HMV strains B1230 and IR5061-M had reduced cell association (**Figure 4F**). These findings provided evidence that the HMV phenotype enables *K. pneumoniae* to evade host immune responses by impairing bacterial association with host cells and blocking phagocytosis. This evasion likely increases the clinical risk of persistent infection, potentially contributing to the high dissemination rate of these strains.

We then tested whether Wzc_A533V_-induced cell-free EPS offered protection against complement-mediated serum killing. Unexpectedly, the B1230 and IR5061-M mutants were significantly more susceptible to serum-mediated killing after incubation in human active serum (**Figure 4G**). This indicates that Wzc mutation-induced cell-free EPS did not offer protection from serum-mediated lysis. Instead, it may have assisted complement-mediated killing. These findings were also confirmed in four additional strain pairs (**Supplementary Figure S3**), revealing a trend contrary to previous observations in hvKp genetic background (Mike et al., 2021).

### 3.5 Wzc-induced cell-free EPS may attenuate virulence by modifying the composition and physical distribution of exopolysaccharides

**Figure 5:**
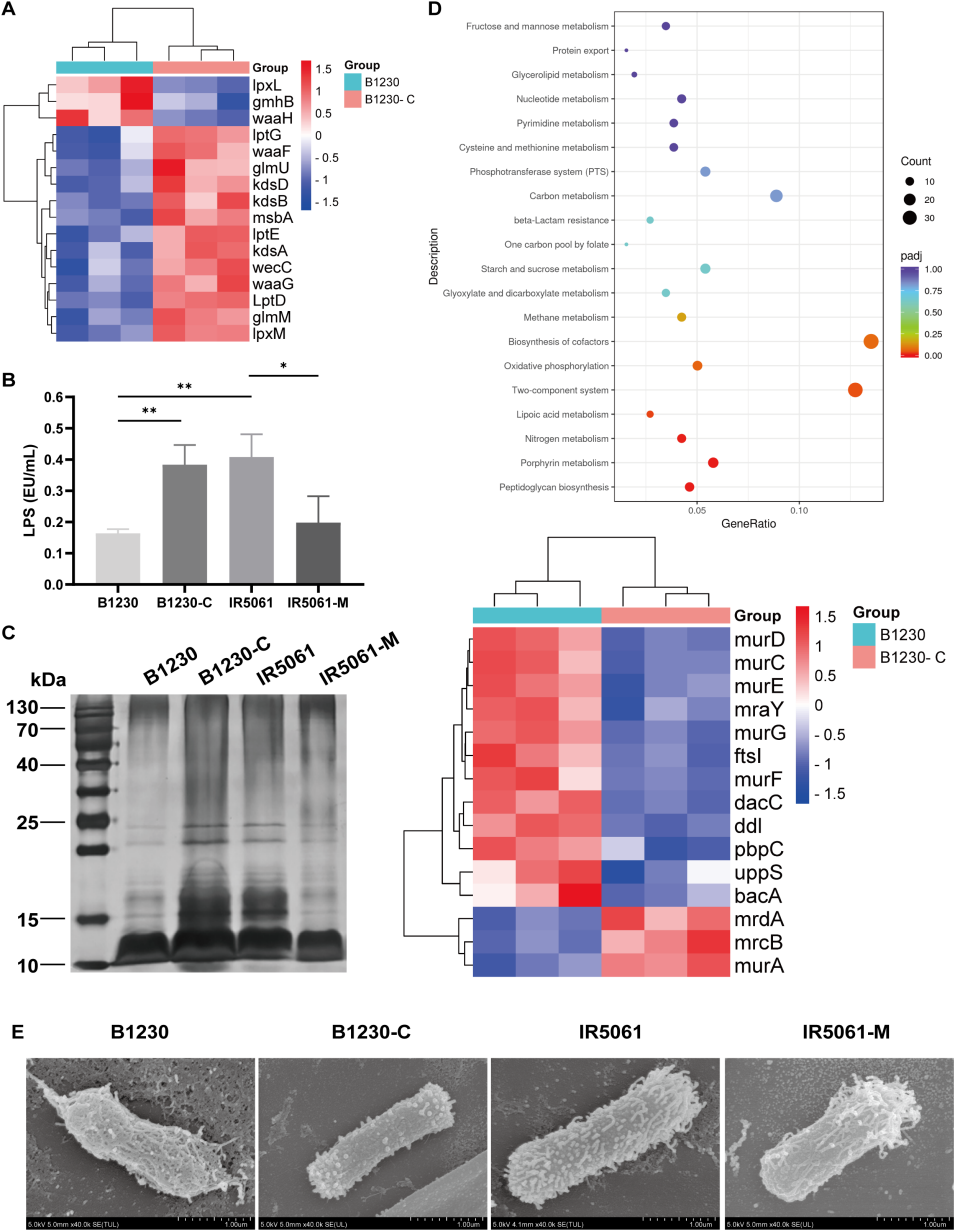
The exopolysaccharide composition and physical distribution of B1230, IR5061, and corresponding mutants. **(A)** Transcriptomic analysis of the differential expression of LPS synthesis genes between HMV B1230 and NMV B1230-C. Data represents three biological replicates of each strain. *P*< 0.05 was defined as a significant difference. **(B)** LPS content. n = 3 biological replicates, bars indicate mean ± SD. *P* values from left to right: 0.0047, 0.0041, 0.0312. *P < 0.05, **P < 0.01; one-way ANOVA test with *post-hoc* Tukey’s multiple comparisons. **(C)** LPS silver staining. **(D)** KEGG pathway enrichment analysis of upregulated genes of B1230 compared to B1230-C. Data represents three biological replicates of each strain. *P*< 0.05 was defined as a significant difference. **(E)** Representative SEM images of B1230, IR5061, and the corresponding mutants. Images are representatives of three independent experiments.

Alongside CPS, lipopolysaccharides (LPS) and peptidoglycan are essential glycan polymers on cell envelope that contribute to bacteria fitness and pathogenesis. CPS, LPS, and peptidoglycan synthetic pathways compete for a common pool of the universal lipid carrier undecaprenyl pyrophosphate (Und-P) whose flux alterations are closely related to the exopolysaccharide composition (Tatar et al., 2007). Transcriptomic analyses revealed that HMV strain B1230 exhibited the downregulation of genes associated with LPS biosynthesis compared to the NMV strain B1230-C (**Figure 5A**). The majority of the downregulated LPS biosynthesis genes (such as the *waa*, *wec*, *kds*, and *lpx* operons) were responsible for producing lipid A and core oligosaccharide, which aligned with a recent study (Hu et al., 2024). We further quantified the bacteria LPS lipid A (endotoxin) using the Limulus Amebocyte Lysate (LAL) test. The HMV strain B1230 showed a significant reduction in LPS lipid A levels compared to the NMV strain IR5061, a distinction further validated in their isogenic strains (**Figure 5B**). Structural analysis of LPS revealed that both strains, as well as their isogenic counterparts, were O-antigen deficient (**Figure 5C**). Notably, we found a marked reduction in lipid A and core oligosaccharides in the HMV strains B1230 and IR5061-M compared to the NMV strain IR5061 (**Figure 5C**). This reduction was reversed in the complementary strain B1230-C (**Figure 5C**), indicating diminished lipid A and core oligosaccharide production in HMV strains. KEGG pathway enrichment analysis revealed that the peptidoglycan biosynthesis pathway was significantly upregulated (**Figure 5D**). These findings suggest a significant compositional difference in polysaccharides between Wzc-mediated HMV and NMV strains in ST11-KL47 CRKp. We speculated that the excess production of KL47-type cell-free EPS may overconsume the raw material and divert it toward peptidoglycan synthesis, thereby reducing LPS synthesis, and ultimately contributing to reduced virulence.

Additionally, Granton et al. recently demonstrated that EPS conceals LPS from Toll-like receptor 4 (TLR4) recognition during *P. aeruginosa* pneumonia (Granton et al., 2024). To investigate whether a similar mechanism applies to *K. pneumoniae*, we used FESEM to visualize the surface capsule architecture. FESEM revealed notable differences between the capsules of the HMV strain and the NMV strain. HMV strain B1230 displayed a thick, filamentous capsule that tightly enveloped the bacterial cell, with its outer membrane enshrouded in a dense network of filaments. In contrast, NMV strain IR5061 exhibited a looser capsule composed of short, fine, and diffuse fibrils, which left the outer membrane more exposed. These states could be reversed by Wzc mutation (**Figure 5E**). We applied FESEM on four additional strain pairs, and the K1, K2 serotype representative hvKp strain NTUH-K2044 and ATCC43816. NMV strains exposed the outer membrane, while the extracellular matrix of the HMV strain showed varying degrees of encapsulation. The milder the virulence, the tighter the extracellular matrix encapsulation (**Supplementary Figure S4**). These observations suggest that the dense extracellular matrix of the HMV ST11-KL47 strain’s capsule may conceal lipid A of LPS and other surface antigens on the outer membrane.

## 4 Discussion

Morphological plasticity is a key survival strategy utilized by pathogenic bacteria to adapt to hosts and various environments. The high genetic heterogeneity in the CPS biosynthesis locus confers *K. pneumoniae* to express diverse mucoid traits via complex regulatory pathways. Yet, how changes in mucoviscosity affect the adaptive evolution and virulence of CRKp during host infection remained to be elucidated. Here, we proffered a perspective that the virulence of the ST11-KL47 CRKp strain could be attenuated by acquiring the HMV phenotype through mutations in the *wzc* gene. This provides novel insights into the regulatory relationship between CPS biosynthesis, the HMV phenotype, and virulence.

Wzc is a conserved protein involved in CPS assembly in various pathogenic bacteria (Yang Y. et al., 2021). Understanding its role in HMV phenotype development and bacterial virulence is essential. Our analysis revealed that mutations in Wzc occurred more frequently in KL47-type *K. pneumoniae* compared to KL64 strains (He et al., 2023). *Hu* et, al found that KL47 accounted for the majority of the isolates from northern and northeastern China (Hu et al., 2024). Hence, Wzc mutations might be prevalent in certain geographic areas, largely contingent upon the distribution pattern of KL47. Moreover, ST11-KL47 CRKp strains harboring virulent plasmids or the *rmp locus* did not exhibit high sedimentation values, highlighting the influence of genetic context on gene expression and function (Li et al., 2021). This suggests that the regulatory mechanisms of mucoviscosity vary across different K-serotypes, with *wzc* mutations rather than the *rmp locus* being the primary drivers of the HMV phenotype in ST11-KL47 CRKp strains.

Our study confirmed that the A533V mutation in the Wzc Walker A motif essential for ATP/GTP binding and ATP-dependent dimerization (Leipe et al., 2002) increases mucoviscosity in ST11-KL47 CRKp. Moreover, HMV is a distinct phenotype primarily influenced by changes in CPS spatial distribution. Cell-free EPS is the prominent component responsible for mucoviscosity and increased sedimentation efficiency. Mutations in the function regions of Wzc may impact its hyper-phosphorylation, disrupting the reassembly of the Wzc octamer and/or its interaction with Wza or Wzi. Consequently, this disruption likely results in a pronounced release of non-polymerized EPS subunits into the surrounding environment and explains why Wzc variants regulate homogeneous CPS and EPS chain length independent of the *rmp locus* (Khadka et al., 2023; Ovchinnikova et al., 2023).

Intriguingly, our findings revealed a paradox between phenotype and virulence in Wzc mutated ST11-KL47 strains. Despite exhibiting the HMV phenotype, these strains showed relatively low virulence in animal models. Wzc-mediated HMV strains exhibited a competitive disadvantage in LB broth and decreased biofilm formation and serum resistance compared to NMV strains *in vitro.* The overproduction of cell-free EPS and maintenance of the HMV phenotype likely impose a significant metabolic burden. Strains with a higher metabolic burden exhibited diminished fitness or viability (Hathaway et al., 2012). The thick capsular layer of HMV strains may impede the efficient uptake of nutrients (such as oxygen or iron) and energy, leading to slower growth rates compared to NMV strains, which may further reduce its adaptability to environmental stresses (Mike et al., 2021). These results contrast with HMV strains from other K types carrying virulent plasmids and the *rmp locus* (He et al., 2023; Liao et al., 2024). This raised the consideration that although virulence plasmids and the *rmp* locus have limited influence on the formation of the HMV phenotype, they may be pivotal in mediating virulence (He et al., 2023). Previous studies have linked virulence to mannose content and structure of CPS. Strains possessing mannose-α-2/3 mannose structures exhibit reduced virulence, likely due to activation of the complement lectin pathway (Yu et al., 2007). These strains, which express mannose-α-2/3 mannose-containing repeating units, showed significantly reduced serum resistance when compared to strains without these repeats (Sahly et al., 2009). Moreover, KEGG analysis revealed up-regulation of fructose and mannose metabolism pathways in the HMV B1230 strain relative to its isogenic NMV strain B1230-C (**Figure 5D**). This suggests that Wzc mutations might alter the structure or composition of KL47-type cell-free EPS, leading to distinct biological activities and reduced capacity to endow high levels of virulence *in vivo*.

In addition to CPS, LPS, and cellulose were also extra-cytoplasmic glycan polymers that account for the sickness process during infection. LPS is a key virulence factor in gram-negative bacteria. It is composed of lipid A, core oligosaccharide, and O-antigen. Lipid A is a potent activator of the innate immune system, leading to a complex cascade of signaling pathways and inflammatory responses, such as activating Toll-like receptor 4 (TLR4) and inducing pyroptosis (Ellermann et al., 2015; Heine and Zamyatina, 2022). Cellulose is the major exopolysaccharide component of the biofilm matrix with important implications in protecting the bacterial population from exterior insults (Romling and Galperin, 2015). Und-P is a universal lipid carrier of glycan biosynthetic intermediates and serves as an essential energy and raw material required for the biosynthesis of extracellular polysaccharides (Tatar et al., 2007). Competition for raw material was a general phenomenon across the synthesis pathways of CPS, LPS, cellulose, and peptidoglycan (Touz and Mengin-Lecreulx, 2008). Our observations suggest a potential link between the polysaccharide composition of the cell wall and changes in virulence-related characteristics. Excessive cell-free EPS production may compete for resources with other polysaccharides, which reduces LPS and cellulose synthesis and affects bacteria viability and fitness. Notably, LPS O antigen-positive strain B6_A533V_, which harbors the same Wzc mutation as O-antigen deficient strain B1230, did not exhibit the same reduction in virulence, indicating that HMV-related virulence variability depends on multiple factors. We also speculate that the secreted cell-free EPS of HMV CRKp could physically block the LPS and other surface antigens from immune recognition, as observed in *P. aeruginosa* (Granton et al., 2024). The thick EPS layer physically blocks direct contact between phagocytes and bacterial surfaces, impairing bacterial engulfment and adhesion. Cell-free EPS acts as a physical barrier that masks cellulose and pathogen-associated molecular patterns (PAMPs) such as LPS. This shielding restricts bacterial adhesion to surfaces, a critical step in crucial for biofilm initiation. Moreover, it reduces PAMP exposure to immune cells, thereby weakening the host immune response and enabling bacterial persistence without triggering acute inflammation. Conversely, NMV strains were advantageous for expressing and exposing LPS and surface antigens, hence amplifying sickness. *Granton* et al. recently demonstrated that EPS^+^ *P. aeruginosa* infections in neutropenic hosts were lethal, whereas EPS^-^ were not (Granton et al., 2024). Neutropenia is a major risk factor for severe bacterial infection, particularly in immunocompromised individuals. Hence, the Wzc-mediated HMV strains might pose a greater risk in particular clinical environments, such as intensive care units (ICUs), and present significant challenges for infection control and therapeutic interventions. Overall, our findings suggest that defining hvKp cannot rely solely on HMV phenotype, sedimentation values, and genotype, as the clinical implications of those indicators across diverse strain backgrounds remain unclear. Clinical outcomes and multiple virulence assessments must be considered across diverse strain backgrounds and patients’ conditions (He et al., 2022). Definitive conclusions on the clinical risk associated with Wzc variants require correlations with clinical outcomes in the patient population and comprehensive epidemiological investigations, which future research endeavors should aim to address.

## 5 Conclusion

In summary, Wzc mutations are the current and future evolutionary trend in ST11-KL47 CRKp, leading to the acquisition of the HMV phenotype and virulence diversity. This study expands our understanding of the genetic and virulence characteristics associated with distinct CRKp morphotypes and offers novel insights into the widespread prevalence of HMV strains.

## Data Availability

The datasets presented in this study can be found in online repositories. The names of the repository/repositories and accession number(s) can be found in the article/**Supplementary Material**.
