# Supplementary material for: Adaptive attenuation of virulence mediated by Wzc mutation in ST11-KL47 Carbapenem-resistant Klebsiella pneumonia
Source: Front Cell Infect Microbiol. 2025 Mar 11;15:1561631. doi: 10.3389/fcimb.2025.1561631 (PMC11933079; doi:10.3389/fcimb.2025.1561631)
Supplement: Supplementary file 1 [file DataSheet1.pdf]

Adaptive attenuation of virulence mediated by Wzc mutation in ST11-KL47 Carbapenem-resistant *Klebsiella pneumonia*

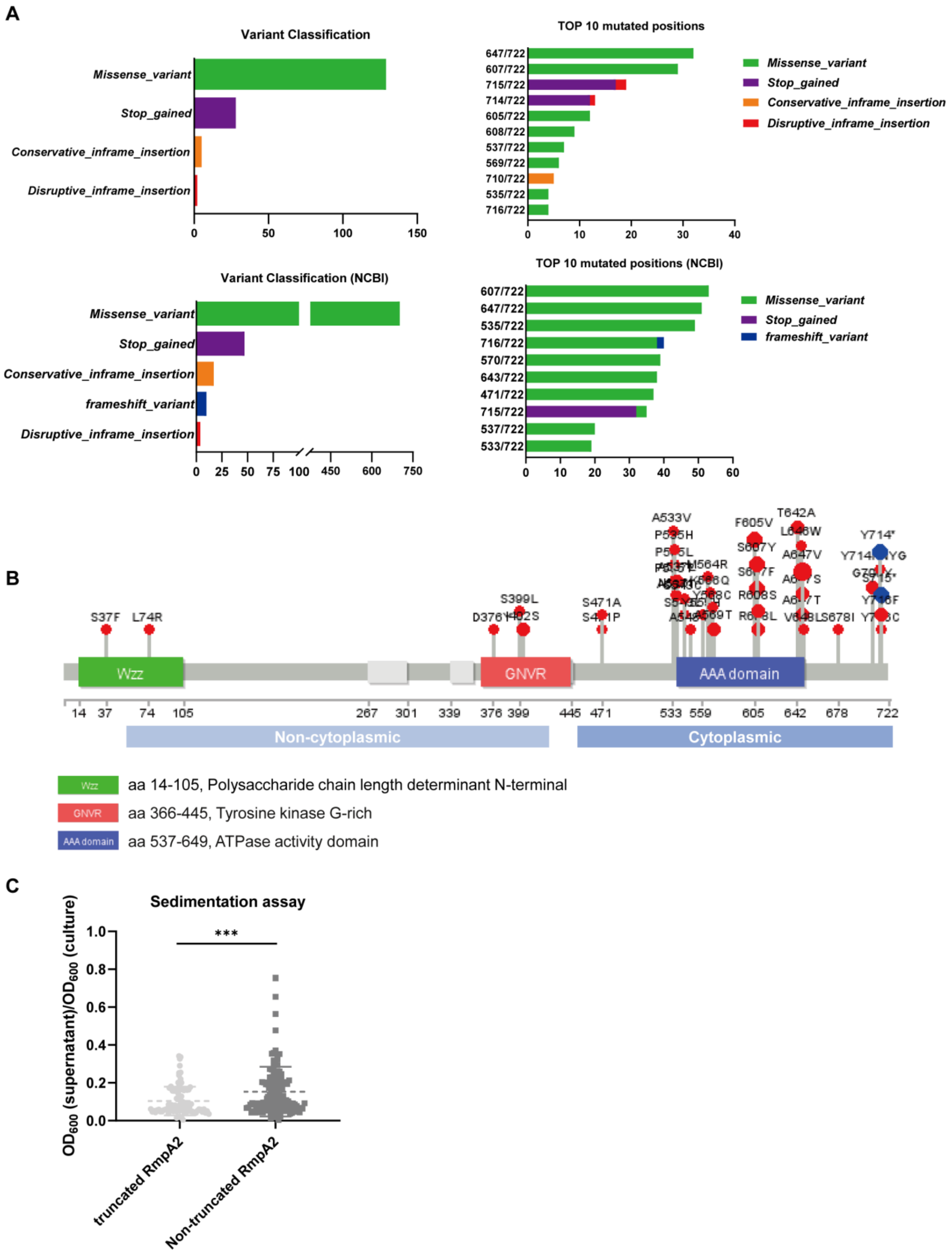

Figure S1 Variant classification and mutated positions of Wzc. A Variant classification and mutated positions of 230 clinical ST11-KL47 CRKp and 2517 *K. pneumoniae* genomes from NCBI. **B** Diagram of Wzc. The predicted functional domains (Wzz, GNVR, and AAA domain) from UniProt and cellular localization of each domain, active site, and oligomer interface predicted by InterPro. **C** Sedimentation values between RmpA2-truncated harboring group (n=90) and Non-RmpA2-truncated harboring group (n=140). Each point represents the mean value of three biological replicates of one strain. P = 0.004. ***P < 0.001; Unpaired two-sided Welch's t-test.

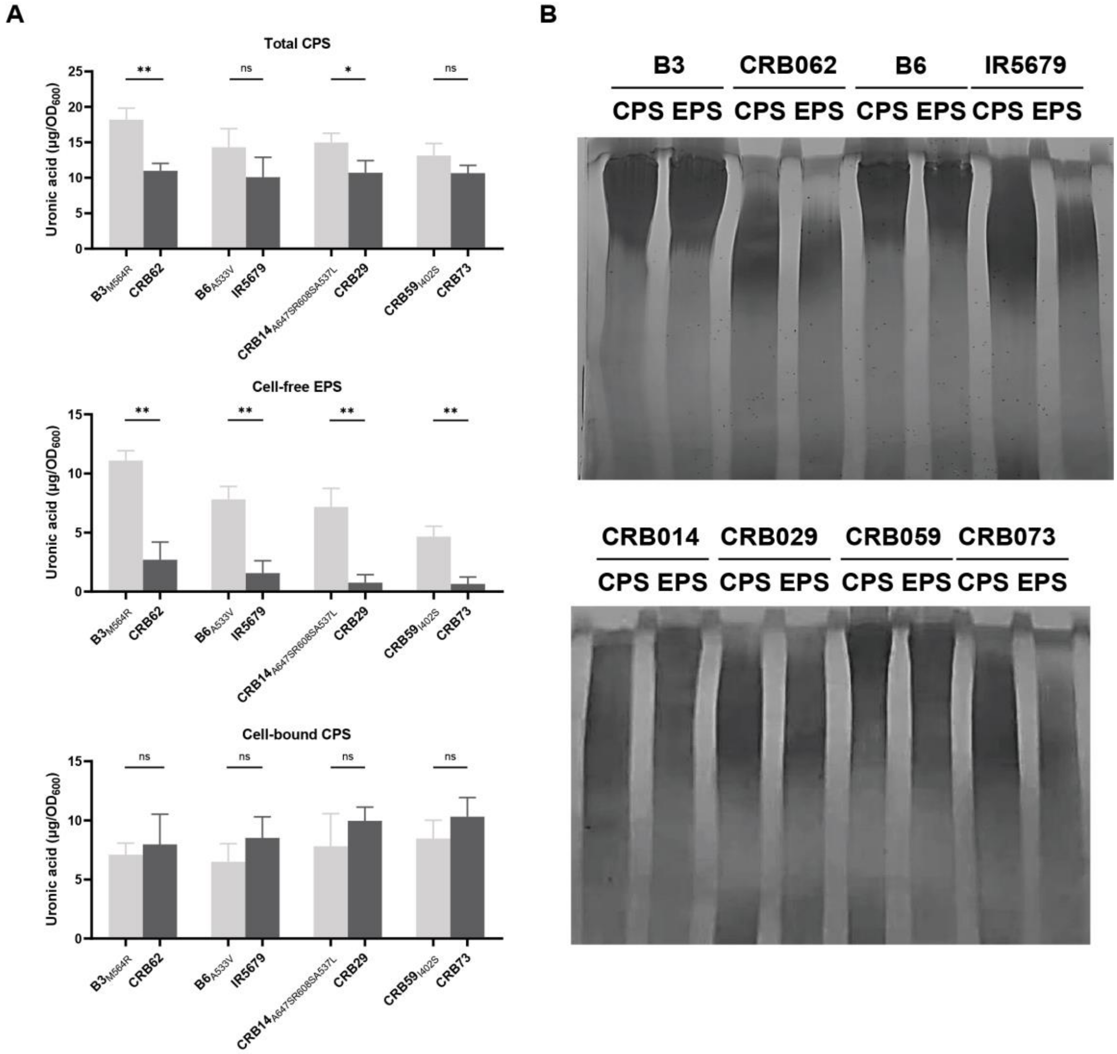

Figure S2 The capsule-traits of ST11-KL47 CRKp. A Uronic acid measure for capsule polysaccharide (CPS) quantification. n = 3 biological replicates, bars indicate mean ± SD. *P* values from left to right: 0.0031, 0.1286, 0.0254, 0.1027, 0.0010, 0.0020, 0.0029, 0.0026, 0.3383, 0.4094, 0.2187, 0.7118. ns, not significant; **P* < 0.05, ***P* < 0.01; two-tailed Student's *t*-test. **B** Silver staining of CPS and EPS resolved on SDS-PAGE. Images are representatives of three independent experiments.

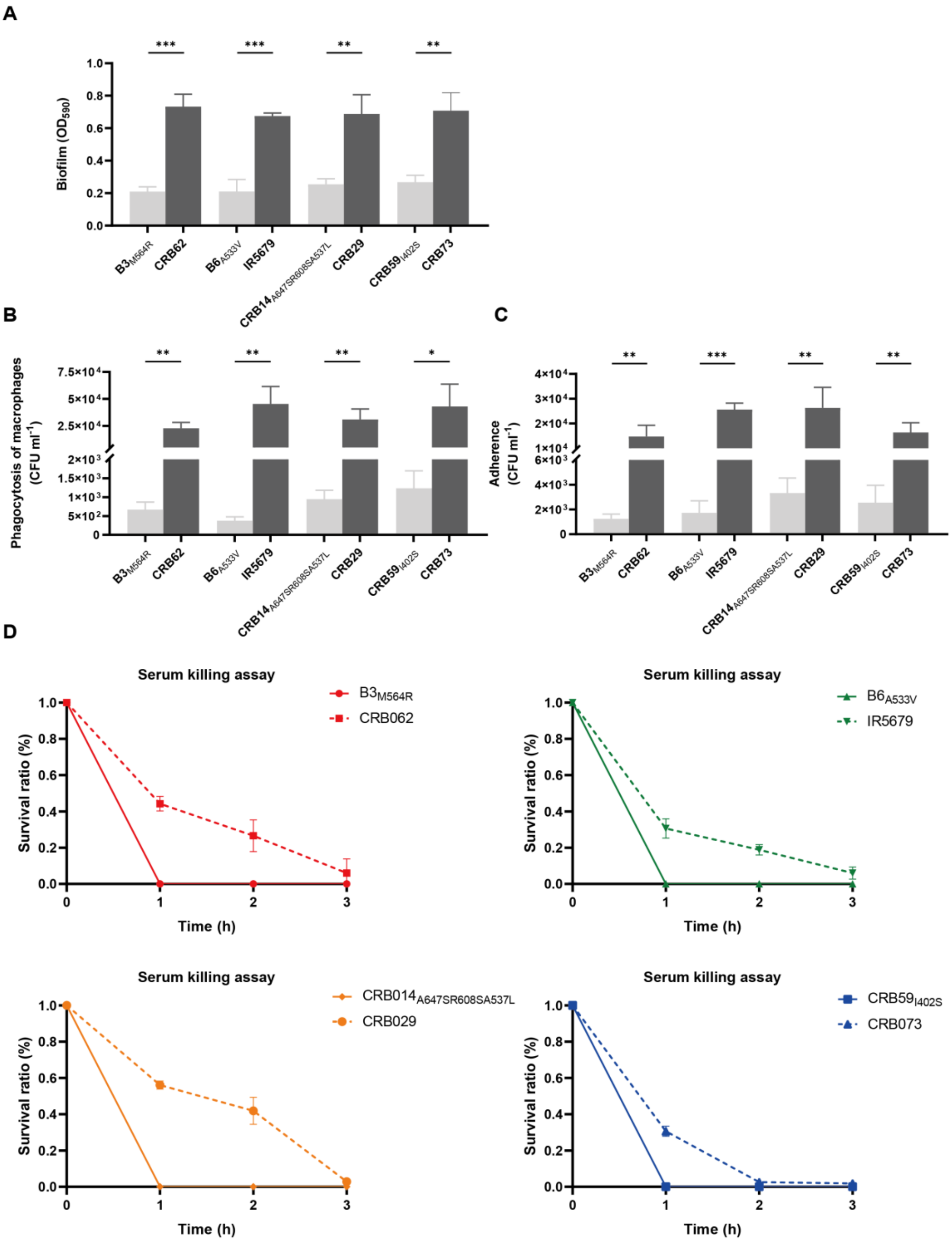
A
B
C
D
Biofilm (OD590)
Phagocytosis of macrophages (CFU ml-1)
Adherence (CFU ml-1)
B3M564R
CRB62
B6A533V
IR5679
CRB14A647SR608SA537L
CRB29
CRB59I402S
CRB73
Serum killing assay
Survival ratio (%)
Time (h)
CRB062
CRB014A647SR608SA537L
CRB029
CRB073

Figure S3. The fitness-traits of ST11-KL47 CRKp. A Biofilm formation. n = 3 biological replicates, bars indicate mean ± SD. *P* values from left to right: 0.0004, 0.0005, 0.0036, 0.0029. **P* < 0.05, ***P* < 0.01. ***P* < 0.01, ****P* < 0.001; two-tailed Student's *t*-test. **B** Phagocytosis of B1230, IR5061, and corresponding mutants by RAW 264.7 macrophages. n = 3 biological replicates, bars indicate mean ± SD. *P* values from left to right: 0.0021, 0.0085, 0.0057, 0.0249. **P* < 0.05, ***P* < 0.01; two-tailed Student's *t*-test. **C** Cell adhesion of B1230, IR5061, and corresponding mutants to A549 lung epithelial cells. n = 3 biological replicates, bars indicate mean ± SD. *P* values from left to right: 0.0069, 0.0001, 0.0090, 0.0045. ***P* < 0.01, ****P* < 0.001; two-tailed Student's *t*-test. **D** Survival in human serum.

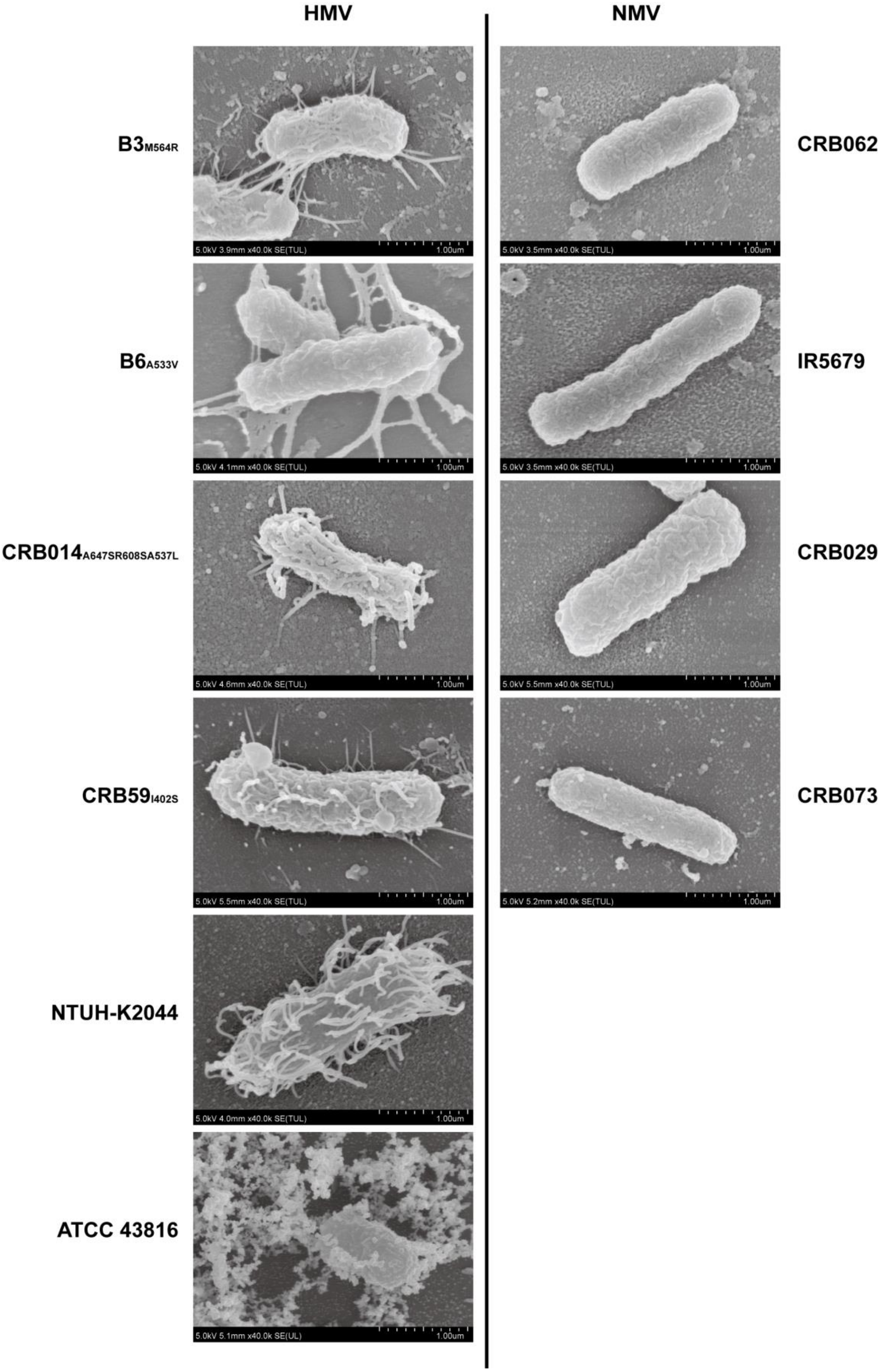

Figure S4 Representative SEM images of SEM of four pairs ST11-

KL47 CRKp, NTUH-K2044, and ATCC 43816. Images are representatives of three independent experiments.

Table S1. Primers used in this study.

Primers	Sequence (5'->3')
01	cgcGGATCCACGCTGAGCCAGGTACTAGT
04	acgcGTCGACCCGGATCAGTGACAGCCAAA
F0	TCGTGATGTCGAATCTGGGC
R0	CACCACGCCGTTTAAGATGC

Table S3. The genomic characterization of CRKp B1230 and IR5061.

<table>
<tr><th>Isolates</th><th>ST</th><th>K locus</th><th>L locus</th><th>Genome Size (bp)</th><th>Drug Resistance genes</th><th>Virulence genes</th></tr>
<tr><td>B1230</td><td>ST11</td><td>KL47</td><td>OL101</td><td>5872290</td><td rowspan="2">aac(3)-IId, aph(3'')-Ib, aph(6)-Id, bla$_{\text{CMY-2}}$, bla$_{\text{CTX-M-14}}$, bla$_{\text{CTX-M-55}}$, bla$_{\text{KPC-2}}$, bla$_{\text{SHV-182}}$, bla$_{\text{TEM-104}}$, bla$_{\text{TEM-105}}$, dfrA1, dfrA25, floR, fosA6, mdf(A), mph(A), qnrS1, sul1, sul2, tet(A), tet(D)</td><td rowspan="2">entABES, fepABCDG, fimAE, fyuA, irp1, irp2, iucABCD, iutA, mgtC, ompA, xcpR, ecpABCDER, ybtAEPQSTUX</td></tr>
<tr><td>IR5061</td><td>ST11</td><td>KL47</td><td>OL101</td><td>5882101</td></tr>
</table>

Table S4. Thirty-two nonsynonymous SNPs between B1230 and IR5061.

GENE	NT_POS	AA_POS	Description
hycE	1367/1710	456/569	Formate hydrogenlyase subunit 5
modA	78/774	26/257	Molybdate-binding protein ModA
glpA	268/1623	90/540	Anaerobic glycerol-3-phosphate dehydrogenase subunit A
ackA	701/1203	234/400	Acetate kinase
fadJ	734/2145	245/714	Fatty acid oxidation complex subunit alpha
yecM	409/411	137/136	Protein YecM
dhaD	866/1098	289/365	Glycerol dehydrogenase
prs	730/948	244/315	Ribose-phosphate pyrophosphokinase
mdtH_1	1027/1233	343/410	Multidrug resistance protein MdtH
maeA	547/1698	183/565	NAD-dependent malic enzyme
bcsA	941/2619	314/872	Cellulose synthase catalytic subunit [UDP-forming]
lacI_1	934/1071	312/356	Lactose operon repressor
mdoH	432/2529	144/842	Glucans biosynthesis glucosyltransferase H
cadC	357/1569	119/522	Transcriptional activator CadC
lapA	223/225	75/74	Lipopolysaccharide assembly protein A
ttuB_3	296/1311	99/436	Putative tartrate transporter
chbR	173/825	58/274	HTH-type transcriptional regulator ChbR
yphA	103/468	35/155	Inner membrane protein YphA
dld_1	1215/1746	405/581	Quinone-dependent D-lactate dehydrogenase
rlhA_2	982/1362	328/453	23S rRNA 5-hydroxycytidine synthase
xerC_4	7/903	3/300	Tyrosine recombinase XerC
ytbE	247/870	83/289	putative oxidoreductase YtbE
gntR	550/945	184/314	HTH-type transcriptional regulator
mukB	314/4449	105/1482	Chromosome partition protein MukB
nfi	166/672	56/223	Endonuclease V
wzc	1598/2169	533/722	Putative tyrosine-protein kinase in cps region
traC_5	221/2628	74/875	Protein TraC
