# Supplementary material for: Adaptive attenuation of virulence mediated by Wzc mutation in ST11-KL47 Carbapenem-resistant Klebsiella pneumonia
Source: Front Cell Infect Microbiol. 2025 Mar 11;15:1561631. doi: 10.3389/fcimb.2025.1561631 (PMC11933079; doi:10.3389/fcimb.2025.1561631)
Supplement: Supplementary file 3 [file Table2.docx]

>IR1230_00001

ATGCATGTTGTCATCAGCACTGTGGAAGAAAATATGAACAACAACAATCTGATTATCGTCGCAGAAGACGATGATGACATCGCTGCCATACTGACAGGCTATTTACGTAAGGCAGGTATGAAAACCTTAAGAGCCGAAGACGGTGAGCAGGCAATCAATCTTACCCGTCTGAACAAGCCTGATCTCCTGCTATTGGACATCCACCTGCCCGTGTACGATGGCTGGAATGTTCTCACCACGTTAAGAAAGGAAACGAATGTACCGGTCATCATGGTGACAGCACTGGATCAAGACGTTGATAAACTGATGGGGCTACGGCTCGGGGCTGACGACTATGTGATAAAACCCTTCAATCCATCTGAGGTTATTGCCCGGGTTGAAGCTGTGCTTCGCAGAACCAGACCGGTTGCAGAGTCTACACATTCACGGCCTTTAAGAACGCCTTTTTTAACGATTTATCCTGATGAGTTTTACGTTGAAATCACTGCACAAGGTGAGGTTTCCACGCCAGTATTAACCACCACTGAGTTTAAGCTTTTAACCTATTTGGCGAGAAACCCGAGAAAAGTCTGTTCACGTGAAGAGTTACTCGATGCGTGTTTACCTGAGGGAGACTCGCTGGATCGTACTGTAGACAGTCATATGAGTAAACTTCGGAAAAAGCTTGAACATGCCGGATTGAAGGGCATTCCGGAAAGCATCCGAGGATTAGGCTACCGGTTGGGTGACAAAAAATGA

>IR1230_00002

ATGAACAAAGAAACGATTCTGAGCCGTCAGATTTTGACATATATGTTGTTGTTAACGTTTGTTATTATTGCCATTGCAATACTAGGTTCCTACTTATTTTATAGTTTCCTGATTTATTACCTGCCGGGTGGAATGAACGAAGGTAGTGAAGATGCTATGACATTCCTTGACTGGATGTGGATTCTGATGGCGTCCATTACCAGTCTGGTCGTTGCACTTTTCTTTACCGTAAAGCTGTCTGCAAGAATATTCAACCCACTGAATGACGTTGCATACAGCCTGAAACAAATCTCTCAGGGAAACCTTAGTGCCAGAGCATTTAACAGCAGTTCTAAGTTGGGAGAGATGAATAGGCTCGTCAATGACTTTAATGAGATGGCTGAAAAGTTACAGACGCTGGATGCGCAGCGAAATTTATGGAATGCTGCAATTGCTCATGAACTCCGGACGCCGGTGACTATCTTACGTGGGAGGCTTCAGGGACTGGTTGACGGCGTTTTCGAACCAGAACCGTCGTTATTCAGAAACCTTCTTAAACAAACTGAAGGTCTGACCAACTTAATTGAAGATCTCCGCGTCGTGAGTTCTTCAGGAGGCGCAGGGTACTCCTTGATGCTGAGTGAAGTCGATCTCAAGGCCACGATTTCAAATGCGCTTGATACCTTTTGGCCGGACTTTGATAAAAAGCATTTCAAAATTGTCACTGAGATTAACCAGCAGTATTGCGTTTGCGATCCCTTACGCATTATTCAGTGCCTCACCGTTCTTTTTGACAATGCCTTAAAATACTCCACATCACAAACGCTGTTAATAAAGAATGGTATTACAGGAAACGATAATTTTATTATCGTTCAGGACCAAGGACCTGGAATTCCTGAAGAACTCCAGAAGTCCTTATTCCAGCCTTTTCAGCGAGGAGAATACGCCAAAAATATTAATCCAGAGGGCTGTGGTTTAGGGTTGTCGGTAGTTAAAGCCATTATGTGCGCCCACGGTGGAGACGTTAGCTATAGTTTAACACCAGAAAATGGCTCACTTTTCAAACTCTCATGGCCTGTTTAA

>IR1230_00003

ATGAACAAAATAAACTATCAAATCAAATACATTGAATATTTGTTAAGAAAATGCAGAACAATTTTGACTAATGACATTTCATTTCATGCAGACAGGTTAAGAGAAATATCTGGCACATATCCGGATTTACTCAACCCTGTAACGCTTAATGAGAAAATTTGCCATCGCATTTTATTTATCCACAACCCATTTTATACATTGCTTGCTGATAAACTTCTGGTCAGGCAGTATGTTGAGAAACGGACTAATTTAATTAAGCTTATTCCTTTAGTAGGTGTTTATAATAGAGTTGATGATATTGATTTCGACAAACTTCCCTCAAAATTTGTTTTGAAATGCAATCATGATAGTGGTAGCGCGGTCATATGCACGGATAAAACGAATATTGATCCCGCTAAGGTTAAAAGCAAATTAAAACTATCTCTTAAAAAGAACATGTATTATACGACCCGAGAATGGCAGTATAAAAACATACCACCCGTGATCCTATGCGAAATGTATCTGGATCTATTTTCCAGTAAACACCAGAATATAACTCCGGAAATGATAAGAATACATTGCTTTCACGGTGTGGCATGTTTTATCGAGGCCGACTTTACTGACAGCGATGGAAACGAATTCATTAACGTTTATGACAGAGCGTGGAATTTACAACCCTTTCAGATGGAATACCCAAACACGCCATTGCCGGTTGATGAACCAGAATCATTTCATAAATCCGTTATTGCCGCCCAGGACTTAGCAAAAGAAATAGACTACTGCCGCGTCGATCTTATGCTAAAAGGAGATGATATCTATTTCAGCGAAATAACATTAAGTCCCAAAAGAGGCAAACTTAAAATCACCCCATCAATTTGGGACGCAAAACTCGGCAGTATGTGGGATCTGTCTCTTGCTAAAACAGGTTCGATCGAGCCTGTGTATTCATGCCCTTAA

>IR1230_00004

ATGAGTGTCCTCTTTCCACCCAGGCTGGCTCCAGGAGATGTTATTGGTGTAACCGCTCCCTCTGCTGGCGTACCTGAACATCTGCATCCCAGACTGGAACTCGCCATAAAAAACCTAAAGAAAAGAGGTTACCAGGTCCGCGAAGGACGCTGCTTACGCTCGCAGCACAAAAATAAAAGCGCCACTAAATTTTCCCGCGTTGAAGAATTAATGTCTTATCTTACTGACCCAGATATAAAAGCTGTTATGCCACCTTGGGGCGGTGATCTGGCGATGGAATTGCTTGATTTGATTGATTTTGATCTGTTGTCCCGGTCTAAACCAAAATGGTTTGTGGGCTTTTCAGATCTGAGTACACTTCATTTCCCACTGACGACGATATCGGGCTGGGCTACTTTGCATGGGCCAAACCTCATGGATCTTGGCGCTCAAAAGTTAGATGCCACCACTCAGGCCGTATGGGAAATTCTGGAATCGAATCGGGGAACTGTAATCAAACAGTACTCTTCAACAGCATTTCAAGCGGATGAAAATCAATGGGGAACAGCGTCAGACGGAGGTTTCAATCTGACCCAAAAAACACAGTGGAAACGCCTTGATGGTGTTACATCTTCTCTGACATTTAGCGGGAAACTGATCGGCGGCTGCCTCGAAATAATATCCAGGTTAGCAGGTACACCATTCGGGAATGTGCCCTTGTTCAAGGCGAGCAATAGCCCTCAAGGAATAATCCTCTATTTTGAAAATGTGGAAATGGCTCCCTGTGAATTAACACGCGCATTATTTAGCTTACGATTACAGGGTTGGTTTGATAACTTAAATGGTGTTCTTATCGGCAGAAGTGCTGCACCCGACGTAAGCGACCCGACTAAACATAATTATCTGGATGCCCTTAAAGCCGCTTTTGAGAATATTGCCGTGCCCGTACTGTATGATGTTGATATTGGCCACATACCTCCTCAAATATCCCTGGTCAATGGAGCCGATGCGACGGTATTTTTCGCTGAAAATGGTAGCTGGGTAACTCAGCAGTTGTAG

>IR1230_00005

ATGCAAGCTATCAGACTATTCACCCTGATTCTGCCATTGTTCCTGGCACTCTGCGCCGGTATACCGGCCAGCGCCAGCACACCTTCAGGCAGCATTTCCTCTGCAAACACAGCAATTACCACTCATGATAATAACGGAAACCTCCTGCCGAATGCTCTGCGGATTCAGTATGATGTTGATTCAGATGGCAGAGTTCAGAATGTAAAGATACTGGAATCGACAACGACTCCGGAGTTTGAACATAAAATCATCGAGAAAATGATGAGTAAATGGCGCTTTGAAAAGGGGAAACCGGGAATAGCTAAAAGAGTAGTCGTCATGAGCCAACCCAAATCAACCGGCGGTCCCGCGAATAAGCAGTAA

>IR1230_00006

ATGAAAGAACTTTCACTTCTTGTTCCACTGGTGTTTACTGCGCCGGTACAGGCTTCTGAAGTCACGGTAGGTCAGATTTGCAAGGCGGCTTCTGCTGCGATGTTTGGCCGTGACCATAAGATCATGCAACTGGATAAAGTGGAATCAGGTATTGCCTACGTACATTATATCCGACAGAACGATGGCACCCGTTGGGCAATAAAGTGTAAACTTATAGGTGATCAGGTTATGTGGGCATCTGATAACCCTGACAGCACCGGTCGCTGGCGGGATGATCCTGCGGACAGTACAGTCAAATATTCTATTGATGGTAAAAAAATAATTATTACCGAGCTCTATACTGACGGTTCCAGTACCACGAACTCTTATCCCTTGATGCAGCTAAAATGA

>IR1230_00007

ATGAAGATCAGACTTGCTGTGCCTGTGGAGGCAGAAGAATGCTGGAACATTCGAAATCAGGCTATACGTTACGGATGTAAGAGTAGTTATGATGATGCGGTTATTGCAGCCTGGACACCGGAAAAAATGCCTGAGAGCTACAGGAACGCCATTGTAGTGAATCCTTTTTTCGTTGTTGCTGCCCCTGATGGCAGACTGTTAGCTACAGGCTATCTTGATCTATCTACAGGAAGTATTGAGGCTGTATTTACGCTACCTCAATATACAGGTAAAGGTTTAGGTAGTCAGATTATTGAAGCCATCAAAAGTGAAGCTCGTGGACGTGGTTTTGAGCAATTGACTTTGTCCTCAACACCTAATGCCCAGACTTTTTATGAGAAACATGGTTTCAAACTTATGCAGGAAAGCATGTATCCCTCTGCCTTGGCGCAGGCTGAACTGCGCTGCATGGAAATGTCTATAAATCTCCTGGGATAG

>IR1230_00008

ATGGAACGGCAACGACTGCCGTACGGTTCTCTCTCCCTCCGGAAGAAGGCCTGGGTGAGGGAAAGCACTGCCGGGAAGTCTTTTATGTTATCTGTGGCTTCAGCGTATTTTGCGCTCCCGGTTAATCTTCTGCGTTATGCCTGGCATGGCGCAGGGAGCGCACAGGGGGCGGCCAGTCGCCGCCGCCCCCTGTACCCCCGGGCTCCGGCCAGCAAAATCGCCGCTACGCGGTCCCTTCGACTTATCCCTGCAGGCTTCGGGTCGGGCCGAGGCAGCGTCCGTGCAAAACACGGCCCTCAGCCCGCATCCATGCGGGCTGCCCCGGCCTTCCGGGAACGTCTCAGCGATTTTGAGGCCGCAACACCGGCAGTTTCAGCAACAATGGTGAATAAATTAACGACAAGTCGCACGGACCGTAGGCCGGGTAAGTCGCAGCCGCCACCCGGCACAAAACCGGCTCCGCACCCGCTGCCCTTGCCCGGCGGCGCTACGCTTGCGCGGGCCTACGAAGGGCGCGAATTGAGCGCCAAATCACCGGGTCGGGTAAGGCGTAGCTGCCACCCGGCAAAAATACGGCACCAGAGCGTAGCCCGGATAAGGCGCCATACGCCGCCATCCGGGATTTTCTCTGCTGGCGCTGTCATGCCGCTGACGCGGCTTTCAGAAGCCTCTCGATGTTGTAAGTGTACGAATGCTATTCCAGAATTTCATGTCTGCTGTGTGCCAGGAGCGGACGTTGCCGCCTCACTCAAATTTGATGGGTATGCTTTAGAATTTTTATGGTAG

>IR1230_00009

ATGAGCCTGTTGGTGGTTATCGTCGCGCTGCTGCTGGCTGGCGCGCTGGGGCTGCTGTACTTCCCGTGGTCGGGCAAAGGGGCGGTAGACCGCGATGCCCTCAACCGCGCCCTGTACCAGTCGCGCCTGCAGGAGCTGGCGCAGGAGCGCGGCGAGGACAACCCGGCGCTGGTCGTCGAGCTGCAGCGCACGCTGCTGACGGATATTCCGCCCCAGGCACAGTCTGGCGAACGGCCGCTACGCCGCTGGGCGCTGCTTCCGGGCGCGCTGCTGCTGGTGGTCCTGAGTCTGGGCCTGTATCTGAAAACCAGCGATATTGGTCAGGTGCTGCTCTGGCAGCAGGCTGAACGACATTTTCCTGCGCTGTTGCAGCAGGTTAAGGACCCGACGGCGGCGCCGCTGCGAATGGATGAGCTGGCGGAGCTGCGGCTTGGCCTGCGCAGCCACCTGCAGGATACTCCGAACGACCTCGCCGGCTGGCAGCTGCTTGGGCGGCTGGGCCTGTTGTTAAACGATGGCGAGACGGCGATCGGCGCCTTTGGCCGGGCGCATGCTCTGGCGGCGGACGACCCGGCAGCGGCTTTCGATTACGCCAGCGCGCTGGTGCGGGCGGGAGATAGCGGTCAGGTTCGGATGGGCGAGCTGCTGCTGCGTGACCTGCATCAGCGGCAGCCGAACAGCCTGCCGGTGCTGGAGATGCTGGCCCTGAGCGCGGTGCGCAATGAAGATTACCCCGAGGCCGTGGCGGCCCTGCAGGCGTTGCTGGCCCGGCTACCGGAAGGGGATGCGCGGCGCGAGGCGATCGTCCGCCAGCTGGCGCAGGCGCAGCAGCAGGCGCAGTAA

>IR1230_00010

ATGAGAGGGGTAATGGTCATCCTGCTGGCGCTGATGCTGACCGGCCAGGCGTGGGCCGCCATCGATACCTGGCAGTTTAAGGATGAGGCCCAGGAGCAGGCCTTCCGCGAGATCACCTCCCAACTACGCTGCCCTAAATGCCAGAACAACAGCATCGCCGACTCAAACGCGATGATTGCCGCTGATATGCGGCAGAAGGTGTACGAGCTGATGCAGCAGGGTAAAACGAAAGGGCAGATCGTTGACTATATGGTGGCCCGCTACGGTCATTTTGTCAGCTATGAGCCGCCGCTGACCGCGGGCACGGTGCTGCTGTGGCTGGGACCGGGGTTGTTCGTGCTGGCGGGCGCGGGAGTGATTATCGCCCGCGCCCGCCGTCGCGACATTCCTGACGCGGCACTCACCGCCGAAGAGCGCCAGCGGCTGGCGGCATTATTGCAAGAAGGAAAAGAACGATGA

>IR1230_00011

ATGAACCGGAAGCTACTGTTTATCCCGCTGGTGCTGTTTCTGGCGCTGGCCGCCGCGCTGTTCTGGCAGCTGATGCGCAACGCCGATGGCGATGACCCGACGACCCTCGAGTCGGCGCTGATTGGCAAACCGCTGCCGGAATTTCGCCTTGAAGCGCTGAATACCCCCGGTCAGACGCTGGACCGACGGACGCTGATTGACGGCAAGCCGCTGCTGCTCAACGTCTGGGCCACCTGGTGTCCAACCTGTCGCGCCGAGCATCAGTTTCTCAACGGCCTGGCGCAGCAGGGCGTGCGGGTGGTGGGGATGAACTACAAAGACGATCGCCAGAAGGCGATGAGCTGGCTGCAGCGCCTGGGTAATCCCTACAGGCTGAGCCTCTACGACGGCAACGGCATGCTTGGTCTGGATCTCGGGGTCTATGGCGCGCCGGAGACGTTTCTCATCGACGGTCAGGGCATTATCCGCTGGCGGCACGCCGGCGACCTGAACGAACGGGTGTGGCGCGAGGAGCTCCAGCCGCTGTGGGATCAATACAACCGGAGGGCGGGTTGA

>IR1230_00012

ATGATGCCGGAACTGGGCAACTTTTTATTGTGTCTGGCGGCGGGGCTGGCGCTGCTCCTGAGCGTTTATCCCCTGTGGGGCGCGGCGCGGCAGGATCGGCGGCTGATGGCCCTGGCCCGGCCGCTGGCCTGTGGGCTATTCGCCTGTATCGGCGGCGCCTTCCTGCTGCTGGTGCACGCCTTTGTGGTCAATGACTTTACCGTCCGCTACGTGGCGGAAAACTCCAACAGCGCGCTGCCGGTGTGGTATCGGGTGGCGGCAACCTGGGGGGCGCACGAGGGCTCACTGCTGCTGTGGGTTCTGCTGCTCAGCGTCTGGACCTTTGCCGTCGCCATCTTCAGCCGCCGGATGCCGCTCGACGCCGTCGCCCGGGTGCTGGCGGTGATGGGGATGATCGCCTTTGGTTTCCTGCTGTTTATCCTCTTCACCTCCAATCCGTTCAGCCGCGGCCTGCCGCAGTACCCCATTGATGGTCGCGACCTCAATCCGCTACTGCAGGATATCGGCATGATTTTCCATCCGCCGATCCTCTATATGGGTTACGTCGGCTTCTCGGTGGCCTTTGCCTTCGCCATCGCCTCGCTGCTGGCGGGCCGGCTGGATACTGCCTGGGCGCGCTGGTCGCGTCCCTGGACCCAGGCGGCATGGATGTTCCTCACTCTCGGCATCGCGCTGGGCTCGGCGTGGGCATACTACGAACTGGGCTGGGGCGGCTGGTGGTTCTGGGACCCGGTGGAAAACGCCTCGTTTATGCCGTGGCTGGTGGGAACCGCGCTGCTGCACTCCCTGGCGGTGACCGAAAAGCGGGGCAGCTTCCGCGCCTGGACGGTGCTGCTGGCCATCGCCGCCTTCTCCCTGTGCCTGCTGGGCACCTTCCTGGTGCGCTCCGGGGTGCTGGTTTCGGTGCATGCCTTCGCCTCGGATCCGTCGCGCGGGCTGTTTATTCTGGTACTGCTGATTGTCGCCATCGGCGGATCGCTGCTGCTGTACGCCCTGAAGGGTGGCCGGGTGCGGGCGCGGGTGGAACATACGCTGTGGTCGCGCGAGTCGTTCCTGCTTGGCAACAATATCTTGCTGATGGCCGCGATGCTGGTGGTGCTCCTCGGAACGCTGCTGCCGCTGGTGCATAAAGAGCTGGGCCTCGGCAGCATCTCCATCGGCGAGCCGTTCTTCAATACCATGTTCACCGCCCTGATGGCGCCGTTTGCGCTGCTGCTGGGGCTGGGACCGCTGATCCGCTGGCGGCGCGATGACGTCGCCAGGCAGATTAAGCGCCTGATTATCGCTCTGCTGGTGACGTTGTCACTTTCTCTGGCGCTGCCGTGGCTGCTGCAGGACCGTATCACCGCCATGGCGGTCATAGGCCTGATGATGGCCCTGTGGGTGCTGATCTTCGCCCTGCTGGAGGTTCACGAACGGGCCACCCACCGCCACGGTTTCTGGCGCGGCCTGCGCGCCCTGACCCGCAGCCAGTGGGGGATGGTGCTCGGTCACGTCGGCGTGGCGGTGACCGTTATCGGCATCACTTTCAGCCAGAACTACAGCGTGGAGCGCGATGTACGGATGCGTCCCGGCGACAGCATCGATATCCACCGCTATCACTTTGTGTTTAACGGCGTGCGCAATATCGTCGGCCCCAACTGGACCGGCGGCGAAGGCATTATCGCCGTGACCCGCAACGGCCGCCCGGAAGCGACGCTGTATGCTGAGAAGCGCTTTTACACCGCCAGCCGGATGATGATGACCGAGGCGGCGATCAGCGGCGGGCTGACCCGCGATCTGTACGCCGCGCTGGGGGAAGAGCTGAGCGACGGCAGCTGGGCGGTGCGTCTGTACTATAAACCGTTCGTTCGCTGGATCTGGTACGGCGGGGTGCTGATGGCCCTCGGCGGTCTGTGCTGCATGCTCGACCCGCGCTACCGGATGCGTAAGAAGCTGCAGGAGGCCTCATGA

>IR1230_00013

ATGCAAGCGCGTCGTAAAACCCGGCTATATATCGTGCTGGCGGTGCTCGCCGGCCTCGGGCTAACCGTCAGCCTGACGCTGTACGCCCTGAGTAGCAATATCGATCTGTTCTACACCCCGGGGGAAATTATCTACGGCAAAACGGAAACCCGGGCGCTGCCCCATACCGGGCAGCACCTGCGGGTGGGAGGCTACGTTCAGCCGGGCTCCCTGCAGCGCGACCCGCAAACCCTCGACGTGCGCTTTAAGCTGTATGACGCCCGCGGGGTGGTGGACGTCAGCTATAAGGGCATTTTGCCGGACCTGTTTCGTGAAGGGCAGGGCGTGGTGGCCCAGGGCGTACTGGACGGCGAGCGGCATATTACCGCCCAGCAGGTGCTGGCCAAACATGATGAAAATTACACGCCGCCGGAGGTGAAAAACGCCATGACGCCGGAGAAAACGGGGGCGCAGCCATGA

>IR1230_00014

ATGAGCCCCGCCTTTTCGTCTTTCGCCGCCTTCCTGAATATGGGCGGCTATGCCGTGTACGTCTGGCTGGCGGTGGCCGTGGCCGTCGCCGCCTTTGGCCTGCTGACGGTGCATACCCTGTGGGCGCGGCGGGCGCTGTTCCATGAGGTGCGACGCCAGCAGGCGCGCGAGCGGCGGATCGCTGCCGCCCGCGAACATGACAAGGAGGCCGCGGATGCAAGCGCGTCGTAA

>IR1230_00015

ATGTGGAAAGCGTTACATCAACTGGCGATCCCCGAGCGGCTGTACCGCCTGTGCGGATGCTGGATCCCCTGGCTGGCGGCGTTGAGCGCGCTGTTGCTGGTCATCGGCCTGGGGTGGGGCTTTGGCTTCGCCCCCGCGGACTATCAGCAGGGGGAAAGCTACCGGATCATGTACCTTCACGTCCCGGCGGCGACGTGGTCGATGGGGCTGTACCTGGCGATGGCCGTCGCGGCCTTCGTCGGGGTGGTCTGGCAGATCAAAATGGCCGATCTGGCGATCGCCGCCCTGGCGCCGGTGGGGGCGGTCTGCACCCTGGTGGCGCTGGTCAGCGGCGCGGCATGGGGCAAGCCGATGTGGGGGACCTGGTGGATCTGGGACGCCCGCCTGACCTCAGAGCTGGTGCTACTGTTTCTGTATGCCGGGGTCATCGCCCTGTGGCACGCCTTTGACGACCGGCGGCTGGCCGGGCGCGCGGCGGGGATCCTCGTGCTGGTCGGGGTGGTCAATCTGCCCATCATTCATTACTCGGTCTACTGGTGGAACACCCTGCACCAGGGCTCGACCAACCTGCAGCAAACCATCGACCCAAGCATGCGTCTGCCGCTGCGGATCTGCATCTTTGCCTTCCTCACGCTGTCGGTCACCCTGACCCTGATGCGCCTGCGTAACCTGATCCTGCAACTGGAGCGCCGTCGACCGTGGGTGGTGGCGCTGGTGAATAAAGGAGCCGCCCGATGA

>IR1230_00016

ATGATGTGTGCGCTGTTGGCCCGGGAACTGCGCCTGGCGTGGCGCAGCGGGGCGGAGATCCTCAATCCGCTCTGGTTCTTTCTGATCGTCATCACCCTGTTTCCGTTCGGCGTCGGCGCCGCGCCGCAGCTGCTGGCGCAGATTGCGCCGGGCGTGGTGTGGGTCGCCGCGCTGCTGGCGGCGCTGCTGGTGATGGATCGGTTGTTTCGCGACGACTGGCAGGACGGTTCCCTGGAGCAACTGATGCTCCTGCCGACGCCGCTGGTGGCGGTGGTGCTGGTGAAGGTGGTTGCCCACTGGATGATGAGCGGCCTGCCGCTGCTGATCGTCTCGCCGCTGGCGGCGCTGCTGCTGGGGATGAGTCTGCATGACGCCGGTGTGCTGGCGCTGACCTTGCTGCTCGGCACCCCGACGCTCAGTTTTCTCGGCGCGGTGGGGGTTGGCCTGACGGTCGGCCTGAAGCGCGGCGGCGTCCTGCTGAGCCTGCTGGTGCTGCCGCTGGCGGTGCCGCTGCTGATTTTCGCCACCGCGGCCTGCCAGGCGGCGGCCGCCGGGCTCCCGGTCAGCGGCTATCTGGCGATGCTGGCGGCGTTCTTAACCGCCAGCGCCACCCTGTGTCCGTTCGCCACCGCCGCTGCGCTGCGGCTGACCGTGCGATAA

>IR1230_00017

ATGCTGCATGCAGAGCGACTGACCTGCATCGTTGACGATCGACCGCTGTTTGCGGCGCTGACGCTGTCCCTCGCTGCCGGCGAGCTGCTGCAGGTCGCCGGTGATAACGGCGCGGGGAAAACCTCGCTGCTGCGGATCCTCTGCGGCCTGGCGCGCCCGGAAAGCGGGGTGGTGAGCTGGCAGGGCCTGCCGCTGGCGAAGGTCCGGGAGTCATTTCACCGTCAGCTGCTGTGGCTGGGCCATAAACCCGGCGTTAACGCGGCGCTGACCGCCGATGAAAACCTGCGTTTCTTCTTTCCGGCCAGCCGCCTTCAGCAGCGGGAAAGCGCCCTCGCCGCCGTCGGCCTGGCGGGCTATGAAGATCTCCCCTTGAGCCAGCTCTCCGCCGGGCAGCAGCGTCGGGTGGCGCTCACCCGCCTGTGGTTAACCGACGCGCCGCTGTGGATCCTCGATGAGCCGTTCACCGCCCTCGACGCCACGGCGATGGAAACCCTGACCCGTCGCCTGGAGCAGCACGCCCGGCAGGGGGGATGCGCCATCCTCACCACCCACCAGCCGCTGCGGCCGCTGGGCTGTCCGTTGCGCACCCTGCGCCTCGGCGGCGACGCTGGAGGCGGGCAATGA

>IR1230_00018

ATGAAAATGCAATGGTTATCGGCCCTGGTGCTTGGGGCATTGAGCTGCGCGGCTTTTGCCGAAGAGGCGCCTGCGGACAGCAATCTGATTAAGCAGGGGGAGTATCTGGCGCGGGCGGGGGACTGTGTCGCCTGCCACACCAACGGCAAAGCGGGGAAACCTTTCGCCGGCGGTTTGCCGATGGAGACGCCGATCGGCACCATCTACTCCACCAATATCACGCCGGATAAAGAACACGGCATTGGCGGGTACACCTTCGAAGAGTTCGACGACGCGGTGCGCAAGGGCGTGCGGAAAGACGGTTCCACGCTCTATCCGGCGATGCCGTATCCCTCGTTCGCGCGGATCAGCGAAGCGGACATGCGCGCCATGTACGCCTACTTTATGCACGGCGTGGAGCCGGTGAATGTCGCCAACAAGGACACCGACATCCCATGGCCGCTGTCGATGCGCTGGCCGCTGGCGTTCTGGCGCGGCATCTTCGCCCCGACTCCGAGCGACTTTGTCGCCAACCCGCAGGTTGACCCGGTGCTGGAGCGCGGCCGCTATCTGGTGGAAGGCCTGGGCCACTGCGGCGCCTGTCATACCCCGCGTAGCCTGACGATGCAGGAAAAAGCGCTCAGCGAAAGCGAAGGCGATGATTACCTGGCGGGCAGCAATGCGCCGATTGACGGCTGGGTCGCCTCCAGCCTGCGCGGCGAAAACCGCGACGGTCTGGGGACCTGGAGCGAAGCCGAGCTGGCCGAGTTCCTCAAAACCGGACGCAACGATAAATCGGTGGTCTTCGGCGGCATGAGCGATGTGGTGGAGCATAGTCTGCAGTATCTCTCTGATGATGACATCACCGCCATCGCTCGCTACCTGAAGTCGCTCCCGCCGCGCGGCGGCAAACAGACCCCAGCCCCGGTGGAAGACAGCGTGGCGAAAGATCTGTGGAAGGGTAACGACAGCAAAACCGGCGCCGCGCTGTACGTCGATAACTGCGCCGCCTGCCACCGTACCGACGGCGCGGGCTATAAACGCGCCTTCCCGTCGCTGAAGGGCAACCCGGTGGTACAGACGGAAGATGCCACTTCGCTTATCCACATCGTTCTGACCGGGAGCACCACGCCGGCGGTGAAAGATGCGGTCTCCAACCTGACCATGCCGTCGTTCGGCTGGCGCCTGGACGACCAGCAGGTGGCCGATGTGGTCAATTTCATCCGCACCAGCTGGGGCAACAACGCGCCGGCGGTCAGCGCCAGCGATGTGGCGAAGGTGCGTAAGGAGACCGCGGCGCACGATGAGAAGGCGTTAGGCAACGCCGATATCTCGAAGCTGCCGGGGGCCGGACAGTAA

>IR1230_00019

ATGGCCACCGTATTGAAAAAAACCGATGTCGCGATCGTCGGCTTCGGCTGGGTTGGGGCGATCATGGCCAAAGAGCTGACCGAAGCGGGCCTCAACGTCGTCGCGCTGGAGCGCGGCCCGATGCGCGACACCTGGCCGGACGGCGCCTATCCGCAGGTGATTGATGAGCTGACCTACAACATCCGCCGCAAGCTGTTCCAGGATCTATCGAAAAGCACCGTCACCATCCGGCATAACACCAGCCAACAGGCGGTGCCCTATCGCCAGCTGGCGGCCTTCCTGCCGGGTACCGGCGTGGGCGGCGCCGGGCTGCACTGGTCCGGCGTCCATTTCCGCGTCGATCCCATTGAGCTGCGGATGCGCAGCCACTATGAAGAACGCTACGGCAAAAACTTCATCCCCCAGGATATGATCATCCAGGATTTCGGCGTCACCTACGACGAGCTGGAACCGTTCTTCGATAAAGCGGAAAAAGTATTCGGTACCTCCGGGACCGCCTGGTCGATCAAAGGCAAGGTCGTCGGCAAAGGCCGCGGCGGCAACGCCTTCGCCCCGGACCGCTCGGACGACTTCCCGCTGCCGGCGCAGAAAAACACCTGGTCGGCGCAGCTGTTTGAAAAAGCGGCGCTGGAAGTGGGGTATCACCCCTATAACCTGCCGTCGGCCAACACCTCCGACTCCTATACCAACCCCTACGGCGCGCAGATGGGCCCGTGCAACTTCTGCGGTTTCTGCAGCGGCTACGCCTGCTACATGTACTCTAAAGCCTCGCCGAACGTGAACATTCTGCCGGCGCTGCGCCAGGAAAAACGCTTTGAGCTGCGGACCAACGCCAACGTGCTGAAGGTCAACCTGACCGACGACAAATCCCGCGCCACCGGCGTGACCTACGTCGACGGCCAGGGGCGCGAAATGGAACAGCCGGCGGACCTGGTGATTATCGGCGCCTTCCAGTTCCACAACGTGCACCTGATGCTGCTCTCCGGGATCGGCAAACCGTACAATCCGGAGACCGGCGAAGGGGTGGTGGGGCGTAACTTCGCCTACCAGAACATGACCACCATCAAGGCCATTTTCGACAAAGACACCTATACCAATCCGTTTATCGGCGCGGGCGGCAACGGCGTCGGCGTCGACGACTTCAACGCCGACAACTTCGACCACGGCGCGGCGGGCTTTGTCGGCGGTTCGCCGTTCTGGGTCAACCAGGCCGGGACCAAGCCCATCTCCGGTTTCCCGGTACCGCCGGGCACCCCGGCGTGGGGCAGCAAGTGGAAAGCGGCGGTGGCCGACACCTACACCCATCACCTGTCGATGGATGCCCACGGCGCGCACCAGTCCTATCGGCAGAACTACCTCGATCTTGATCCGAACTACAAAAACGTCTTCGGCCAGCCGCTGCTGCGCATGACCTTCGACTGGCAGGAAAACGACATCAAGATGGCGCAGTTTATGTTCGATAAGATGGCGCCGATCGCCAAAGCGATGAAGCCGAAATATATCCTCGGCAGCCCGAAAAACGCCAACAGCCACTTTGATACCACCACCTACCAGACCACCCATATGAACGGTGGGGCGGTGATGGGGGAAGATCCGAAAACCAGCGCCGTGAACCGTTATCTGCAAAGCTGGGACGTGCATAACGTCTTCGTCATCGGCGCCTCCGCGTTCCCGCAGGGGCTGGGCTACAACCCAACCGGCACGGTGGCCGCGCTGGCCTACTGGTCAGCGAAGGCGATCCGTGAGCAGTATCTGAAAAATCCGGGTCCCCTGGTGCAGGCATAA

>IR1230_00020

ATGATGTCGAGCGAGAAAACCAACAATTCCAGGCGTGATTTCCTGGTGAAATCGATGGCGCTGATCCCGACGGTGGTGATCGGCGGCGCGGGAGCAGGGGCTATTGGCGTGGCCACCAGCGCGACCGCGCAGGCGGCCCCCGCTTCAGAGCCAGCCTCCGGGAACACCGCGGCGGCCAGCGACTGGAAGCCGCAGTTCTTCAACGATCGTGAGTGGGCGTTTATCAACGCCGCCGTCGCTCGCTTAATCCCGGCGGATGAACTCGGTCCCGGTGCCAAAGAGGCCGGCGTCCCGGAGTTTATCGACCGCCAGCTCAATACCCCTTACGCCACCGGCTCCATCTGGTATATGCAGGGGCCCTTCAATCCCGACGTGCCGAAAGAGATGGGTTATCAGCTGCCGTTGGTGCCCAAACAGATCTATAACCTCGGGATCGCCGACGCCGAGGCGTGGTGTCAGGACAAATATCATAAAACCTTCGCTGAACTGAGCAGCGAGCAGCAGGACGAGGCGCTCGGCCTGTGGGAATCGGGTAAAGCCGAGTTCAAACAGCTGCCGGCCTCGCTGTTCTTCACCTATCTGCTGCAAAACACCCGCGAAGGGTTCTTCAGCGACCCGATCCATGGCGGCAATAAAGGCATGGTCGGCTGGACGCTGATTAATTTTCCCGGCGCGCGCGCCGACTTTATGGACTGGGTTGAACGGGGCGAACGCTACCCCTTCCCGCCGGTATCAATTAATGGGGAGAGGGCGTAA

>IR1230_00021

ATGTTCGATCATGTGAAATTCGGCGTTAGCGACTATGCCGCCAGCAAAGCGTTCTTTTTGCAGGCCCTGGCCCCGCTTGGCGTAACGCTGGTCGGCGAAGGCGAACCGAGCTACGGCGCAGAGCTGGCCGGCAGCGGCGATGCTTCTCTATGCCTCTATCAAAGTGCGGAAAAACCGGCGCCGCTGCACATTGCCTTTCGCGCCGACAGCCGCGAGCAGGTCGATGCCTTCTGGCAGGCGGCGCTGGCCGCCGGAGGAAAGGACAACGGCGCGCCGGGGCTACGGCCAAACTACCACGCCAGTTACTACGCCGCGTTTGTGATTGCGCCAGACGGACACAATATTGAAGCCGTGTGCCATCTGGCGGGGTAA

>IR1230_00022

ATGATTGAGTTATCGGTAGAAAATCTGCATCTCACCTATGGCGATAACCCGGTGTTGAAGGGGGTGTCCATGACGCTCGGCCGGGGGGAGGTAGTATCGCTGCTGGGGCCTTCGGGAAGCGGCAAAACCACGCTGCTGCGGGCGGTGGCCGGGCTGGAAAAACCGACGAGCGGACGGATCGCCATCGGCAACCGCACGGTGTACGACGGCACGCCGCGCAGCGAGATCCCGGCCGAAGAGCGTAACCTGGGCCTGGTCTTTCAGTCCTATGCCCTGTGGCCGCACAAAACGGTGTTCGACAATGTGGCCTATCCGTTGAAGCTGCGTAAAGTCGCCGCCGGGGAGATAAAAGAGCGGGTGCAGCGTGTGCTGGATCAGCTGGGGCTGGGGCACCTTGGCAACCGCCATCCGCACCAGCTCTCCGGTGGCCAGCAGCAGCGGGTGGCCATCGGCCGGGCGCTGGTTTACAACCCGCCGGTGATCCTGCTGGATGAACCGCTCTCCAACCTTGACGCCAAGCTGCGCGAAGAGGCGCGGGTGTTTCTGCGCGAGCTGATCATTAAGCTGGGCCTGTCGGCGCTGATGGTCACCCACGATCAGAATGAAGCAATGGCCATCTCCGATCGTATTTTGCTGCTGAACAATGGCGTCATTGAGCAACAGGGGACCCCGCAGGAGATGTACGGCAGCCCGGCGACGCTGTTCGCCGCTGAGTTTATGGGCAGCAATAACCGCCTGCACGGCAAAGTGATGGCGCTGGAGAACGGCAGGGCGCGGATCGAAGGCGCCAGCTGGAGCCTGTGGGGGCGAGCGGGCGAGGGGGTTAGCGTTGGCGAGCCGGCGACGGCGGTGATCCGCGTCGAGCGCCTGCAACTTGACGGCGCCGCGCAGGATAATAGCCTCCAGCTACCGCTGCTGACCAGCATGTACCTCGGCGACCGTTGGGAGTACCTGTTCCGTACCGAAGGCGACGACTTTCCGCTGCGCGCCTACGGAACGGCGCTGCGCGATGCCGAACACTGCCATCTGACGCTCCCGGCGGAGGATGTGTGGATTTTTCCGCAGCGGTAG

>IR1230_00023

ATGAACGTATTACGCAGAAAGTGGCAGGGGCTGCCGCGCGGCGTGGTGGTGTGCATCACGGCGCTGGTGATTTATGTCCCGCTGTTATTTATTGTGGTGCAGAGTTTTCTCTCGGCACCGTTCTTTTCCCGCTCCAAATCGTGGAGTCTGGAAGCCTTCGCCTTTATTTTTACCGACCCGGATTTTTATCTGGCATTAAGGTCAGGTTTTATTCTGGCCTTTGGCCTGGTGATTATCGCCATTCCGCTGGGCGGTATTTTGGCGTTTCTGATGGTGCGCACCGATTTACCCGGCCGGCGGATCATTGAGCCGCTGATCCTGGTGCCGATCTTCGTCTCGCCGATGGTGCTGGGCTTTGGCTACGTGGTGGCCGCCGGGCCGGTAGGCTTCTTTTCCCAGTGGGCGCAGCAGCTGATCGGCTTTGTGCCGTGGAATATCTACTCGATGTTCAGCATTGTGGTCATCGCCGGCTTAACTCACGTTCCGCATGCCTATCTCTATATCTCTTCGGCGCTGCGCAGCGTCGGCTCCGACGTGGAAGAGGCGGCGCGCACCGTCGGCGCCACGCCGCTGCAGGTGATGACCTCGGTGAGTCTGCCGATGGTGCGGCCATCCATTCTTTACGCCTGCGTGTTGCTCTTTTTCCTCGGTCTGGAAGTCTTCGGTCTGATGCTGGTGCTGGGCGACCCCGAGGGCAACATGGTGCTGGCGACCTATCTCTACAAGCTGACTAACAAGCTCGGCACCCCCTCCTACCATCTGATGGCGGCGGTGGCGGTGGTGCTGATCTGCATCACCATCCCGCTGGTGATGCTCCAGCGGCGGCTGATGCGCACCGCCAACCGCTTTGTCACCATGAAGGGCAAGGCGTCCCAGGCGCGGGCGCTGCCGCTGGGCAAATGGCGCTGGGTGGCCGGGGCGGTGGTGGTGGCCTGGCTGACGGTGACCATCGGCGTACCGCTGCTCGGCGTGGCGCTGCGCGCTTTTATCTCCAACTGGGGCGTCGGCGTGTCGCTGTGGGATGAGCTGTCGCTGGCGACGTTCCACAATATCTGGCAGCAGCCCAACCTGCTGCGGGCGATCGTCAACTCGATGGCTATCGGCATCATCGGCGGCGCGCTGGCGGTGATCTGCTATCTGTTTGTCGGCATCGCCATGCACCGCAAAGCGGACAACGTCACCCGCTTTCTCGACTACAGCGTGCTGGTCCCGCGCGCGGTACCGGGGCTGCTGGCCGGCCTGGCCTTCCTGTGGGTATTCCTGTTTGTACCGATGTGGCTCGATCAGTCGCTGAAGCACGGCTGGCTCTCGGCGCTGCCGGTGGCCGACTGGCTGCGGGAGCACCTTATCGTGCAGCTGCGCGCCCTGCGCAACACCATCTTCAGCGTCTGGCTGGCCTACACCGTGGTGTGGATGGCCTACGGCCTGCGGCTCATCTCCTCGACGCTGCTGCAGGTGGGCCCGGAGCTGGAGGAGGCGGCGCGTAGCACCGGCGCCTCGCGGGGGCAGATCACCCGCCACGTGACCGTTCCGCTGTCGCGCTATGGGCTGATTGGCTCCTGGCTGCTGATGTTCCTGATCTTTGAGCGCGAGTATTCCACCGGCGTCTATCTGCTGTCGCCGGGTACCGAAACCATCGGCTCGATGCTGGTGTCGCTGTGGGCGGCGGGGGCCATCGATATCGTCGCGGCGCTCTCCTTTATCAATATTCTGCTGGTGGTGATTGGCCTGGGTATCGCCCTGCGCTTTGGAGTGAAATTACATGATTGA

>IR1230_00024

ATGTATAAGAAAATAAGTGGTTTAGTGGTTTCACTGACGGCCGTTTTCGCCTGTGCCGCCTGGGCGGAGGTGCCTGCGGGCTATCCCGCGGATTATCAAAAAACGATTGATGCCGCCGTAAAAGAGGGCAAGGTGGTGATCTATTCCACGACCGATACCAAAGCGGCCGGGCCGTTAATTAAGGGATTTGAAGCGCAATACCCGGGCGTCAAAGTCGAATATAACGACATGAACAGTACTGAGCTGTATAACCGCTATATCAGCGAACAGGCCGCCGGGGGCGGCAGCGGGGATGTGGTGTGGAGCTCATCGATGGATACCGCGCTGAAATTAGCCACCGAATACGCCGAACAATATGCCTCGCCGGAGGTGAAGCAATTACCCGACTGGGCGGTCTGGCAGCAAAAAGCCTATGGCACTACCTATGAGCCGGTGGTGTTTATCTATAACAAACGGCTGATCCCGCAGAATGAGGTTCCGGATTCGCATACCGCGCTGGCAAAACTTATCGCCAGCCAGGCCGATAAATTTAAAGGCAAAGTCACCACCTACGATATCGAAAAATCGGGCCTCGGCTTTATGCTGGCGGTACAGGACAGCCAGGCCGACGCGAACTATTTTGCCGACCTGGCGAATATCGCCAAAGGCGGCCTGACGGTGCAGTCTTCCACCGGCACAATGATGGAGCGCGTCTCCTCCGGCGAAAACCTGATCGGCTATAACATTCTTGGCTCTTATGCCGAAGCGCGGGCGAAAAACGATCCCTCTTTAGGTATTGCCTATCCGAAAGATTACGTGCTGGTGCTCTCCCGCGTCTCGTTTATTAGCCAGGAGAGCGAACATCCCAATGCGGCAAAATTGTGGCTCGACTATGTCTTGTCGGAAAAAGGCCAGCAGATCCTCGCCAGCCAGGCGGATATTCCCTCAATTCGTCGCGATATTGCAGGTAAAAATGATATCGATGGTATGACCGCGCTGTTGGGCAAAGCGTTAAAACCGATCCCGGTCAACGAAACGCTGCTCGATTATTTACAGCCGCAAAAACGTCTGCAGTTTATTAAACAATGGCGCAGCGCCGCCGCGAAATAA

>IR1230_00025

GTGCCTACAGGACACCGCTTTCTGGCGACGGATCTGCATCAGTTTGTCGTCTCACTGTTTACCCATCTCGGCAGTACCCCAGGGGAAGCCACCCTCGTCGCGGACCATCTGATCGCCGCCAATCTTGCCGGGCATGACTCCCACGGCGTCGGGATGATCCCCAGCTACGTCAAATCCCACGCCGGTGGTTTTCTGCAGCTCAACCGCCACGCCACGGTGACCAAAGACGCCGGCGCCGTGGTCACCCTCGACGGCAACGCCGGCTTCGGCCAGGTGGTCGCCCACGAAGCGATGCAGTTAGGCATTGAAAAGGCGAAGCAGCATGGTATGGCGGCTATCGCCCTGCGCAATGCGCATCACGTCGGTCGCATCGGCTACTGGGCCGAACAGTGCGCCGCCGCCGGGCTGATCTCCATTCACTTCGTCAGCGTCATCGGCGACCCGATGGTCGCCCCGTTCCGCGGGAAGGACAGCCGCTTCGGCACCAACCCGCTGTGCGTGGTGTTTCCCCGCGCGGGCCATCCGCCGCTGCTGCTGGACTACGCCACCAGCGCCATCGCCTTTGGTAAAACCCGCGTCGCCTGGCATAAAGGCGAAGCCGTGGCCCCCGGCTGCCTGATTGACGCCGAGGGTCGACCCACTACCGACCCGGCGGTGATGCAGACCTCGCCGCTGGGCGCCCTGCTCACCTTCGCCCAGCACAAAGGCTATGCCCTGGCGACGCTGTGCGAAGTGCTCGGCGGCGCGCTCTCCGGCGGGCAAACCACCCATCAGGAGAGCCTGCAGACCAGCGTCGACGCCATCTTTAACTGCATGACTACGGTGATCCTGCGCCCGGACGCGTTCGATGCCCCGGACAGCCAGGCACAGACGGAGGCCTTTATCGCATGGTGTAAGCAGTCGCCGCACGATGCCGATGCCCCGGTGCTGGCGCCCGGCGAATGGGAGGCCGCCAACCGCGAAGCGCGGCTGGCGCAGGGGATCCCGCTGGATGCCGGCAGCTGGCAGGCTATCTGCGCCGCCGCGCGCGACGTCGGCCTGAGCGAATCGCACTTCGACCGCTGCCGCCCTCTCGCCTAG

>IR1230_00026

ATGAAAAAAATCACGAGCGTGTGCCCCTACTGCGGGGCGGGTTGCAAACTTAAACTGGTTGTCGACAACAATAAAATCATTCGCGCGGAGGCCGCCGACGGCGTCACCAACCAGAACCAGCTGTGCCTGAAAGGCTACTACGGCTGGGATTTTCTTAACGATACGCAGCTCCTGACGCCCCGTCTTAAACAACCGATGATCCGCTACCAGAAAGGCGGCGCCTTCACGCCGGTCAGCTGGCAGGAGGCGATTCGCTATACCGCCAGCAAGCTCAGGGAGATCAAAGAGAAGCATGGCCCGCGCGCCATCATGACCACCGGCTCCTCGCGCGGCACCGGCAACGAAACCAACTATGTGATGCAAAAGTTCGCCCGCGCGGTGCTGAACACCAATAACGTCGACTGCTGCGCCCGCGTCTGCCACGGCCCCTCCGTCGCCGGGCTACAGCAGGCGCTGGGCAACGGGGCGATGAGCAACTCGATTAGCGACATCGAAAACTCGAAGTGCCTGCTGGTCTTTGGCTACAACTGCGCCGACTCCCATCCCATCGTCGCCCGCCGGGTAATCAAGGCCCGGGACAACGGCGCCAAAATCATCGTCTGCGATCCGCGGCGCATTGAAACCGCGCGCATTGCCGATCGTCATCTGCAGCTCAACAACGGCAGCAACATGGCGCTGGTAAATGCCTTCGGCTATGTGCTGCTGGAGGAGGAGCTGTACAACAAAACCTATGTCGAACGCTATACCGAAGGGCTGGATGCTTACCGCGAGGCGGTGAAGGATTACGCCCCGGAAGCGGTGGAAGGGATTGTCGGGGTCAGCGCGCGGGAAATCCGGGAGGCGATGCGCATGTTCGCCGCCGCCCCATCCGCCACCATTATGTGGGGCATGGGGGTCACCCAGTTTGGCCAGGCGGTGGACGTGGTGCGCGGCCTGGCAAGCCTGGCGCTGCTCACCGGCAACCTGGGACGACCCAACGTCGGCGTGGGGCCGGTCCGCGGGCAAAACAACGTACAGGGCGCCTGCGATATGGGCGTGCTGCCGAACCTGTTCCCCGGCTATCAGGAGGTCACCGACCCCGCCGTGCGGGCGAAATTCGCTGCCGCCTGGGGCATCGACCCGGCGCTGATGGACGACCAGGTCGGCACCCGCATCACCGAGGTGCCGCATAAAGCGCTGACCGGCGAGATCAAAGCCTATTATATCATGGGCGAAGATCCGCTGCAGACCGAAGCCGACCTCGGCCTGGTGCGCAAAGGCATCGAGGCGCTGGACTTCGTGGTCGTGCAGGACATCTTCATGACCAAAACAGCGGAAATGGCCGATGTCCTGCTGCCGGCCACCTCCTGGGGCGAGCACGGCGGCGTCTTCACCTGCGCCGACCGCGGGTTCCAGCGGTTTGAACAGGCGATCCCTCCGGCGGGAAATGTGAAGCGCGACTGGGAGATCATTAGCCTGCTGGCCAGCGAATTGGGCTATCCGATGCACTATGAGAACAACCAGCAAATCTGGGACGAGATGCGCGAGCTCTGCCCGCTGTTCTACGGCGTGACCTGGGAGAAAATGGGCGATATGGGCCATGTCCAGTGGCCGTGCCCGACCCTCGATCATCCCGGTACGCCATGGCTGTATAAAGACAACCGCTTCGACACCCCCTCGGGTAAAGGGCAGCTGTTCGCCACCGCCTGGCGCGCGCCGGCGGAGCGCCCGGATGACGAGTGGCCGCTGGTGCTCTGCACCGTGCGGGAAGTCGGGCATTATTCCTGCCGCTCGATGACCGGCAACTGCGCCGCGCTGCAGTCGCTGGCCGACGAGCCGGGACGGGTGCAGATGAATCCCGCGGATGCGCAACGACTGGGGATCGCCGACAAACAGCTGGTGTGGGTCAGCTCCCGGCGCGGGAAGGTGATCTCCCGCGCCGACCTCAGCGATCGCATCAATCCGGGGGCGGTCTATATGACCTATCAGTGGTGGGTGGGCGCCTGCAATGAGCTGACCCAGGATAACCTCGATCCGATCTCCAAAACGCCGGAAACCAAATACTGTGCGGTGAAAGTGGAAGCGATTGCCGACCAGCAATGGGCGGAGCGCTATGCCTGGACGGCCTACAGCGACATGAAAGCGCGACTGAAAGCCGCTGCCGACGTCTGA

>IR1230_00027

ATGGCCGTATCGCCGTCACTGATCGCCGAAGCCGTTGGCTGGCGACGTGAATTTCACGCCGCGCCTGAGCTCGGATATCAGGAGCAGGAGACCTCGCGCCGCGTTGCGGAGCTGCTGGCCTCGTTTGGGCTGCAGGTGCACCGAGGGCTGGCCGGCACCGGCGTCGTGGCGACGCTGGAGAACGGCCCCGGGCCGGTTATCGGCCTGCGCGCCGATATGGACGCCCTGCCGATCACCGAGCTGGGGAGCGTCAGCTACCGTTCACGCCGTCCCGGGGTGATGCACGCCTGCGGCCACGATGGCCATACCGCGATGCTGCTGGCGGCCGCCGCCCATCTGGCGCAGACCCGCCATTTCAGCGGCACCGTCCACTTTGTCTTTCAGCCGGCGGAAGAGAACCTCGGCGGCGCGCGCAAAATGGTGGAGGAGGGGTTGTTCGAGCGCTTCCCGATGGACGCCATCTACGCGCTGCATAACTGGCCGGGGATCCCTCTTGGCGAAGTGGCGCTCAGCGATGGCGCGATGATGGCCTCGCTGGACGCCTTCGAGATCACCCTGCGCGGAAAGAGCTGTCATGCGGCGATGCCGGAGAGCGGGGCCGATCCGATCGTCGCGGCGGCGCAGCTGATAATGGCGCTGCAGACCATTCCTTCCCGCCGCTTGTCGCCGCAGGATTCCGCGGTGGTCAGCATCACCCAGATAAACGGCGGCGAGGCGATCAACGTCCTACCGGACACCGTGGTGCTGCGCGGTACCTTCCGCTGTCTGAGCAATCGCGTTCGCGCCCGCGTGCGGGAGCTTATCGAAAGCTATGTGGCGACCCAGCCGCAGGTGTCCGATGTGCAGGGCGAGATTAGCTGGTTCCCGGGTTATCCGGTGACCAAAAACCATGCGCTGCAGGCGCAGCAGGTGCGCGAGGTGGCCGTCGCCACTCTCGGCGCGCAAGCGGTGCGCTGGAATCAGGCGCCGTCGATGGCCTCCGAAGATTTTGCCTGCATGCTGGAGGCCTGTCCGGGGGCCTATTTCTGGTTAGGCACCGACGGCGAAACCCCGTCGAAGCCGCTGCATAACGCCAGCTATGACTTTAACGATGCGCTGATCGGCCCCGGCGTCGCCATGTGGGTGGGGCTGGTGGAGAAACAGCTGCCGGCGGCGTAA

>IR1230_00028

ATGTTTAACCCAGACCAGAATCGTCTTGCCCCCACGCTGGCGATGATCATGGCCGCGTCGCTTGTGGGATTTATCACCGGCTATACGGTGCCGTTAATCAGTCTTGAGCTGGCGCAGCAGCAGATCGCGCCGCTGTACGTCGGCCTGCTGGCCGCCCTGCCGCCCGCCGGCATGATGATCTCCTCTTTCCTCTCTCCTGCGCTGTGCCGGCGCGTTGAAATGGGCGTGCTGCTGAGCGGCAGTCTTATCCTGCTGGCGCTGGCGACCATCGCCTCATGCATGACTACCGATATGACGCTGCTTTTGCTGCCGCGCCTGCTGACCGGCCTGGCCTCCGGGGTCATTATCGTACTCGGCGAAAGCTGGATCACCGGCGGCGCGGCGGGCAGCCAGCGCGCTACCCTCACCGGTCTCTACGCCTCGGCCTTTACCGGCTGCCAGCTGGCCGGGCCGCTGCTGATCTCCGTCGGCCCCGCCTGGCAGACATCAGCGCTGATAGCGATTGTCGCGGTCACCGCCGTCTGCCTGCTGATGCTGCGCCATCTGCCCACCGGCACTCGTGAAAGCCTCGGGGAGCGCGCCAGCTGGCGCAGCCTCGGCGCCTTTCTGCCGGTACTGGCCTCCGGGGTCTTCTGCTTCGCCTTCTTTGACGCCAGCATCCTCGCCCTGCTGCCGCTGTACGGCATGGACAAAGGGCTGAATGAAGGGCTGGCGGTGCTGCTGGTCACCGTGGTGCTGACCGGAGATGCGATGTTCCAGACCCCGCTCGGCTGGCTGGCCGACCGGGTCGGGATTCGCCGCGTGCATCTGAGCTGCGCGGTGGTTTTCAGCCTGTCGCTGCTGGCCCTGCCGCTGATGCTCGGTTCGCGCATTCAGCTGATGGCCATTTGTCTGCTGCTGGGCGCCGCGGCGGGCGCGCTTTATACCCTGTCGCTGGTGCGGGCGGGGAAAACCTTCAATGGGCAGAAGCTGATTATGATTAACGCCCTGTTCGGCTTTTTCTGGTCCGCCGGCAGCGTTGCCGGGCCGGTAGTCAGCGGCATGCTGATCGGCATCACTGGTTACGATGGCCTGATCGTCACCCTGGTGGCCAGCGGCGTGCTGTTCCTGTTGATTCAGTGTCTGTGCAAAAACGAGAAAACCCTGCTTGCCAGTGAGCAGGAAGATGACATGGACGAGGCGACGGAGTCCGCACGGTAG

>IR1230_00029

ATGGAAAGTAATACCTTACATCGTGGCAGATTGATCGATCATATTCTGCTGGTTGTCGAAGACTTTGAGGCAAGCAAAGACTTCTATACCGCCGTACTGTCGGCGCTGGAGATCCCGGTTATCACCACCGCCAATGACTACTTGCTGGCCGACGAACTGGTGGTGGCCTCACGCCACAGCCCGGAGGCTGCCGGGAAACTGACTGGGCGCCACCACCTGGCATTCCAGGCCAGAGATCGTGACAGGGTGGACGCTTTTTATCATGCGGCGCTGACCCATGGCGGACGTGATAACGGCGCGCCTGGCGAACGACACTATCATCCTGGTTACTATGCCGCTTTTGTGCTGGATCCGCAGGGGAATAATATTGAGGCGGTGTACCATGGCAAGGCTGAGCGCAGCGCCGGGTCTGTTGCGATCAGCTTCACGCAGTAA

>IR1230_00030

ATGAAATTAGCGTTGGGACCGTACATCCGTTTTAGCAGAATCTTGTTATTTCCGCTGGCGATGGTTATCTATGATTTTTCTGCTTATCTGACTACCGATCTCATTCAGCCGGGTATTATCCACATCATCCGGGAATTTGAGGCTGATGTTGCGTTAGCCCCGGCCTCCGTCAGCCTCTATTTAGCCGGGGGGCTGGCATTACAGTGGCTGCTGGGGCCGCTCTCCGACCGCATTGGTCGCCGCCCCGTACTACTGACCGGAGCCATCATCTTTGCTTTGGCCTGCTTCAGCATGATCTTTGTCACGTCGATTGATCAATATCTTATCGCCCGTTTTATCCAGGGAACCAGTATCTGCTTCATTTCGACAGTCGGTTATGTCTCCATCCAGGAGGCTTTTGATGAAAAAGAGTCTATCCGCATTATGGCGGCGCTGACATCCATCGTTTTGCTGGCGCCGGTAATCGGGCCCCTGGCGGGCGCTGGCCTGATGAATTTCCTGCACTGGAAGTTATTGTTTGCGATCATCGGCGCCATGAGCTTGCTGGCCTGGGCGTTACTGATTTTTAACATGCCAGAAACGGTCACCTCTCAGGGCCGGGGGTTTCGTCCGGGAGAGGTATTTTCCGAGTTTGTCCGCGCCTTTAAACAGCCGGTGGTGCTCACGGGCGCGCTGGTGTTGTCATTCAGTAACCTGCCGATAATCACCTGGGTAGCACTCTCTCCGGTGATCCTGATCGATGACGGCGGGATGTCGCGCGGCGCCTATGCCTGGACCCAGGTGCCTGTCTTTGGCGGCGTTATTATTGCCAGCATCATCGTGGCCCGTTTTATCAAGGATCCGACCTCTCCCCGCTTTATCTGGCGCACTATCCCGATACAGCTGACCGGGTTGTTGGTATTACTGGCGGGAAATATTGCATGGCCCCATACCTGGTGGTGGTCCGTTTCAGGAACAAGTCTCTATGCCCTCGGAACAGGCCTGTTGTTCCCCGTATTATTCCGTTTTGCGCTTTTTTCCCACTCCCTGCCGAAAGGGACGGTTTCAGCCACCATCAACATTGTGGCGCTCAGTTTTATGGCGGCCTCGGTGGAAGTAGCGCGCTGGGTTTACTTCCAGGCTGGCGGCAGAATTGCTTTTCACTGCCTGGCGCTGATAGCGGGAATCGTCGTCATCATGCTGGTCTCGCGCTTGTTAAAACTCCGTCAACAACATCTCCTTCAGCCAGCCTGA

>IR1230_00031

ATGTTTTCATACATTATGCTGGGAACGAACGATCTGCCACGCGCCATCAAGTTCTATGACCCGTTAATGGCGCTGCTGGGGCACGTCAAAGCCGGCCGTAACGAACAGGGGGCGTCCTGGGGCACCTTCAACGGCAATCACACCTGCGGGCTGTGCGTGGGCGTTCCTTTTGACCAGCAGCCTGCCGGGGTGGGTAATGGAACGATGGTGGCGCTTAACGCCCGCTCCGTCGCACATATTGCGGAACTGCATGCCCTCGCCCTGCAACTCGGCGGACGTGACGAAGGCGCTCCCGGTCACCGGCCGCAGTATGGCGACGGTTTTCACAGCGCCTACGTCCGCGATCCCGACGGCAATAAGCTGGCGTTTGTTTACTATGCGTCTGCAGATTGA

>IR1230_00032

ATGACAGTTACATGGACGGTCACGCCCGTGGGGTATCAGCACATTGCCAAACGCTGCCCCGCCTGCAATGTAAAACGCGACTTTGCGCCTTCCGGCGCCATCCGGGTGAATTCGCAAAAGAAGCTGCTGGATATCTGGAGCATCTATAAATGCACGCACTGCGATTACACCTGGAATATCGCCCTGTTTTCCAGGCTGCACGTCAGCAAGATAAACCGCGAACTGCTTCAGCGCCTGCTGAAGAACGATGCAGCCATGGTTCACTATTATGCCGCTGACCTCGCGACGCTGAAGCGCAACCGCGCCGAGCCGTCGGGGCAACCTGATTTTCGTATTCACGAGCAATGGTCGGTTACGTTGATGGCATGTCAGCGGATAACGGTGCGCGTCAGAGTCAGTCAGCCATTTCGCATCAGCCTGCTATCAATACTGAAAAAACAGCTCAAGCTGAGTACGGCTGAAATCCGGTGGTTGGTCGCGACAGGGCATATCGAGGGTTTTCCACTAAAACAGCTCAAGACGAAAAAGCTGAAGGCGATGGAATATGATTTTCAGCTGGCGGCGGAAACGCTGTATGCGCGACGCCGGATTACGTTATCACTTTGCGGTAGATAA

>IR1230_00033

GTGATTCACATTAATAACTTACATAAGCGCTTCGGCGACAGCCACGTGCTGCGCGGCATCAGCTGCGATATTAAACCGCAGGAAGTGGTGTGCATCATCGGCCCATCCGGCTCGGGGAAAAGCACCTTCCTGCGCTGCATGAACGCCCTGGAGACGGTCAGCGAAGGCGAGGTGGTGGTTAACGGCTTTGCCGCCCACGACCGGACCACCGACCTCAACAAGATGCGCGAAAGCGTCGGCATGGTGTTCCAGCGCTTTAACCTGTTCCCGCACATGACGGTGCTGGAAAACCTGATCATGGCGCCGATGAATCTGCGCAATATGCCGCGCCAGCAGGCGGTCGACCTGGCGGAAGCCTTGCTGGCGAAGGTGGGCTTAAGCGATAAGCGCGACGCCTGGCCCTCGAGCCTGTCCGGCGGCCAGCAGCAGCGGGTAGCCATCGCCCGCGCACTGGCGATGAAGCCGTCGATAATGCTGTTCGATGAGCCCACTTCGGCGCTGGACCCGGAGCTGGTGGGCGACGTGCTGGAGGTGATGAAAAACCTCGCCAGCGAGGGGATGACCATGGTGATCGTGACCCACGAAATGGGCTTTGCCCGCGAAGTGGCCGACCGGGTGATCTTCATCGACCAGGGCGTGATTCAGGAAGAGGGCAAGCCGGGGCAGATCTTTAGCGCGCCGACCAACCCACGCACCGCCGCGTTCCTCAGCAAGGTGCTGTAA

>IR1230_00034

ATGACGGGATTCCGTTGGGAGATCATCGAGGAGTACGGCCCGCTGTTTGTGGACGGCGCCCTGATGACGATTAAATGTACCATTATCTGCGTGATCCTCGGGACGCTGTGGGGGTTAACTCTCGGTCTCGGGCGGATGGCGAAGGCCGAGCACGGGCCGTGGAAATATATTCTCCGCTACCTGGTGCAGTTTCCGGTGCGCTTTTACGTCAGCGCCTTCCGCGGCACGCCGCTGTTCGTGCAGATTATGGTGGTGCACTTTGCGCTGGTGCCGCTGTTTATCAACCCGCGCGACGGCCTGCTGGTGACCAGCGGCCTGATGAGCGCCGACTTTGCCCGCGAGCTGCGCGCCAGCTACGGGGCGTTCCTCTCCTGCATCGTGGCGATCACCCTTAACGCCGGGGCCTATGTGTCGGAGATTTTCCGCGCCGGCATCCAGTCGATCGACAAAGGGCAGATGGAGGCCTCCCGCGCGCTGGGCATGCCGTGGTGGAAAACCATGCGCAAGGTGATCCTGCCGCAGGCGTTTCGCCGTATTCTGCCGCCGCTGGGCAATAACGCCATCGCCATTGTCAAAGACTCGTCGCTGGCCTCGGCGATCGGCCTGGCCGATCTGGCCTATGCCGCGCGCACCGTCTCCGGCGCCTACGCCACCTACTGGGAACCCTACCTGACCATCTCTCTGGTCTACTGGGTGATCACCTTCCTGCTGGCGCAGCTGGTTAACCGTCTGGAAAAGAGGTTTGGCAAAAGTGATTCACATTAA

>IR1230_00035

ATGGTCAAGAATTTGCTCAAAGCGTGTTGCATGATAGCCGCGCTGACGGCTGCCGGACAGGCGGCTGCGGAAACCTATACCGTCGGCTCCGGCGGCACCTACCGGCCGTTTGAATTTGAAAACAGCCAGAAACAGCTGGAAGGTTTCGACATCGACATCATCAAAGCCATCGCCAAAGCCGAAGGCTTTGACGTTAAGCTGGTCAATACCCCATGGGAAGGCATCTTCGCCACGCTGAACACCGGCGACCGCGACATCATCATCTCCGGGATCACCATCACCGATAAACGCAAACAGATGGTCGATTTCTCCGCGCCGTACTTCCCGGCCGAACAGTCTATCGTCGTCGCTCAGGACTCGCAGGTGGATTCACTGGCGGCCCTGAAAAACGAGAAGGTCGGGGTGGTGAACTCCAGCACCGGCGATATCGTGGTTTCTGAGGTGCTGGGTAAAAACAGCACCGCGATCAAGCGTTTCGACAATACCCCGCTGATGCTCCAGGAGCTGTTTGAGGACGGCGTCAGCGCCGCGGTTGGCGACGTCGGGGTGGTGAAGTACTACATCAAACAGCACCCGGAGAAGCAGTTCAAGCTGGTGCCGGATGCCAAATTTGAGCGTCAGTATTTCGGTATTGCGGTGGCGAAAGGCAACAGCGAACTGCTGGGCAAAATCAACGCCGGGCTGCAAAAGATCGTTGCTGACGGGACTTACGCCAAAATCTATAAAACCTGGTTCGACGACAACGTGCCGACGCTGCCAGCGCAATAA

>IR1230_00036

ATGAAAAGCGCGGTAAAAAAAGAGAAGGGTAAAGTCGATCTGTTCGGCGAACGCTATCGCGCCCGGGCGCACCAGCTCTCCCCCCGGCTGCGGGCGGTGGTCAGCTATATCAACGACAACCGCGAGGTGGTGCTGGAGCATACGGCGATGGAGATCGCCACCGCGACCCAGACCTCCGACGCCACGGTGGTGCTTGCCATCCAGGCGCTGGGCTTCGCCGGGCTGCGCGAGCTTAAGCAGACCATGGAGCGCTGGTTCGGCACCTCGGTCACCTCGGCGGAAAAGATGCGCTCGACGGTTACCGCGCTCTCCAGCGACGTCAACTCCAGTATTGATTTTGTGCTTGAAGGCCATCAGCGGGTGTGCGAGGTCCTGTCGCGGGCCGACAACCGCGCGGCGGTGGCTCAGGCGGTGGCATTGCTCAGCGACGCGCGCCAGGTGGGGATCTTCGGTATCGGCGCCTCGGGGATCCTCGCCGAATACACCGCCCGGTTGTTCAGCCGTATTGGCCTGCCGGCGTACGTGATGAACCGCACGGGCTTTAGCCTTGCCGAACAGCTTATCGACCTGCAGCGCGGCGATGTGCTGATCATGATGGGGCAAAAATCCCCCCATCGCGAAGGGTTGACCACCCTGCGCGAGGCGAAGCGGCTGGGTATTCCGACCATCCTGCTGACCCAGGCGGTGGACTCGCGCTTCAGCCAGGAGGCGCAGGTGGTGATCGACGTCCCGCGCGGTGGGGACAGTAGCCGGATGCCGCTGCACGGTACGGTGCTGGTCTGTCTCGAGATGATTGTCCTGTCGGTGGCCTCTACCACGCCACAAAAAACGGTGAAATCCCTGAAACGAATCAACGATCTGCATCGGGCTATCGGTAAATCCGGCGGTAAACGGGGCCATTGA

>IR1230_00037

ATGCGTTGGTTCGTCTCATTGCTGCTGTTGCTGACGGGGGCCGTCAGCGCCGCCGCCCCACAAACTCAAACTTTCACCGATGATTTAGGCCGCACGGTCACCGTCCCGCTGCATCCGCAGCGTATCGTTTCGATGCACGATCTCGACATTACCATTCCGCTGATTGAGCTCGGGGCGCCGCCGATCGCCAGCCACGGCCGGACGCGCCCGGATGGTAGCCACTATCTGCGCGCCAGCGCCCAGCTCACCGGTGTCGATTTCGACAACAGCGATATCCGCTTTATTGGCACCGCCGATATCGATCTGGAAGCGGTAGCCGCAGCCAGGCCGGATCTGATCATCACTGAGCCCAGCCGCCACGTGTCAGTGGAGCAACTGGAGAAGATTGCCCCGACGGTGAGCATCGACCATCTGCAGGGCAGCGCCCCGGAGATTTACCGCAAGCTGGCGCAGCTGACCGGCACCCAGCCGCGGCTGGCGATCCTCGAGCGCCGCTATCAGGAGCAAATCAAGCAGCTGAAAGCGATGGTCAACCCACCGCAGTACAGCGTCTCAGTGATCCAGGCGAACAACGGCAAGGTCACGGTGCATCATTCGTATCATGCGCTGGGCCGGGTGCTGCGCGACGCCGGGTTCCGCTTTCCGCCGCTGATTGAGCGGATCCCCGATGGTCAGCGCATTGACGTCAGCGCGGAACAGCTGCCGGAGCTGGACGCTGATTTTGTCTTCGCCACCTGGCGGTCGGATACCGGCGGTAAGCCGCAGGATGAGCTGCAGGCGATGGAAGGCGTGATGCCCGGCTGGTGCGACTTTATGCGCGCCTGCCGCACCGGGCACTATATTTTACTGCCGCGGGAAGAGGTGATCTCCAACTCCTACGCCGCGCTGTCCCTGATGGTGGCCCAGGTGCAGTCGCATATTGCCGGCAGGCCGATCCCGGCGGAGGCCAAATGA

>IR1230_00038

ATGACGCGCGCCGTTCACCGGGCCGGTTTTCGGCCGCTGGCTTTCGCCTCCCGTCATCTGCTGCTGCGTCCTGCGGCGTTAAAAATTGCCGCCAGCATAGTGCTGACGCTGCTGGCGCTGGGGCTGTACAGCCTTAGCCGCGGCAGCTATCCGCTGCCCGCGTCGACGCTGGCGCGGGCCCTGCTGGCGCCGCAGGAGATGGGCGAGCAGCCGCGCTTTATTCTGTTTGATATCCGCCTGCCGCGGATCCTGATGGCGCTGCTGTGCGGGGCCATGCTGGGGCTGGCCGGCGCGGCGATGCAGAGCATCACCCGTAACGGGCTGGCCGACCCGGGGCTGATCGGCGTCAAAGAGGGGGCCAGCATCGTGGTCCTGGCGCTGGTGCTGTTTTTCCCGGCGGTAGGCCTGGTCTGGCGGCCGCTGGCGGGGATGGTCGGCGGCATAGCGGTGGCCCTGCTGGTCCTGACGCTGGCCCGCGACTGTTCGCGGCCGCGCTTTATTTTAATTGGCATCGGCGTCTCCTGGAGCCTGGCGGCCGCGGTCGGCATCTTTATGACCACCGCCGACGTACGCGACGTGCAGACGGCGATGATCTGGCTGGCGGGCAGCCTGCAGGCCGCCACCTGGCCGCTGCTGGCGGTGGCTTTTTGCTGGGCGCTGCCCGGGGCCATCATCCTGTTCTGTACCGCCCGGGCGGCGGACGTTGCGCTGCTGGGCGACCGGACCGCTGTCGGCCTCGGCGTACGTCTGCAGCAGCTGACGGTGCTGCGCTTTTTTGCGCCGGTGCTGCTGACCTCGGCCAGCGTCTCCTGCGTCGGCAGCCTTGGATTTGTGGGCCTGATGGCGCCGCATATGGCGCGTTTCGTGCTGCGCGGCGGCCAGGTATCGCTGCTGTGCGGCAGCGCGCTGATTGGCGCCTTGCTGGTGCTGGCGACCGATACCCTTGGCCGGCTGGCGTTTGCCCCGCTGCAGATCCCGGCGGGGATTGTCATTGCCCTGGTGGGCTGCCCGTTCTTCGTTGTCTTGCTCTGGCGTCGCCGTGATGCCCTTTAA

>IR1230_00039

ATGAGCGTGGCCATCCTGCTTTCCGCGCGGCGTCGCCCCCGCCCCCGGCTGGCGCTGCTTCTGCTAACGCTGCTGCTTATCGCCGCCTCGCTGGTGCATCTTGGCTTAGGCGCCCGCTGGATCGCCCCGCAGACGGTGCTGCAGGCGCTGCTGGAGTACGATCCGCGCAACTTTGAGCAGCGGATTATCATCGATCTGCGTCTGGTGCGGCTGGCGGCGGCGCTGCTGACCGGCGCGGCGCTCGGCGTCGCCGGGCTGCTGCTGCAGACGGTGATCCGTAACCCGCTTGGCGAGCCGCATATTCTCGGGCTTAATGCCGGCGCCTCGCTGGCGGTGGTGGCGACCTCGGCGCTGGGCCTGTCCTTCGGCGCTTTCCCCGCCGGGCGCCCGCTGACCGCCGCCTGCGGCGCGGGGCTGCTGTTCGGTGGCGTGATGGCGCTGGCCTCTGCCGGGCGCGGCGGGGCGACGCCGCTGCGCATCACGTTGTGCGGAGTGGCGCTGTCAGGGTTTGCGTCGGCGGTGACGGCGGCGATATTAATTCTCGATGAACAGACGCTGCTGGCGATGCGCACCTGGCTGGCCGGCGACCTGGCCGGACTGAACTGGAGCACGCTGCAGACGGCGCTGGTCCCGGCGCTGATCGGCCTTGGCGTCGCGCTGCTCATTGCCCCACGGCTTAATGTGCTGGCGCTGGGGGATAAGGTGGCGCTGGGCCTTGGCGTTAATCTCGTCCAGACGAGGCTGCTGGGCCTGCTGGCGATTGCGCTGCTGTGCGGCGCGGCGGTGGCGGTGGCCGGGCCGATCGGCTTCGTCGGGCTGGTGGTGCCGCACGTGGTGCGCCGTCTGGTGACGGAAGACATCCGCCTGGCGCTGCCCCTGGCTGCGCCGGTGGGAGCGCTGGCGCTGGTGCTGGCGGATATCGCCGCCCGGACGCTGGTCGCGCCGCAGGAGCTGGCGACCGGGGCGATGACCGCCCTGGTGGGTGCGCCGTTGTTTATCTTTATCGCCGCGAGGTTTTTTAAATGA

>IR1230_00040

ATGCCACAGCGGGTGAAACCTGCAGAGCAGGGGATCGTACTGGATGCCCTGTCGGCCGGCTACGGCCAGACCCTGATCGTTGACGATATCAACCTGACCATTCCCACCGGCAAAATGACGGTGCTGGCCGGGGCGAACGGATCCGGCAAATCCACGCTGCTCTCGACCATCGCCAGAATGCTGAAACCGCTCGGCGGCTGCGTGCGTCTGGACGGGAAAGCCATTCACCAGATGCCGACGAAGACCGTCTCGCGGCAGCTGGGCATCCTCCCGCAATCGCCGTTGACCCCGGAAGGATTGACCGTTTTCGAGCTGGTCTCGCGCGGGCGCTACCCGTGGCAGGGGCTGATGCGCCAGTGGTCCGAGGCCGATGAGCTGGCGGTAGAGGAGGCGCTGCGCTTAACCGGTACCGCCGAATTCGCCCATCTGCCGGTGGACAGCCTCTCCGGCGGCCAGCGTCAGCGCTGCTGGATCGCCATGGCGCTGGCCCAGCAAACGGCCACCATTCTGCTCGATGAGCCGACCACCTGGCTCGATCTGCGCTATCAGGTCGATATCCTTGAGCTGCTCCAGACCCTCACCCGCGAACATGGCCGCACGGTGGTGACAGTGCTCCATGACCTTAACTTTGCGGTGAACTACGCCGATCTGCTGGTATTCCTTAAACAGGGGCGCATCGCCGGCACGATTAGCGACAACGACGTCTGTAGCCCTGAGCTGATTAAACGCGTGTTTGACGTTGACGTGCAGATGTCGATTAACCCGCAAACCGGCAAACCGTTCTTTATGCCGTTTCGCGCCCGCCAGGCAGCGGCACAATGA

>IR1230_00041

ATGGCCGCCGACAATCGCGCGGCGAAAAGTACTAACGACGCGGAAGAGACTATCACCGTAGTGGCCGACGGCGCGCAGCAAAGCGCCACCAGCGGCTATCAGCCGCTCAGCTCCGCCACCGCGACGCTAACCTCGATGCCGCTGCTGGATATTCCCCAGGTGGTCAATACCGTGAGCGATCGGGTTCTCGAAGATCAGCACGCCACCTCACTCGATGAGGCGCTGTATAACGTCGCTAACGTGGTGCAGACCAACACCCTCGGCGGTACCCAGGACGCCTTTACCCGCCGCGGCTTCGGCGCCAACCGCGACGGCTCGATCATGACCAACGGCCTGCGCACCGTGCTGCCGCGCAGCTTTAACGCCGCCACCGAACGGGTGGAAGTCCTGAAGGGGCCCGCCTCGACGCTGTACGGTATCCTCGACCCCGGCGGGCTGATCAACGTCATCACTAAACGGCCGGAGCGGCAGTTCTCCGGTTCGGTTTCCGGGACCTCCACCAGCTTTGGCGGCGGCACCGGCAGCGTCGACATCACCGGCCCCATCGAAGGCACAAATCTGGCGTACCGACTGATCGGCGAATATCAGAATGAGGATTACTGGCGCAATTTCGGTAAAAACAAAAGCAGCTTTATCGCCCCTTCCCTGACCTGGTTTGGCGAGCGGGCAACGGTGACCGCGTCCTATTCGCACCGCGACTACAGCGCCCCCTTTGATCGCGGAACTATCTTCGATCTGAATACCGGCCATGCGGTTAACGTCGATCGCAAAACCCGCTTCGATGAAGCGTTTAATATTACCGATGGCTATTCCGATCTCGCTCAGCTCAACGCCGAGTATCGCCTTAACGACGCCTGGACCGCGCGCTTCGACTACAGCTACAGCCAGGATCATTACAACGATAACCAGGCGCGGGTGATGGCCTATGATTCGGCGACCGGCAACCTCACCCGCCGGGTCGATGGTACCCACGGTTCAACGCAGAAGATGCACTCCACCCGCGCCGACCTGCAGGGCAACGTGGTAGTGGGCGGCTTTTATAACGAGCTGCTGACCGGCGTCGCCTATGAGAATTACGATCTGCTGCGCACCGATATGCTGCGCTGTAAGAACGTTAAAGGCTTTAACATCTATCATCCGGTCTACGGCACTCTCGACACCTGTAATACCGTCTCCGCCTCCGACAGCGACCAGCGGATCCAGCAGGAGAGCTATGCCGCATACGTGCAGGACGCGCTGTACCTGACCGACAACTGGATCGCCGTCGCCGGCGTGCGCTACCAGTACTACACCCAGTACGCCGGTAAAGGCCGACCGTTTAATGTCAATACCGACAGCCGCGATGAGAAATGGACGCCGAAAGCCGGCCTGGTCTACAAGATCACGCCGAACGTCTCCCTGTTCGCCAACGTCGCCCAGTCGTTTATGCCGCAGTCGTCGATCGCCAGCTATATCGGCGAGCTGCCGCCGGAAGAGTCCACCTCTTACGAAGTGGGCGCCAAATTCGACCTGTTAAACGGCATTACCGCCAATATCGCGCTGTTTGATATTCACAAGCGTAACGTGCTGTACACCGAGAGCATTGGCGATGAGACGGTGGCCAAAACGGCGGGCAAAGTGCGTTCCCAGGGCGTGGAAGTGGATCTGGCCGGGTCCATCACCGATAACCTCAGCGTGATCGCCAGCTACGGCTACACCGACGCCAAAGTGCTGGAAGATCCGGATTACGCCGGGAAACCGCTGCCAAACGTACCGAAACATACCGGTTCGCTGTTCCTGACCTATGACATTCATAACGTCTATAACAGCAATACCCTGACCGTCGGCGGCGGCGGCCATGCGGTCAGCAAGCGTTCCGGCACCAACGGCGCGGATTATTATCTGCAGGGCTATGCGGTGGCGGATGTGTTTGCCGCCTATAAGATGAAGCTGCAGTATCCGGTGACGCTGCAGGTGAATGTGAAGAACCTGTTTGATAAGACCTATTACACTTCCTCGATCGGCACCAATAACCTCGGCAACCAGATTGGCGACCCGCGCGAAGTGCAGTTCACGGTGAAGATGGATTTTTAA

>IR1230_00042

ATGGTAACCGAGACGATAGCGCGCGGCGCGGCGGTATCCATCAGCCATCTTCATCATGCGTTTACGCTTGGCAAACAGACGGTACCGGTGCTGGAGAATATTAGCCTGCAGCTGCGCCCGGGGGAAAGCGTGGCGCTGCTGGGCCCTTCCGGCTGCGGTAAATCAACCCTGCTGCGGCTGCTGGCGGGGCTGGAAGCGCCGCAGAGCGGACAGATGCAGATCGACGGCGCGGCGCTCGGCGCCCCGGGGCCGGAGCGAATTCTGGTGTTTCAGGATCCGACGCTCTATCCGTGGCTGACGGTGCGGCAGAACGTGCTGCTGGGCCCGCAGGCGCAGGGGAAAAAGGGGCTGGAGGCGAAGGCAGACGCGTTGATTGACCGCATCGGGCTGCAGGCCTTCAGCGAGGCGTGGCCGCGGCAGCTGTCCGGAGGGATGGCACAGCGTGCGGCGCTGGCGCGCGCCTTGTTGAACGAACCGCGTCTGCTGCTGCTCGACGAGCCGTTGGGCAAACTCGATTCGCTGACCCGCATCAGTATGCAGCGGGAGCTGATCGCCCTCTGGCAGCAGCAGGGGTATACCAGCCTGCTGGTGACCCACGATATCGAAGAGGCGCTGCTGCTGTGCGAGCGGGTGCTGGTGATGTCGCCGCGTCCAGGGCGGATTATCGCCGAGTTTGCGCTGCCGCTGGCTTTTCCCCGTCATCGCGATAACCCGCAGCTGCTGCAGCATCGTCAGGATATATTGCGCATCCTCGGCCAGGAGGCGGATTGGTAA

>IR1230_00043

ATGCCAACGTATTCGCCTGAGCAGGCTCTGGCGCGCCCCACCGGCCGCGCGCCGCTGTGGGGAGAAGGGCTGCTGGCCGCCGCGCTGTGGCTGCTGGGCGGTCTGTTTACTCTTACCTGGCCCGACGCCGGGCGGCGCTGGCCGTTTAGCGAAGGCTGGGCGCTGGCGCAATTCACCCTCGGCGGCGGCTTGCTGCTGCTGGCGCTGAGCTATCGCTACTGGCGGGCGCGCGGCGCGCGTGTGCTGCATGCCGGCAAGTGGCTGGCGCTGCTGCCGGTGCTGTTTGCTGTCTGGGAAGGGCTGACCGCCAAAACTGCCGTGTTGCCGGTACCATTCTTCGCGCCGCCGCAGGCGCTGATCGAGGTGCTGCACGACGACTGGCCGCGCCTGCTCGACAGCCTGCTGCACTCCCTGGGGCTGCTGGGGCTCGGTGTGCTGCTGGGTACCAGCAGCGGGTTCATCACCGGTCTGGCCATCGGCTGGTCGCAACGGATTGGCTACTGGGTGCATCCGGTGTTACGTCTGCTGGGGCCGGTGCCGTCCACCGCGCTGCTGCCGCTGTGCCTGTTTATTTTTCCCTCGAGCTTCGGCGCCAGCGTGTTTTTGATCGCGCTGAGCACCTGGTTCCCGGTCACGGTGCTGACCTGGTCCGGAGTGATGGGCATTGATAAGGCGTGGTACGACGTGGCGCGGACCCTCGGCGCCAGCCAGCGTTTCCTGATCCTGCGGGTGGCGATCCCCGCCGCGTTGCCGAACGTCTTTGTCGGCCTGTTTATGGGGCTCGGCGCGTCGTTTTCGGTGCTGATTGTCGCCGAAATGGTCGGGGTGAAGTCGGGGATCGGCTTTTACCTCCAGTGGGCCCAGGGCTGGGCGGCCTATCCGAATATGTATGCCGCGCTGCTGGTGATGGCCTTGTTATGCTCCGGCCTGATAAGCGGATTATTTATGCTGCGTGACCGGCTGCTGAGCTGGCAGCGAGGAGGGATGCAATGGTAA

>IR1230_00044

ATGACCAGAACCCCTTTTTCCCGCCGCCGTTTTTTACAGCTCAGCGGCGGCCTGGCGCTGGCCGGGGTCTCTCTGCCGCTGTGGGCTCATGATATGGCGGACATGGCCGGCGGCGATGCCCATCCGGCGCTGCGCCTGCCAGAGCCCTACAAGATCAAGCTGGCGATCAACAAAAGCGCGGTATGCCTCGCGCCGGTCGCCGTCGCCGAGCAGCAGAAGATATTCAGTAAATACAACCTCGACGTTGAGTTTGTTAACTTCGGCAGCTCCACCGATGTGCTGCTGGAGGCGATCGCCACCGGTAAGGCCGACGCCGGGGTGGGGATGGCGCTGCGCTGGCTGAAGGCGCTGGAGCAGGGCTTTGACGTCAAGCTCACCGCCGGGACCCACGGCGGCTGTCTGAACCTGCTGACGGCTAAAGACTCGCCGTTCGGCGGCCTCGAGAGCCTGAAGGGGCAAACCATCGGCGTCACCGACATGGCCGGACCGGATAAAAACTTCTTCGCCATTCTGCTTAAGCGCCACGGTATCGACCCGATCAGCGACGTGCAGTGGAAAGTCTACCCGGCGGATCTGCTCAGCGTGGCGCTGGACAAGCGCGAAATCGCCGCCATCAGCGGCAGCGAGCCGTTCAGCTATCGTCTGCTGGAGACCGGTAAGTATCAGCTGATCGCCAGCAATATGACCGGGGATTATGCCAACCTCAGCTGCTGCGTGCTGGGGGTGAGCGGGAGCCTGGCGCGGGACCATAAACCGGCCGCCGCGGCGCTCACCCAGGCGATCCTTGAGGCGCACAGCTATGCCGCTGCGCATCCGGAAAGCGTGGCGCAATCCTTCCTCGCCCATGCCCTGAATACCAGTGAAGCGGAGGTGAGCGGCATCCTCCACGGGCAGGGCCACGGCCATCACGCGGTGGGCGAAGCGTTCGTGAAAGAGCTGACGCAGTATGCGGTCGACCTGCAGCGGGTGCAGGTGATCAAGCCGGGTACCGATCCCCATCAGTTCGCGGAGAGTATCTATGCCAACGTATTCGCCTGA

>IR1230_00045

ATGCGTCTATCATTTTCAGGCCGGTTGGCGGCCGCGCTGATGCTGCTGGCGCTGGGCGGCTACGCGACAGCGCAGGAGCGGCTTACCCTGCGTATCGCCGATCAAAAAGGCGGCATGCGTTCGCAGCTGGAGGCGGCCAACGCCCTGCAAAATCTCCCTTACGACATTAAGTGGGCCGAGTTCCCGGCCGCCGCGCCGCTGGCGGAAGCCCTCAACGCGGGCGCCGTGGACGCGGGGATCATCGGCGATGCGCCGTTGCTCTTCGCCCTGGCCAACGGTGCGCCGGTCAAAGCTATTGCCGTGGATAAAAGCAACCCGGCGGGGACCGCGGTGCTGGTATCCCCCGGCAGCACGTTAAAAAGCGGCGCTGATTTAAAAGGCAAACGCATCGCCACCGGTAAAGGGTCGATTGGTCATTTCGTGGCGCTGAAAGCGCTGGAGCAGGCGGGGATTTCACCGAAGGAGGTGCAGTGGGTGTTCCTCGGCCCGGTGGACGCCAAAGTGGCGTTGCTAAACGGTTCGGTGGACGCCTGGGCGACCTGGGAGCCCTACACCACCCAAATGGTGAAGACCAACGAGGGGCAGATCCTGGTGAGCGGCAAAGGGCTGCTGCCGGGGAATACCTTCCTCGCGGCAACGGATTCCGCGCTTAACGATCCGCATAAACGCGCCGCGCTGCAGGATTATCTCCAGCGCCTGGCCGGCGCCGAGCGCTGGGCCTACGCCAATCTCGACAGCTACGGCAAAACGTTGGGGGAGATCATCCGCTTCCCGGCGGAGATCGCCCGTGCGCAGTTTGCCAACCGCCAGTCGCAGTGGCAGCCGCTGGCCGAGGAAACGGTGGCCCAGCAGCAGGCAACGGCGGACTTTTATCTCGCCAACGGCCTGATCCGCACCCGGCTGGACGTGAAGCCGACCTTTGACCGCCGTTTTAGCGTGCCCGCCGCCGAGGTGACGCCATGA

>IR1230_00046

GTGATCCATCCTGAGCGCGACGACTGGGCCCGGCAGCTGACGGCGCTTCGCCAGCAGATGGCGGAGCAGGCAGCCAGCCTTGACGCCAGCGGCGAGTTTCCCTGGCGTAATATCGACCATCTGCGTGCGGGGGGCTGGTTAAGTCTCGCCGTGCCGCCATCCTGCGGCGGCGCGGGCGCCAGTCTGGCCCAGCTGCAGCAGGTCATCGCCGCCATCGCCTGGGGAGAGCCGGCGACAGCGCTGATTGTCTGCATGCAGTATCTCCACCATTTGCGGCTGGCGGAAAACGACGCCTGGCACGCGCCGCTGCGCCAGCAGGTTTTTCACGATGCTGTCGAACACGGTGGCCTCATCAACAGCCTGCGCGTCGAGCCGGAGCTGGGCTCCCCGGCGCGGGGCGGCCTGCCGGACACCGTGGCGACCCGTCGCGCGGAGGGCTGGGACATCAGCGGCCATAAAATTTATACCACCGGCATTGAAGGACTGCGCTGGCTGGCAGTGTGGGCCAGAAGCGATGATAACCCGCCGCTGGTGGGTACCTGGCTGGTGCCTGGCGACAGCCCGGGTATCACGGTGGTGAAGAGCTGGGATCACGCCGGGATGCGGGCCACCGGCAGCCATGAAGTTATTTTTAACCACGTGCGGGTGGCGGCAGAGCACGCGGTGGATGTCTGGCCGACCGATACGCCATCCGCCGCCCAGGCCGAACCGTTTCGCCTGTTCGCCAACCGGCAGACGGCGCTGCTGGCGGCGATCTATGACAGCATTGCCCACGCCGCTCACGACTGGCTGGTGAAGTGGCTGGCGGGGCGGGTGCCCGCCGGTCTCGGCCATCCGCTCTCTCGTCTGCCACGGGTGCAGGAGAAAGTCGGGCAGATCGCCGGGCTGCTGCTGGTCAGCCGCAGCCTGCTGGAGCAGGCCGCCGCGCTGCGCTTTTCCGCCATCGAGGCCAATCTGGCGAAAGTCACCATCACCGACAACGCCATTCAGGCGGTGAATATCGCGCTCGAGCTGACCGGCAATCATGGTCTCAGCCGACAAAACCCGCTGGAACGCCACTACCGCAATGTGCTCTGCGGACGGGTACATACCCCACAGAGCGACAGCGCCTGGCTGGCAGCTGGCAACTTCGTCTTTCAATCACAAGGATAA

>IR1230_00047

ATGAGCATTCAGTTTCTCGGCATGATTGGCCACCGCCTCTCTTCCGAAACGATTGCCCCGGTGGGGCCGATCTTTGACCGCGACTATATCGTTCGCTTTGCGCAAACCCATGAAGCCGCCGGGTTTGATCGTCTGCTGGTCGGCCACTGGTCCGATCAACCCGACGGCTTTCTGGTGACGGCCCTGGCCGGACTGTCGACGCAGAAAATTCAGTACCTGCTGGCCCATCGTCCCGGTTTTGTTTCCCCAACCCTTGCCGCGCGCAAATTCGCTACCCTTGAGCACCTGCTGGAGGGCCGCTTGGCGGTGCATATCATCAGCGGCGGCAACGATGCCGAGCAGCGCCGGGATGGCGATTATCTCGACCACGACCAGCGCTACGCCCGCACCGACGCCTTCCTCGACATCGTGCGCCAGGTGTGGACCAGCGAGCAGCCGGTGGATATTCACAACGATTTCTATCAGGCCGAGCAGGCCTGGTCCGCCATTCGCCCGCTGCAAAAACCGCATCTTCCGATCTATTTTGGCGGCTCGTCGGAAGCGGCCATCGCCGTCGCCGGCAAACATGCCGACGTCTTCGCCCTGTGGGGCGAGTCGCTGGCGCAAACCGGCGAGACCATCCAGCGGGTGCGCGCCGAAGCGGCGAAACACCAGCGTGATATCGGCTTCAGCGTCTCGTTCCGGCCGATTATCGCCGACAGCGAAGCCGAAGCCTGGGAGAAAGCCGAGCATATCCTGCACGTCGCCACCGAGCAGGCGGCGCAGCGCGGGGGCGGTTTCAAAGCCAAACCGGAGAGCATCGGCGCCCAGCGGCTGCGGGCTACTGCGGCGCAGGGCAGAGTGGTGGACAAACGCCTGTGGACCGGCATCGCCCAGCTGGTGGGCGGCGGACATAACTCCACCGCGCTGGTGGGCACCCCGGAGCAGGTGGCGGATGCGCTGCTCGATTACTACGACCTCGGGGTGCGTAACTTCCTGATCCGCGGCTTCGATCCGCTGAACGACGCGGCCGACTATGGCCGGGCGCTGTTGCCGATCGCCCGGGAAAAAGCCGCCCGGCGTGCAGTGGCGGAGCGCGCATCGTGA

>IR1230_00048

ATGCGCATTGACGATATCGACGCTTTGCTGGCCACGGTGCAGTTTTCCTCCCTCAATCAGGCCGCGGAATATCTGGGGATCACCCAGTCGGCCATCACCCGGCGCCTGCAGCGTCTGGAACAGGAGCTGAACGTCACGCTGTTAGAGCGCCAGACCCGCCCGCTGACTCTGACCGCCGCCGGACACCGGGTGTACGAGCAGTGTCTGAGCATTAAGCGTGAGACGAAAAAACTCTACAGCCTGTTGGATCCGGAAGGCGAGCCGCGCGGCGCGCTGCGTCTCGGCGTGCCGCAGAGTCTCAGCGAGATTGCCCTGCCGGCGGCGCTGTCGGCGCTGAGCCAGCAGTTTCCCGGACTGTCGCCGCAGATCACCTGCGGCTGGAGCGGACAGCTGCAGCGCTGGCTGGAAAACGGCGAGCTGGACGGCATGCTGGCGATGGGGCCCGCCCAGCAAAGCTTCGCCGAGGGCTACAGCGGGCGCCTGCTGTGTCCCCTGGAGGTGGTGCCGATCGTCGGCCGGCGTTTGAATTTGCGCGCGTCATCCCTGCGGGAGTGCGCCGAACGGGGCTGGATCCTTAACCCCGACGGCTGTGGCCTGCGCGCCGGGCTCATTCGCGAGCTGCAGAGCCAGGGATTGCGGTTGACGCTCAACGTCGAGAGCGCGGGCGCGCAGCTGCAGATAGCGCTGGTGGCGCAGGGGCTGGGGCTGGGTCTGGTGCCGAGGGCGGCGCTGGCCAGCAGCCCGTGGCGGGACGAGGTCGCGGTCCTCAGCCTCAGCGATTTTCAGCCTGCGGTCTCACTGTGGCTGATCCACGCCCAGTATCTGGCGAACCTGCAGCCGCCGCTGACCTTTTTTGCCAGCAAAGTGATGCAGCAGCTGACCGTATCCGACTGA

>IR1230_00049

ATGACTGCACCACGACTGGAAAAATTACGCCATTTCATTCATGAGGTAGACCGCCTGCACCGCGAACACCACCAGACCGCCCCGCTGCTTGACGCCGTGGCGCAGCGGCTGGCGGCGCTGGTGCGTTACGACGACTGGCTGCCGGAAGAGTACACCCTGCCGCATCCGCACCATTACCAGCAGTATCTGCTGCATGCCGACTCCGGGGAGCGCTTCTCGATTGTCAGCTTCGTCTGGGGACCGGGGCAAGCGACGCCGATCCATGACCACCGGGTGTGGGGGGCCATTGGGATGCTGCGCGGCGCGGAGGAAAACCAGCGCTACCAGCTGGATGCCGATGGCATACCGCGCGCGCGCGGCGCCGCCACGCTTCTGGAAGCAGGCCAGGTGGAGAAAGTATCCGCTGACGATGGCGATATTCATCGCGTCAGCAATGCGCTGGCGGATCGCGTCTCTATCAGCATTCATGTCTACGGCGGCAATATCGGCGCGGTGAAGCGCGCCGTGTATACCCCGGAAGGGCAGCAGAAACCGTTTATCTCCGGCTACTCTAACCGCCATTTACCCAATATCTGGGATTCCTCCCGCGAACATCAAGGATAA

>IR1230_00050

ATGTCCACCTACGCTTATCGCCAGGCCGCCGGGATCCGCCAGGCGCTGCTCGACCGTCGTGAACTGGCGCTGATCGACGTCCGTGAAGAGGCCGATTTCGCCACCGCTCATCCGCTGTTTGCCGTCAATCTGCCGCTCAGCAAACTGGAGCTCGAGGTCCGCCGGCGGATACCGCGCTTCACCACCCCGCTCACCGTCTACGACAACGGCGAGGGTCTGGCGGAGATCGCCGTCGAACGGCTGCGGTCCTGGGGCTATCAGGATGTGGCCCTGCTGGCCGAGGGTCTCGCCGGCTGGCGCCGCAGCGGCGGCGAGCTGTTTCAGGACGTCAACTCCGCCAGTAAAGCCTTTGGCGAGCTGGTGGAAAGCGTGCGTCACACACCGTCGCTCAGCGCCCAGGAGGTTCAGGCGCTCATCGACAGCCGGCAGGAGGTGGTGATCGTCGACGCCCGGCGCTTCGATGAGTACCAGACCATGAGCATCCCCGGCAGCATCAGCGTGCCCGGCGGCGAGCTGGCCCTGCGGGTAGAGAGCCTGACGCCCTCCCCGCAGACGCCGGTTATCGTCAACTGCGCCGGACGCACCCGCAGCATCATTGGCACCCAGTCGCTGATCAACGCCGGCGTGCCAAACCCGGTCCATGCCCTGCGCAATGGCACTATCGGCTGGACGCTGGCCGGCCAGACGCTGGCGCACCAGCAACAGCGGCAATACGATCCCTCCGCCCGCGCCTCCGGCGCCCGGGCCGCTGAGGTGGCGCATTTTGCCGAACGGGCCGGGGTGGCGGTCATCGATGAGGCCACGCTGCAACGCTGGCAGCAGCAGAGCGACCGCACCACCTTCCTGTTTGATGTCCGCAGCCCCGAAGAGTACGCGGCGGGTCACTATCCCGGCAGCCTGAGCGCGCCAGGCGGCCAGCTGGTGCAGGAGACCGACCACTTCGCCAGCGTACGAGGGGCGCGCATTGTGCTGCTGGATGACGATGGCATCCGGGCGGCGATAACCGGCTCGTGGCTGGCGCAGATGGGCTGGGAGACGGCGCGGCTGAGCGCGCTGTCGACCAGCCAGTTGAGCGAACGCGGGGTGCCTGCCGCCGAGGTGCCGCCCGGCCCGCAGGCGGAGGAGATAAGCCCGACGCAGCTGGCGCAACAGCTGGAAGAACCCGGTACGGTGGTGCTCGATTTTACCACCAGCGCCAACTTTGTCGCTCGCCATATCCCCGGCGCGTGGTGGCTAACGCGCTCGCAGCTGCGCCAGGCCCTGGAAGCAATCCCTCCCGCCCAACGTTACGTGGTGACCTGCGGCAGCAGTCTGCTGGCCCGCTACGCGGTGCCGGAGGTGGCGGCCTTGACCGGTAAACCGGTGCAGTTGCTTACCGGCGGGACGCTGGCGTGGATCGCCGCCGGTCTGCCGCTGGCGCATGGCGACAGCGGCCTCGCCGTCGAGCGCCGTGACCGCTATCGTCGTCCGTACGAAGGAACCGATAATTCCGCCGAGGCCATGCAGGCCTATCTGGAGTGGGAGTATGGCCTCGTGGATCAGCTGGCCCGCGACGGCACCCACGGTTTTCGGGTGCTCTAG

>IR1230_00051

ATGGCTTACACCTCCCGTTTGCTAAATGCGATCCCTGGCATCCCCCACGCTTTTCTCGATGTCCATGAAACCGCCGCCTTTCCCTATGCCGAGCTGGCTCCGGTGAAGCTGGTGCATGGTAATGAGGTTCATCATTATCAGCAGCCGCTGCCGACGCGCCCGCACGCCGATGCGGTGTTTACCGCGGTAGCCGGGCAAAAGGTGGGGGTGGTGACCGCCGACTGTCTGCCGCTGCTGATCGCCTCGCGCGACGGTCGCTACGTCTGCAGCGTGCATGCCGGCTGGCAGGGGCTGGTCAGCGGTATTGTCGACAACAGTCTGGCCTGTTTTCGCCAGCAGGGGGTGGCATTGGCCGATCTGGTCATCGCCGTGGGGCCGTATATTCACCCCTGCTGTTACGAAGTCTCCGCCGGATTCTATCAGCAACTGCTGGATCAGCCCGGCGGTGACCGGGTGGCCCGCCATCGACAGCGGCTGTTCCACTCCCGTTCGGGCCCGGTCAGCGATCCGCTCAAAGCCGCCGCGCGTGGCAGCGATAATCTGTGGTTTGATCTGCGCGCCTTTGCCGAAGCGATCTTCGCAGAGGCGGGGGTGGCGCCGGCGAGCGTTGAATGGCTGGGGAGTTGTACCTACTGCACCCCGCAGTCGCTGGGCTCCTACCGGCGCCGGACCCATTTTCCGGCGCCGAAGAGCTTTCAGTATTCGTGGATCATGCGCGAAGCCTGA

>IR1230_00052

ATGACCAAGATAACGACTGCAAACCCCTCGCGCCTGGTGGTCACCATCGGGCTCTGTTTTATGGTGGCCCTGATGGAAGGGCTGGATCTGCAGGCGGCGGGGATCGCCGCCGTCGGCATGGCGCAGGCCTTTGCGCTGGATAAAATGCAGATGGGCTGGATCTTCAGCGCCGGGATCCTCGGCCTGCTGCCCGGCGCGCTGGTGGGCAGCATGCTGGCGGACCGCCACGGGCGTAAGCGCATTCTGTTGGGGTCGGTGCTGCTGTTTGGCCTGTTCTCGCTGGTCACCGCGCTGGCATGGAGCTTCCCGACGCTGCTGCTGGCGCGCCTGCTCACCGGCGTCGGGCTCGGGGCGGCATTGCCGAACCTGATTGCCTTAACCTCGGAAGCCGCTGGTTCACGCTTCCGCGGCCGGGCGGTAAGCCTGATGTACTGCGGCGTACCCATTGGCGCCGCGCTGGCGGCAGCGCTGGGCTTTTCCGGTCTCGCCGCGGCCTGGCAGACTATTTTCTGGATTGGCGGCGTGGTCCCGCTGCTGCTGATCCCGTTGCTGATGCGCTGGCTGCCGGAGTCGCAGGCGTTTCAGCGCGCGGAGGCCAGCGTGCCGCTGCGCACCCTGTTTGCCCCGGGCCAGGCCGCCGCTACGCTGCTGCTGTGGCTGGGTTATTTTTTCACCCTGCTGGTGGTCTATATGCTGATCAACTGGCTGCCGATGCTGCTGGTGGGCCAGGGATTCCGCGCCAGCCAGGCGGCGGGGGTGATGTTCTCTCTGCAGACCGGCGCCGCCTGCGGCACGCTGCTGCTGGGGGCGCTAATGGATAAGCTCACCCCGCTGCGGATGTCGCTCCTGATCTACAGCGGCATTCTGGCGTCGCTGCTGGCGCTGGGCAGCGCGTCGTCGCTGACTGGCATGCTGCTGGCGGGGTTTGTCGCCGGGCTGTTCGCCACCGGCGGACAAAGTGTGCTTTACGCTCTGGCGCCGCTTTTTTATCCCGCGGCCATCCGCGCCACCGGGGTGGGCACCGCCGTGGCGGTGGGGCGTCTTGGGGCGATGAGCGGCCCGCTGCTGGCCGGCAAGATGCTGGCCCTCGGCGCCGGCACCGTCGGGGTGATGGCGGCCTCGGCGCCGGGCATTGTGTTGGCCGGCGTGGCGGTGTTCTGGCTGATGCATCGCCAGCAGCGCGCGGCCATGGTCTGA

>IR1230_00053

ATGAACGGTAAAAAACTTTATATCTCGGACGTGACGCTGCGCGATGGTATGCACGCCATTCGTCACCAGTATTCCCTGGCGCAGGTGCAGCAGATCGCCAGCGCGCTGGATAAGGCTGGGGTCGACTCCATCGAAGTGGCCCATGGCGATGGCCTGCAGGGATCCAGCTTCAACTATGGCTTTGGCGCCCACAGCGACATCGCCTGGATTGAAGCGACAGCGGATGTCGTGAGCCAGGCGAAAATCGCCACCTTGCTGCTGCCGGGCATCGGCACGCTGCACGATCTGAAAGCAGCCTATCAGGCCGGGGCGCGGGTGGTGCGGGTCGCCACCCACTGCAGCGAAGCGGACGTCGCCGCGCAGCATATTGCCTTTGCCCGCGAGCTGGGGATGGACACCGTCGGTTTTCTGATGATGAGCCATATGATCTCCCCGCAGGCGCTGGCGCAGCAGGCGCTGAAGATGGAATCTTATGGCGCGACCTGTATCTATGTGGTGGATTCCGGCGGGGCGATGAACATGAACGACATTCGCGACCGCTTCCGCGCCCTGAAGGCGGTGCTGAAGCCGGAGACCGCCACCGGGATGCATGCCCATCATAATCTGAGCCTCGGGGTGGCGAACTCGATCGTCGCGGTGGAAGAGGGGTGCGATCGCATTGACGCCAGCCTGGCCGGGATGGGGGCGGGGGCGGGCAATGCGCCGCTGGAGGTGTTTATTGCCGCCGCCGACAAGCTCGGCTGGCAGCATGGCACCGATCTCTACGCCCTGATGAACGCGGCCGATGAGCTGGTGCGGCCGCTGCAGGATCGCCCGGTGCGTGTCGACCGCGAAACGCTGGCGCTGGGCTACGCCGGAGTCTACTCCAGCTTCCTGCGCCACAGCGAAGCGGCGGCCAAGCGCTATGGCCTCAGCGCGGTGGATATTCTCGTCGAGCTGGGCAAGCGGCGGATGGTCGGCGGCCAGGAGGATATGATCGTCGACGTCGCGCTGGATCTGCTTAACCGCAATAAATAA

>IR1230_00054

ATGCGTAAACGTAAAGTCGCCATTATCGGCTCCGGGAATATCGGCACCGATTTAATGATTAAAATCCTGCGCCACGGCCAGCATCTGGAGATGGCGGTGATGGTCGGCATCGACCCGCAGTCGGATGGCCTGGCCCGCGCCAGGCGCCTGGGCGTTGCGACCACTCACGAAGGGGTGGGCGGCCTGATGCAGATGGCGGAATTTGCCGACATCGATTTTGTTTTTGACGCCACCAGCGCCGGGGCGCACATCAAAAACGATGCCGCGCTGCGCGAAGCGAAGCCGGGGATCCGCGTTATCGACCTGACGCCGGCGGCGATTGGCCCATACTGCGTACCGGTGGTCAACCTCGCGGCCAATCTCCATCAGGGCAACGTCAATATGGTGACCTGCGGCGGGCAGGCGACCATCCCAATGGTCGCCGCGGTCTCGCGAGTGGCGAAGGTGCATTACGCGGAGATCGTGGCCTCGATCGCCAGCCAATCCGCCGGGCCGGGCACGCGGGCCAATATTGACGAATTTACCGAAACCACCTCGCAGGCGATTGAAAAAGTCGGCGGCGCGGGCAAGGGCAAAGCGATTATTGTACTGAACCCGGCGGAGCCGCCGCTGATGATGCGCGATACCGTCTACGTCCTGAGCGAGCTGGCGTCGCAGGAGGCGATTGCCGCCTCGATCGCCGAAATGGCGGCGGCAGTGCAGGCTTACGTCCCGGGCTACCGGCTGAAGCAGCAGGTGCAGTTTGAGGTTATCCCCGAGGACAGGCCGGTCAACCTGCCCGGCGTCGGCTGTTTTTCCGGCCTGAAAACCGCGGTTTACCTCGAAGTGGAAGGGGCGGCGCACTATCTGCCAGCGTACGCGGGCAACCTCGATATTATGACCTCGGCGGCGCTGGCGACGGCGGAGCAAATGGCCGGGGCAATGCACAGCGCGGCGGGAGCGACAGCATGA

>IR1230_00055

ATGACTTTTTCTCTTGACGCGCTGGCCCGGCAGCTCCGCGACGCGGAACAGAGCGGCCAGGCGATCGCCCCGCTGCGCGATATTCTCGGCGTCGACAATGCCGACGCCGCCTATGCCATCCAGCGCCTGAACGTCCAGCACCATGTGGCCCATGGCCGGCGGGTGGTGGGCCGCAAGGTGGGCCTGACCCATCCCAAAGTGCAGCAGCAGCTGGGCGTCAATCAGCCGGACTTCGGCACCCTGTTTGCCGACATGTGCTACGGCGACAACGCCGAGGTGCCGTTTGGCCGCGTGCTGCAGCCCAAGGTGGAGGCCGAGATCGCCCTGGTGCTGAAGCAGGATCTGCCGCATGCCGATACCACTTTTGATGAACTGTATAACGCCATCGAATGGGTATTGCCGGCGCTGGAGGTGGTGGGCAGCCGCATTCGCGACTGGTCAATCGGCTTTGTCGACACCGTCGCCGATAACGCCTCCTGCGGCCTGTACGTCATTGGCGGCCCGGCGCAGCGTCCGGCGGGACTGGATCTGAAGCAGTGCGCCATGCACATGACCCGCAACCAGGAGCTGGTCTCCAGCGGCCGCGGCAGCGAGTGTCTGGGGCATCCGCTGAACGCCGCAGTCTGGCTGGCGCGCAAGCTGGCGAGTCTCGGCGAACCGCTGCGGGCGGGGGATATCGTGCTGACCGGGGCGTTAGGCCCGATGGTGACGATTAACGAGGGGGACAGCTTTGTCGCCCATATCGAAGGTATTGGCTCAGTCGCGGCACGCTTCGTTGCCGCCGGAGAAGGAGATCGTGATGCGTAA

>IR1230_00056

ATGAGCTATCAACCGCAAACTGAAGCCGCCACCAGCCGATTCCTCAATGTCGACGAGGGGGGCCGCACGCTGCGCATCCATATCAATGACTGCGGCGACGGGAAAGAGACCGTGGTCATGCTGCACGGCTCCGGGCCGGGGGCCACCGGCTGGGCCAACTTCAGCCGCAATATTGACCCGCTGGTCGAGGCGGGCTATCGCGTGCTGCTCCTCGACTGTCCGGGGTGGGGCAAAAGCGATGCTATCGTCAACAGCGGCTCGCGTTCGGACCTCAACGCCCGCATTCTGAAAAGCGTGGTCGATCAGCTGGGGATCGATAAAGTCCATCTGCTGGGCAACTCGATGGGCGGCCATAGCGCGGTGGCCTTCACCCTGAGCTGGCCGGAGCGGGTGGCAAAGCTGGTGCTGATGGGCGGGGGGACCGGCGGCATGAGCCTGTTCACGCCAATGCCGACCGAGGGCATCAAGCTGCTGAACGCCCTGTACCGCGAGCCGACCATTGAAAACCTCAAAAAGATGATGAGCATCTTCGTCTTCGACACCCGCGACCTCACCGAGGCGCTGTTCGAGGCGCGTCTCAACAATATGCTGTCGCGCCGCGACCATCTGGACAACTTCGTCAAGAGCCTGGAAGCCAACCCGAAGCAGTTTCCTGACTTTGGCCCGCGGCTGGGCGAAATCAGCGCGCCAACTCTGATCGTCTGGGGACGCAATGACCGCTTCGTGCCGATGGACGCCGGGTTGCGCCTGCTCGCCGGGATCGCCGGTTCGGAACTGCATATTTACCGCGACTGCGGCCACTGGGCGCAGTGGGAACATGCCGACAGTTTTAATCAGCTGGTGCTCAATTTCCTCGCACGGGCTTAA

>IR1230_00057

ATGCATGCCTATTTGCACTGTCTCTCCCACACTCCGCTGGTGGGATTCGTGGACCCGGAGCAGGCGGTCCTCGATGAAGTGAACGGGGTGATTGCCGACGCCCGGCGGCGGATCGCTGAATTCAATCCAGAGCTGGTGGTGCTCTTCGCCCCCGATCACTACAACGGTTTTTTCTATGACGTGATGCCGCCGTTCTGCCTCGGGGTCGGCGCCACCGCGATTGGCGACTTCGCCAGCGCCGCCGGCGACCTGCCGGTGCCGACCGAGCTGGCGGAAGCCTGCGCCCACGCCGTGATCAACAGCGGTATTGACCTGGCGGTGTCGTACAACATGCAGGTGGACCACGGCTTCGCCCAGCCGCTGGAGTTTCTGCTGGGTGGCCTGGACCGCGTACCGGTGCTGCCGGTGTTTATTAACGGCGTCGCCGCGCCGCTGCCGGGTTTTCAACGCACCCGGCTGCTGGGCGAGGCGATGGGCCGCTTTCTCAATACCCTCAATAAGCGGGTGCTGATCCTCGGCTCGGGCGGACTCTCGCACCAGCCGCCGGTGCCGGAGCTGGCGAAGGCCGACGCCCATCTGCGCGATCGCCTGCTCGGCGGCGGAAAGCAGCTGCCGCCGGACGAACGCGAACGGCGCCAGCAGCGGGTGATCAATGCCGCCCGACGGTTTACCGAAGACCCCCACTCGCTCCATCCCCTCAACCCGGTCTGGGACAACCGCTTTATGAGCCTGCTGGAGCAGGGGCGGCTGAGCGAACTGGACGCGATCGGCAATGACGAGCTGTCGGCGATGGCCGGGAAATCGACGCACGAAATTAAGACCTGGGTGGCCGCTTTTGCCGCGCTATCCGCTTTTGGCCGCTGGCGCAGTGAAGGCCGCTATTACCGGCCGATCCCCGAGTGGATCGCCGGATTCGGCTCGCTGAGCGCCACCACCGAGATTTGA

>IR1230_00058

ATGACAACATCTACACCCGATATTCAACCCGCCGTGCAGCATACCGCTCAGGTCGCCATCGTTGGCGCCGGTCCGGTTGGTCTGATGATGGCCAACTACCTTGGCCAGATGGGCATCAGCGTGCTGGTGGTGGAAAAGCTCGCCACACTGATCGATTACCCGCGGGCCATCGGCATTGACGATGAATCCCTGCGGGCGATGCAGGCGGTGGGGCTGGTGAATGACGTGCTGCCGCATACCACGCCGTGGCACGCCATGCGTTTTCTCACCCCAAAAGGCCGCTGCTTTGCCGATATCCAGCCGATGACCGATGAGTTTGGCTGGTCGCGGCGCAACGCCTTCATTCAGCCGCAGGTCGATGCGGTGATGTATCACGGTCTGCAGCGTTTCCCGCAGGTCCGCTGCCTCTTCTCCCGCGAAGTGGAAGCCTTTAGCCAGACCGGTGACGGTGTCACGTTAAATCTTAAAGGACCGGACGGCGAGCGGGAGACGGTGCGCGCGGACTGGCTGGTGGCCTGCGACGGCGGCGCCAGCTTTATTCGCCGTACGCTGAACATCCCCTTCGAAGGTAAAACCGCGCCGAATCAGTGGATCGTCATCGATATCGCTAACGACCCGCTGGCCACCCCGCACGTTTACCTGTGCTGCGACCCGGTGCGACCCTACGTTTCCGCCGCGCTGCCCCACGGCGTGCGCCGCTTTGAATTTATGGTGATGCCCGGCGAAACCGAAGCTCAGCTCAGCGAACCGCACAATATGCGCCGCCTGCTGAGCAAAGTGCTGCCGGACCCGGATCGCGTCGAGCTGATCCGCCAGCGGGTCTATACGCATAACGCCCGCCTTGCCGAGCGCTTTCGCATCGACCGCGTGCTGCTGGCCGGCGATGCGGCACACATCATGCCGGTCTGGCAGGGCCAGGGGTACAACAGCGGCATGCGCGACGCCTTCAACCTGGCGTGGAAGCTGGCGCTGGTGGTCAACGGTAAAGCGGGAGAGGCGCTGCTCGACAGCTACCAGCAGGAGCGCCGCGACCACGCGAAAGCGATGATTGATCTGTCGGTCACCGCCGGCTACGTGCTGGCGCCGCCGAAGCGCTGGCAGGGGGCCGTGCGCGACGGTCTCTCCTGGCTGCTGAATTACCTGCCGCCGGTGAAGCGCTACTTCCTCGAGATGCGCTTTAAGCCGATGCCGCAGTACCGCGAGGGGGCGCTGCTGACCGACGGCGCCGGCAAGACCTCGCCGGTGGGCAAAATGTTTATTCAACCGCAGGTGACGCTGGAGAGCGGCGAGAGCGTGCTGCTCGACGACGTCATCGGCGCGAATTTCGCCATTATCGGCTGGGGCTGCAACCCGCAGTGGGGGCTCAACGCCGGGCAGATCGCCCGCTGGCGCGCGATTGGCGTGCGCTTTATCCAGGTGGTGCCGGAGGTGCAGATCCACCGCGAGCAGGATAACGCCCCCGGCACGCTGCGGGTGGGCGACAGGCAAAATCGCCTGAAGAGCTGGTTTGCTCAGCATAACACCGCCATCGCCGTGGTCAGACCGGACCGCTTTGTCGCCGCTCTCGCCATCCCGCAGACCCTCGGCGCGCAGCTGACCGCCCTGGCGGAAAAAATGACCCTCGCAATCGAGGAAACTGCCCACGCAGAAGAGAAGGTGGCCTGA

>IR1230_00059

ATGCGTGACGATGAGTCCGCAGCGTACAAAACGGTGCGCGGATTAAGCCGGGGTTTATTGTTATTAAAATTGTTAAATAAATTCGACGGCGGCGCGACGCCGGGCCTGCTGGCGGAGTTTAGCGGCCTGCATCGCACCACCGTGCGCCGGCTGCTGGAGACGCTACAGGAGGAGGGTTTTGTCCGCCGCAGCCGCTCCGATGACAGCTTCCGGTTGACCATTAACGTCCGCCAACTGAGCGATGGATTTCGTGACGAACATTGGATTTCTGCCCTCGCCACGCCGCTGCTCGGCGAGCTGCTGCGCGAGGTGCAGTGGCCAACGGACATCACCACCCTCGACGTCGACGCCATGGTGGTGCGCGAGACTACCCACCGCTTCAGCCGACTGTCGTTTCACCGCGCGATGGTCGGACGGCGGCTGCCGCTGCTGCTCACCGCCTCCGGACTGACGTGGCTGGCCTTTGCCCCCGACGCGGAACGCGACGCCATCGTCAGCATGCTGGCCGCCCGTCCGGAAGCTGAGTATCAGTTGGCCCGCGAACCGGAACGGCTGGCGGCGATCCTCGCCCGCACCCGCCAGAACGGCTATGGCGAAAACTTCCGCGGCTGGCGGCAGGAGGAGAAAATCGCCTCGATCGCCGTGCCGGTCTGCAGTCAGCAGCGGGTTATCGGCTGCCTGAACCTGGTCTATATCGCCAGCGCAATGACCATTGAGCAGGCGGCGCAGAAGCATCTCCCTGCACTGCAGAGGGTGGCGAGGCAGATAGAAGCCCGCATGGAAGAGGAAGAAGTGTGGTACGAAATGCCCTGA

>IR1230_00060

ATGTCAAAACACCCCTTACTGCTGGGCCTGACGCTGCTCAGCGCCAGCCTGTTCACCGGCCAGACCTTCGCCGACCGCACCGTCACCGACCAGCTGGGTCGCCAGGTCACCCTCCCCGACCACGTCACCCGCGTTGTGGTTCTGCAGCATCAGACGCTCAACCTGCTGGTACAACTGCATGCCGCCGAGGATATCGTCGGCGTCTTAAGCAGCTGGAAGAAACAGCTCGGGCCGCAGTTCGCCCGCTTTATGCCCGAGATCGGGCAACTGGCGACCCCGGGCGATCTCACCCAGGTCAATATTGAAAGCCTGCTGGCGCTGCGTCCGCAGGTGGTTTTCGTCGCCAACTATGCGCCGCCGGCGATGATCGCGCAGATCCAACAGGCCGGGATCCCGGTGGTGGCCATCTCGCTGCGCCACGATGCCGCCGGGGAAAAAAATAAAATGAACCCCACCATGGCTGACGAAGAGCAGGCCTATAACGCCGGGCTGATGGAGGGGATCCTCCTTATCGGCGAGGTGGTCGAGCGCCAGCCCGAGGCCGAGGCGCTGATTCACTACACCTTCGAGGCGCGCAAGCAAGCCAATGCGCCGGTGGCCGATATTCCCCAGAACCAGCGGGTGCGGGTGTACATGGCCAACCCCGATCTGAATACCTATGGCGCTGGCAAATACACCGGGCTGATGATGGCGCACGCCGGGGCGCTGAACGTGGCGGCCGCCAGCGTAAAAGGCGCCCGCCAGGTGTCGCTGGAGCAGGTGCTGGAGTGGAACCCCCAGGTGATCTTCGTCCAGGACCGTTATCCGCAGGTGGTCAAACAGATAGAGAACGACCCGCAGTGGCAGACGATCGATGCGGTGAAACATCACCGCGTGTGGCTGATGCCGGAGTATGCCAAAGCCTGGGGTTACCCGATGCCGGAGGCCCTGGCGCTGGGTGAGCTGTGGATGGCGAAAAAACTCTATCCTGCGCGCTACCAGAGCATCGATGTGGACAGTAAAGCCAGGGATTATTATCAGCGTTTTTACCGCGTGGCGTGGACGCCAGATGCCCGCTAA

>IR1230_00061

ATGCCCGCTAACTCTCAGCCCTGGCTGGTGCAGGGAAGCCTGGCGCTGGCGACGCTGGGGATCGCGGTCGCCTCGCTCTGTTTCGGCCAGTACCCGTTAAGCCTGAGCGCCGTTGGCCACACCCTGGTCCATCTGCCGCCAGGTGAAGGGGTGATCGGGCAGATCGTCTGGTCGGTTCGCCTGCCACGCGTGGTGATGGCCCTGCTGGCCGGGGGCGCGCTGGGGCTGTGCGGCGCCACCTTGCAGGGCGTGTTCCAGAACCCGCTGGTGGACCCGCACATCATCGGGGTGACCGCCGGCTCCGCCTTTGGCGGCACGCTGGCGATCCTGTTGGGCGTCGGCAGTCTGCTGATGATGGCCTCGACCTTTTTCTTCGGCCTCGTCGCGCTGGGGCTGATTTACGCCCTCGCCGCCCTGCAGGGGCGGGACAGCACGCTGGGGCTGATTCTGTCCGGGATCATTCTCAGCGGCTTCTTCGCCGCGCTGGTCAGCCTGATGCAGTACCTCGCCGACAGCGAAGAGACGTTACCGAACATCGTTTTCTGGCTGCTCGGCAGCTTCGCGACCGCCAGCTGGCATAAGGTACTGCTTATGTCCCTGCCCCTGGCGGTGGCTGCCGGGGCGCTGTGGAAGCTGCGCAGGCGTATCAACCTGCTGGCCCTGGAGGAACGCGATGCCCGCAGTCTTGGCATTCCGGTGGCCGCGCTGCGTCGCGGCGTGCTGGTTTGCTGCGCGGTGCTGGTGGCAGCCCAGGTGGCGGTCAGCGGCAGCATCGCCTGGATGGGTCTGGTGGTCCCGCACCTCGCCCGTCTGCTGGTAGGGGCTGACCATCGTCGCCTGCTGCCCACCGCCTTCTGGCTCGGCGCCGCGCTGATGCTGGTGGTGGACGATCTGGCGCGTACGCTGACCCAGGCGGAGATCCCGATCGGGATCATCACCGCCCTGCTCGGGGCGCCGCTGTTTACCGTGTTGCTGGTGCAATCCCGACGCCGGAGTACGACCCGATGA

>IR1230_00062

ATGAACAGCTCATTATCGCTGCAAGCGCTACGCTACGGCCACCGTCAGCCGCTCTTCGCCCCGCTCACGCTGGCCTGCCGGCCCGGTGAGATCTGGGCGGTGCTGGGCGCCAATGGCCGGGGAAAAAGTACCCTGCTGGATACCCTGACCGGCGTGCTGCCGCCGCTGGGCGGCGAGATGCAGTGCGAGGGCGGCGTGGCCCTGGTTCCGCAGTCGTTTCGTCCCGCTTTTCGCTGGCGCGTGAGCGATGTGGTGCTGATGGGCCGGGCCCGTCACGTGGATCTCTTTGCCCAGCCGGACGAGGAGAATGCCCGGCGGGTTGAGCAGGCGCTGGCGCAGCTGGGGATTGCCGCCCTGGCCGAAGATGACTTCGGCGCCCTCTCCGGCGGCCAGCAGCAGCTGGTGTTGATTGCCCGCGCGCTGGTCAGCGCCAGCCAGAATATCCTGCTGGATGAGCCCTGCTCGGCGCTGGACCTCGGTAACCAGCAGGTGGTATTGCAGCTGATCGGCGACCTCGCCCACCGCCAGGCGCGCACCGTCCTGTTCACCACCCATGATCCCAACCACGCCCTACAGGTGGCCAGCCATACGCTGCTTCTGCTGCCGGAGGGCCGGTGGCTGGCGGGCGAGACCGCCGACGTGCTGAGCGAAACGCATCTCCGGCAAGCGTACGGCCTGCCGGTACGCCTGATCCGCCATGCCGCCTCCGCGTTCCCTCTGCTGGCGCCGGGGTTTACGCTGCGTCGCTGA

>IR1230_00063

ATGGCAAATACCACTCTCACGCTGCTGGCGGCGGGAAGCCTGAGAAGCGCCTTCCTGCCGCTGGTGGCGCACTTTCGGCAGCACACTGGCCTGGCGGTCGATGCGCAGTTTGGCCCTGCCGGCCTGCTGCGCGAGCGGATCGAAGCCGGGTCGCCGTGCGCGGTTTTTGCTTCCGCCAACGCGGCGCACCCGCAGGCGCTGCTGCAGGCGGGTCTGGCGCAGGAATGCCAGGGATTTGCCAGCAACCAGCTGATGCTGACCGCCCGGCGGTCGCCGGACAACGATGGCCTGGACTGGCTGGCGCTGCTCAGCACGCCCCGCCTGCGGTTGGCGACCTCCACGCCGGGCTGCGATCCGTCGGGAGATTATACCTGGCAGCTGTTCGCCCGAATTGAGGCACGCTATCCAGGGTTGGGCAACGCGATGGCCGGGCGGGCGCAGCAGCTGGTCGGCGGCAGAGACTCACTGAGCGTGCCGCCGGGAGAGATAGCCGGCGCGTGGCTTATTCGCCAGAATCTGGCGGATCTGTTTATCGGCTATGCGCACTATGGCCCCGCGCTGGCCACCTGCGACGATCTGCGTACCCTGACCATCCCGGCGCCGTGGAATATTCGCTGCGATTACCAGCTGGCGCGTCTCAGGGCCGATCCCGCCGCGCTGGCCCTGTATCGCTTTATCCTCGACGACGTTGGCCAGGGGTATCTCCGGCAGGCGGGTTTTATGCCGTTCAGCGACGCAGCGTAA

>IR1230_00064

ATGGTGATTAGTGGGAATACGCGGCTGATAGCGCACCTTGGCTATCCGACCGCCAGTTTTAAGGCGCCGATGATCTACAACCCGTGGTTCGTTGACCAGCAGGTGGACGTGAAGGTGGTGCCGATGGGGGTCAGGCCGGAGGACTACGCCGCGTTTATTCCGCCGCTGTTTACGATGACCAACATCATCGGCGCGCTGGTGACGATGCCGCATAAAATCGCCACCTGCGATCTGGTGCAGCGCCTGAGCCCGACGGCGGCCATCGCTGGCGCCTGCAATGCCATCCGCCGCGAGGCTGACGGTAGCCTGAGCGGAGACATGTTCGATGGCGAGGGATTTGTGCTGGGCCTGCGTCGTAAAGGGTTTAACCCGCAGGGGACGCGGGCGCTGGTGGTTGGCAGCGGCGGGGTGGGATCGGCTATCGCCGCCTCCCTCGCGGCGGCGGGGGTCAGCGCCCTGACCCTGTACGATACCCGGCCCGGGGTGGCGCACGCCCTCGCCGGGCGGCTGCATCAGCACTATCCGCAGCTCGATATCACCCTGATGCAGCCCGATCCTGCCGGACACGAGCTGGTGGTCAATGCCACCCCGCTGGGGATGAAGGCCAGCGATCCGCTGCCGCTGGACGTTGACCGTCTGGCGCCAGGCGCCTGGGTGGGGGAAGTCGTCATGACTCAGGAGTACACCCCGCTGCTGAGGGCCGCTCAGGCACGACAGTGCCACATCCAGCGCGGGACCGACATGCTGTTTGAAATGATCCCTGCCTATCTGCGCTTTTTTGACCTACCGGTAGCCACCCCCGAGCAGCTGCGGACCCTGGCAGAAATTCGCTACTGA

>IR1230_00065

ATGAGCTCATATAGCCCTAACCCGACGGCTGCCGCCCAGCCAGCCGTTACCGTGCGCCGTTCCCGGCGGCGTATCGGCATTCTTGCGCTGTTGGCCATTGGCACCATGATCAACTATCTCGATCGCACCGTACTGGGGATCGCCGCCCCAAAGCTCACCGCTGAGCTGGGGATCGACCCGGCGATCATGGGAATCCTCTTCTCCGCCTTCGCCTGGACCTATGCTCTGGCGCAGATACCCGGCGGATTATTTCTCGACCGCTTTGGCAACAAAGTGACCTACTTTTTGTCACTGACCCTGTGGTCGCTGTTTACCCTGTTCCACGGCATGGCGGTGGGACTGAAGACGCTGCTGCTGTGCCGCTTCGGGCTCGGCATTAGCGAAGCGCCGTGCTTTCCGGTCAACAGCCGGGTGGTCAGCGCCTGGTTCCCACAGCAGGAGCGGGCGAAGGCCACGGCGGTGTACACCGTGGGGGAGTATCTCGGGCTGGCCTGCTTTGCGCCGCTGCTGTTCTGGATCATGGACGGTTTCGGCTGGCGGGTGCTGTTCGTCAGCGTTGGCGCCGTCGGCATTCTGTTTGCCCTGGTGTGGTGGCGCTGCTACCGCGAACCGCATGAAGATCCGCGCCTTAGCCAGCAGGAGCGCGAGCATATCGAAAACGGCGGCGGCCTCAGCGCCCCGACCGACCAGCAGGTGGCGTTCAGCTGGCCGCTGGTCCGCCAGCTGCTGAGCAAACGGCAGATCATCGGCGCCAGTATCGGCCAGTTTGCCGGCAATACCGTGCTGGTGTTCTTTCTCACCTGGTTCCCCACCTGGCTGGCCACCGAGCGGCATATGCCCTGGCTGAAGGTTGGCTTCTTCTCCATCCTGCCGTTTGTCGCCGCCGCCGGCGGGGTGATGTTTGGCGGCTGGCTCTCCGACAAGTTGCTGAAAGCTACCGGGTCAGCCAACCTCGGTCGTAAGCTGCCGATCGTTGCCGGCCTGCTGATGGCCAGCTGCATCATTACCGCCAACTGGCTGGAGAGCGACCTGGCGGTGATCCTGGTGATGTCGTTTGCCTTTTTTGGCCAGGGGATGGTGGGTCTCGGCTGGACGCTGATCTCCGATATCGCCCCGAAGGGGCTGGGCGGCCTGACCGGCGGCCTGTTTAACTTCTGCGCCAACCTCGCGGGGATCCTCACCCCGCTGGTGATCGGCTTTATCGTTGCCGGGTTCGGCAATTTCTTCTACGCCTTGATCTACATCGGCGGCGCCGCGCTGCTGGGCGTGGTGGCCTATCTGTTTATCCTCGGCGACGTCAAACGTATCGAGTTATCGCAGTAA

>IR1230_00066

ATGCTGCGCTCTATCGCCACCGTTTCGATTTCCGGCACCCTGCCTGAGAAGCTGCACGCTATTGCGGCGGCGGGGTATCAGGGGGTGGAAATTTTCGAGAACGATCTGCTCTATTATACGGGGACGCCGGCGGAAATCCGCCAGCTTGCCGCCGATTTAGGGTTAAAAATCACGCTTTTTCAACCCTTTCGCGATTTTGAAGGCGCCAGTCGGGCGCAGTTTGCGGCGAATATGGCCCGCGCTCGGCGCAAGTTTGCTCTGATGCGCGAGCTGGGCTGCGAGACGCTGCTGCTGTGCAGCAATGTGCAGCCGGACTGCTCGGCGGATAGCGAATTGCAGGTCGCGGACCTGCGGGCGCTGGCCACGCTGGCGGAAGAGGAGGGGATCGCTATCGGCTATGAGGCCCTGGCCTGGGGAACCCATGTGAACCGCTGGCAGCAGGCCTGGGAGCGGGTGCGGCGGGTGGACAGCCCGGCGTTGGGCCTGGTGCTCGACAGCTTCCATATTCTGGCCCGCGGCGACACGCTGGACGCGCTACCGTCGGTGCCGGTGGAGAAAATCACCTTTGTACAGCTCGCCGATGCGCCGTATATGAAAATGGATTTGCTGGAGTGGAGCCGCCACTTCCGCTGCTTTCCCGGACAGGGGGAGCTGCCGCTGGAGGCGTTTGCCGAGCAGATCACCCGCTGTGGCTACCGCGGCCCCTGGTCGCTGGAGATCTTCAATGACGGTTTTCGCGCCTCGCCGAACGGTGCGACGGCCAAAGATGGTTATCGTTCGCTGCTGTGGCTGGAGGAGCAAACCCGCCGTCGGCTCCCGACGTGCGATGCCTATCTGTTTTCACCGCCGCCGCTGCCGGTCTATCACGGGCTGGAGTTTATCGAGTTTGCCGCCAGCGCCGCCGAGGCGCAGCGCCTGGGGCAACATCTGCAGGCGCTGGGTTTTCAGCACGAGGGAAGCCACCGCTCCAGACAGGTGACGCTGTGGCGCAACGGCGGGGCGCGGATCGTCATCAACCATCAGCCGCACAGCTGGGCCGACCATTTTTATCAACGCCACGGGGTATCGCTCTGCGCGATGGCGCTGCGGGTCGAGCACAGCGCGTCGCTTGTCGCCCGCGCCCGCGCGCTGGGGTATGCCACCTGGCAGGGCGACGCCGGGCCGAACGAAACGCCGATCCCGGCGATCTGCGCCCCCGACGGCAGCCTTATCTATCTCATCGACGCCGGGGAGGCTATCTACGAGCGCGATTTTCATCTGCGTGATGGCGTGACGGTGCGCGAGGACTATCTCGGTATCGATCATCTGGCGCTGGGAATGGAGGCCGACAGCCGCGATAACTGGGTGATGTTCTTCCGTACGGTGTTTGGTTTTTCCCTTGAGCATGAGCAGACGCTGCCGGACCCGTATGGACTGGTGCGCAGCCTGGCGGTGCGCAGCCCGCAGGGCGATATCCGTCTGGCGCTGAATATTTCGCAGAGCCGGGCGACGCAGATCGCCCGCTCCGTTGCCTGCTACCAGGGGGCGGGGCTGCAGCATGCCGCCTTTGCCTGCCGCGATCTGCCGGCCGCCTGCGACCAGCTTGCCGAGGTTGCCCGCCATACGCTGCCGATCCCGGCCAATTATTATGACGATTTGCTGGCGCGCTTTGGCGGAGAGTTGGACGTCGGGCAGCTTCAGCGCCAGCAGCTCCTCTATGACCGCGATCCGCAGGGGGGAGATTTCCTGCATCTTTACACCCGGCCCTTTACCGCCGGCCGCTTTTTCTTTGAGTTAACCGAGCGCCGGGCTGGCTATGCGCTCTATGGCGCAGCGAATGCGGCCGTCCGTCTGGCGGCGATGCAGTATTGTTAG

>IR1230_00067

ATGACCGTCGTTGCCCATGATGAAGCACAATCCCTGAAGGCGCGGATCTTCAGCGCCGCTATCGCTGTCTTTGCTGAACACGGGCTGTCAGGCGCCCGCATGGAGCAGATCGCCACCGAGGCGCAGACCACCAAACGCATGGTGGTTTACTACTTCAAAAGCAAAGAACAGCTCTATCAGGAGGTGCTGCAGCATGTGTATGCGCGGATCCGCGAAACCGAGCAGCAGCTGGGGCTGGAGAATGTGCCGCCGGTGGAGGCGCTGGTGCGGCTGGTGCGCTGGAGCGTGCGCTATCACGCCACCCATGCGGACTATATGCGCGTCATTTGCATGGAGAATATGCAGCGCGGCAAGTGGTTAAAAAGCTCGGGTGAACTGAAGCCGCTGAATCGTACCGCCCTGTCGATTCTGGAAGACATTTTACTGCGCGGCCAGCAGCAGGGCGTCTTTCAGGCGGGGCTCGACGCGCGCGATGTCCACCGGTTAATCAGCAGCTTCAGCTTTTATCAGGTGTCGAACTTCTACACCTTCAGCAGCCTGTATCTTGACGATCCGCTACCCGCCATCGACGATGAAGCGATGGTGGCTCACCATTGTGACATTGCCGTCAGGGCGGTGATCCGCTTCGTGATTTCCTGA

>IR1230_00068

ATGGGAAATAAAGCGAAAGACGACGACCTGTACCAGGAAATGTGCCGCGTGGTGGGTAAAGTCGTGCTGGAAATGCGTGACCTGGGTCAGGAGCCGAAACACATTGTCATTGCCGGTGTGCTGCGCACCTCGCTGGCGAACCGCAAAATCCAGCGTTCGCCGCTCACCGTGGAGGCTATGGAGAAAGTCATTCATGCGCTTTCCGGGCACTGA

>IR1230_00069

ATGAAAAAGACCAAAATTGTTTGCACCATCGGTCCGAAAACCGAATCTGAAGAGATGCTGACCAAAATGTTGGAAGCCGGCATGAATGTGATGCGTCTGAACTTCTCCCACGGTGACTATGCGGAACACGGTCAGCGCATCCAGAACCTGCGCAATGTGATGAGCAAAACCGGTAAGAAAGCCGCTATTCTGCTGGACACCAAAGGGCCGGAAATTCGTACCATCAAGCTGGAAGGCGGCAACGACGTCTCCCTGAAAGCAGGCCAGACCTTCACCTTCACTACCGATAAATCCGTCATCGGCAACAACGAAATCGTTGCGGTAACCTATGAAGGCTTCACCTCCGACCTCGCCGTCGGCAACACCGTCCTGGTGGACGATGGCCTGATCGGTATGGAAGTCACGGCTATCGAAGGCAACAAAGTTATCTGTAAAGTGCTGAACAATGGCGACCTCGGCGAGAACAAAGGCGTTAACCTGCCGGGCGTCTCCATCGCCCTGCCGGCGCTGGCTGAGAAAGACAAACAGGATCTGATCTTCGGTTGCGAACAGGGCGTGGACTTCGTCGCGGCCTCCTTCATCCGTAAACGTTCCGACGTCGTCGAGATCCGTGAGCACCTGAAAGCCCACGGCGGCGAAAACATCCAGATCATCTCCAAAATCGAAAACCAGGAAGGCCTGAACAACTTCGACGAAATCCTCGAAGCGTCTGACGGCATCATGGTTGCGCGTGGCGACATGGGCGTGGAGATCCCGGTTGAAGAAGTTATCTTCGCCCAGAAAATGATCATCGAAAAATGTATCCGCGCGCGTAAAGTGGTTATCACCGCCACCCAGATGCTGGACTCCATGATCAAGAATCCGCGTCCGACCCGCGCTGAAGCCGGCGACGTGGCTAACGCCATCCTCGACGGCACCGATGCGGTGATGCTGTCCGGCGAATCCGCGAAAGGTAAATACCCGCTGGAAGCGGTCACCATCATGGCGACCATTTGCGAACGTACCGACCGCGTGATGACCAGCCGTCTGGACTTCAACAACGACAACCGTAAACTGCGCATCACCGAAGCGGTGTGCCGCGGCGCGGTAGAAACCGCTGAGAAACTGGAAGCGCCGCTGATCGTGGTCGCCACCCAGGGCGGTAAATCCGCACGCGCTGTGCGCAAATACTTCCCGGATGCCACCATCCTGGCGCTGACCACCAACGAAACCACCGCTCGTCAGCTGGTGCTGAGCAAAGGCGTGGTACCGCAGCTGGTGGAAGAGATTGCCTCTACCGATGATTTCTATCATCTGGGCAAAGACCTGGCGCTGAAGAGCGGCCTCGCGCGTAAAGGCGACGTAGTGGTGATGGTTTCCGGCGCATTAGTGCCGAGCGGCACGACTAATACCGCTTCCGTACACGTGCTGTAA

>IR1230_00070

ATGAATCGTACTAAACTGGTACTGGGCGCGGTAATCCTGGGTTCTACTCTGCTGGCTGGTTGCTCCAGCAATGCTAAAATCGATCAGCTGTCTTCTGACGTTCAGACTCTGAACGCTAAAGTTGACCAGCTGAGCAACGACGTGAACGCAATGCGTTCCGACGTTCAGGCTGCTAAAGACGACGCAGCTCGCGCTAACCAGCGTCTGGACAACCAGGCTCACTCTTACCGTAAGTAA

>IR1230_00071

ATGAAACGCAAAACGATGATCACCTTAGCCCTTCTCAGCGCCCTTGGCGCCTCCTCGGCCGCCTGGGCCGTTGATTATCCGCTTCCTCCCGCCAACAGTCGGCTGATTGGTCAGAATCAGTACTGGACGGTCCAGGAGGGAGATCGCAACCTGCAGGCGATTGCGCGCCATTTCGATACCGCGGCGATGCTGATCCTCGAAGCCAACGATACGATTGCGCCGGTGCAGCCGAAGCCCGGCACTCAGGTCCTTATCCCTTCTCAGATGCTGCTGCCCGACGTGCCGAGGGAGGGCATTGTCGTCAACCTCGCCGAACTGCGGCTGTATTACTTCCCGCCGGGAGAGAATCAGGTGCAGGTCTATCCGCTGGGCATTGGCCAGTTAGGTCTGGAAACGCCGGAGATGACCACCCGCGTGGGGCAGAAGATCCCTAACCCTACCTGGACGCCTACCGCCGGCATCCGCGCGCGCTCGCTGGAGAAAGGGCTGACGCTGCCAGCGGTGGTCCCGGCCGGACCGAACAATCCCCTGGGCCGCTATGCGCTGCGTCTGGCCTACGGTAACGGTGAATATCTCATCCACGGAACTAACGCCCCAGACAGCGTTGGTCTGCGCGTCAGCTCGGGCTGTATGCGGATGAACGCTGATGACATCAAAGCGCTGTTCAGCCAGGTGAAAACCGGGACGCCGGTACGGATTATCAATCAGCCGGTGAAGTTTGCCGTCGAGCCGGATGGTAAACGCTATGTCGAAGTCCACAGGCCGCTTTCGCAGACCGAAGGGGAAAACACCCGGACCATCGCTTACACGCTGCCAGCGGCGTTTCACGCCTTCGCAGAGGATAAAGCAGTGGATGACCTGCAGTTGAAAAAAGCGATGTCGAGAAGGGCGGGGTATCCGGTGGTGGTGTCGGCGGGGGCAGGTAGCACGGCGACGTCGCTGTCGGCGCAGAATAGTTCGTCTGACAACGGTCTGCTTACCCAATAG

>IR1230_00072

ATGGCGGCACTACCGGACAAAGACAAACTGTTGCGTAACTTTAGCCGTTGCGCCAACTGGGAAGAGAAGTATCTCTATATCATCGAGCTTGGCCAGCGTCTGGCGCCGCTAAGCCCGGAAGAACATAGCGTGCAGAACATTATTCAGGGCTGTCAAAGCCAGGTCTGGATCGTGATGGACCAGGATCCCACAGGCGTGATCACCCTGCGCGGCGATAGCGATGCGGCGATCGTCAAGGGTCTGATAGCGGTGGTGTTTATCCTCTATGACCGGATGACCGCTCAGGACATCACCGAGTTCGACGTGCGTCCGTGGTTTGAAAAAATGGCGCTCACCCAGCATCTCACCCCCTCCCGCTCGCAAGGCCTCGAAGCCATGATCCGCGCGATACGCGCGAAAGCGGCAAATATTAGCTAG

>IR1230_00073

GTGCTGGGGACGGAAAACCCGCTGGCGGCGCTGATTGCTCTTGCGCATCAGCATGGCGCGAAAGTGCTGGTGGATGGCGCCCAGGCGGTGATGCACCACCCGGTGGACGTCCAGGCGCTGGGCTGCGATTTTTATGTTTTCTCCGCCCACAAGCTGTATGGGCCGACCGGGATCGGCGTGCTCTACGCCCGGTCCGAGCTGCTGCAGACGATGGCGCCTTGGGAAGGGGGCGGGTCGATGATCGCCACCGTCAGCCTGACGGAGGGCACCACCTGGAATCAGGCGCCGTGGCGCTTTGAGGCCGGCACGCCGAATACCGGGGGGATCATTGGCCTCGGCGCGGCGCTGACCTACGTCAGCCAGCTGGGACTGACGCAGATTGCCGAGTATGAGCAGACGCTGATGCGCTACGCGCTCGATGCCCTGCGCGCGGTACCGGATCTGATCCTTTACGGCCCGGCGCAGCGTAAAGGGGTAATTGCTTTCAACCTCGGTCAGCATCACGCCTATGACGTCGGCAGCTTCCTCGATAACTACGGGATCGCCGTGCGGACCGGACACCACTGCGCGATGCCGTTGATGGCCCGGTATCAGGTACCGGCTATGTGTCGGGCATCGCTGGCGATGTACAATACCACGGAAGAGGTGGACCGCCTGGTGGCCGGCCTCCAGCGGATCCGCAAACTGTTAGGGTAA

>IR1230_00074

ATGACATTTTCAGTAGAACAGGTTCGCGCCGATTTTCCGGTACTGAACCGGGAGGTCAACGGTCAGCCGCTGGTCTATCTCGACAGCGCCGCCAGCGCCCAGAAGCCGGAGGCAGTGATCGGCGCCGAAGCGGAATTCTATCGCCACGGCTACGCGGCGGTGCACCGCGGCATCCATACCCTCAGCGCGGAAGCCACCGCGCGGATGGAAGCCGTGCGTCAGCAGGTGGCCACCTTCCTTAACGCCGGGTCGGCGGAGGAAGTGGTGTTCGTCCGCGGCACCACCGAGGGTATCAACCTGGTGGCCAACAGCTGGGGAAACGCCAACGTCGGCGCCGGGGATAACATCATCATCAGCGAAATGGAGCACCACGCCAATATTGTGCCGTGGCAGATGCTGTGCGCCCGCGTCGGGGCTGAGCTGCGGGTGATCCCCCTGAACCCGGACGGTACGCTGCAACTGGATGTTGTCCCGTGA

>IR1230_00075

ATGGCTGGCTTACCGAACAGCAGTAACGCGCTGCAGCAGTGGCACCATCTGTTTGAAGCGCAGGGCGGCCCGCGTACCCCGGAGGCCAGTCAGCATCTGCAGCAGCTGCTGCGCCTTGGGCTGCCGACGCGCAAGCACGAGGACTGGAAATACACTCCGCTGGATGCCCTACTTAACGGTCGCTTTGTCGCCGATGAGGGGGCCTCTCTCAGTGCTGAACAGCGTGATGCGCTGGCGCTGCCGCTGGACGCCTGGCGGCTGGTGTTTATCGATGGCCGCTATCATCCTCAGCTGAGCGACGATCTTGCCGCCAGCGGCGTGGAGGTCAGTGTCGATAATCAGCGCCAGCATCTGCCGGATGCCCTGCAGCCGGAGGTGTTTCTCCATCTGACCGAAAGCCTGGCCCAGACGGTGACCCGCATTCGCGTGCCGCGCAACCGCCGCCTGGATAAACCGCTGCTGCTGATGCATATCACTAGTGGCCTGGCCGGCGATGCGCTGAATACCGCCCACTATCGCCATCACCTGGCGCTGGAGAGCGGGGCGGAGGCGACGATTGTCGAGCATTACCTGAGCCTCAATGAGCAGCCGCATTTCACCGGCGGGCGATTGACCATGACGGTGGCCGATAACGCGCACCTCCAGCATATCAAGCTGGCATTTGAGAATGCCCGCAGCTATCACTTCGCCCATAACGACCTGCTGCTGGGCCGCGACGCCTCGGCCTTCAGCAGCAGTTTCCTGCTTGGCGGCCAGGTGCTGCGTCACCAGACCAGCACCCGCCTGGGCGGCGAAAACAGCAACCTGCGCCTCAATTCGCTGGCGATGCCGGTGAAAAATGAGGTCTGCGATAGCCGCACCTGGCTCGACCATCAGGTCGGCTACTGCACCAGCCGCCAGCTGCACAAAACCATCGTCAGCGATAAGGGGCGGGCGGTGTTTAACGGGCTGATCAACGTCGCGCCGCTCGCGCTGAAAACCGACGGTCAGATGACCAACAATAATCTGCTGCTCGGGCGGCTGGCGGAGGTCGACACCAAACCGCAGCTGGAAATTTACGCCGATGACGTGAAATGCAGCCACGGGGCGACGGTGGGGCGCATTGACGAAGAGCAGCTGTTCTATCTGCGCTCGCGAGGTATCGAACAGCAGGCGGCGCAGCAGATGATCCTCTACGCCTTCGCCGCCGAGCTCACCGAGGCTATCCGCAGCGACGCGCTCAGAGAGCAGGTGCTGGCGCGTATTGGACAGCGTTTGCCGGGAGGCACTGTATGA

>IR1230_00076

ATGTTAAGTATTCAAGATTTACACGTCGCCGTCGAAGATAAAGCGATCCTGCGCGGGCTGAACCTTGAGGTGCGCCCGGGAGAGGTCCACGCCATTATGGGGCCGAACGGCTCAGGCAAAAGTACGCTTTCGGCCACCCTGGCCGGGCGTGAAGATTATGAGGTGACTGGCGGTCGCGTTGAATTTAAGGGCAAAAACCTGCTCGAACTCGCTGCGGAAGATCGCGCCGGGGAAGGTATCTTTATGGCCTTCCAGTATCCGGTAGAGATCCCCGGCGTCAGCAACCAGTTTTTCCTGCAGACTGCGCTGAATGCCGTGCGCAACTATCGTGGCCAGGAGGCGCTGGATCGTTTCGATTTCCAGGACTTAATGGAAGAAAAGATCAAGCTGCTGCAGATGCCGGAAGATCTCCTGACCCGCTCGGTGAACGTTGGCTTCTCCGGCGGTGAGAAAAAGCGCAACGATATTCTGCAAATGGCGGTGCTTGAGCCGGAGCTGTGCATTCTCGATGAATCGGATTCGGGGTTGGATATCGATGCCCTGAAAATCGTCTCCCAGGGAGTGAATGCCCTGCGCGACGGCAAACGCGCCTTTATCATTGTCACCCACTATCAGCGCATCCTTGACTATATCAAGCCGGACTATGTCCATGTGTTGTATCAGGGGCGGATTGTGAAGTCGGGCGATTTCTCTCTGGTCAAACAGCTGGAGGAGCAGGGCTATGGCTGGCTTACCGAACAGCAGTAA

>IR1230_00077

ATGTCGCGTAATACTGAAGCAACTGACGATGTCAAAACCTGGACCGGCGGCCCGCTCAACTACAAAGAGGGCTTTTTTACTCGCCTGCAAACCGATGAGCTGGCCAAAGGCATTAATGAAGAAGTCGTTCGGGCTATCTCGGCTCGCCGCAATGAGCCGCAGTGGATGTTGGAGTTTCGTCTGAATGCCTACCGGGCGTGGCTGGAAATGGAGGAGCCGCACTGGCTGAAAGCGCATTACGATAAGCTGAATTATCAGGATTACAGCTACTATTCCGCGCCCTCCTGCGGCAACTGCGATGAGACCTGCGCCTCCGAGCCTGGCGCGGTGCAGCAGACCGGGGCCAATACCTTTCTGACCAGCGAGGTGGAAGAAGCCTTTAACCAGCTGGGCGTGCCGGTACGCGAAGGGCGCGAGGTGGCGGTGGACGCGATATTTGACTCGGTGTCGGTGGCGACCACCTACCGAGAAAAGCTGGCCGAGCAGGGGATCATCTTCTGTTCTTTCGGCGAGGCGATCCACGATCATCCGGAACTGGTGAAAAAGTATCTGGGCACCGTAGTGCCGGGGAATGACAACTTCTTTGCCGCGCTGAACGCCGCGGTGGCTTCGGACGGCACCTTTATCTATGTGCCGAAGGGCGTCCGCTGCCCGATGGAGCTGTCGACCTATTTCCGCATCAACGCCGAAAAGACCGGGCAGTTTGAACGCACCATTCTGGTGGCTGATGAAGGCAGCTACGTCAGCTATATTGAAGGCTGCTCGGCGCCGGTGCGCGACAGCTATCAGCTGCATGCCGCGGTCGTTGAAGTCATCATCCATAAAGACGCGGAAGTGAAATACTCCACGGTGCAGAACTGGTTCCCCGGCGATAACAACACCGGCGGGATCCTCAACTTCGTCACCAAGCGCGCCCTGTGCGAAGGGGAGAACAGCAAAATGTCATGGACCCAGTCGGAAACCGGCTCGGCCATCACCTGGAAATACCCCAGCTGCATCCTGCGCGGCGATAACTCGATCGGCGAATTCTTTTCGGTGGCGCTGACCAGCGGCCATCAGCAGGCGGACACCGGCACCAAAATGATCCATATCGGCAAGAACACCCGCTCGACCATCATCTCGAAAGGGATCTCCGCCGGACACAGTCAGAACAGCTATCGCGGGCTGGTCAAAATCATGCCGACCGCCACCAACGCCCGCAACTATACCCAGTGCGATTCGATGCTGATCGGCCCGGACTGCGGGGCGCATACCTTCCCGTATGTTGAATGCCGCAACAACAGCGCCCAGCTGGAGCACGAGGCGACCACCTCGCGGATTGGCGAAGATCAATTGTTCTACTGCCTGCAGCGCGGGATCAGCGAAGACGATGCCATCTCGATGATCGTCAACGGTTTCTGTAAAGACGTCTTCTCCGAGCTGCCGCTGGAGTTCGCTGTAGAGGCGCAAAAATTGCTGGCCATTAGCCTTGAACATAGCGTCGGCTGA

>IR1230_00078

ATGGAAGTACAAGCAGGAACTTTTAATCCCGCCGATTTCAGCTGGCAGGGACTGACCATGACGCCGGCCGCTGCCGCGCATATCCGCGATCTGATGCGCAAGCAGCCGGATAAGAAAGGGCTGCGGCTGGGCATTAAAACCAGCGGCTGCGCCGGTTTTGGCTATGTGCTCGAAATGATTGCCGAGCCGGCGCCCGACGATCTGCTCTTTGAATCTGACGGGGCGAAGCTGTTTGCGCCGCTGCAGGCGATGCCGTTCATCGACGGCACCGAGCTGGATTATGTCCGGGAAGGTTTAAATGAAATCTTTAAATTTCATAACCCGAAAGCGCAGCATGAGTGCGGCTGCGGCGAAAGTTTTGGGGTGCAGGCGGAGTAA

>IR1230_00079

ATGAAAGATAAAGATGAACAAACGGCATTGATTGGCATGGCCATCGGCGCGGCGGTCATTAGCTTAGTGGCTACACAAAAGCAGATTAATCAGGGGAGTATCGTGGATGAACTGGTGAGGCTGGGCAGACAGAAGGGGGACGGGGTAGAGGATGAGGTCTTTGTCCAGGCCGCCCGGCTGGTGAGTAAAGGCACCTAG

>IR1230_00080

ATGCCTAATTCGACCTTATTTTTCATCACCCTTTGTTTCTCTCGCGTCCTGGTGACCTTCTTCTTCTGGATGGCAGGCATCTACGGCGTCTTTCATTTCGCGGAGATCGTTCAGGAGATGGTGACCGTCGGCCTGCCGTGGCCGGTCTTGTTCGCGGCGGGCACGATTTTTTGCCAGCTGGCGGGTTCGGCGCTGATCGTTTTTAATCCTGCCGGCTACGGCTGGATTGGATCGGGGATGCTGATTGTGTTTACATTACTCACTATTCCGCTGGGACACGCCTTCTGGGCCTTCAGTGAACCCCGGCGCACTGGGGAATTCCATATTGCGCTTGAACACATTACCGTGGTCGGAGGATTAATTATGAGCGCGCTGTTTGCAGGCTATCGGCGATGA

>IR1230_00081

ATGAGCGAAACCCCTCGTCAGAAAGGTCGGCCAAAAGATCCGTTGAAAACGCAGGCGATACTTCAGGCCGCCCGTAAGCTGTTTCTGGAACAGGGCCTGGAGGTGACAACGGCCGAAATCGCCCGCGTAGCCGGGGTGGCGAAAGCCACCCTGTATGCCAATTTCAGCGATAAAGAACATCTGATAGAGGCGGTCTTACGCCAGGAATCGGATCTCACCATCAGCGACCATGATTTCGCCCAGCGCCATCATCTGCCGCTGATCGAGGTACTGACGGCGTTTGGTTATCGCTTTGTCCGCTTTATCAACCAGCGGGAACTGACCGGCTGGGACCGACTGATCGCCTCGGCTGCCGTGCGTCACCCCGATCTCCCCGGCCGCTTTTACGCCGCCGGGCCGGGGCGCGCGCAGCAGATGCTGGAAGCGATCATCGCCGAGGCGATCGAAGCCGGTACTTTACGAGCCTGCGATCCGCAGGAGGCTGCCGACGAACTGGCCGGTTTATGGCTGGGAATGACCAGCCTGGCGATAAAACTTGGCGCAAGACCTCCGCTGTCCGACGACGAAATCAAACAGCGGGTTGAGCGCGCCCTGGACGTCTTTCTGCGCGCGTATTCCCTGGCGCCGGGCTGA

>IR1230_00082

ATGATTGTTTCAGCCCCCAGCGATTATCGCGAAGCCGCTCGTCGTCGTCTGCCCCGCTTCCTTTTCGATTATATTGACGGTGGCGCGGTGGCGGAAAACACCATGAACGCCAACGCCGCCGAGCTTGCCTCGGTAGCTCTTCGCCAGCGCGTACTGTGCGGGGCCGGCGAACCGACGCTGGCGACCACGATCCTTGATGCCCCATGGGCGATGCCCGTGGCGCTGGGGCCCGTCGGCGCCACCGGGATGTACGCCCGCCGCGGCGAAGTCCAGGCGGCCCGCGCCGCCTCCCGCGCCGGGATCCCGTACACCTTATCCACCGTGTCGGTCTGCTCGATTGAAGAGGTGGCGAGCCACGCCAGCGGCGCGCTCTGGTCCCAGCTGTACGTACTCAAAGATCGCGGCTATATGCGTAACGCGCTGGAGCGCGCCTGGGCCGCTGGCATGAAAACCCTGGTATTTACCGTCGATATGCCAATCCCCGGCTCGCGGTATCGCGATAACCGCTCAGGGATGTCCGGGCCGCACGCCACCCTGCGACAATATCTCCAGGCTTGCACCCATCCGCGCTGGGCGATGAACGTCGGGCTGGCGGGCAGACCCCTGTCGTTTGGTAATATCGAAGCCTACACCGGCCACAAAATGACCATGGACGACTATATGGGGTTCATCAGCAACAACTTCGACCCGTCCATCGCCTGGCACGATCTGGAGTGGATCCGCGACAGCTGGCAAGGAAAATTAATCATCAAAGGGATCCTCGATGCCGACGATGCGCGCAATGCCGTGCGGCTGGGCGCCGCTGGTATTGTGGTGTCGAATCATGGCGGCAGACAGCTCGATGGCGCCATCCCCACCGCCCGGGCGCTGCCCAGGGTGGTGGATGCGGTCGGCGACGATCTGACAGTGCTTGCCGATTCCGGGGTGCGCTCCGGCGTGGACGTTATACGTCTGCTGGCCCTCGGCGCGAAAGGCGTGCTGCTCGGTCGGGCCTATATTTATGCCCTGGCCGCGGCGGGCGAAGCGGGCGTGGCGCATCTGCTGCGCTTATTTGCCGAGGATATGAAGGTTACCATGACCCTCACCGGCGCGACCTCGCCATCGGCCATCAGCCTCGATTGCCTTGATCGCCTCGAACAGGATCAACACCGCACTCATGCGGTGCCGGTATCCTTACCCGCCTGA

>IR1230_00083

ATGAGCGCCGGGCGTTTTAATCTCTCCGCGCTGGCCGTCCGCGAGCGGTCGGTGACCCTGTTTTTGATTATCCTTATCTCCGTCGCCGGGCTGGTGGCGTTCTTCGGACTCGGGCGAGCGGAAGATCCGCCCTTTACGGTCAAGCAAATGACGGTCATTACCGTCTGGCCCGGCGCCACCGCGCAGGAGATGCAGGATCAGGTCGCCGAGCCGCTGGAAAAACGGCTCCAGGAGCTAAAGTGGTACGATCGCACGGAGACCTATACCCGCCCTGGCATGGCGTTGATCACCCTGTCGCTGCAGGATCAGACCCCGCCTTCCGAGGTGCCGGAGCAGTTTTATCAGGCGCGCAAAAAGCTCGGCGACGAGGCGAAAAATCTGCCTGCCGGCGTCTCCGGCCCGATGATGAATGACGAATTCGCCGATGTCACCTTTGCCCTCTTTGCCCTGAAAGCACGGGGAGAACCGCCGCGGCAGCTGGTGCGTGACGCCGAAGCCCTGCGCCAGCAGCTGCTGCATGTTCCCGGGGTGAAAAAAGTGAATATCCTCGGCGAACAGGCGGAGCGCATTTATCTCTCGTTTTCTCATGACCGTCTCGCCACCCTGGGCCTTTCACCAGAGGCGATTTTTGCCGCGCTGAACAGCCAGAATGTGCTGACCGCCGCCGGGGCTATCGAGACGCGGGGCGGGCAGATCTTTATCCGCCTCGACGGGGCGTTCGACCGCCTGCAGCAGATCCGCGACACGCCGATTATTGCCGGGGGCAGAACGCTGAAACTGGCCGATGTCGCCACGGTTGAGCGGGGATATGAAGATCCCGCGACCTTCCTGATCCGCAATCAGGGCGAACCGGCGCTGCTGCTGGGCGTGGTGATGCGCGAGGGCTGGAATGGTCTGGCGCTGGGGAAAGCGCTGGACGCCGAAACGACCAGCATCAATCAGAGCCTGCCGCTCGGCATGTCGTTGACCAAGGTGACCGATCAGTCGGTGAATATCAGCGCCGCGGTCGATGAGTTCATGATCAAATTCTTTGTCGCCCTGCTGGTGGTGATGTCGGTGTGCTTTGTCAGCATGGGGTGGCGCGTGGGCGTGGTGGTCGCGGCGGCGGTGCCTTTAACCCTCGCCGTGGTGTTTGTGGTGATGGAGGCGACCGGCAAAAACTTCGACCGCATCACTCTCGGCTCGCTGATCCTCGCCCTGGGGCTGCTGGTGGATGATGCCATCATTGCCATCGAAATGATGGTGGTCAAAATGGAGGAGGGGTACGACCGCCTCAAAGCCTCGGCCTATGCCTGGAGCCATACCGCGGCGCCTATGCTGGCCGGTACCCTGGTGACGGCCGTGGGCTTTATGCCTAATGGATTTGCGCAGTCCACCGCCGGGGAGTACGCCAGCAATGTGTTCTGGATCGTCGGTATCGCGCTGATTGCCTCCTGGATTGTGGCGGTGATTTTTACCCCCTGGCTGGGGGTGCACCTGTTGCCGAACAGAAAGCCCGCGGCGGCCGGCCACGCCGCGCTGTATGACACCCCGCGCTATCAGCACTTCCGTCGGCTGCTGACCCGGGTTATTGCTCACAAATGGCGCGTGGCCGCCGGGGTGGTGGCGCTGTTTATCGTGGCGATCCTGGGCATGAGCGTGGTGAAAAAGCAGTTTTTCCCCACCTCCGATCGTCCGGAAGTGCTGGTTGAAGTGCAGCTGCCTTACGGGTCGTCGATTAGCCAGACCAGCGCCGCGGCGGCGAAAATTGAGCACTGGCTGCACCTGCAGCCGGAGGTGAAGATTGTCACCAGCTATATCGGCCAGGGCGCGCCGCGCTTTTACCTGGCGATGGCTCCGGAATTGCCCGATCCCTCCTTCGCCAAACTGATGGTGTTGACCGACGGGCAGGGTGCCCGCGAGGCGCTCAAGCGGCGGCTGCGGGAGGCGGTGGCCAATGGCCTGGCGCCGGAGGCGCGGGTGCGCGTCACTCAGCTGGTGTTTGGCCCTTATTCGCCTTATCCGGTCGCCTGGCGGGTGATGGGGCCCGATCCGCACGCTCTGCTCGACATCGCCGAGCGGGTTAAATCGGTGCTGCAGGCCAGTCCGCTGATGCGCACCGTCAATACCGACTGGGGTTCGCGGGTACCGGTGATGCATTTCAGCCTCAATCAGGACCGGCTGCAGGCGAGCGGACTCAGCTCCCAGTCCGTCGCCCAGCAGCTGCAGTTCCTGCTGTCAGGGATCCCGATCACCACCGTCCGGGAAGATATTCGCGCCGTGCAGGTGATCGGCCGCGCGGCGGGCGACATCCGTCTGGATCCGGCGAAAATCGCCGACTTCACCCTGGTGGGCAGCGGCGGGCAGCGGGTTCCCTTGTCACAGATTGGCGACGTGTCGATCAGGATGGAGGACCCGCTGCTTCGCCGTCGCGACCGCACGCCGACCATCACCGTCCGCGGAGATGTTGCGGAGAACCTGCAGCCGCCGGATGTCTCCACCGCGCTGATGAAGCCGCTGCAGCCCATTATCGACTCGCTGCCGCCTGGCTATCGCATCGAGACGGCGGGGTCGATTGAGGAATCCGGCAAGGCCACCCGGGCGATGGTGCCGTTATTTCCGATAATGATCGCCCTCACGCTGCTGATCATTATCCTGCAGGTGCGTTCGCTGTCGGCGATGGTCATGGTTTTCCTGACCGCGCCGCTGGGGCTGATTGGCGTGGTCCCGACGCTGCTGCTGTTCAATCAGCCGTTTGGCATCAATGCCCTGGTGGGCCTGATCGCCCTGTCGGGGATCCTGATGCGCAATACGCTGATCCTGATTGGCCAAATCCATCATAACCAACAGGCGGGGCTCGATCCGTTCCACGCGGTGGTGGAGGCGACGGTGCAGCGCGCCCGCCCGGTTCTGCTGACCGCGCTGGCGGCGATCCTGGCGTTCATTCCGCTCACTCATTCGGTCTTCTGGGGAACGCTCGCCTATACGCTGATTGGCGGGACGCTGGGGGGAACCATCATGACCCTTATCTTCCTGCCGGCCATGTACGCGATCTGGTTCCGCATCCGTCCGGAGAACACGGTACAACAGACAGAGTTGCACCTTCAGAGGTAA

>IR1230_00084

ATGCTCAGGCTCAACGCCGTCCATCTTGCCGTCTGCCTCCTGCCGCTGGCCCTCGCGGGCTGCGGCGAACCTGCCGATCATGACGACCCCCGCATCCGGCCGCCGCTGGTCCGGGTGGCGACCGTCGAGCGCGCGGAGGCCGGCTCGCGGGCCTTTACCGGCGTGGTAGTGGCCCGAACCCAAAGCGACCTCGGCTTCAGGGTGGCGGGAAAAGTGCTTGAACGGCGGGTGGAAACCGGGCAGAGCGTCAAGCGCGGTCAGCTGCTGCTGCGCCTGGATCCGGCTGATCTAGCCTTGCAGGCGCAGTCCCAACAGCGGGCCGTAGATGCCGCGCGGGCGCGAGCCAAAAAGGCGGCCAACGATCTGGCCCGCTATCGCGGCCTGGTGGCGAGCGGCGCCATCTCGGCCGCGGAATTCGACCAGATTAACGCGGCAGCAGAGGCGGCGAGAGCCGACCTCAGCGCGGCCCAGGCGCAGGCCAATGTGGCGCAAAACGCCACCGGCTATGCCGGACTGCTGGCGGATGCTGACGGCGTGGTGGTGGAAACCCTCGCTGAGCCGGGGCAGGTGGTCAGCGCCGGGCAGGTGGTGATCCGCCTGGCGCGGGCGGGGCAGCGCGAAGCGCGGGTGCAACTGCCGGAGACGCTGCGCCCGGCGGTCGGCAGCGAGGCGCTGGCGACACGCTATGGCAGCGAATCTCAGCCGGTCACGGCGACCCTGCGCCTGCTTTCGGACGCGGCTGACGCCACCACCCGCACCTTTGAAGCGCGCTATGTACTGAACGGCGCGCTGGCGAATGCCCCGCTGGGGTCTACCGTGACCCTGCGCATCGGCAACGACCAGGCGCCAGGCCAGGTGCTGGCGGTGCCCCTGGCGTCGGTCTACGATCCGGGTAACGGCCCTGGCGTCTGGCGCATTGCCTCTCGTCCGGCCACCGTCTCCTGGCAACCGGTGACCGTGCTCGGCCTGGATGATGAAACCGCGCGGGTGACCGGCCCGCTGAAGCCCGGCGAGCCGATCGTCGCCCTGGGCGCCCATCTTCTGCACCAGGGCGAGGCGGTACGTCTGGCGGAACGACGCGAGCATAATGCCGCCGGGAGCCAGCCATGA

>IR1230_00085

ATGGCGCACTTTAGTCGCTATGGCTATGAGAAAACGACGGTCACCGATCTCGCCAAAGCGATAGGCTTTTCCAAAGCCTATATCTATAAATTCTTTGACTCCAAGCAGGCGATTGGCGAGGCGATTTGCGCCAGCCGGCTGGAGAAGATCATGGTGGCCGTCAGCGAGGCCATCGCCGATGCGCCGTCCGCCAGCGAGAAACTGCGCCGCCTGTTCCGGGCGCTGACCGAGGCCGGCAGTGAACTGTTTTTCGAGGATCGCAAACTGTACGACATCGCCGCCGTCGCGGCGCGCGATAAATGGCCTTCAACGGAACAGTATGCCGGTCATCTGCAGCAGCTGATTGGTCAAATCCTTGTCGAAGGCCGCCAGGCGGGTGAGTTCGAGCGCAAAACCCCGCTGGATGAAGCGACCCTGGCGGTGTATATGGTGATGTGTCCTTTCATCAACCCGGTGCAGTTGCAATACAATCTCGACACCGCGCCCACCGCGGCGGTGCTGCTTGCCTCCCTGATCCTGAGAAGTCTCTCCCCCTGA

>IR1230_00086

ATGAACAGCAGCGGCAAGGTACTTATTCTGGGAGCAAGCGGGGGCATCGGCGGGGAAGTGGCGCGCCGGCTGGTGGCAGACAACTGGCAGGTTCGGGCGCTGAAGCGCGGCGCCCAGATGCGTGATCCCGAAGATGGCATACAGTGGATAGCCGGCGACGCGCTGGATGGCGGCCAGGTCGCGGCGGCGGCTGCCGGTTGCGACGTCATCGTCCATGCGGTGAATCCGCCGGGCTACCGGCACTGGCGGCAACAGGTACTGCCTATGCTGCGCAACACCCTGCAGGCTGCCGAACGGCAGCGGGCGCTTGTAGTCCTGCCCGGCACGGTCTACAACTATGGACCGGATGCCTTTCCGCTGATTGCTGAAGAGGCCGCACAACAGCCGGTGACCCGCAAAGGCGCCATCCGGGTGGCGATGGAGCTGGCGCTGAAGGATTACGTGCAGCGCGGCGGCCGGGCGTTGATTGTTCGGGCGGGCGATTTTTTTGGCCCGCGCGCCGGAAATAACTGGTTTTCTCAGGGGCTGGTCAAGCCTGGTCAGCTTCCCCGCATCATTAGCTACCCGGGGGCGATCGGCGTGGGGCATCAGTGGGCCTGGCTGCCCGACGTGGCCGCCACCATCGCCGCGCTGCTCGCCCGCAGGCGCGAGCTTGAGCCGTTCGCCCGCTTTCATATGCAGGGCCACTGGGACCCGGACGGCAGCGAAATGAGTCAGGCGATCCAGCGGGTGGTCGCCCGCTATGGCGGCAGGGCGGTAGTGAAGTCCTTTCCCTGGTGGCTGGTGAAGATGGCGGCGCCCTTCAACGCCACCCTGCGCGAAATGGTCGAGATGCACTATCTCTGGCGTCTGCCGGTACGCCTGCGCAACGATAAACTGGTTGATTTTTTAGGCGCGGAACCGCATACCCCGCTCGACAGCGCGGTTTATCAGACGCTGCAAGGCCTGGGTTGTCTGCCCGCCGGCGCGATAAACCAAGAGGCGGGTGAGGCGTAA

>IR1230_00087

ATGAATACCTCTATTCCCTGGGAGTGGTACCGAACCTTTCTCGCCGTCCTGCAGGAAGGCTCTTTATCGGGCGCATCGCGCACCCTGAACATCACTCAGCCGACGGCGGGGCGCCATATTGCCGGGCTGGAAACCGCCCTCGGCCAGGCCTTATTTACCCGCTCGCAAACCGGCCTGCTGGCGACCGACGCGGCGCTGGCGCTGCGGGTGCATGCCGAGGCGATGGACAACACCGCCCGGGCGCTGGAACGCACGGCGGCCAACTTTAGTCGCGACAGAGCCGATCTGCGTGGCGTGGTGCGGGTGGCCGCCAGCGAGGTGGTCGGCGCCGAGGTGCTGCCGCCGCTGGTTGCCCGGCTCAGGCAGGCGTGCCCGAATATCGTCATCGAACTGATGCTCTCCAATCGATTTCAGGATCTGCTTCACCGGGAGGCGGATATCGCGGTGCGGATGGTCGCCCCGCAGCAGGAGCAGCTTATCGCCCGCCGTCTCGGCAGGATTGAGCTTGGGTTACATGCCACCGCCGCCTACCTGACGCGCCAGGGCCTGCCCACGACCCTCGACGATCTGGCCAGCCACGCGCTGATTGGTTTCGATAGCGCCACCCCACTTGTTCGCCGGGCGCTGCAGGCTTACCCTCGTTTTCAGCGTGAGGCCTTCGCGATGCGGACCGATAGCGACCTCGCCCAGTTGAGCCTGATTCGCGCCGGAGCGGGGATCGGCATCTGCCAGGTGCCCCTTGCGGATGGCATTATTCCCCTGCAGCGAGTGCTGGCGGCCGACTTTTCTCTGTATCTTGATACCTGGCTGGTAATGCATGAGGATCTGCGTCACAGCCCTGCCTGTAAGCGGGTATTTGATTTTCTCGCCCAGGGGTTGCAGGCGTATATTCGTGGCCCACTGGCGAGCTGA

>IR1230_00088

ATGAAGAAGGTCCTTTGCGCGTTAGGCTTGATGTTCACCGCCGTCAGTTCCGCGCTGGCAACAACCTACCCCCTGACGATTGAAAACTGCGGCTATCAGGAGACCTTCACCCGGCCGCCGGAGCGCGTGGTCGCGCTGGGGCAAAATACGGTCGAAATTCTGCTGCTGCTCGGGCTGCAAAAGCAGGTGGTCGCCAGCGCCTTCTGGCCGACCAGCGTTTTACCGCAGCTGGCTGAGCAAAACGCGAAGATTAAAACCTTGACGGTGGAAATTCCGAGCCTGGAATCGGTGCTGGCGCAAAATCCTGATTTCGTTCCCGCCCAGTTGCCGCTGCTGCTGGGGCCGGAAAGCAAAGTCGCCAGGCGTGAAGATCTCGCCACCGTCGGCGTGAACAGCTACGTTTCACCGGGGATGTGCGCCACTAAAAAGGCCACCGGCGATATGTACGGCAGCCGGCAAAAGCTGTGGGATATGACCTGGCTGTATCAGGAGATTAGCGATTTCGCCCGCATTTTCAACGTCGAAGATCGTGGTCAGGCGCTAATTGCCGATTTCAAAAAACGTGAAGCCGACCTGCGCCAGGAGTTTGGTAAAAGCAAAAAGGATCTCTCCTTCGTCTTCTGGTTCTCCAGCGCCTCGCCTTCGGCCGATGCCTATGTCGGCGGTAAAAACAGCGCCTCCGGCTTTATCGCCAGCGTGCTGGGCGGTCATAATGCGATAACCTCCGAGACCGAGTGGCCCACGGTGAGCTGGGAGAGCATTATTGCCGCCAACCCGGATGTGATCGTGGTCGCCAGCCTGGATCGTAACCGCTGGGCGCTGGATAAGGCCGAGGAGAAGATCAAATTCCTCAAAAGCGATCCCGCCGTCAGCCAGCTGGAGGCGGTGAAAAAAGGCCATATCGTGGTCATGGACGGTCAGGCGATGAATCCGACCATTCGCACGCTTTACGGCGCGGAACAGGTGGGCGAGCAGCTGAGAAAAATGGGGCTGAACTGA

>IR1230_00089

ATGAGCGTCGCCGCGATTGAGACGCGCCGCAGCATACTGCTCACCGGCTGGTGTGTACTGGCAGCGATCGTGCTGGCGCTGGTGATAGCGGTGGGCGTCAGCGTCGGCGAGCTGGCCATCCCGCTGCAGAATGTCTTTTACGCTATCAGCAACAGAACCGGCTTAACCGCTGAGCCGCTCAACCGTATCTATGAGAGCGTAATCTGGGATTTCCGCCTCAGCCGCGCGCTGGTCGCCGCCTGCTGCGGCGCCGGCCTCGCTATCTGCGGCGTCGTGCTGCAAAGCTTGCTGAAGAATGCCCTCGCCGAACCCTACGTCCTCGGCGTCTCCGCCGGGGCCTCCACCGGCGCGGTGTCGATCGTGGTGCTGGGCCTTGGCGCCGGTGCGATTTCCCTCTCGGCCGGGGCCTTTGCCGGGGCCTTCGCCGCCTTCGCCTTTGTCGCCTTGCTGACTAACGGTGCGCGCGGCGGTAATGAGCGAACCATTCTGGCGGGCGTCGCCGCCTCGCAGTTATTTAACGCCATCACGGCCTATACCATCAGCACCTCCGCCAGCGCGCAGCAGGCGCGCGATGTGATGTTCTGGCTGCTGGGCAGCTTCAGCGGCGTTCGCTGGCCCGAATTCCAGTTGGTCATCGTGGTGGTGCTCGCGGGTCTGGCGGTCTGCCTGTGGTATGCCCGGGCGCTGGACGCGTTCACCTTCGGCGACGATGCCGCCGCCTCGCTGGGGATTGCTGTGCCGCGCGTGCGCCTGATCCTCTTCACCACTGCGGCGCTGATCACCGCCACCATCGTCAGCATGGCCGGCTCAATCGGCTTTGTTGGCCTGGTGGTGCCCCACGTGATGCGTTTCTTCTTTGGCCCGCTGCACCGAACGTTGCTGATCGCCAGCGCGCTGGCAGGGGCGATATTGATGGTACTGGCGGACATTGCGTCGCGCCTGCTGATTGCCCCGCAAAGTCTGCCCGTTGGGGTAGTAACCGCCCTGGTTGGGGTGCCTTTCTTTGCCGTGATTATCTACCGCTCAAGGAATAAGTGA

>IR1230_00090

ATGAGTATTTGCGCTGAAAATATCACCTGGAAGGCAGGCAAAAAGGTCATCGTCAATAATGTCTCGCTGCGGGTGCCGCGAGGCGAAACGGTCGGACTGCTGGGGCCCAACGGCTGCGGCAAATCCTCGCTGCTGCGCGTTCTGGCGGGACTGCGCCGCCCGGATGCAGGTCGCGTCACCCTCGACGGCCAGGATATCGCCCGGATGTCGAAAAAGCAGCTCGCCCGCCGCGTGGCTTTCGTCGAGCAACACGGCATGACCGAGGCCAATATGCGGGTGCGCGACGTCGTGCGCCTGGGACGCATTCCCCACCACTCTCCGTTCTCAAACTGGAGCGCTCAGGATGACGAGGCGATTGCCGCCGCGCTGCAGCGGGTAGCGATGCTGGAGAAAAGCGAACAGGGATGGTTAAGCCTCTCCGGCGGCGAGCGGCAGCGGGTGCATATCGCCCGCGCGCTGGCGCAGAGTCCGAGCGAAATCCTGCTGGATGAGCCGACCAACCATCTGGATATACACCATCAGATGCAGTTAATGCAGTTGATCAGCGAGCTGCCGGTAACCAGCATTGTGGCCATTCACGATCTTAACCATGCCGCGATGTTCTGCGATTCGCTGATCGTGATGCAGCAGGGGCAGATCCTCGCCAGCGGGACGCCGGAGGAGATTTTGTCCGAAGCGCTGCTGTGGGACGTTTTCCGGGTGAAAACCAAAATCGAGATCTCCCCTTACCACGGCAAAAAGCACATTCATTTCATCATTTAG

>IR1230_00091

ATGCCTCTTTTCTCCCTGCGCCCTGCGGCAACCCTCTGGCCTCCGGTGTTACTGGGAAGCCAGTTCGTTTTTAATATTGGCTTTTACGCGGTCGTTCCTTTCCTCGCCCTCTTCCTGCGCGACGACATGCTGCTCTCAGGCGGACTTATCGGCCTGATCCTCGGGCTGCGTACCTTCTCTCAGCAGGGCATGTTTATCCTCGGCGGGACGCTGGCCGACCGCTATGGCGCTAAGGCGATCATTCTCGCCGGCTGCGTCGTTCGCGTCGCCGGTTTTCTCCTGCTGGCGTGCGGGGCGTCGCTGTGGCCCATTATCCTCGGCGCCTGCCTGACCGGCGTCGGCGGCGCCCTGTTCTCCCCGTCGATAGAGGCCCTGCTGGCGCGGGCCGGTACCCACAGCCAGGCCAACGGGAAACGCAGTCGCGCCGAGTGGTTTGCGCTGTTTGCGGTATGCGGTGAACTGGGAGCGGTGATCGGCCCGGTGGCCGGAGGCGTGCTGAGCGGGATCGGCTTTCGGCATATCGCCCTCGCCGGCGCGGGAATATTTCTTCTGGCGCTGGCGGTGCTCTTTTTCTGTCTGCCCGCCGACGGGCACACGACAACAACGCGCAGACGAGTCCCATGGTGGACGCCTTTGCGCCAGCCGCGCTTTGTCGCGTTTATTCTCGCCTACAGCTCATGGCTGTTGAGCTACAACCAACTGTACCTCGCCCTGCCGGTGGAGATCCAACGTTCCGGCGGCCGCGAGCAGGATCTGGCGCCGCTCTTTATGCTGGCCTCGCTGTTGATTATCACCCTCCAGCTCCCTCTCGCCCGCTTCGCCCGGCGAATGGGCGCCGTGCGCATCCTGCCGGTGGGCTTTCTGCTGCTGTCGGCTTCCTTTGCCAGCGTGGCGTTATTCGCCGCCGCCCCACCCGCCGAAGGTTGGCTGCGGTTAATGCCGGCGGCTGGTTTTGTCACGCTGCTCACCCTCGGCCAGATGCTGTTAGTGCCCGCAGCCAAGGATCTGATCCCGCTGTTCGCCGAGTAG

>IR1230_00092

ATGAAAACATGGCTTCCGACATCGACCGCCGGCAGTTTACCGAAACCTTCCTGGCTGGCGCAGCCAGAGACTCTGTGGTCGCCCTGGAAACTGTCCAGCGAAGAATTACTGGCCGGCAAGCGCGACGCCTTGCGGTTATCCCTCGACGACCAGCTGCGGGCCGGGATCGATATTGTCAGCGACGGCGAGCAAACGCGGCAGCACTTTGTCACCACCTTCATCGAGCACCTCAGCGGGGTGGATTTTGCCAAACGCGAGATCGTGAAAATTCGTAACCGCTATGAGGCCAGTGTTCCAACGGTGGTGGGCGCCGTGGAGCGCCAGAAACCGGTCTTCGTGGAGGACGCTCGCTATCTACGGCAGCTCACCAGTCAGCCGATCAAATGGGCGCTTCCGGGCCCGATGACCATGATCGACACGCTGTATGACAACCACTATAAAAGCCGGGAAAAGCTGGCCTGGGAATTTGCTAAAATCCTCAATCAGGAAGCGAAAGAGTTAGAGGCTGCGGGGGTTGATATTATTCAGTTCGATGAGCCGGCCTTTAACGTCTTCTTCGATGAGGTCAACGACTGGGGGATCGCCGCCTTAGAACGAGCCACCGAAGGGCTGAAGTGTGAGACAGCGGTGCATATCTGCTACGGCTATGGCATCAAGGCCAATACCGACTGGAAAAAGACGCTGGGCTCCGAGTGGCGACAGTATGAAGAGGCTTTCCCGCAACTGCAGAAATCGTCCCTTGATATTATCTCTCTCGAGTGCCACAACTCGCGCGTGCCGATGGATCTGCTGGAGTTGATCCGCGGTAAAAAGGTGATGGTCGGGGCGATCGATGTCGCCAGCCATGCTATCGAAACCCCGGAAGAGGTGGCTGGAACCCTGCGCAAGGCGCTGGCGTTCGTCGATGCCGACAAACTCTACCCGTCCACCAACTGCGGTATGGCGCCGCTGCCGCGCCATGTCGCGACCGGCAAACTGCACGCCCTGAGCGCCGGCGCCGAGATTATTCGTCGCGAGCTGGCGGCAAAGTAA

>IR1230_00093

ATGAATAATAAATTTACCTATACGATTAAGCGCACCCGGTTTGATGAAAACTATAACCCTGCGGAAAATACGCGTATTACCACCAACTTCGCTAATTTAGCCAGAGGGGTGAACCGTGAGGAAAATCTGCGCAATACGTTAATCATGATGAATAACCGTTTTAATAGCCTGGCCCACTGGGATAATCCCCATAACGATCGCTATGCGGTGGAACTGGATATTATTTCTGTCGAGATGAATATTGCTCAGGATAGCGCCAGCTTCCCGGTGATTGAGATCCTGCAAACGCATATTGTCGATAAAAAGAGCGGGGAGCGTCATGCCGGTATCGTCGGCAATAACTTCTCCTCCTATGTGCGGGATTACGATTTTAGCGTACTGCTTCTGGAACATAATAAGGACCAGAGTCGCTTCAGCGTACCGGAAAACTTTGGCGAGCTGCACGGCAATATCTTTAAGGATTTTGTGCAATCCAGCGCCTGGCGCGCCAACTTCAGCAAGGCGCCGGTGATTTGCCTGAGCGTCTCCAGTAAAGATGTTTACCATCGCACCGGTAACGAACATCCGGTCCTCGGCATTGAATATGCTCAGGAAGGCGTCTCGCTGACCGAGCGCTATTTCAGCAAAATGGGTCTGCAGGTACGCTATTTTATGCCAAAAAACAGCGTCGCGCCGTTGGCCTTTTATTTTACCGGCGATCTGCTCAGCGATTACACCAGCCTGGAACTGATCGCCACCATCAGCACGATGGAGACGTTTCAGAAAATATATCGCCCGGAGATCTACAATGCCAACTCCCCGGCGGGGCAATACTATCAGCCCAATCTCAGCCACCTGGATCATTCATTAACAAAAATCGTCTATGACCGGGAAGAGCGCAGCCTGTTGGCGATTGAGCAGGGGAAATTTACACAACAACATTTTATCAACCCGCACAAGACGTTGCTTGAGCAATGGTCTGCTAATTTCGCGCTTTGCTAA

>IR1230_00094

ATGAGAGCGAGTGTCATTGCGTTCGGGAGCCTCTTAATTCAGCAGTACGCTCATAATCAGCAGTACGGCCATGCACATCGACATAATACTGATATCAACAATCATTCTGTACGACAAAAGCTCCAGCTAAACGGGTACGGCGGGCAGGTAAAAGCCCATCTTATAGTGGTTAAAGAGTGTACCCTGCCTACATCCATTGAACAAACGTTTATTGACTAA

>IR1230_00095

ATGAAAAAAGCTCTGCTTGCCCTGTTCGCTATCGCCACCGTTACCGCCGCCAGCGCCTTTGCGGCTGATAAAACCGACGTCCCGGCGCCTGTCGTCGATAACGGAGTCCGCCCGCCGATGGAGAGGGATCGTGGCCCCCTGCCGCCGTTCCCGCCGCAGCTTCCGCCGCACAGACCGGTGCTGTTTTCAGCCACGGTAGCAACCGATACGCCAGCGGAAACCATTAACAAACTGACCGCGCTGATCCCCGCCGGCCAGGCGAAACACTACGAAGTGCACGTTGAGGTGGTGCCGGTTGGCGAGGGAACCGACAGACAATAG

>IR1230_00096

ATGGGTATTCTTTCCTGGATCATTTTTGGACTTATTGCCGGTATTTTAGCCAAGTGGATTATGCCGGGAAAAGATGGCGGCGGATTTATCGTGACCGTTATTTTGGGGATCATCGGCGCCGTGGTCGGCGGCTGGATCAGTACCCTGTTTGGTTTTGGCAAGGTCGATGGGTTTAATTTCGGCAGCTTCGTGGTAGCGGTTATCGGTGCGCTGGTGGTACTGTTTATCTACCGGAAAGTGCGTAGCTAA

>IR1230_00097

ATGATTTGGAAACGTCAGGCCACGCTGGAGCAGCTCAACCGGCTCGGTGAGGGGAACATGGTAGGGCTGCTCGATATTCGCTTTGAGACCGTTACCGACGATACGCTGGAGGCCACTATGCCGGTCGATAGCCGCACACAGCAGCCGTTTGGCCTGCTGCACGGCGGAGCGTCGGTGGTGCTGGCGGAGACCCTGGGGTCGGTGGCAGGCTATCTGTGCAGCGAAGGCGAACAAAAGGTGGTGGGGCTGGAGGTTAACGCCAACCACATCCGTTCGGCGCGCGGGGGCCGGGTGCGCGGCGTCTGCAAAGCGCTGCACGTCGGGACGCGCCATCAGGTCTGGCAAATTGAAATCTTCGACGAACAGTCGCGGTTATGTTGTTCATCACGACTAACCACCGCGGTAATATAA

>IR1230_00098

ATGATCCCACAAATTTCTCAGGCGCCAGGGGTAGTTCAACTGGTGCTGAATTTTTTGCAGGTACTGGAGCAACAGGGTTTTACCGGCGATACCGCCACCAGCTATGCCGACAGGCTAACGATGGCCACCGACAACAGCGTCTATCAGCTGCTGCCGGATGCGGTCATTTTTCCACGATCCACGGCGGACGTGGCGCTGCTGGCGCGCGTCGCCGCCGAACCGCGTTTTAAATCGCTGATTTTCACCCCGCGTGGCGGCGGCACCGGCACTAACGGCCAGGCGCTGAACGGCGGGATTATCGTTGATATGTCCCGCTATATGAATCGCATCATCGAGATTAACCCTGACGAGGGCTGGGTGCGCGTTGAAGCCGGGGTCATCAAAGATCAGCTGAACCAGTTTCTTAAACCCTATGGTTACTTTTTTGCGCCTGAGCTATCCACCAGCAACCGCGCCACGCTGGGGGGGATGATTAACACCGACGCGTCCGGCCAGGGATCGCTGGTGTACGGCAAAACGTCGGATCACGTGCTCGGCCTGCGGGCGGTGCTGATGGGCGGGGATATCCTCGATACCCAGGCGGTGCCGGTGGCGCTGGCGGAGACGCTGGGCAATACCCCCTCGACGGTTGGCCGGATCTACAACACGGTCTACCAGCGCTGCAAGGCGCAGCGTGACCTGATCATTGATAAGTTTCCCAAACTCAACCGCTTCCTCACCGGATACGATCTGCGTCACGTCTTTAACGATGAGATGAGCGAGTTCGATCTGACCCGTATTTTAACCGGTTCGGAAGGGACGCTGGCGTTTATTACCGAAGCGCGGCTGGATATCACCCGCCTGCCGAAGGTGCGCCGTCTGGTCAACGTCAAATATGACTCCTTCGACTCTGCGCTGCGCAATGCGCCCTTTATGGTCGAAGCGAAAGCCCTGTCGGTGGAGACCGTCGATTCTAAAGTTCTCAATCTGGCGCGCGAGGATATCGTCTGGCACTCGGTAAGCGAACTGATCACCGACGTACCGGATAAAGAGATGCTCGGGCTCAATATCGTCGAATTCGCCGGCGATGACGCGGCGCTGATTGACCAGCAGGTTACTACCCTGTGCCAGCGGCTGGACGAGCTGATGGCCGCCAGCGAGGCGGGCGTCATTGGCTGGCAGGTCTGTCACGATCTTGAAGGCGTGGAGCGCATCTATGCCATGCGCAAGAAGGCCGTCGGCCTGTTGGGCAATGCCAAAGGCGCCGCCAAGCCTATTCCTTTCGCCGAGGACACCTGCGTACCGCCCGAACATCTGGCGGATTACATCGTCGAGTTCCGCGCGCTGCTCGACAGCCACGGCCTGAGCTATGGCATGTTTGGCCACGTTGATGCCGGGGTGCTGCACGTGCGCCCGGCGCTGGACATGTGCGATCCGCAGCAGGAGGTGCTGATGAAGCAGATCTCCGACGATGTGGTGGCGCTGACGGCGAAGTATGGCGGCCTGCTGTGGGGCGAACATGGCAAAGGGTTCCGCGCGGAGTACAGCCCGGCCTTCTTTGGCGAGACGTTGTATGCCGAGCTGCGCAAGATCAAAGCGGTCTTCGATCCCGATAACCGCCTGAATCCGGGTAAAATCTGTCCTCCGGAAGGTATCGACGCGCCGATGATGAAAGTCGATGCGGCGAAGCGCGGCACCTGGGATCGGCAGATCCCCATCGCGGTGCGCAGCAGCTGGCGTGGGGCGATGGAGTGTAATGGCAACGGGCTGTGCTTCAACTTTGACGTCAAGAGCCCGATGTGTCCGTCGATGAAAGTCAGCAACCAGCGCATCCACTCGCCGAAAGGGCGGGCGACGCTGGTGCGGGAATGGCTGCGCCTGCTGGCCGACCGCGGCGTCGATCCCAACCAGCTGGAGAAGGCGCTGCCGGAGCAGGGCGTCAGCCTGCGCTCGCTGGTCGCGCGCACGCGCAACAGCTGGCATGCGCGCAAAGGCGAATATGACTTCTCGCATGAGGTCAAAGAGGCGATGTCCGGCTGTCTGGCCTGTAAAGCCTGTTCGACCCAGTGCCCGATCAAAATCGATGTTCCCGAGTTCCGCTCGCGCTTCCTGCAGCTGTACCACAGCCGCTATCTGCGGCCGGTGCGCGATCATCTGGTGGCCTCGGTCGAATCTTATGCGCCGCTGATGGCGCAGGCGCCGAAGACCTTTAACTTTTTTATTAACCAGCCTTGGCTGAAAAAGCTGTCGGAGAAGCATATCGGGATGGTCGATCTGCCGCTGCTCTCGGCGCCGTCGCTGAAGCAGCAGATGGCCGGCCACCGCTCCGCCAATATGACCCTTGAGCAGCTCGAAGCGCTGAGCGCCGAGCAGAAAGCGAAGATGGTGCTGGTGGTGCAGGATCCCTTCACCAGCTACTACGACGCCCAGGTGGTGGCCGACTTTATCCGTCTGGTCGAGGCCTTAGGCTATCAGCCGGTGCTCCTGCCGTTCTCGCCGAACGGCAAGGCGCAGCATATCAAAGGCTTCCTCACCCGCTTCGCGCGCACCGCGCAGAAGACGGCGGACTTCCTCAACCGCGTGGCGCAGCTGGGCATGCCGCTGGTGGGCGTCGATCCGGCGCTGGTGCTCTGCTACCGCGATGAGTACAAGCAGACGCTCGGCGACAAGCGCGGCGATTTCCAGGTGCTGTTAGTCCATGAGTGGTTGCCGAAGGCCCTAACCAGCGACGCCCGTCCCGATCTGGGCGGTGAGCCCTGGTACCTGTTCGGTCACTGTACCGAAGTGACCGCGCTACCGGCGGCGACAAAGCAGTGGGCAGATATTTTTGCCCACTTCGGCGCGAAGCTGGAAAACGTCAGCGTTGGGTGTTGCGGGATGGCTGGCACCTATGGTCATGAAGTGAAAAACCACGCCAACTCGTTGGCCATTTACGCGCTCTCCTGGCAGCAGGCGATGCAGCGGCTGCCGCGAAACCGCTGTCTGGTGACGGGCTACTCCTGCCGCAGTCAGGTGAAGCGTATTGAAGGCAGCGGCGTTCGCCACCCGCTGCAGGCGTTACTGGAGATAATCGGATGA

>IR1230_00099

ATGATTAATCCCCATCAGCCTCGGGACATTCCGCAAATACTGCTGTCGGTGCTGTTCCTGGCCCTGATTATCATATCCTGCCTGTGGGTTGTGCAGCCGTTTATTCTGTCCTTCGCCTGGGCCGGTACGGTGGTGATCGCCACCTGGCCGGTGCTGCTGCGTCTGCAGCGTGTGCTGTTCGGCAAACGACTGCTGGCGGTGCTGGCGATGACCCTGTTGCTGTTTCTGCTATTCGTTATTCCTATCGCTCTGCTGGTCAACAGCCTGGTGGATAACAGCGTTCCGCTGATCAAACTCATCAGCAGCGGAAATGTGACGCTGCCGGATTTCGCCTGGCTCAACAGCGTACCGCTGGTCGGCGATAAACTGTACTCCGCCTGGCACGGTCTGCTGGATATGGGCGGCTCGGCGATTATGGCCAAGGTTCGTCCCTATATTGGCACCACCACCAGCTGGTTTGTCGGTCAGGCGGCGCACATCGGCAAGCTGCTGGTCTACTGCGGTTTGATGCTGCTGTTCAGCGCGCTGCTCTACTGGCGCGGCGAGCAGGTGGCCTATGGTTTCCGCTACTTCGCCACGCGCCTCGCGGCCAAACGCGGCGACGCCGCGGTGCTGCTGGCCGGCCAGGCCGTGCGCGCCGTGGCGCTGGGCGTGGTGGTCACTGCCCTCACTCAGGCGGTACTGGGCGGGATTGGCCTTGCCGTCTCCGGCGTGCCCTACGCCGCTCTGCTGACCGTGGTGATGATCTTTACCTGTCTGGTTCAGCTCGGCCCTTTACTGGTTTTGGTGCCGTCGATTATCTGGCTTTACTGGAGCGGGGACACCACCTGGGGCACCGTGCTGCTGGTCTGGAGCTGCGTAGTCGGCACCATGGACAACGTTATTCGTCCGGTGCTCATCCGCATGGGCGCCGACCTGCCAATGATCCTTATCCTCACCGGCGTCATTGGCGGCCTGATCGCCTTCGGCATGATCGGCCTGTTTATCGGTCCGGTGCTGCTCGCCGTCTCCTGGCGACTGTATGACGCCTGGGTGCATGAGGTGCCGCCGCCGCCGAAAGACCCTGACGTCGTCCTTGAAGAGCTCAGTGAGCTGGAGGCCGGTCGCAAGTAA

>IR1230_00100

ATGTCCAACAATGGCTCGTCACCGCTGGTGCTTTGGTATAACCAACTCGGCATGAATGATGTAGACAGAGTTGGGGGCAAAAATGCCTCCCTTGGTGAAATGATTACGAATCTGTCCGGTATGGGTGTTTCCGTACCTAACGGGTTTGCCACCACCGCTGATGCTTTCAATCAGTTCCTGGACCAAAGCGGTGTTAACCAGCGCATCTATGCGCTGCTGGATGAAACCGACATTGACGACGTTTCCGCATTAGCGAAAGCGGGCGCGCAGATCCGACAGTGGATCATCGATACCCCCTTCCAGAGCGAACTGGAAAACGCGATTCGCGATGCTTATGACCTGCTGTCCGCCGATGACGCCGAGGCCTCATTCGCCGTGCGCTCTTCCGCCACCGCGGAAGATATGCCGGACGCCTCCTTCGCCGGACAGCAAGAGACATTCCTCAACGTGCAGGGCTTTGACGCCGTGCTCGTTGCCGTGAAGCACGTGTTTGCTTCGCTGTTTAACGATCGCGCAATCTCCTATCGCGTGCATCAGGGTTACGATCACCGCGGCGTGGCGCTCTCCGCCGGGGTGCAGAGGATGGTCCGCTCCGATCTGGCCTCCTCCGGCGTCATGTTCTCCATCGATACCGAATCCGGCTTTGACCAGGTGGTGTTTATCACCTCGGCGTGGGGTTTGGGCGAGATGGTGGTGCAGGGCGCGGTGAACCCGGACGAATTCTACGTCCACAAACCGACGCTGGCCGCTGGCCGTCCGGCGATCGTGCGCCGCACCATGGGGTCGAAAAAGATCCGTATGGTTTACGCGCCGACCCAGGAGCACGGTAAACAGGTGCGTATCGAAGATGTGCCGCAGGCGCAGCGCGATATTTTCTCGTTAAGCAACGAGGAGGTGCAGGAGCTGGCGAAGCAGGCGGTGCAGATCGAGAAGCACTACGGCCGTCCGATGGATATCGAGTGGGCGAAAGATGGCCACACTGGTAAGCTATTTATCGTCCAGGCGCGTCCTGAAACCGTGCGTTCCCGCGGCCAGGTGATGGAGCGTTATACCCTCCACGCTCAGGGGCAGATCATCGCCGAAGGGCGCGCCATCGGCCACCGCATTGGCGCCGGTCCGGTCAAGGTGATCCACGATATCAGCGAGATGAATCGCATTGAGCCAGGCGACGTACTGGTTACCGACATGACCGACCCGGACTGGGAACCGATCATGAAAAAAGCCTCGGCGATTGTCACCAACCGCGGCGGGCGTACCTGTCACGCGGCGATTATCGCCCGTGAGCTGGGGATCCCGGCGGTGGTCGGCTGCGGCGATGCTACCGATCGCATTCAGGAAAATCAGAATGTCACCGTCTCCTGCGCCGAAGGCGACACCGGCTATGTCTACGCCGAGCTGCTCGACTTCAGCGTCAAGAGCTCCAGCGTCGGCGACATGCCGGATCTGCCGCTGAAGGTGATGATGAACGTCGGCAACCCGGATCGCGCGTTCGACTTCGCCTGCCTGCCGAACGAAGGCGTCGGTCTGGCGCGGCTGGAATTTATCATCAACCGTATGATTGGCGTTCATCCGCGCGCGCTGCTGGAGTTTGACGACCAGGAGCCGGGCCTGCAGAACGAAATCCGCGAGCTGATGAAAGGCTACGATTCGCCGAGAGAGTTCTACGTTGGCCGCCTGACCGAAGGGATCGCCACCCTCGGCGCCGCTTTCTATCCGAAACGGGTGATCGTGCGTCTGTCGGACTTCAAATCGAACGAATACGCCAACCTGGTGGGCGGCGAGCGCTATGAGCCGGAAGAAGAGAACCCGATGCTCGGCTTCCGCGGCGCCGGACGCTACGTTTCCGAAAGCTTCCGCGACTGTTTCGCCCTTGAATGCGAAGCGATGAAGCGCGTGCGTAACGACATGGGACTGACCAACGTTGAAGTGATGGTCCCGTTCGTCCGTACCGTCGCCCAGGCGAAAGCGGTGGTCGAAGAGCTGGAGCGTCAGGGGCTGAAGCGCGGCGAGAACGGGCTGAAGATCATTATGATGTGCGAGATCCCGTCGAACGCCTTGCTGGCGGAGCAGTTCCTCGAGTACTTCGACGGCTTCTCCATCGGTTCGAACGACATGACCCAACTGGCGCTCGGTCTGGACCGCGATTCCGGTGTGGTTTCTGAGCTGTTTGATGAACGCAATGACGCGGTGAAAGCCCTGCTGTCGATGGCCATCCGCGCGGCGAAGAAACAGGGTAAATACGTCGGCATCTGCGGCCAGGGGCCGTCTGACCATGAGGATTTCGCCGCCTGGCTGATGGAAGAGGGGATTGATAGCTTGTCCCTGAACCCGGATACCGTGGTACAAACCTGGTTAGGTCTGGCGGAACTGAAAAAATAA

>IR1230_00101

ATGGAAAGTGCTGTAGATCGCCACGTATTTTATATTTCTGACGGTACCGCCATCACGGCGGAGGTGCTGGGGCATGCCGTGATGTCGCAATTCCCGGTCGCCATCAGCAGCGTAACCCTGCCGTTTGTGGAAAACATCAGCCGCGCGCGGGCGGTAAAGGAGCAGATCGACGCCATCTATCAGCAAACGGGTATTCGCCCGCTGGTGTTCTACTCCATCGTCATCCCGGAAATACGCGACATTATTCTGCAAAGTGAAGGCTTTTGTCAGGATATCGTGCAGGCGCTGGTGGCCCCGCTGCAGCAGGAGCTCAACCTCGACCCGACGCCGGTGGCCCACCGTACCCACGGCCTCAACCCCGGCAACCTGATTAAATACGATGCGCGTATCGCCGCTATCGATTATACCCTTGCCCATGACGATGGCATCTCGCTGCGCAACCTTGACCAGGCGCAGGTGATCCTCCTCGGCGTCTCGCGCTGCGGCAAAACACCCACCAGTCTGTATCTCGCCATGCAGTACGGTATTCGCGCCGCCAACTATCCGTTTATCGCCGACGATATGGACAATCTGGTTCTGCCGGCCTCGCTGAAACCCCTGCAGCATAAGATGTTCGGCCTGACCATCAATCCGGAGCGTCTGGCGGCGATCCGCGAGGAGCGCCGGGAGAACAGCCGCTACGCCTCCCTGCGCCAGTGTCGGATGGAAGTGACCGAAGTGGAGGCCCTGTATCGCAAGAATAAAATCCCCTGCCTGAACAGCACCAACTATTCCGTTGAGGAAATAGCGACAAAAATCATGGACATCATGGGGCTGAATCGCAGAATGTACTAA

>IR1230_00102

ATGAATAAAACAGACGAATTGCGTACCGCGCGCATTGAGAGCCTGGTAACGCCCGCGGAGCTGGCCCAGCGCCATCCCGTGACGGCCGACGTGGCTGCCCACGTCTCGGCCTCCCGCCGCCGAATTGAAAAAATCCTCAACGGCGAAGACCGCCGACTGCTGGTGATTATCGGCCCCTGCTCCATTCACGACACCGACGCGGCGCTGGAGTATGCCCGCCGCCTGCAGGGCATGCGCGAGCGCTACCAGCCGCAGCTGGAGATCGTGATGCGCACCTATTTTGAAAAACCGCGCACCGTGGTGGGCTGGAAAGGCTTAATCTCCGATCCCGACCTCAACGGCAGCTACCGGGTCAACCACGGAATTGAGCTGGCGCGGCGCCTGCTGCTGCAGGTTAATGAGCTGGGGGTACCGACGGCCACCGAGTTTCTCGATATGGTGACCGGTCAGTTTATCGCCGATCTCATCAGCTGGGGAGCCATCGGGGCGCGCACCACAGAAAGCCAGATCCACCGTGAAATGGCCTCAGCGCTCTCCTGCCCGGTTGGATTTAAAAACGGCACCGACGGCAATACGCGCATCGCGGTGGACGCCATCCGCGCCTCCCGCGCCAGCCATATGTTCCTCTCTCCGGACAAGCAGGGTCAGATGACGATTTACCAGACCAGCGGCAATCCGTATGGGCATATTATTATGCGCGGCGGCAAACGGCCAAACTATCACGCCGAGGATATCGCCGCCGCCGGCGAGGCGCTGCGTGAATACGATCTGCCGGAACAGCTGGTCGTTGATTTCAGCCATGGCAACTGCCAGAAGCAGCACCGCCGTCAGCTGGAGGTGTGCGCCGATATCTGTCAGCAAATTCGCGCGGGCTCCACCGCCATCGCCGGGATTATGGCCGAAAGCTTCCTGCAGGAAGGCACTCAGAAAGTGGTCCCCGGCCAGCCGTTGACCTGGGGCCAGTCGATCACCGACCCCTGCCTGAGCTGGGAAGACAGCGAAAGATTATTGAGCGAGCTGGCTGCGGCCACCGCCACCCGCCTGTAA

>IR1230_00103

ATGATTATCATTGTTGCCTTGATTTTTATTCTGAGAGCTATGACGCGTATGGATAACACTGCCACAGCTGAAAAACCAAAAGACAATGCCCCTTATCCCGTGGCGACGGACAGAGAGATCAGCAGTCGGGTTCTGTTAGGCAGCGAAGGACGAGTAGTGATTGAGCATGGCGGCCAGCGCTATCTGCTGCGCCAGACCCACGCCGGGAAGTTAATTCTGACCAAGTAA

>IR1230_00104

ATGACCCTTTCTTTTACCACCCACTGGCGGGATGAACTGCCGGACTTTTACACTTCCCTGTCACCAACGCCGTTGGATAACGCCCGACTCATCTGGCGCAACGCCCCGCTGGCGCAACAGCTGGGGGTGCCCGACGCCCTGTTTGCGCCTGAAAGCGGGGCCGGTGTCTGGGGCGGCGAAGCGCTGCTTCCGGGCATGTCGCCGCTGGCGCAGGTATACAGCGGCCATCAGTTTGGCGCCTGGGCCGGCCAGCTGGGCGACGGACGCGGGATCCTGCTCGGCGAGCAGCAGCTGGCGGATGGCCGCCGTTACGACTGGCATCTGAAAGGCGCCGGCCTGACTCCTTATTCGCGGATGGGGGACGGCCGCGCCGTGCTGCGCTCGACGATCCGGGAAAGTCTGGCGTCGGAGGCGATGCATGCTCTGGGGATCCCGACGACGCGCGCCCTGGCGATGGTGACCAGCGATACCCCGGTCTACCGCGAGCGCGTGGAGCCCGGCGCGATGCTGATGCGGGTGGCCGAGAGCCATGTCCGCTTCGGCCATTTCGAACATTTTTACTATCGCCGTGAGCCGCAGAAGGTCCAGCAACTGGCCGACTATGTTATTCGCCACCACTGGCCGCAGCTGCAGGATGAGGCGGATAAGTACCTGTTATGGTTCCGTGACATTGTCATGCGTACCGCGCAGACTATCGCCAGCTGGCAAACCGTGGGCTTTGCCCATGGGGTGATGAATACCGACAACATGTCGATTCTCGGTCTGACCATCGACTACGGCCCGTATGGCTTCCTTGACGATTTTCAACCGGACTTCATCTGCAACCACTCCGATTATCAGGGGCGCTACAGTTTTGAAAACCAGCCGGCGGTGGGGCTGTGGAATCTGCAGCGTCTCGCCCAATCGCTGTCGCCGTTTATCAGCGCTGAAGCGTTAAACGCGGCGCTGGATGAGTATCAGCACGCCTTGCTGACCGCTTATGGCCAGCGGATGCGCGATAAACTCGGCCTGTTCAGCCAGCAGAAAGGGGATAACGACCTGCTGGACGGGCTGTTTGCCCTGATGATCCGCGAGAAGAGCGACTATACCCGGACGTTCCGCCTGCTGAGCCACAGCGAACAGCTCAGCGCCGCTTCGCCGCTGCGCGATGAGTTTATCGACCGCGCGGCCTTTGACAGCTGGTTTGCCGGGTATCGTGCGCGGCTGCGTGATGAACAGGTGGACGACGCGCAGCGCCAGCAGAGGATGCAGGGCGTGAATCCGGCGCTGGTGTTACGTAACTGGCTGGCGCAGCGGGCGATCGAGCAGGCTGAGGCGGGCGATATGGGCGAGCTGGAGCGCTTGCATGCCGCGCTGGCAGACCCCTTTACCGATCGCGAGGACGACTACGTCCGCCGCCCGCCGGACTGGGGGAAACGTCTGGAAGTCAGCTGCTCGAGCTAA

>IR1230_00105

ATGCGCTTCTGGTTCATGTTGGCGGCGGCGTTAATTTTAGCCGGCTGCAGCAGCCATCGGGCACCGCCGCCGAATCCTCGGCTGGCAGACTCCATTACGGTGGTGGCGAATCTGAACGAGCAGCTGCGCAGCTGGCGCGGCGCGCCGTATCGCTACGGCGGCATGACGCCGCGCGGCGTTGACTGCTCCGGCTTCGTGGTGCGCACGTTTCGCGATAAATTTGCCCTCCAGCTGCCGCGTGAAACCCGCGAGCAGGCGGAGATCGGCACCCGCATCGATAAGCGCGATCTGCTGCCCGGCGATCTGGTGTTCTTCAAAACCGGCTCAGGGGAGAGCGGTTTGCACGTTGGCATCTACGATACCGACAACCAGTTTATTCACGCCTCCACCAGCCAGGGCGTCACCCGTTCCTCGCTGGATAACGTCTACTGGAACAAAAAGTTCTGGCAGGCGCGGCGAATCTGA

>IR1230_00106

ATGTCGTTTCTGATGCAGCTACAGGATGTAGAAGCGGCGGGGCGCCTGGCCCCGTTTTCTGCGGCCTTTCGCGCCGGGGAGATTGTCCATCTGGTGGGGCCGAACGGCGCAGGGAAGAGCACGCTGCTGACCCGCATGGCGGGGCTGTCCGACGGCCCCGGCACGGTTCATTTTAACGGCCGGCTGCTTGACGAGTGGCCGGCGAGGGAGCTGGCGCGCCGTCGCGGATATCTGTGTCAACACCAGACCCCGCCGTTCGCCATGCCGGTATGGCACTACCTGGCGCTGCATATGCAGCAGCAGGGTGATAGCGCCCGTCTGAGCGATATCGCGGCCAGGCTTGGTCTGGACAATAAGCTGGGCCGTCCGGTCAATCAGCTGTCGGGCGGCGAGTGGCAGCGCGTGCGGCTGGCGGCAGTGCTCCTGCAGATCGATCCCTTGAGTAATTCCGCGGGTCAGCTGCTGCTGCTGGATGAGCCGATGAACAGTCTGGATGTGGCCCAGCAGGCGGCGCTGGATCGGCTGTTACACGAGCTGAGCGCGGCGGGGATTGCGGTGGTGATGAGCAGCCACGATCTCAACCATACCCTGCGCCACGCCGGTCAGAGCTGGCTGCTCTGCCAGGGGGAAGCCATCGCCTGCGGGGAGACCGCCGAGGTGCTGAATGAGGAAAATTTAACCGCCGCCTATGCGATCCCGTTCCAGCGCGTTGAGGTGGCCGGTCATATTATGCTCATCGCGTCACAGTAG

>IR1230_00107

ATGCAACATGACATTCTGAATACCGAAGTGACGACCATTGATGGCGAGAAAACCACCCTGGCATCCTTTGCCGGCAAGGTGCTGCTGATCGTCAATGTGGCCTCGAAATGCGGCCTGACGCCGCAGTATGAGCAACTCGAGGATCTGCAAAAGCAGTTTGCCGCCGAGGGCTTCAGCGTGCTGGGGTTCCCCTGCAACCAGTTCCTCGGCCAGGAGCCGGGCAGTGAAGAGGAGATTAAGACCTTCTGCAGCACCACCTACGGCGTCACCTTCCCGCTGTTTAGCAAAATTGACGTCAACGGTGAACATCGCGCGCCGCTCTATCAAAAACTGATTGCCGCCGCGCCGAAAGCGGTGGCCCCGGAAGGCAGCGGTTTTTATGAGCGGATGGCCAGCAAAGGCCGGGCGCCGCTGTATGTGGACGACATTCTGTGGAACTTCGAAAAATTCCTCATCGATCGCCAGGGCAACGTCATTCAGCGCTTCTCGCCGGATATGACGCCGGATGATCCGCAGCTGGTGGCGGCGATTAAAGGAGCGCTGGCGCAATAA

>IR1230_00108

ATGAAGAAGTTGTTTAGCCTGGCGTTAATCGCCACCTCTGTCGCCCTGCTGAGCGCCTGCTCCCCGGATGAAGATAATAAAGTAAAAGTCGCTATCAATACCGGGCCGGACGAAGCCATCTGGAAAGTGGTGGAGCAGGTGGCGAAGGATAAATACCACCTCGACGTCGAAGTGGTCTCCTTCAATGATTATGTGCTGCCCAACGAAGCGCTGAACAACAAAGATGTCGATGCCAACGCCTTCCAGACCCTGCCGTACCTTGAGGCGCAGTCGAAAGAACGCGGCTATAAATTTGCGGTGGTCGGTAAAACCTTTGTCTTCCCTATCGCCGCCTATTCTCACCGGATCAAAAATATCAGCGAACTGCCCGAAGGGGCGACGGTGACCATCTCTAATGAAACCACCACCCTGGGCCGCAGCCTGCTGCTGCTTCAGGCGCAGGGGTTACTCAAGCTGAAACCGGGCGTCGGCTATTTACCCACCACGCTGGATATCATCGACAATCCGAAACAGCTGAAAATTGTTGAAGTGGATACCCCGCAGCTGACCCGTACCCTTGACGATCCGAACGTCAGCCTGTCAATCATTAACACCAACTTCTCCGCCCAGGCGGGCCTGTCGGCCGCCCGCGACGGTCTGTTTATGGAGGGGCCGGATTCCCCATACGTGAATGCGATAGTCGCCCGCGAGGATAATAAGGACAGCAAGAAAATTCAGGAATTAAAAGCCGCCTTCCAGACCAGCGAAGTCGCGGAAAAAGCGAAGGAAGTCTACAAAGGCGACGCGATCAAAGGCTGGTAA

>IR1230_00109

ATGCTGACTCTCGCCCATCTTCAACAGCGCCGCAGCCGGCGTTGGCTCTTCGGATTAACGCTGTTGCTGCTGGTCACCCTGCTGATCAGCCTGTGCGCGGGGGAGCAATGGATCCCGCCCGGCGAGTGGCTCAGCGCCAAAGGTCAGCTGTTTATCTGGCAGATCCGCCTGCCGCGAACCCTGGCGGTGCTGCTGGTGGGGGCGGCGCTGGCGCTCTCCGGGGCGATTATGCAGGCGTTGTTTGAAAATCCTCTGGCGGAGCCGGGGCTGCTGGGGGTCTCCAACGGCGCGGGCGTCGGGCTGATTGCCGCCGTCCTGCTGGGCAAAGGAGTCTTGCCCGGCTGGGCGCTCGGCCTGTGTGCCATCTTTGGCGCGCTGCTGATCACTTTTATTCTGCTGCGTTTTGCCCGCCGCCATCTGTCTACCAGCCGTCTGCTGCTGGCGGGGGTGGCGCTGGGGATCATCTGCAGCGCCCTGATGACCTGGGCAGTCTACTTCTCCACCTCTTTTGACCTGCGTCAGCTAATGTACTGGATGATGGGCGGGTTTGGCGGCGTCGACTGGCAACAGCTGTGGCTGATGATAGCCCTCCTGCCGGTGCTCTGCTGGGCCTGTCTGCAGTCGCAACCCCTGAATTTACTGGCGCTGGGCGAAGTCTCCGCTCGTCAGCTTGGGCTGCCGCTGTGGCTGTGGCGCAAATTACTGGTGGTGGCGACCGGCTGGATGGTCGGCGTTAGCGTGGCGCTGGCGGGGGCGATTGGCTTTATCGGTCTGGTGATCCCGCATATTCTGCGGCTATGCGGCTTGAGCGATCACCGGGTGTTGCTCCCGGCCTGTATGCTGGCCGGGGCAAGCGCCTTGCTGGGGGCGGATATCATTGCCCGGCTGGCGCTTTCCGCCGCAGAGCTGCCGATTGGCGTGGTGACGGCGACCCTGGGCGCGCCGGTCTTTATCTGGTTGCTGCTGCGTTCGCGGGGGCGGGGATAG

>IR1230_00110

ATGAGTGACTCAACGCTCACAGGCAATGCGCCTGTCAGACAAAATATCACGCGGAAAAACGTTATTATTGGTCTGTTATTGCTGCTGTTCGTGTTGATCGCGCTGTGGTGCCACGGTCGACCAGGCAGCGAGCTTGGTCTGCTGGGATTTACGCCGCTGGTGGCGCTAGCGATATTATCGCTGATCGGCGTTGATATTGTGCTGGCGGTCATCTCCTCGATTATTATCGCCATGATAATGACCTCGACCGGCCTGCCGGAAATGGGCACGATGCTGGCGAAATCCACCGGGTCATTTATTGCCACCGTTGGGCTTATCATTATGCTCGGCGCCGGCGTGGGGGAAGTGGCCACCCGCACCGGCGCAGCGGTTGAGCTGGTAAAATTTGTCGTCCATCGCATCGGGTTGTCGAGTCAGACGCGGGTGAAATTCGGCATTGTGGCGTCATCGATTTTAATTTGCGGTTCGCTGGGCACTATGGCCGGTGGCAATGCCATTATCGTGGCAGTAATCATTCCGGTGGCGGCAGCGGTACGCTTGACGCCGCCAACGGTCGCCGCGCTGATGATGACCGCCGGTTCCGTTGGGCTGTTTACCGGGCCTTTCACCCCCAGTACCGTGACGATCCTCAGCCTCGGTGGGTTGAGCTATCCGGACTATCTGCTGTACGTTGGTCTGCCGATGAGCGCGGTCACGCTGCTGGCCGGCTGGGTAATGGCGGGACGGATCCAGAAGATGACCGAAGGCAAACTGCGCTATGACGTCGATCTCGCGGAAAAGCCGCAAGAGGATCTCAGCGCCGCGCAGCAGCGTCGGCGTAAATTAAGCGCGCTGGCCTTCGCGGCGACCATCATCGTGATGGCGATCGTTGGCGTGGTGATTAAAGCCGGCTTCAGCTTCGCGATAATCGTGATGCTGCTGGTGGCCCTGATGACCGGCCTGGTAGGCGGCCTGCGACCGACGCAGATCCTTCAGGCACTGTATCACGGCTGCGGCCGTCTGGTGTGGATGTTTATTCTCTACTGGCTGTACAACCCGATCCTTGAGCTGATGGATGGCCTGCACGCCTATCAGGGGCTGCTGGAGTATACCCAACCGCTGCTGGAGGGGATCTCCCCGGCCTGGCTGTGCTTTAGCATCTTCGCGTTCAATATTATCGGCCATGTGCCCGGTGCGGCGGTGGCCCAGATGACCTTTACTCATAAAATCTTTGGCCCGATGCTGATGGCCGCCGGCGTGCCGCCCCAGGGGACCACCGCCGTGCTGCTGGCCTCATCCCAGGTGGACTGGTTCGGGCCGTTTCCCTCATCAGATATGTTCGGCCAGATGGGGCTGGCGCAGTCTACTCATCTGAAATATATGCTCTATAACGGCTGGGCAATCGTGGTGGCGAACATCATTCTCTTTGCGCTGCTGTTTCAGATTCTGGTGTGA

>IR1230_00111

ATGAAACTGCGTCATCTGGAAATCTTCTATGCCGTCATGACCTGCGGCTCTTTATCGCGGGCGGCGGAAGCGCTCAATATCTCCCAGCCCGCGGCCAGCAAAGCGTTAAAAAGCGCCGAAATGAAGCTGGGTTTTACCCTGTTTCAGCGGGTGCGCGGCAAGCTGCTGCCGACCAGCGAGGCGATCACACTTTTCGAAAAAGCGCAGAGCATTTATCAGGACCTGGACAACCTGCGCCTGCTGGCCGACAACCTGGCTCGCGATCCGCGCGCCAAGATCACTTTAGGCTGTCTGCCAAGCCTGGGGTTAAGCCTGGTGCCGGAGCTGGTGACCGACTTTTATCAGCAAAACAGCAACGTGGTGATGACGCTCACTACCGAGCACACCGAGACGCTGGTCAAAAAACTGGATTTGCGCGAGATAGATTTGGCGCTGACGCTACAGCCGGTGCAGCAGGGAGAGATCCTGACCACCCTGATTGCCGAAGTACCGCTGGTGTACATCGACCGTCATTATCGTCAGGGGGCGGTGGAGATCGATCAGATCGACCAGCAGCGCTGGATCTCGCCGGGACCGCACTCGCTCTCTGCCGCCATCGCCACCCGTCGCGACTTTTCCACCACCCGGCTAAACGTCCAGACCTACTATATGGCCACCGAGTTCGTGAAGCGCGGCATGGGCTGCAGCATCACTGATATCTTCTCCGCCCAGCACAACCTGGCCCCGGAGATGATCCATCCCATTACGCCGCCGATGGCGATTAACCTCTGTCTGTTACGCCGGGCCGACGTTTCCCTGAGCCCGATGGCGCAAAAGTTCGTCGATTTTCTCTGCCAGCGTCTGCGCCAGCAGCTCAAGGAGATTAACCTGCGGTTATACCCTGACCATAAAAAGTCAATTGCGCCGCTGGGGTAA

>IR1230_00112

ATGAACAAACGCATTGTCATTATCGGCGGCGGCGTGGTCGGTCTGGCCACGGCCTGGGAACTGATCAAGCGCGGCCATCAGGTGCAATTACTGGAGCGCAATGCCGAACCGGGCAGCGCCACCAGTTTTGCCAACGGCGGACAATTAAGTTACCGCTACGTCGCCCCCCTTGCCGACAGCGGCGTTCCCCTGCAGGGAATGAAATGGATGGGCAAAGCCGATTCGCCGCTGAATATGCGTCTCAGGATGTCTCTCCAGCAGTGGCGCTGGCTGCTGCAGTTTCTGCGCGCCTGTAATAACCAGACCAATAAAATGAATGGCGACCATATTTTACGCCTGTCGTTGCTCAGCCGTCAGGTCATGCAGGCGTGGCGGGATGAGGATAATCTTGCCGATTTCCACTGGCGGCGCAGCGGAAAATTAATTATCCATCGTCGCGAATATGATTTTAATAAAGCAGCAAAAGGGATTGATCCTCAGTATCAGCAGGCGCTGAATGCTGAAGCCTGCCTGCAGCTGGAGCCGGCGCTCAAACATATTAGTCCTTCTCTGCAGGGCGGAATCTATTCGCCAGGCGATGAAACGGCAGATTGTCATCAGTTCTGCCTTGCCCTGCTCGACAAGCTCAATGCCAGCAGCGACTTTAGCCTGCTGACCCAGTGTGAGGTGCGGCGACTCAATAAGCGCGGGGGACGCATCAGCAGCCTGGAGACCAGTCAGGGGACCCTCACCGGCGATGAGTATGTCGTCGCCGCCGGTAACGGCAGTGGTTCTCTGCTTGGGCATCTGGGGGTTCGCGTGCCGCTGTGCGCCCTGAAGGGGTACAGCCTGACCCTGCCCTATCCTGAAAAAGCAGGTATTGCCCCTGACATCAGCGTCACCGACTACGGGCACAAAATCGTTTATGCCCGCCTTGGCCAACAGCTGCGTATCGCGGCGATGGTGGACATCGGCTACGACGGCGACGAACTGCGCGAAAGCCGCATTCAGGCGCTGAAAAACATCGTCGCCCGCAGCTTTCCTGAACTGGAGGGGCTGGACGAGGCCGAAGTCTGGACCGGCATGCGCCCGTCAACGCCAGCTGGCCCGCCGATGCTGGGGCGCGCCGGATACCCCAACCTGTGGATGAACCTCGGCCAGGGTAGCCTCGGCTTTACTCTCGCCGCCGGCAGCGCGGTGGTGCTGGGCGCGTTAATCGACAATCAGATGCCAGACATTTCTTTAGAAGGTCTCACATGGAAACAAACCGCATGA

>IR1230_00113

ATGGAAACAAACCGCATGACAATTATTCGCAATAATCCACAACCTCGTCTTGCCGCCAGCGTTGCGTATGGCGATCTGCTGTTCCTCTCCGGGCAGACGCCAAAAAGCAATGAAGACGATATCGTTTTACAAACCCGTGAAGTACTGGAGAAGATCGACGCGCTGCTGGCCGCGGCCGGGAGCGATAAGCAGCACATTCTCTCCGCGCAGATCTGGTTGAAAAATATCGAGCGTGACTTTGCCGCATTCAATGAGGTGTGGGTGCAATGGATGCCGGAAGGCTATAGCCCGGCGCGAGCGGCCGTGCAGGCGGAGATGGCGCGACCAGAAATCCTGGTGGAGATCATGCTCACCGCCGTCAAAGCGTAA

>IR1230_00114

ATGGCGCTTACAAAAGCTGAAATGTCAGAATATCTGTTTGATAAGCTTGGGCTTAGCAAGCGGGATGCCAAAGAACTGGTCGAGTTGTTTTTCGAAGAGATCCGTCGTGCTCTGGAAAACGGAGAACAGGTCAAACTCTCCGGGTTCGGCAACTTCGATTTGCGGGATAAAAACCAACGCCCGGGACGTAACCCGAAGACGGGGGAGGATATTCCCATTACAGCCCGGCGCGTGGTGACCTTCAGACCCGGTCAGAAGCTGAAAAGCCGTGTCGAAAACGCGTCGCCGAAAGACAAATAA

>IR1230_00115

ATGAAATTCAGTGAACTGTGGTTACGCGAATGGGTTAACCCGGCGATCGACAGCGAAGCGCTTTCCGATCAGATCACCATGGCGGGCCTCGAAGTAGATGGCGTTGAGCCGGTCGCCGGCAGCTTCAACGGCGTCGTTGTCGGTGAAGTGGTGGAGTGCGGTCAGCACCCGAACGCCGATAAACTGCGCGTCACCAAAGTCAACGTCGGCGGCGAACGCCTGCTGGACATCGTCTGCGGCGCGCCAAACTGCCGTCAGGGGCTAAAGGTCGCGGTAGCCACCATCGGCGCCGTGCTGCCGGGCGATTTCAAAATCAAGGCCGCGAAGCTGCGCGGCGAGCCGTCTGAGGGGATGCTGTGCTCCTTCTCCGAGCTGGGTATTTCCGACGACCATAGCGGTATCATCGAGCTGCCGGCAGATGCGCCCATCGGCACCGATATTCGCGAATATCTCAAGCTTGACGATAACACCATCGAAATCAGCGTCACGCCAAACCGCGCCGACTGCCTGGGCATCATTGGCGTGGCGCGCGATGTCGCTGTCCTGAACAAAGCGCCGCTGAACGCGCCGGAAATCACGCCGGTTGCCGCGACGATTGACGACGTGCTGCCGATTCAGGTTGACGCGCCGCAGGCTTGCCCGCGCTATCTGGGGCGCGTGGTGAAAGGTATCAACGTCAAAGCGCCGACGCCGCTGTGGATGAAAGAGAAGCTGCGCCGCTGCGGGATCCGCTCCATCGACGCGGTGGTTGACGTGACCAACTATGTGCTGCTTGAGCTGGGTCAGCCGATGCACGCCTTTGACCGCGACCGTATTGAAGGCGGGATCGTGGTGCGCATGGCGAAAGAAGGGGAAACCCTGGTCCTGCTCGATGGCTCTGAAGCAAAGCTCGACAGCGACACTCTGGTCATTGCAGACCATAACAAAGCGCTGGCGATGGGGGGGATCTTCGGCGGGGAACACTCCGGCGTTAACGACGAAACCCAGAACGTGCTGCTGGAATGCGCGTTCTTCAGCCCGCTGTCCATTACCGGCCGCGCGCGCCGTCATGGCCTGCACACCGATGCCTCTCACCGCTATGAGCGCGGCGTCGATCCGGCCCTGCAGTATAAAGCGCTGGAGCGTGCAACCCGTCTGCTGATCGACCTCTGCGGCGGTGAAGCCGGCCCGGTCATTGACGTTACCAGCAAAGAGAATCTACCAACACGCGCCACCATTACGCTGCGTCGCAGCAAGCTGGATCGCCTGATTGGCCACCATATCGCTGACGCGCAGGTCACCGACATTCTGCAGCGCCTCGGCTGCGAAGTGACGGTTGGCGAGGGCGAATGGCAGGCGGTTGCGCCGAGCTGGCGTTTCGACATGGAGATCGAAGAAGATCTGGTTGAAGAAGTGGCTCGCGTTTACGGCTACAACAACATTCCTGACGAGCCGGTGCAGGCGGGTCTGATCATGGGAACCCACCGCGAAGCGGACCTGTCGCTGAAGCGCGTGAAAACGCTGCTGAACGACAAAGGCTATCAGGAAGTGATCACCTACAGCTTCGTCGACCCGAAAGTCCAGCAGCTGATCCACGCCGGGGAAGAGGCGCTGATCCTGCCAAGCCCGATCTCCAGCGAAATGTCGGCGATGCGCCTGTCGCTGTGGACCGGCCTGCTGGGCACCGTGGTCTACAACCAGAACCGTCAGCAGAGCCGGGTGCGCATCTTTGAGAGCGGCCTGCGCTTTGTGCCGGACACCAATGCGCCGCTGGGCATTCGTCAGGATGTGATGCTGGCCGGGGCGATCTGCGGCAACCGCTACGAAGAGCACTGGACACTGGCGAAAGAGACGGTCGATTTCTATGATCTGAAAGGCGATCTGGAAGCCGTTCTGGATTTAACCGGAAAATTAGCGGATATCGAATTCCGTGCTGAAGCGACCACCGCGCTGCATCCGGGACAATCCGCAGCGATTTATCTTAAAGGTGAACGCATTGGTTTCATTGGGGTTGTTCATCCTGAGCTGGAACGTAAACTGGATCTGAACGGTCGCACTCTGGTGTTCGAACTGGAGTGGAACAAGCTCGCAGACCGCGTGGTGCCTCAGGCGCGCGATATTTCCCGCTTCCCGGCGAACCGTCGCGACATCGCGGTGCTGGTCGCGGAAAACGTGGCTGCCGCCGATGTTTTAGCCGAATGTAAGAAAGTTGGCGTAAATCAGGTAGTTGGCGTAAACTTATTTGACGTGTACCGTGGTAAGGGTGTGGCGGAAGGGTATAAGAGCCTTGCGATTAGCCTGATCCTGCAGGATACCAGCCGTACACTCGAAGAAGAGGAGATTGCCGCTACCGTTGCCAGATGTGTAGAGGCATTAAAAGAGCGATTCCAGGCATCATTGAGGGATTGA

>IR1230_00116

ATGTCACATCTCGCAGAGCTGGTTGCCAGTGCCAAGGCAGCCATTAACGAGGCATCAGATGTTGCTGCGCTGGACAACGTCCGCGTGGAATACCTGGGTAAAAAAGGGCATCTGACCCTGCAAATGACCACCCTGCGTGAGCTGCCGCCGGAAGAGCGTCCGGCAGCCGGTGCGGTCATCAACGAGGCGAAAGAGCAGGTGCAGCAGGCGCTGAACGCGCGTAAAGCTGAACTGGAAGGCGCAGCGCTGAATGCGCGTCTGGCCGCAGAGACCATTGACGTCTCGCTGCCGGGTCGCCGCATTGAAAACGGCGGCCTGCATCCGGTTACTCGCACCATCGACCGTATTGAAAGTTTCTTCGGTGAGCTCGGCTTTACCGTGGCGACGGGCCCGGAAATCGAAGACGATTACCACAACTTCGATGCGCTGAATATTCCGGGTCACCATCCGGCGCGCGCTGACCACGACACTTTCTGGTTCGATGCCACTCGCCTGCTGCGTACGCAGACCTCCGGCGTGCAGATCCGCACCATGGAAAACCAGCAGCCGCCTATCCGTATTATCGCGCCGGGCCGCGTCTATCGTAACGACTACGATCAGACCCACACCCCGATGTTCCATCAGATGGAAGGTCTGATCGTTGATAAAAATATCAGCTTCACCAACCTGAAAGGGACGCTGCACGACTTCCTGAACAACTTCTTTGAAGAAGATCTGCAGGTCCGCTTCCGTCCGTCCTACTTCCCGTTCACCGAACCGTCTGCGGAAGTTGACGTGATGGGTAAAAACGGCAAATGGCTGGAAGTGCTGGGCTGCGGGATGGTGCACCCGAACGTGCTGCGTAACGTGGGTATCGATCCGGAAGTCTACTCCGGCTTCGCCTTCGGCATGGGCATGGAGCGTCTGACCATGCTGCGCTATGGCGTCACCGATCTGCGCGCGTTCTTCGAAAACGATCTGCGTTTCCTCAAACAGTTTAAATAA

>IR1230_00117

ATGGCTCGCGTAAAACGTGGTGTAATTGCACGTGCACGTCACAAGAAAATTTTGAAACAAGCTAAAGGCTACTACGGTGCGCGTTCTCGCGTATACCGCGTTGCCTTCCAGGCTGTTATCAAAGCTGGTCAGTACGCTTACCGTGACCGTCGTCAACGTAAACGTCAGTTCCGTCAACTGTGGATTGCACGTATCAACGCAGCAGCACGTCAGAACGGTATTTCTTACAGCAAATTCATCAATGGCCTGAAAAAAGCCTCTGTTGAAATCGACCGTAAGATCCTGGCTGACATCGCTGTATTCGACAAAGTAGCGTTCACCGCTCTGGTCGAAAAAGCGAAAGCAGCTCTGGCATAA

>IR1230_00118

ATGCCAAAAATTAAGACCGTACGCGGTGCTGCTAAGCGCTTCAAAAAAACCGGTAAAGGTGGTTTTAAGCACAAGCACGCTAACCTGCGTCACATTCTGACTAAAAAAGCTACCAAGCGTAAACGTCACCTGCGTCCGAAAGCCATGGTTTCCAAAGGCGATCTGGGCCTGGTTATCGCGTGCCTGCCGTACGCATAA

>IR1230_00119

GTGAGTCTGAGAGAAGCTATCGAAAAGGCTGAAGAGGCTGGAGTAGATTTAGTTGAAATCAGCCCTAACGCCGAGCCGCCAGTTTGTCGTATCATGGACTACGGCAAGTTCCTTTATGAAAAGAGTAAGTCTTCTAAGGAACAGAAGAAGAAGCAGAAAGTTATCCAGGTTAAGGAAATTAAATTCCGCCCTGGTACTGATGAAGGTGACTATCAGGTAAAACTCCGCAGCCTGATTCGCTTTCTCGAAGATGGCGATAAGGCCAAGATCACGCTGCGTTTCCGCGGTCGTGAGATGGCCCACCAGCAGATCGGTATGGAAGTGCTTAACCGCGTGAAAGACGATCTGGTTGAACTGGCAGTAGTCGAATCCTTCCCAACGAAGATCGAAGGCCGCCAGATGATCATGGTGCTCGCTCCTAAGAAGAAACAGTAA

>IR1230_00120

ATGCCTGTAATTACGCTTCCTGATGGCAGCCAACGCCATTTTGACCACGCAGTTAGTCCGATGGATGTCGCGCTGGATATCGGTCCAGGCCTGGCGAAAGCCACCATTGCCGGGCGGGTAAATGGTGAACTGGTAGACGCCTGTGACCCGATCGAATCCGATTCCACTCTCTCTATCATCACCGCGAAAGATGAAGAAGGGCTGGAGATCATTCGTCACTCCTGCGCGCACCTGTTAGGCCATGCCATCAAACAGCTGTGGCCCAACACCAAAATGGCTATCGGTCCGGTTGTCGATAATGGTTTCTACTATGACGTAGACCTCGACCACACCCTGACCCAGGAAGATATCGACGCGCTCGAAAAACGTATGCATGAGCTCGCCGAGAAAAACTACGACGTTATCAAGAAGAAAGTGAGCTGGCATGAAGCGCGTGAAACCTTCGTGAAACGCGGCGAAAGCTATAAAGTTTCTATTCTTGATGAAAACATTGCCCATGATGACAAGCCTGGCCTGTATCATCATGAAGAATATATCGATATGTGCCGCGGTCCGCACGTACCGAACATGCGCTTCTGCCATCACTTTAAGCTGATGAAAACCGCCGGAGCCTACTGGCGCGGCGACAGCAACAATAAAATGTTGCAGCGTATCTACGGCACCGCGTGGGCAGATAAAAAAGCGCTGAATGCCTACCTGCAGCGTCTGGAAGAAGCCGCCAAGCGTGACCACCGTAAAATCGGTAAGCAGCTCGACCTGTATCACATGCAGGAAGAGGCGCCGGGGATGGTGTTCTGGCACAATGACGGCTGGACCATCTTCCGTGAACTGGAAACGTTTGTTCGTTCTAAACTGAAAGAGTACCAGTATCAGGAAGTAAAAGGTCCGTTCATGATGGACCGTGTGCTGTGGGAAAAAACCGGCCACTGGGACAACTACAAAGATGCGATGTTCACCACCTCTTCTGAGAACCGTGAATACTGTATCAAGCCGATGAACTGCCCGGGTCACGTGCAGATCTTCAACCAGGGGCTGAAATCCTACCGCGACCTGCCGCTGCGTATGGCGGAATTCGGTAGCTGCCACCGTAACGAACCGTCTGGCGCGCTGCATGGTCTGATGCGCGTGCGCGGCTTTACCCAGGATGATGCGCATATCTTCTGTACTGAAGATCAGGTTCGCGATGAAGTGAACGCCTGTATTCGTATGGTCTACGATATGTATAGCACCTTTGGCTTCGAGAAGATCGTCGTCAAACTGTCGACTCGCCCGGAAAAACGTATCGGTAGCGACGAGACCTGGGATCGCGCGGAAGCGGATCTGGCGGTGGCGCTGGAAGAAAATAACATCCCATTTGAATATCAACTGGGTGAAGGGGCGTTCTACGGCCCGAAAATTGAATTTACCCTGTATGACTGCCTCGATCGTGCATGGCAGTGCGGTACCGTACAGCTGGACTTCTCTCTGCCGCAGCGTTTAAGCGCCTCCTATGTGGGCGAAAACAACGAGCGTCAGGTGCCGGTCATGATTCACCGTGCGATTCTCGGTTCTCTGGAGCGCTTCATTGGCATCCTGACCGAAGAGTTCGCAGGCTTCTTCCCAACCTGGATTGCACCAGTGCAGGTAGTGGTCATGAATATTACCGATTCTCAGGCTGAATACGTTAACGAATTGACGCGTAAACTACAAAATGCGGGCATTCGTGTAAAAGCAGACTTGAGAAATGAGAAGATTGGCTTTAAAATCCGCGAGCACACTTTACGTCGTGTCCCGTATATGTTGGTCTGTGGCGACAAAGAAGTCGAAGCCGGCAAAGTGGCCGTGCGCACCCGTCGCGGGAAAGACCTCGGCAGCATGGACGTAAATGAAGTGATCGAGAAGCTGCAACAAGAGATTCGCAGCCGCAGTCTTCAACAACTGGAGGAATAA

>IR1230_00121

ATGAAAAACAATCGTTATACCATCGCAGCCCTGGCGCTGTGCGTGCTTTCTTTTGGCGCATCAGCCGCCACGCCCGTTCCGCCATCGGGTCATCAGGATCATACCCAGGTAGGTATCGCCAAAGCCTCTGACGTTGAGGCTGGTTCGAATATTGCGCCCGGCGCCCCTACCACCGGAAAATCCATGAACGACGCCTTTAATGTGCATACGCTGGTGGCCGGCGAGTGGTCTTAA

>IR1230_00122

ATGAAGCTTTTAAAGACAGTACCCGCAGCGGTTATGCTGGCGGGGGGGCTTTTTGCGTCTGTTGGCGCAATGGCCGATGATTCTGTATTTACCGTCATGGATGACCCTTCCGCTGCCAAAAAACCGTTCGAAGGGGTGGTTAACGCAGGCTACCTGGCGCAGTCCGGCAACACCAAGAGCTCTTCGATGACGGCGGATTCCACCCTGACCTGGTATGGTAATACCACCGCCTGGTCGCTGTGGGGCAACGCCAGCAACACGTCGTCCAATGATGAGCGATCCTCAGAAAAATATGCTGTCGGCGGACGTAGCCGCTACAACCTGACCGATATGAACTATGTCTTTGGTCAGGGTAGCTGGCTAACAGACCGCTACAACGGCTATCAACAGCGCGATGTTGTGACGGCGGGCTATGGTCGTCAGATCCTCAACGGGCCGGTGCACAGTCTGCGGTTTGAATTCGGTCCTGGCGTTCGCTATGACGAATATACCGACGGGGATACTGATACCCAGCCGCTGGGTTATGCTTCGGGTACATGGGCCTGGCAGATGACCGATAACGCCAAATTTTCGCAAGGGGTATCGGTGTTCGGCGCCGAGGACACCACGGTCAACTCAGAGAGCGCGTTAAACGTGGCGATTAACGCCCATTTCGCCCTGAAGGTCGCCTACAACGTGACCTGGAACTCTGAGCCGCCTGCGTCCGCGCCGGAGCATACCGATCGCCGGACCTCGCTCTCCCTCGGGTATAAGATGTAA

>IR1230_00123

ATGACCAAAATTTACACGCTCACGCTGGCCCCCTCGCTGGATAGCGCCACGCAAACCCCACAGATCTACCCGGAAGGCAAACTTCGCTGCAGCGCGCCGGTATTTGAACCTGGCGGCGGCGGGATCAACGTGGCCCGCGCCGTGACCTTTCTCGGCGGCAAAGCCACCGCCATTTTCCCGGTCGGTGGCGCTACCGGCGAGCATCTTGCCGCCCTGCTGGCCGATGAGCAGGTGCCGGTCGAGACCGTCGAAACCCGCGACTGGACGCGGCAAAACCTGCACGTCCACGTGGCCGCCAGCGGCGAACAGTATCGCTTCGTTATGCCCGGCGCGGCACTGACGGACGATGAATTCCGCCGTCTGGAAGAGAAGGTTCTGACTATCGAACCCGGCTCTCTGCTGGTGGTCAGCGGCAGCCTGCCGCCAGGTATTAGCGTCGATAACCTGATGCAGCTGGTGAAAAATGCGCAACAGCAGGGACTGCGCTGCATCATTGACAGTTCCGGCGATGCGCTGGCCGCCGCGCTGGACGTCGGCAATATTGAGCTGGTGAAACCGAACCAGAAAGAGCTTAGCGCCCTCGTGCAGCGCGATCTCAGCCAACCCGATGACGTGCGCCTTGCCGCTCAGTCGTTGATCCAGTCCGGTAAAGTGCGCCGGGTGGTGGTCTCCCTGGGTCCGCAGGGGGCATTAGGCGTGGACGCCAGCGGCAGCGTTCAGGTAGTGCCGCCACCGATGAAAAGCCAGAGCACCGTCGGCGCTGGCGACAGCATGGTGGGCGCCATGACCTTGCGTCTGGCGGAAAACGCCAGCCTTGAAGACATGGTACGCTTTGGCGTCGCCGCCGGTAGCGCCGCGACCATCAACCAGGGCACCCGCCTCTGCTCGCGGGCCAATACGCAAAAAATCTATGATTATCTCTGCGGGCGTTGA

>IR1230_00124

ATGAGTAATGAGATTACCGCCTGGGTCGTCAGCGTGACCTTCCATGAACAGGCGCTGACGGAGATCAATGAGGTCAGCAACCATTTTACCCGTGCCGGGTTTGTACTGACGCTCAATGATGAAGAGGGCTCGCCCCATGAGCTGGGTACCAACACCTTCGGCCTGCTGAGCGGCCAGACGGCCGAGGAAGTTAAGGCCCTCAGCGCCGGCCTGGCGGAAGCGGCCCTTGGCCGCCCGGCGGAAATCGCTGTGGCCACATTCGCCGAGTGGTTAAAAGCTCAATAA

>IR1230_00125

ATGTGGCAAGCAATCAGTACTCTTTTACGCGATTGGCATACCGAAGACGCTGAAATCGAACTGAAAACCGAGCTGCCCGGTGGAGAGATCCATTCCGCCTGGCATTTACGCTTCGGCGGGAAAGATTACTTCGTGAAATGCGACGAACGGGAACTTCTGCCTATCTTCACCGCCGAGTCCGATCAGCTGGAGCTGCTGTCGCGAAGCAAAACCGTTCGCGTACCGCAGGTCTTTGCCGTCGGCAGCGATCGCGACTATAGCTTCGTGGTCATGGAGTACCTCCCTCCCCGTCCGCTGGATGCGCACAATGCGTTTCTGCTTGGCCAGCAGCTAGCCCACCTCCATCAGTGGAGCGATCAGCCGCAGTTCGGTCTGGATTTTGATAACGATCTTTCCACCACGCCGCAGCCCAACGCCTGGCAGCGTCGCTGGTCGGTTTTTTTTGCCGAGCAGCGTATCGGCTGGCAGCTGGAGCTCGCGGCGGAGAAGGGATTGCACTTTGGCGATATCGATACCTTAGTCGATATGGTACAGCAGCGACTGGCCAACCATCAGCCGCAGCCTTTGCTGTTGCACGGGGATCTGTGGTCCGGCAACTGCGCTTTAGGGCCGGATGGCCCCTATATATTCGATCCCGCCTGCTACTGGGGCGACCGCGAGTGCGATTTAGCCATGCTGCCGATGCATCCGGAACAGCCCCCGCAAATCTATGACGGCTATCAGTCCGTTTCGCCTCTGCCGTCGGGTTTTCTCGATCGTCAGCCGATTTATCAGCTTTATACTCTGCTGAACAGAGCGATTTTGTTTGGCGGCCAGCACCTGGTAACGGCCCAGCAGGCGCTGGATGATGTATTGATGGAGAAAATGCGTTAA

>IR1230_00126

ATGACCCTTGATTTACCTCGCCGCTTTCCGTGGCCAACGCTGCTGTCCGTGGCTATCCACGGTGCCGTTGTGGCGGGGCTGCTTTATACCTCGGTACATCAGGTTATTGAACAGCCTTCTCCGACGCAGCCGATAGAGATCACAATGGTGGCGCCGGCCGATCTTGAGCCGCCTCCGGCGGCGCAGCCTGTCGTGGAGCCCGTTGTTGAACCCGAACCTGAGCCGGAGCCAGAGGTAGCGCCTGAACCGCCGAAAGAGGCGCCGGTGGTGATCCATAAACCGGAACCTAAGCCGAAGCCCAAACCTAAACCCAAGCCTAAGCCGGAGAAAAAGGTTGAACAGCCGAAGCGGGAAGTGAAGCCGGCAGCAGAGCCGCGTCCGGCCTCGCCGTTTGAAAACAACAATACGGCGCCGGCGCGTACAGCGCCAAGTACCTCGACCGCAGCGGCTAAACCCACCGTTACTGCTCCGAGCGGCCCGCGGGCGATCAGCCGCGTTCAGCCGTCCTATCCGCCGCGCGCTCAGGCGCTGCGCATTGAAGGGACGGTACGGGTGAAGTTTGACGTTTCGCCTGATGGCCGCATTGATAATCTGCAGATCCTCTCTGCTCAGCCGGCGAATATGTTCGAACGCGAGGTGAAAAGCGCGATGCGCAGATGGCGTTATGAGCAGGGAAGACCGGGTACCGGCGTAACGATGACCATCAAATTCCGCCTGAACGGCGTCGAGATTAACTGA

>IR1230_00127

GTGCTTTACGTTATCTACGCTGAAGATATCGCCGATTCTCTGGAAAAGCGGCTGTCGGTTCGCCCCGCGCATCTCGCGCGTTTACAACTCTTACACGATGAAGGCCGACTGCTTACCGCCGGGCCAATGCCGGCCGTTGACAGTAACGAACCGGGCGCAGCAGGATTCAGCGGCTCAACGGTGATTGCCGAGTTTGAATCGCTGGAAGCGGCCCAGGCCTGGGCTAATGACGATCCGTATATCGCCGCCGGGGTTTACCGCCAGGTCTCAGTTAAGCCGTATAAGAAGGTTTTTTGA

>IR1230_00128

ATGTTTTCAATAAGCCCTGTATTTTTTTGGGCTAACATTTATGTACCTGCCGATTTTGAGGATTATTGTGTGAATACCCTGAAAAAAACCGCATTGCTTTCTGTACTTGCCTTATATATTCCTGTGTCGCAAGCCGCGGCTAAGGAATACAGTCTTGATCCACAGCATACCTCGGTTGTGATCTCCTGGAACCATTTCGGCTTCTCAAACCCTACCGCCTATATCTCTGATGTTTCAGGGAAACTGGCCTTTGATAAAGAGAATCCGGAGAAATCCTCGGTGAACGTCACGCTGCCAGTGAAAACCATTGACGCTCACGTCAAGGCGTTAACGGATGAGTTTTTAGGCAAAGAGTATTTTGATGTTAAAACGTTTCCCGACGCCACTTTCCAGAGTACCAAAGTGGAAAGCAAAGGCGACAATAAATATGATGTGGAAGGTAATTTGACCATTAAAGGCATCACCAAACCGGTGGTCCTGCATGCCGTCCTGAATAAGCAGGATATGCACCCAATGGTGAAAAAAGAGGCCATTGGATTTGATGCCACCGGGGTGATAAAACGTTCAGACTTTAAGCTTGATAAATATGTGCCGGCGGTAAGTGATAACGTCACCATCACGTTATCCACGGAAGCCTATGCAAAATAA

>IR1230_00129

ATGGGGGCTGTTTTCACTCGTAGAGTCGTTGCAATGAAGCGTAGTAGAACGGAAGTAGGGCGCTGGCGCATGCTGAGACAGGTGAATCGCCGTAAAGCGCGCTGGCTGGAAGCCCAATCGCGCCGCAATATGCGGATCCTCGCTATCAGGAAGGGGCTGGTGAAACGGCAGCGTCATGCCCTGCTGTTTATCTTCCCTGACAGCTAA

>IR1230_00130

ATGACAACCTTTTACACGGTGGTGAACTGGCTGGTCATTCTGGGGTACTGGCTGTTGATCGCCGGTGTAACATTACGAATTCTAATGAAACGCCGCGCTGTCCCGTCCGCCATGGCATGGTTGCTGATCATTTATATCCTGCCGCTGGTGGGGATCATCGCCTATCTCTCCTTTGGCGAGCTGCATCTCGGCAAGCGTCGGGCCGAGCGCGCCAGGGCGATGTGGCCCTCGACGGCCAAGTGGTTAAACGACCTCAAAGCCTGCAAACATATCTTCGCGGAAGATAACAGCCCGGTGGCCGAATCCCTGTTCAAGCTGTGCGAACGGCGTCAGGGCATTGGCGGCGTAAAAGGCAACCAGCTACAGCTGCTGACGGAATCTGACGACGTGATGCAGGCCCTGATCCGCGATATTCAGCTGGCCCGTCACAACATAGAGATGGTCTTTTATATCTGGCAGCCCGGCGGCATGGCCGACAGCGTCGCTGAATCGCTGATGGCCGCCGCTCGCCGCGGGGTTCACTGTCGGCTAATGCTTGACTCCGCGGGCAGCGTGGCCTTTTTCCGCAGCCCATGGGCGGCGATGATGCGTAACGCCGGTATTGAAGTCGTCGAAGCTCTTAAGGTTAACCTGATGCGCGTATTCCTGCGCCGGATGGACCTGCGTCAGCATCGTAAAATGGTGATGATCGATAACTATATCGCCTACACCGGCAGCATGAATATGGTCGATCCGCGCTACTTTAAACAGGACTCAGGAGTCGGACAGTGGATTGATCTGATGGCGCGAATGGAAGGCCCGGTGGCGACCTCGATGGGCATCGTCTACTCCTGCGACTGGGAAATAGAGACCGGCAAGCGCATTCTGCCGCCGCCCCCGGACGTCAATATCATGCCGTTCGAAGAGGCCAGCGGACATACTATTCACACGATCGCCTCCGGACCGGGCTTTCCGGAAGATCTGATTCATCAGGCTCTCCTCACCGCCGCCTATGCGGCGAAAGAGCATCTGATCATGACCACCCCCTACTTCGTACCCAGCGACGACCTGCTGCATGCGATCTGTACCGCCGCCCAGCGCGGCGTTGACGTGAGCATTATTTTACCGCGCAAGAACGATTCGCTGCTGGTAGGCTGGGCCAGCCGGGCCTTCTTCACCGAGCTGCTGGCGGCCGGGGTCAAAATCTATCAGTTTGAGGGCGGACTGTTGCATACCAAGAGCGTGCTGGTCGATGGCGAGCTAAGTCTCGTGGGGACCGTCAACCTGGATATGCGCAGCCTGTGGCTCAACTTCGAGATTACCCTGGTCATCGATGACGCCGGTTTCGGCAGCGATCTGGCGGCGGTTCAGGATGATTATATCTCGCGCTCGCGCCTGCTGGATGCCCGTCTCTGGTTAAAACGTCCGCTGTGGCAGCGGATCGCCGAGCGACTGTTTTACTTCTTCAGCCCGTTGCTGTAA

>IR1230_00131

ATGGATATGGATTTAAACAACCGCCTGACCGAAGACGAAACGCTCGAGCAGGCTTACGATATCTTCCTCGAACTGGCGGCCGACAATCTCGACCCGGCGGACATCATTCTGTTTAACCTGCAGTTTGAGGAGCGCGGCGGCGCCGAACTGTTCGACCCTTCGGCAGACTGGGAAGAGCATGTGGATTACGATCTCAACCCGGATTTCTTCGCCGAAGTGGTGATTGGTCTGGCGGATACCGACGGCGGGGAAATCAATGATATCTTCGCCCGCGTTCTGCTGTGCCGCGAGAAAGATCATAAGCTGTGCCATATTCTGTGGCGTGAATAA

>IR1230_00132

GTGGGTATGCTTTCTGCCGCTCGTCTGCGGCTTTATCATTTACTTTTCGATCAGAACCGACGTTCCGGCCGCCGCTTTGAAGGGCTGTGCGGACTTTTCGCCCTGCTCAGCGTACTGGTGATATTTATCGAGTCGGGACTGGGCACGCAATATCACCTGACATTAGATGAATGGCATATTTTCGTCTGGCTGGAGCTGCTGGTGACCGCGGTCTTTACCCTGGAATACCTGCTGCGTATCGCTACCTGGCCCAATCCGCTCCACTATATTTTCAGCTTTTGGGGGCTGATTGACCTGGCGACGATTTTACCGCTGTATGTGATGTGGCTATGGCCGGAAATTAGCCTCAATTATGTATTCGCCTGGCGGGCAATGCGGGCGATCCGCGCGCTGCGTATTCTCAAACTACTGCGCTTTATGCCTTCGCTCAACGTGTTCTGGCGAGCGATTGTCAGCGCGCGCCATCAGCTGATCCTGTTTTATTCGTTTATCGCCATTGTGATGGTGATTTTCGGCTCGTTAATGTATCTGATTGAGGGGCCGGAATACGGCTTTACCACGCTTAACGCCTCGGTCTATTGGGCCATCGTGACCATCACCACCGTCGGCTACGGAGACATCACGCCGCACACGCCGTTGGGTCGCATCCTGGCCTCCATTCTTATCCTGATCGGCTACTCCATTATTGCCATACCGACCGGACTTATCACCACGCATATGACCAGCGCCCTGAATCGTCGACGACAGCAGCGCCTGTGTCCGCAGTGTCAGCAGGGCGACCATGACGATAACGCCCGTTTCTGCCATGCCTGCGGTCATGCCTTGCCGAAGTAA

>IR1230_00133

ATGAACGCCGTAACTGAACAACGAAAAGTCCTGCTCGAAATTGCCGACCTGAAGGTGCACTTCGATATCAAAGATGGCAAGCAGTGGTTCTGGCAGCCGTCGAAAACGCTGAAAGCGGTGGATGGCGTGACGCTGCGCCTTTACGAGGGTGAAACCCTCGGTGTGGTAGGGGAGTCGGGCTGCGGCAAATCGACCTTCGCCCGCGCCATTATTGGTCTGGTGAAAGCGACCGATGGCAAAGTGGCCTGGCTGGGTAAAGATCTGCTGGGCATGAAGCAGGAAGAGTGGCGCGACGTGCGCAGCGATATCCAGATGATCTTCCAGGATCCGCTGGCCTCGCTGAACCCGCGCATGACCATCGGAGAAATTATCGCCGAACCGCTGCGTACCTATCATCCGAAGATGCCCCGTCAGGAAGTGCGCGATCGCGTCAAAGCGATGATGATGAAAGTTGGATTGCTGCCCAACCTGATCAACCGCTATCCGCACGAGTTCTCCGGCGGTCAGTGTCAGCGTATCGGCATCGCGCGGGCGCTGATCCTTGAGCCAAAGCTGATTATCTGCGATGAGCCGGTCTCCGCGCTTGACGTGTCGATTCAGGCGCAGGTCGTCAACCTGCTGCAGCAACTGCAGCGTGAGATGGGGCTATCGCTGATATTTATCGCTCACGATCTGGCGGTGGTTAAGCATATCTCCGATCGGGTTCTGGTAATGTATCTGGGCCATGCGGTGGAGCTGGGGACCTACGACGAGGTGTACCATAATCCGCTGCATCCCTATACCAAAGCGCTGATGTCGGCGGTGCCGATCCCCGATCCTGATCTCGAGAAAACGAAAACTATCCAACTGCTGGAAGGCGAATTACCCTCGCCGATTAATCCGCCCTCAGGCTGCGTATTCCGCACCCGCTGTCCGCTTGCCGGACCGGAGTGCGCCAAAACCCGCCCGGTGCTGGAAGGCAGTTTCCGCCATGCGGTTTCCTGCCTTAAGGTAGACCCGTTATAA

>IR1230_00134

ATGACCATGATTGAAACGGCAAAAGCGCCGCAAGCGCAGAGCCACAGCGGGCTACTGCTGGACGTGAAAGATCTTCGCGTCACCTTTAAAACGCCGGATGGCGACGTCACCGCCGTCAATGATCTGAACTTCACCCTGCAGGCGGGCGAGACGCTCGGCATTGTGGGCGAATCCGGCTCCGGTAAATCGCAAACCGCCTTCGCCCTGATGGGACTGCTGGCGGCCAACGGTCGCATCGGGGGTTCCGCCACCTTTAACGGTCGGCAAATCCTCAATTTGCCCGAGCGTGAGCTGAACAAACTGCGCGCTGAACAAATCTCAATGATTTTCCAGGATCCGATGACCTCTCTTAACCCCTATATGCGGGTGGGCGAGCAGTTAATGGAAGTCCTGATGCTGCATAAAGGGCTGAGTAAAGCCGAAGCGTTTGAAGAGTCGGTGAAGATGCTCGACGCGGTAAAAATGCCGGAAGCGCGTAAGCGCATGAAAATGTTCCCGCACGAGTTCTCCGGGGGCATGCGTCAGCGCGTGATGATTGCCATGGCGCTGCTGTGCCGGCCCCGGCTGCTGATTGCGGATGAACCGACCACTGCCCTGGACGTTACCGTGCAGGCGCAGATCATGACGCTGTTGAATGAGCTTAAACGCGAATTCAACACGGCCATTATCATGATCACCCACGACCTTGGGGTGGTGGCAGGTATCTGCGATAAAGTGCTGGTGATGTACGCCGGGCGCACCATGGAGTACGGTCAGGCGCGCGATGTCTTCTATCAACCGTCGCACCCATACTCTATTGGGCTGTTGAATGCGGTTCCACGTCTGGATGCGGAAGGCGATGCGTTGTTGACCATTCCCGGCAACCCACCGAACCTGCTGCGATTGCCGAAAGGCTGTCCATTCCAGCCGCGTTGCCCGCATGCCATGGAGCAGTGCAGCAGCGCGCCGCCGCTGGAATCGTTCGCGCCAGGCTGCCTGCGCGCCTGCTTTAAACCGGTGGGGGATCTGCTATGA

>IR1230_00135

ATGATGTTAAGTAAGAAAAATAGCGAGGCGCTGGAAAACTTCAGTGAGAAGCTGGAAGTGGAGGGGCGTAGCCTCTGGCAGGATGCGCGTCGTCGCTTTATGCATAACCGCGCCGCGGTGACCAGCCTGATTATGCTGGTGCTTATTGCGCTGTTCGTTACCTTCGCGCCGATGGTGTCGTCGTTCTCCTACTTCGATACCGACTGGGGCATGATGTCCAATGCTCCGGATATGGCCTCCGGCCACTACTTCGGAACCGACTCCTCCGGGCGCGATCTGCTGGTGCGCGTAGCGATCGGCGGGCGCATTTCGCTGATGGTCGGGGTGGCGGCGGCGCTGGTGGCGGTGATCCTCGGCACGCTGTACGGTTCGCTCTCCGGTTATCTCGGCGGCAAAGTGGATTCGGTGATGATGCGTCTGCTGGAGATCCTCAACTCCTTCCCGTTTATGTTCTTCGTCATCCTGCTGGTGACCTTTTTTGGTCAGAATATCCTGCTGATCTTTGTCGCCATCGGTATGGTGTCCTGGCTGGATATGGCGCGTATCGTGCGCGGGCAGACGCTGAGCCTGAAGCGCAAAGAGTTTATCGAAGCCGCGCAGGTCGGCGGGGTCTCGACGGCGAGCATCGTTCTGCGCCATATCGTCCCCAACGTGCTGGGGGTGGTGGTGGTGTATGCCTCGCTGCTGGTCCCCAGCATGATCCTCTTCGAATCCTTCCTCAGTTTCCTGGGACTGGGGACGCAGGAGCCGCTCAGCAGCTGGGGGGCATTGCTCAGCGATGGCGCCAACTCAATGGAAGTTTCGCCCTGGCTACTGTTGTTCCCGGCGGGTTTCCTGGTGGTGACCCTGTTCTGTTTCAACTTTATCGGCGATGGTCTGCGTGATGCCCTCGACCCGAAAGACCGCTAA

>IR1230_00136

ATGTTAAAATTTATCTTACGTCGCTGTCTGGAAGCGATTCCGACGCTATTTATTCTTATCACCATCTCATTCTTCATGATGCGTCTGGCGCCGGGAAGTCCTTTTACCGGGGAACGCACGCTACCGCCGGAAGTCATGGCGAATATTGAAGCAAAATACCATTTAAACGACCCTATCATGACGCAGTACTTCAATTATCTGAAGCAGCTGGCGCATGGCGATTTTGGTCCCTCTTTTAAATACAAAGATTATTCCGTCAACGATCTGGTGGCCGCCAGCTTCCCGGTTTCAGCGAAACTGGGCTTCGCCGCCTTTTTACTGGCGGTGGTGATCGGCGTTGCCGCCGGGGTGATAGCCGCCCTGAAGCAAAATACCCGCTGGGACTACGCGGTGATGGGGGTAGCCATGACCGGCGTGGTTATTCCGAGCTTTGTGGTCGCGCCGCTGCTGGTGATGATCTTTGCCATTACCCTGCACTGGCTGCCGGGCGGCGGCTGGAACGGCGGGGCGCTGAAATTTATGATCCTGCCGATGGTGGCGCTTTCCCTGGCCTATATCGCCAGCATCGCGCGTATCACCCGCGGCTCAATGATTGAAGTGCTGCACTCTAACTTTATCCGCACCGCGCGGGCGAAGGGGCTGCCGATGCGTCGCATTATTCTGCGCCATGCGCTGAAGCCGGCGCTGCTGCCGGTGCTCTCCTATATGGGGCCAGCTTTCGTGGGCATTATCACCGGCTCAATGGTCATTGAAACCATTTACGGCCTGCCGGGGATCGGCCAGCTGTTCGTTAACGGGGCGCTTAACCGCGACTACTCGCTGGTGCTCAGCCTGACGATCCTCGTTGGCGCCCTGACTATCCTGTTCAACGCCATCGTTGACGTGCTGTACGCCGTTATCGATCCGAAGATTCGCTACTGA

>IR1230_00137

ATGACCATCATCACTAAAAAGCGTTTAATCGCCGCGGGAGTGCTATCCGCACTGATCGCCGGCAATATGGCAATGGCAGCGGATGTCCCGGCTGGTGTGCAGCTGGCAGAGAAGCAAACCCTGGTACGTAATAGCGGCGCCGAGCCGCAATCGCTGGACCCAAATAAAATCGAAGGGGTGCCAGAGGCCAATATTAGCCGCGACCTGTTCGAAGGTTTGCTGAATACTTCGCCGAAAGATGGCCACCCGATCCCTGGCGTGGCGGAGAGCTGGGATAATAAAGATTTTAAAGTCTGGACCTTCCATCTGCGTAAAGATGCAAAATGGTCTAACGGCGAGCCGGTTACCGCCCAGGACTTCGTATACAGCTGGCAGCGTCTGGTGGATCCGAAAACCGCGTCTCCTTATGCCAGCTATCCGCAATATGGTCATATCGTTAACGTCGACGAAATCATTGACGGCAAGAAAGCGCCGAGCGAGCTGGGCGTAAAAGCCATTGACGACCACACGCTGGAAGTCACCCTCAGCGAACCGGTACCCTATTTTTATAAACTGCTGGTTAACCCGGCGATGTCTCCCGTCTACAAACCGGCCATCGAAAAATTTGGCGAAAAGTGGACGCAGCCTGGCAATATCGTTACCAACGGCGCGTATACTCTGAAAGACTGGGTGGTCAACGAGCGGATCGTGATGGAGCGTAACCCGCACTATTGGGATAATGCTAAAACCGTCATTAACACCGTTACCTGGCTGCCGACCTCTTCCGAAGTGACCTATGTTAACCGCTACCGCAGCGGTGAACTGGACATGACTTATAACCAGCTGCCGATCGAACTCTTCCAGAAGCTGAAAAAAGAGATCCCCAACGAGCTGCACGTTGACCCCTATCTGTGTACCTATTATTACGAAATCAACAACCAGAAGGCCCCGTTCACCGACGTGCGCGTGCGTACCGCGCTGAAGCTGGGTCTGGATCGCGATATTATTGCTAATAAGGTGAAAGGACAGGGCGATCTGCCGGCCTACGGCTACACCCCACCGTATACCGATGGCGCGAAGCTGAGCGAGCCGGAATGGTTTACCTGGTCGCAGGAAAAACGCAACGAAGAAGCGAAGAAACTGCTGGCCGAAGCCGGCTATAGCGCCGATAAACCGTTGACCTTCAACCTGCTGTATAACACCTCTGATTTGCACAAGAAGCTGGCGATTGCCGCCGCCTCGCTGTGGCGTAAAAACCTGGGCATTGACGTGAAGCTGGTCAACCAGGAGTGGAAAACGTTCCTCGATACCCGTCATCAGGGCACCTATGACGTGGCGCGTGCGGGCTGGTGCGCCGACTATAACGAGCCAACCTCTTTCCTGAACACCATGCTCTCTGACAGTTCAATGAACACCGCGCACTATAAGAGCCCGGCGTTCGACAAGATTATGGCTGAATCCGTAAAAGCATCCGATGAAGCGCAGCGCACGGCGGCCTATGCGAAAGCAGAGCAGCAGCTGGACAAAGACAGCGCGATCGTACCGGTCTATTACTACGTTAACGCCCGCCTGGTGAAACCATGGGTCGGTGGCTATACCGGTAAAGACCCGATGGATAACGTCTATACCAAAGACCTCTACGTTATCAAACATTAA

>IR1230_00138

GTGACTCAATCACTGTTCGATTTTTCTGCATATTTCAAGTTTTTCATCGGCTTATTTGCCCTGGTTAACCCGGTTGGCATTATTCCGGTATTCATCAGTATGACCAGCTATCAGCCGGCTGCGGTGCGTAATAAGACCAACCTCACCGCCAACCTGTCGGTGGCGATCATCCTCCTGACGTCGCTGTTCCTCGGCGATGGCATTCTGCAGATTTTCGGTATCTCCATTGACTCCTTCCGTATCGCCGGCGGCATCCTGGTGGTGACCATCGCGATGTCGATGATCAGCGGTAAGCTCGGCGAAGATAAGCAGAACAAGCAGGAAAAGTCGGAGACGGCGGTCCGGGAGAGCATCGGCGTCGTTCCTCTCGCGCTACCATTAATGGCCGGGCCAGGGGCAATCAGTTCAACTATCGTCTGGGGGACCCGGTACCACTCCTGGGTGCATCTGGTCGGGTTCTCACTGGCGATAGCGGTATTTGCCCTCTGCTGCTGGGGGATTTTCCGTATGGCGCCGTGGCTGGTCCGTTTGCTGGGGCAGACGGGAATCAACGTCATCACCCGTATTATGGGCCTGTTGCTGATGGCCCTCGGCATTGAGTTTATTGTGACCGGGATTAAAGCGCTTTTCCCAGGGCTGCTGAGTTAG

>IR1230_00139

ATGGCTGTTACTAATATCGCTGAACTGAACGCGCTTGTTGAGCGTGTCAAGAAAGCCCAGCGTGAATATGCCAGTTTCACTCAAGAACAAGTCGACAAAATCTTCCGCGCCGCCGCTCTGGCCGCTGCAGATGCTCGAATCCCTCTCGCCAAAATGGCCGTAGCCGAATCCGGCATGGGTATCGTTGAAGACAAAGTGATCAAAAACCACTTCGCTTCCGAATACATTTACAACGCTTATAAAGACGAAAAAACCTGTGGCGTTCTGTCAGAAGACGACACCTTTGGCACCATCACTATTGCTGAGCCTATCGGCATCATCTGCGGTATCGTACCGACCACTAACCCGACTTCAACAGCCATTTTCAAATCGCTCATCAGTCTGAAAACGCGTAACGCCATCATTTTCTCTCCGCACCCGCGTGCAAAAGAAGCCACCAACAAAGCGGCTGATATCGTGCTGCAGGCTGCCATCGCGGCCGGCGCGCCGAAGGACCTGATTGGCTGGATCGATCAGCCGTCTGTAGAACTGTCTAACGCGCTGATGCATCACCCGGACATCAACCTGATCCTGGCGACCGGTGGTCCAGGCATGGTGAAAGCAGCATACAGCTCCGGTAAACCTGCTATCGGCGTCGGCGCCGGTAACACCCCGGTAGTCATTGATGAAACCGCCGACATCAAACGTGCCGTAGCGTCCGTACTGATGTCTAAAACCTTCGATAACGGCGTGATCTGTGCTTCTGAGCAGTCCGTTGTGGTGGTTGATTCAGTCTATGACGCGGTTCGCGAACGTTTCGCCAGCCACGGCGGCTATCTGCTGCAGGGTAAAGAGCTGAAAGCCGTTCAGGACATTATCCTGAAAAATGGCGCGCTGAACGCGGCTATCGTTGGTCAGCCAGCGGCCAAAATCGCTGAACTGGCAGGCTTCACCGTGCCGGCCACCACCAAAATTCTGATTGGTGAAGTGACCAACGTTGACGAGAGCGAGCCGTTTGCTCACGAAAAACTGTCTCCGACGCTGGCTATGTACCGTGCAAAAGATTTCGAAGACGCGGTAGCCAAAGCAGAAAAACTGGTCGCCATGGGCGGTATCGGCCACACCTCTTGCCTGTACACCGACCAGGACAACCAGCCGGCTCGCGTGGCTTACTTCGGCCAAATGATGAAAACCGCGCGTATCCTGATCAACACCCCGGCTTCTCAGGGTGGTATCGGTGACCTGTACAACTTCAAACTCGCGCCTTCCCTGACTCTGGGTTGTGGTTCCTGGGGTGGTAACTCCATCTCTGAAAACGTTGGTCCGAAACACCTGATCAACAAGAAAACCGTTGCTAAGCGAGCTGAAAACATGTTGTGGCACAAACTTCCGAAATCTATCTACTTCCGTCGTGGCTCCCTGCCAATCGCACTGGATGAAGTGATTACCGATGGTCACAAACGCGCGCTGATCGTGACTGACCGCTTCCTGTTCAACAACGGCTATGCCGATCAGATCACTTCCGTACTGAAAGCTGCGGGTGTTGAGACGGAAGTCTTCTTCGAAGTTGAAGCTGACCCGACGCTGACCATCGTACGTAAAGGTGCCGACCTGGCGAACTCCTTTAAACCAGACGTGATCATCGCCCTGGGCGGCGGTTCCCCGATGGATGCGGCGAAAATCATGTGGGTCATGTACGAACATCCGGAAACCCACTTCGAAGAACTGGCGCTGCGCTTTATGGATATCCGTAAACGTATCTACAAGTTCCCGAAAATGGGAGTCAAAGCCAAGATGGTGGCGATCACTACCACTTCCGGTACCGGTTCTGAAGTGACGCCGTTCGCCGTGGTGACCGATGACGCGACCGGTCAGAAATACCCGCTGGCAGACTACGCGCTGACCCCGGATATGGCCATTGTCGATGCCAACCTGGTGATGGATATGCCGAAATCGCTGTGTGCGTTCGGTGGTCTGGATGCGGTGACTCACGCCCTGGAAGCTTACGTTTCCGTACTGGCTTCTGAGTTCTCTGACGGCCAGGCGCTGCAGGCGCTGAAACTGCTGAAAGAGTACCTGCCGGCTTCCTATCACGAAGGTTCCAAGAACCCGGTTGCCCGTGAGCGCGTACACAGTGCCGCGACTATCGCCGGTATCGCGTTCGCTAACGCCTTCCTCGGCGTGTGTCACTCGATGGCGCACAAACTGGGTTCCCAGTTCCATATTCCGCACGGTCTGGCCAACGCCCTGCTGATCTGCAACGTTATCCGCTATAACGCGAATGACAACCCGACCAAGCAGACCGCGTTCAGCCAGTACGACCGTCCGCAGGCTCGTCGTCGTTACGCTGAAATCGCCGATCACCTGGGCCTCTCCGCACCAGGCGACCGCACCGCAGCGAAAATCGAGAAACTGCTGGCATGGCTGGAAAGCATTAAAGCTGAGCTGGGTATTCCGAAATCTATCCGCGAAGCTGGCGTTCAGGAAGCTGACTTCCTGGCCCACGTTGATAAACTGTCTGAAGATGCATTCGATGACCAGTGCACCGGCGCTAACCCGCGCTACCCGCTGATCTCCGAACTGAAACAGATCCTGCTGGATACCTACTACGGCCGCGAATTCGTCGAAGGCGAAGCCGGTGCGAAAGCCGAAGTTGCTCCGGTAAAGGCTGAGAAAAAAGCGAAAAAATCCGCTTAA

>IR1230_00140

ATGCGTACCGTCGTTTATACCGCTGAGATTGATGACCGCTTTGGCGCCGGAAAAGTCAGTTCGCGTATCGGGCTCTCCTCGCCGGCCCGATTGTATAATCCACAGACCTCGTTATTTGACGATATTGCGGCGGAGCACCAGCTAAAGCCGATTCACTGCGTGCTGGTGGATGAAAGCCAGTTTCTGACGCGTGAACAGGTTCATGAGCTGTCCGAAGTTGTCGATACGCTGGATATTCCGGTGCTTTGCTATGGGTTGCGGACGGACTTTCGCGGTGAGCTCTTTACCGGCAGCCAGTATTTACTGGCCTGGTCAGATAAGCTGGTTGAGCTGAAAACGATCTGCTTCTGCGGCCGCAAGGCCAGCATGGTATTGCGCCTCGATCAGGAGGGGCGTCCTTATAATGAGGGTGAGCAGGTCGTCATCGGCGGTAATGAGCGGTACGTATCGGTCTGCCGTAAGCATTATAAAGAGGCGCTGAGCGTCGGTTCGTTAACCAAGGTACAAAACCAGCACCGTCCCTGCTGA

>IR1230_00141

ATGAGCGAAGCACTTAAAATTTTGAACAACATCCGTACTCTTCGTGCGCAGGCAAGAGAATGCACCCTGGAAACGCTCGAAGAAATGCTGGAAAAATTAGAAGTCGTCGTTAACGAGCGCCGTGAAGAAGAAAACGCTGCCGCGGCAGAAATTGAAGAGCGTACGCGTAAGCTGCAGCAATACCGTGAAATGCTGATTGCTGACGGTATTGATCCGAATGAACTGCTGAGCACCATGGCTGCCGTTAAAGCCGGTACCAAAACCAAGCGTGCTGCACGTCCGGCTAAATATAGCTATGTTGACGAAAATGGTGAAACCAAAACCTGGACCGGTCAGGGCCGTACCCCGGCTGTGATCAAAAAAGCAATGGATGAGCAAGGTAAATCACTGGACGATTTCCTGATCTAA

>IR1230_00142

ATGGCTGCCCTTAATTCTAAAGTCAGAAAAGCGGTTATCCCGGTTGCGGGATTAGGCACCAGGATGCTGCCGGCCACCAAAGCGATTCCAAAAGAAATGCTGCCGCTGGTAGATAAGCCGTTAATTCAGTACGTTGTTAATGAATGTATCGCTGCAGGAATTACTGAAATTGTTCTGGTAACGCACTCGTCGAAAAACTCTATCGAAAACCATTTCGATACCAGTTTTGAGCTGGAAGCGATGCTGGAAAAACGTGTAAAACGCCAGCTGCTGGAAGAAGTGCAGTCTATTTGCCCGCCGCACGTGACTATTATGCAAGTTCGTCAGGGCCTGGCGAAAGGTCTGGGTCACGCGGTGCTGTGCGCCCATCCGGTAGTGGGAGATGAGCCGGTCGCGGTTATTCTGCCAGACGTTATTCTGGATGAATATGAATCCGATCTGAGCCGCGATAACCTCGCTGAGATGATCTCTCGCTTCGATGAGACTGGCGCCAGCCAGATTATGGTTGAACCTGTCGAAGACGTAACCGCTTATGGCGTGGTTGACTGCAAAGGCGAATCCCTGAAGCCGGGCGAAAGCGTACCGATGGTGGGCGTGGTTGAGAAGCCAAAAGCTGACGTTGCGCCGTCCAACCTGGCGGTGGTGGGGCGTTATGTCCTCAGCGCTGATATCTGGCCGCTGCTGGCGAAAACCCCTCCGGGCGCTGGTGATGAAATTCAGCTGACTGACGCCATTGATATGCTGATCGAGAAAGAAACGGTTGAAGCTTATCATATGAAAGGCAAGAGCCATGACTGCGGTAATAAGCTTGGCTACATGCAGGCGTTTGTCGAATATGGTATTCGTCACAAAACGCTGGGCGACGATTTTAAAGCCTGGCTGGAAACGGCGGTAGCGAAGTAA

>IR1230_00143

ATGACGCAGCCATTGGCGGGAAAACAGATTCTGATAGTTGAAGACGAACCCGTTTTCCGCTCACTCTTGCATGGATGGCTGACCTCGCTGGGAGCAACAACCTTCCAGGCGGAAGATGGCAAGGATGCCCTGCATAAAATGACGGAGGTGCATCCGGACCTGATGATTTGCGACATTTCTATGCCGAGAATGAACGGTCTGGAGCTGGTGGAAACGCTGCGTAACCGCGGCGAACAGTTACCGATCCTGATGATCTCCGCCACCGAAAACATGGCGGATATAGCTAAAGCGTTGCGACTTGGCGTCCAGGACGTGCTGTTAAAGCCAGTAAAAGATTTCGATCGCCTGCGCGAGACCGTTTACGCCTGTCTCTATCCCGCGATGTTCAGTTCGCGCGTGGAAGAGGAAGAGCGTTTATTTGAGGACTGGGATGCGCTGGTCAGTAATCCCATCGCCGCTTCAAGACTATTGCAGGAATTACAGCCGCCGGTGCAACAGGAAATGTCGCACTGCCGCGTTCATTATCGACAACTGGTGTCGGCAGACAAGCCCGGTCTGGTACTCGATATCGCGCCGCTTTCGGAAAATGATTTGGCTTTCTATTGTCTGGATGTCACACGCGCAGGCGATAATGGCGTGTTGGCAGCATTATTGCTGCGCGCGTTGTTTAATGGTCTGCTGCAGGAGCAACTGGCGCACCAGGGACAACGGCTACCAGAAATGGGAAGTTTGTTAAAACAGGTGAATCAACTGCTGCGTCAGGCAAATCTTCCGGGCCAGTTCCCTCTGTTAGTAGGGTATTACCACAGCGGGCTGAAAAATTTAATTCTGGTGTCAGCGGGACTTAATGGCACGCTGAATACAGGGGAGCATCAAATTCAGATTAGTAATGGCGTTCCTTTGGGGACGCTGGGTGATGCTTATCTTAACCAAATTAGCCAACGCTGCACCTCGTGGCAGTGCCAAATTTGGGGGGCTGGCGGTCGTTTACGCTTAATGTTGTCTGCGGAATGA

>IR1230_00144

ATGAGAAAGCTTAAAATTGGTCTGGCGCTAGGTGCGGGAGCCGCTCGCGGATGGTCGCACATTGGCGTCATCAACGCCCTCCAGCGGGCCGGTATTGAGATTGATATCGTAGCCGGTTGTTCTATCGGTTCGCTGGTAGGGGCGGCGTATGCCTGTAACCGACTCCCGGCACTCGAATCATGGGTCTGCTCATTCAGCAACTGGGATGTCCTGAAGCTGATGGATCTCTCCTGGCGCCGGGGGGGACTGCTGCGGGGGGAAAAGGTATTCAATCATTACCGCCGCATTATGCCCGTCACCGAGATTGAACGCTGCGAGCGCCGGTTTGCTGCTGTTGCCACCAATTTAAGCACCGGTAGGGAGCTGTGGTTTACCGAAGGCGATCTACATTTAGCCATTCGCGCCTCCTGTAGTATGCCGGGGTTAATGTCGCCAGTAAGGCATAATGGTTACTGGCTGGTCGATGGCGCGGTGGTCAATCCGGTTCCTGTGTCGCTCACCCGGGCAATGGGAGCGGATATTGTGATTGCCGTCGACCTGCAACATGATGCCCATCTTATGCAGCAGGATTTGTTCTCCCTTGAAACGCCGCGGCAGGAAGCGGAAGAGAAGGAGGGGCCATGGCATACTCGTCTGCGCGCTCGCTTGACGCGAATGAGTTCCCGCCGTACGGCCGTGACGCCGACGGCGATGGAGATCATGACCACCTCTATCCAGGTACTGGAAAACCGTCTTAAACGCAATCGTATGGCGGGTGACCCGCCGGATATCCTGTTGCAGCCATACTGTCCGCAAGTTTCGACGCTTGATTTTCATCGCGCCGGAGAGGCGATTGCCGCGGGGCAACTGGCGGTAGAAAAGAAAATGGATGAATTATTACCCTTAGTGCGTACAGCCGTTTGA

>IR1230_00145

TTGTCGCAATTATGCCCCTGTGGCAGCGCACTGGAGTATAGCTCATGTTGCCAGCGATATCTGTCCGGCGCTGAGCTTGCGCCGGGCCCGTCACAGCTTATGCGCTCCCGTTATAGCGCGTTCGTCATGAAAGACGCTGACTATCTAATCAAAACCTGGCACCCGTCCTGTCAGGCGCAGCAATTTCGCGCCGAGCTGGAGAAAGGGTTTTCCCAGACCGAATGGCTGGGCTTAACCCTGTTCGCCAGTGATGAGGGGCGAGCTCCAAATGAAGGTTTTGTCAGCTTTGTCGCCCGCTTTAATGACAATAACCGCCCTGGCGCCATTATTGAACGTTCCAGATTCTTAAAAGAAAATGGACAGTGGTACTATATCGACGGGACACGACCGCTGATTGGCCGTAACGACCCCTGCCCCTGCGGCTCAGGTAAAAAATTTAAAAAATGCTGCGGCCAGTAA

>IR1230_00146

ATGCATTCATTACAACGTAAAGTACTACGCACTATTTGCCCTGATCAGAAAGGCCTCATCGCGCGTATCACCAATATCTGCTACAAGCATGAACTCAACATTGTGCAGAACAATGAGTTTGTTGACCACCGTACCGGTCGTTTCTTTATGCGCACCGAACTGGAAGGCATTTTTAACGACGCTACTCTGCTGGCCGATCTCGACAGCGCCCTGCCTGAAGGCTCCATCCGCGAACTAAATCCGGCAGGCCGTCGCCGGGTGGTGATTCTGGTGACGAAAGAGGCGCACTGTCTGGGCGATCTGCTGATGAAAGCCAACTACGGCGGGCTGGATGTCGATATTGCCGCGGTTATCGGCAACCATGACACCCTGCGCTCGTTGGTGGAGCGCTTTGGTATTCCGTTTGAGCTGGTCAGCCATGAAGGACTGAGCCGCGAAGAACACGATCAGCGGATGGGCGATGCTATCGCCGCCCACGAACCGGATTACGTGGTGCTGGCCAAGTATATGCGCGTCCTGACGCCAGAGTTTGTCGCCCGCTTCCCGAACAAAATCATCAATATCCACCATTCGTTTTTGCCGGCGTTTATCGGCGCCCGTCCCTATCATCAGGCCTATGAGCGCGGCGTGAAAATCATTGGCGCTACCGCCCACTACGTGAATGACAATCTGGATGAAGGCCCGATCATCATGCAGGACGTGATTCACGTCGATCATACTTATACCGCAGAAGATATGATGCGCGCTGGGCGCGATGTTGAAAAAAACGTCTTGAGCCGGGCGCTGTATCAGGTACTGGCGCAGCGCGTCTTCGTTTACGGTAACCGGACGATTATTCTTTAA

>IR1230_00147

GGTGGGGTTCCCGAGCGGCCAAAGGGAGCAGACTGTAAATCTGCCGTCATCGACTTCGAAGGTTCGAATCCTTCCCCCACCACCA

>IR1230_00148

GGTGGGGTTCCCGAGCGGCCAAAGGGAGCAGACTGTAAATCTGCCGTCATCGACTTCGAAGGTTCGAATCCTTCCCCCACCACCA

>IR1230_00149

ATGAATGACGTACTTGATGAAATTGACCGGGCGATCCTCACCTGCCTGACTCAGGATGCCAGAGTTTCGCTCAAGGTCCTGAGCGCGCGGGTGGGGCTGACCTCGCCGAGCACCGCCGAGCGGGTGAAGCGCCTGGAGGAGCGGGGTGTCATTCAGGGCTATGGGGCGCGGGTAAATCTCGCCGCGCTGGGCTATAGCCTGCAGGCGCTGGTGCGGGTCCGTCCGCTGCCTGGGCTGCTGCAGAAGGTGGATAAATATATCCAGGCGATGCCGGAATGTATTGAAAGTGACAAGGTGACCGGGGAAGATTGTTTTGTGATGCGTCTCGTGGTGCGCGATATTGCCCAGCTGGATACCCTGCTGGACGGTCTGGCGGAGTATGCGCAGTGTAACACCTCGGTGGTGAAAAGCTCGCCGGTAAAAAGGCGGTTGCCACCGCTGTGA

>IR1230_00150

ATGCAACTTACCCGCGGCGTCTGGCAGATGAGCCTGGCAATGATAATCTCCGGCTCGATCGGCGCGTTCGTGCTGCTCTCTGGTTTGCCGGTGACTGATGTGGTGTTCTGGCGCTGCCTGATTGGCGCGCTAACCCTGCTGGTGTTTATCGTGTTAAGCCGCCAGCCCTTCAGCCGGCTAACGCGTTTCACTCTCGCTCTGGCGGTGATTGGCGGCGCGGCGCTGGTCGTCAACTGGCTGCTGCTGTTTGCCGCCTATTCGCGGATCTCAATCGGTATGGCCACCGTGGTCTATAACACCCAGCCGTTTATGCTGGTACTGATGGGGATGGTGCTGGGGGAACGGGTCAGCGCCGTGAAATGGGGATGGTTACTGCTGGCCTTTGGCGGCGTAGTGATCCTGCTGTCGAGCGAGCTGACGCCTGCACACGAAGAGGGGCTTACCACCGGCGTGTTGCTGGCGCTGGGCGCGGCATTCTTCTATGCCCTGACCGCCATCATCGCGCGCAAACTGCATCCGCTCCCCGCGCAGCATATCGCCTTCATTCAGGTTCTCGTCGGCGTCGTGATGCTGCTGCCGCTGGTGCATGCCCCAGAATTGACGGCCAGCTTCCCCTGGCGCTATCTGCTGATCCTCGGCATCGTCCATACCGGGATCATGTATCAGCTTTTATATAGCGCGATACAGAAACTGCCGACGCCCGTTACCGGTTCGCTCTCCTTTATTTATCCGCTGGTGGCGATGGTGGTTGACTATCTGGTCTTCCACCATGCGTTATCCGCGGTGCAATTGCTCGGTGGGATGCTTATTCTGTTTGCCGCCGCAGGGAATAATCTCGGCTGGGGCGAAAAAAAACCCCGCCGCGGCGGGGTCAGTGAGCAGTCGCGGGCCAGTTAG

>IR1230_00151

ATGCACTTCCTGAATATGTTCTTCTTTGACATTTATCCGTACATTGCGGGTTCGGTGTTCCTGATTGGCAGCTGGTTGCGCTATGACTATGGCCAGTACACCTGGCGCGCCGCCTCCAGCCAGATGCTGGACCGTAAGGGGATGAACCTGGCGTCGAACCTGTTCCATATCGGCATCCTGGGTATTTTCGCCGGCCACTTCCTCGGCATGCTGACCCCGCACTGGATGTATGAGTCCTTCCTGCCGATCGACGTCAAGCAGAAGATGGCGATGATCGCCGGCGGCGCCTGCGGCGTGATGACCCTGGTGGGCGGGTTACTGCTGCTCAAGCGTCGTCTGCTGAGCCCGCGCGTTCGCGCCACCACCACCGGAGCGGATATTCTGATCCTCTCGTTGCTGATGGTGCAGTGTGCGCTCGGCCTGCTGACGATCCCGTTCTCCGCTCAGCATATGGACGGCAGTGAAATGATGAAACTGGTCGGCTGGGCGCAGTCGGTGGTGACGTTCCACGGCGGCGCGTCACAGCATCTGGATGGCGTGGCGTTTATTTTCCGCGTTCACCTGGTGCTGGGCATGACCCTGTTCCTGCTGTTCCCGTTCTCCCGTCTGGTTCATATCTGGAGCGCGCCGGTCGAATACCTGACGCGCAAATACCAGATTGTCCGCGCCCGTCGCTAA

>IR1230_00152

ATGATTGAACTCGTGATTGTATCGCGTCTGCTCGAATATCCTGACGCTGCCTTATGGCAGCATCAGCAGGAGATGTTCGAGGCGCTCGCGTCATCAGAGAAACTCAGCAAAGAGGATGCCCACGCGCTGGGCGTTTTCCTGCGCGATTTAGTGGCTCAGGATCCGCTGGATGCCCAGGCGGCCTATAGCGAACTGTTTGACCGCGGCCGGGCCACCTCGCTGCTGCTGTTTGAACATGTCCACGGCGAATCCCGCGACCGCGGGCAGGCGATGGTCGACCTGATGGCGCAGTACGAGCGCCACGGTCTGCTGCTGGATAGCCATGAGCTGCCGGATCATCTGCCGCTATATCTGGAGTATCTGGCGCAGCTGCCGGAAGAGGAAGCGCTTGGCGGCCTGCGGGACGTCGCGCCGATCCTCGGTCTGCTCAGCGCGCGCCTGCAGCAGCGCGAAAGTCGCTATGCGGTGCTGTTCGAGCTATTGCTGAAGCTGGCCAATACGCAGGTCGACAGCCAGAAGGTGGCGGAGAAGATTGCCGACGAAGCGCGCGACGATACGCCGCAGGCGCTGGACGCCGTCTGGGAAGAAGAACAGGTCAAATTCTTTGCCGACCAGGGCTGCGGCGAGTCGGAAATTTCCGCTCACCAGCGTCGTTTTGCCGGAGCCGTGGCCCCGCAATATCTGAATATCTCTAACGGAGGACAGCAATAA

>IR1230_00153

ATGAAAATTCGTTCACAAGTCGGCATGGTGCTGAATCTCGATAAGTGCATCGGCTGCCACACCTGCTCGGTGACCTGTAAAAACGTCTGGACCAGCCGGGAAGGGATGGAATACGCCTGGTTCAACAACGTGGAAAGTAAGCCGGGCGTCGGTTTCCCGAACGACTGGGAAAACCAGGAAAAATGGAAGGGCGGCTGGATCCGCAAAATCAACGGTAAACTGCAGCCGCGCATGGGCAATCGCGCGATGCTGTTGGGAAAAATCTTCGCCAACCCGCATCTGCCGGGCATCGATGATTACTACGAGCCGTTTGATTATGACTACCAGAATCTGCATAACGCGCCGGAGAGCAAACATCAGCCGATCGCCCGTCCGCGCTCGCTGATCACCGGTCAGCGGATGGACAAGATCACCAGCGGTCCGAACTGGGAAGAGATCCTCGGCGGCGAGTTTGAAAAACGCGCCAAAGATCAGAACTTCGACAACATGCAGAAGGCGATGTACGGCCAGTTCGAAAACACCTTCATGATGTATCTGCCGCGCCTGTGCGAGCACTGCCTGAACCCGGCCTGCGTCGCGACCTGCCCGAGCGGCGCCATCTATAAGCGTGAAGAAGACGGCATTGTACTGATCGACCAGGACAAGTGCCGCGGCTGGCGGATGTGTATCACCGGCTGTCCGTACAAGAAGATCTATTTCAACTGGAAGAGCGGGAAATCCGAGAAATGCATCTTCTGCTACCCGCGTATTGAATCCGGGATGCCGACGGTGTGCTCCGAAACCTGCGTGGGCCGTATCCGCTATCTCGGCGTCCTGCTATACGACGCGGACGCGATTGAAAACGCCGCCAGCACCGAGAACGAGAAAGATCTGTATCAGCGTCAGCTGGACGTGTTCCTCGATCCCAACGATCCGAAAGTGATCGAGCAGGCGCTGAAAGATGGCGTACCGCAGGGGGTCATTGAAGCTGCGCAGCAGTCGCCGGTTTACAAAATGGCGATGGACTGGAAGCTGGCCCTGCCGCTGCACCCGGAATATCGCACCTTGCCAATGGTCTGGTACGTGCCGCCGCTGTCGCCGATTCAGTCCGCCGCCGATGCGGGTGAACTGGGTAGCAACGGGATCCTGCCGGACGTGGAAAGCCTGCGTATTCCGGTGCAATATCTGGCGAACCTGCTGACCGCGGGCGATACCCAGCCGGTACTGCTGGCGCTGAAACGAATGCTGGCGATGCGCCACTATAAGCGTGCGGAAACCGTTGACGGCGTCGTCGATACCCGCGCGCTGGAAGAGGTGGGGCTGAGCGAAGCGCAGGCCCAGGAAATGTATCGCTATCTGGCGATCGCCAACTACGAAGATCGTTTCGTGGTGCCGAGCAGCCATCGTGAACAGGCGCGAGAAGCGTTTCCGGAGAAAAACGGCTGCGGCTTTAGCTTTGGCGATGGCTGCCACGGCTCTGACAGTCAGTTCAACCTGTTCAACAGCCGTCGCATCGACGCCATCGACGTGACCAGCAAAACGGAGCCGCACGCATGA

>IR1230_00154

ATGAGTAAATTCCTGGACCGGTTTCGCTACTTCAAACAGAAGGGTGAAACCTTTGCCGATGGGCACGGCCAGCTTCTAAACACCAACCGGGACTGGGAGGATGGATACCGTCAACGTTGGCAGCATGACAAAATTGTGCGTTCCACTCACGGTGTCAACTGCACCGGCTCATGCAGCTGGAAGATTTATGTCAAAAACGGTCTGGTGACCTGGGAAACGCAGCAGACTGACTATCCGCGCACCCGCCCGGACCTGCCGAACCACGAACCACGCGGTTGCCCGCGCGGCGCCAGCTACTCCTGGTATCTGTACAGCGCTAACCGCCTGAAATACCCGCTGATGCGTAAACGCCTGATGAAGATGTGGCGGGAAGCGAAAGTTCAGCATAGCGATCCGGTTGAGGCATGGGCGTCGATTATCGAAGACGCCGACAAAGCTAAAAGCTTCAAACAGGCCCGCGGTCGCGGCGGTTTTGTACGTTCCTCCTGGCAGGAAGTGAATGAGCTGATTGCCGCCTCCAACGTCTATACCGTCAAAACCTACGGTCCGGACCGCGTGGCTGGCTTCTCGCCGATCCCGGCGATGTCGATGGTCTCCTACGCGTCCGGCGCCCGTTATCTGTCGCTGATCGGCGGCACCTGCCTGAGCTTCTACGACTGGTACTGCGACCTGCCGCCGGCTTCGCCGATGACCTGGGGCGAGCAGACCGATGTGCCGGAATCCGCCGACTGGTATAACTCCAGCTATATCATCGCCTGGGGCTCCAACGTGCCGCAGACCCGCACCCCGGATGCGCACTTCTTTACCGAAGTCCGCTATAAAGGGACCAAAACCGTAGCCATCACTCCGGACTACGCCGAAATCGCCAAACTCTGCGATCTGTGGCTGGCGCCGAAGCAGGGTACCGATGCCGCGATGGCGCTGGCGATGGGCCATGTGATGCTGCGTGAATTCCACCTCGATAAGCCGAGCCAGTATTTCACCGACTATGTGCGCCGCTATACCGACATGCCGATGCTGGTGATGCTCGAGGAGCGCGACGGTTATTATGCCGCCGGCCGTACGCTGCGCGCATCCGACCTCGTGGATTCCCTTGGTCAGGAGAATAATCCGGAATGGAAAACCGTCGCCTTTGACGAGAAGGGCGACATGACCGTACCGAACGGTTCCCTCGGATTCCGCTGGGGCGACAAAGGCAAATGGAACCTCGAACAGCGCGACGGTAAAACTGGCGAAGAGATTGAGCTACGTCTGAGCCTGCTGGGCAGCCATGATGAAGTGGCGAACGTCGGCTTCCCGTACTTCGGCGGCGAAGGGTCTGAGCACTTTAACAAAGTCGATCTGGAAAACATTCTGCTGCACAAACTGCCGGCCAAACGCCTGCAGCTGGCCGACGGCTCTACCGCCCTGGTCACCACCGTGTATGACCTGACCATGGCTAACTACGGCCTGGAGCGCGGTCTGAACGATGACAACTGCGCCGCCGGCTACGATGAAGTGAAAGCCTACACCCCGGCGTGGGCGGAGAAGATCACCGGCGTCTCCCGCGCGCATATCATCCGTACCGCCCGTGAATTTGCCGATAACGCCGATAAAACTCACGGTCGTTCGATGATTATCGTCGGGGCGGGCCTCAACCACTGGTTCCACCTCGACATGAACTACCGCGGCCTGATCAATATGCTGATCTTCTGCGGCTGCGTCGGTCAGAGCGGCGGCGGTTGGGCGCACTATGTGGGCCAGGAAAAACTGCGTCCGCAGACCGGCTGGCAGCCGCTGGCGTTCGCCCTTGACTGGCAGCGTCCGGCACGTCACATGAATAGTACCTCGTACTTCTATAACCACTCCAGCCAGTGGCGCTATGAAACGGTGACCGCGCAGGAACTGCTGTCGCCGATGGCGGATAAATCCCGTTACAGCGGCCACCTGATCGACTTTAACGTGCGCGCAGAGCGGATGGGCTGGCTGCCGTCGGCGCCGCAGCTTGGCGTCAACCCGCTGCGTATCGCTGACGAAGCGAAAAAAGCCGGCATGACCCCGGTGGATTACACCGTGAAGTCGCTGAAAGAGGGCTCGATTCGCTTTGCGGCCGAGCAGCCGGAAAATGGCAAAAACCACCCGCGTAACCTGTTTATCTGGCGTTCCAACCTGCTGGGCTCCTCCGGTAAAGGCCATGAATACATGCTCAAGTACCTGCTGGGTACCGAGAACGGCATTCAGGGCAAAGACCTGGGCAAGCAGGGCGGCGTGAAGCCGGAAGAAGTCGAATGGCGGGATAACGGCCTCGACGGCAAACTGGATCTGGTGGTGACCCTCGATTTCCGTCTGTCGAGCACCTGTCTCTACTCCGACATCGTGCTGCCGACCGCCACCTGGTACGAAAAAGACGACATGAATACCTCGGATATGCATCCGTTTATTCACCCGCTGTCGGCCGCCGTTGATCCAGCCTGGGAATCGAAGAGCGACTGGGAGATCTATAAAGGTATCGCGAAGAAATTCTCTGAAGTGTGCGTGGGCCACCTCGGTAAAGAAACCGACGTGGTGACCCTGCCGATCCAGCACGACTCCGCCGCGGAAATGGCGCAGCCGCTGGATGTCAAAGACTGGAAAAAAGGCGAATGCGATCTCATCCCCGGGAAAACCGCGCCGCATATTATTCCGGTTGAGCGTGATTACCCGGCGACCTACGAGCGTTTCACCTCGATCGGCCCGCTGCTGGAAACCATCGGCAACGGCGGGAAAGGCATCGCCTGGAACACCCAGAGCGAGATGGATCTGCTGCGTAAGCTCAACTACACCAAGGCGGAAGGCCCGGCGAAAGGCCAGCCGAAGCTGGAAACGGCCATCGACGCCGCGGAAATGATCCTCACCCTGGCCCCGGAAACCAACGGTCAGGTGGCGGTGAAAGCGTGGCAAGCGCTGAGCGAGATCACCGGTCGCGAGCATACGCATCTGGCGCTGAACAAAGAAGACGAGAAGATCCGCTTCCGCGATATTCAGGCGCAGCCGCGGAAGATTATCTCCAGCCCGACCTGGTCTGGCCTGGAAGATGAGCATGTCTCCTATAACGCCGGTTACACCAACGTTCACGAGCTGATCCCGTGGCGCACGCTGACCGGACGTCAGTCGCTGTATCAGGATCACCAGTGGATGCGCGACTTCGGCGAAAGCCTGCTGGTCTATCGGCCGCCGATCGACACCCGTTCGGTGAAAGCGGTGATGGGCGAGAAGTCCAACGGCAACCCGGAAAAGGCGCTGAACTTCCTGACCCCGCACCAGAAATGGGGTATCCACTCGACCTACAGCGATAACCTGCTGATGCTGACCCTGTCGCGCGGTGGGCCGATTGTCTGGATGAGCGAAGCGGATGCGAAAGATCTGGGTATCGAAGATAACGACTGGATCGAAGTGTTCAACGCCAACGGCGCCCTGACGGCGCGTGCGGTGGTGAGTCAGCGCGTTCCAGCCGGGATGACCATGATGTATCACGCGCAGGAACGTATCGTGAACCTGCCGGGCTCTGAAATCACCGGTCAGCGCGGGGGGATCCATAACTCCGTCACCCGTATTACGCCGAAACCGACCCACATGATCGGCGGCTACGGCCACCTGGCCTATGGCTTTAACTACTACGGCACCGTCGGCTCCAACCGCGATGAGTTTGTGGTGGTACGTAAGATGAAGAACATTAACTGGTTAGACGGCGAAGGCAACGACCAGGTACAGGAGAGCGTAAAATGA

>IR1230_00155

ATGAGTCAATCTTCCCTCCCGGAGAAGGCTAACCGCTCGGTCATAACCGACTGGCGGCCTGAAGATCCTGAATTCTGGCAACAGCGCGGCCATCGTGTGGCAAGCCGCAATTTATGGATCTCCGTTCCCTGTCTGTTACTGGCGTTCTGCGTTTGGATGTTGTTCAGCGCGGTTGCCGTTAACCTCAACAAAGTAGGCTTTCAGTTTACAACCGATCAGCTGTTTATGCTGACCGCGCTGCCGGCGCTGTCAGGCGCCTTATTACGCGTGCCTTACGCCTTTATGGTGCCGCTGTTCGGCGGCCGTCGCTGGACGGCGTTCAGCACCGGGATCATGATTGTGCCATGCGTCTGGCTGGGCTTCGCGGTCCAGGATACCTCTACGCCGTTTAGCATCTTCGTGATTATCTCTCTGCTGTGCGGCTTCGCCGGAGCCAACTTTGCCTCCAGTATGGCCAACATCAGCTTCTTCTTCCCGAAAGCGAAGCAGGGCGGGGCGCTCGGCGTTAACGGCGGACTTGGCAATATGGGCGTCAGCGTGATGCAGCTGGTCGCGCCGCTGGTGGTCTCGATCTCTATCTTTGCCGTTTTTGGCGGTAACGGTAGCGAGCAACCGGACGGCTCGATGCTGTATCTGGAAAACGCCGCGTGGATCTGGGTACCTTTCCTGATTATTTTCACTCTGGCGGCGTGGTTCTTTATGAACGACCTGTCGGCTTCGAAAGCGTCGCTGAGCGAGCAGTTGCCGGTACTTAAGCGCCTGCATCTGTGGATTATGGCGCTGCTCTATCTCGCGACCTTCGGCTCCTTTATCGGCTTTTCCGCCGGGTTCGCGATGCTGTCAAAAACTCAGTTCCCGGATGTGCAGATCCTGCATTACGCCTTCTTCGGCCCGTTCATTGGCGCGCTGGCGCGTTCGATGGGCGGGGCGATCTCCGACCGCCTCGGTGGGACCCGCGTAACGCTGGTCAACTTTGTGGTGATGGCTGTCTTCTGCGCGCTGCTGTTCCTGACCCTGCCGACCAACGGCCAGGGCGGTAACTTTATCGCCTTCTTCGCGGTGTTTATGGTGCTGTTCCTGACCGCCGGGCTGGGGAGCGCCTCCACCTTCCAGATGATCTCCGTGATCTTCCGTAAGCTGACTATGGACCGCGTGAAGGCCCAGGGCGGCAGCGAAGCGCAGGCGATGCGCGAGGCGGCGACCGATACCGCGGCGGCGCTGGGCTTTATCTCCGCCATTGGCGCTATCGGCGGCTTCTTTATTCCGAAAGCGTTCGGTATCTCGCTGGATCTGACCGGCTCGCCGGCCGGGGCGATGAAAGTGTTCCTCGTGTTCTATATTGCCTGCGTGGTGATCACCTGGGCGGTATACGGTCGTAAGCGTCAGTAA

>IR1230_00156

ATGTTAAAACGTCTTTTTACCCCGCTGACGCTGGTCAATCAACTGGCGTTAATCGTGCTGCTCGCCACGATTATTGGCGTCGCCGGCATGGCGATCTCCGCGCGCCTGGTGAACGGCGTGCAGGGCAGCGCGCACGCCATTAACAAGGCCGGTTCATTGCGTATGCAAAGCTACCGGCTGCTGGCGGCGATCCCGCTGAATGAAAACGATCAAAAACTGGTGGCGGACATGACCGCCACCGTCTTCAGCCCGGAGCTGCAAAATTCCGCCCGCCGCGACGGCCAGGAAATCCAGCTTAAAGCCCTGCAGCAGTACTGGCAGCTGGCGCTGGCCCCCGGCATGCAGCGCGCAGTCAATCAGGCGGAAGTGGCCCAGGATGTCGCCGACTTTGTCGATCGTATCGACCAGTTGGTCACCGCATTCGACCACACCACCGAGCAGCGTATCGAACGGGTGGTCTGGATCCATCGCATCCTCGCCATTGGTATGGCGCTGCTGCTGATCTTCACTATCATCTGGCTACGCGCCCGTCTGCTGCGGCCGTGGAAACAGCTGCTGAGCATGGCGCGCGCGGTGAGTCAGCGTGATTTCACCCAGCGGGCGCACATCAGCGGCCGCAACGAAATGGCGACCCTGGGCATGGCGCTGAATAACATGTCGGAAGAGCTGGCGGAAAGCTACGCCGTGCTCGAGCGGCGGGTGCAGGAGAAGACTGCCGGACTGGAGCAGAAGAACGAGATCCTCGCTTTCTTATGGCAGGCCAACCGCCGGCTGCACTCCAGCGCCCCGCTCTGCGAACGTATTTCGCCGGTACTGAATGGCCTGCAGGGGCTGACGCTGCTGCGCGATATCGAGGTACGGGTTTACGACCTGGAAGACGAAGATAATCACCAGGAGTTTACCTGCCACTCGGATGATGACTGTGATGATAAAGGCTGCTACCTCTGTCCACGCAATCTGCCGCCGCTGCCGGATGGGGGAACAACGCTGAAGTGGCGGCTGTCAGATGCGCATAGTCAGTACGGCATCCTGCTGGCGACGCTGCCGGTTGGCCGCCATCTCAGCCACGATCAGCAGCAGCTGGTGGATACCCTGGTGGAACAGCTGACCAGCACCCTGGCGCTGGACCGCCATCAGGAGAAGCAGCAGCAGCTGATCGTCATGGAAGAACGGGCCACTATCGCCCGCGAGCTGCACGACTCCATCGCCCAGTCGCTATCCTGTATGAAGATGCAGGTTAGCTGTCTGCAGATGCAAGGCGACGCCTTGCCGACGGAAAGCCGCCAGCTGCTGGGGCAGATCCGTAACGAACTGAATACCTCGTGGGCCCAGCTGCGGGAGCTGTTAACCACCTTCCGCCTGCAGTTGACCGAGCCGGGGCTGCGCCCGGCGCTGGAGTCCAGCTGTCAGGAGTACAGCGCCCACTTCGGCTTTACGGTGCAGCTTGATTACCAGCTCCCGCCGCGCTTTGTGCCCTCGCATCAGGCGATCCACCTGCTGCAGATCGCTCGCGAAGCGTTGAGCAACGCTCTGAAGCATGCCAGCGCGACCGAAGTGACCGTGACGGTGTCACAACGCGATAATCAGGTTCGTCTGGTGGTAGCGGATAACGGCCGCGGCGTACCGGACCACGCCGAACGGAGTAACCATTACGGTTTAATTATTATGCGGGATCGCGCCCAGAGCTTGCGCGGTGACTGTCAGGTACGACGCCGGGAAACCGGTGGAACCGAAGTGATCGTCACGTTTATCCCGGAAAAAACGTTTTCCATCCAATAA

>IR1230_00157

ATGAGTCAACAGGAACGGGCAACTATCCTTCTCATTGACGACCATCCGATGCTGCGCACCGGCGTGAAACAGTTGATCAGCATGGCGCCGGACATCCAGGTGATTGGCGAGGCCAGCAATGGCGCGCAAGGTATTGAGCTGGCCGAGTCGCTGGATCCTGACCTGATCCTGCTGGACCTTAATATGCCGGGCATGAACGGTCTGGAGACGCTGGATAAGCTGCGTGAGAAATCCCTTTCTGGCCGGGTAGTGGTCTTCAGCGTCTCCAACCATGAAGAGGACGTTGTCACCGCCCTGAAGCGCGGCGCCGATGGCTATTTGCTGAAGGACATGGAACCGGAAGATCTGCTGAAAGCGCTGCAGCAGGCGGCCGCCGGAGAGATGGTATTAAGCGAAGCGTTAACGCCGGTGCTGGCCGCCAGCCTGCGCGCCAACCGCGCCACCTCCGATCGGGACATCAGCCAGCTGACCCCGCGCGAGCGCGACATTCTCAAGCTGATAGCCCAGGGACTGCCGAACAAAATGATCGCCCGTCGCCTGGATATCACCGAAAGCACCGTCAAAGTACACGTAAAACACATGCTGAAGAAAATGAAGCTCAAATCCCGCGTGGAAGCGGCGGTGTGGGTCCACCAGGAGCGAATCTTCTAA

>IR1230_00158

ATGCCTGTGTCGTTCCGTTTGTTGCCAACGTTGACCTTTCTCCTGCTGTTGCCGGGCGTTCCCGTCTGGGCGCTGACCGCCAGCGACACCACCCGCCCTGCGCAAGCGCAAGATCCGCTGCCCGATATGGGGATTGCTCCCCAGGTTGATGACGACGCCCGGCACTTTGCCGAAGTGGCGAAGAAGTTTGGCGAGGCCAGCATGAGCGACAATGGTCTGACGGCGGGTGAACAGGCGCAGCTGTTTGCCATCAGTAAAATCGGCAATGAAGTGAGTCATCAGCTGGAGAGTTGGCTCTCGCCGTGGGGCAACGCCAATGTCGACCTGCTGGTCGATAAAGAGGGGAAATTCACCGGCAGTAAAGGGAGCTGGTTCGTGCCGTTGCAGGATAACGACCGTTACCTGACCTGGAACCAGTACTCGGTGACCCGGCGCGAGCACGATCTGGTGGGCAACATCGGGCTTGGCCAGCGTTGGCGGGTCGGCGGTTGGCTGCTGGGCTATAACTCGTTTTACGATAAAGTGCTGAGCGAAAGCCTGGCGCGCGGCAGCGTTGGCGCCGAAGCGTGGGGAGAATATCTCCGCCTGTCGGCAAACTATTACCATCCGCTGGGCGACTGGCAGCTACGCGATAATCAGACTCAGGAGCAACGGATGGCCGCAGGGTATGACGTCACCGCCCAGGCGCGGCTGCCGTTCTACCAGCACATCAACACCAGCGTCAGCGTCGAACAATATTTCGGCGATAGCGTCGATCTGTTTCACTCCGGCACCGGCTACCATAATCCGGTGGCCGTCTCCGTTGGGCTCAATTACACCCCGGTCCCGCTGGTCACGGTGACCGCTAAGCATAAGCAGGGGGAAAATGGCGTCAGCCAGAACAATGTGGGCCTGAAGCTAAACTACCGCTTTGGCGTACCCCTTAAGCAGCAGCTTGCGGCAGACGAGGTGGCGATCAGCAACTCGCTGCGCGGGAGCCGCTTCGATAGTCCGGAGCGGGATAATCTGCCGGTGGTGGAGTATCGTCAGCGCAAAAATCTGACGGTCTATCTGGCCACGCCGCCCTGGGATCTGCAGTCCGGGGAGACGGTGCAGTTGAAGCTGCAGATCCATAGTCTGCATGGCATCAAGGCGCTGCACTGGCAGGGAGACACCCAGGCCCTCAGCCTGACGCCGCCGGTAGATGCCAGCAGCCCCGATGGCTGGAGCATCATTATGCCGGTGTGGAACAGTGAACCGGGCGCCGCGAACCGCTGGCGCCTGTCGGTGGTGGTGGAGGATAAACAGGGGCAGCGCGTCTCCTCCAATGAGATCGCCCTGGCGCTGACCGAGCCGCTGGTCAAGTTTACCACGCCAGGCGTCTCCTGGACGGACTCGCCTTAG

>IR1230_00159

ATGAAGACCATCCGAACGACCTGCCCCTACTGCGGCGTAGGCTGCGGGGTGCTGGCGAGCGTGGACGACGCGGGGCAGGTGAGCGTGCGTGGCGACGATCAGCACCCGGCGAACCTCGGCCGTCTGTGCGTCAAAGGGGCCGCCTTAGGGGAGACCACGGGCCTGGCGGGGCGGCTGCTGACGCCGGAGGTCGACGGCCAGCAGGTAGCCTGGCCTCAGGCGCTGGCGGAAACCGCTGCGCGCCTGAGACAGATTATCGACCAGCACGGACCGCAAGCGGTGGCCTTTTACGCCTCCGGCCAGCTGTTAACCGAGGATTACTATGCCGCCAATAAGCTGATGAAAGGGTTTATCGGCGCGGCGAACATCGATACCAATTCACGGCTGTGCATGTCGTCAGCGGTCACTGGCTACAAGCGGGCCTTTGGCGCCGACGTGGTGCCCTGCAGCTACGATGATGTGGAAAACAGCGATCTGGTGGTGCTGGTCGGCTCGAACGCCGCCTGGGCGCATCCGGTGCTGTTCCAGCGGCTGGCGCAGGCGAAGCGGGATAATCCCCGATTGCGGATCGTGGCGATCGACCCCCGACGCACCGCTACCTGCGAGATCGCCGACCGCCATCTGGCGCTGGCCCCGGGAAGCGACGGCGGGTTGTTTGCGGGGCTGCTTAACGCCCTGGCTGAGGCAGGAGCCTGCGTCGATGGCTTTCGCGACGGCCCGCAGGCGCTGGCCGCCGCCCGCGGCTGGGACGTGGCGAGGGTGGCGGCCTTTTGCGGTCTGCCTGCCGATGAGGTGGCGGGATTCTATCGCGAGTTCATCGCCGCCCCGCGGGCGATCACCCTCTACACCATGGGAATCAATCAATCCGCCAGCGGCAGCGACAAATGTAACGCCATCATCAACGTCCATCTGGCCAGCGGGAAGTATGGCCGCCGCGGCTGCGGCCCCTTTTCGCTGACCGGTCAACCCAACGCCATGGGCGGCCGCGAAGTCGGCGGGCTGGCGACGATGCTGGCGGCGCACATGGATTTTGTGCCAGACGATCTGCAGCGGCTGGCCCGCTTCTGGGGGACGGAACGGCTGGCGCAGACCCCCGGGCTGACCGCCGTGGAGCTGTTTGCCGCCATTGGCCGCGGCGAGGTCAAAGCGGTCTGGATCATGGGTACCAACCCGGTGGTGTCGCTCCCTGACAGCCACGCGGTGAGCCAGGCGCTGGCCGCCTGTCCGCTGGTGATCGTCTCGGATGTCGCCGCCCAGACCGATACCGGACGTTTCGCCCACATCCGCTTTCCGGCGCTGGCCTGGGGCGAGAAGAATGGCACGGTCACCAACTCGGAGCGGCGGATATCCCGTCAGCGCAGCTTTCTGCCGCCGCCGGGGGAGGCGAAAGCGGACTGGTGGATCATCGCCAGAGTGGGCCAGGCGCTGGGGTATCGCGAGGCGTTTGCCTGGCAGCATCCGCATGACGTCTTCCGCGAGCATGCGGCGCTGTCCGGTTTCGAAAACGACGGCCAGCGGGCGTTTGATATCGGCGCGCTGGCGGATCTCAGCCGTGAGGCGTGGGACGCCATGCCGCCGGTGCGCTGGCCGGTCAGCCGCAGCGAGGTCGCCTGGGATATCACGCGCGGCTGGCATGGCGATGGCAGGCTGCGGATGGTGCCGGTGACGCCTCAACCGACCCGGGCGACAACCGATGCCTTTTATCCGCTGATCCTCAACAGCGGGCGGATCCGCGATCAGTGGCACACCATGACCCGTACCGGCGCAGTGCCGCGGCTGATGCAGCATATCGCGGAGCCAATGGTGGAGGTGGCGCCGCAGGATGCCGTTCGCTATCAGCTTCCCGCTGACGGGCTGGCGCGGATCTGGTCGCGGCATGGGGTGATGGTGGCGAAAGTGGCGATCAGCGAAGGGCAGCGCCCCGGCTCGCTGTTTGTTCCGATGCACTGGAACAACCAGTTTGCCCGTCAGGGGCGGGTGAATAACCTGCTGGCCGCCGTCACGGACCCGTATTCCGGCCAGCCGGAAAGTAAACAGGCGGCGGTGGCGATCGCCGCCTGGCAACCGGCCTGGCACAGCGAGCTTTTTTGCCGCGAACCGCTACCATTTCCTGCGGCCTGGCACTGGCGGCGGCGGGCGACGCCAGGCGTGCTGCACTACTCGCTGGCCGGGGAGGCATCTGCCCGACAGTGGCTCAGCGCGTGGTGCGCCCGGCGCGGCTGGCAGTTACAGGTGGCTGACGGCGGCGCGGTGTGGAACCTGCTGGCCTGGCATCAGGGAAGGCTGATGCTCGGCTGGTGGAGCGATGCGCGCGAGCCGGCGGTGGATTGCGCATGGATAAGCGCTGCCTTTGCCGCGCCGCCGTCCGATGCTGCCCAACGACATGCCTTGCTTAGCGGCCGACCGGGCGCCGCAGTCGCGCCGCGGGGGCGGATCGTCTGCAGCTGCTTTGGGGTGGGGGAATGGTCAATTAACGAGGCCATCGCCAGCGGCTGCGCCAGCGTCGGGGCGCTGGGTGGGAAGCTGAAATGCGGGACCAACTGCGGCTCTTGCGTGCCGGAGCTCAACGCGCTGCTGGCGGCGCAACGAACCCGGGCGTAA

>IR1230_00160

ATGACGCGGCGACTGGTGGTGATCGGTAACGGCATGGCGGCCACCCGGCTGGTGCAGCGGCTGGTTGAGCGCGACCCCGCGCGGTTTGCCATTACCGTCGTCGGCGACGAGCCGCACCCGGCCTATAACCGCATTCAGCTCTCGCCGCTGCTGGCAGGTGAAAAAACGGCGGCGCAGATCCCGTTGCTGCCGGCGGAGTGGTACACCCGGCACGGGGTGTGCCTGCGGTCTGGCGAGGCGGTCGATGAAGTCGATATTCAGCAACGGCGGCTGCGCATCGCGGAAACCTGGCTGCCGTGGGATGAGCTGGTGTTCGCCACCGGTTCGCGGCCCTTTATCCCGCCGCTGCCGGGCATTGATCGTCCGCAGGTTATGCCCTTCCGCACCCTGGCGGACGTGGAACGTATTCTGGCGATACCCGGCCCGGCGGTGGTGATTGGCGGTGGGGTGCTGGGGGTGGAAGCGGCGGCGGCGCTGCGTCGTCACGGCGGAGAGGTCACTCTTCTGCATCGCGGCAGCGGATTAATGGCGCCGCTGACCGACGCTTTTGCCGCCGATGAGCTCAGGCAGCAGCTTGAAGCGCGCGGCATTCGCTGCGTGCTGGAGTGCAGCATCGCCGCCATCGACGCCGATGGCGTGCGGCTGGCGGACGGGCGCGTATTCCGCGCCGCCAGAGTGGTGCTGGCTACCGGCGTGCAGCCCGACAGCCGTCTGGCGGCGCAGTCCGGCGTGCTGTGCCAGCGGGGGATCGTGGTCGACCGTCAGATGGCCTCCTCGCTGCCGGGGATCAGCGCCATCGGCGAATGTTGCGAAATTGACGGCCAGACCTGGGGGCTGGTGGCTCCCTGCCTGCGTCAGGCCGAGGTGCTGGCGGATCGCCTGTGCGGCGCCCCCGGCGAGGGCTTTGTCTGGCAGGACGCCGGGACCCGCCTGAAGGTCACCGGCATTGAATTCTTTTGCGCTGGCGAGCAGCAGGCCGGCGAACAGGATGACATTTATACCAGCTGGGACCCCATCGACCGCCACTATCGCCGCCTGCTGCTGCGCGATGGCCGCCTGCGCGGCGTGCTGCTGATGGGCGACTGCACCGCCGCCGCCGCGCTCACCGCGCGCCTGGAAAGCGATGAGCCTGCCACCGTGGACTGGCTTTTTGACCCCTCTTCAACGCAGCCGCAGGCTGCAGGAATAATGACGATGACGAAACCTGTATTAGTGCTGGTGGGACATGGCATGGTCGGCCACCATTTTCTCGAGCAATGCGTGAGCCGCAATCTGCATCAGCAATATCGAATCGTGGTGTTTGGCGAAGAGCGTTATCCCGCCTACGACCGGGTACATCTCTCCGAGTATTTTGCCGGACGCAGCGCCGAGTCGCTGTCGCTGGCGGCCGGGGACTTCTTTATTGAACATGGTATTGAACTCCGCCTCGGCGAGGCGGTGGCGACCATCGACCGCGAAGCACGGCTGGTACGGGACGCCGAGGGGCATGAAATCCACTGGGATAAACTGGTGCTGGCGACCGGCTCGTATCCCTTTGTTCCGCCAATCCCCGGTAACGATCTCGCGGGGTGTTTTGTCTACCGCACTCTTGACGATCTCGACCGTATCGCCGCCCATGCCGCCGCGGCGAAGAGCGGGGTGGTTATCGGCGGCGGGCTGCTCGGGCTGGAGGCGGCCAACGCCCTGAAACAGCTGGGGCTGGAGACGCAGGTGGTGGAGTTTGCCCCGAACCTGATGGCCGTCCAGCTGGATAACGGCGGGGCGGCGATGCTCCGGGAGAAGATCGTCGCCCTCGGCGTGGGGGTGCATACCAGCAAAGCGACCACCGCCATCGTGCGCGAGGCCGACGGCCTGCGGCTGAACTTTGCCGACGGCGGCGCGTTGCGGACCGACATGGTGGTCTTCTCGGCGGGGATCCGCCCGCAGGACGCGCTGGCCCGCGGCTGCGCGCTGCAGGTGGGCGAACGCGGGGGAATTCACATCGACGGCCAGTGCCGCACCTCTGACCCGGACGTGCTGGCGATAGGCGAATGCGCCCTGTGGGACAATAAAATTTACGGTCTGGTGGCGCCGGGCTATCAGATGGCGCGCATCGCCGCGGCCACTCTCGCAGGAGAGGACGCCTGCTTTAGCGGCGCCGACATGAGCACCAAACTGAAGCTGCTGGGAGTCGACGTGGCCTCGTTTGGCGATGCGCAGGGGCGCACGCCGGGCTGTCAGAGCTATCAATGGACCGACGGTCCGCAGCAGATTTACAAAAAAATCGTCGTCAGTCAGGACGGCAAAGCCCTGCTTGGCGGCGTGCTGGTGGGGGATGCCAGCGACTATGCCACGCTGCTGCAGATGATGCTGAACGGCATGGCGCTGCCGCCGCGTCCGGAGAGTCTGATCCTGCCCGCGCTGGAGGGAGCCGCGCCGAAGGCGCTCGGCGTCGCGGCGCTGCCGGACAGCGCGCCGATCTGCTCCTGCCATAACGTCAGCAAAGGCGATATCTGCCAGGCGGTGAACAATGGGGCGAGGGATATGTCGGCGATAAAAAGCTGCACCAGAGCGGCTACCGGCTGCGGCGGCTGCAGCGCGCTGGTGAAACAGGTGATGGAGTATCAGCTGGCGGAGCAGGGCGTCGAAGTGAAAAAAGACGTCTGCGAACATTTCCCGTGGTCGCGCCAGGAGATCTACCACCTGGTGCGGGTTAACCATATCCATACCTTCGAGCAGTTGATTAGCCGCTACGGCCAGGGGCACGGCTGCGATGTCTGCAAACCGCTGGTGGCCTCGGTGCTGGCCTCCTGCTGGAACGAGTACCTGCTGAAGCCGGCGCACCTGCCGCTGCAGGATACCAACGACCGCTATTTCGCCAATATCCAGAAGGACGGGAGCTATTCGGTGGTGCCGCGGATGGCGGCAGGGGAAGTGACGCCGGACGGACTGATCGCCATCGGGCAGATCGCCAAACGTTACCAGCTCTACAGCAAAGTGACCGGCGGCCAGCGCATCGACCTGTTTGGCGCGCGGCTGGAACAGCTGCCGGCTATCTGGCGCGAGCTGGCCGACGCCGGGTTTGAAACCGGCCACGCCTACGGCAAGTCGCTGCGCACGGTGAAATCCTGCGTCGGCTCAACCTGGTGTCGCTACGGCGTGCAAGACTCCACCGGGCTCGCGGTACGGCTCGAGCATCGCTATAAAGGGCTACGCGCCCCGCACAAAATCAAGATGGCGGTCTCCGGATGCACCCGCGAATGCGCTGAAGCCCAGGGGAAAGATATCGGGGTGATTGCCACCGACAAAGGCTGGAATCTGTACGTCTGCGGCAACGGCGGGATGAAACCGCGTCACGCCGACCTGTTTGCCAGCGATCTCGACGAAGCGACGCTGATCCGCAGCATCGATCGTCTGCTGATGTTCTATATCCGTACCGCCGATCGCCTGCAGCGAACCAGCACCTGGATGGATAACCTGGAGGGCGGCGTCGCCTATCTGCGGCAGGTGGTGCTGGAAGATAGTCTTGGCATTGGCGAGGAGCTGGAGCAGGAGATGGCCCGGATCGTCGACAGCTACCAGTGCGAGTGGCAGACCACCCTCAACGATCCGCAGCGTCTGGCGCTGTTCCGCTCCTTCGTTAATAGCGACCAGCCCGACGAGGCTGTGCAGCGCCGCGATCTGCGCGGTCAGCCGCAGCCGCTGCTGACTGAGACGTTGCCCGAAGGCGAACTCCCATCCCGACCGTGGCAGGCGGTATGCGATCTTGACGCCATTCCGGCGCAGGCGGGGATCGGCGCCCGCTTAGGGGAGCGGCAGATTGCCCTGTTCCGCTTCGGCGAGCGGGTCTACGCCCTCGACAACCGCGAGCCGGGCAGCGCGGCCAACGTGCTGTCGCGTGGGCTGTTAGGGGACGTCGGCGGCGAGCCGGTGGTGATCTCCCCGCTGTACAAACAGCGGATACGCCTGCGCGACGGCTGGCCGTGCGACGGCGACGAACAGGCGGTACGCGCCTGGCCGGTGAAAGTAGAAAACGGCAAAGTGTGGGTGGGCAACCAGCAGCTGCTGGCCCGCGCGGAGGCCTCCTGA

>IR1230_00161

ATGAAACCATTAATTCAGGTGCAGGCCGTCAGCCAACGCTTCAACACCGCCAGCGGCGAGTTCCTGGCGCTGCAGAATGTCTCCTTCGATATCGTCGAAGGGGAGACCATTAGCCTTATCGGCCACTCGGGTTGCGGAAAATCGACCCTGCTGAACCTGATCGCCGGGATCACCACCCCTACCGAGGGCGGACTACTGTGTGATAACCGGGAAATCGCCGGTCCCGGGCCGGAGCGGGCGGTGGTTTTCCAGAACCACTCGCTGCTGCCGTGGCTGAGCTGTTTCGACAATGTGGCGCTGGCGGTGGATCAGGTGTTCCGTCGCACCATGAGCAAGAGTGAGCGCCGGGAGTGGATCGAACACAACCTGGCGCGCGTACAGATGGGTCATGCGCTGCACAAACGTCCCGGGGAGATCTCCGGCGGCATGAAGCAGCGGGTGGGTATCGCCCGGGCGCTGGCGATGAAGCCGAAAGTGCTGCTGCTTGATGAGCCCTTCGGCGCCCTGGACGCGCTGACCCGCGCCCACCTCCAGGACACGGTGATGCATATCCAGCAGGAGCTCAATACCACCATCGTCATGATCACCCACGACGTTGATGAAGCGGTGCTGCTCTCGGATCGGGTGCTGATGATGACCAACGGTCCGGCGGCGACGGTCGGCGAGATCCTCTCGGTCGATCTGCCGCGTCCGCGCCACCGCGTTCAGCTGGCGGACGACAGCCGGTATCACCATCTGCGCCAGCAGATCCTCCATTTCCTGTATGAAAAACAGCCGAAAGCGGCGTAA

>IR1230_00162

ATGAAGCAGGCACAACGTAAGCAACCGGTCGTCAGCGTGGACAACGCACCTGGCGAAGTGATTATCCTGCCGCCGGTGCAGGTCCGGCGCACCACGCCGACCGTCACGCGCTGGCTGCGTGAGCTCACCCAGCGCCTGCTGCCCCCGCTGTTGGGGCTGGGAGTGCTGCTGCTGGCCTGGCAGCTGGCGGCGATGCATAGCAAAGGCTTTCCCACGCCGCTGAGCACGCTGGACTCGGCGCTGACCCTGTTTGCCGATCCGTTCTATCAGGACGGGCCCAATGACATGGGCATCGGCTGGAACGTGCTGGCCTCCCTGCAGCGCGTGGCGGTGGGCTTCGGCCTGGCGGCGCTGGCGGGCATTCCGCTGGGGTTTTTGATCGGCCGTTCGCTGTTCTTCGCCCGCATGTTTAATCCGCTGATTGCTCTGCTGCGCCCGGTCAGCCCGCTAGCCTGGCTGCCGATTGGCCTGCTGCTGTTCCAGAAGGCGGAGCCGGCTTCCAGCTGGACCATTTTTATCTGCTCCATCTGGCCGATGGTGATCAACACCGCGGAGGGTGTGCGGCGGATCCCGCAGGACTATCTCAACGTCGCCCGCGTCCTCCAGCTCTCGGAGTGGACGGTGATGCGCAAAATCCTCTTTCCGGCGGTGCTGCCGGCGGTGCTTACCGGCGTGCGCCTCTCCATCGGCATCGCCTGGCTGGTGATCGTGGCGGCGGAAATGCTTACCGGCGGGCTGGGGATCGGCTTCTGGATCTGGAACGAGTGGAACAACCTCAATGTGGAAAACATCATCATCGCCATCGTCATTATCGGCGTCGTCGGCCTGCTGCTGGAGCAGGGCCTGATGCTGCTCGCTCGCCGCTTTAGCTGGCAGGAAAAATAA

>IR1230_00163

ATGGGCGACAAATTTTCAATTTCCCGGCGGCGCTTGCTGCAGGCAGGGGCCGCGCTGGGCGGCGCGATGCTGCTGCCGGGCGTGATGCAGGCCGCCTGGGCCGGCGGCTCGGACAAACCGGAGCAAACCCGCGTGCGGGTCGGATTTATTCCGCTGACCGATTGCGCGCCGCTGGCCATCGCCGCGGCGAAGGGGTTTGACCAAAAATATGGCATCACCCTGGTGGCGAGTAAGGAAGCCAGCTGGGCGGCGGTGCGCGACAAGCTGGTGGCCGGCGAGCTGGACGCGGCGCATATTCTCTATGGCCTGCTGTACGGCCTCGAACTGGGGATCGCCAGCAAGCCGCAGGCGATGGCCAATCTGATGACCCTCAACCGCAATGGCCAGGCGATTACCCTCTCCAGCGAGCTGCAGGAGAAAGGGGTGACCGACCTCGGCGGGCTGAAGCGGCTGATCGACCGCAGCGCGCCGGGCAGTTACACCTTCGCCCATACCTTTCCTACCGGCACCCATGCCATGTGGCTTTACTACTGGCTGGCGAGCGCCGGCATCGATCCCTTCAACGATGTGCGCACCGTCGTGGTGCCGCCGCCGCAGATGGTGATGAACATGCGCATCGGCAATATGAGCGGCTTTTGTGTCGGCGAGCCGTGGAACGCCCGCGCTATTAACGACCGTATCGGCTTCACCGCGGCCACTTCCCAGGATATCTGGCCCGAGCATCCGGAAAAGGTGCTGGGCACCCGTCGCGACTGGGTGGAACGCAACCCGAACACCGCCCGCGCGCTGGTGGCGGCCCTGATGGAGGCGCAGCGCTGGATCGCCGCCTCGCCGGAGAACACCCGGGAGACGGCCCGCCTGCTCGCCAGACGCGGCTGGCTCAATACCAAGGAGCAATACCTCACCGGCCGGATGCTTGGAGAGTATGACAACGGCCTTGGCCGCCGCTGGCAGGATGCCCATCCGATGCGCTTCTGGGCCGGGGGCGAGGTGAGTTTCCCGTGGCTGTCGGACGGGATGTGGTTCCTCACCCAGTTCCGCCGCTGGGGGTTGCTGAAGCAGGCTCCGGATTACCTCGCGGTGGCGTCGCGCATTAACCGCATCGATGTCTGGCAGGCTGCCGCCCAGGCGGTAGGAGGGATCAGCGCGCCGGCAGCCAGGATGCGCAGCAGCACACTAATGGACGGGACGGTCTGGAACGGGTCCGACCCGGAAGGCTACGCCCGCCATTTCTCTATTCAACGTAAGGGGGCATGA

>IR1230_00164

ATGAATAATACGACTGGTCATGCGCACGATGCGACGGCCTGGCTGCAGCTGGCGCGTCGCCTGCAAAAACAACAGCTTCAGCAGCTTAGCCAACTGGGGGAACTGGCCAGCCAGCTCAGCGCGCTGGTGCATATGCTGCAGTGTGAGCGCGGGGCGTCGAATATCTATCTTTGCTCGGGCGGCTTGCTGTATACAGCGGAGTGTCGGGCGGGAGGGGCGCTGGTTGACGAGCGGCTAGCCCTGTTTTATGCCAGCCTCGAACGGGCGCGCGCGGTAGCCGGCAGCGCGCTGTGCTGGCGTATCGCCAGAGCGCTTGACGAGCTGGCGCAGCTCCCGGCACTACGCGCGCAGATCGGCCGCCGGCAAATTGCCGCTGAAGCCGCGACGGAGCAGTTCAGCCGCGTCATCCGCCATCTGCTGAACATCGCCCCGCAGCTCAATGACAGCATTGACGATCCGCCGGTGGCTGGTCGGATGGTCGCCCTGTACAGCTTTATGCAGGGAAAAGAGCTGGTGGGCCAGGAGCGCGCGCTGGGAGCATTGGGCTTTACCCGCGGCGAGTTCAGCGACAGCCTGCGCCAGCAGCTGGTGGACCGTATTGACGGTCAGCAGCCCTGCTTCGACAGCTTTCAGGCGCTGGGCAGCCCGGCGACCGTCCAGTTGTTCAGAACCCAGTGCCACGCCGGTCTGGATATTGAACAGCTGCGGCGGATCGCCTGTACCCGCCAGCCGGCTGCCGACGGCGGGGAAACGGCGCTGCGCTGGTTTGGTCTGCAAACCCAACGGCTTGAGCAACTGCGCGAAGTGGAAGAGCAACTGATCGACGATCTGCTGGATGCCACCGACGCGCTGTTAGCAGACGACGCGCCTGGCTGGCAGGCCGGAGAGGAAGACGATAGCGTGACGCCACGGCTCGATAAACAGCTACTGCCGCTGGTGCGCCAGCAGGCCTATGAGCTGCAGCAGCTCTCCAGCCAGCTGGCCTCGCTGAAAGACGCGCTCGAAGAGCGCAAGCTGATCGAAAAAGCGAAAAGTCTGCTGATGACGCACCAGGGAATGCAGGAGGAGCAGGCCTGGCAGACGCTGCGCAAGATGGCCATGGACAAAAATCAGCGCATGGTTGAGATAGCCCGCGCGCTGCTGATGGTGAAGGCGATTTGGCCGCTAACACCAAAGGAGTAG

>IR1230_00165

ATGCAACGTATTGTGATCATCGCCAACGGCGCCGCCTACGGCAGCGAATCCCTGTTCAACAGCCTGCGCCTGGCCATTGCCCTGCGCGAGCAGCAGTCCGACCTCGATCTCAAGCTGTTTCTGATGTCCGACGCCGTCACCGCCGGGCTGCGGGGGCAGAAGCCGGCAGAAGGGTATAATGTGCAGCAGATGCTGGAGATCCTCACCGCCCAGCAGGTTCCCGTGAAGCTGTGTAAAACCTGCGCGGACGGCCGCGGGGTCAGCGCACTGCCGCTGGTTGACGGCGTGGAGGTTGGCACTCTCGTTGAGCTGGCCCAGTGGACCCTGGCGGCAGATAAAGTGCTGACCTTCTGA

>IR1230_00166

ATGGCGATGGTTAAAGCAAGTTTGACGTTATTTGGTGGTGACACGCTGGTGGTGCGGTGCTCAGAGCGCTGCCATATTCATCTGATGAGCGCGAAGGTCCCGGGCGACAGTCATGCGGATATTCTGAGCGTGCAGGATCGGGACAGCGCGTATCTGACGGTGCCCTACAGCGGCACCTGGAACGTGCTTATCGACAGTCACAGCCAGTCGCTGGAGCACTCCATCAGCTACGTGCCGGCCTGA

>IR1230_00167

GTGTTAACTCGTGATTTCTTAATGAATGCGGATTGTAAGACAGCGTTTGGCGCTATTGAGGAATCGCTACTCTGGTCGGCAGAACAACGTGCAGCCTCGCTGGCGGCGACGCTGGCCTGCCGCCCGGATGATGGCTCAGTATGGATCTTTGGCTACGGTTCGCTTATCTGGAACCCGGCGCTCAATTATCGCGAGTCCTGCACCGGCACGCTGCCGGGCTGGCACCGCGCGTTTTGCCTGCGGTTGACCGCCGGGCGCGGTAGTGCCTGCCAGCCGGGACGCATGCTGGCTCTCAAAGAGGGCGGCCGAACCACCGGCGTCGCCTATCGTCTGCCTGACGACACGCTGGAGGAGGAGCTGACCCTGCTGTGGAAGCGGGAGATGATCACCGGCTGCTATCTGCCCACCTGGTGCAAGCTGGAGCTGGACGACGGACGGACGGTGAATGCCCTGGTGTTTATTATGGATCCGCGCCATCCACTGTTTGAACCGGATACCAGTGCCCAGGTTATCGCCCCGCTGATCGCCCGGGCCAGCGGGCCGCTCGGCACCAACGCCCAGTATCTGTTTTCGCTTGAGCAGGCGCTGCGCAAGCTGGGCATGCACGATGCCAGTCTCGATGACCTGGTGGCCAGCGTCCGCGCGCTGTTGGGCGAGAGTCCGACGCCGGGCCTGGCCTGA

>IR1230_00168

ATGCCCTATCGCAGTAAACAAGAACTCCCCGACAGCGTTCAGCATGTACTGCCTGCCCATGCGCAGGAAATTTATAAAGAAGCGTTTAACAGCGCCTGGGATCAATATAAAGATAAAGACGAGCGGCGCGATGACGCCAGCCGCGAAGAGACGGCGCACAAAGTCGCCTGGGCGGCGGTGAAGAACAGCTATGAGAAAGGCGATGATGATAAGTGGCATAAGAAAAAATAA

>IR1230_00169

ATGACCCATGCACATGAGGCGGTAAAAACCCGCCATAAGGAGTCTTCCCTCGTCTTTCCGGTGCTGGCGCTGGCGGTGCTGTTTTTCTGGGGCAGCAGCCAGTCACTGCCAGTGGTTATCGCGATCAATATCCTCGCGCTGGTCGGCATTTTGAGCAGCGCCTTCAGCGTGGTCCGCCACGCCGACGTCCTCGCCCACCGCCTGGGCGAACCCTATGGCTCCCTGATTTTAAGCCTGTCGGTGGTTATCCTTGAGGTCAGTTTGATCTCTGCCTTAATGGCCACCGGCGACGCCGCGCCGACCTTGATGCGGGATACCCTCTACTCCATTATTATGATCGTCACCGGCGGTCTGGTCGGTTTCTCTCTGCTGCTGGGCGGGCGTAAATTCGCCACCCAATATATGAATCTGTTCGGCATTAAGCAGTATCTTATCGCCCTGTTTCCGCTAGCGATTATCGTGCTGGTCTTTCCGATGGCGCTGCCGGGCGCGAACTTCAGCACCGGGCAATCGCTGCTGGTGGCGGTAATTTCGGCGGCCATGTACGGCGTTTTCCTGCTTATTCAGACCAAAACCCATCAGAGCCTGTTTGTTTATGAGCATGAAGACGACAGCGACGATGACGACCCGCACCACGGTAAACCGTCCGCCCACAGCAGCGGGTGGCATACCGCCTGGCTGCTGGTGCACCTGGTGGCAGTGATCGCGGTTACCAAAATGAACGCTAACCCGCTGGAAACGCTGTTGACCAGCATGAATGCCCCGGTGGCCTTTACCGGCTTCCTGGTGGCTCTGCTGATCCTGTCGCCGGAAGGCCTTGGCGCCCTGAAAGCGGTGCTCAACAATCAGGTACAGCGGGCGATGAATCTGTTTTTCGGCTCTGTGCTGGCCACCATCTCGCTGACGGTCCCGGTGGTCACCCTCATCGCCTTCCTGACCGGCAATGAGCTGCGCTTTGGCCTTGGCGCCCCGGAGATGGTGGTGATGGTCGCCTCGCTGGTGCTGTGCCATATCTCCTTCTCCACCGGACGCACCAATGTGCTGAACGGCGCGGCCCACATGGCGCTGTTCGCCGCCTATCTGATGACCATTTTCGCCTGA

>IR1230_00170

ATGAAACAAAAAGTGGTTAGCATTGGTGATATCAACGTAGCGAACGACCTGCCGTTCGTGCTGTTCGGCGGGATGAACGTGCTGGAGTCGCGCGACCTGGCGATGCGTATCTGCGAACATTATGTCACCGTTACGCAGAAGCTTGGTATTCCCTACGTATTCAAAGCCTCCTTTGATAAAGCCAACCGTTCCTCCATCCACTCCTATCGTGGGCCGGGTCTGGAAGAAGGGATGAAGATTTTCCAGGAGCTGAAGCAGACCTTTGGCGTGAAAATCATCACCGACGTTCATGAAGCCAGCCAGGCGCAGCCGGTAGCTGACGTGGTGGACGTGATCCAGCTTCCGGCTTTCCTCGCGCGCCAGACTGACCTGGTGGAAGCGATGGCGAAGACCGGGGCGGTGATCAACGTCAAGAAGCCGCAGTTCGTCAGCCCGGGTCAGATGGGCAACATCGTCGATAAGTTTATCGAAGGGGGTAACGACAAAGTAATCCTCTGCGACCGCGGCGCGAACTTCGGTTACGACAACCTGGTCGTGGATATGCTTGGCTTCGGCGTGATGAAGAAAGCCTCCAACAACTCGCCGGTCATTTTCGACGTCACCCACGCGCTGCAGTGCCGCGACCCGTTTGGCGCAGCCTCCGGCGGTCGTCGTGCGCAGGTGAGCGAGCTGGCCCGCGCCGGTATGGCGGTAGGTATCGCCGGTCTGTTTATTGAAGCGCATCCGGATCCGGACCACGCGAAATGCGACGGCCCGTCCGCGCTGCCGCTGGATAAACTGGAGCCGTTCCTCAAACAGATGAAAGCGATTGACGATCTGGTGAAAAGCTTCGACGAGCTGGATACCAGCAAGTAA

>IR1230_00171

ATGGGGTCATTAGCGGATTTCGAATTTAACAAAGCGCCATTGTGTGATGGCATGGTCCTCATTTCTGAACAGGTCCGCGACGATTTTCCGTCGCGTTTTGTCGAGGAAGAGCTGCAGCGATTGCTGCGCCTCGCGCAGGAGGAGATTGCGCCGTCGTGGGACCAGGAGCGCCAGATAGAACGATTGCTGGAGCTGTTTTATGACGAGTGGGGCTTTGGCGCCTCGCAGGGCGTGTATCGTCTTTCCGACGCGCTATGGCTGGATAAAGTGCTGGTTAACCGCCAGGGGAGCGCGGTGTCGTTAGGCGCGATCCTGCTGTGGATCGCCCAGCGCCTGGCGCTGCCGGTGGTGCCGGTGATCTTCCCGACCCAGATGCTGCTGCGTGCGGACCCGGAAACCAGCGAAGAGATGTGGCTGATTAATCCGTTTAACGGCGAGACCCTCGACGAGCATACCCTGGAGGTATGGCTGAAGGGCAACATCGGGCCGGTGGCGGAACTGTTTAATGAAGATCTCGACGAAGCGGATAACGCCGAGGTGATCCGCAAGCTGCTGGACACCCTGAAGTCGGCGCTGATGGAAGAGCGGCAGATGGAGCTGGCCCTGCGCGCCAGCGAAGCGCTGCTGCAGTTTAATCCGGAAGATCCGTATGAGATCCGCGACCGCGGGCTGATATACGCCCAGCTGGACTGCGATCACGTGGCGCTGTTGGATCTGAGCTATTTCGTTGAGCAGTGCCCGGAGGATCCGATCAGCGAGATGATCCGCGCGCAGATCAACACGATTTCGCATAAACAAATTACATTGCATTAG

>IR1230_00172

ATGAACTTATTCACGGCTGTCCTCTACCTGCACATTGCCACAGTGGCGGTGTCGGTAGGATTGTTCGTTTTACGTTACTGGTGGATGTATCACCAGAGCCCGCTGCTGAACCAGCGGTGGGTGCGAATTGCGCCGCACTGCAGCGATACGCTGCTGTTTCTCAGCGGCGCAGGGTTAATGGCGATAACGCACTACCTGCCGTTCACAGAAGACGGCGCATGGCTGACTGAAAAGCTGTTTGGCGTTATCATTTACATCGCATTAGGTTTTATCGCGTTGGGCCGTCGTCGTCCGCGCAGCCAGCAGAGCCGCTTTATCGCGTTCCTGCTGGCGCTGGTGGTGTTGTTTATCATCATTCAACTCGCCATCACAAGAATACCGATACTGGGGTAG

>IR1230_00173

ATGACCTTTCAGGCCTGGCTGCAGCAGGCGATCGCCCGCCTGGCGGAGAGCGACAGCCCGAGACGCGACGCCGAGATCTTGCTGGGACACGTTACCGGCCGGGCGCGAACCTGGATTTTGGCCTTCGGTGAAACGACGCTCTCTGCTGACGAGGCGGCGAGGCTGGAGGCGCTCCTGGTGCGACGCCAGCGCGGGGAACCTATTGCTCATCTGGTCGGGCAGCGCGAGTTTTGGTCGCTGCCGCTGTTCGTCTCCCCGGCGACGCTGATCCCCCGCCCGGATACCGAATGTCTGGTAGAGCAGGCGCTGGCGCGTCTGCCAACCGCGCCGTGCCGTATTCTCGACCTCGGCACCGGGACCGGCGCCATTGCCCTGGCGCTGGCCAGCGAACGTCCGGACTGTGAGGTCACCGCGGTGGACGTGATGCCGGACGCCGTGGCGCTGGCGTTGCGCAATGCCGAACATCTGGGCATCGCTAACGTGACGATAAGTCAAAGCGACTGGTTTAGCGCGCTGGCCGGGCAGCGCTTCGCCACGATCGTCAGCAATCCCCCCTATATCGACGCCGCCGACCCGCATCTCGCCGAAGGCGATGTGCGCTTTGAGCCGTTGACGGCGCTGGTCGCAGGCGACCAGGGGCTGGCCGATCTGGCGCATATCATCCGCGAGGGACGGCAGTATCTGCAGCCCGGCGGTTGGATGCTGCTGGAGCATGGCTGGACTCAGGGCGAGGCGGTTCGCGCGCTGTTCCGCGAAGCGGGCTATCTGGACGTCGCGACCTGTCGCGACTATGGCGACAATGAGCGTCTGACCCTCGGACGTTTACCCGACATGGAGAATGTTGGCTGA

>IR1230_00174

ATGAAGTCTTCTATTGTTGCCAAACTGGAAGCGTTGTACGAGCGCCATGAAGAAGTGCAGGCGCTGCTCGGCGATGCCGCGACGATTGCCGATCAGGATAAATTCCGCGCGCTGTCGCGGGAGTATGCTCAACTGAGCGATGTGGCGCGCTGCTATACCGACTGGCGTCAGGTGCAGGAGGATATTGAAACCGCGCAGATGATGCTCGACGATCCGGAAATGCGCGAAATGGCGCAGGAAGAGCTGCGTGACGCCAAAGAAAAAGGCGACCAGCTGGAGCAACAGCTGCAGGTTCTCCTCCTGCCGAAAGATCCTGATGACGAGCGTAACGCCTTCGTGGAAGTGCGCGCCGGGACCGGCGGCGACGAAGCAGCGCTGTTTGCCGGCGATCTGTTCCGCATGTACACCCGTTACGCAGAATCGCGCCGCTGGCAGGTAGAGATCCTCAGCGCCAACGAAGGTGAGCACGGCGGCTTTAAAGAGGTGATCGCCAAGATCAGCGGCGACGGCGTTTACGGTCGGCTGAAATTTGAATCCGGCGGCCATCGCGTGCAGCGTGTGCCGGCCACCGAATCGCAGGGGCGGATCCACACCTCCGCCTGTACGGTGGCGGTGATGCCGGAGCTGCCGGAAGCCGAAATGCCGGACATTAACCCGGCGGATCTGCGCATCGATACCTTCCGTTCCTCCGGGGCAGGCGGTCAGCACGTTAACACCACCGACTCGGCTATCCGCATTACCCACCTGCCCACCGGCATCGTGGTGGAGTGCCAGGACGAGCGTTCTCAGCATAAAAACAAAGCCAAGGCGCTGTCGGTGCTGGGCGCGCGCATCCGCGCCGCCGAAGTGGCCAAACGTCAGCAGGCGGAAGCCTCCACGCGCCGCAACCTGCTGGGCAGCGGCGACCGCAGCGACCGCAATCGCACCTATAACTTCCCGCAGGGCCGCGTGACCGACCACCGCATCAACCTGACGCTCTACCGTCTGGATGAGGCGATGGAGGGCAAACTCGACATGCTGATTGAGCCGATTGTCCAGGAACATCAGGCCGATCAGCTGGCGGCGCTGTCCGAGCAGGAATGA

>IR1230_00175

ATGACCCTTTTAGCTCTTGGCATCAATCACAAAACAGCTCCGGTCGCCCTGCGAGAACGCGTCACGTTTTCGCCGGAAACGCTCGATAAGGCGCTGGAGAGCTTGCTGGCTCAGCCGATGGTGCAGGGTGGGGTGGTGCTGTCGACCTGCAACCGCACGGAACTCTATCTCAGCGTCGAAGAGCAGGATAACCTGCAGGAAGCGCTGATCCGCTGGCTGTGCAACTACCACGGCCTCAACGAAGAAGACCTGCGAAAAAGCCTTTACTGGCACCAGGATAATGACGCCGTCAGCCATCTGATGCGCGTCGCCAGCGGCCTCGATTCGCTGGTGCTGGGCGAGCCGCAGATCCTCGGCCAGGTGAAAAAAGCCTTCGCCGACTCCAGCCGCGGCCATCTTAACGTCAGCGAGCTGGAGCGGATGTTCCAGAAATCCTTTTCAGTGGCTAAGCGCGTGCGTACCGAAACCGATATCGGCGCCAGCGCGGTCTCTGTGGCTTTCGCCGCCTGTACCCTGGCGCGGCAAATCTTTGAATCGCTCTCCAGCGTCACCGTGTTACTGGTCGGCGCCGGGGAAACCATTGAGCTGGTAGCGCGCCATCTTCGCGAACATCACGTGCGCAAAATGGTGATCGCCAACCGCACCCGTGAACGCGCCCAGGCGCTGGCGGAGGAAGTGGGCGCCGAGGTGATAGCCCTCAGCGATATCGACGAACGGCTGAAAGAGGCCGACATCATTATCAGCTCCACCGCCAGCCCGCTGCCGATCATCGGCAAAGGCATGGTGGAGCGTGCGCTTAAGGCGCGGCGTAACCAGCCGATGCTGCTGGTGGATATCGCCGTCCCGCGCGACGTTGAACCAGAGGTCGGCAAACTGGCTAACGCCTACCTCTACAGCGTCGACGATTTGCAAAACATTATTCAGCATAACCTGGCGCAGCGTAAGGCTGCCGCGGTGCAGGCGGAATCGATCGTCGAGCAGGAAACCAGCGAATTTATGGCCTGGCTGCGCGCGCAGAGCGCCAGCGAGACCATTCGCGAATACCGTTCTCAGTCTGAGCAGGTCCGTGAGGAGCTGACGGCGAAAGCGCTGGCCGCGCTGGAGCAGGGCGGTGATGCCCAGGAAATTATGCAGGATCTGGCGCGCAAGCTCACTAACCGCCTGATCCACGCGCCAACCAAATCTCTTCAGCAGGCCGCCCGTGACGGGGACGACGAACGTCTGCATATTCTGCGCAACAGCCTCGGGCTGGAATAG

>IR1230_00176

ATGAATCGACTGTTCCGCCTGCTGCCGCTGGCCAGCCTCGTCCTCACCGCCTGTTCTCTTCATACGCCGCAAGGGCCTGGCAAAAGCCCGGATTCGCCGCAGTGGCGTCAGCACCAGCAGGCTGTCCGCAGCCTCAATCAGTTCCAGACCCGCGGGGCGTTTGCCTATCTTTCCGATGAGCAAAAAGTCTATGCCCGCTTCTTCTGGCAGCAAACCGGCCAGGACCGCTATCGCCTGCTGCTGACCAACCCGCTGGGCAGCACCGAGCTGTCGCTGACCGCGCAGCCGGGCAGCGTGCAGCTGATCGATAACAAAGGCCAGACCTATACCGCCACTGATGCCGAAGAGATGATTGGCCGTCTGACCGGGATGCCGATCCCGTTGAACAGCCTGCGCCAGTGGATTATCGGCCTGCCGGGCGACGCCACCGACTATTCGCTGGACGATCGGTACCGTCTGCGTGAACTGAACTATACGCAAAACGGCAAAACCTGGCACGTGACCTACGGCGGCTACACCAGCGATACCCAGCCAGCCCTACCGTCCAATGTCGAGCTGAACAATGGCGCTCAGCGCATCAAGCTGAAGATGGATAACTGGATCGTGAAATGA

>IR1230_00177

ATGATGACCCGCTGGCCCTCTCCTGCAAAACTGAATCTGTTTTTATATATCACCGGCCAGCGGGCCGATGGTTACCATACCCTGCAAACGCTGTTTCAGTTTCTCGATTACGGCGATACGTTGACCATCGAACCGCGTACTGACGGCCAACTGCGCCTGCTGACGCCGGTGGCCGGCGTGCCCGACGAGGAGAATCTGATCGTTCGCGCCGCGCGTCTGCTGATGCATGCCGCCAGCGAAAGCGATCGCCTGCCGGCGGGCAGCGGCGCCGATATCAGTATCGATAAGCGCCTGCCGATGGGCGGCGGCCTGGGCGGTGGCTCCTCCAATGCCGCCACCGTGCTGGTGGCGCTCAACCATCTGTGGGGCTGCGGTCTCTCGGAAGATGAGCTGGCGACTCTTGGCCTTCAGCTGGGGGCCGATGTGCCGGTTTTCGTGCGCGGCCATGCCGCCTTCGCCGAGGGGGTGGGTGAAATACTGACGCCGGTAGAACCCGAGGAAAAATGGTACCTGGTGGCCCACCCGGGGGTCAGTATTCCGACGCCGATCATTTTTCGCGATCCGGAGCTACCACGAAATACCCCGCGCCGGTCAATTAATACGTTATTAAATTGTGAATTCAGCAACGATTGCGAGCTTATCGCAAGAAAACGTTTTCGCGAGGTTGATGCCGCGCTTTCCTGGCTGTTAGAATATGCGCCGTCACGCCTGACCGGCACAGGGGCCTGTGTGTTTGCTGAATTTAACACCGAATCCGCTGCCCGTCAGGTGCTGGACACTGCCCCGGCATGGCTAAATGGCTTTGTGGCGCGCGGAGTTAACCTTTCGCCGCTCAAGCAGGCCCTGCTCTGA

>IR1230_00178

GTGCCTGATATGAAGCTTTTTGCTGGTAACGCCACCCCGGAACTAGCACAACGTATTGCCAACCGCCTGTACACTTCTCTTGGCGACGCCGCTGTAGGTCGTTTTAGCGACGGCGAAGTCAGCGTACAAATCAATGAAAACGTACGCGGTGGTGATATTTTCATCATCCAGTCCACTTGTGCTCCCACCAATGACAACCTGATGGAACTGGTTGTTATGGTTGATGCTCTGCGTCGTGCTTCGGCAGGTCGTATCACCGCCGTTATCCCTTACTTCGGTTATGCTCGTCAGGACCGTCGTGTTCGTTCTGCTCGTGTGCCAATCACCGCGAAAGTGGTGGCGGATTTTCTGTCCAGCGTCGGCGTTGACCGCGTGCTGACCGTTGACCTGCACGCAGAACAGATCCAGGGCTTCTTCGATGTGCCAGTCGATAACGTGTTCGGCAGCCCAATCCTGCTGGAAGATATGCTGCAGCTGAACCTGGACAACCCGATTGTGGTTTCTCCGGACATCGGCGGCGTCGTGCGTGCCCGCGCTATCGCTAAACTGCTGAATGATACCGATATGGCTATCATCGACAAGCGCCGTCCGCGCGCCAACGTCTCCCAGGTGATGCACATCATCGGCGACGTTGCCGGTCGTGACTGCGTGATGGTCGACGATATGATCGACACCGGCGGCACCCTGTGTAAAGCGGCAGAAGCGCTGAAAGAGCGTGGCGCGAAACGCTTATTCGCTTACGCGACGCACCCAATCTTCTCCGGCAACGCTATCCAGAACATCAAGAACTCTGTCATTGATGAATTTGTCGTCTGCGATACCATTCCGCTGGCACCAGAAATTAAAGCGCTGGATAAAGTGCGTACTCTGACGCTCTCCGGTATGCTGGCTGAAGCTATTCGCCGTATCAGCAATGAAGAATCTATCTCTGCTATGTTCGAACATTAA

>IR1230_00179

ATGCCTTTCCGCGCCCTCATCGACGCTTGTTGGAAAGAAAAGTACACCACCGCCCGCTTCACGCGTGACCTGATCGCCGGGATCACCGTCGGGATCATTGCGATTCCGCTGGCGATGGCGCTGGCGATTGGCAGCGGCGTGCCGCCGCAGTACGGCCTCTATACCTCAGCGGTGGCAGGGATTGTGATCGCCTTGACCGGCGGGTCACGCTTTAGCGTTTCCGGTCCGACCGCCGCCTTTGTGGTGATCCTCTATCCGGTTTCCCAGCAGTTTGGTCTGGCGGGACTGCTGGTGGCAACCCTGATGTCGGGGATCTTTTTAATCCTCTTCGGCCTGGCGCGCTTTGGCCGCCTGATCGAATACATCCCGCTGTCAGTCACCCTCGGCTTCACCTCGGGGATCGGTATCACCATCGGCACCATGCAGATTAAAGATTTCCTCGGCCTGCAGATGCCCCATGTGCCGGAGCATTATCTGCAGAAAGTGGCGGCGCTGGCGATGGCCCTACCGACCATTAACGTTGGCGATGCCGCCATCGGCGTCGTTACTCTTGGCATTCTGATCCTCTGGCCGCGGCTGGGTATCCGCCTGCCGGGCCACCTCCCGGCGCTGCTGGGCGGCTGCGCGGTCATGCTGGTGGTCAACTTGCTCGGCGGCGATGTCGCGACGATCGGCTCGCAGTTCCATTATCAGCTGGCGGACGGCACTCAGGGCAATGGCATTCCGCAGCTGCTGCCGCAGCTGGTATTGCCCTGGGATATGCCGGGCTCAAACTTTACCCTGAGCTGGGCCTCGCTGCAGGCTCTGCTGCCTGCCGCCTTCTCAATGGCGATGCTCGGCGCCATTGAATCTCTGCTGTGCGCGGTGGTGCTTGATGGCATGACCGGCACCAAGCACAAAGCCAATAGCGAGCTGATTGGCCAGGGGCTGGGCAACATTGTCGCGCCGTTCTTCGGCGGGATCACTGCCACCGCGGCGATTGCCCGCTCGGCGGCGAACGTCCGCGCCGGCGCCACATCGCCGGTTGCCGCCGTGATCCACGCCCTGCTGGTGATCCTGGCGCTGCTGATCCTCGCCCCACTGCTCTCCTGGCTGCCGCTGTCGGCGATGGCCGCCCTGCTGCTGATGGTGGCATGGAATATGAGCGAGGCGCATAAAGTCATCAATCTGCTGCGCCACGCGCCGAAGGACGACATCGTGGTGATGCTGATGTGCATGTCGCTAACGGTGCTGTTTGACATGGTGATCGCTATCAGCGTCGGGATCGTGCTGGCTTCGCTGTTGTTTATGCGCCGTATCGCACGCATGACTCATCTGGCGCCGGTCAACGTCGAGGTGCCAGACGATGTGCTGGTGCTGCGGGTAATTGGGCCGCTGTTCTTTGCCGCCGCCGAAGGCTTGTTCAACGATCTGGAGACCCGGATCGCCGGCAAGCGCATCGTGGTGCTGAAATGGGACGCGGTGCCGGTTCTCGACGCCGGTGGCCTGGATGCCTTCCAGCGCTTTGTGAACAAGCTGCCGGAAGGCTGTGAATTGCGCGTATCTAACCTCGAGTTTCAGCCGCTACGCACGCTAGCGCGCGCCGGCGTCAAGCCGCTGCCCGGACGCCTGAGCTTCTACCCCGATCGTCAGGCCGCGCTGGCTGACCTGTGA

>IR1230_00180

ATGTTAATTCAGGTGGGAGAGCTGGCGAAGCGTGCCGGACTGACGGTACGTACGCTGCATCATTATGAACAGACGGGGCTGTTAACGCCTTCGGCCAGAAGCGAGGCGGGCTATCGGCTCTATAACCTGTCCGCGGTTCAGCGCCTGCATATGATAAAGGCGCTGGCGCAGGCCGGGCTAACGCTCGCCACAATCAAGGACTATCTCGATCGGCAAACGCTGTCGCTGCCCGAGCTGCTGACGCAGCAGATAGATATGCTCAACGCCCAGCTACGCGATGTTGGCAGGCTGCGCGACCGACTATTGGTGCTGCGCGAGGCGCTGGCGAGCGGCAATGAACCCGATCTGGAGTCCTGGCTACAGACGCTGGAGTTAATGAAAATGTACGATCGTTGGTTTAGTCAACAGGAGTTAGCCGCGCTGCCGTTTGCGGCACAGGATGAACAGCGGGCGCAGGCGTGGCGCGAGCTAACGGAGGAGGTGCAGACGCTGATGGCGAGCGGCTGCCCGACGGACAGTCCGCAGGCGATGCGTCTGGCGACGCGCTGGATGGAGCGCCTGGAGCAGGATACGGCCGGGCGTCCGGAGTTTCTGACCCGCCTGAACGAGATGCATGCCGCCGAGCCGCAGATGGTTGAACAGACCGGCGTCACGCCGGCAATCATCGCCTATATCACCGAGGCCTTTGCCGAAAGCAAACTGGCCATCTGGGCGCGGTACCTTGACGAAGAAGAGATGGCGTTCACCCGCCAGCACTATTTTGACCGGCTACAGGAGTGGCCTGCGCTGGTTGCGAAGCTGCATCAGGCGTGCCGGGAGGGCGTAGCGCCGGACTCGGCGTCGGGTCAGGCGCTGGCCCGGGCGTGGCTTGAACTGTTTCAGTCCTACGCCGGTACCCGGCCGCAGACGTTGCAGAAGTTTCGTCGGGCAATGGAGCAGGAGCCGCATTTGATGAAGGGCACCTGGATGACGCCGGCGGTGCTGAGCTGGCTGCAGCAGGCGACCGGATCCTTGATGCGGCAGGCGCAGGGACCTGCCGCCGGGTGA

>IR1230_00181

ATGAAACGCAAAAACGCTTCGTTACTCGGTAACGTACTCATGGGGTTAGGGTTGGTGGTGATGGTTGTGGGGGTAGGTTACTCCATTCTGAACCAGCTTCCGCAGCTTAACCTGCCACAATTCTTTGCGCATGGCGCAATCCTAAGCATCTTCGTTGGCGCAGTGCTCTGGCTGGCCGGTGCCCGTATTGGCGGCCACGAGCAGGTCAGCGACCGCTACTGGTGGGTGCGCCACTACGATAAACGCTGCCGTCGTAACCAGCATCGTCACAGCTAA

>IR1230_00182

GTGACCATTAAACTGATTGTCGGCCTGGCCAACCCCGGTGCGGAATATGCCGCTACCCGGCACAACGCTGGCGCCTGGTATGTGGATTTACTGGCGGATCGCCATCGTGCGCCGCTGCGCGAAGAGAGCAAATTCTTCGGCTACACCTCGCGCATCAACCTGGCGGGCGAGGACGTCCGCCTGCTGGTCCCCACTACCTTTATGAACCTCAGCGGTAAAGCCGTGGCCGCGATGGCGACCTTTTATCGCATCAACCCTGATGAAATTCTGGTCGCCCATGACGAGCTGGATTTACCGCCGGGCGTGGCGAAATTTAAGCTTGGCGGTGGCCATGGCGGCCACAATGGCCTGAAGGATATCATCAGTAAGCTCGGCAATAACCCGAACTTTCACCGTTTACGCGTCGGAATCGGCCATCCGGGCGACAAAAACAAGGTTGTCGGCTTTGTGCTTGGCAAACCACCGGCCAGCGAGCAGAAACTGATCAATGACGCTGTCGACGAAGCGGCGCGCTGTACCGAAATCTGGTTAAAGGACGGGCTGACCAAAGCGACCAACCGTTTGCACGCCTTTAAGGCGCAATAA

>IR1230_00183

ATGGGATTCAAATGCGGTATCGTCGGTTTGCCCAACGTCGGCAAATCCACCCTGTTCAATGCGCTCACCAAAGCGGGTATTGAAGCGGCCAACTTCCCCTTCTGTACCATTGAGCCGAACACCGGTGTCGTACCGATGCCCGATCCGCGTCTGGACAAGCTGGCTGAAATCGTCAAACCGCAGCGCATCCTGCCGACCACCATGGAGTTCGTGGATATCGCGGGCCTGGTGAAAGGCGCGTCCAAAGGTGAAGGTCTTGGCAACCAGTTTCTGACCAACATCCGTGAAACCGAAGCCATCGGCCATGTGGTTCGCTGTTTTGAAAACGATAATATTATTCACGTCGCGGGGAAAGTGAACCCGGCGGAAGATATCGACGTTATCAACACCGAACTGGCGCTGTCTGACCTTGATACCTGCGAACGCGCGATTCACCGCGTCTCCAAAAAAGCCAAAGGCGGCGATAAAGACGCGAAGGTTGAGCTGGCCGCGCTGGAAAAATGCCTGCCGCAGCTGGAAAACGCCGGTATGCTGCGCGCGCTGGATCTGACCAAAGAAGAGAAAGAGGCCATCCGCTATCTGAGCTTCCTGACCCTGAAGCCGACCATGTACATCGCGAACGTCAATGAAGACGGTTTCGAGAACAACCCGTACCTCGATCAGGTTCGCGCCATCGCCGAGCAGGAAGGGTCTGTGGTGGTGCCGGTGTGCGCCGCCGTTGAAGCGGACATCGCCGAGCTGGACGATGAAGAGCGTGACGAGTTCATGGCTGAGCTGGGCCTCGAAGAGCCGGGCCTGAACCGCGTGATCCGCGCCGGTTACGCTCTGCTAAACCTGCAGACCTACTTCACCGCGGGCGTGAAGGAAGTTCGCGCATGGACCATCCCGGTCGGGGCAACCGCGCCGCAGGCAGCCGGTAAGATCCACACCGACTTCGAAAAAGGTTTTATCCGCGCGCAGACCATCGCCTATGAAGACTTTATTACCTACAAAGGCGAACAGGGTGCAAAAGAAGCCGGTAAAATGCGTGCTGAAGGTAAAGACTACATCGTGAAAGATGGCGATGTGATGAACTTCCTGTTCAACGTCTAA

>IR1230_00184

ATGGCAACGGCAAGAAGCGGCTATACGCTGCAGATGATAAAGGCAGGGCAGCAGGGACATGTTGAAATGAGGTGGGGGCATCTCGCAGACGTTGATACGCGCGCGGCGGCGGCGGCTATCATGCAGCATATCGGGCGTTCTTCGTCCTCGCGGGGCCAGCAGGAGATCAGCCTGTCGCTGACCAATGCCGCCAGCGGCATCTCGGTGGAGCTTCATCACCCGGCCTCTGTGGAAAGCACCACCCCGGCTTTTATTGAGGCTGAGTTAAAGAAAATTGTGCAGATCGTTGATGGCTACGAGGCGGCAGAAGATACCCATATTGTGGAGTAG

>IR1230_00185

ATGAAGCACTGTAAAATCATCCTGTTAGTAGGCCTTCTGGCCAGTTCCGCTTCGGCATTAGCAGAGAAGATCGGCGTCTCAATGGCCTACTTCGATCAAAACTTTCTCACTATTATCCGCCAGTCCATTGAGAAAGAAGCTCAAGCCCGCCACGTCGACGTACAGTTTGAAGACGCCCGCGGCGACACCGGACGCCAGGCCGATCAGGTGCAGAGCTTCATTGCCTCCGGCGTGGACGCGATCATCGTCGACCCGGTGGACTCCGCCAGCACCCCGCAGCTGACGAAGATGGCGCAGCAGGCCAAAATGCCGCTGGTGTATGTGAACCGGACGCCGGGGGATAAAACGTTACCGCCCGGCGTGGTGTTTGTCGGTTCGGACGAGCGGGAGTCTGGCACATTGCAGATGGAGGCGCTGGCGAAGCTGGCGAACTACAAAGGCAACGTCGCCATCATGATCGGCAATCTCACCGACGCCGGCGCGCTGCAGCGCACCAAAGATGTGGAGCAGGTGGTGGCCAAGTATCCGGCAATGAAAGTGGTGCAGAAACAGCCGGCCAACTATTCCCGTAGCGAGGGCATGGATCTGATGCAGAACTGGACGGGCAACGGAGAAGCGATTGATATTGTGGCGGCGAACAACGATGAGATGGCGATTGGCGCGGCGATGGCGCTGGAGAAAAGCCAGAAGAAGCTGCTGATCGGCGGGATCGACGCCACCCCGGACGGGCTGAAAGCCCTGGCCAGCGATAAGATTCAGGTGACGGTTTTCCAGGACGCCGTCGGTCAGGGCAAAACGGCCTTAGCGGTAGCGCTGAAGCTGATTAAAGGAGAAAAGGTCGAGTCTCATGTCTGGATCCCGTTTGAGTTAGTGACCAAAGAAAACATGCAGACCTATGTGGAAAAGAGTCATTAA

>IR1230_00186

ATGATGACAACACACCACGACCGTCACTATAAAGCCGGTGGCGACCCGCGCACGCTGGCTGATTTTATGGCCCTGCGTGCGGAAATGAACAAGCTCAGCCACCCGGCGCGGCCGGATATTAACTGGCCGTATGCCGAGCAGCTTGCCCGCGGGCTGTTGGAGCATCACGGCGCGGACCTGCAGACTGTCGCCTGGTATACCCTGGCCCGCGCCCGGCTGGGCGGGGTGGCAGGGATCAATGAGGGGCTGACGCTGATGGAGTCGCTGCTGGTTCGTCAGGGGAAAAACCTGTGGCCACAGGCGCTCCCGGCCCGGACAGAGATCTTCCGCACCCTGAGCAAGCGGCTGCGGCAGGTGATCCGTACCCTGAATCTTACCCCAGAGGATGTTGAGTCGCTTGAGCAGGCTGAACGTTCGCTGCAAAGCTTCGACGCAGTGCTCCAGCGCCTTGAGATCGCCCCTGAAAACCAGCTGAGCGACCTGCGGGCGCTACTGCACAGCACGGCAACTCGCTTCGAGAGCCTCGACCCTGCGCCAGCGCTTCCCACCGCGCCGCCGGTGGCGGTGTCTGACGCGGAACTTCCCGGCACTCTGGTCAGCGAAGAGGATGCGGCGAAGGTTGAGCCAGTGCCAGACCTAAAACGTAGACCAAAAGCTGAGCCGCTGGCGCCGCCTTCACCTGCAAAGCGGCCCGCGCCGGTGGCGGCGTCTACTCCGGCAGCCGCGCCGCGCTGGAAGCCGTTTATCGCCGGGATGGTTACCATGCTGGCGGTAACGGGTATCGCCGTCGGCGGATGGCTGGCGCTGCGTCAGTCCGACTTGCCGCCCATTTCGGTCACCCAAAATGCCGGACCGATACCGGGCCTGCCGGCGACAACGCCCCCGGGGCCGGTTGATCTGCCACAGACGCAGCGCCAGCTTGGCGAGCTTGCCCGCCTGGCGCCGGACTGGGCGGTCAGTTACGGCGACCAGCTGGTCCACCAGGCCCTGATCCGCTGGCCGGATCAGGCGCAACCGCTGGCGCAGCAGTGGCGGCAGCAGCTGAGTGCTGGCGCATTGCCCGCGGAAAACCTTACCGGCTGGTCGGAGGGCATGCAGCAACTGCAGCGGCTCGCCGACCAGCTCAATGCCCTCGACGAGCAGAAGGGGAAGTACCTGACGGTGAGCGAACTGAAAACCGCAGTATTTGCCATCACGCAGTCGTTTAACCGCGCTGTTCCGCTGGAGGAGCAGCTGCGCCAGCTGGCCGCGCTTCCCGTTGATCAACCCTGGCCAGCCGCGCGGGGAAGCCTCGCGGAGCTGCATCTGCAACAGCTGATAGTGGAGTATGCCCTGCTGAAGCGAAAACAGCCGGCGTCGCCGACCGCTGCGCTCCCCGCAACCGGTGAGCCCTCGGTGAGTGAGGCGGTGAAATAA

>IR1230_00187

ATGCCTCGTCCCTCCCTGTATGACATCCTGTACGGCAATTTCGCCGGCGGGCTTGACCTGAATACTGTCAGCGAAACGGATCAGGTCATCCTGTCGGTGCTCGATAACATGCAGCGCATCCTCAACTGTCGGGCCGGAACGCTGGCGCATCTGCCGGACTACGGCCTGCCGGATATGACCGCTGTCCTCCAGGGGATGCCAGCGTCGGCGCATCAGCTGATGAGCACATTGTCGGCGGTGTTGTTGAAATACGAACCGCGCCTGCAGCGGATCGCGGTGGTGATGCTCGATCAGCATGCGCCCGGCGAACTGCGCTATGCCATTGATGCGGAGCTGAAGGATATTGGCCTGGTGCGTTACGGCACGGAGTTTATGCCCGAGGGCAGGGTGTTGATCCGCCACCTCAAACGCCAGCAGTACCTTGACGCCCGCTCCGCCCTCTAA

>IR1230_00188

ATGGCGATTACCGCAGTTAAACCCTCCGCCAGGTTGCTGGCATTCCTCATGGTTACCGTCTTAACCGGCTGCGGACTGACGCAGACCGTGAAAGACGGCGCCGTTTCCGTGACCCAGTCCATTTTTTACCCCCAGGTGAAAACCCTGCATCTGGATCTTCGCGCCCGGGAAGGCGTGAATAACAACGCCAAAGGCGCCTCCCTGGCGACGGTGGTGCGTATTTATCAGCTGAAGGACCGGCAAGCGTTCGACAATACTGACTACCCGTCGCTGTTTGCCGGCGATGGCCAGGCGCTGCAGGCGGACCGGGTGGCGGAAAAAGATGTTCGCCTGCGTCCGGGCGAATCGGTGACCGTCGATATGCCCATGGAAACCAGCGCGCAGTTTGTGGCGGTGGCGGCCATGTTTATCGATCCAGACCTGACGCAAAACAGCTGGCGGCTGGTACTCACCCGGGACGAACTTGATCCGGCGAGGCCGCGCATTATCGAAGCCAGCCAAAATCAACTGACGCTGCACCCGTTTAAGGAGAAGTGA

>IR1230_00189

ATGGCGCAGCTCCGGCCTCAGTTGCCGTACATGAATTTTTACCGCTTCTGCCAGTTGCTGGAGAAGAACCATCCGAAGGCGCCGATCATCGGCAGCGGCTGGCTAGTAGGTGATGAACCCATACGCTTTCGCCCGCATCCGGGGATGGGCTTTCCGGCTGGCGAAATCCGTGGAATGGACGATCCTGAACCGCCCCGTCCGCCGGCGGTTCGCGTGACTTTTATGGGACTCTACGGCGTGGAATCCCCGCTGCCGACCCACTATAGCGATGACATTGCGCAACGGCGGGAAGGCGTCGAGGCCACCGAGGATTTCCTCGATATCTTTAACCATCGGCTGATCGCCCAGTATTACCGTATCTGGCGCAAATATTCCTACCCGGCAACCTTTCGGGCGGGCGGCACGGACAACATCTCGCAGTATCTGCTGGGCCTGGCGGGGCTGGGCATACCGGGATGTGCCGCCGTTGCCGCAGCGCCGCTGTCGCGATTTCTGGCGCTGCTGCCGGTGATGATGCTGCCGGGGAGATCCGGGGAAGGCATGGAGGCGCTGGTGGCGCTCCTGGCCCCGGGGACCCGGGCGACGGTGCACCACCACGATCCCTGCCGGATCCCCTTGTCGCAGCCGTTAACGATGAGCGTCCGTCAGCCGGTTAGCCTGCAACATAGGCCAGTGATGGGAACCCACGCCACGGATGTGAACGGTCAGGTATTGCTGCAGCTGGCCACGGAAAAGCCTGATGAGGTTCGCGGCTGGCTGCCCGGCGGCGAACTGTTCAGCGACCTGATGGCGCTTCTGCACGTCTGGCTCGGATCTCATCTTGACGTGCGGCTGCAGCTTTGCGTCGCCCGCCATTTACTGCCGGATGCGCAACTTTGCTGCCAGCAGGAGCATGCCGTCCAGCTCGGCCGTACCGCTGTGCTGCGTCCGCTGGATGCGCAGAAACAGGCTGACGACAGAGTCACGATTTATCTGGGGCGCTATCAGCGCGTCCGGGAAAATATCCACCGCAGGGAGAGCGATGAAGATGGCGATTACCGCAGTTAA

>IR1230_00190

ATGGACGATTTAACCCTGCGATATTTTGATGCTGAAATGCGCTACCTGCGCGAGGCGGCAAAAGCGTTCGCGCAGGCTCACCCCGATCGGGCGGCGATGCTGGATCTCGATAAAGCCGGTACGCCGGACCCTTACGTTGAGCGCCTGTTTGAAGGCTTTGCCTTTTCCGTAGGTCGCTTGCGCGAAAAAATTGATGATGATCTGCCGGAGCTGACCGAGGGGCTGGTCAGCATGCTGTGGCCGCACTATTTGCGCACTATTCCATCGCTATCGATCGTGGCGCTGACCCCAACGCTGCCGGCGATGAAGATGGCGGAAACGGTGCCCGCCGGGTTTGAGATTAGCTCCCGCCCACTGGGGCCGAAAAATACCGTGTGCCGGTACCGAACCACCCGCGATCTGACGCTTAACCCGCTGGCGATAGAGGAGGCGGTGATGACGGCGGAGCCGGACGGACGTTCGGCCCTGCGACTACGACTCGCCTGTAGCGAGCTGGCTGACTGGTCGCAGACCGATCTCCGCCGCCTGGCGCTGTACCTGGGGGAAGACGCTGTTACCGGCAGCGCCCTCCATCTGTGGCTGACCCGACGCCAGGCCGCGCTGTACCTGCGTCTGCCGGGTCAGACAGAGCGGGTAAGCCTCGACGGCTATTTTTCCCCGGGGGGATTCAGCGAGGAGGATTGTTTATGGCCGAAGGGGGAGAGCGCTTTCAGCGGCTATCAGCTGCTGCTCGAGTATTTCACCTTTCGCGAAAAATTTATGTTCGTGCAGCTAAATGGCCTGGAAAACATCACCCTGCCGGCGGGGATATCGCATTTCACGCTTGAGGTGGTGTTCAGCGAGGTCTGGCAAAGTGATTTACCCGTCAGCGCAAGCAGCCTGCGCCTGCACTGTGTGCCGGTGATTAACCTGTTTACCCTTGAGGCCGATCCGTTGACCATCAGCGGGCTGGAGAGCGAATATCTCCTGCGTCCGAAGCGCCTGCAGGACGGGCATACCGAAATCTATTCTGTTGACAGCGTGACCGGCTCCGGGCGCACCGGGGAGGCACGCTATGTGCCTTTCACCCGCTTTCGTCACCAGGGGGGAATGATGCGTCGCCATGCGCCGGAGCGCTACTACCACACCCGCGTAAAACGGGGCGTAACCGGCATGCACGATACCTGGCTTATCCTCGGCGGACAGCAATGGGAGGCTGACCGGGAGCTGGCGCGGGAAACGGTCTCCTTGCGCATCACCGGCACTAACGGCCAGCTGCCGCGCCGGGCGCTACAGAGCACGCTGCTGGATCGCTGTGAGTCGATATCAGCCACGCCGCTTACCGTCCGCAATCTCTGTAAGCCCACGCTGCCGGCCTATCCCCCGGCGGAAGACCGCTACCACTGGCGGGTCATGAGCCATCTGGGCACCCGTTTTCTCAACATGATGAGCAGCGCCGAGGTGCTGCGCGGCACGCTGTCGCTCTACAACTGGCGGGAGGATGAGCTCAATAATCGCCGTCTGGACGCCATTCTGGCGGTCAGCCATCACCGAATTCAGCGTTTCGAGCAGGGTTTCCTGCTGCGCGGGCTGGATATTGAAGTGACGCTCGACGGTAGCGGATTTACCGGTGCGGGCGATGTCCACCTGTTTGGCGACATGCTCAACCGCTTTTTCGCACTGTATGCCGATATGAATCAGTTCAACCAGCTGACGCTCATTGTTCAACCTGATGGGAAATGTATCCGGTGGAAAGAGAATCACAGTCTGCGCCTGCCCGGCTGA

>IR1230_00191

ATGAATATACCGGGTAATGGCCTGGGCCGGTTTGGCCTGGCCGGGCTTACGTTGGCCGGGATGGCTGCAATATGGTGGGGGATCACCCGCTATGGCGGCATGCTGAATATCAATACGCGGGGCGAGCAGGTACTGGCGTTTGTCCTGGTCTGTCTGGCGTTGGTATTTGTTTTTTACCTTCCTGCAATGTCCCGCCGTGTGAAGGAGAGTTTATATCGCCGGCAGTCGCAGCAGGACAGCGTCTTACCCGGCGAAGAAGGGCGCGTGGCGCTGACACCTCCCCATCATATTACGGTGGGGGAAATTCGCCAGACTCTACGCTATCAGTATGGACGCGCCTGGTCCCGCAAAGTCCGTATTCTGCTGATAATTGGCTCAGTCAGAGACGTAGAACAACTGACCCCAAAGCTCACGCAGGAACTCTGGCAGGAAGATCAGGGTACATTATTGCTGTGGGGTGGAGACCCTGGGGCAGCGGTAGATAACGCCTGGTTCACAGCGCTGCGTAAACTGCGTTATCGCCCGGCTGATGGTATGGTGTGGGTAACATCGGGCTTTGACGGCGTTTTGACAACGATGAACAGAACGAAGCCCAGGCTCACGCCAGATGAAATGGACAGTGTGTCTCACTCTCTAAAGCTACGCTACGAAGCTCTTGGCTGGAAACTACCCCTATACGTCTGGTCGCTGTATGAGGGGGGCAACGAAAAAAATGGACGTATCACTCAGCCTGTAGGCTGCCTGTTACCTGCAGGTTGTACGCCGCAAATAGTTGCCGAACAGTTCACTGCCCTGGCGTCGTTACTGATAGAGCAGGGCATCCAGCAGATATGCGGGCAACCACAGCACAATTTTTTACTCGCGCTGGCCGATCAGTTGACCCGTAAACCGGAAACGGTGACGGAGCCGTTATCGGTATTGCTTAATCCTTACCGCCCGCAGCCGCTGGCCGGGGTGGTATTTAGTGAGGCGTCAGTAGAGGCTGGTCGCAGTGTCCGTCATCACTGGGGACGGGATAACCGCTGGGAAACGATACCAGACTCAGTGCTGTGGTTGCCAGCCGGTCTACGTCCGCGCAAACAGGGAGTCAACTGGATGCGTGGGATGAGCGTGGCCGCCGCCGCCCTGATGTTACTCTGGGCCGCCTCAATGACAGTCTCTTTTATCGCCAACCGCCACCTGGTGGCGATCGCACAGCAGCAGGTGCAGCAGGCCTCGGCGGGAAAACAGCCGCTGGCCGTACGCCTGCATGCGCTCTCCGCGTTACAGAAAACGCTTTCTCAACTGGAATACCGCTCGCAGCACGGAGCGCCGTGGTACCTCCGCGCTGGCCTCAGTCAGAACGATGACCTGCTGGCGGCGCTGTTCCCGCGCTACGGGGAGATGGCGCAACCGCTGCTGCGCGATGCGGCGGCGCATCATCTGGAAGAGCAGCTCACCGCCTTCGTGCAGCTGCCGCCGGACAGCCCACTGCGTGAGAAGATGACGAAGACCGCTTACGGACAACTGAAACAATATCTGATGTTGACCCGCCCGGAAAAAATGGACGCCGCGTGGTTCGCCACTACGCTAATGCAGGACTGGCCGCAGCGCTCAGGCATCGCCGACGCCGTCTGGCAGGGCAGCGGCCCTTCGCTGCTGGCATTTTATGCCGCCAGCCTGGCATCGCATCCGCAGTGGCGTCTGCCTGTCGATGACGGTCTGGTCAGCCAGGTGCGCACACGGTTAATTCGCCAATTGGGCCAGCGCAACAGTGAGTCCACGCTTTACCAGAAGATGCTCGCGCAGGTGGCGAATCAGTATGCCGATATGCGCCTGGCGGATATGACTGCCGACACCGATGCTTCGCGTCTGTTCAGCACTGATGAGGTGGTGCCGGGAATGTTTACCCGTCAGGCCTGGGAACAGGCCGTGCAGCCGGCCATTGAGAAAGTGGTCGCGGAACGCCGCGATGAAATGGACTGGGTGCTGAGCGATACGAAGCAGACTGCCGCGCAGTCGACGTCGCCGGAAGCTCTGCGAGCCAGGCTGGCGGAGCGTTATTTCGCTGATTTCAGCGGCGCCTGGCTGGATTTCCTCAACAGTCTGCGCTGGCAGCGCGCGGCGACCCTCTCCGATGCTATCGACCAGCTGACCCTGATGGCCGATGTGCGCCAGTCGCCGCTGGTGGCGCTGATGAACACCCTGAGCGTGCAGGGGCGAACCGGCCAGACCGGGGAAGCCATCGCCGATTCGCTGGTCAAATCGGCCAGGCAGCTGTTCAACCGTGACAACCCACCCGCCATCGATCAGCAGTCCGGCTCGCGCGGTCCTCTGGATGCCACCTTCGGGCCCGTACTGGCGCTGCTGGACAATCGCGACGGCGGGACACCCACCAGCCGTCTTAGCCTGCAGACCTTCCTGACCCGCGTCACCCAGGTGCGTCTGCGCCTGCAGCAGGTCACCAACGCCACCGATCCGCAGGCGATGACCCGGTTGCTTGCGCAGACGGTATTCCAGGGCAAGGCCGTGGATTTAACCGAAACGCGCGACTACGGCAGCCTGGTGGCGGCGGGGCTGGGCCAGGAGTGGAGTGGATTTGGGCAAACGCTGTTTGTTCGCCCGATGGAACAGGCCTGGCAGCAGGTGCTGACCCCGGCGGCGGAAAGTCTCAACGCCCAGTGGCGCAGCGCGGTGGTGGAGGACTGGAATAGCGCTTTTGGCGGTCGCTACCCCTTTAAAAATACCAGCAGTGAAGTCTCTCTGCCGCTGCTGGCAAAATACCTCGACAGCGAGACCGGGCGCATCGCCCGCTTTCTGCAGACCCGGCTGAACGGCGTGCTGCATAAAGAGGGGAGCCGCTGGATGGCGGACAGTATCAATGCTCAGGGGCTGACGTTTAACCCGGCCTTTCTGCAGGCGATGAATACCCTGAGCCATCTCTCCGACGTCGCGTTCGCCAACGGCGAAGCGGGATTGCATTTTGCGCTACGTCCCGGCACCGCGGACGGCGTGATGCAGACGGAGCTGGTCATCGACAATCAGAAACTTGTCTATATGAACCAGATGCCGGTCTGGCGGCGGTTCAGCTGGCCCGCTGACACCGAAGCGCCGGGCGCCAGTCTGAGCTGGATCAGTACCCGGGCGGGCACCCGCCAGTATGGCGATTTCCCGGGCGCCTGGGGGTGGATCCGTCTGCTGGATAAGGCGGTGGTCAGCGCTTATCCCGGAACGAGCAGCAGCTGGAGCCTGAGCTGGAAAGCGCCGGATGGTCTGTTTCTGAACTACACGCTGCGTACCGAGGCGGGGGAAGGACCGCTGGCGCTGCTGGCGCTGCGAAACTTTACGCTACCGGAGACGATATTCAGCGTGCGCGCGTCTGCTGAACGTGTTCCGCTGACCGACGATATACACCCGGCGAAGAGGGATACTGAACCTTCTCCCTCACTGATTGGCGTTGACCTGCAACGCCAGGCCTCCTTCGCTCATGGCCGCCCGACGGCCAACTAA

>IR1230_00192

ATGGGCTGGGTACGAACAAAAGCATTGACCATGGAGCAGCCATCGGCTCCTTCACTGACCCGATGGCTGTTTGCCGGTGTACTGATGGCGATTATCGGTGTCCTGCTCTTTATCCTCCATGCTTCCGGCACCGTAAAAATTCTCTCAGTGATAAATATCTGGTGGGTATCCCTGATGCCTGCAGGATGCTGGTTGCTGATTTTTTGTCTGCGTTGCTACCTGTGGGACAGGGATCTGAAAGCGCATCAGTTCTTGCTGAAAGAGGCGGAATACGGACAGCAGAGGTGGGAAGACTGGGCCGGGCGCTGGCTGGCCGTTCTGGGCAGTGCAGTGCTGTTGCCGGATCATATTTCTGCGGCGCATTGGGGGAGTGAGCGGCCTCAGCAATACGGACTTACCCGCAGGATTAATTACCTTCCGGTAGAAGAGCCCGTGCAGTTGAGTGCGATGCATGCGCTGCTGACCAGTATAGAAGAGCGCGTGCAGTGCCTGCCGGAAGAGCTACCGCTGTATGTCACCCTGATCACGGATAATCCCTCGCCTGAGCTGACCAGCAGTTTCAGTAATCTCTGGAAAGAACACATTCCTGGCCGTGCCGTGCCTGATGATATTACCGTTACCGGCTCATTCTCACTGTCGGAAGTGGAGGAGCGCCTGAAGCAGCCTGTACTGACGGTCAATTTACTCCTGGTCATCCAGCTCAACGGTGGTACAGCATACTCCGACGGGCTGGCCGTGCTGCTGCTGACCAGCGATGATGTGGCGCAAAAATACCATCTGCCGCACTCATCACGGCTGTTACGTCCTATGCCCTTAGATATGACGAACTTTGAAGACGATATCACCCTGTTTCTGGAAACCCAGACTGTCGCCTGCCATACGCCCTCCGTTATCGGGGACGCAAAAAAATGGACGGAAAGAAGCGCCGCGCTTATCACGCAGGGCGGCAAAATGCATACCCCATGGAAAGCTGAAGATATCGCGCTGCTGGAAAAATGGTGCGGTATTCCGGGACCCGCAGCGCCCTGGCTCCTGACCGCGCTGGCAGCCGATCTGGTCAGCCTCCGTAAACAGCCCCTGCTGGCGTTATTTTCCAGCGAACAGGAACATTTTATCAGTACCATCACCCCAGGGAGTGAAGATGAATATACCGGGTAA

>IR1230_00193

ATGTCCAAAGGTTTTGTATTACTCGGCGATAAAACCACGCACGGCGGTGCTGTTATTTCCGCATCTTCCACCATGATTGTAAATGGAAAGCCCGTAGCGCTGGTGGGGGATAAGGTCAGCTGCCCGATTCCGGGACATGGCACTAATGCCATTATTGAGGGATCGCCGGAATGGTCTTCCGATGGTAAAGCCATCGTGGTCGATGGCTGCAAATGTCAGTGCGGTTGCCAGGTGATTTCCGGCGCGCCGGAATGCGCGATAGGATAA

>IR1230_00194

ATGATTATTGACGTCGAAAAACTGGTAAAACAACTGGGGAATCCCTATCATGAAATTTACAGTCACGGGTTGATTCCTTATAAAACAAAACCAGTTGGACCAGTGGATGTTGATACAGCATCACTGTATATGAAGCGTGAAGAGCTATATTTATCTTTTGAGAATGATAAAGATAAAGTTTTTAACGAAATTACGCTGACTCTTGAAGATGATGAGAAGATGGATTGGGTCTTCCCTAATGCAATGCCTTTTGGTCTTGAACTTGTAATGACACAGAAATGGGTGTGCGAACGCTTTGGGCTCCCAATGATTTATGGTGAGCCTAAAACGATGGGGAGCTATAGTCGTGGTATTAGTGAGGTATACCCACTGTTGCCACCTAATCAAAATATAGCTGTTTTATTCGTTTATAATTCTGATCTTTTTGTTGTTAGTGTAACTTTCTATTCTATAGAACATGCAAAAGAAATTCAGGCGGCTCTCGGGAAAATACGGTTGTCTGGAAATTGA

>IR1230_00195

GTGACATTAACACTCGAAGATGAAGGTAAAACTGACTGGGTATTCCCCAATTCGATGCCATTTAGTATGGAACCGGTGATGACCCAATTGTGGGTTAGAGAGCGCTTCGGGTTGCCCATGATTTATGCTGATGCGGAAATAATCATGACTATTTATATAGGGGTAAAAGAGGTCTATGCTCTGCCTGCCCCTCATCAATATATCGCTGCCGTATTTACCTATAACAAAGATTTATTTGTAGAAACCGTGACTTTTTACCCACTAGAACGGGCCAAAGAGATTCAGGCAGTACTGGAGAAAAAACAACTTGGTGGCAAATGA

>IR1230_00196

ATGATCATTGACGTCGAAAAACTGGTAAAACAATTGGGTAAGCCTTATCATGAAATTTACAGTCACGGGTTGATTCCTTATAAAACAAAACCTTATGGTGCCATTGATGATGATACAGCCAGATTGAATATTAAACGAGAGGGGATATATTTAGCGTTTATTAATAATTCGGAAAAAAACTTAAAGAAGTGA

>IR1230_00197

ATGAATATTGATGTCGAAAAACTGGTAAAACAATTAGGTAAGCTCTATCAGGATATTCTTAAAAACGGCTTGATTCCTTACAAAACTGAACCCTACGGTGCAGTCGATGATGATGAATCTATATTAAATATGAAACGTGAAGGGATGTTTTTGGTCTTTACGAATGATCCTGATAAAAAATTTAAAGAAATATCTTTAAAGTTAGAAGATGAAGGAAAAACTGATTGGTTGTTTCCTAACCCGATGCCATTTGGTCTGGAGCCGGTGATGACCCTGCAGTGGGTGCGTGCACGCTTCGGGCTGCCGATGATTTATGTTGATGCGAAAGTGGTTATGACTCTCTATAGAGGAGTAAAGGAGTTTTATCCGCTATTAGCTCCTGATCAAAATATAGTTGCGTCGTTTAGCTATAACAAAGATTTCTTTGTAGAAAGTGTGACTTTTTATCCGCTGGAACGAGCAAAGGAGATTCAGGTTGCGCTGGAGAAAAAACGACTTGGTGGCAAATGA

>IR1230_00198

ATGAATATTGACGTCGAAAAACTGGTAAAACAATTGGGTAAGTCCTATCAGGAGATTCTTGAGAAAGGGTTGATTCCTTATAAAACAAAACCTTATGGCCCTATTGATGAAGATGAAGCTGAATTTGATATGAAACGAGAAGGAATTCTTTTGGTTTTCACCAATAATTTCGAGAAAAATCTTACTGAAATTACATTAAGGTTAGAAAATAAAGGCAAAACAGATTGGGTATTCCCCAATCCGATGCCATTTGGTATGGAACCGGTGATGACCCAATTGTGGGTTAGAGAGCGCTTTGGGTTGCCCATGATTTATGCTGTATGCTCTACCAGCTCCTCATCAATATATCGCTGCCGTATTTATCTATAA

>IR1230_00199

ATGAAAGAAGTAACATTAACGTTAGAGGATGAAAGCAAGAGTGACTGGTTGTTTCCTAATCCGATGCCTTTTGGTCTGGAGCCTGTGATGACGCAGCCGTGGGTACGGGCGCGTTTTGGGTTGCCGATGATTTATGTTGATGCGAAAGTGGTTATGACGCTCTATAGAGGAGTAAAGGAGTTTTATCCGCTATTAGCTCCTGATCAAAATATAGTTGCGTCATTTAGCTATAATAAAGATTTTTTTGTTGAAAGTGTGACTTTTTATCCGCTGGAGCGAGCCAAAGAAATTCAGGTTGCGCTGGAGAAAAAGCGACTTGGTCGCAAATGA

>IR1230_00200

ATGACACAAATACTGGGACCCGGAGAGGGAAAAACATCATCAGCCGCAAAAGAGCCGGTTGTTGAACGTAAACCCGCACAGGTATTTTTTGAATCGATGGCGCTGGCCGCCTCAATGAGTGGTTATTATGCAGGGCCAACAGCCACAACGCTGGGAAATTCGCTTTCATTGGGGGCGGGGACTGCGGCAGGTGTCGGAGAAGGCGTCGCGGGCGCACGGGTGCTGGCTGCGCCTAATCCCCTCACCGTTGCTCTGCTGGCTATCTTCTATTCTCCGTCACTGAATGAAGGTGAGACGGAAACGCTACAAAATATACGTGGCGACCAGCTCTATCAGAATCTGATCCATGGCCAGATGATGGTGGGCGCTTTTACCCGTGTAAATTCAACGGTCATTGAGGGGGATTATGTCCCGGAATATGAACTCCGGGAAATAGCCCGACAAAACGGCACAGTGCGCACGAGGGTACGCTTTCGTATTGAAGAAGATCCTGAGTCAGGGGAACTGGTTTCCCGCAGCTATGAGGTGGGGGAAAAAAGCGGGTTGGATCGGGTCCGGGTGCGGTTTGCAAAACAGCTGGATAAAGAGACATGGGGTTTTGAAGATCCTTCAATTAAAGGCACATTTGTCTGGTCCCGGTCTGCCGGGCAGGGAAAATTTGAGTGGGGTTCCAGCCAGACGACTGTTCATGACGGGAGCGCTGGCGGCTCTACCACACCGCCTACACCCATTCCGGAACCGCGGAGCATCTGGGGGTTGCCTAACCCGGCCCCGGAATCGTTACCGCCCGTACCGGGAACGCCGATCCCGGAGGAACAGGAACCAAATATTGAAACATTGCCCATCGAAGACCGGGATTTCGATGACTTTATTATTGTTGACCCGATGGGGGTTGTCCCGGCAATCTATGTGTATTTTAAGAAAGCCCCTGTGGAAGAGTATGAGGTTGACTATTATGAAAATTTTGAAGGCCGTTCAAGGCAGGGAAAATATCAAGTCGATCATATTCCATCTAGAGATGCGGTTCGTGTATATTTAGAGGATTTATATCCTGATGAGGGAAGTAAATATATAGATAAGATGGTTGATAAGGTTGCTTCAGTTGCTATTCCAATAGCTGTTCATCAAAAATGTAGTGAAACCTATGGCGGGCGTAATAATCGTAAGGTAGAGACAGAAAGCGGCGAGATGATAACTAAAAAAGAGCTAGATGCTCGTGACTTAGAAGCTGCCGTCAATGCCAACTGGGATGCGAATGCTGAATGCCTTAAGAATGAATATGGTATGAGTAACGAGAAAATCGAAGAGATCAGAGCAAAACTACATAAGTTAAACCGAAATGTGGGGTTATATTAA

>IR1230_00201

ATGAAAATGAAGCCTATCTGGATATGTTGGTGGCAAATGAATATTGATGTCGAAACACTCGTGAAACAATTGGGTAAAGATCATCAGGAAATTTATGATAGTGGACTAATTAAATATAAGACAAAACCAACTGCCACAGCGGGCTATGATACAGCTACATTAGACATGAAACGAGAAGGATTGTTTTTCTCTTTCGAGAATGATAAAAATAAAACCTTCAAAGAAATTACATTAACATTAGAAGATGATGACATAACAGACTGGGTCTTTCCGAATTCAATGCCTTTTAATCTTGAACCGGTAATGACTCAAAACTGGATGCGTGAGCGTTTTGGGTTCCCAATGATTTATGGCGAGCCTAAAACTATGGGGAGTTACAGTCGTGGTATCAGTGAAGTATACCCACTGTTACCACCTAATCAGAATATTGCTGTTTTATTTGTTTATGACGCCGACCTTTTTGTTGTTAGTGTGACTTTTTATTCTATGGCGCATGCAGAAGCAATTCAGGCTGCGTCAGAGCGAAATAGATTAATGAATAAATAA

>IR1230_00202

GTGTGGTGTATTTATCAAATGAGTATTGATGTCGAACAGCTTATTAAGAAAATTAGTCGACCTTATCAGGAAATAGTTGAGCAAGAATTGATTCCCTATAAGAATAAACTGCATGGCCCGGTTGATGAAAATGAAGCCTATCTGGATATGTTGGTGGCAAATGAATATTGA

>IR1230_00203

ATGATCATTGACGTCGAAAAACTGGTAAAACAACTGGGTAAGCCTTATCATGAAATTTACAGTCACGGGTTGATTCCTTATAAAACAAAGCCTTACGGTCCTATTGATGAAGATGAAGCTGAATTAGATATGAAACGAGAAGGAATTCTTTTGGTTTTCACCAATAATTCCGAGAAAAATCTTACTGAAATTACATTAAGGTTAGAAGATAAAGGCAAAACAGACTGGGTATTCCCCAATCCGATGCCATTTGGTATGGAACCGGTGATGACCCAATTGTGGGTTAGAGAGCGCTTTGGGTTGCCCATGATTTATGCTGATGCGGAAATCATCATGACTATTTATATGGGGGTGAAAGAGGTCTATGCTCTACCTACTCCTCATCAATATATCGCTGCCGCATTTACCTATAACAAAGATTTATTTGCAGAAACCGTGACTTTTTACCCACTGGAACGGGCCAAAGAGATTCAGGCCGTACTGGAGAAAAAACGATTAGAGAGTTAA

>IR1230_00204

ATGAGCTTGAATGTTGAGAAGTTGATAAAGAACCTTGGGAAATCGTATCTGGATATTTATGAGCAAGGATTGATCCCCTACAAAACAAAACCAAGTGGTACGGTTAGTGATGACATTTATAGATTAGATATGAAACGAGAGGGGGTATTTTTATCATTTTTTAATAATCAAGATAAAAATCTTAAGGAGGTGACCTTAAGGTTAGAGGATGAGAATAAAACTGACTGGTTATTTCCTAACTTGCTGCCATTTGGTCTTGAACCAGTAATGACACAGCGATGGGTACGAGACCGATTTGGTCATCCGATTACTTACGTTGATGCCTGCGTGATTATGACTATTTATGTCGGGGTGGAGGAAACATATATTTTACCGACTCCGAATCAAAATATTGCAGCGGCATTTTCTTACAATAAAGATTTTTTTGTTAACAGGATCACATTTATTCCGGTTGAACAGGCGAAAGAAATCCAGTCTGCACTTGAGAAAAAACGACTTGGTGGCAAATGA

>IR1230_00205

ATGACACAAATACTGGGAACCGGAGAGGGAAAAACATCATCAGCCGCAAAAGAGCCGGTTGTTGAACGTAAACCCGCACAGGTATTTTTTGAATCAATGGCGCTGGCTGCCTCAATGAGTGGTTATTATGCAGGGCCAACAGCCACAACGCTGGGAAATTCGCTTTCATTAGGGGCGGGGACTGCGGCAGGTGTCGGAGAAGGCGTCGCGGGCGCACGGGCGCTGGCTGCGCCTAATCCCCTCACCGTTGCTCTGCTGGCTATCTTCTATTCTCCGTCACTGAATGAAGGTGAGACGGAAATGCTACAAGATATACGTGGCGACCAGCTCTATCAGAATCTGATCCATGGCCAGATGATGGTGGGCGCTTTTACCCGCGTAAACTCAACGGTCATTCAGGGGGATTATGTCCCGGAATATGAACTCCGGGAAATAGCCCGACAAAATGGCACAGTGCGCACGAGGGTGCGCTTTCGTATTGAAGAAGATCCTGAGTCAGGGGAACTGGTTTCCCGCAGCTATGAGGTGGGGGAAAAAAGCGGGCTGGATCGGGTCCGGGTGCGGTTTGCAAAACAGCTGGATAAAGAGACATGGGGCTTTGAAGATCCTTCAATTAAAGGAACATTTGTCTGGTCCCGGTCTGCCGGGCAGGGAAAATTTGAGTGGGGAGCCAGCCAGACGACTGTCCATGACGGGAGCGCTGGCGGCTCTACCACACCGCCTACACCCATTCCGGAACCGCGGAGCGTCTGGGGATTGCCTAACCCGGCCCCGGAATCGTTACCGCCCGTACCGGGAACGCCGATCCCGGAGGAACAGGGACCAAATATTGAAACATTGCCCATCGAAGACCGGGATTTCAATGACTTTATTATTGTTGACCCGATGGGGGGTGTCCCGGCAATCTATGTTTATTTTCAGAAAGCTTCAGTTAGAGATTTAGAGGTTGACTATTACGGAAATTTTGAAGGTCGTTCAAGGCAGGGCCTATATGAAGTGGATCATATTCCTTCAAAGGAAGCTGTTAGGATATATTTGAAAGGGACATATCCAAACCTGAAAGATGACATTATAGAACAAATGACTGACAGAGTGGCTGCTGTCGCTATACCGATAGAAGTACACCGGCAATGTAGTGAAACCTATGGTGGGAAAAACAATAGTAAATTTAGAACACAAGACGGAGGATTAGTCACTCAAAAAGTTCTAGATGCCAGTAATTTAGAAGCTGCAGTTGATGCTAACTGGGATGTCAATGCTGAGTGCCTTAGGAATGAATATGGTGTAAGTGATGAAAAAATTGAAGAGATCAGGGCCAAACTACATGAGTTAAACCGGAAAGCGGGGTTATATTAA

>IR1230_00206

ATGAATATTGATATCGAAACACTTGTGAAGCAATTGGGTAAAGATTATCAGGAAATTTATGATAGTGGGCTAATTAAATATAAGACAAAACCAACTGCCACAGTGGGCTATGATACAGCTACATTAGATACGAAACGAGAAGGATTATTTTTATCTTTCGAGAATGATAAAAATAAAACCTTCAAAGGAATTACATTAACATTAGAAGATGATGACATAACAGACTGGGTCTTTCCGAATTCAATGCCTTTTAATCTTGAACCTGTAATGACTCAAAACTGGATGCGTGAGCGTTTTGGGTTCCCAATGATTTATGGCGAGCCATAA

>IR1230_00207

ATGAAAATTGATGTTGAAACACTTATAAAACACTTAGGAAAACCGTACCAGGAGATTTTTGAGAAAGGATTGATTCCTTATAAAACAAAACCCCATGGTCCTATTGATGAAGATGAAGCTGACCTGGATATGAGACGTGAAGGAATGCTTTTGGTCTTTGTTAATGATTCAGAAAAGAAACTTAAAGAAGTCACACTAAGATTAGAAGATGAGGGTAAGACAGATTGGTTATTTCCTAACCCTATGCCGTTTGGTCTGAAACCGGTGATGACCCAGCAGTGGGCTCGTGAGAATTTAGGCTTGCCGATGGTTCATGTTGAGGCAAAGATTGTTATGACTATTTATATGGGGGTTAAAGAAATTTACGCTTTACCAATGCCCAACCAGCATATTGCAGCAGCGCTCACATATGATAAAGATTTTTTTGTAAAAAAATCACTTTTTATTCTTTGGAGCGAGCCAAAGAGATACAGGTCGCGTTACAGAAAAAGCGACTTGGTGGTAAATGAATATTGA

>IR1230_00208

ATGACACAAATACTGGGAACCGGAGAGGGAAAAACATCATCAGCCGCAAAAGAGCCGGTTGTTGAGCGTAAACCCGCACAGGTATTTTTTGAATCAATGGCGCTGGCTGCCTCAATGAGTGGTTATTATGCAGGGCCAACAGCCACAACGCTGGGAAATTCGCTTTCATTGGGGGCGGGGACTGCGGCAGGTGCCGGAGAAGGCGTCGCGGGCGCACGGGTGCTCGCTGCGCCTAATCCTCTCACCGTTGCTCTGCTGGCTATCTTCTATTCTCCGTCACTGAATGAAGGTGAGACGGAAACGCTACAAAATATACGTGGCGACCAGCTCTATCAGAATCTGATCCATGGCCAGATGATGGTGGGCGCTTTTACCCGTGTAAACTCAACGGTCATTCAGGGGGATTATGTCCCGGAATATGAACTCCGGGAAATAGCCCGACAAAACGGCACAGTGCGCACGAGGGTACGCTTTCGTATTGAAGAAGATCCTGAGTCAGGGGAACTGGTTTCCCGCAGCTATGAGGTGGGGGAAAAAAGCGGGCTGGATCGGGTCCGGGTGCGGTTTGCAAAACAGCTGGATAAAGAGACATGGGGTTTTGAAGATCCTTCAATTAAAGGCACATTTGTCTGGTCCCGGTCTGCCGGGCAGGGAAAATTTGAGTGGGGTTCCAGCCAGACGACTGTTCATGACGGGAGCGCTGGCGGCTATACCACACCGCCTACACCCATTCCGGAACCGCGGAGCATCTGGGGGTTGCCTAACCCGGCCCCGGAATCGTTACCGCCCGTACCGGGAACGCCGATCCCGGAGGAACAGGAACCAAATATTGAAACATTGCCCATCGAAGACCGGGATTTCGATGACTTTATTATTGTTGACCCGATGGGGGTTATCCCGGCAATCTATGTGTATTTTAAGAAGGCTCCTGCCGGATTTCTAGAAACCGGTTATTATAACGACTTTGATGGGAGGTCAAGAGAAGGAATGTACGAGGTTGACCATTTACCATCAAAAGCGGCGGTTAGAGAATATTTGATTAATAAATATCCTGAAGCAGAAAAGGATGATATAAAAAAATTACTTGGTAAGGTTGCAGTCGTTTCTATCCCTATAGATGTACATCGTGACTGTAGTGAAACCTTCAGAGGGCGTAATAATAGTAGGATAGAAACAGAAAATGGTGAGACAATATCTAAAAAAGAACTTGATGCTCGTGATTTAGAATTTGCCGTTGACTCGAATTGGAATGCTAATGCTAAGTGTCTTAAAGAAAGATATGGCATAAGTGATGAGAAAATTGAAGAAGTTAGAGCAAAATTGCATGACTTAAACAGAAAAGTGGGGTTGTACTAA

>IR1230_00209

ATGGATACCTCTTCAATAATTACCGGCACCACCCTTAACCGTTACCAGCTGGATATTCCTTCATGTACCGCATCGCTGGATGTGGAAGAATTCAGTGGGGCAGAAAAACTGAGTGAACTTTATTATTACACTATTACGTTTACCAGCGCAGAGAAAAATATTGATGCCGCACAGCTCCTGAGCAAACCCGCCATGCTGACGATGGGCGGCGGCGCACTGCAGCAGCTGGCGGACTGCAAACGCGTCCATGGCGTGGTGACTGCCTTCCGGCGTATCAGCAGTTCAGAAGATCAGTCGAAATATCAGATAACCCTCGAACCCTTCCTCTCCTTACTGGATAAACAGTTTCGCAGCCACCGTTTTTTCGTGAATAAATCGGTGCCGGAGGTGGTGGAGCAGGTATTGCAGGAACATCACCTGCATGACTGGGAGTATGAGTTTAATCTCAAGCAACACTATCCGCGGCGCGAGCAAATTAATCAGTATCAGGAGAGCGACCTGGCATTTATCCAGCGCCTGCTGGCAGAGGTGGGGATATTTTATTTCTTCACCCTGCAGGAAGAGGCCCAGAGCGAAGTGGTCCATTTCGCCGACGCGCAGCGGGCGCTGATGTTCGATAAAACACTTCCTGTAAACAGCCCGTCCGGGATGAGCGACAGCGGTACGGAATCGATATGGGGTCTGAACATCACGCACAATGTCGTAGAGGCGAACGTCACTACCCGGGATTATAACCCCCGCGATGCGCAGAGCGTTCTCCAGTCGGCAACAGCGGATATGACCCGGGGAAATGGTGAAGGCATCACTTACGGTGAGGTATACCACTATAAGCTTCGCCACCGGGAGCGTGGCGATAAAATCGACCCGCAGACGGAGACCGCCAACTTCTATGCCCGCCTCGACCATGAGCGTTTTCTGGCTCACCAGACGCTTATTACCGCGAGCAGTACTGCAGCCTGGCTGGCGCCGGCCCAGGTGCTGACCGTTACCGACAGCCTGCCGTCGACCCTGCCTGCGCCCGTACAGGATCCGCTGTTAATCACCGGCACCGGTTTTACCGCCAGCCGCCGGGAGGCGCTGCGGGTGTCCCTGCTGGCGGTGCCCTACAGCGAAACATTGTGCTGGCGTCCGCCGCTGCTGCCGCGCCCGAAGGTGACTGGCACCATGACGGCGCGGGTGACCAGCGCGAAGGCGAATGATATCTATGCCTGGCAAGATGCGTCCGGCCTGTACCGGGTGAAATTTGACGCTGACAGAGAGGAGAAAGGGCAGGGTCAGGAAAGCATGCCGGTGCGTCTCGCCAAACCCTACGGCGGCGACGTGTACGGCTTTCACTTCCCGCTGATCCAGGGCACGGAGGTGGCGATTGCTTTCCACGAAGGCGACCCTGACCGGCCGTATATTGCGCATGCGCTGCACGACTCGCGGCATGTCGACCCGGTGACGGAGAAAAACAGCACCCGCAATGTGATTCGTACTCCGGCCAATAATAAGCTGCGAATGGAGGACAAGCGCGGCGAGGAGCATATCAAGCTCAGCACCGAGTACGGCGGCAAGACGCAGCTGAACCTGGGGCATAACGTGAATGCGCAGAGGGAGTTGCGGGGCGAGGGTGCGGAACTGCGTACGGATAAATGGGTGAGCATCCGGGGCGGTGCGGGGGTGTTTATCAGTGCGGATAAACAGCCTTCGGCAGGCGACAGGATGCTGGCAATGGAGGAGGCGATTGCGCAGCTTGAAAATGCCCTGGGTATTGCGAAAAGCCTGGCGTCGGCAGCGGAATCGGCTCAGGCCCTGCCATCGGATACCGGCAACCAGCAGACGCTGAACGATGCGCTGAAAGAGCTGGCGCAGCCGGGGATAGTGCTGAATGCGCCGCAGGGCGTGAGCATCAGCAGTCCGCAGGCGGTACGGTTGTCTTCGGGTAGCGCCAGCGTGGGGATTGTGTCACAGCAGAATACGGACATCAGTGCGCTGAAACGTTTTACGGTGGCGGCGGGAGAAGCCGTCAGCCTGCTGGCGCGAAAGGCGGGGATGAAGCTGTTTGCGGCGAAGGGAAAGATTGAAATCCAGGCGCAGGATGATGCGCTGGAGGCGACGGCGAAAAAGGATATCACCGTGACCAGCGTGGAAGGGCGAGTGGAAATCACGGCGGCGGAGGAACTGGTGGTGAACTGTGCGGGAGCGTATATCAGGCTAAGTGGTGGCAATATCGAGCTGGGCTGCCCTGGGAATATTCTGCTGAAATCGGCCAATGTGCAGAAAATGGGTAAGGCGGATTTTCGGGTGCCTCCGCTGGAATTGCCTAAAGGGTTCGAAGAGCGTTTTACTGTCAAAGATCAGAAGACCGGTGCCATTGTGCCATTTGCCCGTTACCGCATCACCACTGAAAAAGGCCCGGTATTCGAAGGCCGGGCAGATGCTGAGGGGAAAACGGCCAGCGTTTATACCGCATTGCCTGAGTCAATAAAAATAGAGCTACTGTGA

>IR1230_00210

ATGCGTGATAACGCCCGTCGTCTGCTTACGATACTGACCGTCATCCTGGCCCTGTGGCTGGTGCTGGGCTTCTGGCCGCTGTCCATCGGCAACCAGGTGATCTTCAGTCTGTGCATCCTGCTGGCGGGCGGCGCGGCGCTCTGGCGTCAGCGTCGCCGGGCAGTCAGCCGTCAGCGATCGGAAATCGTCCTGCCGCCCGAGGATTTTCAGGGGGCGGTGGTTCTGGTCTGCGGGGATACCGACGGTCTGTTTCCCGGGCGTTCGGCGCACGGCGAAACCCGTCACGGCTGGTATCTCCGGGGTGACAGCGCGGAGCAGTTGCCGCGGCTGGCGCAGTACCTGGCGGCGGCGCGTCCGGCGCTGGTATCGCAGGTTTCCGTGCTGCTGGCGGTGGTACCGGAGCACCACCCCTCCGGGGAGCATCTGGCGCAGTCGCTGCGCGACTGGCGGCGGAGTATCGTTCAGTGCCGAACCTGGCTAAACGGTCTGCCGCCGGTGTGGAGCGTGTTCTGGGTGACGCCGCCAGGCGGCCAGGCCGCGGAAAGCCGCTGGTTTACCATCACGCCGGAGAGGGCTGGTCTCCAGGTGCAGCTGAAGGGACAAGCGCCCCAGGCGGTTGCCGGGTGGCAGCGGGAGGGCAGCTCTGCCTCACGCCTGCATCAGACGCTGTGGCTGGAGAGCATTCTGACCTTGGCCGAAAACGCTCTTTTCCGGCCATTCCGCGCCCGGCAGGCAGAACTTCCTCCGCTGAACCTGTGTGCCGCGGGCATCTGCCTTACGCCGGTGGCGGCGGTGGCGAATAACCTCTGGCAACAGCAAATCGCCGGGATCACCACGCTCTCCCCGGGCAACGACGCGGCGCCAGGTCCTCACCCGCTGCCGGATCTTCTTCTGTCGTCACTGCCGCACCGCCACGGGGTCAGCCGCCGGATGCGCGATGCCGGTCTTGCCGCTGGGGTGGGCTTCCTCTTTCTGGCGCTGGCGATGCTGGCTTCCTTTATCAACAACCAGCGCCTGGTGCGCAGCGTCGGCGATCATCTGGCGGTGTATCACCGCCTCAGCGGCACGCCGCCGACGCCGAAGCTGCAGGCGCAGCAGCGTCTGCGTGCCGACAGCCGCCTGCTGGATGACTGGTTGCGCCGGGGGGAGCCGCTGCGTTATCGGCTGGGGCTGTATCAGGGGGGGCGGCTGATCCCCTTCGTTGAAGCCGCTATCAATGACTGGGCGCCGCCCCCGCCGCCGCGTCCGGTCATTAAGCAGGTTGTGCAGGGGCCGCAGACGATCCGCCTCGATAGCATGGCGCTGTTCGATACCGGCAAATCGACGCTGAAACCGGGTTCGACAAAGCTGCTGGTGAACTCGCTGCTGGGCATTAAGGCCAAACCCGGCTGGCTGATCGTGGTGGCAGGCCATACCGACAGCATCGGCAACGATAGATCCAATCAACAACTCTCCCTGAAGCGTGCCGAGGCGGTCCGCGACTGGATGCGCGATACCGGCGATGTGCCGGTGAGCTGTTTTGCGGTGCAGGGCTACGGCGCAAGCCGTCCTGTCGCCAGCAACGAGACGCCAGAGGGGCGGGCGCAAAATCGCCGGGTGGAGATCAGTCTGGTCCCGCAGAAGGACGCCTGTCTGACGCCGGGCACGGCCAATACCTCAGGAGCAGAGACTAACGGATTAAAAAGCGAAACCGAGTAA

>IR1230_00211

ATGAATACCTCTGAAAGCGATCTGATCAACAAAACTTTTTACCCGGGCTGGCTGATGGTCAGCCAGTTACGCTGCGGCCAGCCGGTGACGGATGGCGAGGCGCTCTATCGCCAGGCCTGTCGATGGGTGACCGAGGCGCGCGAGGCGCTGACGGCGGGGGGAGTCAGTGAAGCCAGCGCTGAGCAGATGCTCTATGCCTACTGCGCGCTGCTCGATGAGAGCGTCCTCAACCGCGCCAGTCAGGATGATGGCTACCGCAGGTGGCGCAAGGATCCGCTGCAGGCGCGCTTTTTCAGCACTCTCAACGCCGGGGAAGAGCTCTGGGAACGGATCCGCCAGCTGCTGCGAGAGCCCACGGCGGATGCGGCAGTGCTGACTTGTTTCTATCGTACTTTGCAACTGGGGTTTGTCGGACAGTACCGCGCCGAGGATGACGAACGGCGTGAGGACGTGGCGCAAGCGCTTGGCTCGCGGGTCCCGCCGTTCAGCCTGACCCAGGAGGCGCCGGTGGTGGTTCGTGCCTCCCGGCTGCGCAGCGGACGGCGGATGTACTGGTGCGGCTGGGCGGCTGGCATTGTGGCGCTGGCGGCGCTGTGGTTGACCTTCTCCGCCATGCTGTCACAGATGGTGGCGCAGATAGCAGGGCAGGGATAA

>IR1230_00212

ATGAAGATTTTCCGCCCATTATGGCGCGACGGGGCTTTTCTGGTCCCGCAGCAGTTTCAGCAACAGGCCCGCTGGGACGCGCACGTCGCCGATACGGTCTCCCGCATGGCGCTCGCCCACCCGTGGGGGGTATTGCGCGCGGAGTTTGACGCGAGCGCGCTCACCCTTTCCCGGCTCAACGCTACCCGGCTGATCGTGCGTTTCGCCGACGGAACGCTGATTGACACCGAGCTGGCGGATATTTTGCCACCGGTCCGCGATGTATCGGACGTGATGCAGGACAGCGTGGAGGTTCTGCTCGCTCTGCCGCTGCTCAGCGCCAGCGGCGGCAATCTTGATGACGGCCAGGAGAGCGCCCGCCCGCGCCGCTGGCGCGCCGAGCAGGTGACCGTCCAGGAGCTGGCCGGCCATGAACGCAGCGAGCTGGCCGTTCTGCGGCACGCGCTAACGCTGCGTCTCTCCACCGAGGAAAATGCAGCTTTCCTCACCTGCCCGGTAGCGCGTCTGGTGCGCGACGCCCAGGGGCAGTGGATCGTTGACCCGGAATTCATCCCGCCGCTGCTGTCGCTGGCGGCAAGCCCGACGCTGGTTAGCGAGCTGGGTGAGCTGTTACATCGCCTGCAGGCCCGACGCAGACGCCTGATGGCCATGCGTCGTGAAAGCAATGCGCGGATGGCTGATTTTGCCGTGGCGGATGTTTCCCTGTTCTGGCTGCTCAACGCGCTGAACAGCGCTGAACCGGTGCTCAGCGAGCTGCATCAGGACCCTTCGCGCCATCCGGAGCTGCTCTATCGCGAACTGGCCAGGCTGGCCGGCAGTCTGCTGACTTTCTCCCTTGAACATCATCTGGAAGCAATCCCCCGCTACCGACATGCCTCGCCAGAGCAGGTATTCCCACCGCTGTTTGCCCTGCTGGATACGCTGCTCGAAGTCAGCCTGCCCTCAAGGGTGATCGCCATCGTTCTGGAGCAGGGCGCCGACCGGGAGATCTGGCGGGGCAGGCTGCACGATGCGCGTCTGCGCGAGGGGGCGGATTTTTATCTGTCGGTGCGCTCTTCACTCCCCCCTCACCAGCTACAGAGCCGCTTTCCGCAGCTCTGTAAGGCGGGTAGCCATGATGATGTCGCCGAGGTCGTCAATATTGCCCTCAGCGGCATCGCGATCAAACCGCTGAGCCATGTGCCGGCGGCCATTCCGCTACGTCTGGAAAACCAATATTTCGCTCTGGATCTCTCCACCGATGCCGCCCGCGCGATGCTGGAGGCCGGCAACTGCACCTTCTATACCCCGGAATCGCTTGGCGACGTGAAACTTGAACTCTTTGCGGTACTGCGGTCATGA

>IR1230_00213

ATGAAACCACTATTTGTCTATGGGACGCTCTGTCCCGGTCGCAGCAATGCGCATATTCTGGAGGCGATCGGGGGCGAGTGGCGTCCCGGCTACGTCACCGGCACCTTTTACGCGCGCGGCTGGGGCGCAGCCGCCGATTTCCCGGGGATCGTCCTCGACGCTCACGGCCCACGGGTCAACGGCTATCTGTTCCTGTCCGATCGGCTGGCGGCGCACTGGCCCATGCTGGACGACTTTGAGGAAGGGTACGATCGGGTGCCGGTGGAAGTGACCACCGACGATGGACAGCAGATCAGCGCCTGGATTTATCAGCTTCAGCCCCGGGGGTAA

>IR1230_00214

ATGACAAAACTCACCTTACAAGAGCAGATGCTCAAAGCCGGCCTGGTCAGCAGCAAAAAAATGGCTAAAGTCCAGAGGACGGCGAAAAAATCCCGCGTTCAGGCCCGTGAGGCCCGGGAAGCGGTGGAAGAAAATAAAAAAGCCCAGCTGGAGCGTGATAAACAGCTTAGCGAGCAGCAAAAGCAGGCGGTGCTGGCGAAAGAGTATAAAGCCCAGGTGAAGCAGCTGATCGAGATGAACCGCATTACGGTCGCCCGGGGCGATATCGGCTTTAACTTTACCGATAATAATCTGATCAAAAAAATCATGGTCGACAAGCCCACCCAGACCCAGCTGATTAACGGCCGTCTGGCGATTGCCCGCCTGGCGGTGGATAACAAGCCCGAAGGTGAATATGCGATTATTCCGGCGGTAGTGGCTGAGAAAATAGCCCAGCGCGACGCCAGCAGCATCGTGTTGCACAGCGCCCTGAGTCAGGATGAGCAGGATGAAGACGATCCGTACGCCGACTTTAAGGTGCCCGACGATCTGATGTGGTAA

>IR1230_00215

ATGTCCACCATTATCGATACCTTTGTCGCGCCGCCGTGTCGCGAACAGATCACCCTCTTGTATCAGGACGAACACCTGCTGCTGATCAATAAGCCCGCCGGGCTGCTGAGCCTGTCGGGCAAAGATCCGCGCAACCTCGACTCCGTGCATCATCGGCTGGTACAGCGCTTTCCGGGCTGCACCCTGGTGCACCGCCTGGACTTCGGCACCTCCGGCCTGATGGTGATCGCCCGCAACAAGGGCATTAACGCCTTGCTGTGCCAGCAGTTCAGCCAGCGCACGGTGGACAAGGTCTATACGGCGCTGTTGTGCGGCCATCTGGCGGAAGATGAGGGGATAGTGGAGGCGGCAATCGCCAAAGATCCGGCCCGTTTCCCGCGCATGGCGCTCTGCGCCCGCCACGGCAAACCGGCGCGTTCGCGCTATCGGGTCATAGACCGCCTGTATCAGGCGCGAGAAGGCGAGAGGGCGCTGGCGTTAACCCGGGTGACGCTCACTCCGGAGACCGGACGGACCCATCAGCTGCGTATTCACTGCCAGCTGCTGGGCTATCCGATCCTTGGCTGCGATCTGTACGGTGGCCGGGAGCTGCCCGGCACGGAACAGGCTCCGCGGCTGATGCTGCACGCCAGCGAACTGCGCTTTGTCCACCCCGTCAGCCATGAGCCGATGCATATCCAGCAGGCGAGCCCGTTTTGA

>IR1230_00216

ATGGCGATGAACGGAACAATCACAACGTGGTTTAAAGATAAAGGTTTTGGATTTATCAAAGATGAAAACGGCGAAAATCGTTATTTTCACGTGATTAAGGTCGCCAATCCTGATCTTATCAAGAAAGACGCGGCGGTGACTTTCGAACCTACCACCAACAATAAAGGGCTGTCGGCCTACGCGGTGAAGGTCATTCCGGAAAGCAAATATATCTATATTGCCGGCGAGCGGTTGAAGCTGACCTCCATCAAATCCTATGTGGTGTACCGGGAAGAAGAGCCGGTCGAGACCCGCGTCGATAAAGAAAACGCCGTGCTGTCGGTCGGGCTGCTGATGAACAGCATCCGGCCAAAAGCCACGGTGCAGCCGGGAGAGATGCGCTCGGTGAAAAAGCTGGCGATCACCACCTTTCAGGGGACAACCCTGATCTTTTCGGAAGACGAAATCGATATCGATGCCACGGTAAAACTGCTTAAGGTGTAA

>IR1230_00217

ATGCCCGTAAATTTTGACTTCAACGATCTCTACGCCTTTCGGGCCCTGATGGAATATGGCAGTTTTCGGCTGGCGGCCGAATCCATCTGCCTGTCGCAGTCGGCCCTTAGCCGACGCATCGAAAAGCTGGAGACGGCGCTGGGCAACCGGCTGTTCGATCGCACCACCCGGCGGGTGACCCTCACGCTGTATGGGCAAAATTTTGCCGAACGTTCGGAGCAACTGCTGGCCCATGTCGAGACGGTGCTGGCGGATATCAGCCAGGTCAGCAAGGCGCGCACCGGGCTGGTGACGGTGGCGACAGTGCCTTCCGCCGCCTACTACTTTATGCCGGAAATCATCCGCAGCTTTCAGGCGCGTTATCCCCAGGTGCGCATCCGGTTGATTGACAGCAGCGTCGGGAATGTGATCGAGGCGGTCAGCAGCGGACAGGCTGACTTTGGCCTCTGCTTTGCCAAAAACCTGCCAGCCAGTATCGAATTCACCCCTCTTGCCGACGACCGCTATGTGGCCGCCTGTCGGCACGATCATCCGCTGGCGCGCAAGACGCACCTAAGCTGGCAGGCGTATTTTGAACAGGATTATATTGGCCTGGATCGGGTCTCCGGCAACCGGACGCTGCTTGACCGGGAGCTGGCGCATCTGACGCCGGCGCGGCCAAGCATCTGCGAAACCCGCCACGTCACCACGATGCTGGGGATGGTGGAAGCGGGCATCGGCATTGCCGCCGTGCCCGCGATGTCGATGCCCGCCGGGGAGCATTCCGTTCTCCGCGCTGTGCCGCTCACCGACCCGGTGGTCACGCGTACGGTAGGCCTGATCCGCCTCAGCGGCCGTATTCAGTCCTACGTGGCGGCAGAGCTGGAGAAGCTGATTATTGAACAGTATCCTTCTGGCTGA

>IR1230_00218

ATGCGTAAAACGACAGGAACCCTGCTAGCCACGCTGCTGCTGGCCGCCACCGGCGGCAGCGCGCTGTCCGCTGAGGTCACCGTGATGATCTCCGGCGGCTTCAAAGCGGCGCTGGAAAAGCTGGCCCCGGCGTGGGAAAAACAAACCGGCAACCACCTCGTGGTGATCCCTGGCCCGTCGATGGGAAAAACGCCGCAGGCGATCCCTAACCGTCTGGCGCGCGGCGAACACGCGGACGTGGTGATCATGGTCGGGGACGCGCTGACCAGCCTCGAGAAAGCGGGCCGTACGCAACCGGATTCGCGCCGGGAGCTGGCCGACTCGCCGATCGGCGTGGTGGTGAAGGCGGGGGCGCCGCTGCCGGCTATCCACAGCGCAGACCAGCTGCGGGCGACGCTGCTGGCGGCGCCATCGGTCGCGTACTCCGACAGCGCCAGCGGACGCTATGTCAGCTCGACGCTGTTCCACACCCTGGGCATTGATGACGCGATGCAGAGTAAAGCGCAGATGGTGGAGCGCATTCCGGTGGCCTCGGAAGTGGCCAAGGGACGGTATGCGATCGGTTTTCAGCAGGTGAGCGAACTGCTGCCGGTGCCCGGCGTAACCTTCGTCGGCGAACTGCCGGATAACCTGCAGTACATCACCCGCTTTGCCGGGGCGGTGACCATCAGCGCCGACCACCCGCAGGAAGGCAAGGCGCTGCTGACGTATCTGGCGTCACCGGCGGCGCAGGAGACCATCCACGCCACCGGTATGCGAAGCGTAGCCGCCGCCGCGCCGGTCAGCCAGAAGGATACTGTTCAATAA

>IR1230_00219

ATGCATTCAACAACCTCACAGATGAGTACCCGTGACCGGATCGGCGCCATTCTGCGCGTCACGTCCGGTAACTTTCTCGAACAGTTCGACTTTTTCCTGTTCGGGTTTTATGCCACCTACATCGCCCATACCTTTTTTCCGGCGAGCAGTGAATTCGCCTCGCTGATGATGACCTTCGCAGTCTTTGGCGCCGGCTTTTTGATGCGCCCCATCGGCGCTATCGTACTTGGCGCCTATATCGACAAGGTGGGGCGGCGCAAGGGGCTGATCGTCACCCTGTCGATCATGGCCACCGGCACTTTCCTGATCGTGCTGATCCCCTCTTATCAGACCATTGGCCTGTGGGCGCCGCTGCTGGTGCTGATCGGCCGTCTGCTGCAGGGCTTTTCCGCCGGCGCCGAGCTGGGCGGGGTGTCGGTCTATCTGGCCGAGATCGCCACCCCGGGCCGCAAAGGCTTTTACACCAGCTGGCAGTCGGGCAGTCAGCAGGTTGCCATCATGGTGGCAGCCGCGATGGGCTTTGCCCTCAACGCGGTACTGGAGCCGAGCGCTATCAGCGACTGGGGCTGGCGTATTCCGTTCCTTTTCGGCTGCCTGATTGTTCCGTTCATTTTTATCCTGCGCCGTAAGCTGGAGGAGACCCAGGAGTTTACTGCCCGCCGCCATCATCTGGCGATGCGCCAGGTATTCGCCACCCTGCTGGCGAACTGGCAGGTGGTTATCGCCGGGATGATGATGGTGGCGATGACCACCACCGCGTTCTACCTGATCACCGTCTATGCCCCGACCTTTGGTAAAAAGGTGCTGATGCTCAGCGCCTCTGACAGCCTGCTGGTCACGCTGCTGGTGGCGATTTCCAACTTCTTCTGGCTGCCGGTGGGCGGGGCGCTGTCCGACCGCTTTGGCCGCCGGTCGGTGCTGATCGCCATGACCCTGCTGGCGCTGGCTACCGCCTGGCCTGCGCTGACCATGCTGGCGAACGCCCCGAGCTTTTTGATGATGCTGAGCGTGCTGCTGTGGCTGTCGTTTATCTACGGCATGTACAACGGGGCGATGATCCCGGCGCTGACCGAAATTATGCCCGCCGAAGTGCGCGTGGCAGGTTTCTCGCTGGCCTACAGCCTGGCGACCGCGGTGTTTGGCGGCTTCACGCCGGTGATTTCCACGGCGCTGATTGAGTATACCGGTGATAAAGCGTCTCCCGGCTACTGGATGAGCTTTGCCGCCATCTGCGGCCTGCTGGCCACTTGCTACCTCTATCGCCGTAGCGCCGTTGCGCTGCAGACGGCACGTTAA

>IR1230_00220

ATGAATATTATGCATCCTGTCCTGCGCCGGATTGACCTCAACCTGTTGCCGGTCTTTGACGCGGTCTATCGCTCCCGCTCGGTACGCCTGGCGGCGGAAGAGCTGGCGATGAGCACCTCGGCGCTGAGCCATGCGCTTTCCCGGCTGCGCAGCGCCCTCAACGATCCGCTGTTCTACCGCGAAGGCCACCGGATGTGCCCCAGCGTCTATGCCAGCCAGCTGGCGCCCTCTATCGCTTCGGCGCTGAAGTTTCTCAACCAGGAGCTGACGCCGCCGGCGGCGTTTGTGCCGGCCGCCAGCACCGACTGCATGCAGATTGCGATCACCGATTTCACCGCGTTCTGCGTCTTTCCGACGCTGATGCATCACCTGCAGCGTGAGGCGCCCGGGCTGCGGTTTGAGCTCCGTTATCTGCCCCACAGCCCGGCGCTGACCGAGCTGCTGGCGGGCGAGGTCGACCTGGCCCTCGGGTTCAATACCCCTGACGAGCCAGCCCATCCCGATCTTGAGGAGATTAACTGGCTGCGGGACGAATATGTGGTCATCAGCCAGGCCAACCGGACGGCGCTGACCCTGGACGCCTACCTTGCGGCCCGTCATCTGGTGGTGACCCCGTGGAATGAGCGGCAGGGGGTGCTGGACTGTGAGCTGGAGCGACAGGGCTATTCGCGGCAGGTGGCGATGAAAACCCCCTCGATGCTGAGCGCGCCCTTTATTATTGAGCAGAGCGACCTGTTGATGGCGCTGCCCCGGCGGGCGGCGGAGACGATGGCCCGCGCCGCCCGGCTGACCATTTTCCCGCTGCCGTTTCCGGTTCCGCCGTTTGACGTCAAGATTTACGCCCACCAACGCAGCGGGAAACGCGAGGCTACCCGCTGGCTTATCTCCCTGTTGCAGACGCTGGTGGCGGGGTCCACCGCTTCCTGA

>IR1230_00221

ATGAGCATCACAATCCGCCAGGCCACCCCGGACGACGCCACCGCTATCTACGACATGATTTATGAACTCGCGGTCTACGAGAAAGCCCCCCAAGAGGTAGTGACGACGCCAGAGGAGATCAGAGAGACGCTGTTTGCCAGCGGCAGCAAAACCGAAGCGCTGATCTGCGAGGTGGCGGGCAAAGCGGTGGGCTACGCGGTGTTCTTCACCAGCTACTCCACCTGGCTTGGACGCAATGGTATCTATATGGAGGATCTGTACGTCACCCCCGACTATCGCGGGATCGGCGCCGGAAAAGCGCTGCTGAAAACCATCGCGCAATATGCTGTACAACGTCAGTGCGGGCGTCTGGAGTGGAGCGTGCTCGACTGGAACCAGCCGGCGATTGATTTCTATCTGAGCATTGGCGCGCAGCCGCAGGATGAGTGGGTGCGTTATCGCCTCACTGGCGATGCGCTGCGGGCCTTTGCGGAGTAA

>IR1230_00222

ATGTCACAACAAATTGAATCAGTAAAAATGGCTCTGCAGGAGCTGGGGATTAATGCAAGCGGCGAATTCTTTTACAACCCTGATTACGATTTGCTTATTGCACATGAAACCTCTCCTGAGCTGACCGGCGCGGCCCGCGGCGTGATGACCGACTCTGGCGCTGTGGCTGTTGATACCGGGATCTTCACCGGCCGTTCGCCGCGCGATAAATACATCGTCCGCGACGAACAGACTCGCGACACCGTCTGGTGGGCAGACTCCGGCCTCGGCCGTAATGACAATAAGCCGTTATCGCCAGACGTGTGGCGCAGCCTGAAAAGCCTGGTCGCGGGCCAGCTGAGCGGCAAAAAACTGTACGTCATCGACGCCTGGTGCGGCGCCAGCCCGGACACCCGTCTTGGCGTACGCTTCGTCACCGAGGTGGCGTGGCAGGCCCACTTTGTCAAAAACATGTTTATCGTGCCAAGCGCCGACGAGCTGGCCAGCTTTACGCCAGACTTCGTGGTGCTCAACGGCGCGGGCTGCACCAATGCCAACTGGCAGGCGCAGGGGATGAACTCCGAGAACTTTGTCGCCTTTAATCTCAGCGAGCGTATTCAGCTCATCGGCGGTACCTGGTACGGCGGCGAAATGAAGAAAGGGCTGTTCTCGGTCATGAACTACCTGCTGCCGCAGAAAGGCATCGCGTCGATGCACTGCTCGGCCAACCGTGGCGAAGCAGGGGACGTGGCGCTGTTCTTCGGCCTCTCCGGGACCGGGAAAACCACCCTGTCGACCGATCCGCATCGTCAGCTGATTGGCGATGACGAGCACGGTTGGGACGATGACGGCGTGTTTAACTTCGAAGGCGGCTGCTACGCTAAGACCATCAATCTCGATCCGCAGGCGGAGCCGGAGATTTATGGCGCCATCCGCCGCAATGCGCTGCTGGAAAACGTGGTGGTGCGCGCTGACGGCAGCGTGGATTATGCCGACGGCAGCAAAACCGAAAACACCCGCGTCTCCTATCCGCTGTCGCATATCGACAATATCGTCAAGCCGGTGTCCCGCGCGGGCCATCCGTCGAAGGTGATTTTCCTTGCCGCCGATGCGTTTGGCGTTCTGCCGCCGGTCTCCCGTCTGACCACCGAGCAGATGCAGTACCATTTCCTGTCGGGTTTCACCTCGAAGCTGGCGGGCACCGAGCGCGGCATTACCCAGCCGACGCCGACCTTCTCCGCCTGCTATGGCGCGGCGTTCCTGCTGCTTCACCCGACCCAGTACGCCAGCGTCCTGGCGGCGAAGATGGCGGAATCCGGTGCCGAAGCCTGGCTGGTCAACACCGGCTGGAACGGCGAGGGCAAACGCCTGTCGCTGCGCGATACCCGCAGCATCATTAGCGCGATCCTCAACGGCACTACCGGCCCGCTGCGCGAGGAGACCATCCCGGTGTTCGGTCTTGCGATCCCGCAGAGCATTCCAGGGGTAGACAGCGCGGTGCTGGATCCGCGTAATGGCTGGAGTTCAGCGGATAAATGGCAGGAGAAGGCCGAGAGCCTGGCTCAGCTGTTTATGGATAACTTCAAGCAGTACAGCGATACCGAAGCGGGCGCGCGTCTGGCGCTGGCCGGTCCGCAGTTGCAGAAGAGCGCGGTCGAAGCCTGA

>IR1230_00223

GTGTTGGCAAATCTGGAAGTAAAATGGCTGTATGATGTGATTGCCCTTGAGGAGTCGCGCAGCTTTACCCTGGCGGCCAAAGCCCGCAACATCTCTCAGTCTTCGTTCAGTCGACGCATCCAGTCGCTGGAGGCTTCCCTCGGATTCAGCATCTTCGATCGTAGCGCCAACCCTCTGCAGCTGACCAACCGCGGGAAAATTTTTGTCGGCTATGCGCGAAATATGCTCGATGACATGGATTTTCAGATCAGCCGTATTAAAGGCTTAAATAACACTACCCAGAAAATAAGAATCGACGCCGCCCCTTCGCTGTCGGTGTTATTACTGCCGGAGATCATCGCCGGTTACACCGACCGTAAAAATAAGACTTTTCATGTTGAATCGATTAACGTCAACGACGCCGTATTTAACCTGAAAGAGGGAAAAAGCGATTTTATCCTCTCCTTCTATAATGAAGAATTAATGAACTATCCGTTTATTAATCATAAAATTTTTGATTCTTACCTGCACCTGGTCTCCCCCTGCGACGAACATGGCCGCCCGCTGTTTCATTTACATCGCGGCGTGCTGCCGCTGATGAAATACGCCAACGACAGCTATATGGGGCGCCAGGTGAACCAGGTGATCGACCGCACGCCGGAGATCACCTTCTCTCTCACCTTTGTTTCTTCCATGAGCGAGCTGCTCAAGCGCATGATCCTCAATGGCGACGGCGTGGGCTGGCTGCCGCAATACTCTATTCAGCGCGAGCTGGACGAAGGCCGGCTGACGATCCTCGATGAGAGCCTGTCGCTGCCGATCGGCGCCTGGCTCTACCGCTCCGGCTCGCGGCTTAACCAGGCCGCCGAACGTTTCTGGCAGCACATTAAAACCCGTAACGAACCGCGGGAATAA

>IR1230_00224

ATGAGCCAGCCTTTGCTTGCCTTTAATGAGGTGGATGTCTTCTACGGGCCGATCCAGGCCCTGAAGCAGGTCTCCCTCACCGTCAACGAGGGGGAGACGGTGGCGCTGATCGGCGCCAACGGCGCCGGTAAATCAACGCTGCTGATGTCGGTCTTCGGCCAGCCGCGCATTGCCGGCGGGGAGATTTTCTATCGCGGGGAGGCGATCAGCCGGAAGTCGACCCACTTTATCGCCAGCAACGGCATCGCCCAGGCCCCGGAAGGGCGGCGCATCTTTCCCGATATGACCGTCGAGGAAAATCTGCTAATGGGGACCATTGCCATTGGCAACCGCTATCAGGCCGAGGACAAGGCGCGAATGTACCAGCTGTTCCCCCGGCTGGAAGAGCGGCGGGGACAGCGGGCGATGACCCTCTCCGGCGGCGAGCAGCAGATGCTGGCCATCGCCCGGGCGCTGATGAGCCGACCGCGCCTGCTGCTGCTGGATGAGCCGAGCCTGGGGCTGGCGCCGCTGGTGGTGAAGCAGATTTTTCAGATCCTGCGCGAGCTGACGGCTCAGGGGATGACGCTGTTTCTGGTGGAGCAGAATGCCCGCCATGCCCTCAACCTGTCGGACCGCGCCTACGTGATGGTGAACGGCCAGATCCGCCTGAGCGGTAGCGGCCAGACGCTGCTCAACGACCCGGAGGTGCGTAAGGCATATCTGGGGATCAGCGGCTGA

>IR1230_00225

ATGACCGACAGCATCTTACGCGTTGAGCATCTGATGATGCATTTCGGCGGCATCAAAGCGCTGAATGATGTCAATCTGGAGGTGGAGCGGGGGTCGATCACCGCCCTGATCGGCCCCAACGGCGCGGGCAAGACCACGGTGTTTAACTGCCTGACCGGTTTTTATCGGGCGACGGGGGGCAGTATCGTGCTGCGGGCGCGGGAAAAGGTCACCGATGTTATTCAGGTGCTGGGGCAGAAGCTTCACCCGGACGATTTTCTCCACCCGGCGCAGCTGGGGAGGCGGATCTATTACAAGATGTTCGGTGGCACCCATCTGGTGAACCGCGCTGGCCTGGCGCGCACCTTCCAGAATATACGTTTATTCCGCGAGATGTCGGTGATCGAGAACCTGCTGGTGGCCCAGCATACCCAGGTCAACCGCCATCTGCTGGCCGGGATCCTCAATACCCGTGGGTATCGGCAGGCGGAGAGTCAGGCGCTGGATCGCGCTTTTTACTGGCTGGAGGTGGTGGAGATGGTGGACTGCGCCAACCGTCTGGCGGGGACCCTCTCCTACGGACAGCAGCGGCGGCTGGAGATCGCCCGCGCCATGTGCACCCGTCCGGAGATGATCTGTCTGGACGAGCCGGCCGCCGGCCTCAACCCGGTGGAAACCCAGGCGCTGAGCCGGATTATTCGCTTTCTGCGCCAGCAGCACGGCATTACGGTGCTACTGATCGAGCATGATATGGGGATGGTGATGGAGATTTCGGATCATATCATCGTCCTCGATCACGGGGACGTCATCGCCCGCGGCGCGCCGCAGGCGATCCAGGCCAACGCCAGTGTGATTGCCGCCTATCTTGGCGCTGAGGAAGAGGAGACGCGCCGATGA

>IR1230_00226

ATGGGTGAAATTACGCGCCAGCCTGCGCGGGCTATCCAAAACGCGCTGATCGACAGCGTACTGGCCGGCCTTTGCGCGTTAATTGTCTTTGGCCCGATCGTCGGCGTGGTGCTCAAAGGGTATGGTTTTACCCTCGCGCCTGCGCGGGTGGCGATTCTGGTGGCGGTGGTGATGGCCGGCAGGCTGGCGCTCAGTCTGCTGCTGCAGAGCCACAGGGGAAAAGCCTTTATTGCCCGCTTTGAGGGGGCGGATGACGGGGTGTACGTCCGGCCGCCGGGCTACCGCTCGCGGCTGCGCTGGATAATACCGCTGCTGGTGGGGCTGGCCATTGTCTTTCCGTTTCTCGCCACAAAATATCTGCTGACCGTGGCCATTCTTGGGCTGATCTACGTTCTGCTTGGCCTGGGGTTGAATATTGTGGTGGGCCTCGCGGGCCTGCTCGATCTGGGCTATGTGGCGTTTTACGCCATCGGCGCCTACGGCCTGGCGCTGGGCTACCAGTACCTGGGACTTGGCTTCTGGGCGATGCTGCCGCTGGGGGCGGTGATGGCGGCTCTCGCCGGGGCGTTGCTGGGCTTTCCGGTGCTGCGTATGCATGGCGACTATCTGGCCATCGTCACCCTCGGCTTTGGCGAAATTATCCGCCTGGTGCTTAACAACTGGGTGAGCTTCACCGGCGGCCCTAACGGCGTACCGGTGCCATCCCCCACGCTGTTTGGCCTCGAGTTTACCCGCCGGGCCAAGGACGGCGGCATCCCCATCCATGAGTTTTTCCACGTCAGCTACAACCCCAACCTGAAATTCATCTTTCTCTACGCCGTGCTGTGCCTGGTGGTGATGCTGGTGCTGCTGGTGAAGCATCGCCTGACGCGGATGCCCATCGGCCGCGCCTGGGAGGCGCTGCGAGAGGATGAGATCGCCTGTCGGGCGATGGGGCTCAATCACGTGCTGGTGAAGCTCTCGGCGTTTATGCTCGGCGCGTCGACGGCCGGGATCGCCGGGGTGTTCTTCGCCAGCTATCAGGGGTTTGTTAACCCGACGTCGTTCACCTTTTTTGAGTCGGCGCTGATCCTGGCGATTGTGGTGCTCGGGGGAATGGGCTCCACCCTGGGAGTGGTACTGGCGGCGTTTGTTCTGACGGTCACCCCGGAACTGCTGCGCGGTTTTGACGAATATCGGGTGCTGCTGTTCGGCGTGCTGATGGTGATGATGATGATCTGGCGGCCGCGCGGCCTGGTGCGCACCAGCCGCAGCGGGGTTGCGTTGCGAAAAGGAGTCGCGCCATGA

>IR1230_00227

ATGACGGCGTTTTTTCTGCAACAACTGATCAATGGGCTGACGCTGGGCGCAGTGTACGGCTTAATCGCCATCGGCTACACCATGGTGTATGGGATCATCGGCATGATCAACTTCGCCCACGGCGAGGTGTATATGGTCTCCGCCTACCTGTGCGCCATCGGCCTGGCGCTGCTCTCTTTCTTCGGGATCCACTCCTTTCCGCTGCTGATTTTCGCCACCCTGGTGTTCACCATCGTGGTCACCGGCGTATACGGCTGGGCGATCGAGCGCATCGCCTACCGGCCGCTGCGCAACTCGACGCGGCTGGCGCCGCTGATCTCGGCTATCGGGATGTCGCTGATCCTGCAGAACTATGTCCAGCTGAGCCAGGGGCCGAACCAGCAGGGGATCCCGACGCTGCTTAGCGGCGCGTTGCGCATGACGGTGGGCGATGGGGTGGTGCAGATCACCTGGACCAAGGTGTTTATTCTCGTCGCGGCGCTGGTGGGGATGCTTATCCTGACCTGGATTATTCAGTATACCCGGCTGGGGCGCATCTGCCGCGCCACCCAGCAGGACCGGCGCATGGCGGCGATCCTTGGCATCAATACCGACCGGGTGATCTCCCTGGTGTTTGTCATCGGCGCGGCGATGGCCGGTCTGGCCGGCGTGCTGGTGACCATGAACTACGGCACCTTCGACTTCTATATCGGATTTATTATCGGCATCAAAGCCTTCACCGCCGCGGTGCTGGGCGGGATCGGCTCGCTGCCTGGCGCGATGCTGGGTGGTCTGCTGCTTGGGGTGGCCGAAGCCCAGTTCGCCGGGCTGGTGAATTCGGATTACAAAGATGTCTTTTCCTTTGCGCTGCTGGTGGCGATCCTGATTTTTCGCCCGCAGGGGCTGCTGGGACGTCCGCTGGTGGCCAAGGTCTAA

>IR1230_00228

ATGAGCATGGCCTTTTACGCCCAGGCAGACGTGAAGATCGGCGTCGCCGGGCCCTTTACCGGCCCCAACGCCACCTACGGCGCCCAGTACTGGAAGGGGGCCTCGCAGGCGGTGGCCGATATCAATGCCGCCGGCGGCATCAAAGGCGAAAAGATTGTGCTGGTGCAGGGGGATGACGCGTGCGAACCGAAGCAGGCGGTGGCGGTGGCCAACCGGCTGGTGGATGAAGCGAAAGTCTCCGCCGTGGTGGGGCACTTTTGCTCTTCCTCAACGATGCCGGCCTCCGAGGTATATGACGAAGCCGGGATCCTGACCATTACCCCCGGCTCGACCAACCCGCAGATCACCGAGCGCGGCATGAAAGATCTCTTCCGCATGTGTGGTCGTGACGATCAGCAGGGGGCGATCGCCGCCAACTATATGCTGGACGTGCTGAAGGCGAAGAAAATCGCCGTCATCCACGATAAAGACACCTATGGTCAGGGGCTGGCCGATGCCACCCGCGCGGCGCTGGCGAAGCGGGGTACCAAAGAAGTGTTGTACGAAGGCCTTTCCCGCGGTGAAAAAGACTTTAACGCCCTGGTGACCAAGATCGGCGCCCTCAAGCCGGACGTGGTCTACTTTGGCGGCTGCCATCCGGAAGCGGGCCCGCTGGTGCGCCAGATGCGCGAGCAGGGCGTGCAGGCCAAATTCTTCTCCGGCGACTGTATCGTCACCGAGGAGCTGGTCACGGCCGCCGGTGGGCCGCAGTTCACCAACGGCGTGCTGATGACCTTTGGTCAGGATCCGCGCACCCTCCCGGACGGCAAAGCGGTGATCGAGAAGTTCCGCGCCAGCGGCTTTGAGCCGGAAGGTTATACCCTCTACGCCTACGCCTCTATTCAGGCTATCGCCGCGGCCTGGAACGCCGTCGGTACCGATAACGCCAAAGCCAGCGACTGGCTGAAGAGCCACGACGTCGAGACGGTGATGGGCAAGAAAGCTTGGGATGGCAAAGGCGACCTCAAGGTGTCCGACTACGTGGTCTATCAGTGGGGCGACAAGGGCAAATATCACCAGCTCTGA

>IR1230_00229

ATGAAAGTAGATATCGATACCCAGGATGTACGATACGCCGACGCCTGGCAGGGCTTTCGCGGTACGGCGTGGCAAACGCAGATCGACGTTCGCGACTTTATTCAGCATAACTACACCCCTTACGAGGGCGATGAGTCTTTCCTCGCTAACGCGACGCCAGCGACCACCGCCCTCTGGGAGCAGGTGATGGCCGGGATCCGCGTCGAGAACGCCACCCATGCGCCGGTGGATTTCGACACCAATGTCGCCACCTCCATCACCGCCCACGCGGCGGGCTATATCAACCAGCCGCTGGAAAAGATCGTCGGCCTGCAAACGGACCAGCCGCTGAAGCGCGCCCTGCATCCTTTTGGCGGCATCAAGATGATCAAAAGCGCCTTCGAGGCCTATGGTCGCGAAATGGATCCTGACTTTGAGTATCAGTTTACGGCGCTGCGTAAGACCCACAATCAGGGCGTTTTTGACGTCTATTCCCCGGATATGCTGCGCTGCCGCAAATCCGGGGTGCTGACCGGGCTACCGGACGGCTATGGCCGCGGACGGATCATCGGCGATTACCGACGCGTGGCGCTGTACGGCATTCGCTACCTGGTCCGCGAACGTGAGCTGCAGTTTGCCGACCTGCAGCCGGCCCTTGAGCGCGGCGAGGCGCTGGAGGCCACCCTGCGCCTGCGCGAAGAGCTGGCCGAACAGCGGCGCGCGCTGCTGCAGATGCAGGAGATGGCGGCCCGCTACGGCTGCGATATCGCCCACCCGGCGCGCACTGCCCGGGAGGCGGTGCAGTGGCTCTACTTCGCCTACCTGGCGGCGGTGAAGTCGCAGAACGGCGGCGCCATGTCGCTGGGGCGCACGGCCACCTTCCTTGACATCTATATCGAACGCGATCTGCGCGCCGGTTTATTGAATGAGGAGCAGGCCCAGGAGCTCATTGATCACTTCATCATGAAGATCCGCATGGTGCGTTTCCTGCGCACCCCGGAATTTGATTCGCTGTTCTCCGGGGATCCCATCTGGGCGACGGAGGTGCTGGGCGGCATGGGGCTGGATGGTCGCACCCTGGTCAGCAAAACCACCTTCCGCTATCTGCATACTCTGCACACCATGGGCCCGGCGCCGGAGCCGAACCTGACCGTGCTCTGGTCGCAAGCGCTGCCGGCGGCGTTTAAAAAATATGCCGCCCGGGTCTCTATCGCCACCTCGTCGCTGCAGTATGAGAACGACGATCTGATGCGCAGCGATTTTCACAGCGACGACTACGCCATCGCCTGCTGCGTCAGCCCGATGGTGATCGGCAAGCAGATGCAGTTCTTCGGCGCCCGCGCCAATCTCGCCAAAACGCTGCTCTACGCGATTAACGGCGGGGTGGATGAGAAACTGAAGATTCAGGTGGGGCCGAAAACCGCACCGTTGCGCGATGAGGTGCTGGACTACGGCACCGTGATGGCCAGCCTCGACCACTTTATGGACTGGCTGGCGGTGCAGTACATCAGCGCGCTGAACATCATCCATTGCATGCATGATAAGTACAGCTACGAGGCGGCGCTGATGGCCCTGCACGACCGCGACGTCTACCGCACGATGGCCTGCGGCATCGCCGGCCTGTCGGTAGCGGCGGATTCGTTGTCGGCGATAAAGTACGCCCGGGTGAAGCCGGTGCGCGATCACCACGGGCTGGCGGTGGACTTTGTCATTGAAGGCGACTATCCGCAGTACGGCAACAACGACGATCGGGTCGATGCCATTGCCTGCGACCTGGTGGAGCGCTTTATGCGCAAAATTCAGGCGCTCCCCACCTGGCGTCAGGCGGTGCCGACCCAGTCGATCCTGACCATCACCTCCAACGTGGTATACGGCCAGAAAACCGGCAACACCCCGGACGGACGTCGGGCGGGGACGCCGTTCGCTCCGGGGGCCAATCCGATGCATGGTCGCGACCGGAAAGGGGCGGTGGCGTCGCTGACCTCGGTGGCGAAACTGCCGTTTACCTACGCCAAAGATGGCATCTCGTATACCTTCTCCATCGTGCCGGCGGCGCTCGGCAAAGCCCCGTCAGCGCAGGAAAACAACCTGGTGGGCCTGCTGGACGGCTACTTCCACCACGAGGAGACGGTGGAGGGGGGACAGCATCTCAACGTCAATGTGCTGAACCGGGAAAAGCTGCTCGATGCGATAGAACACCCGGAGCAGTATCCGAATCTGACGATCCGCGTCTCCGGCTATGCGGTCCGCTTTAACGCGCTGACCCGCGAACAGCAGCAGGATGTGATCTCCCGAACGTTCACCAGCCAACTCTGA

>IR1230_00230

ATGACTGAATTTCCGGTAGTACTGGTGATTAACTGCGGCTCGTCGTCAATCAAATTTTCCGTCCTGGACGCCGCCAGCTGCGACTGCCTGCTCAACGGCGTGGCCGAGGGCATCAACGCGGAACGGGCGTCTCTCTCGCTCAACGGCGGCGAGCCGGTGGCGCTGGCGCAGCGCGGCTACGAAGGCGCCCTGCAGGCGATTGCCGGGGCATTAGCCCAACGCGACCTCATCGACAGCGTGGCCCTGATTGGCCACCGCGTCGCCCACGGCGGCGACCTGTTCACCGAGTCGGTCATTATTAGCGAGGAGGTTATCAATAACATTCGCCAGGTATCGTCCCTGGCCCCGCTGCATAACTACGCCAGTCTCAGCGGCATCGCCTCGGCGCAGCGGCTGTTCCCGGAGGTGATGCAGGTGGCGGTCTTTGATACCAGCTTTCACCAGACCCTGGCCCCGGAAGCTTTTCTCTATGGTCTGCCGTGGGAGTATTACCAGAATCTCGGGGTACGCCGCTACGGTTTCCACGGCACCTCGCACCGCTATGTGTCCCAGCGCGCCCTGGCGCTGCTCGGGTTGCCGGAGCAGGAGAGCGGCCTGGTGATCGCCCACCTGGGCAATGGCGCCTCGATCTGCGCGGTGCGTAACGGCCGCAGCGTGGACACCTCGATGGGGATGACGCCGCTGGAGGGGCTGATGATGGGCACCCGCAGCGGTGATGTCGACTTCGGCGCCATGGCGTGGATTGCCGGGGAAACCCGGCAGACCCTCAGCGACCTGGAGCGGGTAGCCAACACCGCCTCCGGCCTGCTGGGGATCTCCGGTCTTTCCTCCGACCTGCGGGTGCTGGAGCAGGCGTGGCATGAGGGCCATGCCCGCGCCCGCCTGGCGATTAAAACCTTTGTCCATCGTATCGCCCGCCATATTGCCGGCCACGCCGCGGCGCTGCAGCGTCTCGACGGCATTATTTTCACCGGCGGGATTGGCGAGAACTCGGTGCTGATCCGTCGGCTGGTGAGCGAGCGGCTGGCGGTCTTTGGCCTTGCGATGGACGCCGCCCGCAATCAGCAGCCCAATTCGGCCGGCGAGCGCCTGATCTCCGCCGACGGCAGCCGGGTGCGCTGCGCGGTTATTCCTACCAATGAAGAGCGCATGATTGCCCTCGATGCGATCCGCTTAGGCCGGATCCACACCGCGGCGGCACTGGCCTGA

>IR1230_00231

ATGAGTACGACTGAGAGCATTGCATCCAGCCAGACAAGCCTTTCGTCCTGGCGTAAATCTGACACCACCTGGACCCTGGGCCTGTTTGGCACCGCTATCGGCGCCGGGGTGCTGTTTTTCCCGATCCGCGCCGGCTTCGGCGGGCTGATCCCGATCCTGGTGATGCTGGTGCTGGCCTATCCCATCGCCTTTTATTGCCATCGGGCGCTGGCCCGGCTATGCCTGTCGGGGGCTAACCCCTCCGGCAATATCACCGAAACGGTGGAAGAGCATTTTGGCAAAACCGGCGGGGTGGTGATCACCTTTCTGTACTTCTTCGCCATCTGCCCACTGCTGTGGATTTATGGCGTCACAATCACCAATACCTTTATGACTTTCTGGGAGAACCAGCTGCAGATGCCGGCTCTCAACCGCGGGGTGGTGGCGCTGCTCCTGCTGCTGCTGATGGCGTTTGTCATCTGGTTTGGTAAAGACCTGATGGTCAAGGTGATGAGCTATCTGGTGTGGCCGTTTATCGCCAGCCTGGTGGTGATCTCCCTGTCCCTGATCCCCTACTGGAATAGCGCGGTGATTGACCAGGTCAACCTCAGCGATATCGCCTTAACCGGTCACGACGGCATTCTGGTGACGGTATGGCTGGGCATCTCCATCATGGTGTTCTCCTTCAACTTCTCGCCGATCGTCTCCTCGTTTGTGGTCTCCAAACGCGAAGAGTATGAAGCGCAGTTTGGCCGCGAGTATACCGAACGCAAATGCTCACAGATCATCTCCCGGGCCAGCATGCTGATGGTGGCGGTGGTGATGTTCTTCGCCTTCAGCTGCCTGTTTACCCTCTCACCGCAGAACATGGCGGACGCCAAGGCGCAGAATATCCCGGTGCTCTCCTATCTGGCGAACCACTTTGCCTCGATGTCGGGCACCAAATCCACCTTTGCCACCCTGCTGGAGTATGGCGCGTCGATCATCGCCCTGGTGGCCATCTTCAAATCGTTTTTCGGCCACTATCTCGGCACGCTCGAAGGGCTGAACGGTCTGATCCTGCGCTTTGGCTATAAAGGGGATAAAACCCGGGTCTCCAGCGGCAAGCTCAATACCCTGAGCATGGTGTTCATCATGGGCTCGACCTGGGTGGTGGCGTATGCCAACCCCAATATCCTCGACCTGATTGAGGCCATGGGCGCGCCGATTATCGCCTCGCTGCTGTGCCTGCTGCCGATGTACGCCATTCGTAAAGCGCCTTCGCTGGCCAAATACCGTGGCCGTCTCGACAACCTCTTTGTGACCGCTATCGGTCTGCTGACCATCCTCAACATTGCTTACAAACTGTTCTAA

>IR1230_00232

ATGCATATTACCTACGATCTTCCGGTATCTATTGACGATATTCTCGAAGCGAAACAACGCCTCGCGGGAAAAATATATAAAACAGGTATGCCCCGTTCGAATTATTTTAGCGAGCGCTGCCAGGGCGAAATATTTCTTAAATTCGAAAATATGCAGCGCACGGGTTCATTTAAAATTCGCGGCGCCTTTAATAAACTGTGCGGTTTAACCGCGGCGGAAAAACGCAAAGGGGTGGTGGCCTGCTCAGCGGGCAACCACGCCCAGGGCGTCTCGCTCTCCTGCGCCATGCTCGGCATTGACGGGAAAGTGGTGATGCCGAAAGGGGCGCCGAAATCGAAAGTTGCCGCCACCTGTGATTATTCGGCGGAAGTGGTGCTACATGGCGATAATTTTAACGATACCCTCGCCAAAGCCAGCGATATTGTTGAACTTGAGGGCCGCATTTTTATTCCCCCCTATGACGACCCGCAGGTTATCGCCGGGCAGGGGACGATTGGACTCGAAATATTAGAAGATCTGTATGACGTGGATAATGTCATCGTGCCCATTGGCGGCGGCGGCTTAATTGCCGGTATCGCGATTGCGATTAAATCCATTAACCCGACAATCCGCATTATCGGCGTGCAGTCGGAGAATGTTCACGGCATGGCCGCCTCCTGGTATGCCGGGGAAATCACCAGCCATCGCCACGCCGGCACCTTAGCCGATGGTTGCGACGTCGCCCGCCCGGGGAAACTGACCTATGAAATCGCCCGCCAGCTGGTGGATGACATCGTCCTGGTGAGTGAGGATGACATTCGCCAGAGCATGGTCGCCTTAATTCAGCGCAATAAAGTGATCACCGAAGGGGCCGGGGCGTTAGCCTGCGCCGCGTTATTAAGCGGCAAACTCGACAGCTATATCCAGAACCGCAAAACGGTCAGCCTGATTTCCGGGGGCAATATCGATCTCTCCCGGGTATCGCAAATTACCGGTTTTGTTGACGCTTAA

>IR1230_00233

ATGAATACTTTTTCTCTGCCTAAAACACAGCATCTGGTAGTTTTCCAGGAAGTGATCCGCAGCGGGTCAATCGGGGCCGGAGCAAAAGCGTTAGGACTCACTCAGCCGGCGGTCAGTAAAATTATTGGCGACATGGAGAGTTATTTTGGCAGCGAATTAATCGTCCGGCGAAATACCGGCGTCTCGCTCACCGAGGCCGGGCAGGTATTTCTCAACTGGTCAGAAGCGATCACCCGTGAAATGAAAAATATGGTCAATGAAATGAATGCTCTGACCAACAGCGCGGTGGTGGATGTCTCCTTTGGTTTCCCTTCGCTGGTCGGGTTTACCTTTATGTCGGAGATGGTGCATCAGTTTAAAATGACTTTCCCCAAGGCGCGGGTCTCGATGTATGAGGCGCAGCTCTCCGCCTTTTTGCCGGCAATCCGCGACGGGCGCCTCGACTTCGCCATCGGGACGCTGAGCGATGAGATGAAACTGCAGGACCTGCACGTCGAGCCGCTGTTTGAGTCTGAGTTTGTGCTGGTGGCGAACCGGGCGCGGATCGGCAACGGCACCGTCCGCCTGGCCTCGCTCCAGCACGAGCAGTGGGTGCTGCCGGAGACCAATATGGGCTACTACAGCGAACTGCTGACCACCCTGCAGCGCAGCGGTATCCGCCGTGAAAATATCGTTAATACCGATTCGGTGGTGACGATCTATAACCTGGTGCTCAATGCCGATTTTCTGACGGTGATCCCCTGCGACATGACCACGCCGTTTGGCTCGAGCCAGTTTGTCACCATCCCCATTAAAGAGGCCTTGCCGGTGGCGCGCTATGCGGCGGTATGGTCGAAAAATTACCGGTTAAAAACCGCCGCGGCGTCGCTGGTGGAGATGGCGAAACACTATTCCTCGGGGCAGGGCAACCGCCACTGGCAGCCATTACAAATTATTGCGTAA

>IR1230_00234

ATGATCCCGCGGCTGCGCCAGCAGCTTAACGAGCAGCTGATGAGCGACCAGCTGGCTGTCGACCTGCTCTCTTCCTATCTGCGGATGGATCTTAGCGCCCAGCCGTGGGGCCAGCCGCTAAAGCCCGCCCCGGCGCAGATCGCTGGCCCGGGCATTTCGCCTATAGCTGGCTGA

>IR1230_00235

ATGGCTGAACGCATGACCTTTCCGATGTATGCCATCCACCGTCAACAGACCCAGGCCCTGTGGCAGACGGTGCAGTCGCTGCTGGACGAACGCGGCGTGATGGTGGCGGGGGACCCTCCGGCGGCTGACCCCGGGGATCTGCTGGCTCACTGGCGCCAGCCGACGCTGTTGCTGAGCCAGACCTGTGGCTACCCGCTGGTGACCCAATTGCCGGAGGTGCAGACGGTGGGCTGCTTCCACTACGCTGCGCCCGGTTGCGAAGGGCGCCGCTATCGCAGCCTGCTGGTGGTCCGCGAAGCGGATAGTCACCGGATGCTGGGGGACTTTTTTGGCCGTCGGGCGGTGTGCAATGCCGAGCACTCGCAGTCCGGCTACAACGTACTGCGTAAAATGGTCGCGCCGCTCTCCCGGGAGGGGCGTTTTTTTTCAGCGGTGATGTTCAGCGGCAGCCATCGGCAATCGCTGCGCGAGCTGCAGCAGGAAAACGCCGATATCGCCGCCATCGACTGCGTGACCTACGCTCTGCTGCAGCGCCATCAGCCGCAGGCTCTGGCTGGCCTGGTGGCGATCGGCTGGAGCCCGGCCGCGCCAGGGCTGCCGCTGATCACCGCCGGCGCCACCCCGGCGGCAACGCTGAATAGCCTGCGCGAGGCGCTCCAGCAGCTGGTAAGCGATGATCGCTATCGTTCCCTGTGCGACGCCTTGCTGATCTGCGGCTATAGCGATATGTCGCGGGAGGCCTATGCGCCGCTGCTGGCGTGGCGGGATGAAGCCGCGGCGCTGGGGGTCAGCCAGCTATAG

>IR1230_00236

ATGAGGCAACACGGCAAGGTATCGGAAGTGACGGAAAAGAAGACGGCTGTCTATGTGGATGACCAACAGCGCATCCTGATCCGCCAGCTGGCGCGCAGCTGGCTGTGGCGCAGCGAGCTGCCGACCTGGCTGCTGATTGTGACGGTTTACGGCGGCTGGTTTGCCTGCGTGGCGGGCTGGCGGACGCTGGGACTGTTTCCGGCCACGCTGCTGTTGATCTGGTTTACCGCCTGGTATATGTCCTTACAGCATGAGCTGATCCATGGGCATCCCACCCGGCTGGCATGGTTTAACCAGCTGTTGGGGACGCTGCCGCTGGCGGTCTGGTATCCCTATGGCGTCTACCGCGACAGCCACCTGGCGCACCATCGCAACCATCTGCTGACGCACCCTGAGGATGACCCCGAATCGTACTACGTAACGGCGGAAAGCTGGCAGCGCTTCTCAGCCTGGCAGCGCCGCCTTATCCACCTGCGCAACACCTTCTGGGGGCGTCTGCTGCTGGCGCCGATGATGGATATTATTCACACCCTGAACAGCGCGCTGCGGGCTTTCCGCGAGGGCGACCGCCGCGCTATCGCCATGTGGAGTCTGCATCTGTTGCTGCTGACGGGCCTGCTGACCTGGATGGCGGCGCAGGGTTTTTCTCCGCTGTGGTTTGTGCTGGCGGTGAGCTACCCGGCGCTGGCCCTGACCAAAGTACGCTCTTTTCTCGAACACCGCGCCGCGGATGACCCGCTGGCCCGCTCGGTGATCAACGAGGCCGGGCTCCCCTGGCGGGCGCTGTTCCTCAATCTCAACTACCATGCGGTGCATCATGATCTGCCAGGGGTCCCCTGGTACGCTTTACGTCAACTGTATCTGCATCGCCAGGCGGCGTATCTGCAGCGTAATCAGGGGTTCCTGGTGCGCTGGTACGGCGAGTGGCGACGCCATTTTAGCCGCCGGGCGGTAGCGGTGAACGCCCATCCGGGCTTTGGCGAGCAGGCGCAGGCGGGAGGAGAACATGGCTGA

>IR1230_00237

ATGACCTACTGTGTGGCCATGTGCCTGGCGGATGGACTGGTGTTTGCTTCCGACTCCCGCACCAATGCGGGCGTCGACCATATTGCCACTTTTAAAAAGCTGCACGTTTTCCATCAGGAGGGGGAACGGGTGCTGGTGCTGCAATCCGCCGGCAATCTGGCCACCACCCAGAGCGTGATAAGCCTGCTCAGCGCGCGCATCCGTACCCAGCAGGAGCCGAATCTGATGCAGGTCACTTCCCTCTACGACGCCGCGATGCTGATCGGTAAAACGCTGCTGGAGGTGATCCAGCGCGACAGCAGCAATCAGCAGACCTGCAGTAACACCAACTTCAACTGCAACCTGTTGCTTGGCGGCCAGATCGCCGGCGAAACCCATCGTCTGTTCCATATTTATCCGGAGGGCAACTTTATTGAAGCCACCCGCGACACGCCTTATTTCCAGATTGGCGAAAGCAAATACGGCAAACCGATCATCGATCGGGTGCTGACCATCGACACGCCGCTGGAGCAGGCGATGTGCTGCGCGTTGATCTCCATTGACTCGACGCTGCGCAGTAATCTGTCCGTCGGCCTGCCGCTGGATACGCTGCTCTACCGCAGCGGCAGCTTCAGCAGTGCCGGGCAGCATCGCATCACCGACAGCGATCCCTACTTCAACCGCATTCGCAAGGCGTGGTCCGAAGGGCTGTTGCACACCTTCCAGACTCTGCCGACCTGGACGCCAGCGGAGCGGGAAGAGGAGTAG

>IR1230_00238

ATGAAACTGGTCATCGATCATCTCACCCGCTATGGCTACGATGAAGAAGTGAAGTTTTCCACCCAGTATCTGCGCCTGACGCCGCGCAGTACCGCCCGGCAGACGATCACCGCCTGGACGCTGACCCTGCCGGACGGCGCGGCGGTGACCACTACCGACGGCTGGGGCAATGTGCTGCATGTGCTGACGCTGGATAACCCGCACAAGGAGATCACCATCCGCGCCAGCGGGATTGTCGATATCGCCGACGAAGGGGAGGAGACGCGTGATGAAGAGGCCGAGCTGCTGTCGCCGCTGGTTTTTCTGCGCTGTACGCCGCTAACCCGCGCCGATACGGCGATCCGTGAGTTTGCCCAGCGCCTCTATCGTCCCGATGCGGCGGAAGAGAGTCTGAATCAACTGATGGCGGATCTGCTGTTAAGAATGCCATACTCCCCCGGCGCGACCCAGGTGCAGGACTCCGCCGCCGACGCCTTCGCCCGCGCGAAGGGGGTGTGCCAGGATCATACCCATGTTTTTCTCGCCTGCTGCCGCGCGCTGGAGATCCCGGCGCGCTACGTCAGCGGCTATGTCTACAGCGATAACGCCCAGCATGTGGCGATGCATGCCTGGGCGGAAGTCTGGCTCGACGGCCGCTGGCTGTCGTTTGATATCACCAATAATACCCGTCGCCTGAATCAGCATTTACGGCTGGCGACCGGGCTGGATTATCTCGACGCCTGCCCGGTACGGGGCACCCGGCTGGGCGGCGGCGGGGAGATTATGTTAACTAACGCTGAAGTGCGCGAACACAGCCAGCAGGCGCAGCAGCAATAA

>IR1230_00239

ATGCTAAGTCGTACGGCGAGTGAACTGTTCTGGATGGCCCGCTATCTCGAGCGGGCGGAGAGCTACGCCCGGGTGCTGGACGTGACCTGGAAATTATCAATGATCCCCCGCCACAGCCAGCAGTCGCGGGATCTGGCGCTACCGCTGAATTTGTCGATGACCCACGAGTTGTTTCAGGCGCGCCATGCCCGCTTTACCATGAGCAATCTGCTCAACTTTTTTGCCCTTGATGGCAACAACCCCTGCAGTATTTACAGCTGCGTGGAGATGGCGTGGAACAACGCGCACGCGGTGCGCGGCAGTCTGTCGGCCGAAGTGTGGGAGAGCATCAATGCCACCCGCATTGAACTGCGGACCCTCCGCCAGCAGGGGCTGGGCGAGCTGGGGAGCGACGGCTTTTTTGAGTGGGTGAAAGAGCGGGTGCATCTGTTTCGCGGGGCAGTGATCGGCACGCTGCTGCGCAACGACGCCCTGAGTTTTATCGGTATCGGCACCCTGATTGAGCGCGCCTTCGCCACCACCCAGCTGCTGCTGATTAAGGATCAACAGCTGACCAACGACCCGGACCCGGTGCGGGAATACTACCGGCTGGATACCCTGCTCAATGCGGTCAGCGCCCGCGAAGCCTACAACAGCCTCTACCGCCAGCCGGTCAGTCGCGAAACGGTGATGGAGCTGCTGATCCTGCGCAACGATATTCCGCGTTCGCTGCGCGCCAGCATTGCCGATCTGGTGGGCGAGCTGGAGAAGATCGCTAACGACCGCTCCTATCAACCGCTGCGCCTCGCCCATCAGCTTAACGTTGATCTGCGCTTCAGCACCCGTGACGACCTGGCGCAGGCGGACCTGCAGACCACCCTTAACGGGCTGCTGGCGAGAATTAACGCGCTGTCCGACAGCATCAGACAAACCTATCTGGAGGCCTTATGA

>IR1230_00240

ATGATTAAAATTACGCTACCTGACGGGCATTATTATGACGAGATGCTGACCGCGCAGGGTGAACAGCGGCCGCACTATAATGCCTGGTGGCAATGGTTTCGCAACACCGATCAATTCTCTATCCGGCAGAAAAAAGCGCAAGCGGAACTGCTTTTTCACCGTATCGGCATCACCTTTAACGTTTACGGCGAGGATGAGGGGACCGAGCGGCTGATCCCGTTTGATAGCGTGCCGCGGATCATACCTGCCGGGGAGTGGCAGCGCATTGACCGCGGCATACGCCAGCGGGTGAAAGCGCTGAACGCCTTCCTCTATGACATCTATCATGAGCAGAATATCCTGCGCGCGGGGCTGATCCCGGCCGAGCAGGTGCTGGCCAACGAGCAGTATCAGCCGTGCATGCAGGGCATCAACCTGCCAAACAACACCTATGCCCATATCACCGGCGTCGATATGGTGCGCAATAACGACGGTCAGTACTACGTGCTGGAGGATAATCTGCGCACGCCGTCCGGCGTCTCCTACATGCTCGAAAACCGTAAGATGATGATGCGCCTCTATCCGGAGATGTTCGAGCAGCATCACATTGCGCCGGTGGAGCGCTACCCGAGCTACCTCCTGCAGACCCTGCGCGAAAGCTCGCTGGTGGATGATCCCTGCGTCGTGGTGATGACCCCGGGTCGCTTCAACAGCGCCTACTTCGAGCACAGCTTCCTGGCGCAGCAGATGGGGGTCGAGCTGGTGGAGAGCGCCGATCTGTTTATTAAAAACGGCGCGGTGTATATGCGCACCACCGAGGGGCCGCGGCGGGTGGATGTGATTTATCGCCGCATCGACGACGCCTGGCTCGACCCGCTGGCCTTCCGCGCCGATTCGATGCTCGGCGTCCCGGGGCTGCTGTCCGTCTATCGCGCCGGCGGCGTAGTGCTGGCCAACGCCATCGGCACCGGAGTGGCTGACGACAAATCGATCTATCCGTACGTCCCGGAGATGATCCGCTTTTACCTCGGCGAACAGCCGATCCTCAGCAATATCCCCACCTGGCAGTGCCGGAAAGCGGAGGACCTGCGCTACGTGCTGAGCAATCTCGAGCTGATGGTGGTCAAGGAGGTCCATGGCGCCGGGGGCTACGGCATGCTGGTCGGCCCGCGTTCGACTAAAGAGGAGCGAGAGGCCTTCCGCCAGCGCCTGCTGGCCAATCCGGCCAACTATATCGCCCAGGACACCCTTGCGCTGTCGACCTGCCCCACCTTCGTTGAGGAAGGTCTGTCGCCGCGACATATCGATTTACGGCCCTACGTGCTGTCCGGGCAGGAGATGCGGCTGGTGCCCGGCGGCCTGACCCGCGTGGCCCTCACCGAAGGGTCGCTGGTGGTCAACTCGTCGCAGGGCGGCGGAACCAAGGATACCTGGGTGATGGAGGATGACGCATCATGCTAA

>IR1230_00241

ATGACGAGTACTGATGCACGTCGTCCCGCTGCTTTGCCCTGTTCCCTGCGCCTGGCGATCGGCGGCGCTCTGATCGCCCTGATGAGCCTGAACGCTCAGGCGGAAGACGGCAAAACCGCGCCGCCCCCCTCGCCGGATATTCTGCTGGGCCCGCTGTTCAATGATGTCCAGAGCGCGAAGCTGTTTGCCGATCAGAAAACCTTCGCCGACGCTATCCCTAACAGCGATCCGCTGATGATCCTTGCCGATTACCGGATGCAAAAAAACCAGGCCAGCTTCGACCTGCGCCACTTCGTCGAACTGAACTTCACGCTGCCGAAAGAGAACGACACCTATGTGCCGCCCAAAGGGCAAACTCTGCGTCAACATATCGATGGCCTGTGGCCGGTCCTGACCCGCAGCACCGTCGAGGTGGAAAAATGGGACTCGCTGCTGCCGCTGCCTAAGCCCTACGTGGTGCCCGGAGGACGTTTCCGCGAAGTCTATTACTGGGACAGCTATTTCACCATGCTGGGTCTTGCCGAAAGCGGTCACTGGGATAAAGTCGAGGATATGGTCGCCAACTTCGCCGCGGAAATTGACGCCTGGGGCCATATCCCCAACGGCAACCGCACCTATTATCTCAGCCGTTCGCAGCCCCCCTTCTTCTCCTTTATGGTGAGCCTGCTGGCGACGCACGATGGCGACCAGGTGCTGAAAACCTACCAGCCGCAGCTGGAGAAAGAGTATCGCTACTGGATGGCCGGAGCGGACGCGCTGGCTCCCGGCAGCGCCGACAAACGGGCGGTGCGGATGGCGGACGGCGCGCTGCTCAACCGCTACTGGGACGATAACGACACCCCGCGTCCCGAGTCCTGGCTTGACGACGTCAAAACCGCCAAAAGCAACCCGAATCGTCCGGCAACCGAGATCTATCGCGACCTGCGCTCCGCCGCCGCCTCCGGCTGGGATTTCAGCTCCCGCTGGATGGACAATCCGCAGCAGCTCGCCACCATTCGCACCACCTCGATTGTCCCGGTCGATCTCAATGCCCTGATGTTCCATCTGGAGAAAACCCTCGCCCGCGCCAGCAAGGCATCCGGGGACAGCGCTGGCGCTACGCAGTACGATGCGCTGGCTAACGCCCGCCAGCAGGCCATCGAGAAATACCTGTGGAATGATAAAGAGGGATGGTACGCCGACTACGATCTGAAAACCCACAAGGTGCGCAATCAACTGACCGCGGCGGCGCTGTTCCCGCTGTACGTTAATGCCGCGTCACGCGAGCGGGCGACGAAAGTGGCCGCCGCCGCCGAGTCGCGCCTGCTTAAACCCGGCGGGCTGACCACCACCACCGTCAACAGCGGCCAGCAGTGGGACGCTCCCAACGGCTGGGCGCCGCTGCAGTGGGTGGCGGTCGAGGGGCTGCAAAACTATGGTCAGCAGAAGATCGCCATGGAGGTTACCTGGCGCTTCCTGACCAACGTGCAGCATACCTATGACAGTAAGCAAAAGCTGGTGGAGAAGTATGACGTGAGTTCGACCGGCACCGGCGGCGGCGGCGGGGAATATCCGCTGCAGGACGGCTTTGGCTGGACCAACGGCGTCACCCTGAAGATGTTGGATCTGATCTGCCCGCAGGAGAAACCCTGCGATGCGCTGCCCGCTACCCGTCCGGCGACAACCCCTTCACCGCAGGACAAACCTGTTGCGGCGCCCGCGGCTAACGACCCCGCCCCTGCGGAACCGCAAAAGACCGGCTCCTGA

>IR1230_00242

ATGAACGTGGCTATTTCTCGAAAACGCCCGGGGCTGCTGTATGCCCTTGCGGTCGCACTCCCCTTCACCGCGCAAGCCGAAGAGACGGTGGTGGTCACTGCCACCCCGCCGGCGTCCGCCAGCGCGCCGACGGAGGGCTACAGCGCCAGCACCTCGCTCGGGGCGACGAAAACCGACCAACCGTTAATCACTACCGCCCAGTCGGTGTCGGTGGTCACCCGCCAGCAGATGGCGGATCAGGGGGCGAATACCATCAGCCAGGCGCTGGAATATACCCCGGGGGTCTACTCCAGCTTCGGCGGCGGCGCCACCCGGTTCGACGCCATCTCCCTGCGCGGCTACCACGGCGGCGACGTCGATAACCTGTTCCTCGACGGCATGCGCCTGATGAGCGACGGCGGCAGCCATAACGTATTGCAAATCGACCCGTGGTTTATCGAACGCGTGGATGTGATCCGCGGCCCCTCCTCCGCGCTCTACGGGCAGAGCGTGCCGGGCGGCGTGGTCAACCTGACTTCCAAACGTCCGCAGTTCAGTCAGCAGGGCCACATCCGCCTGACCGGCGGCACGCAAAATACCAAAGGCGCGGCCTTCGATTACACCGACGCCATCAATGACCAGTGGGCATGGCGGCTGATCGGGATGACCCGCAGCAGCGACACGCAGTATGACCATACCCGCGAAGAGCGCTACGCGATTTCGCCTTCCCTGCTGTGGCAGCCGGACAGCGACACCTCGCTGCTGCTGCGCGCCTATCTGCAAAAAGATCCTTCCGGCGGCTACCACGGCTCTTTGCCGCTGGACGGCACCCGCTACGCGCACAATGGCCGTAAGCTCTCCCCCAGCACCAACGAAGGCGATCCGGGAGATGGCTATCAGCGCCGCCAGCAGATCTACAGCTATGAGTTTGACCACCAGTTCACCGACGTCTGGTCGGTCTATTCCGCCGGGAGCTACACCCATACCAACGTCTCCCTCGATCAGGTCTACCAGGTCGGCTGGATAGATGACAGCGACATGCTGGCCCGCGGCTACAGCGGTTCGCGCGGTTCGCTGGACGGCTGGTCAACCGATAACCGCCTGCGCGCCGATTTCAATACAGGCGACCTGGCGCACACCCTGATCCTCGGCGCCGAATATCATCGCTTCCGTAACGACCTGTGGACCGGCGCCGGCGGCGCGGCGCCCCTTAACCCGTTTAGCGGCTATACCGCGCAGACCGGACATACCGTTACCTACAGCGACGACAATAATCGCCGCTATTACCAGACCGGGCTGTATCTGCAGGATGAGATGGTCTGGAACCGCTGGCATGTGGATGTTTCCGCCCGCTACGACCGCATCGTTTCCCAGCAGGTCAGCGATACCCAAGGGACCTCAAACCGCCGTTCAGACGACCATATCAGCGGCCGCGCCTCGCTGTTGTACGCCCTGGACAACGGTCTGTCGCCCTACCTGAGCTACAGCCAGGCGATCACTCCGGCGATGCTGCCGGGCGCGGACGGCAAACCGTTGAAACCGACCACCGCCGAACAGGTTGAAGCCGGCCTGAAGTTCCAGCCGCCGGGCAGCAGCGATCTCTATAGCATCGCGATTTACGACCTGACGCAAAAGGATGTCGCCACTCGCGACCCGAACATCGCCACCGCCACCTATATTCCGGCGGGTAAGGTCCATTCCCAGGGCGTTGAGCTGGAAGCGCACCACCAGATCACCCCGCAGCTGAGTACTATCGCCTCGTATACCTGGAATCGTCTGCGTTTCCAGGACACCAAAGACGGGACCGACAATAACACGCCGCAGCTGACCCCGGATCAGATGGCCTCCTTCTGGGCGCGCTATCAGTTCCCGGCGGGGATCTCCGTTGGCGCCGGCGTCCGCTACATCGGTAAACAGTGGGCGGATGATGCCAACACCGCGCGGCTGCCGTCGGTCACGCTGATGGACGCCATGATGCGGGCCGACCTCAGCGTCTGGTCGCCAACGCTGAAAGGCGCTTATGTGCAGGTTAACGCCAACAATATCGGCGACCGCGAGTATATTTCCGGCTGCTATGGCACCGGCAACTGTTACTGGGGAGCAGAGCGCAGCGTTATAGCCACCGTGGGCTACGATTTCTGA

>IR1230_00243

GTGAAATTGAGATGGTTGCTTATTTTAGTCGTTTTTCTGGCCGGTTGTAGTTCGAAACATGATTACACTAACCCGCCGTGGAACCCCGAGGTCCCGGTGAAGCGGGCGATGCAGTGGATGCCGATCAGTGAAAAAGCCGGCGCCGCCTGGGGCGTGGATCCGCAGCTGATCACGGCGATCATCGCCATCGAGTCGGGTGGTAATCCTGCGGTGGTGAGTAAATCCGGCGCCGTCGGGCTGATGCAGCTCAAGCCGTCGACCTCCGGACGCGATGTCTATCGGCGGATGGGGTGGCGCGGCGAGCCGTCGGTCAGTGAGCTGAAAAACCCGGAACGTAATATCTCGATGGGGGCCGCCTATCTGAGCATTCTGGAGAACGGTCCGCTGGCGGGGATCAAAGACCCGCAGGTGATGCGCTATGCGGTGGTGGTCTCCTATGCCAACGGCGCTGGCGCGCTGCTGCGTACCTTCTCCTCGAACCGCCAGGACGCCATCGAGGAGATTAACGATCTCGACGCCGATGAGTTCTTCGAGCATGTGGTGAAAAAACACCCGGCACCGCAGGCGCCGCGCTATATCTGGAAACTACAGAAAGCGCTGGACGCCATGTAA

>IR1230_00244

ATGTCTCAGTTTTATCTGGTCGCGCCATCCGGGTATTGCATCAACCAGCAGGCGGCAGCCCGCGGCGTCGAGCGTCTGCAGCAGGCCGGACACGAGGTGGCCCACCAGCAGGTCATTCCCCGCCGGCAGCAGCGTTTCGCCGGGACTGAACATGAACGGCTGGCCGATATTAACCAGCTGGCCCAGTTGCCCGGCCGCAACCGGATCGTCCTCGCCGTGCGCGGCGGCTACGGCGCCAGTCGCCTGCTGCCGCATATCGACTGGCAGGGGCTGGTTGCCCGCCAGCAGCGCGACCCGCTGCTGATTTGCGGCCACAGCGATTTCACCGCTATCCAGTCGGGCCTGCTGGCCATGGGCAACGTCATCACCTTCAGCGGGCCAATGCTCGCCGGCAACTTCGGCGCGGAGACCCTCGACCCGTTTACCGAGCACCATTTCTGGCAGGCGCTACGCCAGCCGGAATTCACCCTCGAATGGCCGGGCGAAGGGCCGAACTGCCGGGTGGAAGGCACCCTGTGGGGGGGAAATCTGGCGATGCTGACCTCGCTTATCGGCACGCCGTGGCTGCCGGCGATCCGCGACGGCATCCTGGTGGTGGAGGATATCAACGAGCACCCGTTCCGCGTCGAGCGCATGCTGCTTCAGCTGTTGCACAGCGGCGTGCTGGCGGCGCAGAAAGCGGTGATCTTCGGCAGCTTCACCGGCAGCGCGCCGAACGACTATGACGCGGGATACGATTTGCCGCAGGTCTTTGATTATCTGCGCCAGCAGCTATCCCTGCCGCTGATTAGCGGACTGGAGTTTGGCCACGAGCAGCGCACGGTGACCCTGCCGCTGGGCGCCCGCGCGCGCCTGGTGAATCAGGCGGCGGCCACCACCCTGACGATCGGCGGCCATCCGGTGCTGACAGAATAA

>IR1230_00245

TTGGACGCCGCTGCGGTCATTAGTCTGTTTATTCTGGGTTCTGTTTTAGTAACCTGCAGTATCTTATTGAGCTCCTTTTCTTCGCGCCTTGGCATCCCGATTCTGGTCATCTTTCTGGCCATCGGGATGCTGGCAGGCATTGACGGCATCGGCGGCATCCCCTTCGACAACTACCCCTTCGCCTATATGGTGAGTAACCTGGCGCTCGCGGTGATCCTGCTGGATGGCGGGATGCGCACCCAGGCCAGCTCCTTTCGCGTCGCGCTGTGGCCCGCGCTGTCGCTGGCGACGGTTGGGGTGCTGATCACCTCGGCGCTGACCGGGATGATGGCGGCCTGGTTGTTCAAGCTGGATCTGATCGAAGGCCTGCTGATCGGCGCCATTGTCGGCTCCACCGACGCCGCGGCGGTCTTCTCGCTGCTCGGCGGCAAGGGGCTCAACGAGCGCGTCGGCTCGACGCTGGAGATAGAGTCAGGCAGTAACGACCCGATGGCGGTCTTCCTGACCATCACCCTGATCGAAATGATCCAGCAGCACCAAACCGGCCTCAGCTGGATGTTCGCCGTGCATATCATTCAGCAGTTCGGCCTCGGGATCGCCATCGGCCTCGGCGGCGGCTACCTGCTGCTGCAGATGATTAACCGCATCGTGCTGCCTGCCGGCCTCTATCCGCTACTGGCGCTGAGCGGCGGGATCATGATTTTTGCCGTCACCACCTCCCTCGACGGCAGCGGTATCCTCGCCGTCTATCTCTGCGGCTTTTTGCTGGGCAACCGGCCGATCCGCAACCGGCACGGCATCCTGCAGAACTTCGACGGCCTGGCGTGGCTGGCGCAGATCGCCATGTTCCTGGTGCTGGGCCTGCTGGTGACGCCCTCCGACCTGCTGCCGATCGCCATCCCGGCGCTGCTGCTGTCGATGTGGATGATATTCATTGCCCGGCCCCTGTCGGTGTTTGCCGGCCTACTGCCGTTTCGCGGCTTTAACCTGCGCGAACGGGTGTTTATCAGCTGGGTTGGCCTGCGCGGCGCGGTGCCCATTATTCTCGCGGTGTTCCCGATGATGGCCGGCCTGGACAACGCCCGCCTGTTCTTCAATGTCGCCTTCTTCGTGGTGCTGGTGTCGCTGCTGCTGCAGGGCACTTCGCTGTCGTGGGCGGCGAAAAAAGCCAAAGTGGTGGTGCCGCCCATCAGCTGGCCCATCTCCCGCGTCGGGCTGGATATTCACCCGGAAAACCCGTGGGAGCAGTTTGTCTACCAGCTGGGGGCCGATAAGTGGTGCATCGGCGCGGCGCTGCGCGACTTGCACATGCCACCGGAGACGCGGATTGCCGCGCTGTTTCGCAACAATGTCCTGCTGCACCCCACCGGCAGCACCCGCCTGCGGGAAGGCGATATCCTGTGCGTTATTGGTCGGGAGCACGACCTGCCGGCGCTGGGCAAAATGTTCAGCCAGTCGCCGCCGGTGGCGCTCGATCAGCGCTTTTTCGGCGACTTCATTCTTGATGCTGAGGCCCGCTTCGCCGATGTGGCGCAAATCTACGGTCTCGACGGCGGTGAGGAGTTCCGCGAGCATCAGCAGTCGCTCGGGGAAGTGGTGCAGCAGCTGCTGGGCGCCGCGCCGGTGGTGGGCGACCAGGTGGAGTTCGCCGGGATGGTGTGGACGGTAGCTGAAAAAGAGAACGATCACGTGCTGAAGGTTGGGGTGCGGGTCGCGGAAGACGAGGCGGAGTGA

>IR1230_00246

ATGACCCGTCCGGTAGTGGCCAGCATCGACCTGCTGGCCTTGCGGCAGAATTTACAGATAGTGCGTCGCGCGGCCCCCGGGTCGCGCCTGTGGGCGGTGGTCAAGGCCAACGCCTACGGCCACGGCGTGGCGCGCGTATGGAGTGCGTTAAGCGCGGCGGATGGTTTCGCCTTGCTCAACCTGGAAGAGGCGATCCTGCTGCGCGAGCAGGGCTGGAAAGGCCCGATCCTGCTGCTGGAGGGCTTCTTCCATGCCGATGAGCTGGCGGTGCTGGATCAATATCGTTTAACCACCAGCGTCCACAGCAACTGGCAGATTAAGGCCCTGCAACAGGCGAAGCTGCGCGCGCCGCTGGATATCTATCTCAAGGTGAACAGCGGCATGAACCGGCTGGGCTTTATGCCTGAGCGGGTCCACACCGTCTGGCAGCAGCTGCGGGCGATAAGCAACGTCGGCGAGATGACGCTGATGTCGCACTTCGCGGAGGCGGAGAACCCGCAGGGAATTGTCGAGCCGATGCGCCGTATCGAACAGGCGGCGGAGGGGCTGGATTGCCCGCGCTCGCTGGCCAACTCGGCGGCGACCCTCTGGCATCCGGAAGCGCATTTTGACTGGGTTCGTCCAGGCATCGTGCTGTATGGCGCGTCGCCTTCCGGGCAGTGGCAGGACATCGCCAACACCGGGCTGAAGCCGGTGATGACGCTGCGCAGCGAGATCATCGGCGTGCAGAACCTGCGTCCCGGCGAGGCGATTGGCTATGGCGGCCTGTACCGCACCACCCAGGAGCAGCGGATCGGCATCGTCGCCTGCGGCTATGCCGACGGCTACCCGCGGGTGGCGCCGAGCGGCACGTCGGTGCTGGTGGATGGCGTACGTACCACTACCGTGGGACGCGTATCGATGGATATGCTGGCGGTCGATTTAACGCCTTGTCCGCAGGCCGGGATCGGCGCGCCGGTCGAGCTGTGGGGCAAAGAGATTAAAATCGACGACGTGGCGGCCAGCAGCGGCACCGTCGGCTATGAGCTGATGTGCGCCCTGGCGCCGCGGGTGCCAGTTGTGACCCTGTAA

>IR1230_00247

ATGCGTGTCGTCATACTGGGAAGTGGGGTGGTTGGGGTAGCCAGCGCGTGGTATTTGAGTCAGGCGGGTCATGACGTGACGGTCATCGACCGTCAGCCCGGTCCGGCAGAGGAGACCAGCGCGGCCAACGCCGGGCAGATTTCTCCCGGCTATGCGGCGCCCTGGGCGGCGCCGGGGGTGCCGCTGAAGGCGATCAAATGGATGTTTCAGCGCCATGCGCCGCTGGCGATCGGTCTTGACGGCACCTCGTTCCAGTTGAAGTGGATGTGGCAGATGCTGCGCAACTGCGACACCCGCCACTATATGGAGAACAAAGGCCGTATGGTGCGCCTGGCGGAGTACAGCCGCGACTGCCTGAAGGCGCTGCGCGACACCACCGGCATTCAGTATGAAGGGCGCCAGGGCGGGACGCTGCAGCTGTTCCGTACCGCCAAACAGTATGAAAATGCCACCCGCGACATCGCGGTGCTGGAGGACGCCGGCGTGCCGTATCAGCTGCTGGAAGCGAAGCGGTTGGCGGAGGTGGAGCCGGCGCTGGCGGAGGTCAGCCACAAACTGACCGGCGGCCTGCGCCTGCCAAATGATGAAACCGGCGACTGCCAGCTGTTTACCACCCGTCTGGCGGCAATGGCGGAACAGGCCGGCGTGACCTTCCGCTTCAACACCGCAGTGGATGCGCTGCTGCACGAGGGCGATCGCATTGCCGGGGTGAAATGCGGCGATGAGATTATTAAGGGCGACGCCTATGTGATGGCCTTCGGCTCCTACTCGACGGCGATGCTTAAAGGGCTGGTGGATATACCGGTCTACCCGCTGAAAGGCTACTCATTGACTATTCCGATCGCTCAGGAGGACGGCGCGCCGGTTTCAACTATCCTTGATGAGACTTACAAAATCGCCATCACCCGCTTCGATGAGCGCATTCGGGTCGGCGGCATGGCGGAGATCGTCGGTTTTAACAAAGCGCTGCTGCAGCCGCGTCGCGAAACCCTGGAGATGGTGGTGCGCGATCTGTTCCCGCGCGGCGGCCACGTGGAGCAGGCGACGTTCTGGACGGGCCTGCGGCCGATGACGCCGGACGGCACGCCGGTGGTCGGCCGCACAGCGTATAAAAATCTGTGGCTCAACACCGGCCACGGCACGCTTGGCTGGACCATGGCCTGCGGCTCCGGGCAGCTTATCAGCGATCTGATCTCCGGCCGCACGCCGGCGATCCCTTACGACGATCTGGCGGTTGCCCGCTACAGCCCCGGTTTTACCCCGGCGCGCCCGCAGCACCTGCACGGCGCACACAACTAA

>IR1230_00248

ATGGCTACGATTGACTCCATGAACAGGGACACCACCCGTTTAAGCGATGGACCCGACTGGACGTTCGAGTTACTGGAAACCTACCTGGCCGAAGTGGACCGGGTCGCCAAGCTCTATCGTCTTGACACCTACCCGCACCAGATCGAAGTCATTACTTCCGAGCAGATGATGGACGCCTACTCCAGCGTCGGCATGCCCATCAACTATCCGCACTGGTCGTTTGGCAAAAAATTTATTGAGACCGAGCAGGCCTATAAGCACGGTCAGCAGGGTCTGGCGTATGAGATAGTCATCAACTCCAACCCCTGTATCGCCTACCTGATGGAGGAAAACACCATCACCATGCAGGCGCTGGTGATGGCCCATGCCTGCTACGGGCATAACTCGTTTTTTAAAAATAACTATCTGTTTCGCAGCTGGACCGACGCCAGCTCGATCATCGACTATCTGATCTTTGCCCGTAAGTACATCACCGAGTGCGAAGAGCGCTATGGCGTCGACGAAGTAGAGAAACTGCTCGACTCCTGCCACGCGCTGATGAACTACGGCGTCGACCGCTATAAACGTCCGCAAAAAATCTCTCTGCAGGAGGAGAAAGCGCGGCAGAAAAGCCGGGAAGAGTATCTGCAAAGCCAGGTGAATATGCTGTGGCGCACCCTGCCGAAGCGCGAGGAAGAAAAAGCGATTGAATCCGCTCGTCGCTATCCCTCCGAACCGCAGGAGAACCTGCTGTACTTTATGGAGAAGAACGCCCCGCTGCTCGAACCGTGGCAGCGCGAGATCCTGCGCATCGTGCGTAAAGTCAGCCAGTATTTCTACCCGCAGAAACAGACCCAGGTGATGAACGAGGGGTGGGCCACCTTCTGGCACTACACCATCCTCAACCATCTCTACGATGAAGGGAAAGTGACTGAGCGCTTTATGCTGGAGTTTCTCCACAGCCATACCAACGTGGTGTTCCAGCCGCCGTACAACAGTCCGTGGTACAGCGGGATTAACCCCTACGCGCTGGGCTTCGCCATGTTCCAGGACATCAAGCGCATCTGTCAGTCGCCCACGGAAGAGGATAAGTACTGGTTTCCCGATATCGCCGGTTCTGACTGGCTGGAAACGCTGCATTTCGCCATGCGCGACTTTAAGGATGAAAGCTTTATCAGTCAGTTCCTGTCGCCGAAGATCATGCGCGATTTCCGCTTCTTTACCGTGCTCGACGACGATCACAACAATTATCTGGAGATCTCGGCGATTCACAACGAAGAGGGTTATCGCGAGATCCGCAATAAGCTCTCCGCGCAGTATAACCTCAGCAATCTTGAGCCGAACATCCAGGTGTGGAACGTTGACCTGCGCGGCGACCGATCGCTGACCTTGCGCTATGTGCCGCACAACCGCGTCCCGCTGGACAAAGGCCGGCGCGAAGTGCTGAAGCATGTGCATCGTCTGTGGGGGTTCGATGTGCTGCTGGAGCAGCAGAACGCCGACGGCAGCATTGAGCTGTTGGACCGCTGCCCGGCGCGGCCGAACGCGCTATAG

>IR1230_00249

ATGGTCATTAAGGCGCAGAGCCCTGCGGGTTTCGCGGAAGAGTACATCATTGAGAGTATCTGGAATAACCGCTTCCCTCCCGGATCGATTCTCCCCGCTGAACGCGAGCTTTCTGAGCTGATCGGTGTCACCCGCACAACCCTGCGCGAAGTATTGCAGCGTCTGGCCCGTGACGGCTGGCTGACCATTCAGCATGGTAAGCCCACTAAAGTGAATAACTTTTGGGAAACCTCAGGCCTGAATATCCTCGAAACGCTGGCGCGCCTCGATCACGACAGCGTACCGCAGTTGATTGATAATCTGCTCTCCGTGCGCACCAACATTTCCACGATTTTTATCCGCACCGCGTTTCGCCAGCATCCGGATAAAGCGCTGGCGGTCCTGGACAGCGCCCGGGAGGTGGAGGATCACGCCGACGCCTTCGCCGAGCTTGATTATAATATTTTCCGTGGTCTGGCGTTTGCCTCCGGCAACCCGATCTACGGTCTGATCCTCAACGGCATGAAGGGGTTGTATACCCGCATCGGCCGCCACTATTTCTCCAGCCCGGAAGCGCGCAGCCTGGCGCTCGGTTTCTATCATCAGCTGGCGAAAGTGTGTGAGGCTGGCCTGCATGACCAGGTTTACGAGCTGGTTCGTCGCTACGGCCATGACAGTGGCGAGATCTGGCACCGGATGCAGAAATCGCTGCCCGGTGATTTAGCCATGAATATGCGTTAA

>IR1230_00250

GTGGAAATTTCTTATGGCCGCGCGCTGTGGCGCAATTTTCTTGGCCAGTCGCCGGACTGGTACAAGCTGGCATTAATCATTTTCTTAATCGTAAATCCGCTGGTGTTCGCCGTGGCGCCGTTTGTCGCCGGCTGGCTGCTGGTGGTCGAGTTTATCTTTACCCTCGCCATGGCGCTGAAGTGCTATCCGCTGCTGCCGGGTGGTCTGCTGGCTATCGAGGCGCTGCTGATCGGCATGACCAGCCCGGCGCACGTGCGGGACGAGATCGCCGGCAATCTGGAGGTGCTGCTCCTGCTGATGTTCATGGTGGCGGGTATCTACTTTATGAAACAGCTTTTGCTGTTCGTGTTTACGCGCCTGCTGCTGGGGATCCGCAGCAAAATGCTGCTATCGCTGTCCTTCTGCCTGGCGGCCGCCTTCCTCTCGGCCTTTCTCGATGCGCTGACGGTGGTGGCGGTAGTGATCAGCGTCGCCGTCGGGTTCTACGGCATCTATCACCGCGTCGCCTCGGCGCGCCCGGACGATAGCGACCTGCTCGATGACAGCCATATCGAGCAGCACTACCGTGAAGTGCTGGAGCAGTTCCGCGGCTTTCTGCGCAGCCTGATGATGCACGCCGGCGTCGGGACCGCGCTCGGCGGCGTCATGACCATGGTCGGCGAGCCGCAAAACCTGATTATCGCCAAAGCCGCGGGCTGGCATTTTGGCGAGTTCTTTCTTCGCATGGCGCCCGTGACCCTGCCGGTGATGGTCTGCGGTCTGTTAACCTGTCTGCTGGTCGAAAAGTATCGCCTGTTTGGCTACGGCGAACCGCTACCGCCGACGGTGCGCAAGGTGCTGCAGGAGTTTGACGATCGCAGCCGCGCCCAGCGCAGCCGTCAGGAGCGACTGCGGCTGATTGCCCAGGCGCTCATTGGCGTTTGGCTGATCGTGGCCCTGGCTTTTCACCTGGCTGAGGTCGGCCTGATTGGCCTGTCGGTGATCATCCTCGCCACCACCTTTACCGGCGTTACCGATGAGCACGCCATTGGCAAGGCGTTCACCGAGGCGCTGCCCTTTACCGCGCTGCTGACGGTCTTCTTCTCCATCGTCGCGGTTATCATCGATCAGCAGCTGTTCACGCCGGTGATCGAGTTTGTCCTGCAGGCCTCGCCGCACGCGCAGCTGTCGCTGTTTTATCTGTTCAACGGCCTGCTGTCGTCCATTTCGGATAACGTGTTTGTCGGCACGGTCTACATCAACGAAGCGAAAGCGGCGCTCGAACATGGCGCCATCAGCCTGCCCCAGTTTGAGATGCTGGCGGTAGCGATCAACACCGGCACCAACCTGCCCTCGGTGGCCACCCCGAACGGCCAGGCGGCGTTTCTCTTCCTGCTGACTTCCGCCCTGGCGCCGCTTATTAGGCTCTCCTATGGCCGTATGGTGTGGATGGCGCTGCCCTACACCATCGTACTGACGCTGGTCGGCCTGTTATGCGTCGAGTTCACCCTGATGCCAGTCACCGACTGGCTGCTGGCGCACGGCTGGCTGGTGACGCCCACCCTGCCCTGA

>IR1230_00251

ATGTTGCAATATTTAAACCAGTGCTCAAGAGGACGCGGTGCCTGGCTCCTGATGGCGTTGACCGCTTTTATTCTCGAACTTGTCGCGCTGTGGTTCCAGCACGTGATGCTGCTGCAGCCGTGTGTCATGTGTATTTACGAACGCTGCGCGCTGTTCGGCATCATGGGCGCGGGACTGGTGGGCGCCATCGCCCCCAAAACGCCGCTGCGCTATGTCGCCATGGCAATCTGGTTGTACAGCGCGATACGCGGACTACAGCTGGCGTGGGAGCATACGATGATCCAGCTGCACCCTTCACCGTTCCAGACCTGCGATTTCGCCGCCCGCTTCCCAACCTGGCTGCCGCTGGATAAATGGCTGCCGCAGGTTTTCGTTGCGTCCGGAGACTGTTCAGTACGTCAGTGGCAGTTCCTGTCGCTGGAGATGCCGCAATGGCTGGTGGGGATTTTCGCCGCCTATCTGCTGGTCGCTATTCTGGTGATCGTTGCTCAGCCTTTCAAAGCGAAAAAACGCGATCTGTTTGGTCGTTAA

>IR1230_00252

ATGTCATCTTTACTGATCCCCGCAGACTGGAAAGTTAAACGCTCCACCCCATTCTTTACCAAAGAGAATGTCCCTGCCGCCCTGCTGAGCCACCACAACACCGCGGCTGGCGTCTTCGGCCAGCTGTGCGTCATGGAGGGAACGGTGACCTATTACGGTTTTGCCAATGAACAGGCGACGGAGCCGGAGAAAAAAGTCGTGATTCATGCCGGGCAGTTTGCTACCAGTCCGCCGCAGTACTGGCACCGCGTCGAACTCAGTGACGACGCCCGCTTCAATATTCACTTCTGGGTGGCGGAAGAAACCGACGGTGAAAACGGGCTGTTCCACGCGAAGAAAGCGTGA

>IR1230_00253

ATGAGCGAACAACCTTTCTGGCAACAAAAAACACTGGATGACATGAGCGACGCGGAGTGGGAATCGCTGTGTGACGGCTGCGGCCAGTGCTGCCTGCACAAGCTGATGGATGAAGACACTGACGAGATCTACTTCACCAACGTCGCCTGTCGTCAGCTCAATATCAAAACCTGCCAGTGTCGTAACTACGCGCGTCGCTTTGAGTATGAGCCGGACTGCATCAAGCTTACCCGCGAAAATCTGCCGACCTTCGAATGGCTGCCGCCGACCTGTGCCTATCGCCTGCTGGCGGAAGGCAAGCCTCTGCCGGCCTGGCACCCGCTGCTGACTGGCTCAAAAGCGGCGATGCACGGCGAACGCATCTCCGTTCGCCATATTGCGGTGCCTGAGTCCACGGTGGTCGACTGGCAGGACCATATCCTTAATCTGCCTGACAGGGCGCGCTAA

>IR1230_00254

ATGTACCAACATCATAACTGGCAGGGTGCGCTTCTGGATTATCCAGTAAGCAAAGTAGTGTGCGTCGGCAGTAATTATGCGAAACACATCAAGGAAATGGGCAGTGCGACGCCGGAAGAGCCGGTGCTGTTTATTAAACCTGAGACTGCGCTGTGCGATCTTCGTCAGCCGTTGGTGCTTCCTGAGGGGCTGGGCTCGGTGCATCACGAAGTCGAGCTGGCGGTGCTTATCGGCAGCACGCTGCGTCAGGCGACGGAAGAGCATGTGCTGAAAGGGATTGCCGGCTATGGTGTCGCGCTGGATCTCACGCTGCGCGACCTGCAGGCGAAAATGAAAAAAGCGGGACAACCCTGGGAGAAGGCCAAAGGCTTCGATAACGCCTGTCCGATCTCCGGCTTTATCCCGGCCGCTGAGTTCCACGGCGACCCGCAGAATACGTCGTTAAGCCTGAAGATTAATGGCGAAGTGCGCCAGCAGGGCACCACTGCCGACATGATCCATAAGATTGTGCCGCTGATCGCCTATATGTCGCGCTTCTTTACCCTCAAAGCCGGGGATGTGATCCTCACCGGCACCCCGGAAGGCGTGGGGCCGCTGCACAGCGGTGACGAGCTGGAGGTCGGCTTCAACGGCCTGGCGCTCACTACCCGCGTGCTGTAA

>IR1230_00255

ATGTTTTGTGTGATCTATCGAAGTACTAAACGTGAACAAACCTATTTATACGTCGAAAAAAAGGACGATTTCTCGCGCGTTCCTGACGAGCTAATGCGCAGCTTTGGCACGCCGCAAATGGCGATGCTGCTGCCGCTGGATGGGCGCAAAAAACTGGTTAACGCCGATCTGGAAAAAGTGAAACAGGCGTTAAGCGAACAGGGCTATTACCTGCAACTGCCTCCGCCGTCTGAAAATCTACTAAAGAAACACCTGGCGGAACAGGGGAAACAATCCGATTAA

>IR1230_00256

ATGTCAAATACGCCAATCGAACTTAAAGGCAGTAGCTTCACGTTATCTGTCGTTCATTTGCACGATGCAAATCCCGAGGTTATTCGTCAGGCGTTAGAAGACAAAATCGCCCAGGCTCCCGCCTTTTTACGTCATGCTCCGGTGGTGGTGAATATCGCCAGCATCGAGGAGGAGGTAGAATGGCGCGCCATCAACGAGGCTATCGCCGCGACCGGTTTACGTATTATGGGCGTTAGCGGATGCAAAATTCCGCGCCTGAAAACCGAAATCGACCGCGCCGGCATCCCGTTATTAACCGAAGGGAAAGAAAAAGCCTCTCGTCCGGCACCGTCCGAACCCACCCCGCCGCCGCCAGTTGCTAGTCAGATCACAAAAACGCGTTTGATTGATCAGCCGGTACGTTCCGGTCAGCGCATTTATGCGCCACACTGTGATCTTATTGTTACAAATCATGTGAGTGCCGGTGCGGAACTTATCGCTGACGGAAATATCCATGTATATGGCATGATGCGAGGACGCGCGCTGGCAGGCGCCGGTGGCGACAGAGACGCCCAGATATTTTGTACCCACCTTGCGGCGGAGCTGGTCTCCATCGCCGGGGAATATTGGCTGAGCGATAACATCCCGGCCGAATTTTATGGCAAAGCGGCGCGCCTGCGTTTAGGTGAAAGCGCTTTGGCAATTCAACCGTTAAATTAA

>IR1230_00257

ATGGCACGCATTATTGTTGTGACTTCGGGTAAAGGGGGCGTTGGCAAGACCACCTCCAGCGCGGCCATCGCTACAGGTTTGGCCCAGAAGGGAAAGAAAACCGTCGTTATCGACTTCGACATCGGCCTGCGTAACCTCGACCTGATTATGGGCTGCGAACGTCGCGTCGTTTATGATTTCGTCAACGTCATTCAGGGCGATGCCACACTGAACCAGGCGCTGATTAAAGATAAGCGCACGGAAAATCTCTACATTCTCCCGGCTTCCCAGACCCGGGATAAAGACGCTCTGACTCGCGAAGGCGTCGACAAGGTTCTCGAAGAACTGAAGAAAATGGAATTCGATTTCATCGTCTGTGATTCCCCGGCAGGCATTGAAACCGGTGCGCTGATGGCGCTTTACTTTGCTGATGAAGCCATCATCACCACTAACCCGGAAGTCTCCTCCGTTCGCGACTCCGACCGTATCCTTGGCATTCTCGCCTCTAAATCCCGCCGCGCGGAAAATGGCGAAGAGCCAATCAAAGAGCATCTGCTGCTGACCCGCTACAATCCAGGCCGCGTTAATAAAGGCGATATGCTGAGCATGGAAGACGTGCTGGAAATTCTGCGCATCAACCTGGTAGGCGTGATCCCGGAAGACCAGTCAGTCCTGCGCGCATCCAACCAGGGTGAGCCGGTGATCCTCGATGCCGCCTCGGACGCAGGCAAAGCCTATGCCGATACCGTTGAACGTCTGCTCGGAGAAGAACGCCCTTTCCGCTTCATTGAAGAAGAGAAGAAAGGATTCCTCAAACGCCTGTTCGGAGGATAA

>IR1230_00258

ATGGCTTTACTCGACTTTTTTCTCTCGCGAAAAAAGAACACGGCTAATATTGCGAAAGAACGCCTGCAAATCATCGTCGCCGAGCGCCGCCGCGGAGACGCGGAGCCGCATTACCTGCCGCAGTTACGCAAAGATATCCTGGAAGTGATCTGTAAATACGTACAGATCGACCCGGAGATGGTCAGCGTGCAGCTGGAGCAGCGGGACGGGGATATATCCATTCTTGAGCTGAACGTGACCCTACCCGAAACGGAAGAGTCGAAATCCTGA

>IR1230_00259

ATGATCACCACCGACGATGGCCTGCGCGCTGTCTGCGAAGCGGCAAGCACGGCCTCCGCCGTCGCTCTGGATACGGAATTTGTCCGCACCCGCACCTATTACCCGCAGCTGGGCCTGCTGCAGCTGTTCGACGGCCAGCAGGTCTCTCTGATCGATCCCTTGACGATTAACGACTGGGCGCCGATGCGCGACCTGCTGCTCAACCAGGATGTGACTAAATACCTGCATGCCGGCAGCGAAGATTTAGAAGTCTTCCTTAATGCATTTAACCTGATGCCGCAGCCGCTGATTGACACCCAGATCCTGGCGGCCTTCTGCGGCCGCCCAATGTCCTGGGGCTTCGCCTCGATGGTGGAAGAGTATTCCGGGGTAGCGCTGGATAAAAGCGAATCCCGCACCGACTGGCTGGCGCGCCCGCTGACTGAGCGTCAGTGTGAATATGCCGCGGCCGACGTCTGGTATCTGCTGCCGATCGCCAGCCAGTTAATGGCGGAGACCGATCGCGCTGGCTGGCTGCCGGCGGCGCTCGACGAGTGCCGCGTGATGCAGCAGCGTCGTCAGGAGGTGGTCGACCCGGCCGAGGCCTGGCGCGATATCGGCAACGCCTGGCAGCTGCGTACCCGCCAGCTGGGTTGTCTGCAGCTGTTGGCGGAGTGGCGCCTGCGCAAAGCGCGCGAGCGCGACCTGGCGGTAAACTTTGTGGTGCGGGAAGAGCATCTGTGGAGCGTGGCGCGCTATATGCCCACCAGCCTGGGCGAGCTCGACAGCCTCGGGTTATCCGGTAGCGAAATTCGCTTCCACGGCAAAACGCTGATTAGCCTGGTCGAAAAAGCGCAGGCGCTGCCGGAGTCGGCCCTGCCGGCGCCGCTGCAGAACCTGATCGACATGCCGGGCTACCGCAAAGCCTTTAAAGATATTAAAGCGCTGGTGCAGGAAGTGAGCACGGAGAAGGGCGTAAGCGCGGAGCTGCTGGCGTCGCGTCGGCAGATCAACCAGCTGCTGAACTGGCACTGGCAGCTGAAAACGCAAGCCGGTGAACCTGAGCTGATTTCCGGTTGGCGCGGGGAGCTGATGGCCGAGCGGCTGAAGAGGCTGTTAAACGACTATCCGCGCTAG

>IR1230_00260

ATGACGACGAATAACTATTTCAGAGGTGATGCAGTGAAAAAGGTTTGGCTAAACCGTTATCCCGCAGATGTTCCGGCGGAGATAAATCCTGACCGCTATCAATCCCTGGTTGAACTGTTTGAACATGCCACCACCCGTTACGCCGACCAACCGGCGTTTATCAATATGGGTGAAGTGATGACCTACCGTAAGCTGGAGGAGCGTAGTCGCGCCTTCGCCGCTTATCTTCAGGAGGGGTTGGGGTTACAGAAGGGCGATCGCGTGGCCCTGATGATGCCTAACCTGCTGCAGTATCCGGTGGCTCTGTTTGGCATCCTGCGCGCCGGGATGATCGTGGTTAACGTCAACCCCTTATATACCCCGCGCGAACTGGAGCATCAGCTCAACGACAGCGGCGCGGCGGCGATCGTCATAGTCTCCAACTTTGCCCATACCCTGGAAAAAGTGGTGGCGAAAACTCAGGTCCAGCATGTGATCCTGACGCGCATGGGCGACCAGCTTTCCACCGCCAAAGGCACGCTGGTGAACTTTGTGGTCAAGTACATTAAGCGCCTGGTGCCGAAGTACCACCTGCCGGACGCCATCTCGTTTCGCAGCGCGTTGCAGCACGGCTATCGCATGCAGTACGTCAAGCCGGAGATCGTTGCGGAAGATCTGGCGTTTCTGCAGTACACCGGGGGCACCACCGGCGTGGCCAAAGGTGCGATGCTCACCCACCGCAATATGCTGGCTAACCTCGAGCAGGTCAATGCCACCTACGGCCCGCTGCTGCATCGCGGCAAAGAGTTCGTGGTGACCGCGCTGCCGCTGTATCACATTTTCGCCCTGACCATGAACTGCCTGCTGTTTATCGAACTGGGCGGGCAAAATTTGCTGATCACCAACCCACGCGATATCCCCGGGCTGGTGAAGGAGCTGGCGAAGTATCCTTTTACCGCCATGACCGGGGTCAATACCCTGTTCAACGCCTTGCTGAACAACAAAGAGTTCCAGCAGCTTGATTTCTCCTCGCTGCATTTGTCCGCCGGCGGTGGGATGCCGGTGCAGCAGGCGGTGGCCGAGCGCTGGGTAAAACTGACCGGGCAATATCTGCTGGAGGGCTACGGCCTGACAGAGTGTTCTCCGCTGGTCAGCGTTAACCCGCACGATATTGACTACCACAGCGGCAGCATTGGCCTGCCGGTGCCCTCCACCGAAGCCAAACTGGTGGATGATGATGATAACGAAGTGGCGCCGGGTCAGCCGGGCGAGCTCTGTATTAAAGGGCCGCAGGTGATGCTGGGCTACTGGCAGCGCCCTGACGCGACGGATGAGATTATCAAAGACGGCTGGCTGCACACCGGGGATATCGCGGTGATGGATGAAGAGGGGTTCCTGCGCATCGTCGATCGCAAAAAAGATATGATCCTGGTGTCGGGTTTTAACGTCTATCCGAACGAAATCGAAGATGTGGTCATGCAGCATAGCGGGGTGCTGGAAGTGGCGGCCATCGGCGTGCCGTCAGGCAGCAGCGGCGAAGCGGTGAAGATTTTCGTAGTCAAGAAAGATGCTGCGTTAACCGAAGAGGCGCTGATTACGTTCTGCCGTCGCCATTTGACCGGATACAAGGTGCCGAAGCTGGTGGAATTCCGTGATGAACTGCCGAAATCCAACGTCGGGAAAATTTTGCGGCGAGAACTGCGTGACGAAGCGCGCGCTAAAGTGGACAATAAAGGCTAA

>IR1230_00261

ATGGCGGGTCAAAAACAGGGCGTTCGCTGGCTGTTAGCCGCGGCGGTTGCCGTTGCGCTAAGCGGCTGCGTGTCAGTGCCGGATGCCATCAAAGGCACCAGCCCGACGCCGCAGCAGGATTTGGTGCGGGTGATGAACGCCCCGCAGCTGTACGTTGGCCAGGAAGCGCGCTTTGGCGGTAAGGTGGTCAATGTGCAAAACCAGCAGGGGAAAACCCGCTTAGAAATCGCCACCGTACCGCTGGACAGCGGCGCGCGGCCAGTGCTGGGCGAGCCGTCCCGCGGAAGGATTTTTGCTGACGTCAATGGCTTCCTCGATCCGGTGGATTTCCGCGGTCAACTAGTGACCGTGGTCGGACCGATCGCCGGGGTGGTTGACGGCAAGGTGGGCAGCACGCCGTATAAATTTATGCTGATGAACGCCACCGGCTACAAGCGCTGGAACGTAGTGCAGCAGGTGGTGATGCCTCCGCAACCTATCGACCCGTGGATGCTGGGTCCGCGCCCCTGGGGCTACGGCTATGGCGGCTGGGGCTGGTACAATCCCGGCCCTGCTGAGGTGAGAAACGTTGTAACCGAATGA

>IR1230_00262

ATGCGAATTTTGGCTATCGATACCGCCACAGAGGCCTGCTCCGCGGCTCTGTGGAATGATGGCACCCTTAGTGCTCATTTCGAAATTTGTCCCCGCGAACATACCCAACGTATCCTGCCGCTGGTGCAGGAGGTCCTCACCGAGAGCGGCACCACGCTGAGCGAGCTGGACGCGCTGGCCTTTGGCCGCGGTCCGGGCAGCTTTACCGGCGTGCGCATCGGCATCGGTATCGCTCAGGGGCTGGCGCTGGGCGCCGAACTGCCAATGATTGGCGTTTCCACGCTGGCCACCATGGCGCAGGGCGCCTGGCGCAAAACCGGCGCCACCCGTGTGCTGGCGGCCATTGATGCGCGTATGGGCGAAGTCTACTGGGCCGAGTACCAGCGCGACGAGCAGGGCGTCTGGCATGGCGAAGAGACGGAAGCGGTGCTCAAACCGGACGCGGTAGCCGAACGACTGGCACAGCTTTCCGGCGAATGGGCCACCGTCGGCACCGGATGGCAGGCGTGGCCGGATCTGGCGAAAGCGAGCGGACTGACCTTAAGCAGCGGCGAAATTGAATTGCCGGCGGCGGAGGATATGCTGCCGTTAGCCTGTTACCTGCTGGCGGCGGGGAAAACCGTGGCCGTGGAGAAAGCGGAGCCGGTTTATTTGCGAAACGAGGTGGCGTGGAAGAAACTTCCAGGCCGCGAGTGA

>IR1230_00263

GTGATCGACGATTTTGCAGCAGACGGCCAGCTAGCCAAAGCCATACCGGGATTTAAACCGCGCGAGCCGCAGCGCCAGATGGCGGTGGCGGTCAGCGAAGCGATTGAGGCCTCCCGGCCGCTGGTGGTGGAAGCGGGGACCGGAACCGGTAAAACCTACGCTTACCTGGCGCCTGCGCTGCGGGCGAAAAAAAAGGTGATTATCTCCACCGGCTCGAAAGCGCTGCAGGATCAGCTTTACAGCCGCGATCTGCCCACCGTCGCCAAAGCGCTCAAATTCACCGGGAAACTGGCGCTGCTCAAAGGGCGCTCCAACTACCTGTGTCTCGAACGTCTTGAGCAGCAGGCGCTGGCGGGCGGCGATCTGCCGGTGCAAACCCTCAGCGATGTGATCCTCCTGCGCTCCTGGTCGAATCAAACCCAGGATGGCGATATCAGCACCTGCGCCAGCGTCGCGGAAGACTCTCAGGCCTGGCCGCTGGTCACCAGCACCAACGATAACTGTCTCGGTAGCGACTGCCCACTGTATAAAGATTGCTTCGTGGTGAAGGCGCGTAAAAAAGCGATGGACGCCGACGTGGTGGTGGTCAACCATCACCTGTTTCTGGCCGATATGGTGGTCAAAGAGAGCGGCTTTGCCGAGCTGATCCCCGAGGCGGAAGTGATGATCTTCGATGAAGCCCATCAGCTGCCGGATATCGCCAGTCAGTACTTTGGCCAGTCGCTCTCCAGCCGCCAGCTGCTGGACCTGGCGAAAGACATCACCATTGCCTACCGCACCGAACTGAAAGACACTCAGCAGCTGCAAAAATGCGCCGACCGCCTGGCGCAAAGCGCGCAGGATTTCCGCCTGCAGCTGGGAGACCCGGGCTACCGCGGCAATCTGCGCGAGCTGCTGGCGGACAGCCACATTCAGCGAGCGCTGCTGCTGCTCGATGACGCGCTCGAGCTATGCTATGACGTCGCCAAACTTTCGCTGGGTCGCTCGGCGCTGCTCGACGCCGCTTTTGAGCGCGCCACCCTCTATCGCGGGCGCCTGAAGCGGTTGAAAGAGATCAACCAGCCGGGCTACAGCTACTGGTATGAGTGTACCTCGCGCCACTTTACTCTCGCGCTGACGCCGCTGACCGTGGCCGAGAAGTTTAAAGAGGTGATGGCGCAGAAGTCGGGGAGCTGGATCTTTACCTCGGCGACGCTGTCGGTGAACGACGATCTGCATCACTTTACCGCCCGGCTGGGGATCGACGAGGCGCAGACTCTACTGCTGCCGAGTCCCTTTGACTATCAGCACCAGGCGCTGCTCTGCGTGCCGCGCAACCTACCCCTGCCGAACCAGCCCGGCGCGGCGCGACACCTGGCGGCGATGCTCAAGCCGCTGATCGAGGCCAACGATGGTCGCTGCTTTATGTTGTGCACCTCGCACGCCATGATGCGCGATCTGGCTGAGCAGTTCCGCGCCACCATGACGCTACCGGTGCTGCTGCAGGGCGAGACCAGCAAAGGCCAGCTGCTGCAGCAGTTCGTCAGCGCCGGCAACGCGCTGCTGGTGGCTACCAGCAGCTTCTGGGAGGGAGTCGATGTGCGCGGCGACGCGCTGTCGCTGGTGATTATCGATAAGCTACCGTTCACCTCGCCGGACGATCCGCTGCTAAAAGCGCGGATGGAAGATTGCCGGCTGCGCGGCGGCGATCCCTTCGATGAAGTGCAGCTCCCGGATGCGGTGATCACCCTCAAGCAGGGGGTGGGTCGCCTGATCCGCGATATCGACGACCGCGGGGTGCTGGTGATCTGCGACAACCGGCTGGTGATGCGCCCCTACGGCGCGGTGTTCCTGGCCAGTCTGCCGCCGGCGCCGCGAACCCGCGATATCCGTCGGGCGGTGCGCTTTCTCGCCGTACCGCCGGCAAGGTAA

>IR1230_00264

ATGACAATTACGCGTATTGATGCCGAAGCCCGTTGGTCAGACGTGGTGATCCACAACCAGACCCTGTACTACACCGGCGTGCCGGCAAATCTCGATGCCGACGCTTTCGAGCAGACCGCCAATACCCTGGCGCAGATTGACGCGGTGCTGGAAAAACAGGGCAGCGACAAGTCGCGTATTCTTGACGCCACCATCTTCCTCGCCGATAAAAGCGACTTTGCGGCGATGAATAAAGCCTGGGACGCGTGGGTGGTGGCTGGTCACGCCCCGGTGCGCTGCACGGTGGAAGCGACGCTGATGAACCCGCAGTATAAAGTTGAGATCAAAATTATCGCCGCGGTGTAA

>IR1230_00265

ATGTTTGCAGGTTTACCTTCGCTGAGCCACGAGCAACAGCAAAAAGCGGTCGAACGTATCCATGAGCTAATGGCTCAGGGGATAAGCAGCGGCCAGGCGATTGCCCTGGTGGCGGAAGAGCTGCGCGCAACGCATACCGGCGAGCAGATCGTTGCGCGCTTTGAAGATGAGGATGAGGACGAGTAA

>IR1230_00266

ATGTTGTCCCCCGCGATTATTACCCTGCCCTGGCGCCCGGATGCGGCCGAACACTATTTTGCGCCGTTAAGCGCCCTGCCCTGGGCGATGCTGCTGCATTCCGGCTTTGCCGACCATCCGCATAACCGCTTTGATATTCTGGTGGCTGCCCCGCGCGCCACGCTACTGACGCGCGATGAACAGACGTGGGTCGACGACGGCGAAACCGTTGTTGTCTCAGCGGAAGATCCGCTGCAGCTCCTGCAGCAGCAGTTGGATCGTCAGCCGTTCACGCCGCAACCCCATGACGATCTGCCGTTTCTCGGCGGCGCGCTGGGATTATTCGGTTACGACCTGGGCCGTCGCTTTGAGCGTCTCCCTTCGCACGCGCAGGCCGATATCGCGCTGGCGGATATGGCGGTGGGGATCTATGACTGGGCGCTTATCGTCGACCACCAGCGCCAACAGATATCGCTGCTCAGCTATGACGACCCGCAGCAGCGTCTACAGTGGCTGGAGGCGCAAACGCCGACGCCGGGTGAAACCTTCGCCCTGACCTCTGCCTGGCAGTCAAATATGAGCCGCCAGCAGTACGGCGAAAAATTTCGCCAGGTACAGGCCTATCTGCACAGCGGCGACTGCTATCAGGTCAACCTCGCCCAGCGTTTTCAGGCCAGCTACGTCGGTGATGAATGGCAGGCCTTCCGCCAGCTGAACGCCGTCAACCGCGCCCCCTTTAGCGCCTTTATTCGCCTCGATGAAGGGGCGATTTTAAGCCTGTCGCCGGAGCGCTTTATCCAGCTGCGCCAGGGGGAGATCCAGACGCGGCCGATAAAAGGCACCCTGCCGCGGCTCGATTCGCCGCTGGCAGATGCGCAGCAGGCTGAGAAGCTGGCGAATTCGCCGAAAGATCGCGCCGAGAATTTAATGATTGTCGACCTGATGCGCAACGACATCGGCCGCGTCGCCGTCCCGGGCAGCGTCCGGGTCCCCGAGCTGTTCGTGGTGGAGCCGTTCCCGGCGGTCCATCATCTGGTCAGCACCATCACCGCCCGCCTGCCAATGACGCTGCACGCCAGCGACCTGCTGCGCGCCGCCTTTCCCGGCGGCTCGATCACCGGCGCGCCGAAAGTGCGGGCGATGGAGATTATCGATGAACTGGAGCCCCAACGACGCAACGCCTGGTGTGGGAGCATTGGCTACCTGAGCTATTGCGGCAATATGGATACCAGCATCACCATTCGCACCCTGACGGCATGGCAGGGACAGCTGTACTGCTCCGCCGGCGGCGGAATTGTGGCGGATAGCGAGGAAGCGGCGGAATATCAGGAAACTTTTGATAAAGTTAATCGTATCCTGCAGCAACTGGAGAACTAG

>IR1230_00267

ATGGCGGATCGCGCCCTTAATCTGGATGATTTTCTGTCCCGCTTTCAGCTTCTGCGCCCGCAGCCTTCGCGCCATGCGCTCAATCAGCGGCAGGCGGCGGTGCTGGTGCCGATCGTGCGCCGGCCGCAGCCCGGCCTGCTGCTGACCCAGCGTTCGCCGCTGCTGCGCAAGCACGCCGGCCAGGTCGCCTTTCCCGGCGGCGCAGTAGATAATACCGACGCGACGCTTATCGCCGCCGCCCTGCGCGAAGCTCAGGAAGAGGTGGCGATCCCGCCGGAGGCGGTCGAGGTGATCGGCGTCCTGCCGCCGGTGGACAGCGTCACCGGCTTTCAGGTGACGCCGGTGGTGGGCATTATTCCCCCGGATCTGCACTACCACGCCAGCCAGGACGAAGTCTCCGCGGTATTTGAAATGCCGCTCGCCGAAGCCCTGCGCCTTGGGCGCTACCATCCACTCGATATCCACCGCCGGGGCAACGACCACCGCGTCTGGCTCTCCTGGTACCAGCATTATTTTGTCTGGGGAATGACCGCCGGGATCATTCGCGAGCTGGCGCTGCAAATTGGCGCACGTCCTTAG

>IR1230_00268

GTGATTAGTCTATTCGACATGTTCAAGGTGGGGATTGGTCCCTCATCTTCCCACACTGTTGGCCCGATGAAGGCCGGAAAACAGTTCGTCGATGACCTGGTCGAAAAAGGATTGCTTAATGCAGTGACTCGTGTGGCGGTTGACGTCTACGGTTCACTGTCATTAACCGGCAAAGGCCACCATACGGACATCGCCATTATCATGGGACTGGCGGGCAATCAGCCCGATACGGTCGATATTGACGCGATCCCGGCTTTTATCCGTGACGTCGAAGCGCGCGGTCGTCTGCTGCTGGCCAACGGCCAGCACGAGGTCGACTTCCCGGCGGATGACGGCATGCGTTTTCGCAGCGACAACCTGCCGCTGCACGAAAACGGCATGACCATTCACGCCTGGGCCGGCGAGAAAGAGATCTACTGCAAGACCTACTACTCGATCGGCGGCGGCTTTATTGTCGACGAAGAGCACTTCGGCAAGGAAAACGCCAACGAACTGCAGGTGCCCTACCCATTTAAATCGGCGCAGGAAATGCTGGCCTACTGCAAAGAGACTGGCCTGTCGCTCTCCGGGATGGTGATGCAGAACGAGCTGGCTCTGCACAGCAAAAAAGAGATCGAGGATTATTTCGCCAACGTCTGGCAGACCATGCGCGCCTGTATCGACCGCGGGATGAACACCGAGGGCGTGCTGCCTGGCCCGCTGCGCGTACCGCGTCGCGCCTCGGCGCTGCGTCGTCTGCTGGTGGCCAGCGATAAGCTCTCCAGCGATCCAATGAACGTTGTTGACTGGGTCAATATGTTCGCGCTGGCGGTCAATGAAGAGAACGCCGCAGGCGGTCGCGTGGTAACCGCGCCGACCAACGGCGCCTGCGGCATCGTTCCGGCGGTACTCGCCTATTACGATCACTTTATCGAGTCCGTCAGCCCGGAGATCTATATTCGCTACTTTATGGCCTGCGGGGCGATCGGCGCGCTGTATAAGATGAATGCGTCCATTTCCGGCGCGGAAGTCGGCTGTCAGGGCGAGGTGGGCGTCGCCTGCTCGATGGCGGCGGCGGGCCTGGCTGAACTGCTCGGCGCCAGCCCGGAACAGGTTTGCGTGGCAGCGGAGATCGGCATGGAACACAACCTCGGCCTGACCTGTGATCCGGTCGCCGGTCAGGTGCAGGTGCCATGCATTGAACGTAACGCCATCGCCTCCGTCAAGGCGATCAACGCCGCGCGAATGGCGATGCGCCGCACCAGCGAACCGCGCGTCTCGCTCGATAAGGTGATCGAGACCATGTATGAAACCGGTAAAGACATGAACGCCAAGTACCGCGAAACCTCGCGCGGCGGCCTGGCGATTAAAGTTCAGTGTGACTAA

>IR1230_00269

ATGCAGACTGCACAAAAAGTCATTACGGCCTATCGTCACAAACGCATTATTGTCTGCCTGCTGGTAGCCCTGGCGACCCTGGGCGCCACATTAGCCATCCGATTTATTTCGCAGCGCAGCGTAAATGAAGATTATATTCGCACGGCCGCCAGCCAACGGGTGGCGGCGCTCAATGACATCTTGCGCCCGCTAAGCGCACAGCGAGCCACCCTGCTCCCTCTGGTGGGCAAACCCTGCCCTGATATCCATCTGACGCTGCGCAAAATGGCGGCCTCGTTGCAAACCATTCGTTCCGTCGCCCTCGTCACGTCAGGTATCGTTTACTGTTCCAGTATTTTTGGCCCGCGACAGGCAGACTTACATCGCCTGCAGCCCGCGCTTCCTGCGCCCCGCCCTCTGCTGCTCTTCTCGAACGACAGCTCCTTGCTCAAAGGCTCGCCGGTACTGATCCAATGGTATCCAGCCGCGGAGAGCGGCCTGGACGGCGTGATGCTGGTGGTGAATATTGAGCTGCTGGGAACCTTAATTCTTAATGAAAAATCGGCCCTGATCAGCGACGTCAGCCTGCAGGTTGGCGATCGTTATTTTAGCAGCCGCCATGGGCTTCTCGAAAAAGCGCATGTCCCGCAGGGCACGGTGATTTATCGCCAGCGCTCTACGGAGTTTCCGTTTACTGTCAATATTAACGGCCCTGGCGCCTCGGCGATTGCGCTTGAGGAGCTGCCCGGCGAGCTGCCGCTGGCGCTGATTTTTAGCCTGCTAATGACCGGCATCGCCTGGCTGGCCACCGCCGGCAGAATGAGTTTCTCGCGGGAAATAAGCCTTGGCATTTCGGCCCGGGAGTTTGCCCTCTGGTGCCAGCCGCTGCAGGATGCGCGCAGCGGCCGCTGCTGCGGGGTGGAGATCCTGCTGCGCTGGAATAATCCCCGCCGTGGGACGATTTCGCCGGAGGTATTTATCCCCATCGCCGAAGGCAATAATCTGATCATCCCTCTCACCCGCTATGTGATTGCCGAAACCGCTCGCCGCCTGGACGCTTTTCCTCGCGACCGCCATTTTCACATCGCGATCAATGTCGCCGCGCGCCATTTCGCTAACGGACTCCTGCTGCGCGACCTGCACAATTACTGGTTCAGCGTGGATCCGGTGCAGCAGCTGGTGGTCGAGCTAACCGAACGCGATGTCCTGCAGGATGGCGACCAGCATATGGCCGAACATCTGCACTTTAAGGGCGTACAGCTGGCGATCGATGATTTTGGTACCGGCAATACTTCGCTCTCATGGCTGGAAAAGCTGCGCCCGGATGTTTTGAAGATCGACCGCTCGTTCACCAGCTCGGTCGGCATAGATAGCGTGAACGCGACGGTGACAGATATTATTATCGCCCTCGCCAACCGCCTGCATATTGTTACCGTTGCCGAAGGGGTGGAGACGCTGGAGCAGGAAAACTACCTGCGTAGCCACGGCGTCGATGTGCTGCAGGGGTTTTATTATGCGCGGCCGATGCCGGTGGAGGCGTTCCCGGCGTGGCTGGCCAGCAGGGAGGAGAGTGAGAGTGAGAGTGAGAGCGAGACGACAGAATAA

>IR1230_00270

ATGGAATTGTTAATGGATCCCTCAATCTGGGCCGGCCTGTTGACGCTTATCGTTCTGGAAATTGTGCTCGGTATCGACAACCTGGTGTTTATTGCCATCCTGGCGGACAAACTGCCGCCGAAGCAGCGCGATAAAGCGCGTCTGATCGGCCTGTCACTGGCGCTGGTGATGCGCCTGGGGCTGCTGTCGGTCATCTCCTGGATGGTGACGCTGACGAAACCACTGATTACCATCGCCGATTTCTCCTTTTCCGGGCGCGATCTCATCATGCTGCTCGGCGGGATCTTCTTATTGTTTAAGGCGACAACCGAGCTGCATGAACGGCTGGAAAACCGCCAGCACGATGCCGGTCACGGTAAAGGCTATGCCAGTTTCTGGGTAGTAGTATTGCAGATAGTGGTGCTGGATGCCGTCTTCTCCCTGGACGCCGTGATCACGGCGGTCGGGATGGTTAACCATCTGCCGGTGATGATGGCGGCGGTGGTGATCGCGATGATCTTGATGCTGCTGGCCTCCAAGCCGTTGACGCGCTTTGTCAACCAGCACCCGACGGTGGTCGTGCTCTGTCTGAGCTTCCTGTTGATGATTGGCCTGAGCCTGGTGGCGGAAGGGTTTGGCTTCCATATTCCGAAAGGGTATCTGTACGCGGCGATCGGTTTCTCGATCATTATCGAGTTCTTTAACCAGGTGGCGCGCCGTAACTTTGTTCGCCACCAGTCGACGCTGCCGCTGCGCGCTCGGACCGCGGATGCCATCCTGCGCCTGATGGGCGGCCGCAAACAGGCCTCCGTCAGCCATGACGCGGACAGTCCAGCCGCGGTGCCCGTTCCTGAGGGCGCCTTTGCGGAAGAAGAGCGCTACATGATTAACGGCGTGCTGACCCTGGCCCAGCGCTCCCTGCGCAGCATCATGACCCCGCGCGGAGAAATCAGCTGGGTCGACGCTGAGCAGAGTGAAGATGAAATTCGTCGTCAACTGCTGTCGTCGCCGCACAGCCTCTTCCCGGTCTGCCGCGGAGAGCTGGATGAAATCATTGGTATCGTGCGGGCGAAAGAGATGCTGGTGGCCCTGGAGTCGGGGGAAAACGTCGCGGCGCTGGCTTCTGCCTCCCCGGCAATTGTCGTTCCGGAAACGCTGGACCCGATCAATCTGCTCGGGGTCCTGCGTCGGGCGCGCGGCAGCTTCGTCATCGTAACTAATGAATTTGGCGTGGTGCAGGGGCTGGTCACGCCGCTGGACGTCCTTGAAGCCATCGCCGGTGAATTCCCGGACGCTGATGAGACGCCAGAGATTGTCATCGATGGCGATGGCTGGCTTATTAAGGGGTCGACCGACCTGCATGCGCTGCAGCAGGCGCTGGGGCTGGATCCACTGATCAATGACGATGAGGATATTGCCACCGTTGCCGGGCTGGTGATCTCCGCCAATGGTCATATCCCGCGCATTGGCGACGTCGTGTCGCTTCCGCCGCTGCATTTTACCGTAGTGGAAGCGAATGATTACCGGGTCGATCTGGTGCGCGCGGTGGTCACTCGTCCGCCGAGCGACGAAGAAGAGTAA

>IR1230_00271

GTGACGATTGCTATTGTAATAGGCACACATGGTTGGGCTGCAGAACAGCTGCTGAAAACAGCAGAAATGCTGTTGGGCGAGCAGGAAAACGTTGGCTGGATCGATTTCGTTCCGGGTGAAAATGCCGAAACGTTGATCGAGAAATACAACGCCCAGTTGGCAAAACTGGATACCAGTAAAGGCGTGCTATTTCTCGTCGATACATGGGGAGGCAGCCCTTTCAACGCTGCGAGCCGCATTGTCGTCGATAAAGAGCATTACGAAGTTATCGCCGGCGTCAATATCCCGATGCTGGTGGAAACCTTCATGGCCCGCGACGACGATCCTTCCTTTGATGAACTGGTCGCCTTAGCGGTTGAAACCGGCAGCGAAGGCGTGAAAGCGCTTAAAGCCAAACCGGTGGAAAAAGCGGCCCCTGCGCCCGCGCCTGCCGCAGCGCCAAAAGCCGCCGCACCGGCCAAACCCATGGGCCCGAACGACTACATGGTAATTGGCCTTGCGCGTATAGACGACCGTCTGATCCATGGTCAGGTCGCCACCCGCTGGACCAAAGAGACCAACGTTACCCGCATCATCGTTGTCAGTGATGAAGTGGCCGCGGATACCGTGCGTAAAACTCTGCTGACCCAGGTTGCGCCGCCGGGCGTCACCGCTCACGTGGTGGATGTGGCCAAAATGATCCGCGTCTACAACAACCCCAAGTATGCCGGACAGCGCGTTATGCTGCTGTTTACTAACCCGACCGACGTTGAACGTATCGTCGAGGGCGGCGTGAAAATCACCAGCGTCAACATCGGCGGTATGGCGTTCCGCCAGGGTAAAACCCAGGTTAACAACGCGATTTCAGTCGATGCCAAAGATATTGAGGCGTTTAAAAAGCTGAACGCCCGCGGTATCGAGCTGGAGGCACGTAAAGTGTCCACCGACCCGAAACTGAAAATGATGGATCTGATCGCTAAGGTTGATAAATAA

>IR1230_00272

ATGGAGATTACCCTTCTTCAGATTGTGCTGGTGTTCATCGTCGCGTGTATTGCGGGTATGGAGTCGGTACTTGATGAATTTCAGTTCCACCGTCCTCTGATCGCCTGTACGCTGATCGGCGCCGTTCTCGGCGATATGAAAACCGGTATTATCATCGGCGGTACGCTGGAGATGATAGCCCTGGGCTGGATGAACATCGGTGCGGCGGTAGCCCCTGATGCCGCGCTGGCGTCCATTATTTCCACCGTCCTGGTTATTGCCGGCCATCAGAGCATCGGCGCCGGTATCGCGCTGGCTATCCCGCTGGCGGCGGCAGGCCAGGTGCTGACCATCATCGTGCGCACCATCACCGTGGCCTTCCAGCACGCGGCGGATAAAGCGGCGGAGAATGGCAACCTGACCGCCCTGTCGTGGCTGCACGTCTCGTCCCTGTTCCTGCAGGCGATGCGTATCGCTATCCCGGCCGTTATCGTGGCGATTTCCGTCGGCACCAGCGAAGTTCAGGGCCTGCTGAATGCTATCCCGGAAGTGGTCACCAGCGGTCTGAACATCGCCGGCGGTATGATCGTGGTTGTCGGTTATGCAATGGTCATCAACATGATGCGCGCAGGCTATCTGATGCCGTTCTTCTACCTCGGCTTCGTCACCGCCGCCTTCACCAACTTCAACCTGGTGGCCCTGGGTGTGATTGGTGCGGTCATGGCAATCCTCTACATCCAGCTGAGCCCGAAATATAACCGCGTCGCCGGTGCGCCGGCTCAGGCGGCTGGTAACAACGATCTCGATAACGAACTGGACTAA

>IR1230_00273

ATGGTTGATATGACTAAAAATACCACCGAGAAAAAACTCACACAGAGTGATATTCGTGGCGTGTTCATTCGTTCTAACCTGTTCCAGGGTTCATGGAACTTCGAACGTATGCAGGCGCTGGGGTTCTGCTTCTCTATGGTTCCGGCGATTCGTCGTCTGTACCCGGAGAACAACGATGCGCGTAAGCAGGCGATTAAGCGTCACCTTGAGTTTTTCAACACCCACCCTTACGTTGCCGCTCCGGTACTGGGCGTAACGCTGGCGATGGAAGAGCAGCGCGCCAATGGCGCAGAGATTGACGATGGCGCTATCAACGGCATCAAAGTCGGTCTGATGGGACCGCTGGCCGGCGTTGGCGACCCGATCTTCTGGGGTACCGTGCGTCCGGTGTTTGCAGCCTTAGGCGCCGGGATCGCGATGAGCGGTAGCCTGCTCGGTCCTCTGCTGTTCTTTATCCTGTTCAACGCGGTGCGTCTGCTGACCCGTTACTACGGCGTCGCCTATGGTTATCGCAAAGGCGTGGATATCGTTAAAGATATGGGCGGCGGCTTCCTGCAGAAACTGACTGAGGGGGCGTCAATCCTCGGCCTGTTTGTCATGGGGGCGCTGGTTAACAAGTGGACGCATGTGAACATTCCGATGGTGGTGTCAAAAATCACCGGCTCTGACGGACAGGTTCACGTCACCACGGTGCAGACTATCCTTGATCAGCTGATGCCGGGTCTGGTGCCGCTGCTGTTGACCTTCGCCTGTATGTGGCTGCTGCGTAAGAAAGTTAACCCGCTGTGGATCATCGTTGGCTTCTTCGTCATCGGTATCGCCGGCTACGCGGTCGGCCTGCTGGGTCTGTAA

>IR1230_00274

ATGACCTTCACGGACCTGGTGATCATCCTGTTTATCCTTGCGCTACTGGCCTACGCGATATATGACCAGTTTATTATGCCGAGGCGAAACGGCCCGGTACTGCTGGCCATCCCCCTGCTTCGCCGCAGTCGCGTTGACGGCATGATCTTTGTCGGCCTCACCGCCATTCTGATCTATAACAACATCACCCAGCACGGCACGGCGATCACCACCTGGCTATTATCTGCGCTGGCGCTGATGGGGCTCTATTTATTCTGGATCCGTACGCCGAAAATCATTTTTAAACCGCACGGCTTTTTCTTCGCCAACGTGTGGATAGAATATCAGCGGATTAAAGAGATGAATTTATCCGAGGACGGTGTGCTGGTGATGCAATTAGAGCAACGGCGGCTGCTGATACGCGTACGAAATATCGACGATCTGGAGAAGATATACAAACTTCTCATTACAACTCAATGA

>IR1230_00275

ATGAATTTATCCGCTACCATTCTTCTCGCTTTCGGCATGTCCATGGACGCCTTCGCAGCCTCCATCGGCAAAGGCGCCACCCTGCATAAACCCAAATTTTCAGAGGCAGTACGCACCGGGCTGATTTTTGGCGCCATTGAAACCCTGACACCGCTGGTCGGCTGGGGGCTTGGCATGCTGGCCAGCCAGTTTATCCTCGAGTGGAATCACTGGATTGCCTTTATTTTACTGGTGTTTCTCGGCGGGCGAATGATCGTCGAAGGTTTTCGCGGCGACAGCGACGAGGCGTGCGAGGCGCCTCACCGACATGGTTTCTGGCTTCTGGTCACCACCGCCTTCGCCACCAGCCTTGACGCGATGGCCGTCGGCGTCGGTCTGGCCTTCCTGCAGGTCAGCATTGTGACCACCGCGCTGGCGATCGGCTGCGCCACCTTTATCATGTCGACGCTGGGGATGATGGTCGGTCGCTTTATCGGCCCGCTGCTTGGCAAACGAGCCGAAATTCTCGGCGGTATCGTGCTGATTGGCATCGGCAGCGAAATCCTCTGGAGCCATTTCGCCGGTTAA

>IR1230_00276

ATGTCATACAGTTGCCCCCTTTGTCACGCGCCGCTCAGCCGTAGCGACAACCACTATTCCTGCCCGCAGCGGCACCAGTTCGATCTGGCGAAAGAGGGGTACGTCAATCTGCTACCGGTGCAGTTCAAGCGGTCGCGCGATCCCGGCGATAGCGCCGAAATGATGCAGGCCCGGCGGGCGTTCCTCGACGCCGGACATTATCAGCCGCTACGGGATGCTATCGCTGAGCGGCTGCGACATTACGCCCCTACGGATTTACTGGATATCGGCTGTGGCGAGGGGTATTACACCCACGCGTTTGCCGCCATCGCCAGCCGCAGCTGGGGGCTGGACGTGTCGAAGCCGGCGATCCGCGCGGCGGCAAAACGCTACCCGCAGGTGAATTTTTGCGTGGCCTCCAGCCAGCGCCTGCCATTTTCCGACGCTAGCTTCGACGCGGTGGTGCGCATTTACGCGCCCTGCAACGCCGAAGAGCTGGCGCGGGTGGTGCGTCCCGGTGGTTGGGTGATCACCGCCACGCCGGGTCCCCGTCACTTACTGGAGCTGAAAGGGCTTATTTACGATGAAGTTCGCCTGCATGAGCTGAAGACGGAAGCGATGCCGGGGTTCCGCCTGGAGGCTCAGCAGCAGCTGGCTTATCCGATGATCCTGACGGGAAGTGAAGCGCAGGCCCTGCTGCAGATGACGCCGTTCGCATGGCGGGCTAAAGCCGAAGTGCACGCCGCGCTGCGTCAGCAGCCGACCTTTGGTTGCCAGACCGATTTTATGATCCACTGCTGGCAGCGCGAAGCGTAA

>IR1230_00277

GTGCTAAAGAAAAAAACGAAGTCCGCCGCCAGTTTCACCCCGATTCGCTTCGGGTTACTGTGTGTGGCCATTCTCGGTTGTCTGGGGCTGCTGCTGGTCCGCGTGGGCTGGCTACAAATTATCTCCCCCGATAACCTGGTAAAACAAGAAGATATGCGCTCCCTGCGCGAAGAACCCGTAGCGGTTGAGCGGGGGATGATCAGCGACCGGGAAGGACGGCCGCTGGCGGTGAGCGTTCCGGTCAGTGCTATCTGGATCGACCCGCAGACCACTATGGAGAAGGGCGGTGTGGGCTATGGGCCCCGCTGGCAGGCGATGGCCGAAGCGCTGCACCTCAACCTCGGAGAGCTGGCCCAGCGGGTACAGAGTCATCCCCACGCTCGCTTTCTCTATCTGGCGCGTCAGATCAATCCCGAGCAGGCAGAGTGGATTGATAAACTGCATCTGCCGGGCGTCTACCTGCGCGATGAATCGCGACGGTTCTACCCGGCGGGCCACGTGGCTGCCAATCTGCTGGGCTTTACCAATGTTGATAATCAGGGCATCGAAGGGGTGGAGAAAAGCTTTAACGCCCAGTTGACCGGTAAACCCGGGCGACGTCTGGTGCGTAAAGATAAACATGGTAATGTCATTGAGAACATTACCGAAGTGCCGCCGGTACCGGCGCATAATCTGCAGCTGAGTATTGATGAACGCCTGCAGACGGTGACGGAAGACGCCCTCGACAACGCCGTCCGCTGGAACAAAGCAGAGTCCGGGGCGGCGGTATTGATCAAAATTGATACCGGCGAGATCCTGGCGATGGCCAGCTATCCGGACTTCAATCCGAACAATCGCGATAGCGCGACGCTGGATGATTTTCGCAACCGGGCCATCAGCGACACCTTCGAGCCCGGGTCGACGGTCAAACCGCTGGTGATCATGACTGCGCTACAGCAGGGGATTGTCCAGCCAGACAGCGTGGTGGATACCCATCCGTTCGTCCTCGATGGCCATCGTATTCGCGATGTGGGCTATTATCCGGAGCTGAGCCTGACCGGGATCCTGCAGAAATCCAGCGATACCGGCGTGTCCCATCTCTCGCTCGCCATGCCGGTGCAGCATCTGATCGATACCTATAAAGCTTTCGGCTTTGGCGAACCGACGGGGCTGGGGTTGACCGGCGAAAGCGCCGGGCTGATGCCGCATCGCCGCTACTGGGGGCAACTGGATCGCGCCACCTTCGCCTTCGGCTATGGTTTGATGGTCACCCCGCTCCAGCTCGCCCACGTCTACGCCACCATCGGCGGCTTCGGCATTGCGCGACCACTGTCGATTACCCGCATCGATCCGCCGGTGATGGGCACGCGGGTGATGCCGGAGAGTATTGTGCATAGCGTGGAACATATGATGGAGAGCGTGGCGCTCCCGGGCGGGGGCGGTACCAAAGCGGCGGTACGCGACTACCGGGTGGCGGTAAAAACCGGCACGGCGAAGAAAATTGGCCCGGACGGCAAATATATCGATAAGTATGTGGCCTATACCGCCGGCGTGGCGCCCGCCAGTCGACCACAGTTCGCGCTGGTGGTGGTGATGAACGATCCGAGCAATGGTTCCTATTACGGCGGCGCTGTGTCCGCGCCGGTCTTCAGCCAGATCATGGGCGATGTCCTGCGGCTGGAGAACGTCATGCCGGACGGCATGCCGCAAGGGGCGGAGAATCTGATCGTAATGCACGATAGCCACCCACAGGGGCCGGCGCTGTAA

>IR1230_00278

ATGGCAAAGATTAAAGGTCAAGTTAAGTGGTTCAACGAGTCTAAAGGTTTTGGCTTCATTACTCCGGCTGATGGCAGCAAAGACGTGTTCGTACACTTCTCCGCTATCCAGGGTAACGGCTTCAAAACTCTGGCTGAAGGCCAGAACGTTGAGTTCGAAATTCAGGACGGCCAGAAAGGTCCGGCTGCAGTTAACGTAACTGCTATCTGA

>IR1230_00279

ATGAGTGAGTTACTAAATCCTGGGATTTTAAATCTGGCATCGCTGGCTGTGTCCGTGGCGCTGCTGCTTGTCGGTCTGTTGTTATGGTTCTTCGTCAACCGCGCCAGTTCGCGGGCGAATGAGCAGATAGAGCTGCTGCAAGCGCTGCTGGATCAGCAAAAGCGGCAGAATGCGCTGTTACGTCGCCTGTGCGAAGCTAATGCGCCAGAGAAAGAGGATGTGGCTGAGCCGACCGTCGCCGGCAAGGCGAAAGGGGAGGATGAGTTTATCCGCCTGGTAGCTGAACGTTAA

>IR1230_00280

ATGCGATTAATCATTCGTACTATTATCTTGGTGGCTATCTTATGGATTGGCGTATTATTGAGCGGTTATGGGGTGTTATTCCACAGTGAGGAAAACGTCGGCGGTCTGGGTCTTAAGTGCCAATACCTCACCGCCCGCGGAGTCAGCACCGCACTTTATGTTCATTCCGACAGCGGAGTGATCGGCGTCAGCAGTTGCCCTCTGCTGCGTAAAAGCACAACCGTGGTTGATAACGGCTAA

>IR1230_00281

ATGGCAGGTGCAGATTTGGATAAACAGCCAGATTCTGTCTCTTCGGTATTGAAGGTTTTCGGCATCCTACAGGCGCTGGGTGAAGAGCGTGAAATTGGCATTACCGAACTGTCCCAGCGCGTCATGATGTCGAAAAGCACCGTTTATCGCTTTTTACAGACCATGAAATCACTGGGCTATGTGGCACAGGAAGGAGAGTCTGAGAAATACTCTCTCACCCTCAAGCTGTTCGAACTGGGCGCGCGCGCGCTGCAGAACGTCGACCTGGTGCGCAGCGCCGATATTCAGATGCGCGAACTTTCGCGACTGACGAAAGAGACCATCCATCTTGGGGCGCTGGATGAAGACAGTATCGTCTACATCCATAAAATTGACTCGATGTATAACCTGCGGATGTACTCGCGTATTGGCCGCCGCAACCCGCTGTACAGTACCGCGATTGGCAAGGTGCTGCTGGCATGGCGCGATCGCAGCGAAGTGGAGCAGATCCTCGACGGCGTGGAGTATAAGCGCAGCACCGAGCGGACCATTACCAGCACCGAGGAGTTGCTAAAAGTGCTGGATGGCGTTCGCGAGCAGGGCTATGGCGAGGATAATGAGGAACAGGAAGAGGGGCTGCGCTGCATTGGCGTGCCGGTGTTTGACCGTTTCGGCGTGGTCATCGCCGGGCTGAGCATCTCTTTCCCAACGCTACGTTTCTCTGAAGAACGCTTGCATGAATACGTGGCGATGCTCCATCAGGCGGCGCGTAAGATTTCTGAACAAATGGGATACAACGACTATCCGTTCTGA

>IR1230_00282

ATGGATAAAAATTCTTCTGATGGCGTGCCGTTGCCGCAACGCTATGGCGCCATTCTGACCATCGTGCTTGGGCTGACCATGGCCGTGCTCGACGGGGCTATCGCCAACGTCGCGCTGCCGACTATCGCCAGCGATCTCAACGCCTCACCGGCGGCTTCAATCTGGATCGTTAACGCCTACCAAATCGCCATTGTCATCGCCCTGCTGCCGCTCTCCTTCCTCGGCGACATGGTCGGCTATCGGCGTATCTATAAGATAGGCCTGGTGGTGTTTATCTTTACCTCGCTGGCCTGCGCGCTGTCGCGTAGCCTCGAAATGCTAACCTTCGCCCGCGTCGCGCAGGGATTGGGCGGCGCCGCGCTGATGAGCGTCAACACCGCGCTGATCCGTCTGATTTATCCGCAGCGCTTTCTTGGTCGCGGCATGGGCATTAACTCCTTTGTCGTTGCCGTCTCTTCGGCGGCAGGCCCGACTATCGCCGCAGCGATCCTCTCCCTCGCCTCATGGCAATGGCTGTTTTTAATTAACGTGCCTCTCGGCATCGTCGCCTTTGTTCTGGCCATGCGCTTTCTGCCGCCCAACAGCGCGCGCAGCAAAATCATCCGCTTCGATCTGCCGAGCGCCATTATGAACGCCCTTACCTTCGGGCTGCTGATCACCGCCCTGAGCGGTTTCGCCCAGGGGCAGTCCACTCAGCTGGTGCTGGCGGAGGTCGCCGCTATGCTGGTGGTGGGGTTCTTCTTCGTTCGCCGCCAGCTCACGATGCCCGTCCCCCTGCTGCCGGTCGACCTGCTGCGTATCCCACTCTTTTCTCTCTCTATCTGCACTTCCATCTGCTCCTTCTGCGCGCAGATGCTGGCGATGGTCTCTCTGCCCTTTTTTCTGCAGTCGATGATGGGGCGCAGCGAAGTGGAGACCGGTCTACTGCTAACGCCGTGGCCGTTAGCGACCATGGTGATGGCGCCGCTGGCCGGCTATTTGATCGAGAAATGTCATGCCGGGCTGCTGGGGGCTATCGGTTTGCTGATTATGGCCTGCGGCCTGTTCGGCCTGGCGCTGCTGCCCTCGTCGCCCTCCGATCTGGATATCATCTGGCGCATGGCGCTGTGCGGCGCCGGCTTCGGGCTGTTTCAGTCGCCGAACAACCATACCATCGTCGCCTCGGCTCCGAGCCATCGTAGCGGCGGCGCCAGCGGCATGCTGGGCACCGCTCGCCTGCTCGGACAGAGCACCGGCGCGGCGCTGGTGGCGCTGCTATTCAATTTGCTCGGTAACAACGGCACCCACACCGCCCTGCTGCTGGCCGGCACCCTGGCTATCGTCGCCGCGCTAATTAGCGGTCTGCGGGTCACTCAGCCGCGCGCAGCCTGA

>IR1230_00283

ATGATGCGAATCGCGCTTTTCCTGCTGACGAACCTGGCAGTGATGGTCGTGTTCGGGCTGGTGTTAAGCCTCACGGGGATCCAATCCAGCAGCATGACCGGTCTTCTGATTATGGCCCTGCTGTTCGGCTTCGGTGGTTCTATCGTTTCGCTGATGATGTCGAAGTGGATGGCGCTGAAGTCAGTGGGTGGGGAAGTGATCGAGCAGCCGCGCAACGAAACGGAACGCTGGCTGATGAATACCGTTGCGCAGCAGGCGCAGCAGGTAGGTATTGCCATGCCGCAGGTAGCTATCTATCACGCGCCTGACATTAACGCCTTCGCCACCGGCGCGCGTCGTGACGCCTCGCTGGTCGCCGTCAGCACCGGGCTGTTGCAAAACATGAGCCGTGATGAAGCGGAAGCGGTCATTGCCCATGAAATCAGCCACATCGCCAATGGCGATATGGTTACCATGACCCTGATCCAGGGGGTGGTGAACACCTTCGTTATCTTTATCTCCCGCGTTATCGCGCAGATTGCGGCCGGTTTCCTCGGCGGCAACCGGGAAGATGAAGGGGAGAGCAGCAATGGCAACCCGCTGATCTACTTCGCGGTCGCCACGGTGCTTGAGCTGGTGTTCGGTATTCTGGCCAGCATAATCACCATGTGGTTCTCGCGTTACCGTGAGTTCCATGCTGACGCCGGCTCGGCGCGTCTGGTAGGCCGCGAGAAGATGATTGCAGCCCTGCAGCGTCTGAAAACCAGCTATGAGCCGCAGGAAGCCAGCAGCATGATGGCGTTCTGCATTAACGGTAAAGCGAAATCCATGAGCGAACTGTTTATGACTCACCCGCCGCTGGATAAGCGTATTGAAGCCCTGCGTAGCGGTGAATATCTGAAATAA

>IR1230_00284

ATGAACACATTTTTTAAAATCACCGCGCTTGCGGGCCTGCTGGCAATAGCAGGCCATGCATTCGCTGTTGATGACATAACCCGCGCAGATCAAATCCCCGTGCTGAAAGAAGAGCCGCAGCACGCGACGGTAAGCGAGCGCGTGACGTCGCGCTTTACCCGCTCTCATTACCGTCAGTTTGATCTCGACAACGCCTTCTCGGCAAAGATTTTTGACCGTTACCTGAACCTGCTGGACTACAGCCACAATGTGCTGCTGGCCAGCGATGTGGCGAAGTTTGCGGCTAAAAAAGACCAAATTGGCGATGAGCTGCGCAGCGGGAAACTGGACGTCTTTTACGACCTCTACAACCTGGGGCAACAGCGCCGCTTCGAGCGCTATCAGTATGCGCTGAAGGTGCTTGAACGTCCGATGGACTTTACCGGCAATGATAATTTCAACCTTGACCGCTCCAAAGCGCCGTGGCCAAAAGACGAGGCCGAGCTGAACAAGCTGTGGGACGCTAAGGTTAAGTTTGACCAGTTAAGCCTGAAGCTGGCGGGTAAAGACGATAAAGAGATCCGCGACACCTTAACCCGTCGCTATAAGTTTGCTATACGTCGCCTGGCGCAAACCAACAGCGAAGATGTCTTCTCGCTGGCGATGACCGCCTTCGCTCGCGAAATCGATCCGCACACAAATTATCTGTCGCCGCGTAATACCGAGCAGTTCAATACCGAAATGAGCCTCTCGCTCGAGGGGATCGGCGCCGTGCTGCAGATGGATGATGACTATACCGTCATTAATTCGCTGGTGGCGGGCGGTCCAGCCGCGAAAAGCAAAGCGATCAGCGTCGGCGATCGCATCGTTGGCGTTGGCCAGACCGGGAAGTCGATGGTTGATGTCATCGGCTGGCGTCTGGATGACGTCGTGGCGCTGATTAAAGGGCCGAAGGGCAGCAAGGTTCGTCTTGAAATCCTGCCGGCCGGTAAAGGAGCCAAGACGCGTATTGTAACGCTGACCCGCGAGCGCATTCGTCTGGAAGACCGTGCGGTTAAGATGTCGGTGAAAACCGTCGGGAAAGAAAAAGTCGGCGTGCTGGATATCCCAGGCTTTTACGTTGGCCTGACCGATGACGTGAAAGTGCAGCTGCAGAAGCTGGAAAAACAGAACGTCAGCAGCATCATCATTGACCTGCGCAGCAACGGCGGCGGAGCGCTGACAGAGGCGGTATCGCTCTCGGGTCTGTTTATTCCGTCCGGTCCGGTGGTGCAGGTGCGGGATAATAATGGCAAAGTGCGCGAAGACAGCGACAACGATGGCGTGGTTTATTACAAAGGGCCGCTGGTGGTGCTGGTTGACCGCTTTAGCGCCTCGGCGTCTGAGATCTTCGCCGCAGCGATGCAGGACTATGGCCGCGCGCTGATCGTTGGCGAACCGACCTTCGGTAAAGGGACGGTACAGCAGTATCGCTCGTTGAATCGTATTTACGATCAAATGCTGCGTCCGGAGTGGCCGGCGTTGGGTTCCGTCCAGTACACCATCCAGAAGTTCTATCGTATTAACGGCGGCAGTACGCAGCGCAAAGGCGTGACGCCGGATATCATGATGCCAACCGGCAATGAAGATCGCGAAACCGGCGAGCAGTATGAAGACAACGCGCTGCCGTGGGATAGCATCAACGCGGCCACCTATGTGAAGTCCGGGGATCTGACCCCGTTTGGACCGGAGCTGCTCAAGCGCCACGACGAGCGTATTGCGCAGGATCCTGAATTCCAGTACATCATGAAGGATATTGCCCGTTATAACGCGATGAAGGACAAACGTAACATCGTCTCTCTGAACTACGCCCAGCGTGAGAAAGAGAACGAAGAAGATGATGCGATTCGTCTGGCGCGGATTAACGATCGCCTGAAACGCGAAGGCAAGCCGCCGCTGAAAAAACTGGACGATCTGCCGAAGGATTACCAGGAGCCGGATCCGTATCTTGATGAAACGGTCCATATCGCGGTAGACCTGGCGCATCTGGAAAAAGCGCGCCCGGCGGTGGAACCGCCGGCCAGCAAATAA

>IR1230_00285

ATGGAAAATCAACCTAAGTTGAATAGCAGTAAAGAAGTTATCGCCTTTCTGGCCGAACGTTTCCCGCAGTGCTTTAGCGCTGAAGGTGAAGCGCGCCCCCTGAAAATCGGAATTTTTCAGGATCTCGTTGAGCGAGTGGGTGGTGAGATGAATCTCAGCAAGACCCAGCTTCGCGCTGCATTACGTCTCTACACGTCCAGCTGGCGTTATCTGTACGGCGTAAAAGCCGGCGCTATTCGCGTTGACCTGGATGGCAATCCGTGCGGCGAACTGGAAGAGCAGCACATTGCCCATGCGCGTCAGCAGCTGGAAGAAGCGAAAGCTCGCGTTCAGGCGCAGCGCGCAGCCCAGCAGGCGAAAAAACGCGAAGCCGCTGCGGCCGCAGGCCAGCAGGATGAAGGCGTTCGCCGCGAGCGTAAACCGCGTCCTCAGCAGCCGCGTCGTAAAGAAGGCGCTGAACAGCGTAAACCGCGTCCTGTTGCGGCCAAAGCCCCGCGTGAAGAGCGTCTTACTCCGGTATCGGATGTTTCCGTTCTGACCGTCGGCCAGGCGCTGAAGGTAAAAGCAGGCAATAACGCCATGGACGCCACCGTTCTGGAAATCACCAAAGATGGCGTTCGTGTACAGCTGACTTCTGGTATGTCAATGATTGTACGCGCAGAACACCTGGTGTTCTGA

>IR1230_00286

ATGCTTATTTTAGTAGGCTGTACGTATCAGGCAGTAAGACAACAAAGTGAAGATATAATGAGCAAAACAGAATTTTACGCGGATCTGAACCGCGATTTCCAGGCATTAATGGCAGGTGAAACCAGTTTTCTGGCGATGATCGCCAATACCAGCGCGCTGCTGTTCGAGCGGCTGAGCGAGGTCAACTGGGCCGGTTTCTACCTTCTGGAAGGCGATACCCTAGTGTTGGGACCGTTCCAGGGCAAACTGGCCTGCGTGCGGATCCCGGTCGGGCGAGGCGTATGCGGCGCGGCGGTGGCGCAGGCTCAGGTCCAGCGCGTCGAGGACGTCCACGCCTTTGACGGCCACATTGCCTGCGATGCCGCCAGTAATTCGGAAATTGTCTTCCCTCTGCGCGTGAATGGCCAGATTATCGGCGTGCTGGATATCGATAGTCCGGCTTACGGGCGCTTCACCGCTGAAGATGAGCAGGGGTTGCGCACGCTGGTCGAGCATCTGGAAAAACTCATTGCGGCCACGGATTATCAAAAAATCTTTACCCGTGTCGTGGGATAA

>IR1230_00287

ATGCCGATAAAAACACCCACCCTCAAACCTGCGAAAAAGATGGTCGTGCATACTGTCAGCACGCCGCTTCCCTATGCGCATTATCAGCGCTGTACGCAGTGTGACATGCTCTTCCGCTTACCGGTGCTCAAACGCAACCAAAGCGCCTGGTGTCCGCGCTGTAACGCGAAGGTGCGCGATGGCCGCGACTGGTCGCTGACCCGCCTGGGCAGCATGGCCCTCGCGATGCTGTTGCTCATGCCTTTCGCCTGGTCGGAGCCTCTGCTGCGCCTGCATCTGTTGGGCGTACGCATCGACGCCAACGTCCTGCAGGGGATCTGGCAGATGACCGCGCAGGGCGATCCGATTACCGCCGCCATGGTGCTGTTTTGCGCGGTGGTCGCCCCGGTGCTGCTGGTGGTCTCCATCAGCTATCTCTGGCTGGGCAATGTGCTGGGGATGAACCTGCGGCCGGTGCTGCTGATGCTCGGCAAGCTTAAAGAGTGGGTGATGCTGGATATCTATCTGGTGGGCATCGGCGTCGCCTCCATCAAGGTGCAGGATTACGCCTTCCTGCAGCCGGGCATCGGTCTGGTGGCGTTTATCTCCCTGACGTTGTTGAGCATCCTGACGCTTATTCATATGAACGTCGAAGAGCTATGGGAGCGTTTCTATCCCGAGCGTCCGGCGACCCGCGCCGATAATAATCTGCAGGTGTGCACCGGCTGCCATTACACCGGCTATCGCGACGCGCGCGGCCGCTGCCGACGCTGCCACACGCCGCTGCATCATCGCCGACCGCAGAGCCTGCAGCGCAGCTGGGCGGCGCTGATTGCCTCGCTGGTTTTCCTCTTTCCGGCCAACCTGTTGCCAATCTCCATCATCTACGTCAACGGCGCGCGCCAGGACGACACGATCCTCTCGGGGATCATCTCCCTGGCCAGCAGCAATATCGCCATTGCCGGGGTCGTGTTTATCGCCAGTATTTTGGTGCCATTCACCAAAGTTATAGTGTTATTTACCCTGCTGCTCAGCATACAATTCAAGTGCGAGCAGGGATTGCGCACCCGCATCCTGCTGCTGCGGCTAATCACCTGGATTGGCCGTTGGTCGATGCTGGATCTGTTTGTTATCTCACTCACCATGTCGCTAATAAATCGCGATCAGCTCCTCGCCTTCACCATGGGGCCGGCTGCGGTTTACTTTGGCGGCGCGGTAATTTTAACTATTCTTGCAGTTGAATGGCTGGACAGCCGCTTACTTTGGGACGCACATGAGTCAGGAAACGCCCGCTTCGCAGACTGA

>IR1230_00288

ATGAGTCAGGAAACGCCCGCTTCGCAGACTGAAGCCAGAATAAAAACCAAACGCCGTATTTCGCCATTCTGGCTGCTGCCGGTCATCGCGTTGCTGATTGCCGCCTGGCTAATCTGGACCAGCTTTGACGATCGCGGATCGACGATTACGATTGATTTCCAGTCCGCGAACGGCATCGTGCCGGGACGGACGCCGATCCGCTATCAGGGGGTTGAAGTCGGCACCGTCCAGGATATTAGCCTCAGTAAAGATCTGAGTAAGATTGAGGTTTCGGCCAGCATCAAACGCGATATGAAAGATGCGCTGCGCAAAGAGACCCAGTTCTGGCTGGTCACGCCGAAAGCCTCTCTGGCCGGCGTATCGGGCCTCGATGCGCTGGTGGGCGGGAACTACATCGGCATGATGCCGGGCAAGGGTGAACCCGAAGATCACTTTGTCGCCCTCGACACCCAACCTAAATACCGCATCAACAACGGCGAGCTAATGATCCACCTGCAGGCGCCGGATCTTGGCTCTCTGAACAGCGGCTCGCTGGTCTACTTCCGAAAAATCCCGGTCGGGCGGGTGTATGACTATTCCCTGAATGCCAATAATCAGGGGGTGACCATCGATGTGCTAATCGAGCGCCGGTTTACCAATCTGGTGAAAAAAGGCAGCCGCTTCTGGAACGTCTCAGGCGTCAAGGCCGACGTCGGCCTGAGCGGGGCCAAAGTGCAACTGGAGAACCTGTCCGCGCTGGTGAACGGCGCCATCGCCTTTGACTCCCCCGCCGATTCGCATGTCGCCTCGCAAAACGATGAATATCACCTGTATGAAGATCTGGCGCATAGCCAGCGCGGCGTGGTGGTGACCCTCGATCTGCCTGATGGCGATGGACTGAAAGCGGGCTCGACGCCGCTCATGTATCAGGGGCTGGAGGTGGGCCAGTTAAGCAAACTGAATCTAAACCCCGGCGGCAAAGTTACTGGCGAGATGACCGTCGACCCGAGCGTGGTGACGCTGCTGCGGGAGAAAACGCTGATTCAGATGAAGAAGCCGAAGCTCTCCCTCGATAACCCCAGCATTAGCACCCTGCTCACCGGCAATACCTTTGAGCTGGTGCCCGGCGAAGGCGAGCCACGCAATCACTTCTCGGTAATGCCGGCGGATAAAGCGCTGCTGGATGAGCCCAACGTCGCCACCGTTACCCTCTCGGCGCCGGAGAGCTACGGCATCGACGGCGGTCAGCCGCTGGTGCTGCACGGAGTAAAAGTCGGCCAGGTGCTGGAGCGTAAACTGACGGCCAAAGGGGTAACCTTTCAGGTTGCCATTGATCCTGAATACCGCGATCTAATTCACGGCGACAGCAAATTTGTGGTTAACAGTCGTCTGGACGTCAAAGTGGGGCTCGACGGCGTGCAGGTGCTGGGCGCCAGCGCCAGCGAATGGGTGAACGGCGGGATCAGGGTGATCCCGGGCGAAAAAGGCAAAATGCAAAGCAGCTATCCGCTGTACGCTAACCTTGAAAAGGCGCAGGAAAATAGCCTGAGCGAAGTGCCGACCACTACCCTGAGCCTGTCTGCTGAGACGCTGCCTGACGTTCAGGCCGGCTCGGTGGTGCTGTATCGTAAATTCGCCGTCGGCGAGATCATCGCCGTCAAACCGCGCAAGGATGCATTCGATATCGATCTGCATATCAAGCCGGAATACCGCTACCTGCTGACCAACAACAGCGTCTTCTGGGCGGAAGGCGGTGCGAAGGTCAAGCTTGACGGTAACGGACTCACCGTGCAGGCCTCGCCGCTGGCGCGCGCGATCAAAGGGGCGATCAGTTTCGATAACCTCAATGGCAGCAGCGCCGGAGCACGGCTGAATAACAAACGTATCCTTTATGCATCTGAAACGGCGGCGCGCGCCGTGGGCGGCCAAATCACCCTGCACGCGTACGACGCCGGGAAGATGGCGGCCGGGATGCCGATTCGTTATCTCGGCATCGACATCGGTCAAATCCAGTCGCTGGAGCTAATCACCGCCAAAAACGAAGTGCAGGCGAAAGCGGTGCTCTATCCGGAATACGTCGGCACCTTCGCCCGCGCCGGGACCCGCTTCTCGGTGATCACTCCGCAAATCTCCGCCGCGGGCGTGGAACATCTCGACACCCTGTTCCAGGCCTACATCAACGTTGAGCCCGGCCGCGGCCCGGCACGACGCGATTTTGAGATCCAGGATACGACGATCAGCGATTCGCGTTATATCGACGGCCTGAACATCGTGGTGGAAGCGCCGGAGGCGGGATCGCTTGGCATCGGTACGCCGGTCCTGTTCCGCGGACTGGAAGTCGGGACGGTTACCGGACTGTCGCTGGGGTCGATGTCTGACCGGGTGATGGTTAAGCTGCGCATCAGTAAGCGCTACCAGTACCTGGTACGTAATAACTCGGTGTTCTGGCTGGCCTCGGGTTACAGCCTCGACTTCGGCCTGATTGGCGGGGTGGTGAAAACCGGTACCTTCAATCAGTTTATCCGCGGCGGGATCGCCTTTGCCACGCCGCCGGGCACGCCGCTGGCGCCGAAGGCCCAGGATGGCAAGCACTTCCTGCTGCAGGAGAGCGAGCCGAAAGAGTGGCGGGAGTGGGGCACCGCTCTGCCGCAGTAA

>IR1230_00289

ATGCGCGCGGCGATGCCAGCGCATCTCTCTTTCGACGACTTTATCGCCGCCTGTCAGCGCCCGCTACGCCGCAGTATTCGCGTCAATACGCTGAAGATTAGCGTCGATGATTTCCTCTCTCTGGTTGCCCCCTACGGCTGGCAGCTCGCGCCGGTGCCCTGGTGCGCAGAGGGATTCTGGATCGAGCGAGAGGATGACGACGCGCTGCCGCTGGGCAGTACCGCCGAACATCTCAGCGGGCTGTTCTATATTCAGGAAGCCAGTTCGATGCTGCCGGTCGCCGCCCTGTTCGCCGATAACCGGCAGCCCGAACGGGTGATGGACGTTGCCGCCGCCCCGGGATCGAAAACCACCCAAATTGCAGCCCGAATGGGCAACGCGGGCGGCATTCTCGCCAATGAATTCTCCGCCAGCCGGGTGAAGGTGCTGCATGCCAACATCAGCCGCTGCGGCATCAGCAACGTCGCTCTGACCCACTTCGACGGCCGGGTTTTTGGCGCGGCCTTGCCCGAAACCTTCGATGCCATCCTGCTTGACGCCCCCTGCTCCGGCGAAGGGGTTGTACGTAAAGACGCCGACGCGCTGAAAAACTGGTCGCCGGAGAGCAACCTCGACATCGCCGCCACTCAGCGCGAGCTTATCGACAGCGCCTTCCATGCTCTGCGCCCTGGCGGGACGCTGGTGTATTCCACCTGCACCCTGAATCGTGAAGAAAATCAATCGGTCATTGAGTGGCTGCTGTCCCGCTATCCGCAAGCGGTGGAGATCCTGCCGCTGGGAGAGTTGTTTCCCGGCGCAGCCGACGCCCTAACCGCGGAAGGTTTTCTGCACGTTTTTCCGCAAATTTACGATTGCGAAGGCTTCTTTGTCGCGAGGTTGCGCAAAACTGCGGCGATCGATCCGCTCCCGGCGCCAGGCTATAAGGTCGGTAAATTCCCGTTTACGCCGCTGAAGGACCGGGAGGCCGCCGCGGTAACTGCCGCCGCCAGGGCTGTTGGCCTGGAGTGGGACGCAGGCCATACGCTGTGGCAGCGGGATAAAGAGCTGTGGCTGTTTCCGCTGGCTTTGGAGCCCCTGTTCGGCAAGGTGCGCTTTTCTCGCATCGGCGTGCGCCTGGCGGAACTGCACAATAAGGGGTACCGCTGGCAGCATGAGGCGGTTATCGCCTTTGCCGCGCCGCAGCGCGCCTTCGAGCTGTCGCAGGAGGAAGCGGAGGAATGGTATCGCGGGCGCGACGTCTATCCGCAAACCGCGCCTGGGCAGGATGAAACCATTGTGACCTTCCAGGGCGTTCCGCTGGGCCTGGCGAAGCGCGTCGGCTCCCGACTGAAGAACAGCTATCCGCGCGAACTGGTGCGCGATGGAAAACTTTTTGCCGGCAAGGTGTGA

>IR1230_00290

ATGAGTAAAACCAATGTCCGCATCGGCGCGTTCGAAATAGACGATGCCGAACTGCACGGTGAGCATCAGGGTGAGCGAACGTTAAGCATACCCTGCAAATCCGATCCAGACTTATGTATGCAGCTTGATGCCTGGGATGCCGACACCAGCGTGCCCGCGATTCTCAATGGCGAACATTCCGTTCTCTACCGTAAACATTACGACCGCCAGTCCGATGCCTGGGTCATGCGCCTTGCCTGA

>IR1230_00291

ATGTTTGCCTTAGTGTTGTTTATTTGCTATCTGGATGGCGGCTGCGAGGACATTGTGGTTGACGTCTACCGCGAGGAAACGCAGTGCTTAGTGGCGATGGAGGATCAGCGGATCCGCCACGGTGGCTGCTACCCGATAGAAGATTTTATTGATGGCTTCTGGCAACCCGCCAGCGAATACAGCGACTTTTAG

>IR1230_00292

ATGTATCAACGTATTAACGGCAGCGACTGGCGTAATATCTGGCTGATGGGCGATCTGCATGGCTGCTTTGCGCTGCTGATGAATCGCCTGCGTCAGTTGCGTTTTGATCCCTGGGCCGATCTGCTGATCTCGGTGGGCGATCTGATCGATCGCGGGCCGCAGAGCGCCGATTGTCTTGGCCTGTTGCGCTGTCGCTGGTTCAGGGCCGTTCGGGGGAATCATGAGCAGATGGCGCTGGAGGCGCTGGAGAGCGGAGATATGCGGCTCTGGCAGATGAACGGTGGCGACTGGTACGTCAAGGGTGATGCGCGGCAGCGGGCCGACGTCGATCGGCTGCTGGCGCACTGTCGGCGGCTACCGTTGATTATTGAAGTGGAATGCGGGAAAGCGCGGCATGTGATCGCCCACGCCGATTACCCGGCGCCGGTCTATCGCTGGCAGCAGCCGGTGGATCCCCAACGGGTACTATGGAGCCGCCATCGTCTGAGTGAACACCTGGCGGGGCGCCATGGCGCTATTGCCGGCGCGGACCATTTCTGGTTTGGCCATACGCCGCTTCAGGCGCGTTATGACCACGATAATCAACACTATATTGATACGGGCGCTGTGTTTGGTGGGACGCTGACGCTGGTGGCGCTGCAGTCGGCAGGCTAA

>IR1230_00293

TTGTCAGTGGCTCGCTTTAAAATAGGGGGAAATCACAACGAGGTGTTTATCGTGACAGACAATAGCCTGCATTCATCCGGCGTGCTAGCGCCGCGCTCGCATCTTATTGCCGATCTGGTGATGGGCAGATTGACACCTGCCCCTATCTGGCGGCAAAAAAATTACCGCTTTAAGTTTTTGCTGCGCACGGCCTTATTTTATAACGCCACCAGAGCGATGCTGGAAGCGCTCAGCGCTCGGGATGATTTTAATCAGCTGCTGGCCGCGCAGGCTACGCTGCCGGGCAAAGTACACCGTCAATATCTGACCCGCGATCTCAACGCCTGGCAGCGGGCGGTGGCGGTAATCAACCATTATCGTTATATCGACACCCTACGCGGATCGCGGCTGGCGCACGCTATGACCGCGGTGTCTGAGGTCCCGCTGCTGACGTTAAACGGAAAAGAGGACCGCCGGTTTACTCTTTATGCCTCCTCGGCAGGCAAAGCCGAACGTGAGGGTGAAACCACTCTGTGGCTGCGCGACAGCGACCACACCCTGCTGGCCAGCGCCACCTTCAGCGTAACCCGTGATCACGATGCCTGGCAGCTGGTTATCGGCGGTCTGCAGGGGCCGCGACGTCACGTTTCTCATGAGGTGATTAAGCAGGCGACTCGCGCCTGCTACGGCCTGTTTCCGAAGCGCCTGCTGCTGGAGTTTATCTGGCAGCTGGCCGCGCGCAGCCAAATCGCGGCTATTTACGGCGTCAGCGATAATGGTCACGTGTTCCGCGCCCTGCGCTATCGCCTGAGCAAAGGCCGCCATTTCCACGCCAGCTACGACGAGTTCTGGCAGTCTATCGACGGCCAGCCGGAAAGCCCCTGGCGCTGGCGGCTACCGCTATGTCTGGAAAGAAAATCGCTGGAGAGTATCGCCAGTAAAAAACGGGCTGAATATCGGCGGCGATTCCAGCTGCTGGATCAGCTGACGGAGCAGGTGGCAATATTGACGCAGCGGCATGCCGGTGACTAA

>IR1230_00294

ATGTTGGCCCGCCAGTTAGCCTGCACGCCGGGCCTGTTTAGGTGCAGGTTTTTTCTTTTCTCCGCCCTCGTTTACGGTTCCACCGTTGCCACCTCCCCTTCCACAGGATGCGATTTATGTGTAAATGAATTGTGTATGCTAAAAACGGTGTGCTATAACGGGACATCCAGAGGCCAGCGGGTGTTTGACTGA

>IR1230_00295

ATGAAAAAAATTGTTCTGACCATGTTGTTACTGGCAAGCTCCGGGGCTGCTCTGGCAGCGCCACAAATTATCACCGTGAGCCGTTTTGAGGTGGGGAAGGAGAGCTGGGCGTTCAACCGGGAAGAGGTTATGTTGACCTGTCGACCGGGTAACGCGCTTTACGCCATCAACCCGAGCACGCTGGTTCAGTATCCGCTCAATGAGGTGGCTGAGCAGCAGGTCAAAGCCGGCAAAACCACCGCGCAGCCGATCTCAGTGATCCAGATCGACGATCCGCAGCATCCGGGACAAAAAATGAGCCTCGCGCCCTTTATCGAGCGAGCGCAGAAGCTCTGCTAA

>IR1230_00296

ATGCTGACCGGGCTCTATATTACCCTGCGCTTTGGGCACTTTATCTCCCTGATGCTGGCTTTTGGCTGCGTGCTGTATGGCGCCTGGTGGGCGCCGGTACCGCTGAGACGCGTGCTGATGCTGCGCTTTTATCCACTACTGCGCCCGCTGCTGCTGATCGGCGCGATCTCTACGCTGGCGCTGTATCTCCTGCAGGGAGGAATGATGGGCGAAGGCTGGACGGATGTCTGGCGACCCGCCGTCTGGCAGGCGGTGGCCGGGACCCGCTTTGGTGGAGTGTGGATCTGGCAGATCCTACTGGCGTGGATCGCACTTGCGGTAGTGTGGATCCGTCCCCGTCACGGCGCCCGCCAGCTGGTGGCCCTGCTGGCGGCACAGCTGCTGCTTTCCGCCGGGGTAGGGCACGCCGCTATGCACGATGGTCTCACCGGCGTGCTGCAGCGGACAAACCACGCTGTGCATCTCTTCTGCGTCGCCAGCTGGTTCGGCGGTCTGTTGCCCTTTATCTATTGCCTGCGCCTGGCGCAGGGGCGCTGGCGGCCGGCCGCGGTCTATACCATGGCGCGCTTTTCACGTTACGGGCATCTGGCCGTCACCGGCACGATTGCCAGCGGAGCGCTCAATGCGCTGTTGATCCAGGGAGGATTGATTGGCGCATCGCCGTGGGGACGCATGTTGTTGATCAAATGTGCGCTGGTCGCCGGGATGGTGGTAATTGCGTTAGTGAACAGGTATGTTCTGGTACCGCGCATGTCGGCAAGCGGTTCGCAGGCGGAAAGCCTGATCCTGCGAACCACGCAGGCTGAAATAGGGCTGGGCGCGCTGGCGCTGCTGGCCGTCAGTCTGTTTGCCACCTGGGAACCTTATTGA

>IR1230_00297

ATGCGACTTCTTGCTGGACGCGCGTTGCGCCTCTCCGTTGCGCTTGCCGGGATGTTAACCGCGGCGGGCGCCTTCGCCCACGCTCACCTGCAGCAGCAGATCCCGACCGCCGGGGCGCAGCTTTCAGCTTCCCCACAGACGCTGACGCTCAGTTTTTCCGAAGGTATTGAACCCGCATTCAGCGGCGTGACCGTCACCGGTCCGCAGCAGCATGCGGTGGCTACCGGAAAACTGACCCGCAGCGCCGATAATCCGGCCGAGGTCACGTTACCTTTGGCGGAAGCCTTGCCGCCGGGAGAGTACACCGTGGCATGGCATGTGGTCTCGGTGGATGGTCATAAAACCAAAGGGCAATATACCTTTAGCGTGAAGTAA

>IR1230_00298

ATGGAAAGAAATCTGGCCAAAATTTCGCAAGATGAGATGGATAAAGTCAATGTCGATCTCGCCGCCGCCGGCGTTGCTTTTAAGGAACGGTACAATATGCCGGTGGTCGCTGATTTAGTCGAGCGTGAACAGCCTGCACATCTGCGCGACTGGTTCCGTGAACGACTGATCGCCCATCGGCTGGCCTCCGTCTCCCTTTCGCGACTACCGTGGGAGCCGAAACAGAAATAA

>IR1230_00299

ATGCTGCGCGTCATTGATACCGAAACCTGCGGCCTGCAGGGGGGCATCGTTGAAATCGCCTCGGTCGATATAGTCGACGGGCAAATTACCAATCCCATGAGCCATCTGGTGCGTCCCGATCGGCCCATCAGCCCGCAGGCGATGGCTATCCACCGGATCACGGAAGAGATGGTCGCTGATAAGCCGTGGATTGAGGAGATTATTCCGCACTATCATGGCAGCCCGTGGTACGTCGCGCATAACGCCAGCTTCGATCGCCGGGTGTTGCCGGAGATGCACGGCGAGTGGATCTGTACGATGAAGCTGGCCCGACGCCTGTGGCCGGGAATCAAATACAGCAATATGGGGTTATATAAATCACGTAAGCTGAACGTGACTACGCCGCCGGGACTGCATCACCACCGGGCGCTGTATGACTGCTATATCACCGCCGCGCTGCTGCTGGATATCATCAATGTTTCCGGCTGGACCCCTGACGAAATGGCCGATATCACCGGTCGGCCGGCGCTGCTGACTACTTTTACCTTCGGCAAATACCGGGGTAAGGCCGTCGCCGAGATTGCTGAAAACGATCCAGGCTATCTGCGCTGGTTATTCAACAATCTCGACCGCATGAGCCCCGAACTGCGCCTGACGCTCAGGCATTACCTCGGGGAATAG

>IR1230_00300

ATGACCTTGCATGGCGATACACGCATCGATAATTATTACTGGCTGCGCGACGATGAGCGCGCGCGACCTGACGTGCTGGAGTATCTGCACGCGGAGAACGCTTACGGCAAGCAAGTGATGGATTCGCAGCTCAGCCTGCAGGAGCGTCTGCTGAAGGAGATTATCGACCGCATTCCGCAGCGGGAAGTCTCTGCCCCCTACAGTAAAAACGGCTTTCGCTACCGTCAGGTGTATGAGCCGGGCTGCGAATACGCTATCTACCAGCGGCAGTCGGTGCTGAAAGAAGAGTGGGATGAGTGGGAGATCCTGCTCGATGCCAACCAGCGGGCGGCGAAGAGCGAGTTCTACACCCTGGGCGGACTGGGCATTGCGCCCAACAACCAGCTAATGGCGGTGGCGGAAGATTACCTTTCCCGCCGCCAGTACGGCCTGCGCTTTTGCGACCTCAGCAACGGCGAGTGGTACCCGGAAATCCTCGAAAATGTCACCTCCGGCTTTGCCTGGAGCAACGATTCACGCTTTGTTTGGTATGTGCGTAAACATCCCACCACGCTGCTCCCTTATCAGGTCTGGCGCCACACCGTCGGGACACCGGCGCAGAGCGACGCGCTGGTCTACGAGGAAAAAGATGAGACCTTCTACGTCAGCGTGCATAAAACCACCTCCCAGCAGTTCGTGGTAATTTATCTCTCCAGCGCCACCACCAGCGAAGTGCTGCTGCTCAACGCCGAACTGCCAGACGCCGAGCCGGTCTGCTTTCTGCCACGGCGTAAAGATCATGAATACAGTCTTGATCACTATCAGCACGCTTTCTATCTTCGTTCCAACCGGGAGGGGAAGAATTTCGGCCTCTACCGTACCGTGCTGCGTGATGAAGAACAGTGGACCACGCTGATCCCCCCGCGTCACGACGTGATGCTCGAAGGATTTACCCTGTTTACCGACTGGCTGGTTGTGGAAGAGCGTCAGCGTGGCCTGACCAGTCTGCGGCAAATTAACCGCAAGACCCGGGAGGTGGTGGGGATCGCTTTTGACGATCCGGCCTATGTCACCTGGCTTGCCTATAATCCGGAGCCCGAAACGTCGCGACTGCGCTACGGCTATTCCTCCATGACTACGCCGGATACGCTGTTTGAACTGGATATGGATACCGGCGAACGAAGAGTGATCAAACAGCAGGAGGTAAAGGGGCTGGATACGAGCTGCTACCAGAGCGAACACCTGTGGGTGACCGCCCGCGATGGCGTCGAAGTCCCGGTCTCTCTGGTCTACCATCGCGAACATTTTCGCAAAGGGAGCAACCCGCTGCTGGTCTATGGCTATGGCTCATATGGCGAGAGTATCGATGCCGATTTCAGCGCCAGCCGGCTGAGCCTGCTCAACCGCGGCTTTGTCTACGCCATCGCCCACGTCCGCGGCGGCGGCGAGCTGGGTCAGCAATGGTATGAAGACGGTAAATTCCTGTGTAAGAAAAATACCTTCAACGACTATCTGGACGTCTGTGACGCGCTGCTTGCCCAGGGGTACGGCGATCCGCGGCTTTGCTACGGCATGGGGGGCAGCGCCGGCGGAATGCTGATGGGCGTGGCCGTGAATGAACGGCCAGAGCTGTTCCATGGCGTGATAGCCCAGGTGCCGTTCGTCGATGTGGTGACCACCATGCTTGATGAAACTATTCCGCTGACCACCGGTGAATTTGAAGAGTGGGGAAATCCGCAGGATGAAACCTATTATCACTATATGAAAAGCTACAGTCCCTATGACGGCGTGAGGGCGCAGGCCTATCCCCACATGCTGGTGACTACCGGTCTGCATGATTCCCAGGTGCAATACTGGGAGCCGGCTAAATGGGTGGCGAAACTGCGGGAACTGAAAACCGACGATAACCTGCTGCTGCTCTGCACCGATATGGATTCCGGACATGGCGGAAAATCCGGGCGCTTTAAAAGCTATGAAGGGGTGGCGCTGGAGTACGCCTTTTTCATCGCCCTGGCGCAGGGGACACTGCCGGGAAAAGCGGCAGTGTAG

>IR1230_00301

ATGGCTAACTGGCTACATCAACTGCAGTCGCTGCTCGGGCAGCAGGGTGCGTCACCGTCCGGCGAGTCGTCAAAAGGGAGCGGCCTGCTGCCCGGCGCCATCGGTGGGCTGGCCGGATTGCTGGTGGCCAGCAAATCGTCACGCAAATTGTTGGCGAAATATGGCACTAGCGCGCTGCTGGCAGGCGGCGGGGCGGTGGCGGGCACGGTGTTATGGAACAAGTATCAGCAGAAAATGCGCGCGCAGAATACGTCCGCCCAGGCGCCTGAGACGGCAGCGGCGCCGACGGATGTCGATCCGCGCAGCGTGCGGCTGATCACGGCCCTGGTATTTGCCGCGAAAAGCGACGGTCATATTGATGACCACGAGCGGGCGAATATTGAAACTCAGCTGCGGGCGGCCAATATTGACGTTCAGGCCCGGGTGCTAATTGACCAGGCGCTGGCCCAGCCGCTGGATCCGCAGCGTCTCTCCGAGGGCATCATCGACCCGCAGGAGGCGCTGGAGATCTACTACGTCAGCTGCGCGGTGATTGATATCGACCATTTTATGGAGCGTAGCTACCTTAATGCGCTGGGAGACGCCCTGGCGCTGCCGAAAGATGTCCGGGCCGATATAGAACAGGATATCCAGTCACAAAAACAAGCGCTAAGCGTCTAA

>IR1230_00302

ATGGCGGTTGAAATTAAATATGTGGTGATCCGCGAAGGTGAGGAAAAAATGTCTTTTGCCAGCAAAAAAGAGGCCGACGCTTACGACAAAATGCTCGATCTGGCAGAGGTGCTGAATGACTGGCTGGTGGCATCGCCGCTGGAGATGGATGACGTCCAGTGCGATACCATGGCGATGTGGCTGGCGGAGCGTAAAGAAGCGCTGCAGCATATCCTGCGAGTTGGCCGACTGCCGGAGCAAGAGACGCCCGCACAGGACGCCTCCGCGCCGTCGGCTACCGCCGAGACTTCTCCGGCGGGTGATGACGCCGTGCCGGCGGCCAAAGCGCGCAAAGCCAGGGCAGCCTGA

>IR1230_00303

ATGACTGTATTAGGCACCGCGCTGCGCCCGGCAGCCACCAAAGTTATGCTGTTAGGTTCCGGCGAGTTGGGTAAAGAAGTCGCTATTGAATGTCAGCGTCTGGGCATTGAAACCATCGCCGTAGACCGCTACCCCGATGCCCCGGCGATGCAGGTCGCCCACCGCGCCCACGTCATCAATATGCTGCACGGCGAGAGCCTGCGCGCCCTGATTGAACAGGAAAAACCTGATTATATCGTGCCGGAGATCGAAGCTATCGCTACCGACACCCTGGTCGAACTGGAACAGGCCGGGCAGAAAGTGGTCCCCACGGCGCGGGCGGCAAAGCTCACCATGAACCGCGAAGGCATTCGCCGTCTGGCGGCGGAAGAGCTGCAGCTTCCCACCTCCCGCTATCGTTTTGCCGACAGCGAAGAAGGCTTCCGCGCGGCGGTTACCGAGATCGGTTTGCCATGCATCGTTAAACCGGTGATGAGCTCCTCCGGCAAAGGCCAGAGCTTTATCCGCTCGGCTGACCAGCTCAGCGAAGCCTGGCGCTATGCGCAGCAGGGCGGCCGCGCTGGCGCCGGACGGTTGATCGTCGAGGGGGTGGTGAACTTCGATTTCGAAATCACCCTGCTCACCGTCAGCGCTGTCGATGGCGTGCATTTCTGCGCTCCGGTTGGCCATCGTCAGGAAGACGGCGATTACCGCGAGTCCTGGCAGCCGCAGCAGATGAGCGACCTGGCCCTCGAGCGTGCCCAGGCCATCGCCCGCGAGGTGGTGCTGGCGCTGGGAGGTTATGGTCTGTTTGGCGTGGAGCTGTTCGTATGCGGCGACGAGGTGATCTTCAGCGAAGTCTCCCCGCGACCACACGACACCGGGATGGTGACGCTTATCTCCCAGGATCTCTCGGAATTCGCCCTGCACGTTCGCGCCTTCCTCGGGCTACCCGTCGGCGCTATCCGCCAGTATGGTCCGGCCGCCTCCGCGGTGATCCTTCCGCAGTTGTCCAGCCAGAACGTCAGCTTTGGCCAGCTGCAGTCGGCCGTCGGGGCTGGCCTGCAGCTGCGTCTGTTCGGCAAGCCGGAGATTGACGGTACCCGCCGCCTGGGGGTAACCCTCGCCGTCGCCGATTCGGTTGAGGAGGCCGTGGCGCGGGCTAAAGCCGCCGCCGCGGCGGTCATCGTCGAAGGGTAA

>IR1230_00304

ATGAAAAACTGGAAAACAACAGCAGAAGCAATCCTCACCTCCGGTCCGGTTGTCCCGGTTATCGTAGTGAAAAAGCTGGAACACGCGGTACCGATGGCGAAAGCGCTGGTGGCGGGCGGCGTTCGCGTGCTGGAAGTTACCCTGCGCACCGAGTGCGCCCTGGAGGCGATCCGCGCGATTGCCAAAGAAGTTCCGGACGCGATTGTCGGCGCGGGCACGGTTACCAACGTTGAGCAGTTGAAAGCGGTAACGGAAGCCGGCGCGCAGTTCGCTATCAGCCCGGGTCTTACCGAATCCCTGCTGAAAGCGGCGACCGAAGACGGCACCATCCCGCTGATCCCGGGGATCAGCACGGTATCTGAGCTGATGCTGGGTATGCAGTATGGCCTGAAAGAGTTTAAATTCTTCCCGGCGGAAGCCAACGGCGGCGTGAAAGCCCTGCAGGCGATTGCGGGTCCGTTCGGCCACATTCGTTTCTGCCCGACCGGCGGTATCTCTCCGGCCAACTACCGTGACTACCTGGCGCTGAACAGCGTACTGTGCATCGGCGGTTCCTGGCTGGTGCCAGCCGACGCCCTCGAAGCCGGCGACTACGATCGCATCACCACCCTCGCGCGAGAAGCGGTGGAAGGCGCGAAATAA

>IR1230_00305

ATGAATTCGACAATGTTACGGGTAACAAATCGCATTATCGAACGGTCGCGTGACACCCGCGCGGCTTACCTCGCAAGGATTAACCAGGCCAAAACCGACACCGTGCATCGTGCGCAACTGGCCTGCGGCAATCTGGCTCACGGATTCGCGGCCTGCCAGGCTGACGACAAAGCCTCCCTGAAGAGCATGTTGCGCAACAACATTGCCATCATCACCTCCTACAACGACATGTTGTCCGCTCACCAGCCCTATGAACACTACCCGGAGATCATCCGCAAAGAGCTGCACAGCGCCAATGCGGTCGGCCAGGTCGCCGGCGGCGTGCCGGCGATGTGCGACGGCGTAACGCAGGGGCAGGACGGGATGGAGCTTTCACTCCTGAGCCGCGAGGTGATCGCTATGTCCGCCGCCATCGGCCTGTCGCATAACATGTTCGACGGGGCCCTGTATCTCGGGGTCTGCGATAAAATTGTTCCCGGTCTGACCATGGCGGCGCTCTCATTTGGCCATCTGCCGTCGGTCTTTATTCCCTCCGGTCCGATGGCCAGCGGCCTGCCGAATAAAGAGAAGGTTCGCATTCGCCAGCTGTATGCAGAAGGGAAAGTTGACCGTATGGCGCTCCTGGAGTCCGAAGCCGCCTCTTATCACGCGCCGGGGACCTGTACTTTCTACGGAACGGCGAACACCAACCAGATGGTGGTCGAATTTATGGGCATGCAGCTGCCGGGCTCGTCCTTTGTCCATCCGGATGCTCCGCTGCGGGAAGCACTGACCGCCGCCGCCGCGCGCCAGGTCACCCGCATGACCGGCAACGGCAATGAGTGGATGCCGCTGGGCAAAATGTTTGACGAAAAAGTGGTGGTCAACGGCATCGTGGCGCTGCTGGCGACCGGTGGCTCCACCAACCACACTATGCACCTGGTGGCGATGGCCCGCGCGGCGGGAATTATCATTAACTGGGACGATTTCTCCGACCTGTCAGACGTGGTGCCGCTGCTGGCGCGCCTCTATCCCAACGGCCCGGCGGATATCAACCACTTCCAGGCGGCAGGCGGCGTTCCGGTACTGGTGCGCGAGCTGCTGAAAGGCGGCCTGCTGCATGAGGATGTTCACACCGTGGCCGGCTTTGGCCTGTCGCGCTACACCCTGGAGCCATGGCTGAATAACGGCGAGCTGGACTGGCGCGAAGGGGCGACAGCGCCATTGGACGACCAGGTCATCGCGACCTTTGAAAAACCATTCTCCCGCCACGGCGGCACCAAGGTGCTGAGCGGCAACCTGGGACGCGCGGTGATGAAAACTTCGGCGGTCCCGGTGGAGAACCAGGTGATTGAAGCGCCGGCGGTGGTATTTGAGAGCCAGCACGACGTGTTGCCGGCCTTTGACGCGGGGCTGCTGGACAAAGACTGCGTGGTGGTGGTCCGTCATCAGGGGCCAAAAGCGAACGGGATGCCAGAATTACATAAACTTATGCCGCCACTTGGTGTATTATTGGACCGCCGTTTCAAAATAGCGCTGGTGACCGATGGCCGACTCTCTGGCGCCTCCGGTAAAGTTCCGTCAGCCATTCACGTCACGCCTGAAGCGTATGATGGCGGCTTGCTGGCGAAAGTGCGCGATGGCGATCTTATTCGCGTGAACGGACAGACGGGGGAACTGACGCTGCTGGTGGATGAGGCCGAACTGGCGGCGCGTCAGCCGCATATCCCTGACCTCAGCGCGTCGCGCGTGGGAACCGGTCGGGAGATGTTTGGGGCGCTGCGTGAGAAGCTCTCTGGCGCGGAGCAGGGCGCAACCTGCATCACTTTTTAA

>IR1230_00306

ATGGCGGTAACGCAAACGGCCCAGGCATGCGACCTGGTCATTTTCGGCGCGAAGGGCGACCTGGCGCGGCGTAAATTGTTGCCTTCCCTGTATCAGCTTGAAAAGGCGGGCCAGATCCACGCTGACACCAGAATCATTGGCGTCGGCCGTGCCGACTGGGATAAAGCGGCTTATACCAAAGTCGTGCGCGAAGCGCTGGAAACCTTCATGAAGGAAAAAATTGATGAAGGTTTGTGGGATACCCTGAGCGGACGCCTGGAGTTCTGTAATCTTGACGTCAATGACACCAGCGGCTTCACCCGCCTGGGTGAGATGCTGGATCAAAAGAACCGTGTCACCATCAACTATTTCGCTATGCCGCCGAGCACCTTTGGCGCGATCTGTAAAGGGCTGGGCGAAGCGAAGCTTAATGCCAAGCCTGCGCGCGTGGTCATGGAAAAACCGCTCGGCACCTCGCTGGAAACCTCCCGTGAAATCAACGACCAGGTCGGCGAGTTTTTTGAAGAGTGTCAGGTCTACCGTATCGACCACTATCTCGGTAAAGAGACGGTCCTCAACCTGCTGGCGCTGCGCTTCGCCAACTCCCTGTTCGTCAATAACTGGGACTGCCGCACCATCGATCACGTCGAAATCACCGTCGCGGAAGAGGTGGGTATTGAAGGGCGCTGGGGTTACTTCGACCAGGCCGGGCAGATGCGCGACATGATCCAGAACCACCTGCTGCAGATCCTGTGCATGATTGCCATGTCGCCGCCGTCCGATCTCAGCGCCGACAGCATCCGTGATGAAAAGGTCAAGGTGCTGAAATCCCTGCGCCGTATTGACCGCTCCAACGTGCGCGAGAAAACCGTTCGCGGCCAGTATACCGCCGGCTTTGCCCAGGGCAAAAAAGTGCCTGGCTATCTCGAAGAGGAGGGGGCCAACAAGACCAGCAACACCGAAACCTTCGTGGCGATCCGCGTCGATATCGATAACTGGCGCTGGGCTGGCGTACCGTTCTATCTGCGCACCGGCAAACGTCTGCCGACCAAATGCTCAGAGGTAGTGGTTTACTTCAAAACCCCGGAACTGAATCTGTTTAAAGAGACCTGGCAGGAGCTGCCGCAGAACAAGCTGACCATTCGCCTGCAGCCGGACGAAGGGGTAGATATCCAGGTACTGAACAAAGTGCCGGGCCTCGATCATAAACATAACCTGCAGATCACTAAACTGGATCTGAGCTACTCCGAAACCTTCAACCAGACGCATCTGGCCGATGCCTATGAACGTCTGCTGCTGGAGACCATGCGTGGCATCCAGGCGCTGTTCGTTCGCCGCGACGAAGTAGAAGAGGCGTGGAAATGGGTTGACTCCATTACCGAAGCCTGGGCAGCCGACCGCGATGCGCCGAAACCGTATCAGGCCGGGACCTGGGGACCGGTTGCGTCGGTGGCCATGATCACTCGCGACGGTCGTTCATGGAACGAGTTCGAGTAA

>IR1230_00307

ATGAGCGCCCTGTTACTAATGAACATGCTGGAAAAAATCCAGTCCCGACTGGAACACCTTAGCAAATCTGAACGCAAAGTGGCGGAAGTGATCCTCGCCACCCCCGAGCAGGCCATTCATTCGAGCATCGCCGCCCTCGCCCTTGAGGCCGGCGTTAGCGAACCGACCGTCAATCGCTTCTGCCGTAGTCTCGAAACCCGCGGATTCCCTGACTTCAAACTGCATCTGGCCCAGAGCCTCGCGCACGGCACGCTCTATGTTAACCGGAATGTCGATGAAGACGACAGCGTTGAGTCATACACGGGAAAAATATTCGAATCGGCCATGGCGAGTTTAGATCAGGTGCACCATTCTCTCGACATGTCGGCGGTAAATCGCGCGGTGGATCTGCTGACTCAGGCCAAAAAAATCGCCTTTTTCGGCCTCGGCTCCTCAGCGGCGGTCGCTCACGACGCGATGAACAAATTTTTCCGCTTTAACGTGCCGGTCATCTATTCCGACGACATTGTCCTGCAACGCATGAGCTGTATGAATTGCGATGACGATGATGTGGTGGTGATTATCTCGCATACCGGAAGAACCAAGAGCCTGGTGGAGCTGGCGCAGCTGGCTCGTGAAAACGATGCCATGGTGATCGCGCTCACCTCCGCCGGTACGCCGCTAGCCAGGGAAGCCACGCTCGCCATTACCCTTGATGTGCCGGAAGATACCGACATCTATATGCCGATGGTCTCCCGCTTGGCCCAGTTGACCGTCATTGACGTGTTGGCGACTGGCTTTACCTTGCGTCGGGGGGCAAAATTCAGAGATAACTTGAAGAGGGTCAAGGAAGCGCTGAAGGAATCGCGTTTTGATAAAGAACCATTCGTACCCAGTGATAATCAATAA

>IR1230_00308

ATGTCCAGAAGGCTTCGCAGAACCAAAATCGTTACTACCTTAGGCCCGGCAACTGACCGCGATAACAACCTGGAAAAGGTTATCGCCGCGGGCGCAAACGTAGTACGTATGAACTTCTCTCACGGCACCCCGGAAGATCATCAGCTCCGTGCCGACAAAGTGCGTGAGATTGCGGCAAAACTGGGCCGTCATGTGGCCATCCTCGGCGACCTCCAGGGACCAAAAATTCGCGTATCGACCTTCAAAGAAGGCAAAATTTTCCTCAACGTCGGCGATAAATTCCTGCTCGACGCAAACCTTGGCAAAGGCGAAGGCGACAAAGAGAAAGTCGGGATCGACTATAAAGGCCTGCCGGCGGACGTCGTGCCGGGCGATATCTTATTGCTCGACGATGGTCGCGTGCAGCTGAAAGTCCTCGAAGTTCAGGGGATGAAAGTGTTCACTGAAGTGACCGTCGGCGGCCCGTTGTCCAACAACAAAGGGATCAACAAGCTGGGCGGCGGCCTGTCTGCCGAAGCGCTGACCGACAAAGATAAAGCTGATATCGTCACCGCCGCTAAAATCGGCGTCGACTACCTGGCCGTCTCTTTCCCGCGCTGCGGCGAAGACCTGAACTACGCCCGTCGCCTGGCGCGCGATGCCGGCTGCGATGCGAAAATCGTCGCCAAAGTTGAGCGTGCCGAAGCGGTTTGCGATCAGGATGCGATGGACGACGTGATCCTCGCCTCCGATGTGGTAATGGTTGCCCGTGGCGACCTCGGCGTCGAGATCGGCGATCCTGAGCTGGTGGGCATCCAGAAAGCGCTGATCCGTCGTGCCCGTCAGCTGAACCGTTCCGTCATTACCGCCACCCAGATGATGGAGTCGATGATCACCAACCCGATGCCGACTCGCGCGGAAGTGATGGACGTGGCCAACGCCGTGCTGGATGGTACCGATGCGGTGATGCTCTCCGCGGAGACCGCTGCCGGTCAGTATCCTTCGGAAACCGTGGCCGCGATGGCGCGCGTGTGTCTGGGCGCGGAAAAAATCCCTAGCCTCAACGTCTCCAAACACCGTCTGGACGTCCAGTTCGACAACGTGGAAGAAGCGATTGCCATGTCGGCGATGTATGCCGCCAACCACCTGAAAGGCATCACCGCCATCATCACCATGACCGAATCGGGCCGTACCGCGCTGATGACTTCGCGCATTAGCTCCGGTCTGCCGATTTTCGCCCTCTCCCGCCACGAGCGCACCCTGAACCTGACCGCCCTCTACCGCGGGGTGACGCCGGTGTTCTTCGACAGCCAGAATGACGGGGTGGCCGCCGCGCACGACGCCGTCAATCTGCTGCGGGACAAAGGCTATCTGGTCTCCGGCGACCTCGTGGTCGTGACCCAGGGTGACGTGATGAGCACCATCGGCAGCACCAACACCACCCGTATCCTCACCGTCGAGTAA

>IR1230_00309

ATGGAAACGAAAAAAAATAATATTGAGTTTATCCCTAAGTTTGAAAAATCCTTTTTACTGCCGCGCTACTGGGGCGCCTGGCTGGGGGTCTTTGCCTTCGCCGGTATTGCGCTGACGCCGCCATCCTTTCGCGATCCGCTCCTCGGCAAACTCGGTCGTCTGGTGGGGCGTCTGGCGAAAAGCTCGCGCCGCCGGGCGCAGATTAATTTGCTGTACTGCTTCCCGGAAAAAAGCGAGTACGAGCGGGAAGCGATTATCGACGCCATGTATGCCTCGGCGCCGCAGGCGATGGTGATGATGGCCGAGCTGGGCCTGCGCGATCCGCAGAAAATCCTTGCCCGCGTTGACTGGCAGGGGAAAGCGATCATCGACGAGATGCAGCGCAATAATGAGAAGGTGATTTTTCTCGTTCCGCATGCATGGGGTGTGGATATCCCGGCGATGCTGATGGCCTCCGGCGGGCAGAAGATGGCGGCGATGTTCCATAACCAGGGTAACCCGGTGTTCGATTACGTGTGGAATACCGTGCGTCGTCGTTTTGGCGGGCGTATGCACGCGCGCAACGATGGGATCAAACCGTTTATTCAGTCGGTGCGCCAGGGCTACTGGGGCTACTATCTGCCCGATCAGGATCACGGCGCCGAACACAGCGAGTTTGTGGATTTCTTTGCTACCTACAAGGCCTCGCTGCCGGCGATTGGCCGCTTAATGAAAGTGTGCCGCGCCCGCGTCGTACCGCTGTTCCCGGTCTACGATGGCAAGACGCACCGCCTGACGGTGCTGGTGCGCCCGCCGATGGACGATCTGCTGGACGCTGACGATACGACCATCGCCCGGCGGATGAACGAAGAAGTGGAAGTGTTTGTTAAGCCGCACACCGAGCAGTATACCTGGATCCTGAAGCTGCTGAAAACGCGCAAGCCGGGTGAAATCGAGCCCTATAAACGCAAAGAGCTGTTCCCGAAAAAGAAATAA

>IR1230_00310

GTGCAACAGATAGCCCGCGCTGTCACTCAGGCATTCAACAATCTGCCACGACCTCATCGCGTTATGCTGGGGTCGCTTAGCGTTCTTACCTTAGCGGTCGCCGTCTGGCGACCCTACATTTACCACCCGGAATCCGCCCCCATCGTCAGAACCATTGAACTGGAAAAAAGCGAGATCCGCTCCCTGCTGCCGGAAGCCTCCGAGCCGATCGACCAGGCCCCGCAGGAAGAAGAAGTCATTCCTCAGGATGAGCTCGACGAAAAAGCCGATAGCGATGCCGGCGGGCACGAATACGTGGTGTCGACCGGCGATACGCTCAGCAGTATTCTGAACCAGTACGGTATCGATATGGGCGATATCGCCCAGCTTTCCTCCGCCGATAAAGAGCTGCGCAACCTGAAGATTGGCCAGCAGCTCTCCTGGACCCTGACCGCCGACGGCGATCTGCAGAGCTTGACCTGGGAGATGTCCCGCCGCGAGACCCGCACCTATACCCGTGTGGATAACGGTTTTAAAATGAGCAGCGAGCTGCAGAAAGGCGATTGGGTCAACAGCGTGCTGAAGGGCACCGTCGGCGCCAGCTTTGTCTCCAGCGCGCGTGATGCCGGGCTGACCAGCACGGAAATCAACGCGGTGATCAAGGCCATGCAGTGGCAGATGGATTTCCGTAAGCTGAAGAAAGGCGACGAATTCTCGGTATTGATGTCGCGCGAAATGCTCGACGGCAAACGTGAGCAAAGCCAGTTACTCGGCGTGCGTCTTCGCTCTGACGGTAAGGATTACTATGCCATTCGCGCCGAAGACGGCAAATTCTACGACCGCAACGGGACGGGTCTGGCGAAGGGCTTTATGCGCTTCCCAACGGCGCGTCAGTTCCGCGTCTCCTCTAACTTTAATCCGCGTCGTCTGAACCCAGTCACCGGCCGCGTCGCGCCGCACCGTGGCGTCGATTTCGCGATGCCGCAGGGAACGCCAGTGCTGGCGGTGGGCGATGGTGAAGTAGTCGTGGCCAAACGCAGCGGCGCTGCCGGGTATTACGTCGCGATCCGCCACGGTCGCACCTATACCACTCGCTACATGCACCTGCGTAAGCTGTTAGTGAAGCCGGGGCAGAAAGTGAAGCGCGGGGATCGCATTGCGCTGTCGGGGAATACCGGACGCTCTACCGGGCCACACCTGCACTACGAAGTGTGGATCAACCAGCAGGCGGTCAACCCGCTGACGGCTAAGCTGCCGCGTACCGAAGGGCTGAGCGGTTCCGACCGGACCGACTATCTGGCGCAGGTCAAAGAGGTCGTTCCGCAGCTGCGGTTTGACTAA

>IR1230_00311

ATGTTACATAAAAATACGCTTCTTTGCGCTGGACTTGGCGCTGTTTTTTTGTTCGCGCAGGTACCGCTGGCCAGCGCCGCCGTGGTGACTTCTATGAAACCGCTGGGCTTTATCGCGGCGGCCATCGCCGATGGGGTGACCGAGACCCAGGTGCTGCTGCCCGATGGCGCCTCCGAGCATGATTATTCCCTCCGTCCTTCTGATGTAAAACGCTTACAGAACGCAGACTTAGTGGTGTGGATTGGTCCGGAAATGGAAGCCTTTATGGATAAGTCGACGCAAAGCATTGCGGCGAATAAAAAGGTGACGATTGCCGAGCTGGATGGGGTCAAACCGCTGCTCATCACCGGGGCGGACGACGATGACGATCACCATGGTCATGACCATGGCGCAGCAGAAAAAGGTGACGGCGATCACCATCACGGTATTTACAATATGCATCTGTGGTTATCCCCAGAGATAGCGCGGCTTTCGGCGGTTGCAATCCACGATAAATTATTGGAACTTATGCCGCAGAGTCGAGCCAAACTCGACAGCAACCTGCAGCAGTTCGAGACCGCGCTGGCGGCGACCGACAAGCAGGTGAGCAATGAGCTGGCACCGCTGAAAGGGAAGGGCTATTTCGTTTTTCATGACGCCTACGGTTACTTTGAAAAACACTACGGTTTGACCTCGCTGGGGCACTTTACCGTCAACCCTGAAATACAGCCCGGTGCGCAGCGTTTACACGAAATCAGAACACAACTGGTTGAGCAGAAAGCCACTTGCGTTTTTGCTGAGCCACAGTTCAGGCCAGCGGTTATCGAAGCTGTTGCCCGGGGCACGTCTGTGCGCATGGGGACCCTGGATCCACTGGGGACAGGAATTACGCTGGGTAAAACGAGTTACCCGCAATTCCTCACCCAACTGGCGAATCAGTATTCGAGCTGCCTGAAAGGAGATTAA

>IR1230_00312

ATGACAAACCTTGTAACGCTGGAAAATGTCTCGGTGGCCTTTGGCCAACGCCGCGTGTTGTCTGACATCTCGCTGGCTCTCACGCCAGGCAAAATCTTAACCCTGCTGGGCCCCAACGGCGCCGGCAAATCGACCCTTGTGCGGGTGGTTCTCGGGCTGGTAGCACCGACAGAGGGTGTTATCAAGCGTGACGCACGCCTGCGCATCGGCTATGTGCCGCAGAAACTGCATCTGGACGCCACGCTGCCGCTGACCGTTAGCCGCTTCCTGCGTTTGCGCCCGGGCACCCGTAAAGACGACATCCTGCCGGCGCTGAAGCGCGTGCAGGCGGGGCATCTGATCGATGCGCCGATGCAAAAGCTCTCCGGCGGTGAAACGCAGCGCGTACTGCTGGCCCGCGCCCTGCTCAACCGTCCGCAGCTGCTGGTGCTGGATGAACCGACCCAGGGTGTTGACGTCAACGGCCAGGTGGCGCTCTACGATCTTATCAACCAGCTGCGCCATGAACTGGACTGCGCGGTGCTAATGGTATCCCACGATCTGCATCTGGTGATGGCCAAAACCGACGAAGTTTTGTGCCTGAACCAGCATATCTGCTGCTCTGGCGCACCGGAGGTGGTCTCCATGCACCCGGAGTTTATCTCCATGTTCGGCCCGCGCGGCGCGGAACAGCTGGGGATATACCGCCACCACCATAATCACCGCCACGACCTTCAGGGGCGAATTGTACTGCGTCGGGGAAACAGCCGCTAA

>IR1230_00313

ATGATTGAACTTCTGTTACCTGGCTGGCTTGCCGGGATGATGCTGGCCTGCGCCGCTGGTCCGCTGGGCTCCTTTGTGGTCTGGCGCCGCATGTCTTATTTCGGCGACACGCTGGCGCATGCGTCTCTGCTCGGCGTCGCTTTCGGTTTATTGCTTAACGTCAATCCGTTCTATGCGGTTATCGCCGTGACCCTGCTGCTGGCTGGCGGGCTGGTGTGGCTGGAAAAACGACCGCATCTGGCGATCGATACCCTGCTGGGCATCATGGCGCACAGCGCGCTGTCCCTGGGGCTGGTGGTGGTCAGTCTGATGTCTAACGTCCGCGTCGATCTGATGGCGTACCTGTTCGGCGATCTGCTGGCGGTTACGCCGCAGGACCTGATCGCCATTGCCATTGGCGTGGTGATTGTGATTGGCATTCTGCTCTGGCAGTGGCGGAATCTGTTGGCGATGACCATCAGTCCGGACCTGGCGTTTGTCGACGGCGTGAAGCTGCAGCGGGTCAAGCTGCTGTTGATGCTGGTCACCGCCCTGACTATCGGCGTGGCGATGAAGTTCGTCGGGGCGCTGATCATTACTTCCCTGCTGATTATCCCGGCCGCCACCGCCCGCCGTTTTGCCCGTACGCCGGAGCAGATGGCCGCCGTGGCGGTGGGCGTGGGCATGCTGGCCGTTACCGGCGGGTTAACCTTCTCGGCTTTCTATGATACCCCGGCCGGGCCGTCGGTGGTGCTCTGCGCCGCGGTGCTGTTTATTCTCAGCATGACGAAAAAAGCCGCCAGCTGA

>IR1230_00314

ATGATTGAAGCAGACCGGCTGGTATCGGCAGACAGCAGCGGCTTTGAAGAGGCCGCTGACCGCGCCATCCGCCCAAAATTGCTGGCAGAGTATGTCGGCCAGCCGCAGGTGCGTTCGCAGATGGAGATCTTTATCCAGGCGGCGAAGCTGCGCGGCGACGCCCTCGATCACCTGCTGATTTTTGGCCCGCCAGGGTTGGGGAAAACCACCCTGGCCAATATCGTCGCCAATGAAATGGGGGTGAATCTGCGTACCACCTCCGGCCCGGTGCTGGAGAAGGCGGGCGATCTCGCCGCGATGCTCACCAACCTCGAACCGCACGATGTTCTGTTTATCGATGAGATCCATCGCCTCTCGCCGGTGGTGGAAGAGGTGCTCTATCCGGCAATGGAAGACTACCAGCTGGATATTATGATTGGCGAAGGTCCCGCGGCGCGTTCCATCAAGATCGATCTGCCGCCGTTTACCCTGATCGGCGCCACCACCCGCGCCGGTTCGTTGACCTCGCCGCTGCGCGACCGCTTTGGCATCGTGCAGCGTCTGGAGTTCTATCAGATCCCCGATCTGCAGCATATTGTCAGCCGCAGCGCCCGCCATATGGGGCTGGAGATGAGCGACGAGGGCGCGCTGGAAGTAGCCCGCCGTTCACGCGGCACGCCGCGCATTGCCAACCGTCTGCTGCGCCGGGTGCGTGACTTTGCTGAAGTGCGCCACGACGGCACCATCTCCGCCGATATTGCCGCCCAGGCGCTGGATATGCTTAACGTCGATGCGGAAGGGTTCGACTATATGGACCGTAAGCTGCTGCTGGCGGTGATAGATAAGTTCTTTGGCGGTCCGGTGGGGCTGGATAACCTCGCCGCCGCGATTGGCGAAGAGCGGGAAACCATTGAAGATGTACTGGAGCCCTACTTAATTCAGCAGGGCTTTCTGCAGCGTACGCCGCGGGGAAGAATGGCGACGGTGCGCGCCTGGAATCATTTCGGGATTACGCCGCCGGAAATGCCGTAG

>IR1230_00315

GTGATAGGCAGACTCAGAGGCATCATTCTCGAAAAACAACCCCCGCTGGTGTTGCTGGAAACGGCGGGCGTCGGCTATGAAGTGCATATGCCAATGACCTGCTTCTATGAGCTGCCGGAGGCCGGGCAGGAGGCGATCGTCTTCACCCACTTTGTGGTGCGTGAAGATGCCCAGCTGCTGTATGGGTTTAACAACAAACAGGAACGTACCCTGTTTAAAGAGTTGATTAAAACCAACGGCGTGGGGCCCAAGCTGGCGCTGGCGATCCTCTCCGGCATGTCGGCGCAGCAGTTCGTCAACGCCGTTGAACGGGAAGAGGTCGCCTCGCTGGTGAAGCTGCCGGGGATTGGCAAAAAAACCGCTGAACGTCTGATCGTTGAGATGAAAGATCGCTTCAAAGGGCTGCACGGCGACCTGTTCACCCCGGCGGCCGATCTGGTGCTGACCTCGCCGGCAGGCCCAACGGCGGACGATGCCGAGCAGGAAGCGGTTGCCGCGCTGGTCGCGCTGGGCTATAAACCGCAGGAGGCCAGCCGGATGGTCAGCAAGATCGCGCGTCCGGACGCCAACAGTGAAACGCTAATTCGCGAAGCGCTGCGCGCTGCGTTGTGA

>IR1230_00316

ATGGCTATTATTCTCGGCATTGACCCGGGGTCGCGCGTTACCGGTTATGGCGTGATCCGCCAGGTTGGGCGGCAGCTGAGCTACCTGGGCAGCGGATGCATCCGCACCAAAGTGGACGATCTGCCGTCGCGGCTGAAGCTGATCTACGCCGGGGTGACGGAGATCATCACCCAGTTCCAGCCCGACTATTTCGCCATTGAGCAGGTCTTTATGGCCAAGAATGCCGACTCGGCGTTAAAGCTCGGGCAGGCGCGCGGGGTGGCGATCGTCGCAGCGACCAATCAGGCGCTGCCGGTGTTTGAATACGCCGCCCGTCAGGTCAAGCAGACCGTGGTCGGGATTGGCAGCGCCGAAAAGAGCCAGGTCCAGCATATGGTGCGCACCTTGCTCAAGCTGCCCGCCAATCCGCAGGCGGATGCGGCGGATGCGCTGGCGATTGCCATCACCCATTGCCATGTCAGCCAGAATGCCGCTCAGATCAGCGAGACCCGGCTCAATCTGGCGCGGGGGCGCTTACGATAA

>IR1230_00317

ATGGCAGGTCATAGTAAATGGGCCAACACCAAACACCGCAAAGCGGCACAGGATGCCAAACGCGGTAAAATCTTTACGAAAATCATTCGCGAGCTGGTCACCGCCGCGCGTCTGGGCGGCGGCGATCCGGCTTCCAACCCGCGTCTGCGTGCGGCCGTTGATAAAGCGCTGTCTAACAACATGACTCGCGACACCCTGAACCGCGCCATCGCGCGCGGCGTCGGCGGCGATGAAGACGCGAACATGGAAACCATCATTTATGAAGGCTACGGTCCTGGCGGCACCGCGGTGATGGTGGAATGTCTGTCCGACAACCGTAACCGTACCGTTGCGGAAGTGCGTCACGCCTTCACCAAAACCGGGGGTAACCTCGGCACCGATGGTTCCGTCTCCTACCTGTTCAGCAAGAAAGGCGTTATCTCCTTCGAGAAAGGCGATGAAGACACTATCATGGAAGCGGCGCTGGAAGCAGGCGCAGAAGACGTGGTGACCTATGACGACGGCGCCATCGACGTCTACACCGCCTGGGAAGAGATGGGCGCTGTGCGCGACGCGCTGGAAGCCGCCGGCCTGAAAGCCGACGCTGCCGAAGTCTCAATGATTCCGTCCACCAAAGCGGATATGGATGCCGAGACGGCGCCGAAGCTGCTGCGTCTGATCGATATGCTCGAAGACTGCGACGACGTGCAGGAAGTGTACCATAACGGCGAGATCTCCGACGAGGTCGCAGCCACCCTGTGA

>IR1230_00318

ATGTCATTTAAGCTGCCCGTTTCGGTGCTGGTGGTGATTTACGCCGAAGATACGAAACGGGTGCTGATGTTGCAGCGGCGCGACGATCCCGCGTTCTGGCAGTCGGTCACCGGCAGCCTGGAAGCTGGAGAGACCGCGCTGCAGGCCGCCGCGCGTGAAGTAAAGGAAGAGGTCGCCATTGACGTTGCCTGCGAGCAACTGACCTTAATCGACTGTCAGCGCACGGTGGAGTTCGAGATATTTTCTCATTTGCGTCATCGCTATGCGCCGGGTGTAGAGCGCAATACGGAATTCTGGTTCTGTCTTGCGCTGCCTCATGAGCGGGAGATTACTTTCACCGAACATCTGGCCTATCGCTGGGTCAGCGCGACGGAAGCCGCTGCGCTGACCAAGTCGTGGAGCAACCGGCAGGCGATTGAAGAATTTGTAATTAACGCCGCCTGA

>IR1230_00319

ATGCGTACAGAATATTGCGGACAGCTTCGACAGTCCCACGTTGGGCAGCAGGTGACTCTGTGTGGTTGGGTCAACCGCCGTCGCGATCTCGGTAGCCTCATTTTTATCGATATGCGCGACCGCGAAGGCATCGTTCAGGTGTTTTTCGATCCGGATCGTGCGGATGCGTTGAAGTTAGCCTCTGAACTGCGTAATGAGTTCTGCATTCAGGTCACTGGCACCGTTCGCGCGCGTGAAGAGAAAAACATCAACGCGGACATGGCCACCGGCGCTATCGAAGTGCTGGCTTCCGATCTGACGATCATTAACCGCTCAGAATCGCTGCCGCTGGACTCCAATCACGTCAACACCGAAGAAGCGCGTCTGAAATACCGCTACCTCGACCTGCGTCGTCCGGAGATGGCGCAGCGCCTGAAAACCCGCGCGAAAATTACCAGCTTTGTGCGCCGCTTTATGGACGACCATGGCTTCCTCGACATCGAAACCCCGATGCTGACTAAAGCCACCCCGGAAGGCGCCCGCGATTACCTCGTGCCTTCCCGCGTGCACAAAGGCAAATTCTACGCGTTGCCGCAGTCGCCGCAGCTGTTCAAACAGCTGCTGATGATGTCCGGCTTCGATCGCTACTATCAGATCGTCAAATGCTTCCGCGATGAAGACCTGCGTGCCGACCGTCAGCCTGAATTCACCCAGATCGACGTGGAAACCTCCTTTATGACCGCGCCGCAGGTGCGTGAGATCATGGAGGCGATGGTGCGTCAGCTGTGGCTGGAAGTGAAAGGCGTCGACCTGGGCGAGTTCCCGATCATGACCTTCGCCGAAGCCGAACGTCGCTATGGCTCCGATAAACCGGATCTGCGTAACCCGATGGAGCTGGTGGACGTCGCCGACCTGCTGAAGTCTGTGGAATTCGCGGTCTTTGCCGGCCCGGCCAACGATCCGAAAGGGCGCGTGGCGGCCCTGCGCGTCCCGGGCGGGGCCTCTCTGACCCGCAAGCTGATTGACGAATACGGCAACTTTGTGAAGATCTACGGCGCGAAAGGGCTGGCCTATATTAAAGTGACCGAGCGTGCCAAAGGCATGGACGGGATCAACAGCCCGGTGGCCAAATTCCTGACCGCTGAAATCGTGGAAGCGATCCTTGACCGCACCGGCGCGCAGGATGGAGACATGATCTTCTTCGGCGCCGACAACAAGAAAGTAGTGGCCGACGCGCTGGGCGCGCTGCGTCTGAAGCTGGGTAAAGACCTGAGCCTGACTGACGAGAGCAAATGGGCGCCGCTGTGGGTGATCGACTTCCCGATGTTCGAAGACGACGGCGAAGGCGGCCTGACGGCGATGCACCATCCGTTCACCTCGCCGAAAGATATGACCGCGGATGAGCTGAAAGCCGCCCCGGAAGAGGCCGTGGCCAACGCCTACGATATGGTCATTAACGGTTATGAAGTCGGCGGCGGTTCGGTGCGTATCCACCGCGGCGAGATGCAGCAGACCGTATTTGGTATTCTGGGCATTAACGAACAGGAACAGCGTGAGAAATTCGGCTTCCTGCTGGATGCGCTGAAGTACGGTACGCCGCCGCATGCAGGTCTGGCCTTCGGTCTTGACCGTCTGACCATGCTGCTGACCGGTACCGATAACATCCGCGACGTTATCGCTTTCCCGAAAACCACCGCGGCCGCCTGCCTGATGACCGAAGCGCCAAGCTTCGCCAATCCTGCGGCGCTCGGTGAACTGGGTATTCAGGTTGTGGAGAAAGAGGCAAAAGCGTCTCTGGAGAACAAGTAA

>IR1230_00320

ATGTTAGAACTCAATGCAAAAAACACGGCGCTGGTGGTAATCGATCTGCAGGAAGGCATTCTGCCTTTCGCCGGCGGCCCGCATCGGGCTGACGAGGTGGTGGCGCGCGCCGCTCGCCTGGCAGACAAGTGCCGGCAACAGGGTTCGCCGGTCATCATGGTCCGCGTTGGCTGGTCGGCCGATTTCGCCGAAGCGCTGAAACAGCCTGTCGACGCTCAGGCCGGGGCGCATACGCTGCCCGAAAACTGGTGGACCTACCCGGCCACGCTGGGTAAGCAGGAGAGCGATATCGAAGTGACCAAACGCCAGTGTGGCGCGTTCTATGGCACCGACCTTGAGCTTCAGCTGCGCCGCCGCGGGATCGACACCATTATTCTCTGCGGCATCTCCACCAACATCGGCGTCGAATCCACCGCCCGCAATGCCTGGGAGCTGGGTTTTAACCTGGTCATCGCCGAAGATGCCTGCAGCGCCGCCTCCGCCGAGCAGCATCAGGGCAGTATGACGCATATTTTCCCGCGCATCGGCCGGGTGCGCAGTACCGAGGAGATCCTCACGGCGCTATGA

>IR1230_00321

ATGATTTATATTGGGCTTCCCCAGTGGTCGCACCCGAAATGGGTGCGCCTTGGCATCACCAGCCTTGAAGAGTACGCCCGCCACTTTAACTGTGTGGAGGGCAACACCACCCTGTATGCGCTACCAAAGCCGGAGATTGTCGCCCGCTGGTACGAACAGACTCACGATGACTTCCGCTTCTGCTTCAAATTTCCGGCCACCATCTCGCACCAGGCCGCCCTGCGCCATTGCGACGAGTTAAGCAGTGAATTTTTTGCCCGCCTGGCGCCGCTGGCCTCGCGCATCGGCCAGTACTGGCTGCAGCTGCCGGCGACGTTCGGTCCTCGCGATCTCCCCGCGCTATGGCATTTTCTCGACGGACTGCCGAAGGATTTCACCTACGGCGTCGAAGTTCGTCATCCCGAGTTTTTCGCCAAAGGGGAAGCGGAACAGCAGCTCAACCGCGGGCTGCACGAGCGTAACGTCAACCGCGTGATCCTCGACAGCCGCCCGGTCCATAGCGCTGCGGCCACCAGTCCGGCGATGATCGATGCCCAACAAAAGAAACCGAAGGTGCCGGTACATGCGGTGATGACCGCCCGGCAGCCAATGGTGCGCTTTATCGGCGGTGACGATATGGCCCATAACCGGGAACTGTTTCGCGTCTGGCTTCAGACCCTGGCGAAGTGGCATCAGTCGGGGACCCCGTGGTTGTTCCTGCATACCCCGGACATTGCTTTCGCCCCGGCGCTGGTGGATACCCTGTGGGGTGACCTGCGCGCTGCCCTGCCGGCGGCAGGAAATGCGCCGTCGATTCCGCAGCAATCTTCTCTTTTCTGA

>IR1230_00322

ATGGTCAGCGCGCTGTATGCCGTTTTAGGTGCGTTATTGTTAGTGAAGTTCTCATTTGACGTGGTGCGTCTGCGCACCCAATACCATGTAGGCTACGGTGACGGCGGCTTTAGCGAGCTGCAGGTTGCCATCCGCGTTCACGGCAATGCCGTCGAATATGTGCCGATTGGCCTCATTTTGCTGTTATTTATGGAGATGAACGGCGCTCAGACCTGGATGGTGCACGTTTGCGGGATTTTATTGATTGTCGGGCGACTGATGCACTCCTGGGGCTTTCATCACCGCGTCTACTACTGGCGCCGCTCCGGCATGAGCGCCACCTGGTGCGCGTTGTTGCTAATGGTGCTGGCCAATCTTTGGTATATGCCCTGGGAGTTGGTTTTCTCCCTCCGTTAG

>IR1230_00323

ATGTCTCACCGCGATACGCTTTTTTCCGCGCCGATTGCCAGCCTCGGCGACTGGACCTTTGATGAACGGGTTGCTGAAGTCTTCCCCGATATGATCCAGCGTTCTGTGCCCGGCTACTCCAATATCATCTCGATGATCGGCATGCTGGCCGAGCGCTTCGTTCAGCCCAACACTCAGGTTTACGATCTTGGCTGTTCGCTGGGCGCCGCCACCCTCTCCGTGCGCCGCAATATTAGCCATCCGGGCTGTCGCATTATCGCTATCGATAACTCGCCCGCGATGGTGGAGCGCTGCCGTCGCCATATTGACGCTTATAAAGCGCCGACGCCGGTCGAAGTGATTGAAGGGGATATCCGCGACGTGACCATTGAAAACGCCTCGCTGGTGATCCTGAACTTTACTATTCAGTTCCTTGAGCCGGGCGATCGCCAGGCGATCCTCAACAAAGTCTATCAGGGGCTGAATCCGGGCGGCGCGCTGGTGCTCTCGGAAAAATTCAGCTTCGAAGACGCCCACGTTGGCGAACTGTTGTTCAATATGCATCATGACTTTAAGCGCGCCAACGGCTACAGCGAACTGGAGATCAGCCAGAAACGCAGCATGCTGGAAAATGTGATGCTGACGGACTCCGTCGAAACCCATAAAGCCCGACTGCGTCAGGCTGGGTTTGAACATGCCGAACTGTGGTTCCAGTGCTTTAACTTTGGCTCGCTAGTGGCCGTGAAAGCCGGGGAGCAGGCATGA

>IR1230_00324

ATGATCGACTTCAGTAACTTCTATCAGCTGATCGCCAAAAGCCCGCTGTCTCACTGGCTGGAGACGCTGCCTGCCCAGGTAGCCGCCTGGCAGCGCGATGCCCTGCACGGCAAGTTTCGTGAATGGGAGCGCGCCGTAGAATTTCTGCCGGAGCTAACGCCCTGGCGTCTGGATCTTCTGCACAGCGTCACCGCCGAGAGCGAAACGCCGCTCAGCGAAGGCCATCAGCGGCGGATCGAAAATCTGCTTAAAAACCTGATGCCGTGGCGCAAAGGCCCTTATTCGCTGTACGGCATCAATATCGATACCGAATGGCGATCCGACTGGAAATGGGAGCGCGTTCTGCCGCACCTGTCCGATCTCACTGGCCGGACTATCCTCGACGTGGGCTGCGGCAGCGGCTATCACATGTGGCGGATGATCGGCGCCGGCGCACATCTGGCGGTCGGTATCGATCCCACCCAGCTGTTCCTGTGCCAGTTTGAAGCGGTGCGTAAGCTGCTCGGTAACGACCAGCGGGCGCATTTGCTGCCGCTGGGCATTGAGCAGCTGCCGGCGCTGGAGGCCTTCGACACCGTGTTCTCGATGGGCGTGCTCTACCACCGCCGCTCGCCGCTGGACCATCTGTGGCAGCTGAAAGATCAGCTGGCGCCAGGGGGCGAGCTGGTGCTGGAGACGCTGGTGGTTGAAGGAGATGAAAATACGGTGCTGGTTCCGGGCGACCGCTATGCGCAAATGCGCAACGTCTATTTTATCCCCTCAGCGGCGGCCCTGAAAATGTGGCTGGAGAAGTGCGGGTTTATTGACGTGCGTATCGTCGACGCCTGCGTCACCTCGACAGAGGAGCAGCGACGCACCGAATGGATGACGACCGAATCGCTGGCCGATTTCCTCGACCCACAGGATCAGCGCAAAACGGTGGAAGGCTATCCGGCGCCATTACGAGCTGTGATCATTGCCACCAAACCGGAAACCCAGCAGTCGCTGGCGAAAAAAGCGAGATAA

>IR1230_00325

ATGGCGGTACTGGAAGTGTGTTGCTACAGCGTGGCGTGCGCCCGGGAGGCTGAGCGCTACGGCGCCGATCGTATTGAGCTCTGCGCCGCGCCGCAGGAAGGGGGATTAACCCCCTCTTATGGGGTGCTGGTCTCGGCCCGCGAGGCGATTACTCTCCCGGTGCATCCGATCGTTCGCCCGCGCGGCGGCGATTTCTGCTACACCGAGGAAGAGTTCGCCGCGATGCTTAACGATATTCGCATGGTGCGGGATCTGGGCTTTCCCGGGCTGGTGACCGGCGTGCTGGATGCTGATGGCCAGGTCGATATCCCGCGGATGAAAAAAATAATGGCCGCGGCGGGGCCGCTGGCGGTGACCTTTCATCGCGCGTTCGATCTGTGCGCTGACCCGCGTCAGGCCTGGAAAACGCTGGGTGAGCTGGGGGTCAAACGTATCCTGACTTCCGGTCAGCAATCCTCGGCGGAGAAAGGTATTTCATTAATTACGGAACTTATTGCCGCAGGGGATACTCCAATCATTATGGCCGGTGCGGGAGTCCGCGCCGCGAACCTGCCGCTGTTTCTGCAGGCGGGGGTGAAAGAGGTCCATAGCTCTGCTGGCCACTGGTTGCCGTCGGAAATGCGCTTTCGTCATCCGGGCGTTTCGATGTCAGCCGATCCCGATGCCGATGAATACAGACGCTACGCCGTGAATGGCGCAGCGGTGGCGGAGATGAAAAGGATTATCTCCGCCTGGAGAAGTTAA

>IR1230_00326

ATGGCAAACTGGCAGCAAAATGAACAACTGGCCGATATTACGGCGGATCTCCCGCGATTTTCCGATGCGCTTCAGCGCTTCACTGCGCGACTGGGACTGGAGATCGCCGGGCTCGACGCCGACCATATCTCCCTGCGCTGTCATCAGAATACCACTGCGGAACGCTGGCGCCGCGGTCTGGAGCAGTGCGGCACCCTGCTGTCGGAGAATATGATCAACGGCCGGCCCATTTGCCTGTTCAAACTGGCGGAGCCGGTCTGCGTGGCGCACTGGCGCTTCCATATCGTTGAGCTGCCGTGGCCGGGTGAAAAGCGTTATCCCCATGAAGGCTGGGAGCATATCGAAATCGTCCTGCCCGGCGATCCGGCAACCTTAAATGCCCGCGCTCTGGCGCTGCTGGCCGATGACGGACTCAGCCAGCCGGGGATCGTGGTCAAAACCAGCTCGCCGAAAGGGGAGCATGAGCGCCTGCCGAATCCTACCCTGGCGGTGACCGACGGCAGCGTGACGGTCAAATTTCATCCATGGTCTATTGAACAAATCGTCGCCAGCGAGCAAGCCGATACGTAA

>IR1230_00327

GTGAATATTCAGGCTCTTCTCTCAGAAAAAGTCAGCCAGGCCCTGATCGCGGCAGGCGCGCCTGCCGATTGCGAACCCCAGGTCCGCCAGTCAGCAAAAGTTCAGTTCGGCGACTATCAGGCCAACGGCGTGATGGCTGTCGCCAAAAAGCTGGGTATGGCGCCGCGACAACTTGCAGAGCAGGTACTGTCTCATCTTGACCTCAACGGCATTGCCAACAAGGTTGAAATCGCCGGGCCGGGCTTTATCAATATTTTCCTCGACCCCGCGTTTCTGGCGGACAACGTCAACCGCGCGCTGCAGTCTGAGCGCCTGGGCGTCACGAAACCGCAGGCGCAGACTATCGTCGTCGACTACTCGGCACCCAACGTAGCGAAAGAGATGCACGTCGGCCATCTGCGTTCGACCATCATTGGCGATGCTTCAGTCCGTACCCTGGAGTTCCTGGGTCATAAGGTGATCCGCGCCAACCACGTCGGCGACTGGGGCACCCAGTTCGGGATGCTGATCGCGTATCTGGAAAAACAGCAGCAGGAAAACGCCGGCGAGATGGCCCTGGCCGACCTCGAAGGTTTCTATCGCGAAGCGAAAAAGCACTATGACGAAGACGAAGCCTTCGCCGAGCGCGCGCGCAGCTACGTGGTTAAGCTGCAGGGCGGTGATGAGTATTTCCTGCAGATGTGGCGTAAGCTGGTCGACATCACCATGTCGCAAAACCAGATCACCTACGATCGCCTGAATGTGACCCTGACCCGCGATGACGTGATGGGTGAGAGCCTGTACAACCCGATGCTGCCGGGGATCGTCGCCGATCTGAAAGCCAAAGGGCTGGCGGTTGAGAGCGAAGGCGCTACCGTGGTCTTCCTTGATGAGTACAAAAACAAGGAAGGCGAACCGATGGGCGTCATCATCCAGAAAAAGGATGGCGGCTACCTCTATACCACCACCGATATCGCCTGTGCGAAGTATCGCTATGAAACGCTGCACGCCGACCGCGTGCTCTACTACATCGACTCTCGTCAGCACCAGCACCTGATGCAGGCGTGGACCATCGTGCGCAAGGCAGGCTACGTGCCGGACTCCGTGCCACTGGAGCACCATATGTTCGGTATGATGCTGGGGAAAGACGGTAAGCCGTTTAAAACCCGCGCCGGCGGTACCGTGAAGCTGGCGGATCTGCTGGACGAAGCGCTGGAGCGCGCCCGTCGCCTGGTGGCCGAGAAGAACCCGGATATGTCGGCTGACGAGCTGGAAAATCTGGCGAAAGTGGTGGGTATCGGCGCGGTTAAATATGCCGACCTGTCGAAAAACCGCACCACCGACTACGTCTTCGACTGGGATAATATGCTGGCGTTCGAAGGCAACACCGCGCCGTATATGCAGTATGCCTATACCCGCGTGCTATCCGTGTTCCGCAAAGCCGGCATCGACGAAAACGCGATGATCGACGCGCCGGTGGTCATCGCCGAAGATCGCGAAGCTCAGCTGGCCGCGCGCCTGCTGCAGTTTGAAGAGACCCTCTCCGTGGTCGCTCGCGAAGGGACGCCGCACGTGATGTGCGCCTACCTCTACGATCTGGCGGGCCTGTTCTCCGGCTTCTACGAGCACTGCCCGATCCTCAGCGCGGAAAGCGAAGAGACCCGCAACAGCCGTCTGAAGCTGGCGCTGCTCACGGCGAAAACCCTCAAGCTGGGTCTCGATACCCTCGGTATCGAAACCGTCGAGCGTATGTAA

>IR1230_00328

ATGAAAGTTTGGCCGGTCAAACATAGCCCATTACTGCGTCAGCCTGAGCGTTTTATCGCCCGCAGCGAACTGAAGGCGCTGATCCGCAACGTGACGCAAAACCTGGTGAATATTAAGGATGAGAGCGGGCAATTTTTACTACGCCTGGATGACGGGCGCGTGATCGATACCAAAGGCTGGGCCGGCTGGGAGTGGACCCACGGCGTTGGGCTGTACGGCATCTATCAGTATTACCAGCAAACCGGCGATATCGAGATGCGTGATATCATCGATCGCTGGTTTGCCGACCGCTTCGCCGAGGGAGCAACGACCAAAAACGTCAATACTATGGCCCCGTTTCTGACCCTGGCTTACCGCTTTGAAGAGACCGGCCGTATGGCGTATCTGCCGTGGCTGGAGAGCTGGGCGGAGTGGGCGATGCACGAGATGCCGCGCACCGAGCAGGGCGGTATGCAGCACATGACTCTGGCGGAGGAGAACCATCAGCAAATGTGGGATGACACGCTGATGATGACCGTGTTGCCGTTAGCCAAAATTGGCAAGCTGCTTAACCGCCCCCAGTATGTGGAGGAGGCAACCTACCAGTTCCTGCTCCATGTGCAGAACCTGATGGACCGGGAGACCGGGCTGTGGTTTCACGGCTGGAACTATGAGGGGCGGCATAACTTTGCCCGGGCGCGTTGGGCGCGGGGCAACAGCTGGCTCACCATGGTGATCCCGGATTTTCTGGAACTGGTGGATCTCCCCGAGGGGAATGCGGTACGGCGCTATCTGATTACAGTCCTCGACGCACAGATTGCCGCCTTAGCAGAATGCCAGGACGACAGCGGCCTGTGGCATACCTTGCTGGACGACCCGCACTCTTATCTTGAGGCCTCGGCGACCGCCGGCTTTGCCTACGGGATTCTCAAAGCGGTGCGCAAGCGTTACGTCGGGCAGCACTACGCCGGGGTGGCTGAAAAAGCGATTCGCGGGATTGTGCAGAATATTTCGCCGCAGGGGGAGCTGCTGCAGACCTCATTTGGCACCGGGATGGGGTCAGACCTCGACTTTTATCGCCAGATCCCGCTTACCTCGATGCCCTACGGTCAGGCGATGGCGATCCTTTGTCTGACGGAGTATCTACGCAAGTACTTCTGA

>IR1230_00329

ATGAAAACACGAAAAATTGGACTGGCTAACTATCTGGCCTACGGCGCGGGCGATTTTCTCGGCGCCGGCACGACGGCGTTAACCGCCGCATGGCTGCTCTACTTTTACACGACCTTTTGCGGACTGACCCCGATTGAGGCCACGCTTATTTTTGCTGCCGCCCGCGTGCTGGACGCGGTGGTAAGCCCGCTGATGGGCTTTCTCACCGATAACTTTGGCACCACCTGGCTTGGTAAGCGCTTCGGCCGCAGAAAATTCTTTATTCTGCTCGGCATCCCCTGCGTATTCAGCTATTCAGCGATGTGGGTCGGCGAGATGGGCTTCTGGTACTACCTGGCGACCTATCTGCTGTTTGATATGGTCTATACCATGATTCTGGTGCCCTATGAGACGCTGGTGCCGGAGATGACCGATGACTTTAAACAGAAAACCAAATTCTCCGGCGCGCGCATTTCGATGGCGCAGATGTCGGCGATTTTAGCCTCCTTCCTGCCGGGGATCCTTCTCTCCTGGCTCGGCAAAGATAATGCCAGCTCGTTCTTCTATGCCAGTCTGGTGTTCTCGGTGCTGTGCGCGGTGATGCTGACTCTGGTCTGGTGTTTCACCTGGGAACGACCGCGGGAGGCGTGGTCGGAAGCGGCCCTGCGGGCGGAAGCGGAAAAACAAAACTTAACCCTTGCTCAGAGCCTCAATCGCCTGGTGATTGAGCTGAGTTCTACGCTGCGGATAAAAATTTTCCGCCAGCACCTCGGCATGTATCTGGGGGGCTATATCGCCCAGGACGTCTTCAATGCCGTGTTTACCTATTACGTGGTCTTTGTTCTGATGCAGGAAGCCGCCGTCGCCTCGAACCTGCTGGGGACGATGGCCATCTTCCAGTTTATTGCAGTAATCGCCATGATCCCGCTGTGCATTCGTTTTGGCCCCGCGCCGTCGTACCGCATGGTGGTGGTCCTGTTTGGCCTGAGCTCACTCTCCTACGCGCTGCTCTATTACGCCGGACTGAGCGACGTCTATTCCTTATTGCTGCTCATCTCTGCTGTCGCCGGACTGGGACGCGGCGGGATAAATTACGTACCGTGGAATACCTACACCTATATTGCCGACGTCGACGAAGCGATCACCGGCCAGCGCCGGGAGGGGATTTTTGCCGGCATCATGACCCTGACCCGCAAAGCCTCGCAGGCGGGGGCGGTGATGCTGGTGGGGATCATTATGCAGTTGTCGGGGTTTGTTTCCGGGCAAAAAATACAGCCTGAAGGGGTCAGCCATACCATTCTGCTGATCCTCAGCGTCGGCACTCTGGTGGTGTTAGCCTGCGGCTTCCTCGTCTCGCTGCGCTTCAAGCTCAACCTGCACACCCACAGCGTCCTGCGCAGCGAGACCCTCAGAATGCGCGAGCTCGGCTACGCTCAGTCGGAACAGACCAGTGCGGAACACCGTGCGGTGGTCGAGCTGCTGGCGGGGATGCCTTACGACTGTCTGTGGGGCAATAACAATATTGGCTATCTGAATCGTCATAAACCTGCTGCCCCGGCGCTCATTAAGGGCGGCGCTTTGAATTCGACATATACCCGAGGTTAA

>IR1230_00330

ATGCCTTATACCCATCTGCTGGTTGCCGTCGCGCCAACGCCGGAGAGTCAGGTATTAATTAAAAAAGCGGTGTCGATAGCCCGCCCCGTTAACGCGAAAGTGAGCTTAATCACTCTCGCTACGGATCCGGAGCTGTATAACCAGTTCGCCGCACCGATGATGGAAAATCTGCGTGAAGTGATGCAGGAAGAGACGCGGGAGTTTCTCAATGAGCTGGCTCGCCACGCGGACTACCCAATAGAAAGAATGACCATCGCCTGCGGCGAGCTAGGCCATCATATTAAGGACTTCTGCCGGTCGCACCGTGTTGATCTGGTGATCTGTGGCAATCACAACCATTCCCTCTTCTCACGCGCCACCTGCTCGGCGAAAAGTATCGTCGGCAGCTGCGGCGTTGATGTGCTGTTGGTTTCCCTTGAAAAGGGCTAA

>IR1230_00331

ATGAGTCGTTTAGTCGTAGTCTCTAACCGTATCGCCTTACCGGACGATAAAAAATCGAGCGCTGGCGGTCTGGCCGTCGGCATCCTGGGGGCACTGAGAGCGGCGGGGGGTCTGTGGTTTGGCTGGAGCGGCGAGATTGGCGATGACCAGCAGCCGCTCAAGCAGGTCTCCCGCGGGAATATTTCATGGGCGTCGTTCAACCTTAATGAACGGGACCATGATGAATACTACAACCAGTTCTCTAACGCCGTGCTGTGGCCGGCTTTCCACTATCGCCTTGATCTGGTCAGCTTTCAGCGCGAAGCCTGGGAGGGATATCTGCGGGTTAACGCCATGCTGGCGGACAAGCTTCTGCCGCTGATTGAGCCAGACGATACGCTGTGGATCCATGATTACCATCTTCTGCCCTTCGCCAGCGAGCTGCGCAAGCGCGGGGTGAACAATCGCATCGGTTTTTTCTTACATATTCCGTTCCCGACGCCGGAGATTTTTAACGCTCTGCCGCCGCACGCTGAGCTGCTGGAGCAACTGTGCGATTACGATCTGCTGGGCTTCCAGACCGAAAGCGACAGAACCGCTTTTCTCGACAGCATTGCTATGCAGACTCGCCTATCCGACCTCGGCGATAAACGCTATCAGGCATGGGGTAAGGCGTTCAGTACCGAGGTTTATCCGATAGGGATCGATCCGGACGAAATTACGCGCAACGCTAAAGGTCCGCTGCCGCCGAAGCTGGCCCAGCTGAAGAACGAGCTGAAAAATGTGAAGAATATCTTCTCCGTGGAGCGTCTTGACTATTCGAAAGGGTTGCCGGAGCGATTCCTCGCCTACGAGACGCTGCTCGAGAAGTACCCTCAGCACCACGGCAAAATCCGTTATACCCAGATCGCACCGACCTCCCGTGGCGATGTGCAGGCCTATCAGGATATCCGCCACCAGCTGGAAACCGCAGCCGGACGCATCAACGGACAGTTTGGTCAGCTTGGCTGGACACCGCTCTACTATCTGAACCAGCATTTTGATCGTAAGCTGCTGATGAAGGTCTTCCGCTATTCTGACGTCGGGCTGGTGACGCCGCTACGCGATGGTATGAACCTGGTGGCGAAGGAGTACGTCGCGGCGCAGGATCCGGATAACCCTGGCGTACTGGTCCTGTCGCAGTTTGCCGGGGCGGCGCAGGAGCTGACCAGCGCCCTGATCGTCAATCCTTACGATCGCGACGAGGTTGCCGCCGCGCTGGATCGCGCGCTCAGCATGCCGCTGGCGGAACGTATCGCCCGTCATTCAGCGATGCTGGACGTGATCAGAGAAAATGATATTCATAACTGGCAGGCGCGCTTTGTCGAGGATCTGCAGCATATTTCACCGCGCAGCGAGGAGAGCCGTCTGCGGGGGAAGATAGCCACCTTCCCTAAACTGGCCTAG

>IR1230_00332

GTGGATAACCAGATATCTGTACCGCCTGCGCTAACCGGAAACTACGCTTTTTTCTTTGACCTCGACGGTACTCTTGCCGATATCCAGCCGCACCCCGATCAGGTGGTGATACCCGACAGCACGCTGCAGGCGCTCAATGCGCTGGCCCAGCAGCAGGGGGGCGCGGTGGCATTGATTTCAGGGCGCTCAATGGCTGAACTTGACGCGCTAACCCATCCCTGGCGGCTACCGCTGGCCGGCGTTCACGGGGCCGAGCGCCGTGATATCAATGGTAAAACCTACATCGTCTCTCTGCCGACAGCGCTGCGCGATGAGATCGCGGCTGAGCTGACGTCGGCGCTGGAAGCGCTCCCGGGATGTGAGCTGGAGAGTAAAGAGATGGCTTTCGCGCTCCACTATCGCCAGGCCCCGCAGCAGCAGAGCGCCGTGCTGGAGCTGGCGCAGCGGATTGTTCAGCGCTATCCGCTGCTGGCGTTACAGCTTGGCAAGTGCGTGGTGGAGATTAAACCCCGCGGCGTCAACAAAGGGGAGGCGATTACCGCGTTCATGCAGGAGGCGCCATTCGCCGGCCGCGAACCGGTTTTTGTCGGCGATGATTTGACCGACGAGGCGGGATTCAGCGTCGTTAATCAACTGCAGGGAATGTCGGTAAAAGTTGGCGCCGGGGAAACTCAGGCGCATTGGCGGTTGGCGGATGCCGCCGCTGTAAGGACGTGGTTGCAACATCTGGCGTATGACGCGCAAACCGAAAGGAGGGATGACCATGAGTCGTTTAGTCGTAGTCTCTAA

>IR1230_00333

ATGTCATCAGTGACTACGTCCGGGGCTACCCGCTCAACCTTTAGCTTTGCCCGCATCTGGGATCAGTTTGGCATGCTGGTGGTGTTTGCGGTGCTGTTTATCGGCTGTGTGATTTTTGTGCCCAACTTTGCTTCATTCGTCAATATGAAAGGGCTGGGGCTGGCGATCTCGATGTCCGGGATGGTGGCCTGCGGCATGCTGTTTTGCCTGGCCTCCGGCGATTTTGACCTGTCGGTGGCGTCGGTTATCGCCTGCGCCGGGGTCACCACCGCGGTGGTGATTAATCTCAGCGAAAGCCTGTGGCTGGGGATCGCCGCAGGCCTGTTGCTGGGGGCGCTTAGTGGCCTGGTTAACGGCTTTGTCATTGCGCGCCTTAAGATCAACGCGCTGATCACCACGCTGGCCACCATGCAGATTGTCCGCGGCCTGGCCTATATTATCTCCGACGGCAAAGCGGTGGGGATCGAAGATGAACGTTTCTTCACCCTCGGCTATGCCAACTGGTTTGGTTTGCCGGCGCCGATCTGGCTGACCGTCGCCTGCCTGGTGGTTTTCGGATTATTACTAAACAAAACCACCTTTGGCCGTAATACGCTGGCTATTGGCGGCAATGAAGAGGCGGCGCGCCTGGCCGGCGTCCCGGTGGTGCGCACCAAGATTATTATCTTTGTGCTCTCCGGTCTGGTATCGGCCGCAGCGGGGATCATTCTGGCGTCGCGCATGACCAGCGGCCAGCCGATGACCTCGATTGGCTATGAGCTGATTGTTATTTCGGCCTGCGTGCTGGGCGGGGTGTCGCTGAAGGGCGGGATCGGCAAGATCTCCTACGTGGTGGCCGGGATCCTGATCCTCGGAACCGTGGAAAATGCGATGAACCTGCTGAATATCTCGCCATTCTCACAATACGTCGTGCGCGGGGTGATCCTGCTGGCGGCGGTGATCTTCGACCGCTACAAACAAAAAGCCAAACGTGCGGCCTGA

>IR1230_00334

ATGCAACAGTCTACCCCTTATCTCTCATTTCGCGGCATCACCATGACGTTCCCTGGCGTAAAGGCCTTGTCCGATATCAGTTTTGACTGCTACCCAGGACAGATCCACGCGCTGATGGGCGAAAATGGCGCAGGCAAATCGACGCTGTTGAAAATTCTCAGCGGCAACTATATCCCGACCGCGGGCCATCTGCAGATCGGCGGCCAGCAGATGGCATTCGCCAATACCATGGAGGCGCTGAATGCCGGCGTGGCAATCATCTACCAGGAGCTGCATCTGATCCCGGAGATGACCGTCGCCGAAAATATCTATCTCGGTCAGTTGCCGCACAGAGGCGGCATCGTCAATCGCTCGCTGCTCAACTATGAGGCCCGCCTGCAGCTGGAGCATCTGGGTCTGGATATCGACCCGGAGACGCCGCTGAAATATCTCTCCATTGGTCAATGGCAGATGGTGGAGATTGCCAAAGCGCTGGCGCGTAACGCCAAGATTATCGCGTTCGATGAGCCGACCAGCTCGCTCTCCGCCAGAGAGATCGACAACCTGTTTCGCGTCATCCGCGAGTTGCGGGAGGAGGGAAGGGTCATCATCTATGTTTCACACCGGATGGAAGAGATTTTTGCCCTCAGCGATGCCATCACCGTCTTTAAAGATGGCCGCTACGTTTGCACCTTTGACGATATGCCGAGCGTCAGCCACGACGCCCTGGTCCAGGCAATGGTAGGCCGCAATCTGGGCGATATTTATGGCTGGAAGCCTCGCCCCTATGGCGAGGAGCGGCTGCGCCTGGAGGAGGTCAAAGCGCCGGGCGTGCGCACGCCGGTTAGTCTCTCGGTGCGCAGCGGTGAAATTGTCGGTTTGTTCGGACTGGTGGGTGCCGGGCGCAGCGAACTAATGAAAGGCCTGTTTGGCGGCACGCAGATAACCGGCGGTCAGGTGTATATCGATGGCCAGCCCGTCTCCATTCGCAAACCGGCCCAGGCCATCCAGGCCGGGATGATGCTGTGCCCGGAGGATCGTAAAGCGGAGGGGATTATTCCGGTGCATTCCGTGCGCGATAACATCAATATCAGCGCCCGGCGCAAGCATATTCACGCTGGCTGCCTGATCAACAACGCCTGGGAGGCGGACAACGCCGATCAACATATCCAGTCGCTGAATATTAAAACGCCGGGCCCGGAACAGCTGATTATGAACCTGTCGGGCGGCAACCAGCAGAAAGCGATCCTTGGCCGCTGGCTGTCGGAAGAGATGAAGGTGATCCTGCTTGATGAGCCAACCCGCGGCATCGACGTCGGCGCCAAACATGAAATTTACAATGTGATCTATGGCCTCGCCGCCTCCGGTGTCGCCGTGGTGTTTGCCTCCAGCGACCTGCCGGAAGTGCTGGGCGTCGCTGACCGCATCGTGGTGATGCGCGAAGGACAAATCGCCGGCGAACTGCTGCATGAAGAAGCCAATGAACAGCAGGCGTTAAGCCTTGCGATGCCAACAGTGAGCCAGGCTGTCGCCTGA

>IR1230_00335

ATGCACAAATTTACTAAAGCGCTGGCGGCCATTGGTCTCGCTGCGGTTATGTCACAATCAGCTATGGCGGAAAATCTTAAACTGGGTTTTCTGGTGAAACAGCCGGAAGAGCCATGGTTTCAGACTGAGTGGAAATTTGCTGATAAAGCAGGAAAAGATTTAGGCTTTGACGTTATTAAAATTGCGGTCCCGGATGGAGAGAAAACCCTGAATGCCATTGACAGCCTGGCGGCCAGCGGCGCGAAGGGCTTTGTCATCTGTACCCCCGATCCCAAGCTCGGGTCGGCGATTGTCGCCAAAGCGCGTGGCTATGACATGAAGGTGATTACCGTCGATGACCAGTTCGTCAATGCGAAGGGCAAACCGATGGAGAGCGTACCGCTGGTGATGATGGCGGCGAGCGAAATTGGCGCCCGTCAGGGGCAGGAACTCTATAAAGAGATGCAAAAGCGCGGCTGGGATGTCAAAGACACCGCCGTAATGGCGATCACCGCCGATGAGCTGGATACCGCGCGCCGTCGCACCACCGGCTCCATCGATGCCCTGAAGGCGGCCGGCTTCCCGGACGCGCAAATCTACCGCGTGCCGACAAAATCCAACGATATCCCGGGAGCGTTCGACGCCGGTAACTCGATGCTGGTCCAACATCCGCAGGTTAAACACTGGCTGATCGTCGGGATGAACGACAACACCGTCCTCGGCGGCGTGCGCGCGACCGAAGGTCAGGGCTTTAAAGCTCCCGATGTGATTGGCATCGGCATCAACGGCGTGGATGCCGTCAACGAACTGTCGAAGGCGCAGCCGACCGGCTTCTATGGTTCTCTGCTGCCAAGCCCGGATATCCACGGCTACAAAACCAGCGAAATGCTATACAACTGGGTCACCAAGGGCGTCGAGCCGCCGAAGTTCACGGCGGTGACCGATGTGGTGCTGATTACCCGCGATAACTTCAAAGAAGAGCTGGCGAAAAAAGGGCTGTAA

>IR1230_00336

ATGGCAGTTCCAGGAATGGCACAAAAGCTAAACACCCAAATGAATCTTGAGTTTCACGCCTCTAACGTTTACCTGAACTTAAGCGAGTGGTGCGCCCGGCATCGCTTCGACGGCGCCGCGACGTTTCTGCGCACCCGCGCGCAAAGCAGCATCACCCTCACCATGCGCGTATTTGACTACCTGAAAAAAGCCGGCAGCTGGCCGATCGTCAATCCCGACCACGCCTGTAACCCTGAGTGCACCTCGCTGGAAGATCTGTTTACCCAAACCCTGAGCGATTACCAACAGCGCAGTCGTCTGCTGAGCGGTCTCGCTCAGGAAGCCAAAGCGCAAAGCGATGACAGCACCTGGCGGTTTCTCACGCTGTTGGCGGAAGAGCAGCAGCAGGATGGCCTGCTATTGCAAAGCGTGCTTGAGGAAATTCGTAATGCCGATAAAGCCGGTCTCGGGATGGAGCAGACCGATCGGCGTCTGATGGCTATCGTTGGAGAAGCGCAGAAAGCGCATTAA

>IR1230_00337

ATGGCTAGATACACGGCTTCAGCCTCTGAAGGGCTGCAGGGAAGACCGATCTTGTTACGCCATCAGTTAGCCTACGGCGGCGGAAATCTGTTAGGGAGCGGCGCACTGGCGATAAGCGGCGCATGGCTACTCTATTTTTATACGACCTTTTGCGGCCTGACGCTGATCGAGGCCTCATTTATCTTCTCCGTCGCCAGCATCATTGACGCTATCAGCAACCCGCTGATGGGCTATCTGACGGATAATTTTGGCAAAACGCGCTTGGGCAAGCGCTTCGGCCGCCGGCGTTTCTTTCTGCTAATCGGTATTCCGCTGATGATGTTCTACCCGCTGCTGTGGGTGGAAGGCCTGAGTTTCTGGTACTACCTTAGCACCTATGTGGTGTTTGAGATCATCTACACCTCTATCATGGTGCCTTATGAAACGCTGGCGACAGAGATGACCGATGATTTCTCACTGCGTTCAAAACTGACCGGCTATAAAGCTATCTTTGGTAAGCTGGCCAACTTCCTCGCGGCGTTTATTCCCGGCCAGTTCATTTTGCTGTATGGCAAAGATTCTGCCACCCCTTTCTTCCTCACCGGTCTGACCTATGGCGCGATCCTGATCGTCGCTATTTCCTGCCTGTGGTTGTGCAGCTGGGAGCGTGAGCGCGGGGAAGAGGTGGAAACCAGCGCGAAAAAAGGACTGCTGAGCACCCTGCTGTCGCTGGCGAAAGATATGCGTTCGACTTTTTATCTACGCGTATTCCGCAAACATCTTGGTATGTACCTGTGCGGCTTTGGCGCGGAATGGCTGTTTGCCTCCATCTTCACCTATTTCGTGATCTTCGTTCTACAACACGATCCAGCGATGGTGGCCGGGCTGAACAGCCTGAACTCGATTCTGCAGCTGATCTCTACGGCGCTGTTTATCGGCCTGTGCGTGAAAAAGGGCTTCAGCAAACCCTATATCCTGGCGCTGGGGATCGTGATTTTCGCGGTTCTGCTGTATACCTCGCTGTGGTTTTTCCATCTACCGTCCGGCCTGGCGACAGTGCTGATGTTCGGCATTACCGTGCTGTTCGGCCTGGGTACCGGCGGGGTTTACTATATACCCTGGACCGTTTACACCTTCCTGGCCGATGTCGATGAGATCTACACCGGACGCCGGCGGGAAGGGATCTATGCCGGCGCCATGACCTTCTCAGGAAAAATTCTGCGCTCGATTGTGGTGTTCTCGATGGGGGCGATCCTGAGCTTCTACGGTTTCCAGTCAAAAGCGCACAGCCAACCGGAAAGCGCGGTGACCGCTATCGCAGTGGTATTCTGCGTCGGGGTGATCGCATTAGCCCTGGCCGCCATCGTCTTCAGCAAGCAGATGAAGCTGGACCGGAAGGCCCATCTGGTGGTGCTGCAGGAAGTCGCGCGGATAAAAGCGGGCGGCAAAATTAGCGATATTGCGCCGGACGTGCGGGTTATCGTGGAGGATCTGGTCGGCCATCGCTACGAAGAGTGCTGGGGCAACAGCAAATTGTTCAAAGATGCTGCCCCTGCGCCTGCGCAGACCGTCGTTTCCCATTAA

>IR1230_00338

ATGTCTCAACATCTTACCGAACACGACGAGCTGGTTTCCGATGTGGTTGCCTGCCAGCTGGTTATCAAACAGATCCTCGATGTCATTGACGTCATTGCGCCGGTGGAAGTGCGTGAAAAAATGACCACTCAGCTGAAAGCTATCGACTTCGCCAGCCATCCGGCCTCTGCCGATCCGGTGACCCTTCGCGCGGTGCAAAAAGCGATCGCGTTGATTGAGCTGAAGTTTACTCCACAGGAAGAAACGCATTAA

>IR1230_00339

ATGAATACCGCGATCTCTTCCCGCGATGAACGTGCTTTTTCAGCGCCGGCGCTGCTGGTGGCCGGTGCCTTTTTTATGGAGTTTCTCGACGGGACGGTGATCGCCACCGCCCTGCCCGATATGGCGAGGGATTTTGGCGTCACCGCGGTCGAACTGAATATCGGCATCAGCGCCTATCTGATTACCCTTGCGGTCCTGATCCCGGCCAGCGGCTGGATCGCCGATCGCTTCGGCGCCCGGGCTATTTTTACTCTCGCCCTGGCGATCTTTACCCTTGCCTCGGTCTTCTGCGGCCTCTCCACCGAGGTGCATATCTTTGTCGCCATGCGTATCCTTCAGGGAGTCGGCGGCGCGCTGATGGTCCCCGTTGGTCGCCTTGCGGTGCTACGCACCACGCCGAAACACCAGCTGATCAAGGCCATCGCCACCCTGACCTGGCCGGCGCTGGTGGCCCCCATTATCGGCCCGCCTTTGGGCGGCTTTATAACCCGCTACGCCAGCTGGCACTGGATCTTCTTTATCAATGTCCCGCTGGGCCTTGCCGCCATCATCCTTTCGCTGCGCATTATCCCTGATATTCGCGAAACGGAACGGCGCTCCTTCGATCTCAGCGGATTTATCACTACCTCGGTCGCGATGGTCAGTCTGGTGACCGCGATGGAGCGGCTCGGCGATCGCCAGCCGCAGATCTGGCCGACGCTGGCTCTCGCCGCGCTTGGCTTCGGCTGTCTGTTATATTCCATTCGTCATTTCCGCCGGGCGGCGGCGCCTATGGTTCGTCTCGACGCCCTGCAGGTGCCGACGTTCCGGGTGACAATGTACGGCGGCTCGTTGTTCCGGGCCTCCATCAGCGCGGTGCCTTTTCTGCTCCCCCTGCTGTTCCAGGTGGGATTCGGTATGGATCCTTTTCATTCCGGCCTGCTGGTGCTGGCGGTGTTTGTCGGTAATTTAACCATTAAACCCGCCACCACACCGCTCATTCGCTGGCTGGGCTTTCGTCGGCTACTGCTGATCAATGGCGCGTTGAACGTCTGTTCGCTGCTGGCTTGCGCGCTGCTTACGCCGCAGACGCCGGTCTGGGCCATCATGCTGATCCTTTATCTCGGCGGGGTGTTTCGCTCAATCCAGTTTACCGGTGTAAGCACGCTGGCCTTCGCCGATGTCCCCGCCGCGCAGATGAGTGATGCTAATACGCTGTTCAGTACCGCGTCGCAGCTGGCGGTAGGGCTGGGGATCACGCTCGGGGCGATTGGCATTCGCCTGGGCGAGCAGGTCGGCGACTGGCTGCATCTTACTGAACTACCGGGGATCAGCTTCCGCCTGTCGTTTGTGTTTATCGCCTTGATCTGTCTGGTAGGGATGATCGACAGCCTGCATCTGGCCAAAACGGCCGGCAGCAGCGTGTCGGAGAAGAAGAAATAG

>IR1230_00340

ATGAAGATTGCACTGATGATGGAGAACAGCCAGGCGAACAAAAACGCCATCATCCTCAAGGAGCTTAACGCGGTTGCCGACGAAAAAGGATTCCCGGTCTATAACGTCGGCATGAGCGATGAGAACGATCATCATCTGACCTACATCCATCTTGGGATCATGGCCAGTATTCTGCTGAATTCGAAAGCAGTTGACTTCGTGGTCACCGGCTGCGGTACCGGTCAGGGGGCGCTGATGTCGCTGAACATCCATCCGGGCGTGGTCTGCGGCTACTGTATCGATCCGGCGGATGCGTTCCTGTTTGCCCAGATCAACAACGGTAATGCGCTGTCGCTGCCGTTTGCCAAAGGCTTCGGCTGGGGCGCCGAACTGAATGTGCGGTTTATCTTTGAAAAAGCCTTTACCGGCCGCAACGGCGAAGGCTATCCGCCGGAGCGCAAAGAACCGCAGGTGCGCAATGCCGGGATCCTCAACCAGGTCAAAGCAGCAGTAGTAAAAGAGAACTACCTCGATACCCTGCGCGCCATCGATCCGCAGCTGGTGAAAACCGCAGTCTCCGGCCCACGCTTCCAGCAGTGCTTCTTCGAAAACTGCCAGGATAAAGCAATCGAAGACTTTGTTCGCCAGATCGTCGCTTAA

>IR1230_00341

ATGCTGAAAACGGACATGATTGACAAACTCAATGAACAGATGAATCTTGAACTCTATTCTTCTCTTCTTTATCAGCAGATGAGCGCCTGGTGCAGTTACCACAGTTTTGAAGGCGCCGCGGCCTTCCTGCGTCGCCATGCTCAGGAAGAGATGACCCATATGCAGCGCCTGTTCGACTATCTAACCGACACCGGCAGCCTGCCGCGGATCAACGCTATCGCCTCCCCGTTTGCCGAATATGCCTCACTCGACGAGCTGTTCCGCCAGACCTACGAGCATGAGCAGCTGATCACGCAAAAAATAAATGAACTGGCGCACGCGGCCATGACCAGCCAGGATTACCCGACCTTTAATTTCCTGCAGTGGTATGTTGCTGAACAGCATGAAGAAGAGAAATTATTTAAATCGGTGATCGATAAACTGACCCTCGCTGGGAAAAGCGGCGAAGGTCTGTACTTTATCGATAAAGAGCTGGCTACCCTCGACGCGCAGAATTAA

>IR1230_00342

ATGTCTTCTATTCATGGTCATGAAGTTTTACAGATGATGCTCGCCTCGGGTGAGTCCTGGACGGTCGCCTCGCTGGAGGCCGCCATTCGCCGCCGCTTTGGCGAAGAGGCGCGGTTTCACACCTGTTCAGCAGAGAATCTAAGCGCCGCACAGTTGGTGGCGTTTTTAGAGAAAAAAGGCAAATTTATTGCCAGAGAGGAAGGTTTTACTACCGCAGAAAATAAGATCTGCCGACATTAA

>IR1230_00343

ATGCCATTGGCCGCCGCAGGCGTCGGCTTTACCGTCACGTTGGGTTTACTTTTCACCCTGTGGGCATTGATGTGCTATACCGCCCTTTTGCTGCTGGAAGTCTACCAGCATGTCCCTGCCGATATGGGGCTCGGTTCTCTGGCGGCGCGCTATCTGGGCCGTTATGGGCAGTGGGTTACCGGATTCTGTATGCTGTTTCTGCTGTACGCCCTCACGGCAGCCTATATCAGTGGCGCCGGTGAATTACTCGCTTCCAGCCTCAACCAGTGGCTGGACTGGCAGCTACCGCCGGCCGCAGGCGTACTGATCTTCACCCTCCTCGGCGGGGCGGTAGTGTGCATTGGCACCGCGCTGGTGGATCTGTTCAATCGCTTCCTGTTTAGCGCCAAAATCGTCTTCCTGGTGATTATGCTGGCCCTGCTGATGCCGCACATTCATCAGGTGAACCTGCTGACGCTGCCGGTTGAGCAAGGCCTGGCCCTCTCGGCCATTCCGGTGATTTTCACCTCCTTTGGTTTTCACGGCAGCGTCCCGAGCATCGTCAGCTATCTGGGGGGGGATATTCGCAAATTGCGCCGGGTATTTATCATCGGCAGCTTTATTCCTCTGGTGGCCTATATTTTCTGGCAGCTGGCGACGCTGGGCAGTATCGACGCCCCGGCCTTTACCGCCATGCTGGCAAATAACGCTGGTCTGAACGGCCTGCTGGAGGCGATCCGCGAAGTGGTGGCGTCACCGCATGTGGAGCTGGCCGTTCACCTGTTTGCCGACCTTGCGCTGGCGACGTCATTCCTCGGCGTCTCGCTCGGCCTGTTTGATTATCTGGCGGATATGTTTCAGCGTAAAAACAGCGTCGGCGGGCGTCTGCAGAGCGGGATAATCACCTTCCTGCCGCCGCTGGCGTTCGCCCTGTTCTATCCCCGCGGTTTCGTCATGGCCCTCGGCTACGCCGGGGTGGCATTAGCCGTGCTGGCGCTGATAATGCCGGCTCTGCTGGTCATGAAGAGTCGCCGAGAGCATCCGCAGGCTACCTGGCGGGTCGCCGGCGGCGCACCGACGCTATGGCTGGTTCTGCTGTGCGGGATCGGCATCGTGGCTATTCAGTTCAGCATCGCTGCCGGGTTGTTGCCCGCGGTGGGATAA

>IR1230_00344

ATGAATACCGGACCTTTGAACGAAAACGAACTGGAATGGCTGGATGACACTCTGGCGAAATACGCCGCCGAAGGCGCCATTCTGGATGTCTCTGAACTGGATGGCCTGCTGACGGCGATCCTCTCTGCGCCGACCGACATCGAACCCGCTCAGTGGCTGCTGGCGATTTGGGGCGGGGCGGATAATGTCCCGCGCTGGGCCAACGATCGCGAGCGCGATCGCTTTGTCAATCTGACGCTGCAGCATATGAGCGATATCGCCGAACGGCTGGAGTCTTATCCCGATCAGTTCGAACCGCTGTTCGGCACCCGCGAAGCGGAAGGTCAGGAGCTGACCATAGTCGAGGAGTGGTGCTTTGGCTATCTGCGCGGCGTGGCGCTGAGCGACTGGTCGACGTTGCCCGCTGAACTCCAGCCGGAGCTGGACGCTATCGCGCTCCACGGCAGCGAAGAACAGTTTAGCGCGCTGGATAATCTGACGGCGGATGAATTTATCGCCAGCATCGAGCGTATCACGCCGGCGGCGCTGGCGCTTTACCAGTACTGGATAGCGAATCCGCAGCCGGTGGAGGCCCCGCAGCCAATAAGAAATGAAGCGAAAGTCGGACGCAACGATCCCTGCCCATGCGGCAGCGGTAAAAAGTATAAGCAGTGTTGCTTAGCGAAATGA

>IR1230_00345

ATGTTCAGTGGACTTTGCGCATTTCCTTTGACACCTTTGCATCAGCAAGATTTTGATGAAAAAGCGTTTATTCGTATTCTCGCCCGTCTTACCGATGCCGGAGTCGACTCTCTGGGCATCTTAGGTTCAACCGGTAGCTACGCTTATCTCAGCCGTGAGCAGCGCAGACGAGTGGTGCAGGTGGCGAAGGCGCATGCGGGGTCGATCCCGATGATGGTCGGCGTCGGCGCCATTGCGACTAACGAGGTGCTACGCCTGGTGGAGGATGCTCAGGAGGCAGGGGCGGATGCGTTATTACTGCCGATGATGTCCTATCAGCCGCTTTCCGCGGAGGAGATCTTTGCCTTTTACGAAGAAGTCTGTCGGCATGTCTCGGTGCCGGTTTGCCTGTATGACAATCCCCGCACTACCCACGTAATGCTGGCGGATGAGCTGCAGGGGCGCATCGCCGCCTTGCCGGCCATTGCCTCGATTAAAATTCCCGGCCTGCCCGCGCCACAGGCGAGCGAACGTGTAGCGGCCCTGCGTCAGCATCTTCCCTCTCGCGTTACGCTTGGCGTCAGCGGCGATGCGTGGGCCACTGCCGGGTTGCAGGCCGGCTGCGAAGCCTGGTATTCGGTCTGCGGCGGACTCTTTCCCCGCTGCTCACTGGCGCTGGTAAGAGCGATACGTAGCGGCGATGTGGCGCAGACTGCGGCACTGAATGAGCAGCTGGCCCCCCTATGGCGATGCTTTGATCGCTATGGTGGTAGCCTGCGAGTGATAGCCAGCGCCGCAGCGATGCTCGGACTCTGCGATCCAGACTCGCTTCCCCGTCCTTTGCTTTCGTTAGGGGAGGAGGCCTGTCGTGAGGTGGCGTCGGCGCTGCGCGGCCTGGCCTGA

>IR1230_00346

GCCCGGATGGTGGAATCGGTAGACACAAGGGATTTAAAATCCCTCGGCGTTCGCGCTGTGCGGGTTCAAGTCCCGCTCCGGGTACCA

>IR1230_00347

GGCGCGTTAGCAAAGCGGTTATGTAGCGGATTGCAAATCCGTCTAGTCCGGTTCGACTCCGGAACGCGCCTCCA

>IR1230_00348

GCGGGAATAGCTCAGTTGGTAGAGCACGACCTTGCCAAGGTCGGGGTCGCGAGTTCGAGTCTCGTTTCCCGCTCCA

>IR1230_00349

ATGCAATTTAATATCCCTACGTTGCTTACTCTGTTTCGCGTCATCCTGATCCCGTTTTTTGTGCTGGCGTTTTATCTGCCCTTTAGCTGGGCGCCGTTTGCCTGCGCGCTGATCTTCTTTGTTGCCGCGGTAACCGACTGGTTTGATGGTTTCCTCGCTCGTCGCTGGAATCAGAGCACCCGTTTTGGCGCCTTCCTCGACCCGGTTGCCGATAAAGTCATGGTGGCTATCGCCATGGTGCTGGTTGCCGAACACTATCACACCTGGTGGGTCACGCTACCGGCGGCGACCATGATTGCCCGTGAGATCATTATCTCCGCGCTGCGTGAGTGGATGGCGGAGCTGGGGAAACGCAGCAGCGTGGCGGTCTCGTGGATTGGCAAAGTGAAAACGACCGCCCAGATGACCGCCCTGGTATGGATGCTGTGGCGTCCTTATCCGTGGGTGGAGTGGGCCGGGATCGCGCTTTTCCTCGTGGCGGCGGTGCTGACGCTGTGGTCGATGCTGCAGTATTTGAACGCGGCGCGCGGCGATTTGCTTGATCAGTGA

>IR1230_00350

GTGAGTGATGTTTTTGACGCAAAAGCTTTCCTGAAAACGGTGACCAGCCAGCCCGGTGTGTATCGGATGTATGATGCTGGCGGAACCGTCATCTATGTTGGCAAAGCCAAAGATCTGAAAAAGCGGCTCAGTAGCTACTTCCGCAGCAACCTTGCGTCGCGTAAAACGGAAGCGCTGGTAGCGCTGATCGCGCAAATCGACGTGACCGTCACCCACACCGAGACGGAAGCGCTGCTGCTTGAGCACAACTATATTAAGCTGTACCAGCCACGTTACAACGTGCTGTTACGCGACGATAAATCGTATCCTTTTATTTTTCTCAGCGGCGATACTCATCCGCGTCTGGCGATGCATCGCGGCGCCAAACATGCGAAGGGCGAATATTTTGGTCCTTTTCCCAACGGCTATGCCGTGCGCGAGACCCTGGCGCTGCTGCAGAAAATCTTTCCCATCCGTCAGTGTGAAAACAGCGTCTACCGCAATCGCTCCCGCCCCTGTCTGCAGTATCAGATTGGTCGCTGCCTGGGGCCGTGCGTGGCCGGGCTGGTCAGCGAAGAAGAGTATGCTCAGCAGGTAGAGTATGTGCGGCTGTTTCTCGCCGGCAAGGATGACCAGGTGCTGACCCAGCTCATCGCGCGGATGGAGAAGGCGAGCCAGAACCTGGAGTTCGAAGAGGCCGCGCGCATCCGCGACCAGATTCAGGCCGTCCGCCGCGTGACGGAGAAACAGTTTGTCTCCAACACCGGTGACGACCTCGACGTGATCGGCGTCGCCTTCGATGCCGGCATGGCCTGCGTGCATGTGCTGTTTATCCGTCAGGGCAAAGTGCTGGGCAGCCGAAGCTACTTCCCGAAAGTGCCGGGCGGCACCGAGCTGGGAGAGGTCGTGGAGACCTTTGTCGGCCAGTTCTACCTGCAGGGCAGCCAAATGCGCACGCTGCCGGGGGAGATTTTGCTCGACTTTAACCTTGGGGATAAGACGCTGCTGGCCGACTCGCTGTCGGAGCTGGCGGGTCGTCGAATCAATGTGCAGACTAAGCCGCGCGGCGACAGAGCGCGTTATCTTAAGCTGGCGCGGACCAACGCCGCGACAGCGTTAACGACCAAATTGTCTCAGCAATCGACCATTCATCAGCGTTTGCAGGCGCTGGCCGGCGTACTGGAGCTGCCGGCCGTCAAGCGAATGGAGTGTTTTGACATCAGCCACACCATGGGCGAACAGACCGTAGCATCCTGTGTGGTGTTTGACAGCAATGGCCCACTGCGCGCGGAGTATCGGCGCTACAACATCACCGGCATTACCCCTGGCGATGACTATGCGGCGATGAATCAGGTCCTGCGCCGTCGCTATGGTAAAGCGATTGATGACAACAAGATCCCGGATGTGATCCTGATAGACGGCGGCAAGGGACAGCTGGCCCAGGCGAAAGCCGTGTTTGCTGAACTGGACGTCCCCTGGGATAAACATCATCCGCTGCTGCTGGGCGTGGCGAAAGGTAGCGATCGAAAAGCGGGTCTGGAAACGTTATTCTTTGAGCCGGAAGGGGAAGGGTTTAGCCTGCCGCCGGATTCGCCGGCCCTGCATGTGATCCAGCATATTCGCGATGAATCGCACGATCATGCGATCTCCGGGCATCGCAAAAAACGGGCGAAAGTGAAGAGCACCAGCTCGCTTGAGACTATCGAGGGGGTGGGGCCGAAGCGTCGTCAGATGCTGCTGAAGTATATGGGTGGGCTGCAGGGGCTACAGCAGGCCAGCGTCGAAGAAATTGCTAAAGTACCGGGCATCTCTCACGCTCTGGCAGAAAAGATCTTCTACTCGTTGAAACATTAG

>IR1230_00351

TTGATCAACGTTCTTCTTGTTGATGACCACGAACTGGTGCGCGCAGGGATACGACGCATTCTGGAAGATATAAAAGGGATTAAAGTCGCTGGCGAAGCCTGCTGCGGCGAGGACGCCGTCAAGTGGTGCCGGGCTAATTCCGCAGATGTCGTCCTGATGGACATGAACATGCCTGGGATCGGTGGTCTTGAAGCGACGCGCAAAATCGCGCGCTCCGTGGCGGGCACCAAAGTGATCATGTTGACCGTGCATACCGAGAATCCTTTACCTGCCAAGGTCATGCAGGCGGGCGCGGCAGGCTATCTCAGTAAAGGCGCCGCGCCGCAGGAAGTGGTGAATGCCATCCGCTGCGTCGCGTCCGGGCAGCGCTATATTGCCTCCGATATTGCCCAGCAAATGGCGTTAAGTCAGATTGAACCCGAAAAGACAGAATCGCCGTTTGCCAGTTTGTCTGAACGCGAATTGCAGATTATGCTGATGATCACCAAAGGTCAGAAGGTGAATGAGATCTCAGAGCAGCTAAATCTGAGTCCTAAAACGGTTAACAGCTACCGCTATCGGATGTTCAGTAAATTAAACATTCATGGCGATGTCGAGCTGACCCACCTGGCAATTCGCCATGGCCTGTGTAATGCGGAGTCGTTAGCAAGCCAGTGA

>IR1230_00352

ATGAGCACACCAGATTTTTCCACTGCAGAAAATAAGCAGGAACTGGCGCAGGAAGTCTCCTGCCTGAAAGCGATGATTACGCTGATGCTGCAGGCGATGGGCCAGGCGGATGCGGGGCGCGTGATTATTAAAATGGAAAAGCAGATTGCACAGATGGAAGATGAGGCTCAGGCCGCCGTATTCTCCAGCACCGTTAAGCAGATTAAACAAGCCTATCGGCAGTAA

>IR1230_00353

ATGAGGGACAATGATTTTTTCAGCTGGCGGCGGGATATGCTGCACCAATTTCAGTCCATGGCCACCGGCGAGGAGGTTTATAATCTCCTGCAACGTGAAACCGAAGCGCTGGAATATGATTACTACACGCTTTGTGTGCGCCACCCGGTGCCTTTCACCCGCCCCCGCGTTACGTTCCAGTCGACTTACCCCCGCGCATGGATGTCGCACTATCAGGCAGAAAATTATTTCGCGATAGATCCTGTGTTACGGCCGGAGAATTTTATGCGTGGCCATCTGCCATGGAACGACAGCCTGTTTCGTGATGCGCCGGCACTGTGGGACGGGGCGCGTGACCATGGTCTGCAAAAGGGCGTGACGCAGTGCCTGACGTTGCCAAACCATGCTCAGGGATTTCTCTCTGTCTCCGCGAACAATCGTCTGCCGGGCGGTTACCCTGAAGATGAGCTTGAGCTGCGTCTGCGAACGCTGACTGAACTAAGCTTGCTGACGCTGCTGCGTCTGGAAGATGAGATGGTGATGCCGCCGGAGATGAAGTTCAGCCGCCGCGAACTGGAAATCCTCAAATGGACGGCGGAAGGAAAGACTTCGGCGGAGGTGGCGATGATTCTCTCGATTTCGGAAAATACGGTGAATTTCCATCAGAAGAATATGCAGCGGAAATTTAATGCGCCGAATAAAACGCAAATTGCCTGCTACGCCGTGGCGACTGGGCTAATCTGA

>IR1230_00354

ATGAGTGCGATAGAAGTGAAAAGCCTGGTAAAAAAATTCCATGGCCAGACGGTGCTCCATGGTATCGACCTGGAGGTACAGAAAGGGGAAGTGGTGGCGATCATCGGCCCCAGCGGCTCGGGCAAAACCACCCTGCTGCGTAGCATCAACCTGCTGGAGCAACCGGAGAGCGGCACTATCCGCGTCGGCGATGTGACGATCGACGCCGGTCGCAGCCTCGGGCCGCAGAAAGGGCTCATTCGCCAGCTGCGCCAGCACGTCGGCTTTGTCTTCCAGAACTTTAATCTGTTTCCCCACCGTACGGTGCTGGAGAACATTATTGAAGGGCCGGTCATCGTAAAAGGTGAAGATAAGCAGGAGTCCATGGCGCGGGCGCGCGAACTGCTGGCGAAGGTGGGGCTCAGCGGAAAAGAGAATAGCTATCCGCGCCGTCTCTCCGGCGGGCAGCAGCAGCGAGTGGCTATTGCCCGCGCGCTGGCTATGCGTCCGGACGTGATCCTGTTTGATGAACCCACCTCGGCTCTCGATCCTGAGTTGGTGGGGGAGGTGCTCAACACCATCCGTCAGCTGGCGCAGGAAAAACGCACCATGGTGATCGTGACCCATGAAATGAGTTTCGCCCGCGACGTTGCCGATCGGGCGATATTTATGGATCAGGGACGCATTGTCGAACAAGGCGAGGCAAAAGCGTTGTTCGCTTCGCCCCAGCAGCCGCGTACCCGTCAGTTCCTTGAAAAATTCCTGATGCAGTAA

>IR1230_00355

ATGCAAGAAAGTATCCAACTGGTCATTGATTCCGCCCCTTTTCTGCTGAAAGGGGCCGTCTTTACGCTGCAGCTGAGCATTGGCGGGATGTTTTTCGGCCTGGTGCTGGGCTTTATCCTGGCGCTGATGCGCATGTCGCCAGTCTGGCCGGTAAAATGGCTGGCGCGCATGTATATCTCCATTTTCCGCGGCACGCCGCTTATCGCCCAGCTGTTTATGATCTACTACGGCTTGCCGCAGTTCGGCATTGAGCTTGACCCGATCCCGGCGGCGATGATCGGCCTGTCGTTGAATACTGCGGCCTATGCGGCGGAAACGCTGCGCGCGGCCATCGCCTCGATTGATAAAGGGCAATGGGAAGCGGCGGCCAGTATCGGTATGACGCCCTGGCAGACGATGCGTCGGGCCATTTTACCCCAGGCGGCGCGGGTCGCTTTGCCGCCGCTGTCGAACAGTTTTATCAGCCTGGTAAAAGATACCTCGCTGGCGGCAACGATCCAGGTGCCGGAGCTGTTTCGTCAGGCGCAGCTAATCACCTCGCGAACCCTGGAGGTCTTCACCATGTATCTGGCGGCCTCCCTGATCTACTGGGTAATGGCGACAGTGCTCTCCTCGCTGCAGAATTATTTTGAAAACCAGCTGAACCGCCAGGAGCGTGATCCGAAATGA

>IR1230_00356

ATGTCACTGCAAAACTTAACCCGCTTTCCGCGCCTGGAATTGATCGGCGCGCCGACGCCGCTGGAGTATCTCCCGCGTCTGTCCGATCACCTTGGGCGCGAAATTTTTATTAAACGTGATGATACGACGCCGCTGGCGATGGGGGGCAACAAACTGCGTAAGCTGGAGTTTCTCGCCGCCGATGCGCTGCGCGAGGGCGCGGATACCTTAATCACCGCCGGCGCGATCCAGTCTAACCATGTGCGTCAGACGGCGGCAGTCGCTGCGAAACTGGGACTGCATTGCGTGGCGCTACTGGAAAATCCCATCGGCACCCGGGCGGAGAACTATCTGAGCAATGGCAACCGTCTGCTGCTGGATCTCTTTAACACCCAGGTTGAGATGTGTGATGCCCTGACCGATCCCGCCGCCCAGCTCGATGAGCTGGCGACCCGCATTGAGGCGCAGGGGTATCGTCCGTATGTTATTCCGGTCGGCGGCTCCAATGCCCTCGGCGCGTTAGGCTATGTGGAAAGCGCACTGGAAATCAGCCAGCAGTGCGAAGACGCGGTGGCGATCTCGTCAGTCGTGGTCGCCTCCGGCAGCGCCGGCACCCATGCCGGATTGGCGGTCGGCCTTGAACAGCTGATGCCGCAGGCGGAACTGATTGGGGTGACCGTGTCGCGCTCGGTCGCTGACCAGTTGCCGAAAGTGGAGGCGCTGCAGCGGGCCGTGGCCAACAGTCTCGAGCTACAGGCGAAGGCTGAAATTATACTGTGGGATGACTACTTTGCCCCAGGATACGGTACGCCAAACGAAGATGGGATGGCCGCAGTGAAGCTGCTGGCGCAGCTGGAAGGTATTTTGCTCGACCCGGTCTATACCGGCAAAGCGATGGCCGGGCTTATCGACGGCATCACGCAGAAGCGCTTTAAGGATGAAGGGCCGATACTGTTCGTCCACACCGGCGGAGCGCCGGCGCTGTTTGCCTATCATCCTCACCTCTAA

>IR1230_00357

ATGGGCGCGATGGCCGTTGTTTTGATGGCTGGCGTCAGCGTGAAAACTTTTGCGGCGGAAAACCTGCTCAATCAGATTAAAGAGCGCGGCACCCTACGCGTGGGCCTCGAAGGCACCTACCCTCCCTTTAGTTTTCAGGGGGATGACGGCAAGCTGACCGGGTTTGAAGTGGAATTCGCTAACGAACTGGCGAAACATCTTGGCGTGAAGGCCGATCTCAAACCGACCAAGTGGGACGGTATGCTGGCCTCGTTGGACTCCAAGCGTATCGACGTGGTGATTAACCAGGTCACTATCTCTGACGAACGTAAGAAGAAATACGATTTCTCCACCCCATATACCGTCTCCGGCGTGCAGGCGCTGGTGAAGAAGGGTAACGAAGGCGTAATTAAAACCGCGGCGGATCTGAAAGGCAAGAAAGTGGGCGTCGGACTGGGCACTAACTATGAAGAGTGGCTGCGTCAGAATGTCCAGGGCGTGGACGTCCGTACCTATGATGACGATCCCACCAAATACCAGGATCTGCGTGTAGGCCGTATTGACGCCATTCTGGTAGACCGTCTGGCGGCGTTGGATCTGGTGAAGAAAACCAACAATACGCTGGCGGTAACCGGCGAAGCCTTCTCTCGTCAGGAAGCTGGCGTTGCGCTGCGTAAAGGCAACGACGACCTGCTGAAAGCGGTAGATGGCGCGATTGCAGACATGCAGAAAGATGGCAGCCTGAAGGCGCTGTCCGAGAAATGGTTTGGCGCTGACGTGACGAAATAA

>IR1230_00358

ATGAAAAACCCCACCTTATTGCAATGTTTTCACTGGTACTACCCCACCGGCGGCGAGCTGTGGCCTGAGGTCGAAGCGCTGGCGCCCAGCCTGAATGAAATCGGCATCAATATGGTCTGGTTGCCACCCGCCTACAAAGGCGCCTCGGGCGGTTACTCCGTCGGCTATGATACCTACGATCTCTTTGACCTCGGCGAGTTTGATCAAAAAGGGTCTGTCGCCACCAAATATGGCGACAAAGCGCAGTTGCTGGCGGCGATCAACGCCCTGAAGGAGCACAATATCGCCGTGCTGCTGGATGTGGTGGTCAACCATAAAATGGGCGCTGATGAAAAAGAGGCTCTGCGCGTTCAGCGCGTCGATGAGCAGGATCGTACGCAAATTGACGAGGAGATCATTGAGTGCGAGGCATGGACGCGCTACACCTTCCCGGTGCGCGCCGGACAGTATTCGCAGTTTGTCTGGGACTACAAATGTTTTAGCGGCATTGACCACATCGAGAACCCGACGGAAGACGGTGTGTTTAAAATCGTCAACGATTACACCGGCGAAGGCTGGAATGAACAGGTCGATGATGAGCTCGGCAACTTTGACTATCTGATGGGCGCCAATATCGATTTCCGCAATCATGCAGTGACCGAGGAGATCAAATACTGGGCGCGCTGGGTAATGGAGCAGACCGGCTGCGACGGTTTTCGTCTTGACGCGGTCAAGCATATCCCCGCCTGGTTTTATAAAGCGTGGATCGAGCATGTTCAGGAGGTGGCCCCGCAGCCGCTGTTTATTGTAGCGGAATACTGGTCCCATGAAGTGGAGAAGCTCCAGCAGTATATCGACCTGGTCGAGGGCAAAACGATGCTGTTTGATGCCCCACTGCAGATGAAATTTCATGAAGCGTCGCGCCAGGGCCGCGATTATGACATGAGCCAGATTTTCAGCGGGACGCTGGTCGAAGCCGACCCTTTCCATGCCGTCACTCTGGTCACCAATCATGATACGCAGCCGCTGCAGGCCCTCGAGGCGCCGGTTGAACCCTGGTTCAAGCCCCTCGCCTATGCGCTGATCCTGCTGCGGGAGAATGGCGTGCCCTCGGTCTTTTATGCCGATCTCTTCGGCGCTTCCTATGAGGATACCGGCGGCGACGGAGAGACCTATGCCATTGAGATGCCCGTTATCGAACAACTCCACGAGCTGATCGACGCCCGTCAGCGTTTTGCCCATGGCGTGCAAACCCTTTGGTTCGATCATCCTAACTGCATCGCCTTTAGTCGTAGCGGTACTGACGACGATCCAGGCTGCGTGGTGATCATGTCCAATGGCGATGAGGGCGAAAAGACGCTGACGCTGGGCGAAAACTATGGCTACAAACGCTGGCGGGATTTTCTCGGTAACCGGGAAGAGATTGTCGAAACCGATGGCGAAGGCTGCGCGACCTTTACTTGTAACGGGGGGAGCGTGAGCGTGTGGGTATTAGAGGAGGTGCTGTAG

>IR1230_00359

ATGAAAAAGTTATTTTTTGCTGGCATGGTGGTGGCGCTGGCCGGTTGCGTACAGGTTGATCGTTATGAGGACGTGGTTAAAGCGCCGGCGCCGGCTGGCCTTGCAGGTTTCTGGCAAACGAAAGGGCCGCAGAGCGCGATGATGAGTCCTGACGCCATTGCCAGCCTGATTGTCACCAAAGAAGGGGACACCTTCGACTGCCGCCAGTGGCAGCGCGTGATTGCGCAGCCGGGGAAACTGATGAATCGTGACAGTGAGATCTACAACGTGACCGCTTCCCTGGACATCTATCCTGTCGAGCGCGAGGGCAACACGATCTCCTACGATCGGATGACGTTGTCCCGTGTTGAACGTCTCACTCCGGAGTGCGAGAAGGCGTGGGCCAAAGCGCGAGCGACGGGGCCCGTCAGCGCGCCGGCCTCCACGCGCTGA

>IR1230_00360

ATGTCAACGATGATTATGGATTTGTGCAGCTATACCCGGTTGGGATTGACGGGATATCTGACCAGTCGGGGAATTAAAAAACAGGAAATCGTTGAGGTCAACAATGCTGCGGATCTGCAGAAACACTGTACGTCGTGTTGCCCGGCGGTGGTGTTTCTGAATGAAGACTGTTTCGTGCATGATGATGAAAGTAATGGCATTATTCGCCAGATCATTACGCAAAACCCGGCGACGCTGTTTGTTATCTTTATGTCGCTGGCGAACATCCATTTTGACCGCTATTTGCGGGTACGGAAGAATCTGCTAATCAGTTCAAAATCGATAACCCCAAAAGACCTTGATGTTATTCTGGTTAATTATCTTAAATACAAAAACACCAGTGTAGGGCAGTTAACTTTACCGACATTGTCACTGAGTAAAACAGAATCAAATATGCTGCAAATGTGGATGGCCGGGCATGGTACTTCGCAAATCTCAACGCAAATGAACATCAAAGCGAAGACGGTATCGTCGCATAAAGGCAATATTAAAAAGAAAATACAAACGCATAATAAGCAGGTGATTTATCATATCGTTCGGCTGACCGAAAACATCACCTCCGGTATTCAGGTAAATATGCGCTGA

>IR1230_00361

ATGCAGGTGAACGATCGTGTCACGGTGAAGACCGACGGTGGCCCACGTCGATCGGGGGTAGTGCTGGCGATTGAGTCGTTTAGTGAAGGAACGATGTATCTGGTTTCACTGGAAGACTATCCTCTGGGGATCTGGTTTTTTAATGAAATAGGCCACCCGGATGGGATTTTCGTGGAAAAAGAGGTCTCGTAA

>IR1230_00362

ATGATGAAGCACCAATTAACAGGCCAGACCGGGGACGACCTTGACGTCAACGTAGATGCCCTGCTGGCGGCGATAAATGAGATCAGCGAAAGCGAAGTCCATCGCACGCTGGACGACCCGCAGCGCGCCAGCATCGATGGCCGCGGGTCACATACCTGGCGTGAGCTGGCGGAGGCATTCGAACTCGATATTCACGATTTCAGCGCCAGTGAAGCCAACCGTTAG

>IR1230_00363

ATGGATAAGCCACTGCGGTTGAAGTTAAAAAAGTGCTACCACCTGGCTCGCCCGGCGCATGTGGTTAACGCTTGTTTTATCGCAGTGGTGTTTTTCTCCACGCTGCTGATCTGGCGTGAAATCAATGACCTGGAAGAGGCCTACGTGGCCAACCAGCGTAACAATCTGTCTAACGTCGCTCATGAAATGGATGGACTGTTGCAGTTCAACATCGACAGAATGATGTTTTTTCGCCATGGGATGCAGGCGGCGCTCGAACAGCCGCTTGATATCGATGTATTGCGTAAGGCCAGCCAACGCTATCTGAGCCAGCGCCATCAGCAGGCCTGGCGGGTGGCGCTACCGAATCGGCGTACGCTGCCGGTGTTTGGCATCTCAGGCAGCGTCGCTGGCCACAACCCTATCCTGCTGGTGGACGATCCGCTGGCGGCTGATGAACTAATGGCGACGCTCGAGCTGGGTTATCTGCTAAATCTGACGCAGCACGATCGCGATTTCGCCGAGCGGATGCAGTATATCTCTCGTAGCGGCTTTTTTACCTCGACCCTGCCGCTGCGGGATGAGTCGCAGGTGATTACGCACTATTCCCAAGCCCTCGGCGCTCCGTGGTTTACCCGCCAGACCCAACGAAATAATCCGGGGCGAGGCGTTATCTGGCAAACCTTTCCTGACGATGACCCGCAGCTGGAAGAGCAGGTGGTCACCGCTTCGATACCGTTGGATTTTGCCGGTTACTGGCGCGGCGTGCTGGCGATGGATTTCTCAGTCAGTGAAATCAAAGCGTTTTTGGTTAGCGCCATGCAGGGGGGACAAGAGGGAGAGTACCAACTTTATGATAGCCATTTGAACCTGCTGGCTTCTTCCGCGCCGGGGAATGTCCTGACCCTGCTGTCCCCGCGCGAGCAGGAGCTGCTGAGCCGCGCGTTTGTCCATGATAACCAGGGAGGGCTGCGTCTGCTGACGCGCTATATCAGCTGGGCCAAACTGCGCAATTTTGACGGCGTGCTGCTGCGGATCCACACTCTGCGGGAAGGCGTCCGCGGGAACTTTGGCACCATCACCATCGCCCTGACCCTGATGTGGGTCCTCTTCACCTTGATGCTGTTGCTCTCCTGGCTGGTGATCCGCCGCATGGTCCGCAACATGAGCGTTCTGCAAACCTCGCTGGAGTGGCAGGCATGGCACGATGCCTTGACTCGCCTGCTCAATCGCGGGGCTCTGTTTGAACAGGCGATGGCGGTAGCCAGCGACTGCCAGCGCAGCGGACGGCCGCTGGCGGTGATCCAGCTCGATCTGGATCATTTTAAGCACATCAACGATCGCTATGGTCATCAGGCGGGGGACAGGGTGCTGTCGATGGTGGCCAGTACGCTGTCCAGCGCGGTTCGGCAGGGCGATCTGCTGGGCAGAGTGGGCGGAGAAGAGTTTTGTATCGTGATGCCGAATACCACCCTGCAGGAAGCGGCGGCGGTGGCGGAGCGATTGCGCCAGCGTATCCAGGGACGGGAAGTCTTTTTGCATAACAACGTGACGCTGCGGGTTAGCGCCTCGTTAGGGGTGAGCGCCAGCGAAGAGCGGGGGGAGTATCAGTTTGAAGCGCTGCAGTCGGTGGCCGATGGGCGACTCTATCTGGCCAAGCAAAATGGGCGTAACCAGGTCTGTTTTCGCAGCGCAGCGTGA

>IR1230_00364

TTGGCAGGAAGTAGTCTTTTAACATTACTTGATGATATCGCCACGCTGCTGGACGATATTTCCGTGATGGGCAAGGTAGCGGCGAAAAAAACGGCGGGGGTGCTGGGCGACGATTTGTCTCTCAATGCCCAACAGGTTACCGGCGTGCGGGCGAATCGCGAGTTGCCCGTGGTCTGGGGAGTGGCGAAAGGGTCGTTCGTTAACAAAGTGATCCTCGTGCCACTGGCTTTGTTGATCAGCGCCTTCATCCCCTGGGCGATTACGCCATTGTTGATGCTGGGCGGCGCGTTTCTCTGTTTCGAAGGGGTGGAGAAGGTACTGCACAGTCTGGAAGCGCGGAAACACAAAGAGGATCCTGAACGGCGGCAGCAGCGTCTCGCCGCGCTGGCGGAGCGCGATCCGCTGGCCTTTGAGCGTGACAAAGTAAAAGGCGCGATTCGCACCGATTTTATTTTGTCCGCGGAAATTGTCGCCATTACCCTCGGCATCGTGGCGGAAGCTCCGCTGCTTAACCAAATCCTTATTCTTTCGGGGATTGCGATTCTGGTCACTATCGGGGTGTATGGCCTGGTGGGGGTGATCGTCAAGCTGGACGACATGGGTTACTGGCTGGCGGAAAAACGTAGTGCGCTGGCGCAGTGGCTGGGTAAAGGGCTCCTGGCCGTCGCTCCCCGCCTGATGAAGGTGTTGTCCATCGTCGGGACGCTGGCCATGTTCCTCGTCGGCGGGGGGATAGTCGTTCACGGTATTGCGCCGCTGCATCATGCCATCGAGCACTGGAGCGCGGGGTTGGGTGGGGTGATGGCCTCCACGCTGCCGGTCGTCGCCAACCTGGTGCTGGGTTTTATCATCGGCGCGGTGGTGCTGGCGGGCGTTAAGGCGGTGAGCAGCCTGCGTGGCGCTGGAAAGTAG

>IR1230_00365

ATGTCTACCCGTCAGCTTTTACCGCTCATCGGGGCGCTGTTTGCGCTGTATATCATCTGGGGCTCTACCTATTTCGCTATCGCCGTCGGGGTCGCCAGCTGGCCGCCGCTAATGATGGCCGGCATTCGTTTTCTCGCTGCCGGCGTGCTGTTGCTGGGGTGGCTACTGGCCACCGGCCATAAGCTACCGGCGCGTCGTCCGCTGCTTAACGCTGCGCTGATCGGCGTGTTGCTGCTTGCGGTTGGCAACGGCTTCGTGACGCTGGCTGAACATCAGCACGTCCCCTCCGGCATCGCGGCGGTGATGGTCGCCACCGTACCGCTGTTCACCTTATGCTTCAGCCGCTTTTTCGGCATCGCCACCCGTAAACTGGAGTGGCTGGGCATTGCCATTGGCCTTGCGGGTATCGTGATGCTCAACAGCGGAGGCAACCTGAACGGCAACCCCTGGGGCGCACTGCTGATCCTGATTGGTTCCCTAAGCTGGGCCTTCGGCTCGGTTTACGGTTCGCGAATCGTACTGCCGACCGGCATGATGGCGGGGGCAATCGAGATGCTCGCCGCCGGGATCGTCCTGCTGGTGGCCTCATGGCTGAGCGGTGAAACCTTAACCCGCGTCCCTTCCTGGTCCGGGATCGCTGCCCTGGCCTATCTGGCCATCTTTGGCTCGCTGATCGCCATTAACGCCTACATGTTTTTAATCCGCAATGTCACCCCGGCGGTCGCCACCAGCTACGCCTATGTTAATCCGGTGGTCGCGGTGCTCCTCGGTACTGGGTTCGGCGGCGAGAGCCTGTCGCTCATTGAGTGGCTGGCGCTGGCGGTGATTATTTTCGCCGTAGTGCTGGTGACGCTGGGGAAATATCTCTTCCCCGTCCGCAGCGAGGCTACTCCTTGTAAGGCGTCGAAGTAG

>IR1230_00366

ATGATGGCGGATGTTCACGATAAAGCGACGCGCAGTAAAAATATGCGGGCGATCGCCACGCGCGATACGGCGATTGAAAAGCGGATAGCGGCGCTGCTGACCGGGGCGGGATTCACCTTTGTCGCCCAGGATCGCGCGCTGCCGGGACGTCCCGATTTCACTTTACCCGACTACCGCTGCGTTATCTTTACTCACGGCTGTTTCTGGCATCACCATGACTGCTATCTGTTTAAGGTGCCGGCCACCCGCACGGCATTCTGGCTGGACAAAATCGCCGGTAACGTGGCGCGCGACGCCCGCGACCGGCAAAAGCTGGCCGAAGAGGGCTGGCGGGTATTAATCATCTGGGAATGCGCCCTGCGCGGGCGCCTTAGGCTGAGTGATGCGGCGCTCACCGAGCGTCTGGAAGAGTGGATCTGCGGCGCCGGACACGACGCGCAGATCGATACGCAGGGGATCCGCGAACTGACGACTACTTCGACGCCTTACAAGGAGTAG

>IR1230_00367

TTGTCGTTGGCTGAACAGGCCGGGGAAGACGCTGAGGCGCTGCTGCGTCAGCTGATGACGATCTACGATGTAAAAACGCTGGTTGCCGAACTGGTGAGCGTCGGCGAACAGCACTGGAGCGCGGCGATCCTCAAGCGCGTGGCGGCGCTTGGCCGCGCGGCGGAGCGTTTGCGCCCGCAGGAAGTTGCTCATCTGGCGACGCTCCTGCCTTCGCCGCCGGCCCATCATCCGCACTATGGGTTCCGTTTTATCGATCTGTTTGCCGGTATTGGTGGGATCCGCAGTGGTTTTGAAGCCATCGGCGGACAGTGCGTTTTCACCAGCGAGTGGAACAAGCACGCGGTACGCACCTATAAGGCCAACTGGTACTGTGATCCGCAGCAGCACCGTTTTAATGAAGATATCCGCGATATTACCCTCAGCCAACGCTCTGACGTTAGCGACGAGGAGGCGGCGCGGCATATTCGTGAATCTATCCCTCAGCATGATGTGCTGCTGGCTGGCTTCCCCTGCCAGCCTTTCTCGCTTGCCGGCGTCTCTAAGAAAAATGCGATGGGCCGTGCGCATGGCTTTGCCTGCGAAACCCAGGGAACGCTGTTTTTTGACGTGGTGAGAATTATCGCGGCTCGTCAGCCAGCGATTTTTGTGCTGGAGAATGTCAAAAACCTCAAAAGCCACGATCAGGGGCGCACCTTCCGTATCATCATGCAGACGCTGGATGAGCTGGGATATAAAGTAGCTGACGCGGGGCATACCGGGCCGGATGACCCGAAGGTGATTGACGGGCGCCATTTCTTGCCTCAACACCGGGAGCGCATCGTGCTGGTGGGTTTCCGTCGCGATCTGCAGCTGCATGCTGGATTTACGCTGCGCGACATCGCGGCGCAGTACCCGGCGGTGCGGCCAACATTTGGCGAGCTACTGGAGCCGACGGTCGATGCGAAATTTATTCTTACGCCGGTGCTGTGGAAATATCTTTATCGTTATGCCCGCAAGCATCAGGCGCGGGGCAATGGCTTTGGTTATGGCCTGGTTGACCCCGCCAATCCCCACAGCGTGGCCCGGACGCTTTCTGCCCGCTACTATAAAGATGGTGCCGAAATTCTCGTCGATCGCGGCTGGGACCGGCCGCTCGGTGAGAAGCATTTTGACGATCCGCTGAATCAACAGCGGCGGCCTCGCCGGTTGACGCCGCGCGAATGCGCCCGGCTGATGGGCTTTGAATCCCCGCAGGGGGCGCGTTTTCGCATCCCGGTATCGGATACGCAGGCCTATCGTCAGTTTGGCAACTCGGTGGTGGTTCCGGTCTTCGCCGCGGTGGCGAAACTCCTGGCGCCGCGCATCGCTCAGGCGGTGGCGCGCCGCGAAGCGGATGATAATGATGGCGGATGTTCACGATAA

>IR1230_00368

ATGGACCTGGTTGACTGGCAGCAACGATTTGAGCGCTGGATACTGACGCACCATGCGCAGGATGACGCCGCACACGATCTGTCCCATTTTCGTCGCGTCTGGGCGACCGCCACTCAGCTGGCCGCGGGGGAGGAAGTCGACCGGCTGGTACTCCTCACGGCCTGCTATTTTCATGACATCGTCAGCTTGCCAAAAAATCATCCGGAGCGAAGTCGATCCTCGATGATGGCCGCCGAGAAAACCCTCGCCATTCTGCAGTCGGCCTTTGCTGATTTTCCTGCGGATCGTTACCCGGCGGTCAGCCATGCCATCGAGGCGCACAGCTTCAGCGCGGCGATCCCACCGCGCACGCTGGAGGCGAAAATAGTTCAGGATGCCGATCGACTCGAGTCGCTGGGAGCCATTGGTCTGGCGAGGGTCTTCGCTGTCGCCGGCGCCCTGAATACCATCTTGTTTGATGCCGAAGATCCTTTTGCTGATCGGCGGGCGCTGGATGACCGCAAATATGCCCTCGACCATTTCCAGTGCAAACTTCTGCGCCTGCCGGAAACCATGCAAACTGACAAGGGGAAAGCGATGGCGCAGCATAATGCCCGTTTTCTCGTCGAATTTATGGCTAAGCTCAGCGCGGAGCTGCAGGGGGAGCCGCTGGCGCTGGATGAAGCGGTTTTGCGCCGCTTTGCACCGCAGGCGTCAACCGACCGGTGA

>IR1230_00369

ATGGAAGAGCAGGCAAAACGCAGCCCGGGAGGGAAACTTGCGCTCTGGGCGTTGTATGCTTTTTGCGGATATTGTGTCTGGGTCATTGTGCGTTACTGGTGGGTAGTGGGAAAAATTCACAGCGTTCCCGGCGCCAGCGTGGAGGGTGATTTTGGAACAACGGCCGGGAAATGGCTTGGCGCCCTGTTGGGCATGCTGGTCCTCGGCGGCATTGGCTCTATTCTCGGCGCGGTCGTCTGGTATACGCGTCCATCCCGCGGCGAGCAGGATCATCAGCGCTAA

>IR1230_00370

ATGAAAAGAAAAGTACTGGCACTCATGGTTCCGGCATTATTAATGGCTAGCGCCGCTAATGCCGCCGAAATCTATAACAAAAACGGTAATAAACTGGACCTCTACGGGAAAGTTGACGGCCTGCATTACTTCTCTGACGACGCTTCAGAGGACGGCGACCAGACCTATGTTCGCTTTGGTCTGAAAGGCGAAACGCAGATTACTTCCGAGTTAACCGGCTACGGTCAGTGGGAATACAACATTCAGGCGAACACCAGCGAAAAAGAAGGCGCGAATTCCTGGACCCGTCTCGGCTTTGCCGGCCTGAAGTTTGCCGACTGCGGGTCGCTGGACTATGGCCGTAACTACGGCGTGGTCTACGACATTGAATCCTGGACCGATATGCTGCCGGAATTCGGCGGCGATACCTACACCCAGACCGACGTTTACATGACCGGTCGTACTAACGGCGTGGCCACCTACCGTAACAGCGATTTCTTCGGCCTCGTCGACGGCCTGCATTTCGCCCTGCAGTATCAGGGTAATAATGAAAACGCCGGTTCTGGCGAAGGCACTAATAACGGTGGGAAACGTAAGCTGGCGCGGGAAAATGGCGACGGTTTTGGTATCTCCAGCTACTACGATCTCGATATGGGCATCAGCTTCGGGGCAGCGTATTCGTCTTCTGACCGTACCCATAACCAGCTGGCGGCCGCGCGTAGCAGCCAGCGTTATGCCAACGGCGATAAAGCCGATGCCTGGACCGTCGGCGCGAAATATGACGCCAATAACATCTATCTGGCCGCCATGTATGCTGAGACCCGCAACATGACCTTCTATGGCAACGATAGTTTTGGCGGAATTGCCAACAAAACGCAGAACTTCGAAGTGGTCGCGCAGTATCAGTTCGACGACTTTAATTTACCGCTGCGTCCGTCGGTGGCCTATCTGCAGTCGAAGGGTAAAGATCTCTACGCCTATTCTCGCTACGGCGACAAGGATCTGGTCAAGTATGTCGACGTGGGCATGACCTACTACTTCAACAAAAATATGTCCACCTATGTGGATTATAAAATCAACCTGCTGGATGAAGACGACCGCTTCTACAAAAACAGCGGTATCGCGACCGATGATATCGTCGCCCTCGGCCTGGTCTATCAGTTCTGA

>IR1230_00371

GGAGAGATGCCGGAGCGGCTGAACGGACCGGTCTCGAAAACCGGAGTAGGGGCAACTCTACCGGGGGTTCAAATCCCCCTCTCTCCGCCA

>IR1230_00372

ATGTTTAAATGGCCCTGGAAAGCAGATGATGAGTCCGGCAACGCAGAGATGCCCTGGGAGCAAGCGCTTGCCATTCCGGTTTTAGCCCATCTTTCGTCCACTGAGCAGCACAAGCTCACGCAGATGGCTGCCCGTTTTTTACAGCAAAAGCGGCTGGTGGCATTGCAGGGGCTGGAGCTGACCCCGCTGCATCAGGCGCGCATTGCTATGCTCTTTTGTCTGCCGGTGCTTGAGCTGGGCATTGAGTGGCTGGATGGCTTCCATGAAGTGCTGATCTACCCTGCCCCTTTCATCGTCGATGATGAATGGGAGGATGATATTGGTCTGGTCCACAACCAGCGGGTGGTACAGTCGGGACAAAGCTGGCAACAGGGTCCTGTAGTGCTGAACTGGCTGGATATTCAAGACTCTTTTGATGCGTCAGGCTTTAACCTTGTGGTGCATGAAGTGGCGCATAAGCTTGATACGCGGAACGGCGACCGGGCCAGCGGCGTGCCGCTTATCCCGTTGCGTGAAGTTGCCGGCTGGGAGCATGATCTCCACGCCGCGATGAACAACATTCAGGATGAGATAGATCTGGTCGGCGAAAGCGCCGCCAGTATTGACGCCTATGCCGCCACCGATCCCGCAGAGTGCTTTGCTGTCCTCTCGGAATATTTTTTCAGTGCGCCTGAACTGTTCGCGCCTCGCTTCCCGGCCCTGTGGCAGCGTTTTTGCCACTTTTACCGCCAGGATCCGCTGGCGCGTAGACGTGAGAATGGCCTGCAGGACGAAGGCGATCGGCGCATTGTTCACTAA

>IR1230_00373

TCCTCTGTAGTTCAGTCGGTAGAACGGCGGACTGTTAATCCGTATGTCACTGGTTCGAGTCCAGTCAGAGGAGCCA

>IR1230_00374

ATGGAGGATGCTGAAAACAGCCTGTCCAGTGCGGCGATGACGCCCAGGGGACGGCTACGGGTGGATGTACCGAGTCCGCTGGCCCGCCTTATTCTGGTGCCGGCGCTACCGGCTTTCCACGCCCGCTACCCTGATATCCAGATCGACATGGGTGTGAGCGACCGGGTAGTGGATCTGATCGGCGACAACGTGGATTGCGTTCTGCGCGGAGGTCAAATCACCGACCAGTCCCTAATCGCACGCCATGTCGGCGATTTGCAAATCGGCGTCTACGTCGCCCCCAGCTATGTGGAACGCCTTGGCGCTCCCGCACACCCGCGCGAGCTGCAAAATACTGACCATTGCATAGTGGGATTCTTGTCCTCACGCACCAGTAAGATTGATCCTTTGGTACTGTGCAGTGAGAATGAACGTATTGAAATCACGGGCAACTACGTACTTGCCGTGGATGATGGCAATGCTTACCTCGAAGCTGGGTTAGTTGGCTTAGGCGTGATTGCGCTGCCCAACTATATGGCGGCAGCGCATCAGGCCGTTGGCGCTTTGATTCCATTATTTACACAATGGCGGATTAGCCCAATGCCTCTGTACCTGGCGTTTCCACCGAACCGCCATGTCAACGCCAAGCTGCGCGTTTTTATTGACTGGATCGTTGAAGTGATGCTGCAGCATGTCCCCATTGCCAACAATCAGTGA

>IR1230_00375

ATGACGATGCGGCGTGTGAAATTACTCTGTACGGCGCTGATGCTGCTTGCCAGCCACGGCGCGCTGGCCGTCAGCTATCCGCTGCCGCCGGAGGGGAGCCGTTTGGTGGGCAGCGCATTTACCATTGCCGTTCCGGACAATAACACCCAGCCGCTGGAAAGTTTTGCCGCCCAGTACGGCCAGGGGTTAAGCAATATGCTGGAAGCCAACCCCGGGGTCGATGTCTATTTGCCACGCTCCGGTTCGACGCTGACTATTCCGCAACAGCTGATCCTGCCCGATACCGTGCGCGAAGGGATCGTGATCAACGTTGCCGAAATGCGTCTCTATTATTACCCGCCGCTGGGCAATAGCGTCGAAGTCCTGCCAATCGGCATTGGTCAGGCCGGACGCGAAACCCCGCGTAACTGGGTGACGGCAGTAGAGCGTAAGCAGGAAGGCCCCACCTGGGTGCCAACCGCCAATACCCGCCGCGAGTATGCGAAAGAGGGCAAAACCCTGCCGGCGCTGGTGCCCCCCGGGCCCGATAATCCGATGGGGCTGTATGCGATCTACATCGGCAGGCTCTACGCCATCCACGGCACCAATGCCAACTTTGGTATCGGGCTGCGCGTCAGCCAGGGATGCATCCGCCTGCGCAATGACGATATTAAATTCCTGTTTGATAACGTGCCGGTTGGGACGCGGGTTCAGCTGATCGATCAGCCGGTGAAGTACAGCGTGGAGCCGGACGGCAGTCACTGGCTGGAGGTGCACGAGCCGCTGTCGCGCAACCGCGCGGAGTTTGAATCCGACAGAAAAGTCCCCTTGCCGATGACCTCGGCGCTGCGCGACTTTACCCAGGGGCCGGGAGTGAGCCCTGCCCAGGTCGAGCAGACCCTGCAGCGCCGCTCCGGCATGCCGGTGAATATCAGCGCCACAGCGGCGCAGGGAAGTTTGTAG

>IR1230_00376

GTGAACTTCCAGCAGCTGAAAATTATCCGTGAGGCGGCCCGTCAGGATTACAACCTGACGGAAGTCGCGAATATGCTCTATACCTCGCAGTCAGGCGTCAGTCGCCATATCCGGGAACTGGAAGAGGAGCTGGGGATTGAGATCTTTATCCGCCGCGGCAAACGCCTGTTAGGCATGACCGAGCCGGGTAAAGCGCTGTTATCGATTGCCGAGCGGATCCTCAATGAAGCCAGTAACGTTCGGCGGCTGGCGGATCTCTTCACTAACGATGCGTCAGGCGTCTTAACTATCGCCACCACCCATACTCAGGCGCGCTACAGCCTGCCGCCGGTCATTAAAGCTTTCCGCGAGCTGTTCTCAGATGTTCGCGTCGAGCTGGTGCAGGGTACCCCGCAGGAAATCGAAGCGCTGCTGCATAACGGCGGAGCGGATATCGGCATCGCCAGCGAGCGCCTGAGCAACGATCCGACGCTGGCGGCTTTCCCCTGGTTCCGCTGGCATCACAGCCTGCTGGTGCCGAAGGATCATCCTTTGACGCAGGTTTCTCCCCTGACGCTGGAGGCGATCGCCCGCTGGCCGTTAATCACCTACCGCCAGGGGATCACTGGCCGTTCGCGCATTGATGAGGCGTTCAACCGCAAGGGGTTGATGCCGGATATCGTGCTGAGCGCGCAGGATTCCGATGTGATCAAGACCTACGTGGAGCTGGGGCTGGGCGTCGGGCTGGTCGCCGAGCAGTCCGGTGATGCCCGGGAAGCGGACACCTTTACGCGTCTTGATACCCGCCATCTGTTCGATGCCAATACCGTGTGGTTGGGACTGAAGCGGGGCCAGCTGCAGCGGAATTACGTTTGGCGCTTTATCGAATTGTGCAATGCCGGACTGTCGCTGGATGAGATTAAGCGTCAGGCGATGGAGCCGGAAGAGGTCGCAATCGACTATCAGATTTAA

>IR1230_00377

ATGAACTTAAGACGACTGAAATATTTTGTGAAAATCGTCGATATCGGCAGTCTGACCCAGGCCGCTGAAGTCTTGCATATCGCCCAACCGGCCCTCAGCCAACAGGTCGCGACCCTGGAAGGTGAAATGGACCAGCAGTTGCTGATACGCACCAAGCGAGGGGTCACGCCAACGGAAGCCGGTAAAATTCTCTACACCCATGCGCGCACTATCCTGCGCCAGTGCGAGCAGGCGCAGCTGGCGGTCAATAACGTCGGCCAGACGCTGCGTGGTCAGGTGTCGATCGGCCTGGCGCCAGGCACCGCGGCCTCCGCCATTACCATGCCGTTGCTGCAAACGGTGCGCAATGAGTTGCCTGAGGTGATGGTCTATCTGCAGGAGAGCAGCGGCACAGCGCTGAATGACAAGCTGCTGGCGGGCCAGCTGGATATGGCGGTGCTCTATGAGCGTTCGCCGGTGGCAGGCATCGTCAGCCAGCCGCTGCTGAAAGAAGACCTTTACCTGGTCGGCACCCGCGACTGCCCGGGGCAGAGCGTGGATCTCACGGCGGTGGCGGAGATGAACCTGTTCCTGCCGCGCGATTACAGCGCAGTGCGGGCGCGGGTCACTGAGGCATTCACCCTGCGTCGTCTATCGGCGAAAATTATCGGCGAGATCGAATCCATTACCACCCTGACCGCGGCCATCGCCAGCGGCATGGGGGCGACGGTGTTACCGGAATCCGCTGCCCGTTCCCTGTGCGGGGCGGCGAACGGCTGGATGGCGCGGATCAGCACGCCTTCAATGAGCCTGTCGCTGTCGCTGAACATGTCGGCTCGCGGCAGTCTGTCGCCGCAGGCGCAGGCGGTGAAAGAGATCCTGCTGTCTCTGGTGAGCCGCCCATCGCTGGAAAACCGTGAACTGCAGCTGGTCAGTTAG

>IR1230_00378

TCCTCTGTAGTTCAGTCGGTAGAACGGCGGACTGTTAATCCGTATGTCACTGGTTCGAGTCCAGTCAGAGGAGCCA

>IR1230_00379

ATGTCGCTAATTGCTATCGTGCTGGTCTTTATCATGGCCATCGTGGTGACCGTGTTCCTGTCCCATCTTCTGCCCGTTAAAGTGCCGCTACCGCTGATCCAAATCGCCGCGGGCGCCGCGCTGGCCGCCAGTGGCTTTCAGGTCGATTTTGACCCGCATATTTTTTTACTGCTGTTTATCCCGCCGCTGCTGTTCCTCGATGGCTGGCGCATCCCCAAAGACGCTTTTTTCCGCGATATGAAGCCGATTTTATCGCTGGCGATAGGCCTGGTGATGGTGACTATTCTCGGTATTGGCCTGTTTATTCACTGGCTGATCCCGGCTATCACCGTGGCGGCAGGGTTCGCCCTCGCGGCTATTCTGTCGCCTACCGATCCGGTGGCGGTATCGGCAATGACCGCCAGCTCGCCGCTTCCCTCAAGGATGGCGCATATTCTCGAAGGGGAGTCGTTGTTAAACGATGCTTCAGGGCTCGTTGCCTTTAACTTCGCCATCGCCGCGGTGCTGACCGGCAGCTTCTCCCCGGGAGACGCGGTGGTGAAATTTTTCCTGATGGCCTTCGGGGGCATCCTCAGTGGCCTCGTGGTGGTGTGGGTCACCGGCAAATGTAATAACTTTCTGGTTCGGCGCACCCGCGAGGAGCCAGCCATCCAGATCCTGATCAGCCTGCTTATCCCTTTCGCCGCCTACCTGCTGGCCGAGGCGTTCCATGTCTCGGGCATTCTTGCCGCCGTCGCCGCCGGCATTGCGATGCATTATGAGCAGCTGTCGGGGCCCCGGCTTCCGGCGACGCGCATGAAAAGCAGCGCGGTATGGTCCATGCTGCAGACCACCCTCAACGGGATGATCTTTCTGATGCTGGGCGAGCAGCTGCCGCGCATGCTCAGGACGCTGCCGGCGGTAGCCAGCCAGGCCGGCGTCTCTTCGCCCTGGTATCTGCTGCTGTACGCCGTGGCCATCACCCTGGCGCTCGGTCTGATGCGCTTCGCCTGGGTATGGCTGTCGATGAAGCTGACAATTTTTCGCCGCAAACGTCGAGGCAAGGCCATCACCGTCCGTCCACGCTTCAGTATCCTCGCCGTCATGGCGCTGGCGGGAGTGAAGGGCTCTGTGACCCTCGCCGGTATTCTGACGCTGCCGGTGGTGCTGGCAGATGGTTCGCCGTTCCCGGGCCGTGAACTACTGATCTTTCTTTCGATGGCGGTGATCCTGATGTCGCTGGTGGTGGCGGCTATCGGTTTGCCGTTTATGACTCGTTATCTTGCCGATGATCTACCGCATGACACCGGTAAGGACGACATTGGCGCCGTGATGACCGAGGTGGCCATTAACCGTCTTAACGCGCTACTGGACGAGCCGGTGGAGGATCCCAGTGAGCAGGCCCTGCGGGCCGATGCCGGCAATATGCTGCTCGAAACCTATCAGCGGCGGCTTCACTATAACGACAATGACGAGGGGCAGGATGTCGGGCTGGAGCTGGCTAAGCGCGCCAGGCTGGAGAAATATATGCAACGAGAGGTCATTATCGCCCAGCGTCAGGAACTGTTCCGGCTGCGCCGCGCCCATAACATCAGCGATATCACTTTTTATGAGGTGCTGCGGGAGATAGATCTGAAGGAAGAGAGTTTACGCTAA

>IR1230_00380

ATGAATCCCAATACCTCCACCGTCCTGAAGCCCCGCCGCGGCCGTCCACCCAAGGTCGATCGCCAGTTTGACGATACGCGTCAGGCGCTGATTCGCTCAGGCCTTGAAGTGCTGACCGAAACGGGCTATCTCGCGGCGGGTATTGATGCGGTGATCAAGAATATCGCCGTGCCAAAGGGGTCGTTTTATCATTGCTTTAAAAGCAAAGAGGCTTTTGGCCTCGCCGTGCTTGCCGCCTATGGCGACTTCTTTGCGCATAAATTAGATAAATTCCTGCTGGACGACGCCGTGCCGCCGCTGGAACGAATGGCGGCCTTTGTGCGTCATGCCGGTCAGGGAATGGAGAAATTCCAGTTTCGCCGCGGCTGCCTGGTGGGCAACCTGCTGCAGGAAGCCCCGTTGTTACCCGAGACGTTTCCCCAACGGTTGATGGCTATCCTGGCGGCATGGGAGTCGCGCGTGGCGCGCTGCCTGCGCGAAGCGCAGGCAGCCGGGGCCATCGCCAGCGACGCCTCACCGCAGGCGCTGGCCCAGGTGTTCTGGATCGGCTGGGAAGGCGCGGTGATGCGCGCCAGGCTGGTCCAGTCTGCCGCACCGCTTAACCAGTACTGGGATTTTTTTGCGCACAGTATGACAACAAAAACACCTGCACAGGACGGCGCTAGCGCCGATAACCCCCTTCCGACGAGGAATACTCTGTCATGA

>IR1230_00381

ATGAAAGCATTAGTTCTGGACCAGATCGACAACCGCACCGTCGCCACCGTCAAAGATATCGACCTGCCCGCTCTTGCCGAAGGTGACGTCCGGGTCGCCATCGACTGGTCGAGTCTCAATTATAAAGACGCCCTGGCGATCACCGGCAAAGGCAAAATTATCCGTCAGTTTCCGATGGTGCCGGGCATTGATTTTGCCGGTCAGGTGAAAGAAAGCCGCGATCCGCGCTTTGTTCCCGGCCAGGCCGTGATCCTCACCGGCTGGGGCGTGGGCGAAAACCACTGGGGTGGTCTGGCCACCGAAGCCTGCGTTAAAGGCGACTGGCTGGTTGCTCTGCCGGAGACGCTCAGCGCGCGCCAGGCAATGATCATCGGCACCGCCGGCTTTACCGCGATGCTATGCGTTGATGCTCTCGTTAGTGCGGGTGTCACCCCTGATAGCGGGGATATCCTGGTCACCGGCGCCAGCGGCGGGGTCGGCAGCACCGCCGTGGTGTTGCTGAAAGCCCTTGGCTACCGGGTGACGGCCGTATCGGGCCGGGAATCGACACACGATTACCTTCGCCAGCTGGGCGCCGATACGATCCTGCCGCGCAGCGACTTCGCGGAGACCCGTCCGCTGGAGAAACAGCTGTGGGCGGGCGCGGTGGATACCGTTGGCGGCCCGGTGTTGGCCAAAGTGCTGGCGCAAACGCAATATCGCGGCTGCGTCGCCGCCTGCGGTCTGGCCGGGGGTTTCGATCTGCCGACCACCGTGATGCCGTTTATTCTGCGCAATGTGCGTCTGCAGGGGGTGGATTCGGTGATGGTGCCGACCGCTGAACGCGACGCTGTCTGGCAGCGTCTGGCCCAGCTTCTGCCGGAGAGCTACTACCAGCAGGCGGCCACTGAAATCACGCTGGAGCAGGCGCCAGCGTATGCCGCAGATTTCCTCAGCAATAATATTCATGGTCGGACGCTGGTCAATATCGGTCAGTAA

>IR1230_00382

ATGGATGTCATAAGCATCATTATGGCGGCGGGCAAGTCGTCCGTTGACGTCGCGCTGTATACGCTGTTGCCGATCATGGTGATCATGCTGATCATCATGAAATACCTTGAGGTCCGCGGTATCCTCGATGTGATTGTTCGCTGGGTGGCCCCGCTGCTGAAGCCCTTCGGCTTAACCGGGATGAGCGCCTTCGCCCTGATCCAGATTAATTTTGTCAGCTTTGCCGCCCCGCTCGCCACGCTGTCGATTATGGATAAACGCGGCGTCTCGGACCGACAAATGGCCGCCACGCTGGCCATGGTGTTCGCCATGGGGCAAGGCAACGTCTTCTATCCTCTTACCCCTTTTGGTCTGCACTGGCTGGCCTCGATCGTTATCTCGGTGGTCGGCGGCCTGTGCGCGGCCGCCGTCGCCTGGCACGTCACCGGACGGCGCTTATCGGTCGCCGAGAACCCCCGCGCGGAAGCGCTGCCGAACGCCGAGCAGAACAGCCAGGGAATTCTGGCCGTGATTAACAGCGCCGGCTCCGACGCCATCCGCCTGGCGCTCGGCGCGGTGCCAATGCTGATCCTGTCGTTAACCATCGTCGGGCTGCTGCAGGGCGCCGGGGCTATCGACCTGCTGCAGCAGCTGCTGAAGCCGCTTCTCAGCTGGCTGCACATCCCCCAGAACTTTGTGCTACCGGCGCTGGTGAAGTGCGTTGCCGGGGGCACCGCCTACTTTGGCGTCATTTCCGAGCTGATCCAGCAGGGCAAAGTCACCGTGGGCCAGGTCAACGCCTCGGCAGGTCTACTGATCCAGACCTTTGATCTTCCCGGTATCGGCATTTTTCTTGGCATCAGCTCACGGTTTGTCCGCCTGTTCCGCTTCGTGGCTCCCGCGGCGATCGTCGGTATCCTGCTGCGTACCGTTCTGCACCTGATCCTGTTTTAA

>IR1230_00383

ATGAAGCATCTATTTCGACACTGGCGAACGTCCGGCGCCGTTATCGGCTCACTGCTGAAAAAAGGGAGCATTGCGGTACTGGCGCTGCTGGTGGTCTTTCTCGCCGGGCGGATCTATGAGTCCCAACGCGGCCCGTCCCTGCATCGCTGGCATACCTGGAGTGGCAACGAGATGTCGGCCGAAGAGATCGACCAGGCGACCTTTGCCCAGTATCTGGCGCGGGAGAAGACGATTTTCGCCGATCTCCAGCGTGAGGTGACAGAGGCTTTGCCGGAGGAAGATAAAACCCCGGTCAACCGCTTTTATCGTCACAGCCGGGTCTGGCCGGGGCAGTTTAAGCAGGACTGGAACCGTTCTTTTGTGCTGATGCCGCTGGGCAAGCCGCGCGGCGGCGTGGTGCTCCTCCATGGCCTGACCGATTCGCCCTACAGCGTGCGCTATCTGGCGCAACTCTGGCAGCAGCGGGGTTATGTGGCGGTGGCGCCGCGTTTGCCGGGACACGGCACCGCGCCGGGGGCGTTAACGGCGGTTGACTGGGAGACCTGGCTGGCGGCGACGCGTCTGGCGGTACGCGAAGCGACCCGCCTTGCCGGGGCGGACGTGCCGCTGCATCTGGTCGGCTACTCTAACGGCGGCGCGCTGGCGCTCAAGTATGCCCTCGATAGCCTGGAAGACAATCACTTGCGCCAGCCGCAGCAGATCATTCTGCTGTCGCCGATGATCGGCGTGACCGCGTTTGCCCGTTTCGCCGGCCTCGCCGGGCTACCGTCCGTCTTCCCGGCGTTCGCCCGCGCGGCCTGGCTGAACGTGGCGCCTGAGTTTAATCCGTTCAAGTACAACTCCTTTCCGGTCAAGGCGGCGAGACAATCGTGGTTATTGAGCCAGGCGCTGCAGCAGCAGATTATCCGTGCGGCCCGGCAGGGCGAGCTGAAAGCGCTGCCGCCGATCCTCACCTTTCAGTCGGTGATGGATTCCACGGTCAGCACCCGCGCGGTGGTGGAGTCCCTGTATCGTTATCTGCCGGATAACGGCAGCGAGCTGGTGGTGTTCGATATTAACCAGGCGGCGGATCTCCGGGTCCTGTTTCGCCCTGCGCTCTATGCGGCGGTGAATACGCTGCTGCCCCCGGCGCCGCGGGCCTATACCACCACGGTGGTGACCAACGCGACGGCACACACGCTGCAGACCGTCGCCCGGACCACGCTGGCCCAGGATCGGGAAGAGCATCGTTATCCTTTACATCTGGCCTGGCCGGTGGATATGTACTCTTTGTCGCATGTGGCGGTGCCGTTTCCCCTGAGCGACTCGCTGTATGGTCGCGAGCCGGACGAGAAGAACCGCTACGGCATTAGCCTGGGCACCATTTCACTGCGCGGAGAGACCGGGACGCTGAGCGTGGGGCTGGAGACGTTAATGCGCGTCACCTCCAATCCATTCTTTCCATGGATGATGACGCGCGTCGATGAGCGCATCGCCTGCGGCGAACAGGCGGCGGTGGCGGCCTGCCTGAAAGCGCAGGCCAGAGCAGAGGCGTTAAAACAGGATCAGGTGCAGAACGGTACGCAGCAGGATACCGACGATCGCCGCGGGAGCCACGAAGCGGAACAGGCGGACAAACCGTGA

>IR1230_00384

ATGGAGTGGTGGGTAAAAAAAGTACAGGACAACGCTTCGGCCAGCCTCTGCCGCGTCGTGCTTCAGAGCGGTGCGCTAGAGATGATCGCAGAGATTGAAGCCTGTCGCCTCCGCCTGCGGGAAGGGGATAAACTCACTCCGCTGGCCGATGCGCGCTATTGTCTGAATAATAATCCGACGCAAACTTTGAAGATCCGCAACGCCACTCATTATAGCAGCGAGCGTTGGACAAACGCCGGCAAATAA

>IR1230_00385

ATGAATATCAAGGCCGCCAGTCTGACCCCGGAACAAGCGCTTGCCGAGCTGGAAGCACGCTATGAAGCCTCGGTCACGGCGTTACGCAAAGCCATCGGCGACTATATCGACCATAATACGCTCCCCGATACCGAAGCCCGCGCGGAAGGTCTTTTTGTCTATCCGCAGCTGTCGGTCTCCTGGGACGGCGCCGATCATAAAGCCCTCAAAACGCGCGCCTGGGGACGTTTCACCCATGCCGGTTGCTATACCACCACCATTACGAATCCGAAACTGTTCCGCAACTATTTGCTGGAACAGCTGACCCTGCTGTATCAGGACTATGGCGCGCATATCAGCGTCGAGCTGTCGCAGCATGAGATCCCCTATCCTTACGTGATAGATGGTTCCACCCTGACACTCGACCGCTCGATGAGCGCCGGTCTGACGCGCTATTTCCCTACCACCGAGCTGTCACAAATTGGCGATGAGACCGCGGATGGCCTGTTCCATCCCACCGAGTTCTATCCGCTGTCGCACTTTGACGCCCGCCGCGTCGATTTTTCGCTGGCGCGTCTGCGACACTACACCGGCACGCCGGCGGAACACTTCCAGCCCTACGTGCTGTTCACCAACTATACCCGCTACGTGGATGAGTTCGTCAGCTGGGGCTGCAGCCAGATCCTCGATCCCGACAGCCCCTATATCGCTCTCTCCTGCGCCGGCGGGATCTGGATCACCGCCGAGACCGAAGCGCCGGAACAGGCCATTTCCGATCTGGCGTGGAAGAAGCACCAGATGCCGGCCTGGCATCTGATCACCCACGACGGCAAAGGGATCACCCTGATTAACATTGGCGTCGGCCCGGCCAACGCCAAAACCATCTGCGATCATCTCGCGGTACTGCGGCCGGACGTCTGGTTAATGATCGGCCACTGCGGCGGCCTGCGCGAAAGCCAGGCCATCGGCGACTATGTGCTGGCCCACGCCTATCTGCGTGACGATCATGTACTGGATGCGGTGCTGCCGCCAGATATTCCGATCCCCAGCATTGCCGAAGTGCAGCGCGCCCTCTACGACGCCACCAAGCAGGTGAGCGGGATGCCCGGCGAAGAGGTTAAGCAGCGGCTGCGTACCGGGACGGTGGTCACCACCGACGACCGTAACTGGGAGTTGCGCTATTCCGCTTCGGCGCTGCGCTTTAATCTCAGCCGGGCGGTAGCTATCGATATGGAGAGCGCGACCATCGCCGCCCAGGGCTATCGCTTCCGCGTCCCCTACGGCACGCTGCTCTGCGTCTCGGACAAGCCGCTGCACGGTGAAATCAAGCTCCCCGGCCAGGCTAACCGCTTTTACGAGGGCGCTATCTCCGAGCATCTGCAGATAGGCATCCGGGCGATTGACCTGCTGCGCGCCGAGGGAGATCACATGCACTCGCGTAAGCTGCGCACCTTCAACGAGCCGCCGTTCCGTTAA

>IR1230_00386

TCCTCTGTAGTTCAGTCGGTAGAACGGCGGACTGTTAATCCGTATGTCACTGGTTCGAGTCCAGTCAGAGGAGCCA

>IR1230_00387

TTGAACGTCACCTCCGCCGTACGCCAGGCCGTCGAACGGACATCCTGGTTTAAAAAACGTCAGCGTTATCGTGTGCTGTACTGGCGCGAAATCAGCCCTCTCGCTGTGCCCATTCTGCTGGAAAATGCCTGTGTGCTGCTGATGGGGGTGTTGAGCACCTTCCTTGTCAGCTGGCTGGGAAAAGAGGCCATGGCCGGGGTGGGGCTGGCCGATAGCTTTAACATGGTCATCATGTCCTTCTTTGCCGCCATCGATTTGGGCACCACGGTGGTGGTGGCCTTTAGCCTCGGCAAGCGCGATCGGCGGCGGGCGAGGGCGGCCGCGCGCCAGTCGCTGGCCATTATGACCCTGTTTTCCATTTTGCTGGCGGGGGTGATCCATGCCTTTGGCCAGGAGATTATTGATTTTGTCGCCGGCGATGCCACCGCCCAGGTCAAGGACCTGGCGCTGACCTATCTGGAACTGACGGCGCTGAGCTACCCGGCAGCGGCGATTGCGCTGATCGGCAGCGGGGCGCTGCGCGGCGCCGGCACCACCAAAATCCCGCTGCTGATCAACGGCGGGATGAATATCCTCAATATCTTAATCAGCAGCGTGCTCATCTACGGCATCTTCTCCTGGCCGGGGCTGGGGTTTGTCGGCGCCGGGCTTGGGCTGACCATTGCCCGCTATATTGGCGCGATAGCCACGATCTGGGTGCTGATGGTGGGCCTCAATCCGGCGCTGCGGCTGTCGCTGAAGGGTTACTTCAAACCCTTTAACTTCGCCATTATCTGGGAGGTGATGGGGATCGGCATCCCGGCCAGTATCGAATCTGTCCTCTTCAATGGCGGCAAGCTGCTGACCCAGATGTTTGTCGCCGGGATGGGCACCAACGTTATCGCCGGCAACTTTATCGCCTTTTCGGTGGCCTCGCTAATTAACCTGCCGGGCAACGCCCTCGGCTCCGCCTCGACGATCATTACCGGCAAGCGGCTGGGAAAAGGGCAGATTGGTCAGGCTGAATTCCAGGCCTGGCACGTATTTTGGCTGTCGACGATTATTCTGACGCTGATCGCCTGGGGCAGCGCCCCCTTCGCCGGCTTTATCGCCTCGTTCTACACCCATGAAGAAGATGTAAAAGAAGTGGTTAAACAGCTGCTGTGGCTCAACGCCCTGTTTATGCCGATCTGGTCGCTGTCGTGGACTTTACCCTGCGCTTTTAAAGGCGCCCGTGACGTACGCTACACCATGTGGGTGTCGATGCTCGGCATGTGGGGCTGCCGGGTGGTGGCGGGCTATACCCTGGGCATCGTGCTGGGGATGGGGGTGATTGGCGTCTGGTTAGGGATGGTGCTCGACTGGGCGGTGCGCGGCGTGCTGTTTTATTTCCGCATGGTGAGCGGCCGCTGGCTGTGGAAATATCCGCGCGTGAAGGCTTCATCGGAGGAAAGCTGA

>IR1230_00388

TCCTCTGTAGTTCAGTCGGTAGAACGGCGGACTGTTAATCCGTATGTCACTGGTTCGAGTCCAGTCAGAGGAGCCA

>IR1230_00389

ATGTCCCTTACCGACGCAAAAATCCGCACCCTCAAGCCTTCTGATAAACCCTTTAAAGTCTCCGATTCTCACGGTCTGTATCTGCTGGTTAAGCCGGGTGGCTCCCGCCACTGGTATCTCAAATACCGTATTAGCGGTAAAGAATCCCGCATTGCGCTGGGTGCCTATCCAGCCATCTCCCTGTCTGATGCGCGACAGCAACGTGAAGGTATCCGTAAAATGCTGGCGCTGAATATCAACCCGGTACAGCAGCGGGCTGCTGAACGTGGCTCACGAACACCGGAGAAAGTTTTTAAAAACGTGGCGCTGGCGTGGCATAAAAGTAACAGGAAATGGTCGCAGAACACCGCCGACCGTCTGCTTGCCAGCCTGAACAATCACATCTTTCCGGTCATCGGGAACCTACCTGTATCAGAACTTAAACCCCGTCATTTCATTGACCTGCTGAAAGGGATCGAGGAAAAAGGTCTGCTGGAGGTTGCGTCCCGCACACGGCAGCACCTGAGTAACATAATGCGCCATGCGGTCCATCAGGAGTTAATCGATACGAACCCTGCAGCAAACCTTGGCGGCGTGACCACACCTCCTGTCAGACGGCACTATCCTGCCCTGCCGCTGGAGCGGCTGCCTGAACTGCTTGAACGTATTGGGGCATATCATCAGGGCCGTGAACTGACCCGGCATGCCGTTCTGCTGATGCTGCATGTGTTCATTCGCTCCAGTGAACTGCGTTTCGCCCGCTGGTCAGAGATTGATTTCACAAACCGAGTCTGGACGATACCCGCGACGCGAGAACCCATTATTGGCGTGCGTTATTCCGGCCGCGGGGCAAAAATGCGAATGCCGCATATCGTCCCCCTCTCAGAACAGTCCATCGCCATTCTGAAACAGATTAAGGATATCACCGGTAATAATGAACTGATCTTCCCCGGCGACCATAACCCGTATAAGCCAATGTCTGAAAACACGGTCAATAAGGCACTGCGGGTGATGGGTTACGACACGAAAAAGGATATCTGCGGTCACGGCTTCCGGGCAATGGCATGCAGTGCGCTGATGGAATCGGGTTTATGGGCAAAGGACGCAGTAGAACGCCAGATGAGTCATCAGGAGCGCAATACCGTGCGCATGGCTTATATTCATAAGGCAGAGCACCTAGAAGCCCGCAAAGCGATGATGCAGTGGTGGTCGGATTATCTGGAAGCATGCCGAGAATCTTATGCACCGCCTTATACAATTGGTAAAAATAAGTTTATCCCATAA

>IR1230_00390

ATGAAAATCAGTGAATTTCTACATCTGGCGTTACCAGAGGAACAATGGCTACCGACGATTTCTGGCGTTTTACGCCAGTTCGCAGAAGAAGAGTGTTATGTCTATGAGCGTCAACCCTGTTGGTATTTAGGCAAAGGGTGCCAGGCACGGCTGCACATTAATGCCGACGGAACGCAGGCGACATTTATTGATGATGCCGGGGAGCAAAAATGGGCGGTGGATTCCATTGCCGACTGCGCGCGTCGTTTTATGGCGCATCCTCAGGTGAAAGGACGTCGGGTATATGGACAGGTTGGGTTCAACTTTGCGGCGCATGCGCGGGGGATTGCCTTTAACGCCGGGGAGTGGCCGCTGCTGACGTTAACCGTTCCCCGTGAAGAACTTATTTTTGAAAAGGGAAATGTCACCGTTTATGCGGACTCCGCCGACGGGTGCCGACGTTTGTGCGAGTGGGTAAAAGAGGCCGGTACAACGACGCAGAACGCACCACTGGCGGTGGATACCGCCCTCAATGGTGAGGCATATAAACAACAGGTTGCGCGCGCCGTTGCGGAGATCCGCCGTGGCGAGTATGTCAAAGTGATTGTCTCGCGCGCCATTCCCCTGCCATCGCGGATTGATATGCCCGCCACGCTGTTATACGGGCGGCAGGCAAACACACCTGTGCGCTCGTTTATGTTCCGTCAGGAAGGACGCGAAGCGCTGGGCTTTAGCCCGGAACTGGTGATGTCAGTGACGGGCAATAAAGTGGTCACTGAACCGCTTGCGGGCACCCGCGATCGCATGGGAAACCCGGAGCATAATAAGGCGAAAGAGGCAGAACTGCTGCACGACAGTAAAGAGGTGCTTGAGCATATCCTTTCTGTCAAAGAAGCTATTGCTGAACTGGAGGCCGTTTGCCAGCCGGGCAGCGTAGTGGTTGAAGATTTAATGTCGGTTCGCCAGCGCGGCAGCGTTCAGCATCTGGGGTCTGGCGTGAGCGGTCAGCTTGCGGAAAACAAGGATGCCTGGGATGCGTTTACCGTGCTGTTTCCGTCGATTACCGCCTCAGGTATCCCTAAAAATGCTGCTCTGAACGCGATTATGCAAATTGAGAAGACGCCGCGAGAGCTTTATTCCGGCGCAATCCTGCTGCTGGACGATACGCGCTTTGATGCAGCGCTAGTTCTGCGTTCCGTATTTCAGGATAGCCAGCGCTGCTGGATACAGGCGGGGGCGGGAATCATCGCGCAATCTACGCCGGAACGCGAACTGACAGAAACCCGGGAGAAATTAGCGAGCATTGCGCCCTATTTAATGGTGTAG

>IR1230_00391

ATGAGTGATGTTCAGTCGAATGTGAAACCGCTGACGTTGACGACCGGGCGGGTGATTTTTGCTATTGCCGGCGTCTATGTGACGCAGAGTCTGGTATCGGCGCTGTCTATGCAGTCCTTACCCGCGCTGGTGCGCGCTGCTGGCGGTTCGCTGGCGCTTGCCGGTGCGACAACCCTGTTTATGCTGCCCTGGGCGCTGAAGTTTATTTGGGCGCCATGGATCGAGCGCTGGCGGCTTCCGCCCGGTAGCCAGGAACGCCGTTCCCGCATGTTAATCCTGCGTGGTCAGGTCGCGCTAGCGGCGATCCTGACTATTGCCGCAGCGATTGGCTGGTTTGGGCGAGAAGGGGGATTCCCCGATACGCAAATCGTCGCGTTATTTGTTCTGTTTATGGTGGCAGGCACGGTCGCCTCCACCATTGATATCGCCAGCGACGGCTTTTGCGTCGATCAACTGACCCGCACGGGTTACGGCTGGGGAAACAGCGTGCAGGTCGGCGGCAGCTATCTGGGAATGATGTGCGGCGGCGGGGTGTTCCTGATGCTGTCGGCGGCATCCGGCTGGCCTGTCGCCATGCTGATGATGGCGGTGCTGATTATGGCGCTGTCACTCCCGCTGTGGCGCATTACGGAGCCGACGCGAACAGCGACTATCCCGCATGTTCCGGCGTTAGGTTATGCGCTAAGGAGGAAGCAGGCGCGCCTGGGCTTACTGCTGGTATTGATGCTGAATTCAGGCATGCGGTTTGTGCTGCCTCTTCTGGCACCGCTGTTGTTGGATCATGGGTTGAGCATGTCCGCATTGGGCGCGCTGTTCAGCGGCGGCAATATTGCAGCGGGCATAGCAGGAACGCTGGCCGGCGGATTGCTGATGAAATACACCTCACCCGGCAGAGCGCTGTTGACGGCTTATGGCGTCCAGGGGATCGCGCTGCTGGCGGTGGTGATGACGCTCATGATGGCGCCGGGTCATCTGCTGCTGCCGATTCTCCAGTGTCTGGTCATTGTCCAGTCCATTTCGCTGGCCTGCGCGCTGGTCTGTCTTTACGCCACGCTGATGTCGCTTTCATCGCCTTTGCAGGCCGGTGTCGACTTCACCCTCTTTCAATGTACTGACGCGGCAATCGCCATTCTGGCAGGCGTTATCGGTGGCGTTGTTGCTCAACATTTTGGCTATGCGGCCTGCTTCCTGTTTGCCGGGGCATTCACGTTGCTGGCGGCGTGGGTTGCTTATATCCGGCTGCATTCGGCAAGAGAACTGATGACAAGCGCAATTGATTGA

>IR1230_00392

ATGAAAGACAATAATCCTGCGGATAACCTGGCCTGGCGCGTCATCTGGCGCCAGCTTATCTCCAGCGTTGGCAGTCAGGCCAGGATGCTGCGACGCAGTATGCTGGCGCTGTTGCTGGCGGCATTCATGCAGGGGATCGCCTTTGCCTGTCTTTATCCGATCATTGATGCGCTGTTACGGGGAGACGCGCCGCAACTTCTTAACTGGGCTATGGCCTTCAGCGTCGCCGCAATTGTGACGCTGGTGCTACGCTGGTATGGCCTGGGCTTTGAATACCGTGGTCATCTGGCGCAGGCCACCCATGAGTTGCGCCTGCGACTTGGCGAGCAGTTACGCCGCGTGCCGCTGGAGAAGCTCCAGCGCGGCAGGGCGGGTGAAATGAACGCCTTGCTGCTGGGCAACGTGGATGAAAACCTCAATTATGTTATTGCGATAGCCAATATTTTGCTGCTCACCATTGTCACGCCGCTGACGGCGTCGCTGGCGACATTGTGGATAGACTGGCGGCTGGGGCTGGTGATGTTGCTGATCTTCCCTCTGCTGGTGCCGTTTTATTACTGGCGCCGCCCGGCGATGCGGCGACAAATGCAGACGCTGGGGGAAGCGCACCAGCGTCTGAGCGGCGATATCGTTGAATTTGCTCAGGGGATGATGGTATTGCGCACCTGCGGCAGCGATGCCGATAAAAGCCGGGCGCTGCTGGCGCATTTCAATGCGCTGGAAAACTTACAGACCCGCACTCACCGTCAGGGCGCTGGCGCGACGATGCTGATCGCCAGCGTCGTGGAGTTGGGCCTACAGGTGGTGGTGTTATCCGGGATCGTCTGGGTAGTGACGGGCACTCTGAACCTCGCCTTTTTGATTGCCGCCGTCGCGATGATTATGCGCTTCGCAGAACCGATGGCGATGTTTATCAGCTACACCTCGGTTGTGGAACTGATCGCCAGCGCCCTGCAACGGATTGAGCGGTTTATGGCGATAGCACCGCTTCCCGTCGCAGAGCAAAGCGAGATGCCGGAACGTTACGATATCCGCTTTGACAACGTCAGCTATCGCTACGAAGAAGGCGACGGCCACGCGCTTAATCATGTTTCTTTGACGTTCCCGGCAGCCAGTATGAGCGCGCTGGTGGGTGCCTCCGGCGCAGGCAAAACTACGGTCACCAAACTGTTAATGCGCTATGCCGATCCGCAGCAAGGGCAGATTTCTATTGGCGGCGTCGATATTCGCCGCCTGACGCCGGAACAGCTCAATAGCCTGATTTCTGTTGTTTTCCAGGATGTCTGGCTGTTTGATGACACGCTGCTGGCGAATATCCGTATCGCGCGCCCACAGGCGACGCGGCAGGAGGTAGAAGAGGCCGCCCGCGCGGCGCAGTGCCTTGAGTTTATTTCCCGTCTTCCGCAAGGCTGGCTGACGCCAATGGGAGAGATGGGCGGCCAGCTATCGGGCGGCGAGCGCCAGCGGATTTCCATTGCCAGAGCGTTATTGAAAAACGCGCCGGTCGTCATTCTCGATGAACCGACTGCCGCGCTGGATATTGAAAGCGAGCTGGCGGTGCAAAAAGCGATCGATAACCTGGTTCACAACCGGACGGTGATTATCATCGCTCACCGTTTATCCACCATCGCCGGGGCCGGAAACATTCTGGTGATGGAAGAGGGACAGGTGGTTGAGCAGGGTACTCATGCGCAATTGCTCTCACATCATGGACGTTATCAGGCGCTGTGGCAGGCGCAAATGGCCGCGCGCGTGTGGCGCGACGACGGGGTTTCCGCGTCTGGAGAGTGGGTGCATGAGTGA

>IR1230_00393

ATGGCGCTGGCGGGGCTGGCGGCATTAACCAGCCTGGGGGCGCTCCTTTTTCTGGCGTGGAGCCTGCGTGACATTCGCGCGACGCCTGACGCTATTCCGGCCTGGCCGCTGGGCGGCGTGATCGGCTGCGTGGTCTTAACTTTTGTTCTGCGCTTACAGGCGTTCAACACCTCTCATTACGCGGCTTTTCATCTGGAGAACATTCTGCGCAGCAGGTTAGCCCGTAAAGCATTGCAGCTTCCGCCTGGCGTGTTACAGCAAATGGGCAGCGGGTCAGTGGCGAAAGTGATGCTGGATGACGTGAAGTCGTTACATATTTTTGTGGCCGACAGCACGCCGCTCTATGCCCGCGCGATCATCATGCCGCTGGCGACAATCGTTATCTTGTTCTGGCTGGACTGGCGGCTGGCAATCGCGACGCTGGGGGTACTGGCGTTTGGATCGGTTGTTCTCGTGCTCGCCCGCCAGCGTTCGGAAGATATGGCTCAGCGTTATCATAAGGCGCGCGAGCAGGTTAGCGCGGCGGTTATTGAGTTCGTGCAGGCCATGCCCGTGGTGAGAACGTTTGATAGCGGCAGCACCAGTTTTCTGCGCTATCAACGCGCCCTTGAAGAGTGGGTCGATGTGCTCAAAACCTGGTATCGCAAAGCCGGTTTTTCAGCGCGTTTTTCCTTCTCGATTCTGAATCCTCTCCCGACCCTGTTTGTCCTGATCTGGTCGGGATACGGCCTGTTGCACTATGGCAGTTTCGATTTTATCGCGTGGGTGGCCGTTTTACTGCTGGGCAGCGGAATGGCCGAAGCCGTAATGCCAATGATGATGCTCAATAACCTGGTCGCGCAAACGCGTTTAAGCATTCAGCGTATTTATCAGGTTCTCGCGATGCCGGAGTTATCGCTGCCGCAGTCTGACCAGCAGCCGCAAGAGGCGAGCATTACCTTTGAGCAGGTGAGCTTTCATTATCCGCAAGCGCGTACTGGCGCCGCGTTGCAGGAGGTGAGCTTTCATGTGCCTGCCGGGCAAATTGTGGCGCTGGTCGGGCCAAGCGGCGCCGGAAAAAGCACCGTGGCGCGCTTGCTGCTGCGTTACGCCGACCCGGACAAAGGCCATATCCGTATTGGCGGCGTGGATCTGCGTGATATGCAGACGGACACCCTGATGAAGCAACTCTCGTTTGTGTTTCAGGACAACTTCCTTTTTGCCGACACGATAGCCAATAACATTCGTCTGGGCGCGCCGGATACGCCGCTGGAGGCGGTAATAGCGGCGGCCAGAGTGGCGCAGGCCCATGATTTTATTAGCGCTCTGCCAGAAGGTTACAACACACGAGTCGGGGAACGTGGGGTATTTCTCTCCGGCGGCCAGCGGCAGCGCATTACTATCGCCCGGGCGCTTTTGCAGGATCGCCCCATCCTGGTGCTCGATGAGGCGACGGCGTTTGCTGACCCGGAAAACGAAGCGGCGCTTATCAAGGCGCTCGCGGCGGCCATGCGTGGCCGGACGGTCATCATGGTCGCGCATCGTCTCTCAATGGTGACTCAGGCCGATGTGATATTGCTGTTTTCCGACGGACAGCTCAGGGAAATGGGGAACCATACGCAACTGTTGGCGCAGGGCGGCCTGTATCAACGGCTCTGGCAACACTATCAGCAGGCGCAGCATTGGGTGCCGGGTGGAACACAGGAAGAGGTGGTGGAAAATGAAAGACAATAA

>IR1230_00394

ATGACGGAGTCACCGCAAACGCAATCTGAAATCTCTATTCACCAGTTGGTGGTCGGGAAACCTGCAAACGATGGCAATATTCCTGCTCAGTGTGAACTGCTGCGCTGTTCGTTGCAGGAAGGTATGGATATTTTGCTCTGGAGGGGCCATTTTGCGCGGCCTGAGACGTTGCAGCTACACGACGATCTGGGCAGGATTAACTTCTCGTGCATCCTGGAAGGCACATCGCGCTTTGCGATTCAGGGACTCCGGCGCCACACCGACTGGGAGCTTGCGCGCAATCGCCATTACATTACCCATACGCCAGACTGTCGCGGCAGCGCCTCCTATTGTGGCCGATTCGAGAGCATTACCCTCTCCTTCAGCCCTGAAACCCTCGCGCTGTGGGTGCCGGATATTAGCGCGGTCATTAAGAATAAGATCGACTCCCACTGCTGTTGCCAGCAGCAGCGTTGTAATGCTGAAACCCACTTAACGGCTCAGGCCTTGCGTCACGCGCTGATGCGCATGCACGGCGGCTTCAGTCATGAACAAAAGCCGTCGACGCTATGGTTGCAGGGGCAAAGTCTGGTCATGCTCAGTCTGGTGCTGGATGAACATCGCGAAGACGCCAGTTGCCTCTCCTGCCACTTCAACCCGATGGAACGCCAGAAACTGCTGCGGGCGAAGGATCTGCTGCTGGCTGATCTGACGCAAGCGCCGGGCGTCGCGGAGCTGGCCAGAGAGTCCGGTCTGAGCGTTCTCAAAATTAAGCGAGGCTTCCGCGTTCTGTTCAACAACAGCGTCTATGGCCTGTTTCAGGCTGAACGAATGCAGGAAGCCAGAAGACGCCTGGCCAACGGCAATACCTCCGTCATGACGGTGGCGGCCGATCTGGGCTATGCCAACGCCAGCCATTTCTCTGCCGCCTTCCAGAAACAGTTTGGCGTAACCCCTTCGACCTTTAAACGCGTGATGTAA

>IR1230_00395

ATGATTTCTGGCGCACCATCTCAGGATTCGCTGTTACCGGACAACCGCCACGCGGCTGATTACCAACAATTACGCGAGCGGCTCATACAGGAACTGAATTTAACGCCGCAGCAGTTACATGAAGAGAGCAACCTGATCCAGGCCGGCCTGGATTCCATAAGATTGATGAGATGGTTACACTGGTTTCGTAAAAATGGCTACCGCCTTACCCTTCGCGAGCTGTATGCCGCCCCCACGCTGGCGGCATGGAACCAGTTAATGCTCAGCCGGTCGCCTGAGAACGCGGAAGAAGAAACGCCGCCCGACGAATCATCCTGGCCGAACATGACCGAAAGTACCCCCTTCCCATTGACGCCAGTACAGCACGCCTACCTGACGGGCCGCATGCCGGGGCAGACGCTTGGCGGCGTGGGTTGCCACCTGTATCAGGAGTTTGAAGGCCATTGTCTGACGGCGTCGCAGTTGGAGCAGGCCATCACGACCTTGCTGCAACGCCACCCAATGCTGCATATCGCCTTTCGCCCCGACGGGCAGCAGGTCTGGCTACCGCAACCTTACTGGAACGGCGTCACCGTTCATGATTTACGCCATAACGACGCTGAAAGCCGCCAGGCCTATCTGGACGCACTGCGCCAGCGCCTGAGCCACCGTCTTTTACGCGTGGAAATCGGCGAAACGTTTGATTTTCAGCTGACGCTCTTGCCGGACAATCGCCACCGCCTCCATGTCAATATTGACCTGCTGATTATGGATGCCTCCAGCTTTACGCTTTTCTTCGATGAGCTTAACGCCCTGCTGGCCGGAGAATCGCTGCCGGCTATCGACACCCGCTATGATTTCCGCTCGTATTTGCTGCACCAGCAGAAGATCAATCAAACACTGAGAGACGACGCGCGCGCTTACTGGCTGGCGAAAGCATCGACGCTTCCCCCCGCGCCCGTCTTGCCGCTGGCCTGCGAACCCGCCACGCTACGTGAAGTCCGTAATACCCGACGCCGCATGATTGTCCCGGCAACACGCTGGCACGCCTTTAGCAACCGGGCCGGCGAGTATGGCGTGACGCCGACAATGGCGCTGGCGACCTGTTTTTCTGCCGTGCTGGCTCGCTGGGGCGGCCTGACGCGTCTGCTGCTTAACATCACCTTATTCGACCGCCAGCCGCTGCACCCGGCGGTTGGCGCGATGCTTGCCGACTTCACCAATATTCTTCTGCTGGATACCGCCTGCGATGGCGATACCGTCAGCAACCTGGCGCGTAAAAACCAGCTCACGTTTACGGAGGACTGGGAGCATCGCCACTGGTCCGGCGTCGAATTACTCCGTGAACTCAAACGCCAGCAGCGCTACCCCCACGGCGCCCCGGTGGTATTTACCAGCAATCTGGGGCGTTCCCTCTACAGCAGCCGCGCAGAATCGCCGTTGGGCGAGCCGGAATGGGGCATCTCGCAAACGCCGCAGGTCTGGATAGATCATCTGGCGTTCGAGCATCACGGCGAGGTCTGGCTACAATGGGACAGCAACGACGCGCTGTTCCCTCCGGCGTTAGTCGAAACATTGTTCGACGCCTACTGCCAGTTGATTAACCAACTCTGCGATGACGAAAGCGCCTGGCAAAAGCCGTTCGCAGATATGATGCCCGCCAGCCAGCGCGCGATACGCGAACGGGTCAACGCCACCGGCGCCCCCATTCCCGAAGGCTTGCTGCATGAAGGCATTTTCCGTATCGCTCTGCAACAGCCGCAGGCGCTGGCGGTAACGGACATGCGTTATCAGTGGAATTATCATGAGCTGACAGACTATGCCCGCCGTTGCGCGGGCAGGTTAATCGAGTGCGGGGTTCAGCCCGGCGATAATGTGGCTATCACGATGTCGAAAGGCGCAGGACAACTTGTTGCGGTTCTGGCCGTCCTGCTGGCCGGGGCGGTTTACGTTCCGGTTTCGCTGGATCAGCCTGCCGCACGGCGCGAGAAAATCTACGCTGACGCCAGCGTCCGGCTGGTGCTCATTTGTCAGCACGACACCAGCGCCGGGTCAGACGATATTCCCGTCCTTGCCTGGCAGCAGGCCATTGAGGCGGAGCCGATCGCCAACCCGGTAGTACGCGCCCCCACGCAACCGGCCTACATTATCTACACCTCCGGCTCTACCGGTACGCCGAAAGGGGTAGTCATTTCTCACCGGGGAGCGCTTAACACCTGTTGCGATATCAATACCCGCTATCAGGTTGGCCCGCATGACAGGGTGCTGGCCCTCTCCGCCCTACATTTTGATTTATCGGTTTACGACATTTTTGGCGTACTGCGCGCGGGCGGCGCGCTGGTGATGGTGATGGAAAATCAACGGCGCGATCCTCACGCATGGTGTGAGCTGATCCAGCGCCATCAGGTCACGCTCTGGAACAGCGTCCCGGCGCTGTTCGATATGCTGCTGACCTGGTGTGAAGGTTTCGCCGACGCCACGCCGGAAAACCTGCGCGCAGTGATGCTTTCCGGCGACTGGATCGGGCTTGAACTCCCCGCCCGTTATCGGGCCTTCCGGCCACAAGGACAATTTATCGCGATGGGCGGCGCCACCGAGGCGTCTATCTGGTCTAACGCCTGCGAAATTCACGACGTCCCCGCCCACTGGCGCTCCATCCCTTACGGTTTTCCGCTAACCAACCAACGCTACCGGGTGGTGGATGAACAGGGCCGGGACTGCCCTGACTGGGTGCCGGGTGAATTATGGATTGGCGGCATTGGGGTCGCGGAAGGCTATTTCAACGATCCCCTGCGTAGCGAGCAGCAATTTTTGACGCTCCCGGACGAGCGCTGGTATCGCACCGGCGATCTCGGCTGCTACTGGCCAGATGGCACAATCGAGTTCCTCGGTCGTCGCGACAAGCAGGTCAAAGTCGGAGGATATCGCATCGAGCTGGGCGAAATCGAAAGCGCGCTCAGCCAGCTGGCGGGGGTGAAACAAGCAACCGTTCTGGCGATCGGCGAAAAAGAAAAAACGCTGGCGGCATACGTTGTTCCTCAGGGCGAGGCTTTTTGCGTTACCGATCATCGGAACCCGGCACTGCCGCAGGCGTGGCACACGCTTGCGGGAACGTTGCCCTGTTGCGCCATCTCGCCAGAGATCTCCGCAGAACAGGTAGCCGATTTCCTTCAGCATCGCCTGCTAAAACTGAAGCCGGGTCACACCGCTGGCGCCGATCCTCTCCCCCTGATGAACTCACTCGCTATCCAGCCGCGCTGGCAGGCCGTGGTGGAACTCTGGTTAGCATTTCTGGTGACACAACGGCGACTGAAGCCCGCTGCTGAAGGTTATCAGGTCTGCGCTGGTGAAGAACACGAGGATGAGCACCCGCACTTCAGCGGACATGATTTAACGTTATCGCAAATTCTTCGCGGTGCCCGTAACGAACTGTCGTTACTGAACGACGCGCAGTGGTCGCCGGAAAGCCTGGCCTTTAACCATCCGGCCAGCGCCCCGTATATTCAGGAACTGGCGACAATTTGCCAACAGCTTGCACAGCGCTTACAGCGCCCGGTACGCCTGCTTGATGTGGGAACCCGCACTGGCCGCGCCGCAGAATCGCTGTTAGCACAGCTCAACGCCGGACAGATTGAGTATGTCGGGCTTGAGCAGAGCCAGGAGATGCTGCTGAGCGCCCGGCAGAGGCTCGCCCCCTGGCCTGGCGCCCGTCTGTCCCTCTGGAATGCAGACACGCTGGCGGCGCACGCTCACTCGGCGGACATTATCTGGCTTAATAACGCCCTGCATCGTCTGCTGCCGGAAGATCCCGGGCTCCTTGCGACATTACAACAGCTTGCCGTTCCCGGCGCGCTGCTCTACGTGATGGAGTTTCGCCAGTTAACGCCGTCCGCCCTGCTCAGCACGCTCCTGTTAACCAATGGGCAGCCGGAGGCCTTGCTGCATAACAGCGCCGACTGGGCGGCATTATTTAGCGCGGCCGCCTTCAACTGTCAGCATGGCGATGAGGTCGCGGGGTTACAACGCTTCCTCGTACAATGTCCTGACAGGCAGGTGCGCCGCGATCCCCGTCAACTTCAGGCCGCCCTCGCCGGACGTCTGCCGGGGTGGATGGTGCCGCAACGGATCGTCTTCCTCGACGCCTTACCGCTGACGGCTAACGGGAAAATTGACTACCAGGCGCTGAAGCGTCGTCATACCCCTGAAGCGGAAAACCCGGCCGAAGCGGATTTACCCCAGGGCGACATTGAAAAACAGGTTGCCGCCCTCTGGCAGCAACTCTTATCAACTGGCAATGTCACCAGAGAAACCGACTTCTTCCAGCAAGGCGGCGATAGCCTGCTGGCGACCCGTCTGACCGGGCAACTTCATCAGGCAGGTTATGAAGCGCAATTAAGCGACCTGTTTAATCATCCCCGGCTGGCGGATTTTGCCGCCACGCTGCGGAAAACCGACGTCCCGGTCGAACAACCATTCGTCCACTCCCCTGAAGAACGCTACCAGCCCTTTGCGCTTACCGACGTGCAGCAGGCTTACCTGGTGGGGCGTCAGCCGGGCTTTGCCCTGGGCGGCGTCGGCTCACATTTCTTTGTTGAATGTGAAATTGCCGATCTGGACCTCACCCGGCTGGAGACGGTCTGGAACCGATTAATCGCCCGCCACGATATGCTGCGCGCCATCGTGCGTGATGGACAGCAACAGGTGCTCGAACAGACGCCCCCCTGGGTGATACCCACACACACCCTCCATACGCCTGAAGAGGCGTTGCGGGTGCGCGAAAAACTGGCGCATCAGGTACTCAACCCCGAAGTGTGGCCGGTATTCGATCTCCAGGTCGGATACGTGGACGGGATGCCTGCCCGCCTGTGGCTGTGTCTGGATAACCTGTTGCTTGACGGTCTGAGCATGCAGATCCTGCTGGCGGAGCTGGAGCACGGCTACCGCTACCCGCAACAGCTGCTTCCGCCGCTGCCCGTCACCTTCAGGGATTATCTGCAACAACCCTCGCTACAGTCGCCCAATCCAGATTCTCTGGCATGGTGGCAGGCGCAGCTTGATGATATTCCTCCGGCGCCTGCGTTGCCGCTGCGCTGCTTGCCTCAGGAGGTTGAAACACCGCGCTTCGCCCGCCTGAACGGCGCGCTGGACAGCACGCGCTGGCATCGGCTGAAAAAACGGGCGGCTGACGCCCATCTCACCCCGTCGGCCGTACTGTTGTCGGTGTGGTCAACGGTTCTCTCTGCATGGAGTGCACAGCCTGAGTTCACGCTTAACCTTACGCTTTTCGACAGGCGACCGCTGCACCCGCAAATCAACCAGATTCTGGGCGATTTCACCTCGCTGATGCTGCTGAGCTGGCATCCCGGCGAAAGCTGGCTGCACAGCGCGCAGTCACTACAGCAGCGGCTGAGCCAGAACCTCAACCACCGCGATGTGTCAGCCATCCGCGTGATGCGTCAACTGGCGCAACGGCAAAACGTGCCTGCCGTTCCGATGCCCGTCGTCTTTACCAGCGCGCTGGGCTTTGAGCAGGATAACTTCCTCGCCCGGCGTAATCTGCTCAAACCGGTCTGGGGCATCTCCCAGACGCCGCAGGTCTGGCTCGATCACCAGATTTATGAATCCGAAGGCGAACTGCGCTTTAACTGGGATTTTGTCGCCGCGCTGTTTCCTGCCGGGCAGGTGGAGCGCCAGTTTGAACAGTATTGCGCATTGCTAAACCGAATGGCCGAGGATGAAAGCGGCTGGCAACTGCCGCTCGCCGCGCTGGTGCCTCCCGTTAAACACGCAGGGCAATGCGCAGAGCGCTCACCGCGCGTATGCCCTGAGCACTCTCAGCCACACATTGCGGCGGACGAGAGCACCGTCAGCCTGATTTGCGACGCCTTCCGCGAGGTGGTTGGCGAGTCTGTCACGCCCGCAGAAAACTTCTTTGAGGCGGGCGCAACGTCGCTGAATCTGGTGCAACTGCACGTTTTGTTACAACGTCACGAATTTTCCACCCTGACGTTGCTTGACCTCTTCACCCACCCTTCTCCTGCTGCCCTGGCCGATTATCTGGCCGGCGTCGCCACGGTGGAGAAAACAAAACGACCTCGCCCTGTTCGCCGTCGTCAGCGGCGGATATAG

>IR1230_00396

ATGGATAACTTGCGCTTCTCTTCTGCGCCGACAGCAGATTCCATTGATGCATCGATCGCTCAACACTACCCGGACTGCGAACCTGTCGCGGTTATCGGCTACGCCTGCCATTTTCCTGAATCGCCGGATGGCGAAACGTTCTGGCAAAATCTGCTGGAAGGTCGTGAATGCAGCCGACGCTTTACGCGCGAAGAGCTTCTGGCCGTCGGTCTGGATGCCGCCATCATTGACGATCCTCATTATGTCAATATCGGTACGGTGTTAGACAACGCCGACTGCTTCGACGCCACCCTGTTTGGCTATTCGCGACAGGAAGCGGAGTCGATGGACCCGCAGCAGCGCCTATTTTTGCAGGCGGTCTGGCATGCGCTGGAACATGCCGGTTATGCCCCCGGCGCCGTCCCCCATAAGACCGGCGTTTTCGCCTCTTCCCGGATGAGTACCTACCCCGGTCGCGAAGCATTGAACGTGACAGAAGTCGCGCAGGTAAAAGGTCTGCAATCTCTGATGGGCAATGATAAAGACTATATTGCCACCCGCGCCGCGTACAAACTCAACCTGCACGGCCCGGCGTTATCGGTACAGACCGCCTGCTCCAGCTCGCTGGTTGCAGTGCATCTGGCCTGTGAAAGCCTGCGCGCAGGCGAATCCGATATGGCGGTTGCCGGCGGCGTGGCGCTCTCTTTCCCCCAGCAGGCAGGCTACCGCTACCAGCCCGGAATGATTTTCTCTCCTGATGGTCACTGTCGTCCCTTTGACGCCTCGGCTGAGGGCACCTGGGCCGGTAACGGTCTCGGCTGCGTGGTGCTGCGTCGCCTGAGAGACGCGCTGCTGTCAGGCGATCCGATTATCTCGGTGATCCTCTCCAGCGCGGTCAACAACGACGGCAACAGAAAGGTCGGCTATACCGCCCCTTCCGTCGCAGGGCAACAGGCAGTCATCGAAGAGGCGTTAATGCTGGCGGCCATCGACGACAGGCAGGTAGGTTACATTGAAACCCACGGCACCGGCACACCGCTGGGCGACGCGATTGAAATTGAAGCGTTACGCAACGTCTATGCGCCTCGCCCGCAGGATCAGCGCTGTGCGCTCGGTTCCGTGAAAAGTAACATGGGCCATCTGGATACCGCGGCGGGCATTGCCGGACTGCTGAAAACCGTTCTGGCAGTCAGTCGCGGGCAAATTCCTCCCTTACTGAATTTTCACACCCCCAACCCGGCGCTGAAACTTGAAGAGAGCCCCTTTACCATACCGGTGTCGGCACAGGCATGGCAGGACGAAATGCGCTATGCGGGCGTCTCCTCCTTTGGTATTGGCGGCACCAACTGCCATATGATCGTCGCCTCGCTGCCCGACGCGCTCAACGCGCGCCTCCCCGATACGGATAGCGGCAGAAAAAGTACCGCGCTGCTGCTCAGCGCCGCCAGCGACAGCGCGTTGCGGCGGCTGGCGACGGATTATGCCGGGGCGCTGAGAGAGGATGCGGATGCCAGCTCTCTGGCCTTCACAGCCCTGCACGCGCGCCGTCTCGATCTCCCCTTCCGCCTGGCGGCGCCATTAAACCGTGAAACCGCCGAGGCGCTCAGCGCCTGGGCCGGTGAGAAATCGGGGGCGCTGGTTTACAGCGGCCACGGCGCCAGCGGCAAGCAGGTGTGGCTGTTTACCGGCCAGGGCTCGCACTGGCGCACTATGGGTCAAACGATGTACCAGCACTCAACGGCGTTTGCCGACACGCTGGATCGCTGTTTTTCCGCCTGTAGCGAAATGCTCACGCCGTCACTGCGCGAAGCGATGTTTAACCCCGATTCGGCGCAGCTGGACAATATGGCCTGGGCGCAGCCGGCGATTGTCGCGTTTGAAATCGCGATGGCGGCGCACTGGCGTGCTGAAGGACTGAAGCCAGACTTCGCCATTGGGCATTCCGTCGGTGAATTTGCCGCTGCCGTTGTCTGCGGACACTATACGATTGAACAGGTCATGCCACTGGTTTGTCGGCGCGGCGCGCTAATGCAGCAGTGCGCAAGCGGCGCGATGGTGGCGGTATTTGCAGACGAAGACACGCTGATGCCGCTGGCTCGCCAGTTTGAGCTGGATCTCGCCGCCAACAACGGTACGCAACATACGGTATTTTCCGGGCCGGAAGCCCGTCTCGCGGTATTTTGCGCCACGCTCTCGCAGCATGACATTAACTATCGTCGCCTGAGCGTAACCGGTGCGGCGCACTCCGCTTTACTGGAGCCGATACTCGATCGGTTCCAGGACGCCTGCGCGGGACTGCACGCGGAGCCGGGGCAAATACCGATTATTTCCACGCTCACCGCCGACGTCATTGATGAGTCAACGCTCAACCAGGCGGATTACTGGCGCCGACACATGCGCCAGCCGGTGCGTTTTATCCAGAGTATTCAGGTGGCGCATCAGCTCGGCGCCCGCGTTTTTCTGGAGATGGGGCCCGATGCCCAGTTGGTTGCTTGCGGGCAGCGCGAATACCGCGATAACGCATACTGGATAGCCAGCGCCCGGCGTAACAAAGAGGCGAGCGATGTCCTCAATCAGGCCCTGCTCCAGCTTTACGCTGCCGGCGTCGCCCTACCGTGGGCCGACCTGCTGGCGGGCGATGGACAACGTATCGCTGCGCCATGTTATCCGTTTGATACTGAGCGTTACTGGAAAGAGCGCGTCTCCCCGGCCTGCGAGCCTGCCGACGCAGCGCTGTCTGCCGGGCTGGAGGTGGCGAGTCGCGCCGCGACAGCGCTCGATCTCCCTCGCCTGGAAGCGCTTAAACAGTGCGCCACGCGACTGCACGCCATCTACGTCGATCAACTGGTACAACGCTGTACCGGCGATGCCATTGAGAACGGCGTGGACGCCATGACCATCATGCGCCGTGGACGTCTGCTGCCCCGCTACCAGCAGCTACTCCAGCGCCTGCTGAATAACTGCGTGGTCGACGGCGATTACCGCTGCACCGACGGGCGATACGTCCGCGCCCGCCCCATTGAACATCAACAGCGGGAATCACTGCTGACGGAACTTGCCGGTTATTGTGAAGGTTTTCAGGCTATTCCCGACACCATCGCCCGTGCCGGCGATCGGTTATATGAAATGATGAGCGGCGCGGAAGAACCGGTGGCGATTATCTTCCCGCAAAGCGCCTCCGACGGCGTGGAAGTGCTGTATCAGGAATTCAGCTTTGGCCGCTATTTCAACCAAATCGCCGCCGGGGTATTACGCGGCATTGTCCAGACGCGTCAGCCCCGCCAGCCGTTGCGTATTCTTGAAGTTGGCGGCGGAACCGGCGGCACCACCGCGTGGCTGCTGCCGGAACTCAACGGCGTTCCGGCACTGGAGTACCATTTCACCGATATCTCGGCGCTGTTCACCCGTCGCGCCCAGCAGAAATTCGCCGACTATGATTTTGTGAAGTATAGCGAGCTGGATCTCGAAAAAGAGGCGCAGTCTCAGGGTTTCCAGGCACAGTCTTACGATCTTATCGTGGCAGCGAACGTGATTCACGCCACCCGCCATATTGGCCGCACGCTCGATAATCTGCGCCCCCTGCTCAAGCCGGGCGGGCGCCTGCTGATGCGCGAAATCACCCAGCCAATGCGTCTGTTTGACTTCGTTTTCGGCCCGCTGGTTCTTCCGCTACAGGATCTCGACGCCCGCGAAGGTGAGTTATTCCTCACCACCGCTCAGTGGCAACAACAGTGCCGCCACGCCGGATTCAGCAAAGTGGCGTGGCTACCGCAGGATGGCAGCCCGACCGCCGGGATGAGCGAACATATCATTCTCGCCACGCTGCCCGGTCAGGCGGTTAGCGCCGTAACATTCACCGCGCCATCAGAACCCGTGTTGGGGCAGGCGCTGACGGATAACGGTGATTATCTCGCCGACTGGTCTGATTGCGCAGGTCAGCCCGAACGGTTTAACGCCCGCTGGCAGGAGGCCTGGCGTCTGCTTTCACAGCGTCATGGCGACGCTCTACCTGTGGAACCGCCCCCCGTCGCCGCCCCGGAGTGGCTGGGGAAGGTTCGCTTAAGCTGGCAAAACGAAGCCTTTTCCCGCGGTCAGATGCGCGTTGAAGCCCGTCATCCTGCTGGCGAGTGGCTGCCGCTATCGCCCGCCGCGCCTCTTCCTGCGCCGCAGACGCATTATCAATGGCGCTGGACGCCCCTCAACGTCGCCAGCATTGACCATCCGCTTACCTTTAGCTTCAGCGCCGGTACGCTTGCGCGCAGCGACGAGCTGGCGCAATACGGCATCATTCACGATCCGCACGCCTCTTCGCGACTGATGATTGTTGAGGAGAGCGAGGATACGCTGGCCTTAGCGGAGAAAGTGATAGCAGCGCTCACCGCCAGCGCAGCCGGATTGATTGTGGTTACTCGCCGCGCGTGGCGAGTCGAGGAAAATGAAGCACTCTCTGCATCCCATCACGCGCTATGGGCCTTGCTTCGCGTCGCGGCCAACGAACAGCCGGAACGGTTGCTTGCCGCCATCGATCTCGCCGAAAACACCCCGTGGGAAACGCTGCATCAAGGGTTGAGCGCAGTCTCACTATCACAGCGCTGGCTCGCCGCACGGGGTGACACCCTTTGGCTCCCTTCACTGGCGCCCAATACGGGATGCGCCGCTGAATTACCGGCAAACGTGTTTACCGGCGATAGCCGCTGGCATCTGGTGACCGGAGCGTTTGGCGGATTAGGCCGCCTTGCCGTGAACTGGCTCAGAGAAAAAGGGGCGCGACGCATCGCCCTGCTGGCGCCGCGCGTGGATGAGTCATGGCTACGCGACGTGGAGGGCGGGCAGACGCGCGTCTGCCGTTGTGATGTGGGCGATGCCGGGCAACTGGCCACGGTTCTTGACGATCTGGCGGCCAACGGCGGCATTGCCGGAGCGATTCATGCCGCTGGCGTATTGGCTGACGCGCCCTTGCAGGAGCTTGATGACCACCAGTTGGCTGCCGTTTTCGCGGTAAAAGCGCAGGCGGCAAGCCAGCTGTTGCAAACCCTGCGCAACCACGACGGACGCTATCTTATTCTCTACTCTTCCGCTGCCGCCACCCTCGGCGCGCCGGGTCAGAGCGCCCATGCGCTGGCCTGCGGCTACCTGGACGGGCTGGCCCAGCAGTTTTCCACCCTTGATGCGCCGAAAACGCTCTCTGTCGCCTGGGGCGCATGGGGAGAAAGCGGTCGGGCGGCCACGCCGGAAATGCTGGCGACGCTCGCCAGCCGAGGTATGGGCGCGTTAAGCGATGCCGAAGGCTGCTGGCACCTGGAACAGGCGGTGATGCGCGGCGCCCCGTGGCGACTGGCGATGCGCGTTTTTACCGACAAAATGCCCCCGTTACAACAGGCTCTGTTTAACATCAGCGCCACAGAAAAAGCCGCAACGCCGGTCATTCCTCCTGCTGATGACAACGCCTTTAACGGCAGCCTGAGCGATGAAACAGCGGTGATGGCATGGCTGAAAAAGCGGATTGCGGTTCAGCTAAGGCTGAGCGATCCGGCGTCACTGCATCCAAACCAGGATCTGTTGCAACTCGGCATGGACTCGCTGCTCTTCCTTGAACTCAGTAGCGATATTCAGCACTACCTGGGCGTACGCATCAATGCGGAACGGGCGTGGCAGGATCTGTCTCCTCATGGACTCACGCAGCTTATCTGTTCTAAGCCAGAGGCGACGCCTGCCGCTTCGCAGCCGGAAGTGTTGCGGCACGACGCCGACGAGCGTTATGCGCCCTTCCCTTTGACGCCCATTCAGCACGCCTACTGGCTGGGGCGAACCCACCTCATTGGCTATGGCGGCGTCGCCTGTCACGTCCTGTTTGAGTGGGATAAACGCCACGATGAGTTCAATCTCGCCATACTGGAGAAAGCATGGAACCAGCTCATCGCACGCCACGATATGTTGCGTATGGTGGTTGATGCCGACGGGCAGCAGCGAATCCTGGCGACAACGCCGGAGTATCACATCCCGCGTGACGATCTGCGCGCGCTTTCCCCGGAAGAACAGCGCATCGCGCTGGAAAAACGGCGGCATGAACTGAGCTATCGCGTTTTGCCTGCCGACCAGTGGCCTCTTTTTGAGCTGGTGGTCAGCGAAATCGACGATTGCCATTACCGTCTGCATATGAACCTCGACCTTTTGCAGTTTGATGTGCAGAGTTTTAAAGTCATGATGGACGACCTGGCGCAGGTCTGGCGCGGTGAAACGCTGGCGCCGCTCGCTATTACCTTCCGTGATTATGTGATGGCTGAACAGGCGCGCCGACAGACATCGGCATGGCACGATGCCTGGGATTACTGGCAGGAAAAACTGCCGCAACTGCCCTTAGCGCCAGAGCTGCCGGTGGTTGAGACGCCCCCGGAAACGCCACACTTCACCACCTTCAAATCGACGATCGGCAAGACAGAATGGCAGGCCGTGAAACAGCGCTGGCAGCAGCAAGGCGTCACACCGTCTGCCGCGCTGCTCACGCTGTTTGCCGCCACCCTTGAGCGCTGGAGCCGTACCACAACATTTACGCTGAACCTGACGTTCTTCAATCGCCAGCCGATCCATCCGCAAATCAACCAGTTGATTGGTGATTTTACCTCCGTCACGCTGGTTGATTTTAACTTCTCAGCGCCGGTGACGTTGCAAGAGCAGATGCAACAGACCCAACAGCGCCTCTGGCAAAACATGGCGCACAGTGAAATGAACGGTGTTGAGGTGATCCGTGAGCTGGGCCGCCTGCGCGGATCACAACGTCAACCGCTGATGCCGGTAGTGTTTACCAGTATGCTGGGGATGACGCTGGAAGGCATGACTATCGATCAGGCGATGAGCCATCTGTTCGGCGAACCCTGCTATGTATTCACGCAAACGCCGCAGGTCTGGCTGGATCATCAGGTCATGGAGAGCGACGGCGAGTTGATGTTTAGCTGGTACTGCATGGACAACGTGCTGGAACCCGGCGCTGCCGAGGCGATGTTTAATGACTATTGCGCCATCCTGCAAGCCGTCATCGCCGCCCCTGAAAGCCTGAAGACTCTCGCCAGCGGCATCGCCGGGCACATTCCCCGCCGACGCTGGCCGCTGAACGCGCAGGCGGACTACGACCTGCGGGATATTGAGCAGGCGACGCTCGAATACCCCGGCATCCGGCAGGCCAGAGCGGAAATAACCGAACAGGGCGCGTTGACGCTGGATATCGTGATGGCCGACGATCCGTCGCCATCAGCGGCGATGCCTGATGAGCACGAACTTACCCAACTGGCGCTGCCGTTGCCTGAGCAGGCGCAGCTTGATGAGCTGGAGGCGACCTGGCGCTGGCTGGAGGCGCGTGCGCTACAGGGGATCGCGGCTACGCTAAATCGTCACGGCCTGTTTACCACGCCGGAGATCGCCCATCGCTTTAGCGCAATAGTACAGGCGCTGTCCGCGCAAGCGTCTCACCAGCGTCTGCTGCGCCAGTGGCTACAGTGTCTGACGGAAAGAGAGTGGTTAATCCGCGAAGGTGAAAGCTGGCGCTGCCGCATTCCGCTCAGCGAGATTCCTGAGCCTCAGGAAGCGTGCCCGCAAAGCCAATGGAGCCAGGCGCTGGCGCAGTATCTGGAAACCTGCATCGCCCGGCACGACGCCCTCTTCTCCGGGCAGTGTTCTCCGCTGGAATTGCTGTTCAACGAGCAGCATCGCGTTACCGACGCGCTGTATCGCGACAACCCCGCCAGCGCCTGTCTGAATCGCTATACCGCGCAGATTGCCGCCTTGTGCAGCGCAGAACGGATTCTGGAGGTTGGCGCCGGAACCGCAGCCACTACCGCGCCGGTGCTGAAGGCCACGCGGAACACGCGGCAGTCGTACCACTTCACGGACGTCTCCGCGCAGTTCCTCAATGACGCCAGAGCCCGTTTCCATGATGAATCGCAGGTGTCTTATGCCTTGTTCGACATCAACCAGCCGCTGGATTTCACCGCCCACCCGGAGGCGGGTTACGACCTGATCGTTGCCGTCAATGTGCTCCACGACGCCAGCCATGTCGTCCAGACGTTGCGCAGATTAAAACTGTTGCTGAAAGCCGGCGGACGTTTGATGATCGTTGAAGCGACGGAGCGAAACAGCGTATTCCAGCTGGCGAGCGTGGGCTTTATTGAGGGATTAAGCGGATACCGCGATTTCCGCCGCCGGGATGAGAAACCGATGCTCACCCGCTCCGCATGGCAGGAGGTTCTCGTTCAGGCCGGGTTTGCAAACGAGCTGGCGTGGCCCGCGCAGGAATCGTCGCCGCTGCGCCAGCATCTGCTGGTGGCGCGTTCGCCTGGCGTAAATCGCCCGGATAAAAAAGCCGTGAGCCGCTATTTACAGCAGCGCTTTGGCACCGGTCTGCCCATTTTACAGATCCGGCAAAGAGAAGCGTTATTTACGCCGCTGCATGCCCCGTCTGATGCGCCGACTGAGCCAGCCAAACCCACGCCAGTTGCCGGGGGGAATCCGGCGCTGGAAAAACAGGTGGCTGAACTCTGGCAATCGCTGCTGTCTCGCCCCGTGGCAAGGCATCACGACTTTTTCGAACTGGGCGGCGACAGCCTGATGGCGACAAGGATGGTCGCGCAGCTAAACCGGAGAGGGATTGCCAGGGCTAACCTTCAGGATCTGTTCAGCCATTCGACGCTGAGCGACTTCTGCGCCCATCTACAGGCGGCTACGTCAGGAGAGGACAACCCGATACCCCTTTGCCAGGGCGACGGTGAGGAAACCCTGTTTGTCTTCCACGCTTCAGACGGCGATATCAGCGCCTGGCTGCCGCTCGCTAGCGCGTTGAACAGGCGCGTTTTCGGCCTGCAAGCAAAATCGCCGCAGCGCTTTGCCACGCTCGACCAGATGATCGATGAGTATGTCGGGTGCATCCGTCGTCAGCAGCCTCACGGCCCTTATGTGCTGGCGGGTTGGTCGTATGGCGCGTTTCTTGCGGCGGGCGCCGCACAGCGCCTGTACGCCAAAGGCGAGCAGGTTCGGATGGTGTTAATCGATCCCGTGTGCCGACAGGATTTCTGTTGCGAAAACCGGGCGGCCCTGCTGCGCCTGTTAGCCGAAGGACAAACGCCTCTGGCACTGCCCGAACATTTCGACCAGCAGACGCCCGACAGCCAGCTTGCCGACTTTATCAGCCTCGCTAAAACGGCCGGTATGGTGCCGCAAAACCTGACGCTGCAAGCGGCAGAAACGTGGCTCGACAACATCGCGCATCTGCTGCGTTTACTGACTGAGCATACGCCGGGCGAAAGCGTTCCGGTCCCCTGTCTCATGGTGTATGCCGCCGGGAGACCCGCGCGCTGGACGCCAGCAGAAACCGAGTGGCAGGGCTGGATAAACAACGCCGACGACGCTGTGATTGAAGCCAGCCACTGGCAAATCATGATGGAAGCCCCCCACGTTCAGGCTTGTGCGCAACACATTACGCGCTGGCTTTGCGCAACCTCAACGCAACCGGAGAACACGTTATGA

>IR1230_00397

ATGATGCCGTCCGCCTCCCCAAAACAACGCGTACTGATTGTGGGCGCCAAATTTGGCGAAATGTACCTGAATGCCTTTATGCAGCCCCCGGAGGGGCTGGAACTGGTCGGCCTGCTGGCGCAGGGAAGCGCCCGTTCAAGAGAGCTGGCTCATGCGTTTGGCATTCCGCTGTATACCTCGCCGGAACAGATAACCAGGATGCCGGATATCGCCTGTATTGTCGTGCGTTCGACCGTCGCCGGAGGAACCGGTACGCAGCTTGCCAGACACTTTCTGACGCGCGGCGTACACGTCATTCAGGAGCATCCCCTCCACCCGGATGACATCAGTTCCTTACAAACGCTGGCGCAGGAACAGGGCTGCTGCTATTGGGTAAACACGTTTTATCCGCATACGCGCGCGGGACGCACATGGCTTCGCGATGCGCAGCAGCTGCGTCGCTGCCTGGCTAAGACGCCGCCGGTAGTCCACGCCACCACCAGCCGACAGTTGCTCTACTCCACTCTGGATTTGTTGTTGCTGGCTCTGGGCGTTGATGCCGCCGCCGTGGAGTGCGACGTGGTTGGCAGCTTTAGCGATTTTCACTGTCTGAGACTCTTCTGGCCGGAGGGAGAAGCCTGCCTGCTGCTTCAGCGCTATCTCGATCCTGACGATCCAGATATGCATAGCCTTATCATGCACCGCCTGCTGCTGGGCTGGCCGGAAGGTCATCTTTCGCTGGAGGCGAGCTACGGCCCCGTCATCTGGTCATCCAGCCTGTTTGTCGCAGACCATCAGGAGAACGCCCACAGCCTCTACCGCAGACCGGAGATCCTGCGCGATCTGCCGGGTCTGACGCGCAGCGCGGCGCCCCTGAGCTGGCGCGACTGCTGCGAAACCGTCGGGCCGGAGGGCGTCAGCTGGTTGCTACACCAGTTGCGCAGCCATCTGGCGGGCGAACATCCACCAGCGGCCTGCCAAAGCGTACATCAGATCGCGTTATCACGTTTATGGCAGCAGATTTTACGTAAGACCGGCAATGCGGAGATCAGACGCCTGACGCCGCCGCACCACGACCGGTTAGCCGGTTTCTACAACGATGATGATAAGGAGGCGCTGTGA

>IR1230_00398

GTGACGCAATCTGCAATGTGCATCCCGCTGTGGCCCGCCCGAAACGGTAATACTGCGCATCTAGTCATGTGCCCTTTCGCTGGCGGCAGCAGTAGCGCGTTTCGCCACTGGCAAGCTGAGCAACTGGCTGATTGCGCGCTTTCTCTGGTGACCTGGCCGGGGCGCGATCGCCTTCGCCATCTGGAACCGCTCAGAAGCATTACACAACTGGCGGCACTGCTGGCGAACGAGCTGGAAGCATCCGTATCGCCTGACACGCCGCTTTTACTCGCCGGGCACAGCATGGGGGCACAGGTGGCGTTTGAAACCTGCCGACTTCTGGAGCAACGGGGGCTTGCGCCACAAGGACTGATTATTTCCGGGTGCCATGCCCCGCATCTGCATTCTGAACGCCAGCTCAGCCATCGCGATGATGCCGACTTTATCGCTGAGCTGATAGACATTGGCGGATGTTCTCCTGAACTGCGGGAAAACCAGGAATTAATGTCGCTGTTTCTTCCTCTTCTGCGCGCTGATTTTTACGCCACCGAGAGCTATCACTACGACTCGCCCGACGTCTGTCCGCCGCTGCGCACGCCTGCGCTGTTATTGTGCGGCAGCCACGATCGCGAAGCCTCCTGGCAGCAGGTCGATGCCTGGCGTCAGTGGCTGAGCCCCGTTACAGGCCCGGTGGTGATTGACGGCGATCATTTCTATCCCATTCAACAAGCCCGGTCCTTTTTTACGCAGATTGTCCGCCATTTTCCCCACGCATTTTCTGCAATGACCGCGTTGCAAAAACAGCCCAGTACTTCAGAAAGGTGA

>IR1230_00399

ATGAATTCTTCCTTTGAATCTCTGATTGAACAGTATCCCTTACCCATTGCCGAACAGTTGCGCCACTGGGCGGCCCGTTATGCCTCGCGAATTGCCGTCGTTGATGCAAAGGGGTCGTTAACCTACAGCGCGCTTGATGCACAAGTTGACGAACTTGCCGCAGGTCTGTCATCACTGGGTTTGCGTTCGGGGGAGCATGTAATTGTGCAGCTTCCCAACGACAACGCGTTTGTTACCCTGCTGTTCGCCTTGTTAAGACTGGGCGTTATCCCCGTGCTGGCGATGCCCTCGCAACGGGCGCTGGATATCGACGCGCTGATTGAGCTGGCGCAACCCGTCGCTTACGTTATTCACGGGGAAAACCACGCAGAGCTGGCCCGACAGATGGCGCACAAACACGCCTGCTTGCGTCATGTTCTGGTCGCTGGAGAGACCGTGAGCGACGATTTTACGCCGCTCTTCTCCCTTCACGGTGAGCGACAGGCATGGCCGCAGCCTGATGTTTCCGCCACCGCGTTGTTGTTGCTCTCAGGCGGCACAACCGGCACGCCCAAACTCATCCCGCGCCGACATGCCGACTATAGCTATAACTTCAGCGCTTCTGCTGAACTGTGCGGCATCAGCCAACAGAGCGTGTATCTCGCCGTCCTCCCGGTGGCGCATAACTTTCCGCTGGCCTGCCCCGGTATTCTGGGAACGCTTGCCTGCGGCGGAAAAGTGGTGCTGACCGACAGCGCCAGCTGTGATGAGGTGATGCCTTTAATCGCGCAGGAAAGAGTGACTCACGTCGCCCTGGTTCCGGCGCTGGCGCAATTATGGGTGCAGGCCAGGGAGTGGGAAGACAGCGACCTTTCGTCGCTGCGCGTCATTCAGGCAGGCGGCGCCCGGCTCGACCCGACGCTTGCTGAGCAGGTTATCGCCACCTTTGACTGTACCCTGCAACAGGTTTTCGGTATGGCGGAAGGCCTGCTCTGTTTTACCCGGCTGGACGATCCGCATGCCACCATTCTCCACAGCCAGGGGCGCCCGTTGTCCCCTCTGGATGAAATCCGCATCGTTGATCAAGACGAGAACGACGTCGCGCCGGGCGAAACCGGGCAATTGTTAACGCGCGGCCCTTATACCATTTCGGGCTATTACCGCGCCCCTGCCCACAACGCGCAGGCCTTTACCGCGCAAGGGTTTTACCGCACAGGCGACAATGTCAGGCTGGATGAGGTGGGGAACCTGCACGTTGAGGGACGCATAAAAGAGCAGATCAACCGCGCCGGAGAAAAAATAGCCGCGGCTGAAGTGGAATCGGCACTGCTGCGTTTAGCGGAAGTGCAAGATTGCGCGGTGGTCGCCGCGCCGGACACGCTGCTTGGCGAGCGGATTTGCGCGTTTATCATCGCGCAGCAGGTGCCAACTGACTATCAGCAGTTGCGTCAACAACTGACCCGTATGGGGCTCAGCGCGTGGAAAATTCCTGACCAAATCGAGTTTCTGGACCACTGGCCGCTCACCGCCGTCGGCAAGATAGACAAAAAACGCCTGACGGCTCTCGCCGTCGACCGTTATCGCCATTCTGCCCAATAA

>IR1230_00400

ATGAAAATGACACGGCTTTATCCTCTGGCCTTGGGGGGATTATTGCTCCCCGCCATTGCTAATGCCCAAACTTCACAGCAAGACGAAAGCACGCTGGTGGTTACCGCCAGTAAACAATCTTCCCGCTCGGCATCAGCCAACAACGTCTCGTCTACTGTTGTCAGCGCGCCGGAATTAAGCGACGCCGGCGTCACCGCCAGCGACAAACTCCCCAGAGTCTTGCCCGGGCTCAATATTGAAAATAGCGGCAACATGCTTTTTTCGACGATCTCGCTACGCGGCGTCTCTTCAGCGCAGGACTTCTATAACCCCGCCGTCACCCTGTATGTCGATGGCGTCCCTCAGCTTTCCACCAACACCATCCAGGCGCTTACCGATGTGCAAAGCGTGGAGTTGCTGCGAGGCCCACAGGGAACGTTATATGGCAAAAGCGCTCAGGGCGGGATCATCAACATCGTCACCCAGCAGCCGGACAGCACGCCGCGCGGCTATATTGAAGGCGGCGTCAGTAGCCGCGACAGTTATCGAAGTAAGTTCAACCTGAGCGGCCCCATTCAGGATGGCCTGCTGTACGGCAGCGTCACCCTGTTACGCCAGGTTGATGACGGCGACATGATTAACCCCGCGACGGGAAGCGATGACTTAGGCGGCACCCGCGCCAGCATAGGGAATGTGAAACTGCGTCTGGCGCCGGACGATCAGCCCTGGGAAATGGGCTTTGCCGCCTCACGCGAATGTACCCGCGCCACCCAGGACGCCTATGTGGGATGGAATGATATTAAGGGCCGTAAGCTGTCGATCAGCGATGGTTCACCAGACCCGTACATGCGGCGCTGCACTGACAGCCAGACCCTGAGTGGGAAATACACCACCGATGACTGGGTTTTCAACCTGATCAGCGCCTGGCAGCAGCAGCATTATTCGCGCACCTTCCCTTCCGGTTCGTTAATCGTCAATATGCCTCAGCGCTGGAATCAGGATGTGCAGGAGCTGCGCGCCGCAACCCTGGGCGATGCGCGTACCGTTGATATGGTGTTTGGGCTGTACCGGCAGAACACCCGCGAGAAGTTAAATTCAGCCTACGACATGCCGACAATGCCTTATTTAAGCAGTACCGGCTATACCACCGCTGAAACGCTGGCCGCATACAGTGACCTGACCTGGCATTTAACCGATCGTTTTGATATCGGCGGCGGCGTGCGCTTCTCGCATGATAAATCCAGTACACAATATCACGGCAGCATGCTCGGCAACCCGTTTGGCGACCAGGGTAAGAGCAATGACGATCAGGTGCTCGGGCAGCTATCCGCAGGCTATATGCTGACCGATGACTGGAGAGTGTATACCCGTGTAGCCCAGGGATATAAACCTTCCGGGTACAACATCGTGCCTACTGCGGGTCTTGATGCCAAACCGTTCGTCGCCGAGAAATCCATCAACTATGAACTTGGCACCCGCTACGAAACCGCTGACGTCACGCTGCAAGCCGCGACGTTTTATACCCACACCAAAGACATGCAGCTTTACTCTGGCCCGGTCGGGATGCAGACATTAAGCAATGCGGGTAAAGCCGACGCCACCGGCGTTGAGCTTGAAGCGAAGTGGCGGTTTGCGCCAGGCTGGTCATGGGATATCAATGGCAACGTGATCCGTTCCGAATTCACCAATGACAGTGAGTTGTATCACGGTAACCGGGTGCCGTTCGTACCACGTTATGGCGCGGGAAGCAGCGTGAACGGCGTGATTGATACGCGCTATGGCGCACTGATGCCCCGACTGGCGGTTAATCTGGTCGGGCCGCATTATTTCGATGGCGACAACCAGTTGCGGCAAGGCACCTATGCCACCCTGGACAGCAGCCTGGGCTGGCAGGCGACTGAACGGATGAACATTTCCGTCTATGTCGATAACCTGTTCGACCGTCGTTACCGTACCTATGGCTACATGAACGGCAGCAGCGCCGTCGCGCAGGTCAATATGGGTCGCACCGTCGGTATCAATACGCGAATTGATTTCTTCTGA

>IR1230_00401

ATGGCAGTACGAAAACTCACCACAGGAAAATGGCTTTGCGAATGTTACCCCGCCGGACGTAGTGGACGTCGTGTGCGTAAACAATTCGCCACCAAAGGCGAAGCTCTGGCTTTTGAGCGTCACACGATGGAAGAAACCGAAGCAAAGCCCTGGCTGGGTGAATCAGTGGATCGTCGAACACTGAAAGACGTGGTTGAACTATGGTTCAAACTACATGGTAAATCTCTGACTGCTGGGCAGCATGTCTATGACAAATTGCTGCTGATGGTTGACGCTCTGGGCAATCCCCTTGCAACCGATCTCACATCTAAAATGTTTGCCCATTATCGAGATAAACGACTGACAGGTGAGATCTACTTCAGCGAGAAATGGAAGAAAGGAGCCAGCCCGGTCACCATTAACCTGGAGCAAAGCTATCTAAGTAGTGTTTTTAGCGAACTATCCCGTCTGGGCGAATGGTCGTATCCGAACCCACTGGAGAACATGCGAAAATTCACCATCGCAGAAAAAGAGATGGCATGGCTTACCCATGAGCAGATTGTTGAATTGCTGGCTGATTGCAAACGTCAGGACCCAATTCTGGCACTGGTAGTTAAGATATGCTTAAGCACAGGCGCACGCTGGCGTGAAGCCGTAAATCTTACCCGCTCACAGGTGACCAAATACCGAATTACCTTTGTCAGAACAAAAGGGAAGAAAAACAGAAGCATCCCTATCAGTAAAGAGCTTTACGAAGAGATCATGGCGCTTGATGGGTTCAATTTCTTCACAGACTGTTATTTTCAATTTTTATCCGTGATGGAAAAAACGTCTATCGTGCTCCCTCGCGGTCAACTCACACACGTTCTGCGCCATACGTTTGCGGCGCACTTCATGATGTCGGGTGGAAACATTCTGGCCTTACAAAAAATTCTCGGACACCACGATATAAAAATGACTATGCGTTACGCACATCTGGCACCGGATCATCTGGAAACGGCGCTCCGTTTCAATCCTCTGGCAACGCTGCCAAGAGGCGACAAAGTGGCGGCAGCGGTTGGCATTACCCCGTAA

>IR1230_00402

ATGGCCCGTAGACCAAACGATCCGCAGCGCCGCGAGCGCATCCTGCAGGCGACGCTGGATACCATCGCGGCGCACGGCATCCATGCGGTGACGCACCGTAAAATCGCCACCTGCGCGAATGTGCCGCTGGGATCGCTGACCTACTATTTCAGCGGCATTGAGGCGTTAATTGAGGAGGCGTTCAGCCTGTTTACCGCCGAGATGTCGGCGCAGTATCAGCAAGGCTTCGTCGGGGTAACGAACCGGGAACAAGCCTGCGATGCCATCGCGGATCTGATCTTCAGCGCCCAGGTGACCACGGCGCGCAATATGGAGCTGATGTACCAGCTGTACGCGTTCTGCAGCAGTCAGCCGGCGCTGAAGGCGGTGATGCAGAACTGGATGCGGCGCAGCCAGCAGACGTTGGAGCAGTGGTTCGCCCCCGATACCGCCCGCGGTCTCGACGCCTTTATTGAAGGGATGACCTTGCACTTTGTCACCGACCGCGCGCCGCTGTCGAAGGCGGCAATCCGCATGATGGTCGGGCAGCTGGCGGGGGAGAGGGCGCAGGAAGAGGGGCGCTGA

>IR1230_00403

ATGACGACGCAATCCTCGCGCAGAGCGCTGCAGCTTCGGCTATGGGCGCTGTTTATGTTCTTTTTCATCCCCGGCTTGTTAATGGCCTCCTGGGCCACCCGCACGCCGGCGATCCGCGATCTGTTGGCGCTATCCACCGCAGAGATGGGCGTGGTGCTGTTTGGTCTGTCGGTGGGGTCGATGAGCGGTATTCTCTGCTCGGCGTGGCTGGTGAAGCGCTTTGGCACCCGCAAGGTCATCCGCACCACCATGTCCTTCGCCGTGCTTGGCATGCTGGTGCTCAGCCTGGCGCTGTGGGTCACTTCCGCCCCGCTGTTCGCCTTCGGCCTGGCCATCTTCGGCGCCAGCTTTGGCTCTGCAGAAGTCGCTATTAACGTCGAAGGCGCGGCGATCGAGCGGGAGATGAATAAAACGGTGCTGCCGATGATGCATGGCTTCTACAGCTTTGGCACCCTGTTCGGCGCCGGGGTGGGCATGGCAGTGACCGGATTCGGTCTGCCGGCCGCCCCGCACATCCTGGCGGCTGCGCTGGTGGCTATCCTGCCCATCGCCATTGCTATCCGCGCCATTCCCGACGGCACCGGTAAAAATGCCGCCGAAGTCGCCCACGGTGAGGCGAAAGGTCTGCCGGTGTGGCGCGACGCGCAGCTGCTGCTGATCGGCGTCATTGTCCTGGCGATGGCCTTTGCCGAAGGCTCGGCCAACGACTGGCTGCCGCTGCTGATGGTGGATGGCCACGGCTTTAGCCCCACCTCCGGCTCCCTGATTTATGCCGGTTTCACCCTCGGCATGACGCTGGGCCGCTTTACCGGCGGCTGGTTTATCGATCGCTACAGCCGGGTGGCGGTGGTCCGCGGCAGCGCAGTGATGGGCGCCCTCGGCATAGGCCTGATCATCTTCGTCGATAACCCCTGGGTGGCGGGCATCTCGGTGCTGCTGTGGGGCATTGGCGCCTCGCTCGGCTTCCCGCTGACCATCTCCGCCGCCAGCGATACCGGGCCGGACGCGCCCAAGCGCGTCAGCGTGGTGGCGATCACCGGCTATCTCGCTTTCCTCGTCGGGCCGCCGCTGCTGGGTTTCCTCGGCGAACACTTCGGCCTGCGCAGCGCCATGATGGTGGTGCTGGGTCTGGTGATGGTGGCGGCGCTGGTCGCCCGCGCGGTGGCTAAGCCACAATCTGAACCCGTTATGGAGAACAGCTAA

>IR1230_00404

ATGAGCATCAAACTGATTGCGGTGGATATGGACGGCACCTTTTTAAGCGATGCCAAAACTTACAACCGGCCGCGCTTCCTCGCCCAGTACCAGCGAATGCGCGAACAGAATATTCGCTTCGTGGTCGCCAGCGGCAACCAATATTATCAGCTGATCTCCTTTTTCCCGGAGATCGCCCATCAGATCGCCTTCGTGGCGGAAAACGGCGGCTGGGTGGTGAGCGGCAATGAAGACGTCTTCAACTGCCAACTGCCGGTTCACCACTTCAACGCCGTGGTCGACCATCTGCAGACGTTGCCGAATATCGAAATCATCGCCTGCGGGAAGCGCAGCGCCTACACCCTCAATCGCTACAACGACGCGCTGAAGACGGTGGCGGCGAAGTATTATCACCGCCTTGAACTGGTGGATGATTTTAACCATCTCGATGACACCATCCTCAAATTTGGTCTCAACGTGCCAGACAGCCTGATCCCGGAAATTCAGCCGAAGCTGCACGCGGCGCTGGGGGACATGGTCACCGCGGTGGCCACCGGCTACGGCAGCATCGACTTAATTATCCCGGGTGTGCACAAAGCCAACGGGCTGCGCATTTTGCAGCAGCGCTGGGGCATTGAGGATCACGAGGTAGTGGCGTTCGGCGACAGCGGCAACGATATCGAAATGCTGCAGCACGCCGGGTTTGGCTTTGCCATGGCCAACGCCCGGGAAGACGTCAAAGCCGTCGCCCGCTATCAGGCGCCGCATAACAACGAAGAAGGTGTCCTGCAGATTATTGATAAGGTGTTGGATCGCGAAGCGCCGTTCGCCTGA

>IR1230_00405

ATGCAAAATTATTCGCTTTCAGGCCGCCGCCTCGGAAGGCAGGCGCTGTTGTTCCCGTTATGTCTGGTGCTGTACGAGTTTTCCACTTATATCGGCAACGATATGATTCAGCCGGGCATGTTAGCCGTGGTGCAGGAATTTCAGGTGGGCAACGAATGGGTACCTACTTCAATGACTGCCTATCTGGCAGGCGGCATGTTTTTACAGTGGCTGCTGGGGCCGCTGTCGGATCGTATTGGCCGCCGTCCGGTGATGTTGACCGGCGTAGTATGGTTTATCGTCACCTGCCTGGCGACGCTGCTGGCGCAGACCATTGAGCAGTTTACCCTGCTGCGCTTCCTGCAGGGGATCAGCCTGTGCTTTATCGGCGCGGTGGGCTATGCGGCGATACAGGAGTCCTTTGAGGAGGCGGTGTGTATCAAAATCACCGCCCTGATGGCTAACGTGGCGCTGATTGCCCCGCTGCTGGGGCCGCTGGTGGGCGCCGCCTGGGTGCATGTCCTGCCGTGGGAGATGATGTTTGTCCTGTTCGCCGTGCTGGCGGCCATCTCGTTCTTCGGCCTGCAGCGGGCGATGCCGGAGACCGCGACGCGGCTGGGCGAAAAGCTGTCGGTGAAAGAGCTGGGCCGCGACTATCGGCTGGTGCTGAAGAACCTGCGCTTCGTCGCCGGCGCGCTGGCCACTGGCTTTGTCAGCCTGCCGCTGCTGGCGTGGATTGCCCAGTCGCCGGTGATTATTATCAGCGGCGAACAGGCCACCAGCTATGAGTACGGCATGCTGCAGGTGCCGATTTTCGGGGCGCTGATTGCCGGCAACCTGGTGCTGGCGCGCCTGACTGCGCGTCGGACCGTGCGCTCGCTGATTATCATGGGCGGTTGGCCGATCATGTTTGGCCTGATCCTCTCTGCGGCGGCGACCGTGGTCTCTTCCCACGCTTATCTGTGGATGACGGCCGGTCTGAGCTTCTACGCCTTCGGGATTGGCCTCGCGAACGCCGGCCTGGTGCGTTTAACCCTGTTCGCCAGCGAGATGAGTAAAGGCACGGTCTCGGCGGCGATGGGAATGCTGCAGATGCTGATCTTCACCGTCGGCATCGAGCTCAGCAAACATGCTTACGAGCTGGGCGGCAACGGGCTGTTCAGCCTGTTTAACCTGCTGGGTGGGGTACTATGGCTGGGGCTGATGATTTACTTTCTGAAAGATAAAAGCGTCGGCAATTCGCAGCAGGGGTAG

>IR1230_00406

ATGCTGGAAAATATTAACTATGCGCTGTTCGCCCTGCTCAACGCGACGCCTGCCTCGCCGCAGTGGGCCATTGAGATGGCGATCTTCATCGCTAAAGATCTGATTCTGATCGTGCCTCTGCTGGTGGTGACATTGTGGCTGTGGGGGCCTGCCCAGCGGCAGATGGTGTTTAAGCTGATGCTGGCGCTGACGATCAGCCTGACCGTGTCGTGGGCGATCGGCCACCTCTATCCGCACGATCGGCCGTTTGTCGCCGGCGTAGGCTATAACTTCCTGCACCACGCGGCGGACGACTCCTTCCCCAGCGACCATGGCACCGTCAGCTTTACCTTTGCCCTGGCCTTCCTGTTCTGGCATCGCCTGTGGTCCGGCGCCCTGCTGATGGCCATTGCCGCCGCCATCGCCTGGTCCCGTGTCTATCTCGGCGTGCACTGGCCGCTGGATATGGTCGGCGGGCTGCTGGCCGGGATGTGCGGCTGCCTTGGCGCGGCGCTGATCTGGCACACGTTCGGCCCGGCGCTATACCATCAGCTGCAGCGGCTGTATCGTCTCTGCTTCTCCTTGCCAATCCGCAAAGGCTGGGTGCGCGGCTAA

>IR1230_00407

ATGGAAACTCGCCGTGACGAACGTATCAGTCAGCTCATTCAGGCACTTAAACGCAGCGATAAGCTGCATCTGAAAGAAGCCGCTTCCCTGCTTGGGGTGTCGGAAATGACCATTCGTCGCGACCTCAATGGCCACAGCGGCCCGGTTGTGCTGCTCGGCGGATACATTGTGCTGGAGCCGCGCAGCGCCACCCATTATCTGCTCAGCGATCAAAAAACCCGGCTGGTCGAGGAAAAACGCCGTGCCGCTCGCCATGCGGCCGCGCTGCTGGAGGCCCATCAGATGGCCTTCTTCGACTGCGGGACCACCACGCCGTGGATTATTGACGCCATTGACGATGCCCTGCCCTTCACCGGCGTCTGCTACTCCCTGAACACCTTTCTGGCCCTGCAGGAAAAACCCCAGTGCCGCGCGGTCCTGTGCGGTGGGGAGTTTCATGCCAGCAACGCGATTTTTATGCCGCTGAGCCTTGAGGATACCCTCAGCCATCTGAGCCCTGATATCGCCTTTTACTCGGCGGCGGGGATCGACTGCGAGCAGGGGGCGACCTGCTATAACCTGGAAGAGCTGCCGGTTAAGCACTGGGCGATGCGTCACGCGCGCTATCACGTGCTGGTGGTCGACCACAGCAAGTTTGGCAAAGTGCGTCCGGCGCGCATGGGGGCGTTAGCGAAATTTGACGTGATTGCCAGCGATATCTGCCCGGATGATGAGCTGGTCGCGCTGGCGAAGGCGCAGCAGATTTCCCTACTGTATTGA

>IR1230_00408

GTGGCTCTTATGATGCATGACGCTTTTTCCCTTCGCGGCCTCGCGGCAGGTTGCGCGCTGTTATTTCTTGTCGCACCTGCGGTGCAGGCTGCGGAACAACTCCCCGACGCCCCTTCAATTGACGCTCGCGCCTGGATCCTGATGGACTATGCCAGCGGGAAGGTGCTCAGCGAAGGCAATGCCGATGAAAAACTCGACCCGGCCAGTCTGACGAAGATCATGACCAGCTACGTGGTAGGGCAGGCAATAAAAGCGGGAAAAATCAAACTGACCGATATGGTGACCGTTGGGCGCGATGCCTGGGCGACCGGCAACCCGGCGCTGCGCGGCTCATCGGTGATGTTCCTCAAGCCTGGCATGCAGGTTTCCGTAGAAGATCTGAACAAAGGGGTCATCATTCAGTCCGGTAACGACGCCAGCATTGCGATTGCTGACTACGTAGCGGGCAGCCAGGACGCTTTCGTTAGCCTGATGAACGGCTACGCCAAAAAGATGGGGCTGACCAACACCACCTTTATGACCGTCCACGGCCTTGACGCGCCGGGGCAGTTCAGCACCGCCCGCGATATGGCGCTGCTGACCAAAGCGCTGATCCACGACGTACCGGAAGAGTACGCGGTACATAAAGAGAAAGAGTTCACTTTCAATAAAATTCGCCAGCCGAACCGCAACCGCCTGCTGTGGAGCAGCAACCTCAACGCCGATGGCGTGAAAACCGGGACCACCGCCGGGGCCGGCTATAACCTCGTCTCCTCGGCCACTCAGGGTGATATGCGTTTGATCGCCGTGGTGCTGGGGACCAAAACCGACCGCATTCGCTTTAACGAGTCAGAAAAACTGCTGACCTGGGGCTTCCGCTTCTTTGAAACCGTGACGCCGATTAAACCAGATGCCACCTTCGTTACCCAGCGCGTGTGGTTTGGCGATAGCAATGAAGCGAAACTGGGGGCTGGCGAGGCGGGCTCTATCACTTTGCCGAAGGGCCAGCTGAAAAACCTGAAAGCCAGCTATACCTTAAATCAGCCGCAGCTTACCGCGCCGCTGGAGAAGGGGCAGGTGGTCGGGACTATCGACTTTAAGCTAAATGATAAAACCATTGAACAGCGTCCGCTGATTGTCATGGAGTCGGTAAAAGAGGGCGGCTTCTTCAGCCGGATGATCGACTTCGTGCTGATGAAACTGCACGGCTGGTTCGGCAGCTGGTTCTCCTGA

>IR1230_00409

ATGATTACACTGTGGGGACGTAATAACTCGACCAACGTGAAGAAAGTACGCTGGGTGCTGGAGGAGCTGGATCTGCCTTATCAGCAGATCCTCGCCGGGCTGGAGTTTGGTCTCAACCATGACCCGGAATATCTGGCGATGAACCCGAACGGGCTGGTGCCGCTGTTAAAGGACGATGCCACCGGGGTGGTAGTGTGGGAATCAAATACGATTATTCGCTACCTGGCGGCCCAGTATGGCGTTGACCGTCTGTGGCTTGCCGCGCCGGCGCAGCGCGCGCAAGGGGAAAAGTGGATGGATTGGTCCAACGGCACCCTCTCACCCGCCCATCGCCCGGTATTAATGGGCCTGGTGAGAACGCCGCCGGAGCAGCGCGACCCGGCGGCCATCGCCGCGGGGATCAGCGCCTGCGAAGCGCTGTTCGCCATGCTGGATGACGAACTGGCCAAAATGCCGTGGCTCTCCGGCGAGCAGTTCGGCCTTGGCGATATTGCCGTCGCTCCCTTCGTCTACAATCTGTTGTCGATCCTCGACAGCTGGCAACCGCGGCCCCATCTGCAGCGCTGGTATCAGCAGATTAGCCAACGTCCTGCCTGGCGCGAAGTAGTGCAGATCCCGGTGACCTGA

>IR1230_00410
[truncated: 4,815,925 more chars]
